# A 7-lncRNA signature associated with the prognosis of colon adenocarcinoma

**DOI:** 10.7717/peerj.8877

**Published:** 2020-04-10

**Authors:** Xiaorui Fu, Jinzhong Duanmu, Taiyuan Li, Qunguang Jiang

**Affiliations:** 1 Queen Mary School of Medical Department, Nanchang University, Nanchang, Jiangxi, China; 2 Department of Gastrointestinal Surgery, The First Affiliated Hospital of Nanchang University, Nanchang, Jiangxi, China

**Keywords:** Long noncoding RNA, Prognosis, Nomogram, The Cancer Genome Atlas, Colon carcinoma

## Abstract

**Background:**

Colon adenocarcinoma (COAD) is the most common colon cancer exhibiting high mortality. Due to their association with cancer progression, long noncoding RNAs (lncRNAs) are now being used as prognostic biomarkers. In the present study, we used relevant clinical information and expression profiles of lncRNAs originating from The Cancer Genome Atlas database, aiming to construct a prognostic lncRNA signature to estimate the prognosis of patients.

**Methods:**

The samples were randomly spilt into training and validation cohorts. In the training cohort, prognosis-related lncRNAs were selected from differentially expressed lncRNAs using the univariate Cox analysis. Furthermore, the least absolute shrinkage and selection operator (LASSO) regression and multivariate Cox analysis were employed for identifying prognostic lncRNAs. The prognostic signature was constructed by these lncRNAs.

**Results:**

The prognostic model was able to calculate each COAD patient’s risk score and split the patients into groups of low and high risks. Compared to the low-risk group, the high-risk group had significant poor prognosis. Next, the prognostic signature was validated in the validation, as well as all cohorts. The receiver operating characteristic (ROC) curve and c-index were determined in all cohorts. Moreover, these prognostic lncRNA signatures were combined with clinicopathological risk factors to construct a nomogram for predicting the prognosis of COAD in the clinic. Finally, seven lncRNAs (CTC-273B12.10, AC009404.2, AC073283.7, RP11-167H9.4, AC007879.7, RP4-816N1.7, and RP11-400N13.2) were identified and validated by different cohorts. The Kyoto Encyclopedia of Genes and Genomes analysis of the mRNAs co-expressed with the seven prognostic lncRNAs suggested four significantly upregulated pathways, which were AGE-RAGE, focal adhesion, ECM-receptor interaction, and PI3K/Akt signaling pathways.

**Conclusion:**

Thus, our study verified that the seven lncRNAs mentioned can be used as biomarkers to predict the prognosis of COAD patients and design personalized treatments.

## Introduction

Colon cancer refers to a frequently occurring gastrointestinal malignant tumor, and it remains the third most common cause of cancer mortalities (Siegel et al., 2017). It is estimated that each year, 693,900 deaths and 1.4 million newly diagnosed cases of colon cancer are reported (Torre et al., 2016). The commonest type of colon cancer is colon adenocarcinoma (COAD), accounting for 98% of newly diagnosed cases. The main treatment for COAD is surgical resection accompanied with chemotherapy. The biggest limitation of adjuvant chemotherapy is that drugs are absorbed in the upper gastrointestinal tract before entering the colon; therefore, the delivery of colon-targeted drugs is the emphasis of COAD treatment studies (Banerjee et al., 2017). Despite the considerable advancement of COAD treatment and diagnosis, the prognosis of distant metastasis patients is still poor (Lee et al., 2014). At present, the gold standard for estimation of the risk of cancer metastasis and recurrence is the clinicopathological stage. However, many patients with the same stage can have different clinical results, suggesting that the conventional assessment method of colon cancer prognosis is unable to predict it precisely. Therefore, to improve the accuracy of diagnosis and provide directions for personalized treatment of COAD, it is crucial to discover novel prognostic biomarkers and more precise methods that can differentiate between patients with low and high risks of poor prognosis.

Long non-coding RNA (lncRNA), covering over 200 nucleotides, is a subclass of noncoding RNAs, widely distributed in the genome and is able to regulate the expression of genes (Quinn & Chang, 2016). Previous studies have demonstrated that lncRNAs participate in diverse cellular biological functions (e.g., changing chromosome conformation, imprinting genomic loci, and modulating post-transcription) (Ponting, Oliver & Reik, 2009). Although the major function of lncRNAs have not been completely understood, there are accumulating evidence that lncRNAs play a significant role in human carcinogenesis by influencing pathways related to oncogenes and tumor suppressors (Hibi et al., 1996; Ji et al., 2003). The abnormal expression of lncRNAs can alter these biological processes to enhance neoplasm. The altered expression of lncRNAs can reflect the degree of the cancer process, and are regarded as indicators of patient prognosis (Mercer, Dinger & Mattick, 2009). Previous studies have revealed that lncRNAs can be potential biomarkers for prognosis of COAD (Huang et al., 2019). However, most of them explored a limited number of cases without validation groups to test whether the outcome is universally suitable.

Here, from The Cancer Genome Atlas (TCGA), a project containing numerous samples of gene information for 33 cancers, the lncRNA expression profiles of COAD patients were acquired to perform a comprehensively global analysis of prognostic lncRNAs. We detected a 7-lncRNA prognostic signature in COAD and combined it with the traditional clinicopathological risk factors of the disease to form a predictive nomogram. The predictive nomogram is conducive for predicting the prognosis of COAD and guiding clinical treatment.

## Materials & Methods

### Patient cohort and RNA-Seq data

**Figure 1:**
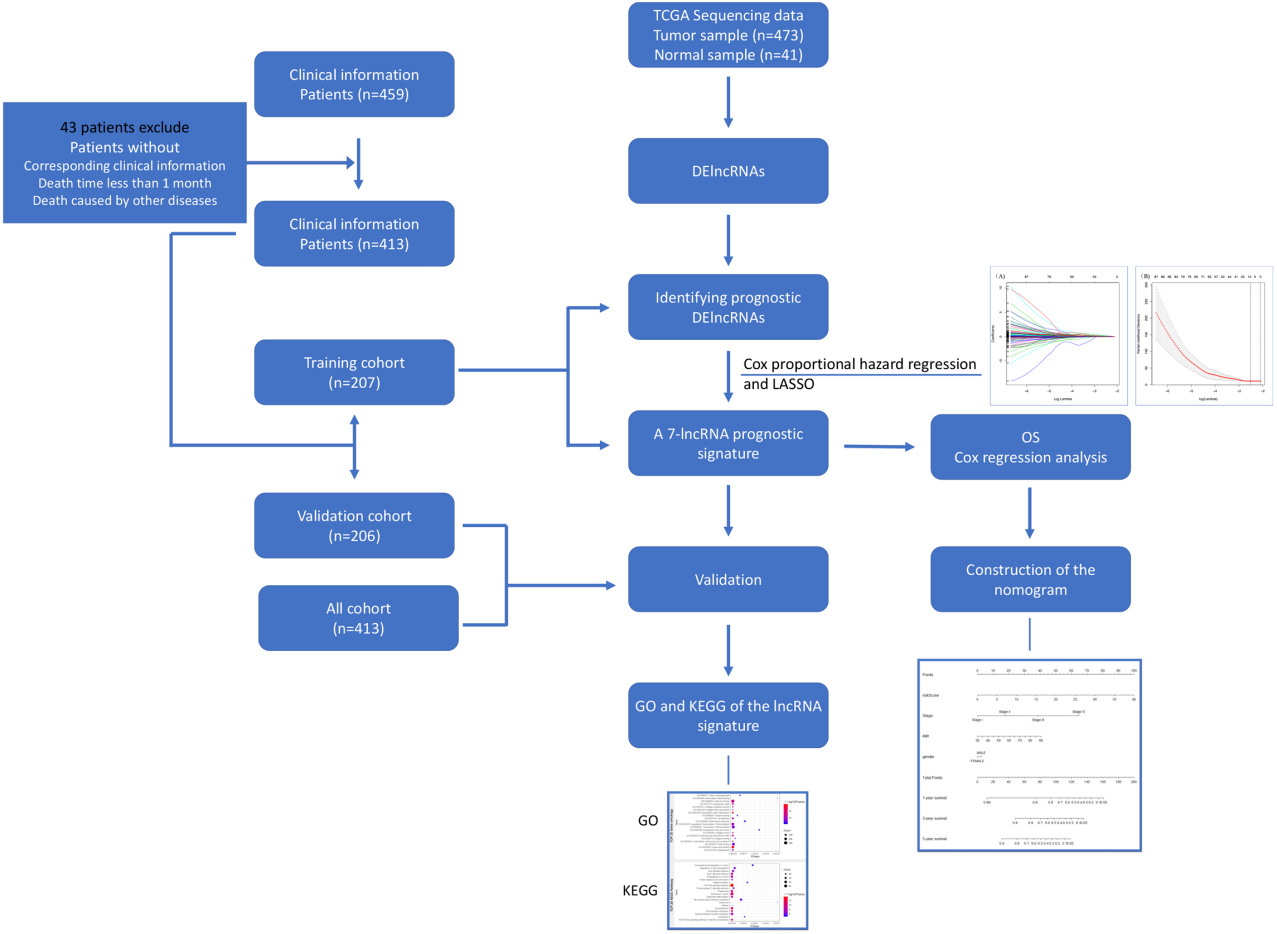
Flowchart for identifying a prognostic lncRNAs of COAD. RNA-Seq data and relevant clinical information for COAD patients were download from the TCGA database. We identified DElncRNAs and further identified and validated a 7-lncRNA prognostic signature. Then a nomogram was built as a prognostic model for COAD. The GO and KEGG were used for the exploration of coexpressed mRNAs’ function. lncRNA, long noncoding RNA; DElncRNAs, differentially expressed lncRNA; OS, overall survival; LASSO, least absolute shrinkage and selection operator; TCGA, The Cancer Genome Atlas; COAD, colon adenocarcinoma.

From the official website of TCGA (https://cancergenome.nih.gov/), we obtained the RNA-Seq (level 3, HTSeq-FPKM) data along with the relevant clinical information of 473 COAD patients. Approval from the ethics committee was not required, since the clinical and RNA-Seq expression data were obtained from TCGA. Furthermore, the quality of the clinical data was assessed, and some patients were not included in our studies for the following reasons: (a) their clinical prognostic information was not available; (b) some patients died in the first month after diagnosis; and (c) some deaths were caused by other diseases and accidents. Patients with complete clinical information were randomly split into training and validation cohorts using the R package tool called “caret”. The *χ*2 test and unpaired two-tailed Student’s *t*-test were performed to detect significant differences in clinical characteristics between training and validation cohorts. A flowchart of this study is shown in Fig. 1.

### Identification of differentially expressed long noncoding RNA between adjacent non-tumor tissue and COAD tissue

The Fragments Per Kilobase of transcript per Million Fragments (FPKM) value of lncRNAs was transformed to transcripts per million (TPM) value. If multiple probes represent the identical lncRNA, the average number of these probes was considered the expression number. The “Limma” package in R and the Wilcoxon signed-rank test were used for identifying the differentially expressed lncRNAs (DElncRNAs). The thresholds for screening DElncRNAs were set at |log2(foldchange)| > 1 and false discovery rate (FDR) adjust *p* < 0.05. Next, the volcano plots of DElncRNAs were constructed.

### Construction of prognostic signature in the training cohort

The univariate Cox model was used for assessing the prognostic implication of DElncRNAs, and DElncRNAs with *p* values less than 0.05 in the training cohort were defined as prognosis-related factors. The lncRNAs from previous screenings were further filtered for crucial lncRNAs using the least absolute shrinkage and selection operator (LASSO) regression method. LASSO is a type of compression estimate. Using LASSO and a penalty proportional to the regression coefficient’s contraction, lncRNAs participating in the prognosis of COAD patients were selected. The R package “glmnet” was used to conduct the LASSO cox analysis to detect crucial lncRNAs. Furthermore, we used multivariate Cox analysis to reduce the dimensionality of these data for further selection. Finally, we constructed a prognostic signature of COAD and calculated the risk score of each patient. According to the risk scores, and with the use of the corresponding median risk score as the cutoff value, COAD patients were split into low-risk and high-risk groups. For the assessment of the survival differences between low-risk and high-risk groups, we used Kaplan–Meier curves and log-rank test.

### Validation of signatures in the validation, as well as all cohorts

Similarly, the model coefficients were used to calculate the risk scores in the validation, as well as all cohorts. Kaplan–Meier and log-rank tests were used to compare high- and low-risk patients. The prediction accuracy of the lncRNA signature was estimated based on the receiver operating characteristic (ROC) curves and c-index using the R package “survivalROC”.

### Generation of the nomogram

A prognostic nomogram was generated according to the prognostic lncRNAs and traditional risk factors related to clinicopathology. It was an applicable model to predict the survival probability of patients in clinics. The “rms” package of R was used to generate the nomogram.

### Functional annotation

To identify the related mRNAs and elucidate the probable biological functions of the seven prognostic lncRNAs, as well as for the assessment of the relevance between the seven prognostic lncRNAs and mRNAs, the calculated Pearson correlation coefficient (PCC) was employed. The PCC value of mRNA was |*r*| > 0.3, *p* ≤ 0.05. The enrichment analyses, including Gene Ontology (GO) and Kyoto Encyclopedia of Genes and Genomes (KEGG) (Ogata et al., 1999) pathway analyses were performed using the R package “clusterprofiler”. Statistical significance was set at *p* <0.05.

## Results

### Patient characteristics

We obtained RNA-Seq data of 41 adjacent normal tissue samples and 473 COAD samples from TCGA. After screening the eligibility of the clinical data, there were 413 COAD patients (all cohort) with relevant prognostic information and RNA-Seq data. All cohorts were randomly assigned to the training (207 patients) or validation cohort (206 patients). The *χ*^2^ test and unpaired two-tailed Student’s *t*-test showed that there was no significant difference between the validation and training cohorts. We have summarized the clinical features of all the cohorts separately in Table 1.

**Table 1:** The main clinic characteristics of the 413 COAD patients.

**Characteristic**		**All cohort number (*n* = 413)**	**Training number (*n* = 207)**	**Validation number (*n* = 206)**	***P* value**
Age	≧60	295	155	140	0.12
	<60	118	52	66	
Gender	Male	192	92	100	0.404
	Female	221	115	106	
Pathologic stage	Stage I/II	230	117	113	0.687
	Stage III/IV	172	84	88	
	Unknow	11	6	5	
T classification	T1 + T2	83	40	43	0.694
	T3 + T4	330	167	163	
N classification	N0	245	124	121	0.809
	N1 + N2	168	83	85	
M classification	M0	307	160	147	0.103
	M1	57	23	34	
	MX	44	23	21	
	Unknown	5	1	4	
Venous invasion	No	270	137	133	0.623
	Yes	88	42	46	
	NA	55	28	27	
Lymphatic invasion	No	229	117	112	0.641
	Yes	144	70	74	
	NA	40	20	20	
New event	No	327	169	158	0.216
	Yes	86	38	48	
Survival status	Alive	328	163	165	0.734
	Dead	85	44	41	
New event time		788.87 ± 699.96	797.91 ± 691.95	779.78 ± 709.50	0.721
Survival time		879.14 ± 737.59	876.01 ± 731.45	882.29 ± 762.82	0.383

**Notes.**

*p* value refers to the *χ*^2^ test except new event time and survival time. *p* value of new event time and survival time refers to the unpaired two-tailed Student’s *t*-test.

Abbreviations COAD colon adenocarcinoma T tumor size N lymph node status M metastasis

### DElncRNAs between adjacent non-tumor tissue and COAD

**Figure 2:**
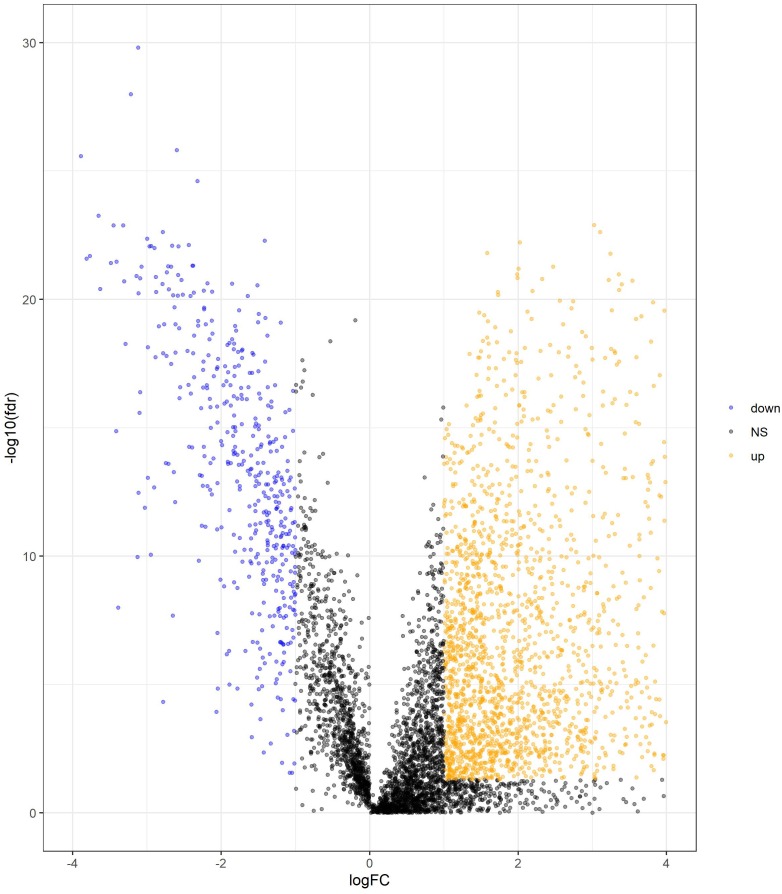
Volcano plot of DElncRNAs between adjacent normal tissue samples and COAD samples. The yellow dot shows the risen expression, and the blue dot the declined expression. The thresholds for screening DElncRNAs were set at |log2(foldchange)| > 1 and false discovery rate (FDR) adjust *p* < 0.05. DElncRNAs, differentially expressed long noncoding RNA; COAD, colon adenocarcinoma.

We determined DElncRNAs based on their log 2(fold change) (log2FC) and *p* values. If —log2FC—>1 and FDR adjust *p* < 0.05, these lncRNAs were differentially expressed in COAD and adjacent non-tumor tissues (If log2FC>1 and FDR adjust *p* < 0.05, these DElncRNAs were over-expressed in COAD. If log2FC<−1 and FDR adjust *p* < 0.05, these DElncRNAs were under-expressed in COAD.). A total of 2417 DElncRNAs (1964 upregulated and 453 downregulated) were identified between normal tissue and COAD (Table S1). The volcano plots of DElncRNAs are shown in Fig. 2.

### Construction of a 7-lncRNA prognostic signature in the training cohort

**Figure 3:**
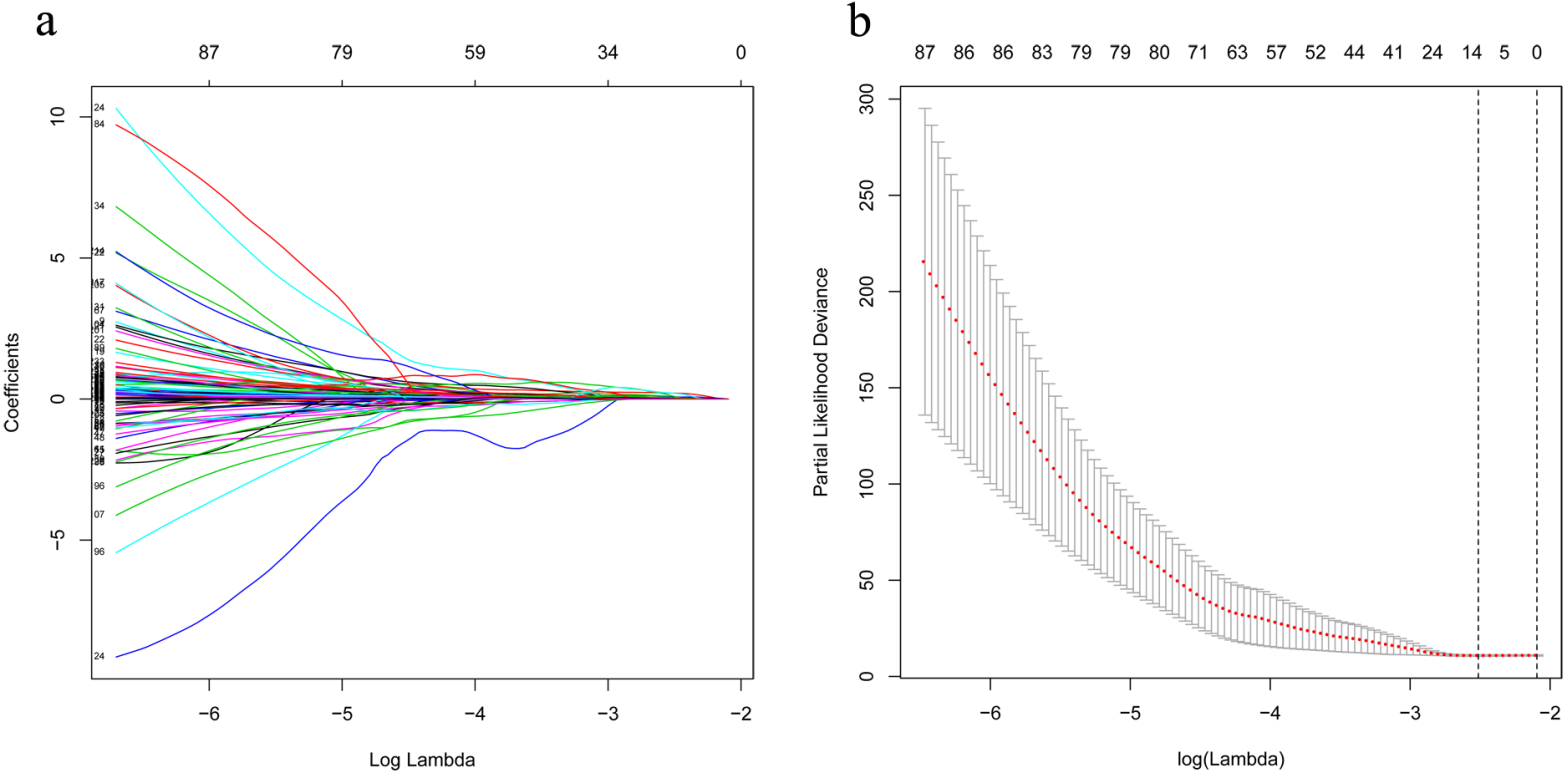
LASSO analysis identified 13 genes which are correlated to overall survival in training set. (A) Ten-time cross-validation for tuning parameter lncRNAs. (B) LASSO coefficient profiles of 233 lncRNAs. LASSO, least absolute shrinkage and selection operator; lncRNAs, long noncoding RNA.

**Figure 4:**
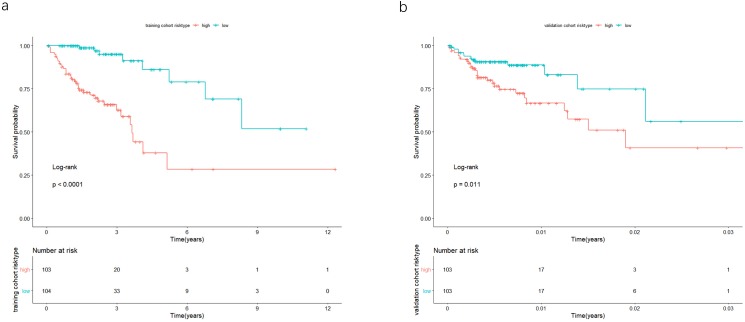
In the training and validation cohort, the patients were split in groups of high and low-risk. Survival analysis was conducted by Kaplan-Meier analysis and log-rank test. Low-risk group exhibited better prognosis than high-expression group.

The univariate Cox analysis in the training cohort identified 233 prognostic lncRNAs (*P* < 0.05) from DElncRNAs (Table S2). Next, LASSO Cox method further identified 13 significant lncRNAs (Fig. 3). We used multivariate Cox analysis to further reduce the dimensionality of the data. Finally, we found that seven lncRNAs (CTC-273B12.10, AC009404.2, AC073283.7, RP11-167H9.4, AC007879.7, RP4-816N1.7, and RP11-400N13.2) were positively correlated with overall survival (OS) and constructed a survival prediction signature. In the multivariate Cox analysis, seven lncRNAs were weighted by regression coefficients to establish the following linear prediction model: $\mathrm{risk~ score}= \left( 0.7577\times \mathrm{expression~ level~ of~ CTC}-\mathrm{273B12.10} \right) + \left( 1.0872\times \mathrm{expression~ level~ of~ AC009404.2} \right) + \left( 0.3082\times \mathrm{expression~ level~ of~ AC073283.7} \right) + \left( 0.4102\times \mathrm{expression~ level~ of~ RP11}-\mathrm{167H9.4} \right) + \left( 1.2925\times \mathrm{expression~ level~ of~ AC007879.7} \right) + \left( 0.6558\times \mathrm{expression~ level~ of~ RP4}-\mathrm{816N1.7} \right) +(0.3350\times \mathrm{expression~ level~ of~ RP11}-\mathrm{400N13.2})$. Patients of the low-risk group had significantly longer median of OS than those in the high-risk group. Log-rank test’s *p* value was less than 0.0001 (Fig. 4A).

### Validation of the 7-lncRNA prognostic signature in the validation cohort

**Figure 5:**
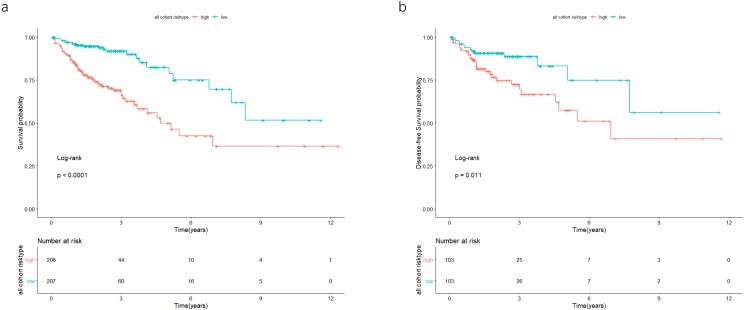
In the all cohort, the patients were split in groups of low and high risk. Using Kaplan-Meier analysis and log-rank test performed survival analysis (A) and disease-free survival analysis (B). Low-risk group exhibited better survival and disease-free survival results.

**Figure 6:**
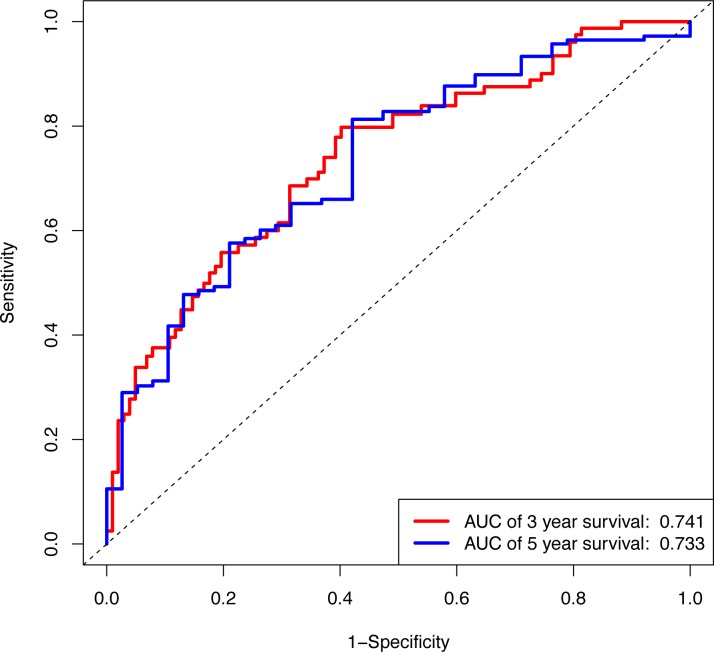
The ROC curve of the prognostic signature for 3-year and 5-year OS probability in all cohort. ROC, receiver operating characteristic; OS, overall survival.

In the validation cohort, there were 206 COAD patients. Using the same signature and cutoff value of the training cohort to calculate risk scores, the validation cohort was also classified into groups of low- and high-risk, and the low-risk groups showed better prognosis than the high-risk groups, as shown in Fig. 4B (*p* = 0.011). Furthermore, we validated our 7-lncRNA prognostic signature in all cohorts (training and validation cohorts) using previous methods and standards. The results showed that the low-risk group exhibited a better prognosis than the high-risk group (*P* < 0.0001), further proving the reliability of the prognostic signature (Fig. 5A). Additionally, the disease-free survival curve analysis was performed in all cohorts, and it suggested that the high-risk group to be inclined to recurrence (*p* = 0.00063) (Fig. 5B). In addition, the area under the ROC curve (AUC) assessed the predictive effect of the signature. The AUC of the prognostic signature was 0.741 for 3-year survival and that for the 5-year survival was 0.733 (Fig. 6). The c-index value was 0.726. Our results indicated the high sensitivity and specificity of the prognostic signature.

### Formation and assessment of the nomogram

**Figure 7:**
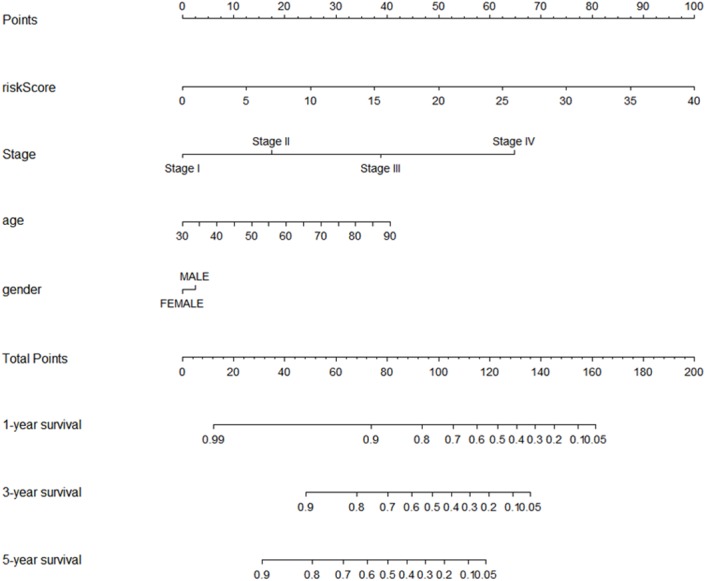
The nomogram for predicting survival probability of COAD patients with 1-, 3- and 5-year OS. The nomogram is applied by adding up points identified for each variable on the points scale. The total points on the bottom scale indicate the probability of 1-,3- and 5-year OS. COAD, colon adenocarcinoma; OS, overall survival.

Using multivariate and univariate Cox analysis, we determined that the 7-lncRNA signature was an individual predictor of COAD patients. In the univariate results, risk score, tumor size, lymph node status, metastasis, stage, venous invasion, and lymphatic invasion were significantly associated with prognosis (Table 2). To further examine whether the 7-lncRNA signature was not associated with previous clinical factors, we performed multivariate Cox regression analysis. The results indicated that risk score and metastasis were the individual prognostic indicators of COAD (Table 2). Furthermore, a prognostic nomogram was constructed based on the previous prognostic factors (Fig. 7).

### Functional annotation of the seven prognostic lncRNAs

**Figure 8:**
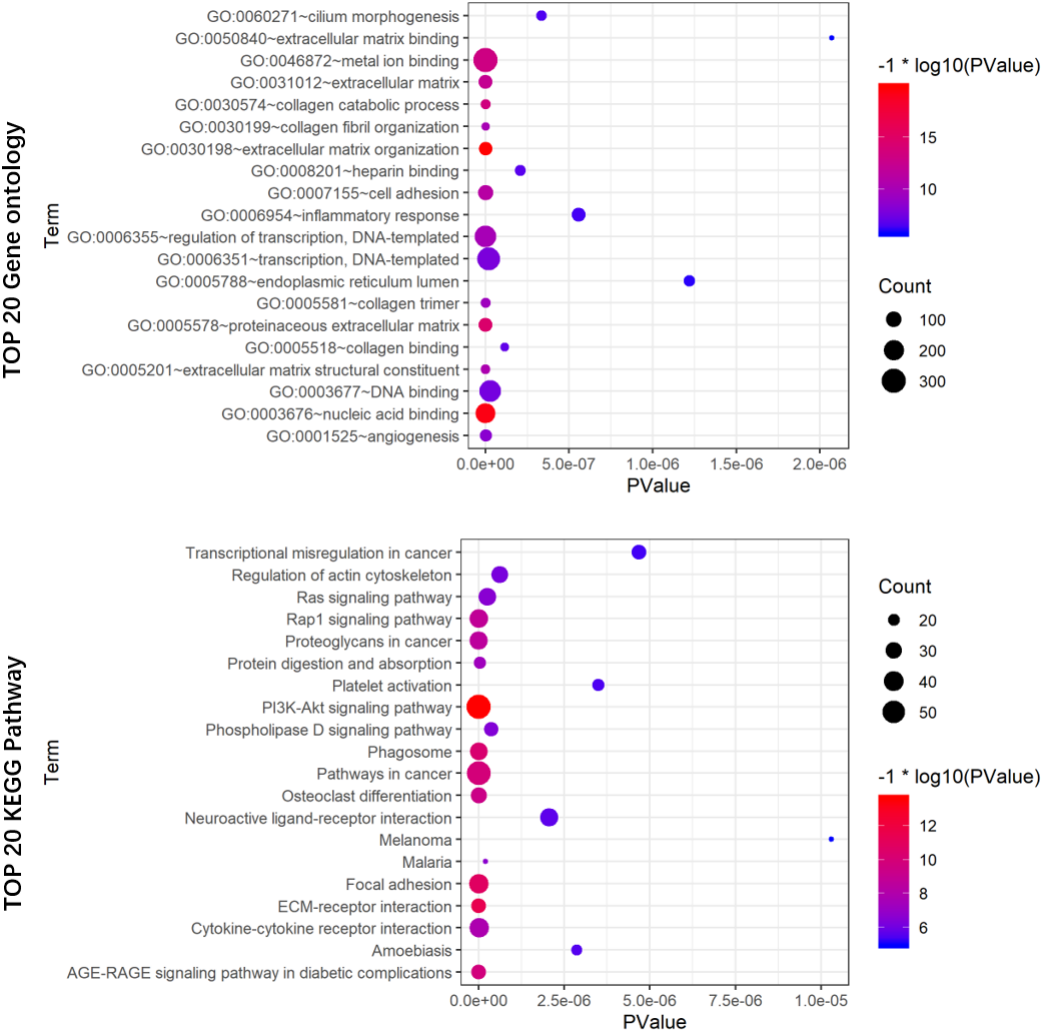
GO (A) and KEGG pathway (B) analysis of the 7-lncRNA related genes. Color represents *P* value, and the size of the balls shows gene number. GO, Gene ontology, KEGG, Kyoto Encyclopedia of Genes and Genomes; lncRNA, long noncoding RNA.

To reveal the potential biological functions of the seven prognostic lncRNAs, the co-expression levels between the seven prognostic lncRNAs and mRNAs were detected using PCC. mRNAs with —PCC— >0.3 and *p* < 0.05 were regarded as mRNAs co-expressed with these lncRNAs. A total of 2012 mRNAs correlated with the seven lncRNAs (Table S3). Extracellular matrix organization, nucleic acid binding, proteinaceous extracellular matrix, collagen catabolic process, and metal ion binding were noticeably upregulated among the GO terms (Fig. 8A). In the KEGG analysis, PI3K/Akt, ECM-receptor interaction, focal adhesion, and AGE-RAGE signaling pathways were significantly upregulated (Fig. 8B).

**Table 2:** A multivariate analysis of survival with clinic factors and the 7-lncRNA signature.

**Variables**	**Univariate analysis**		**Multivariate analysis**	
	**HR (95%CI)**	***P* value**	**HR (95%CI)**	***P* value**
Age (>60 vs ≤60)	1.372 (0.843–2.233)	0.20284069		
Gender (male vs female)	1.198 (0.779–1.843)	0.41154492		
Pathologic stage (stage I/II vs stage III/IV)	3.486 (2.171–5.599)	2.39E−07^*^	3.859 (0.933–15.972)	0.06237977
LncRNA model scores (high score vs low score)	3.494 (2.148–5.683)	4.67E−07^*^	2.363 (1.306–4.275)	0.0044734^*^
T classification (T1/T2 vs T3/T4)	3.458 (1.397–8.559)	0.00730575^*^	2.827 (0.654–12.218)	0.16407723
N classification (N0 vs N1/N2)	3.042 (1.948–4.748)	9.87E−07^*^	0.470 (0.130–1.695)	0.24873161
M classification (M0 vs M1 vs MX)	5.383 (3.328–8.707)	6.82E−12^*^	2.190 (1.147–4.182)	0.01752146^*^
Venous invasion (yes vs no)	2.773 (1.742–4.415)	1.72E−05^*^	1.500 (0.772–2.915)	0.23181158
Lymphatic invasion (yes vs no)	2.414 (1.522–3.830)	0.00018109^*^	1.219 (0.610–2.436)	0.57420717

**Notes.**

Abbreviations CI confidence interval HR hazard ratio lncRNA long noncoding RNA

* *P* value refers to the significant level in the *χ*^2^ test.

## Discussion

COAD is a malignant tumor with the highest lethality, and its etiology involves genetic alteration, environmental exposures, and fat-rich diet consumption (Hu et al., 2011). Due to the molecular heterogeneity of COAD, the traditional factors (e.g., tumor stage, metastasis, number of lymph nodes involved, tumor size, and age) cannot precisely differentiate related cancer risk subgroups, which have distinct clinical outcomes (Burke, 2004). The prognosis and best treatment for this disease can be credibly classified based on cancer-related molecular makers (Levine, 2013). Accordingly, the prognostic potential of molecular markers has been widely studied recently. For instance, Tan & Tan (2011) constructed a well-known gene signature (ColoPrint®) for the prediction of disease relapse in early-stage colorectal cancer.

Initially, lncRNAs were regarded as “junk DNA” in the genome because they do not encode proteins. In recent studies, lncRNAs have been found to function in DNA methylation, histone modification, and regulation of cell proliferation and differentiation (Li et al., 2014). Accumulating evidence has shown that lncRNAs exhibit restrictive cancer-specific and tissue-specific expression patterns; therefore, the emphasis on the identification of molecular biomarkers has been shifted from mRNA and microRNA to lncRNA (Zhang et al., 2016a; Zhang et al., 2016b). Many studies have investigated the lncRNAs involved in the development, diagnosis, and prognosis of colon cancer. For instance, Sun et al. (2016) found a lncRNA (AK098081) that can serve as an independent risk factor for colon cancer by analysis of GEO datasets (150 cases). Moreover, Li et al. (2017) identified eight novel lncRNAs associated with the prognosis of colon cancer. Nevertheless, it is worth noting that our seven lncRNAs only minimally overlap with colon cancer biomarkers identified by previous studies. Previous studies did not contain different cohorts to validate these prognostic lncRNAs and resulted in inconsistent outcomes, potentially due to the different detection methods and the limited sample size.

In our study, we obtained lncRNA expression profiles of COAD from TCGA database with high-throughput analysis of a larger sample size. Using Cox regression and LASSO analysis, we found seven lncRNAs closely associated with the prognosis of COAD patients and form a 7-lncRNA signature, which was estimated by ROC to demonstrate the competitive predictive power of COAD. LASSO can increase prediction accuracy and avoid overfitting risk of predictive models (Wang et al., 2019). Cox regression analysis is the most effective method in the survival time model (Benner et al., 2010). Moreover, validation and all cohorts were used to validate the signature in different groups. Furthermore, the 7-lncRNA signature was combined with some clinicopathological factors to form a predictive prognosis nomogram, which can suitably differentiate patients with high risk of poor prognosis. COAD patients can be managed hierarchically for better treatment. To the best of our knowledge, the seven lncRNAs have not been previously reported, and this is their first report as prognostic makers of COAD.

In our KEGG and GO analyses, we identified genes, which were co-expressed with these seven lncRNAs and pathways related to these genes including PI3K/Akt, ECM-receptor interaction, focal adhesion, and AGE-RAGE signaling pathways. The main function of the PI3K/Akt signaling pathway is signal transduction, and many studies (Blaser, Chadwick & McGinnis, 2010) have suggested that it is involved in cancer progression. Dysregulated gene expression of the PI3K/Akt signaling pathway in colorectal cancer has been reported by a previous study (Slattery et al., 2018). Several studies have reported that ECM-receptor interaction is significantly associated with cancer (e.g., breast cancer (Wang et al., 2018), hepatocellular carcinoma (Zhang et al., 2017), gastric cancer (Zhao et al., 2015), and bladder cancer (He et al., 2019)). Zhai et al. (2017) using the weighted correlation network analysis, found that colon cancer recurrence-associated genes are associated with ECM-receptor interaction. Focal adhesion is believed to regulate the metastasis of numerous cancers (e.g., colon, gastric, and breast cancers (Cance et al., 2000; Lai et al., 2010; Provenzano & Keely, 2009)). Some researchers discovered that focal adhesion molecule modulation can inhibit colorectal cancer cell invasion (Buhrmann et al., 2017). The AGE-RAGE signaling pathway activates downstream signaling, resulting in pathophysiological conditions, such as diabetes and neurological disorders. However, there may also be a relationship between this signaling pathway and cancer (Ahmad et al., 2018). Moreover, the AGE-RAGE signaling pathway has been found to enhance prostate cancer cell proliferation through retinoblastoma phosphorylation (Bao et al., 2015). However, it is not clear whether these seven lncRNAs interact with the PI3K/Akt, ECM-receptor interaction, focal adhesion, and AGE-RAGE signaling pathways in COAD, because we have no experimental information on the mechanisms associated with these lncRNAs. Therefore, further studies on these lncRNAs are needed to better understand the potential mechanisms between the lncRNAs and COAD.

## Conclusions

In conclusion, our study found that seven lncRNAs were significantly associated with prognosis in COAD patients; therefore, the 7-lncRNA signature with some clinicopathological characteristics could be a useful biomarker for the prognosis of COAD.

##  Supplemental Information

10.7717/peerj.8877/supp-1 Table S1 2417 DElncRNAs between normal tissue and COAD 1964 upregulated DElncRNAs and 453 downregulated DElncRNAs.

10.7717/peerj.8877/supp-2 Table S2 233 prognostic lncRNAs from DElncRNAs

10.7717/peerj.8877/supp-3 Table S3 mRNAs correlated with the 7 lncRNAs Abbreviations: lncRNA, long noncoding RNA; mRNA, messenger RNA.

## Additional Information and Declarations

### Data Availability

The following information was supplied regarding data availability: The raw data is available at Dryad: Jiang, QunGuang; Fu, Xiaorui; Duanmu, Jinzhong; Li, Taiyuan (2019), Dr, v4, Dryad, Dataset, https://doi.org/10.5061/dryad.7pvmcvdpc.
