# Supplementary material for: A 7-lncRNA signature associated with the prognosis of colon adenocarcinoma
Source: PeerJ. 2020 Apr 10;8:e8877. doi: 10.7717/peerj.8877 (PMC7153553; doi:10.7717/peerj.8877)
Supplement: Table S1 — 1964 upregulated DElncRNAs and 453 downregulated DElncRNAs. [file peerj-08-8877-s001.docx]

| Gene | ConMean | TreatMean | logFC | pValue | FDR | Type |
| --- | --- | --- | --- | --- | --- | --- |
| LINC00657\|ENSG00000260032\|lincRNA | 127.016309 | 255.353854 | 1.00748408 | 9.78E-14 | 8.47E-13 | up |
| SNHG8\|ENSG00000269893\|lincRNA | 79.2923563 | 161.580201 | 1.02699672 | 1.18E-15 | 1.41E-14 | up |
| GAS5\|ENSG00000234741\|processed_transcript | 64.2496866 | 177.154779 | 1.46324906 | 6.53E-20 | 1.86E-18 | up |
| SNHG6\|ENSG00000245910\|processed_transcript | 62.6178949 | 182.534892 | 1.54352535 | 8.52E-22 | 4.15E-20 | up |
| ZFAS1\|ENSG00000177410\|antisense | 48.7703254 | 194.297993 | 1.9941955 | 1.70E-23 | 1.48E-21 | up |
| NEAT1\|ENSG00000245532\|lincRNA | 29.7687003 | 95.6740299 | 1.68433113 | 0.00049083 | 0.00089968 | up |
| RP11-285F7.2\|ENSG00000242861\|antisense | 25.4466678 | 55.0955572 | 1.11445924 | 1.48E-08 | 5.52E-08 | up |
| MALAT1\|ENSG00000251562\|lincRNA | 22.0293268 | 304.492644 | 3.78891007 | 3.72E-10 | 1.77E-09 | up |
| RP11-304L19.1\|ENSG00000259933\|sense_overlapping | 21.7582928 | 50.3871897 | 1.21149163 | 2.11E-15 | 2.40E-14 | up |
| RP11-304L19.3\|ENSG00000261123\|sense_intronic | 20.0530866 | 47.9143827 | 1.25663446 | 5.14E-16 | 6.50E-15 | up |
| CTD-2377D24.6\|ENSG00000244649\|lincRNA | 18.1005107 | 40.3736602 | 1.15738398 | 1.39E-06 | 3.83E-06 | up |
| CTD-3252C9.4\|ENSG00000267519\|lincRNA | 16.916346 | 45.0846752 | 1.41421915 | 6.71E-13 | 5.12E-12 | up |
| RP11-465B22.8\|ENSG00000272141\|lincRNA | 15.81687 | 42.8670942 | 1.43840649 | 3.20E-11 | 1.79E-10 | up |
| RP11-284F21.10\|ENSG00000272405\|antisense | 12.6097511 | 33.5562188 | 1.41204037 | 1.75E-14 | 1.70E-13 | up |
| ANKRD10-IT1\|ENSG00000229152\|sense_intronic | 11.7547497 | 32.599147 | 1.47159039 | 3.20E-07 | 9.73E-07 | up |
| RP11-284F21.7\|ENSG00000229953\|antisense | 11.3695781 | 22.8669294 | 1.00808393 | 1.09E-08 | 4.14E-08 | up |
| SNHG1\|ENSG00000255717\|processed_transcript | 11.2787626 | 45.3658994 | 2.00799946 | 6.47E-24 | 6.35E-22 | up |
| CTB-193M12.5\|ENSG00000280206\|lincRNA | 10.9212471 | 30.3657273 | 1.47530631 | 6.53E-22 | 3.27E-20 | up |
| SNHG7\|ENSG00000233016\|antisense | 10.8597087 | 30.3587013 | 1.48312467 | 1.82E-18 | 3.69E-17 | up |
| SNHG3\|ENSG00000242125\|sense_intronic | 9.81936012 | 31.7318366 | 1.6922301 | 3.03E-21 | 1.23E-19 | up |
| RP11-150O12.3\|ENSG00000254290\|lincRNA | 9.18419912 | 29.0683744 | 1.66222457 | 4.25E-12 | 2.82E-11 | up |
| CTD-2510F5.4\|ENSG00000265415\|antisense | 9.02730615 | 27.3020116 | 1.59663981 | 5.55E-19 | 1.26E-17 | up |
| CTC-444N24.11\|ENSG00000268205\|lincRNA | 8.30987998 | 18.4561092 | 1.1511989 | 3.75E-16 | 4.92E-15 | up |
| RP11-660L16.2\|ENSG00000254682\|antisense | 8.18478788 | 18.9923569 | 1.21440202 | 4.11E-09 | 1.68E-08 | up |
| RNASEH1-AS1\|ENSG00000234171\|antisense | 7.85779961 | 16.652255 | 1.08352027 | 1.43E-15 | 1.69E-14 | up |
| RP11-303E16.2\|ENSG00000261061\|sense_intronic | 7.83854227 | 23.5273172 | 1.58567953 | 8.06E-21 | 3.08E-19 | up |
| SNHG17\|ENSG00000196756\|processed_transcript | 7.61497812 | 38.0911748 | 2.32254499 | 1.99E-23 | 1.64E-21 | up |
| RP5-881L22.5\|ENSG00000226812\|antisense | 7.32871827 | 32.0177655 | 2.12723982 | 1.38E-09 | 6.04E-09 | up |
| MCF2L-AS1\|ENSG00000235280\|antisense | 6.88803615 | 21.5164847 | 1.64327777 | 4.50E-14 | 4.09E-13 | up |
| RP11-462G2.1\|ENSG00000237643\|lincRNA | 6.88749308 | 103.21462 | 3.90552457 | 1.67E-05 | 3.87E-05 | up |
| RP11-156E6.1\|ENSG00000259623\|sense_overlapping | 6.68428759 | 13.4928222 | 1.01334643 | 1.86E-16 | 2.56E-15 | up |
| AGAP2-AS1\|ENSG00000255737\|antisense | 6.42446063 | 16.1830857 | 1.33283947 | 7.87E-12 | 4.96E-11 | up |
| CTA-29F11.1\|ENSG00000260708\|antisense | 6.28609829 | 12.9998307 | 1.0482561 | 7.63E-12 | 4.83E-11 | up |
| CTD-3184A7.4\|ENSG00000232442\|antisense | 6.25603161 | 24.6544066 | 1.97852582 | 4.18E-20 | 1.27E-18 | up |
| CTD-2228K2.7\|ENSG00000225138\|processed_transcript | 6.16676862 | 22.4405177 | 1.86351934 | 1.82E-09 | 7.82E-09 | up |
| SNHG15\|ENSG00000232956\|lincRNA | 6.13535688 | 24.9237721 | 2.02230326 | 3.01E-25 | 6.14E-23 | up |
| SNHG16\|ENSG00000163597\|processed_transcript | 5.91047149 | 17.7077356 | 1.58303461 | 1.09E-24 | 1.57E-22 | up |
| AC016735.1\|ENSG00000224739\|lincRNA | 5.58946795 | 20.0334065 | 1.84162489 | 5.48E-12 | 3.58E-11 | up |
| SNHG11\|ENSG00000174365\|processed_transcript | 5.58257666 | 15.1586532 | 1.44113851 | 3.74E-19 | 8.84E-18 | up |
| RP5-1085F17.3\|ENSG00000260257\|lincRNA | 5.16589561 | 11.127185 | 1.10699827 | 7.41E-11 | 3.94E-10 | up |
| CASC9\|ENSG00000249395\|lincRNA | 5.1259751 | 14.2587561 | 1.47594976 | 1.71E-06 | 4.65E-06 | up |
| SNHG12\|ENSG00000197989\|antisense | 5.07252746 | 14.6301382 | 1.52816672 | 1.11E-20 | 4.15E-19 | up |
| RP1-239B22.5\|ENSG00000260196\|antisense | 4.99440227 | 14.1850407 | 1.50598636 | 1.34E-16 | 1.90E-15 | up |
| RP11-97C16.1\|ENSG00000271870\|antisense | 4.88092493 | 10.5431565 | 1.1110804 | 1.00E-11 | 6.23E-11 | up |
| LINC00963\|ENSG00000204054\|processed_transcript | 4.77430949 | 9.79996598 | 1.03748465 | 7.61E-17 | 1.14E-15 | up |
| CTD-2015H6.3\|ENSG00000249042\|antisense | 4.53535963 | 9.07143034 | 1.00011309 | 1.40E-13 | 1.18E-12 | up |
| AC006042.6\|ENSG00000227719\|antisense | 4.51344052 | 10.8626957 | 1.26708267 | 1.87E-08 | 6.90E-08 | up |
| RP11-510N19.5\|ENSG00000249007\|sense_intronic | 4.50027256 | 14.7378381 | 1.71144062 | 4.83E-13 | 3.76E-12 | up |
| RP3-508I15.21\|ENSG00000272669\|antisense | 4.42091466 | 9.37999863 | 1.08524283 | 1.70E-08 | 6.31E-08 | up |
| FAM83H-AS1\|ENSG00000203499\|lincRNA | 4.33909817 | 37.3705229 | 3.10643362 | 8.16E-26 | 2.40E-23 | up |
| PRRT3-AS1\|ENSG00000230082\|antisense | 4.24169597 | 11.928718 | 1.49172588 | 1.50E-13 | 1.26E-12 | up |
| MINCR\|ENSG00000253716\|antisense | 4.14953073 | 11.7218636 | 1.49818186 | 1.98E-17 | 3.25E-16 | up |
| RP11-258C19.7\|ENSG00000270189\|lincRNA | 4.14321471 | 9.4450056 | 1.18880107 | 4.85E-08 | 1.69E-07 | up |
| AC021218.2\|ENSG00000204876\|lincRNA | 4.05847127 | 26.1123669 | 2.68572493 | 2.22E-20 | 7.53E-19 | up |
| DIO3OS\|ENSG00000258498\|lincRNA | 3.93158577 | 8.43748457 | 1.10170163 | 0.0146873 | 0.02066278 | up |
| RP11-670E13.6\|ENSG00000274213\|lincRNA | 3.91183418 | 9.08954758 | 1.21636327 | 4.55E-10 | 2.13E-09 | up |
| RP11-3P17.5\|ENSG00000269888\|lincRNA | 3.81378695 | 8.65890083 | 1.18295965 | 2.86E-06 | 7.49E-06 | up |
| CTD-2537I9.12\|ENSG00000267523\|antisense | 3.7887412 | 12.3305189 | 1.70244301 | 4.00E-11 | 2.20E-10 | up |
| RP5-1056H1.2\|ENSG00000278192\|lincRNA | 3.7599273 | 8.13048064 | 1.11263588 | 3.89E-06 | 9.98E-06 | up |
| TRIM31-AS1\|ENSG00000231226\|antisense | 3.75709865 | 8.60344558 | 1.19529556 | 5.09E-10 | 2.37E-09 | up |
| RP3-325F22.5\|ENSG00000272189\|antisense | 3.72173259 | 7.61476451 | 1.03282502 | 2.85E-13 | 2.29E-12 | up |
| H19\|ENSG00000130600\|processed_transcript | 3.59830329 | 58.0033268 | 4.01074695 | 1.40E-11 | 8.41E-11 | up |
| VPS9D1-AS1\|ENSG00000261373\|antisense | 3.56524074 | 23.4598874 | 2.71812469 | 4.17E-22 | 2.23E-20 | up |
| PTOV1-AS2\|ENSG00000269352\|antisense | 3.55449638 | 8.06877884 | 1.18270518 | 6.90E-12 | 4.40E-11 | up |
| RP11-589N15.2\|ENSG00000269899\|sense_intronic | 3.52425559 | 13.809612 | 1.97028233 | 8.49E-20 | 2.32E-18 | up |
| THUMPD3-AS1\|ENSG00000206573\|antisense | 3.36599183 | 7.501007 | 1.15605261 | 9.85E-13 | 7.27E-12 | up |
| SNHG10\|ENSG00000247092\|antisense | 3.36207507 | 7.63319547 | 1.1829352 | 3.85E-16 | 5.04E-15 | up |
| LINC00239\|ENSG00000258512\|lincRNA | 3.35552679 | 9.09033148 | 1.43779363 | 6.91E-09 | 2.71E-08 | up |
| RP11-401P9.4\|ENSG00000261685\|lincRNA | 3.29711104 | 13.0441579 | 1.98412943 | 1.60E-06 | 4.38E-06 | up |
| RP3-368A4.5\|ENSG00000271430\|sense_intronic | 3.23210839 | 9.05803349 | 1.4867223 | 7.98E-06 | 1.94E-05 | up |
| RP11-417L19.6\|ENSG00000273568\|sense_intronic | 3.1947823 | 10.4802918 | 1.71388936 | 1.30E-20 | 4.71E-19 | up |
| RP11-521B24.5\|ENSG00000257270\|antisense | 3.1776612 | 7.44933759 | 1.22914683 | 1.41E-08 | 5.28E-08 | up |
| RP5-908M14.9\|ENSG00000273619\|antisense | 3.14944534 | 9.62211746 | 1.61125664 | 7.75E-17 | 1.15E-15 | up |
| CTD-2006H14.2\|ENSG00000271971\|antisense | 3.149028 | 6.45619058 | 1.03577658 | 2.67E-08 | 9.60E-08 | up |
| RP11-242D8.1\|ENSG00000267002\|lincRNA | 3.12555568 | 6.31595878 | 1.01488905 | 2.15E-10 | 1.06E-09 | up |
| PCAT6\|ENSG00000228288\|antisense | 3.00534338 | 7.18501259 | 1.25746085 | 1.36E-13 | 1.16E-12 | up |
| MNX1-AS1\|ENSG00000243479\|lincRNA | 2.97858373 | 12.7026934 | 2.09243601 | 2.33E-20 | 7.83E-19 | up |
| RP6-65G23.3\|ENSG00000259153\|lincRNA | 2.96813962 | 6.43338712 | 1.11601955 | 3.04E-11 | 1.71E-10 | up |
| LINC01315\|ENSG00000229891\|lincRNA | 2.9391371 | 12.148153 | 2.04727242 | 3.61E-17 | 5.70E-16 | up |
| RP11-403P17.6\|ENSG00000277978\|antisense | 2.90807651 | 7.58988879 | 1.38401352 | 1.18E-14 | 1.19E-13 | up |
| RP11-132A1.4\|ENSG00000232445\|antisense | 2.8683502 | 25.4222482 | 3.14779854 | 1.95E-18 | 3.92E-17 | up |
| LINC00265\|ENSG00000188185\|lincRNA | 2.82544067 | 6.53550854 | 1.2098236 | 1.16E-11 | 7.13E-11 | up |
| RP11-24F11.2\|ENSG00000223552\|antisense | 2.80582832 | 6.53849184 | 1.22053116 | 1.43E-08 | 5.36E-08 | up |
| LENG8-AS1\|ENSG00000226696\|antisense | 2.78624215 | 6.92687947 | 1.31388493 | 7.64E-16 | 9.39E-15 | up |
| AC091729.9\|ENSG00000229043\|antisense | 2.76857229 | 6.02810198 | 1.12256163 | 1.02E-11 | 6.31E-11 | up |
| RP11-11N5.1\|ENSG00000250829\|lincRNA | 2.71711319 | 15.5226815 | 2.51423123 | 0.00327419 | 0.00517487 | up |
| RP11-1149O23.3\|ENSG00000246582\|processed_transcript | 2.69484333 | 5.7700458 | 1.09838137 | 8.42E-14 | 7.39E-13 | up |
| RP11-706O15.1\|ENSG00000205664\|lincRNA | 2.6879282 | 8.8934468 | 1.72624807 | 7.91E-23 | 5.17E-21 | up |
| SNHG20\|ENSG00000234912\|processed_transcript | 2.68508209 | 6.83641027 | 1.34827279 | 4.44E-20 | 1.33E-18 | up |
| AC015849.19\|ENSG00000270871\|antisense | 2.6842442 | 6.23663649 | 1.21625225 | 6.02E-07 | 1.76E-06 | up |
| AC074117.10\|ENSG00000234072\|antisense | 2.67871498 | 5.95690082 | 1.15302086 | 5.29E-15 | 5.63E-14 | up |
| SNHG25\|ENSG00000266402\|lincRNA | 2.65804292 | 79.9707156 | 4.91103549 | 2.25E-21 | 9.56E-20 | up |
| LINC00152\|ENSG00000222041\|lincRNA | 2.64954115 | 8.05740735 | 1.60457316 | 1.92E-19 | 4.79E-18 | up |
| RP11-159D12.2\|ENSG00000264112\|lincRNA | 2.62678357 | 8.07888943 | 1.62085965 | 9.16E-12 | 5.71E-11 | up |
| RP13-401N8.1\|ENSG00000226465\|antisense | 2.619122 | 7.55930899 | 1.5291711 | 0.00071632 | 0.00127166 | up |
| SYNPR-AS1\|ENSG00000241359\|antisense | 2.60150773 | 5.25095848 | 1.01323279 | 5.98E-07 | 1.75E-06 | up |
| RP11-196G18.22\|ENSG00000261716\|sense_overlapping | 2.58997593 | 5.37918969 | 1.05445018 | 1.54E-09 | 6.72E-09 | up |
| RP11-157P1.4\|ENSG00000226332\|antisense | 2.57546579 | 8.29885048 | 1.68807814 | 4.00E-17 | 6.25E-16 | up |
| AC000123.4\|ENSG00000224138\|antisense | 2.56062046 | 5.56433479 | 1.1197158 | 7.64E-08 | 2.60E-07 | up |
| BX470102.3\|ENSG00000238279\|antisense | 2.54985879 | 15.5387861 | 2.60738455 | 2.14E-21 | 9.28E-20 | up |
| AC010761.8\|ENSG00000264577\|antisense | 2.54681869 | 7.07064565 | 1.47314571 | 3.04E-18 | 5.82E-17 | up |
| CTB-31O20.2\|ENSG00000261526\|lincRNA | 2.539026 | 5.51924322 | 1.1201953 | 1.61E-14 | 1.57E-13 | up |
| RP5-967N21.11\|ENSG00000275632\|lincRNA | 2.53093357 | 6.70195359 | 1.40491205 | 1.49E-12 | 1.07E-11 | up |
| SNHG4\|ENSG00000281398\|processed_transcript | 2.52861793 | 10.3980232 | 2.0398883 | 1.03E-19 | 2.73E-18 | up |
| LINC00106\|ENSG00000236871\|lincRNA | 2.46718356 | 6.70910662 | 1.44325562 | 6.18E-09 | 2.45E-08 | up |
| RP13-516M14.10\|ENSG00000275888\|antisense | 2.43969343 | 5.2374557 | 1.10216627 | 1.43E-08 | 5.34E-08 | up |
| ASMTL-AS1\|ENSG00000236017\|antisense | 2.41624662 | 7.13309834 | 1.56176115 | 1.20E-10 | 6.16E-10 | up |
| GS1-124K5.4\|ENSG00000237310\|lincRNA | 2.39510708 | 6.7303214 | 1.49058524 | 3.15E-16 | 4.20E-15 | up |
| TUSC8\|ENSG00000237361\|lincRNA | 2.39160948 | 9.2985118 | 1.959018 | 1.15E-06 | 3.19E-06 | up |
| WFDC21P\|ENSG00000261040\|processed_transcript | 2.38418902 | 6.7549787 | 1.50245261 | 0.0137773 | 0.01951201 | up |
| INE1\|ENSG00000224975\|sense_intronic | 2.38356531 | 4.80425634 | 1.01119198 | 3.49E-07 | 1.05E-06 | up |
| KB-1208A12.3\|ENSG00000245970\|antisense | 2.29994402 | 6.97323638 | 1.60022964 | 1.48E-20 | 5.28E-19 | up |
| AC018766.4\|ENSG00000267896\|antisense | 2.29896044 | 4.72271146 | 1.03863376 | 1.89E-07 | 5.99E-07 | up |
| RUSC1-AS1\|ENSG00000225855\|antisense | 2.27072842 | 6.57181941 | 1.53313767 | 3.73E-15 | 4.08E-14 | up |
| RP11-458F8.4\|ENSG00000273142\|lincRNA | 2.27057902 | 6.79299384 | 1.5809873 | 4.03E-14 | 3.70E-13 | up |
| CTD-2574D22.4\|ENSG00000260114\|sense_intronic | 2.24897468 | 4.8817898 | 1.11814276 | 1.58E-13 | 1.32E-12 | up |
| RP11-51J9.5\|ENSG00000271869\|lincRNA | 2.24303762 | 4.56911591 | 1.02646123 | 6.21E-08 | 2.15E-07 | up |
| CTB-129O4.1\|ENSG00000248367\|antisense | 2.21574525 | 5.14526779 | 1.21545414 | 5.06E-10 | 2.35E-09 | up |
| RP11-390F4.3\|ENSG00000225489\|lincRNA | 2.18848119 | 5.96711859 | 1.44710446 | 1.45E-09 | 6.35E-09 | up |
| RP1-101A2.1\|ENSG00000273893\|sense_intronic | 2.17125343 | 4.59932915 | 1.08289532 | 2.07E-08 | 7.59E-08 | up |
| RP11-876N24.5\|ENSG00000263013\|sense_intronic | 2.16934938 | 4.51000853 | 1.05586774 | 1.86E-08 | 6.86E-08 | up |
| RP11-20I20.4\|ENSG00000273179\|antisense | 2.13941579 | 6.64770455 | 1.63563937 | 1.08E-12 | 7.86E-12 | up |
| CTD-3025N20.3\|ENSG00000272010\|lincRNA | 2.11667608 | 4.53554499 | 1.09947541 | 3.41E-07 | 1.03E-06 | up |
| RP11-783K16.5\|ENSG00000256940\|antisense | 2.10367136 | 9.54931158 | 2.18248739 | 1.26E-21 | 5.87E-20 | up |
| RP11-513M16.7\|ENSG00000272842\|antisense | 2.08459834 | 4.23638022 | 1.02306265 | 4.49E-06 | 1.14E-05 | up |
| UCA1\|ENSG00000214049\|processed_transcript | 2.07472917 | 38.5565067 | 4.21597942 | 2.43E-15 | 2.72E-14 | up |
| RP11-783K16.13\|ENSG00000257086\|lincRNA | 2.05076707 | 4.84928 | 1.24160692 | 4.49E-16 | 5.72E-15 | up |
| RP5-1074L1.4\|ENSG00000273373\|antisense | 2.04938764 | 4.6551284 | 1.18362807 | 1.95E-07 | 6.16E-07 | up |
| RP11-180M15.7\|ENSG00000275560\|sense_intronic | 2.0279074 | 5.51546647 | 1.44349113 | 1.31E-11 | 7.93E-11 | up |
| CTA-228A9.3\|ENSG00000272720\|lincRNA | 1.9912319 | 4.73728281 | 1.25039855 | 2.95E-09 | 1.23E-08 | up |
| RP11-449P15.2\|ENSG00000273151\|antisense | 1.98270545 | 4.33300164 | 1.12789642 | 1.19E-06 | 3.31E-06 | up |
| RP13-104F24.2\|ENSG00000215769\|processed_transcript | 1.95572601 | 4.37919064 | 1.16295999 | 8.03E-09 | 3.11E-08 | up |
| RP4-758J18.13\|ENSG00000272455\|lincRNA | 1.91886439 | 5.36423303 | 1.48311916 | 6.46E-20 | 1.85E-18 | up |
| DLGAP1-AS2\|ENSG00000262001\|antisense | 1.91282387 | 10.6033703 | 2.47074696 | 5.41E-24 | 5.41E-22 | up |
| CTA-445C9.14\|ENSG00000261188\|antisense | 1.90695891 | 3.90901732 | 1.03553222 | 1.77E-12 | 1.25E-11 | up |
| RP11-253E3.3\|ENSG00000250899\|lincRNA | 1.88486663 | 4.98661126 | 1.40359729 | 4.14E-16 | 5.38E-15 | up |
| LINC01272\|ENSG00000224397\|lincRNA | 1.88292306 | 5.38913171 | 1.51707879 | 6.93E-13 | 5.26E-12 | up |
| CTD-2227E11.1\|ENSG00000270933\|lincRNA | 1.87773878 | 9.92143621 | 2.4015526 | 1.28E-17 | 2.16E-16 | up |
| CTD-2349P21.9\|ENSG00000266490\|lincRNA | 1.87546066 | 6.24761836 | 1.73606133 | 6.95E-14 | 6.16E-13 | up |
| AC016831.7\|ENSG00000233559\|lincRNA | 1.86421529 | 6.16968945 | 1.7266294 | 3.17E-09 | 1.32E-08 | up |
| AP006621.6\|ENSG00000255142\|lincRNA | 1.83088927 | 4.37618805 | 1.25713019 | 1.04E-05 | 2.48E-05 | up |
| RP11-379B8.1\|ENSG00000226453\|lincRNA | 1.79968869 | 4.98747504 | 1.47056225 | 0.00019052 | 0.00037589 | up |
| RP11-126L15.4\|ENSG00000236305\|antisense | 1.79615846 | 8.85943165 | 2.30229952 | 3.14E-20 | 1.01E-18 | up |
| TMEM191A\|ENSG00000226287\|processed_transcript | 1.79483202 | 5.70737792 | 1.66897927 | 1.00E-16 | 1.46E-15 | up |
| RP11-268J15.5\|ENSG00000116883\|antisense | 1.78680652 | 6.05052386 | 1.75967663 | 1.61E-11 | 9.46E-11 | up |
| AC026471.6\|ENSG00000260740\|antisense | 1.7770948 | 3.71426878 | 1.06355757 | 0.00020959 | 0.00041134 | up |
| CTC-471J1.2\|ENSG00000260160\|sense_overlapping | 1.77546778 | 3.68733188 | 1.05437809 | 1.60E-07 | 5.15E-07 | up |
| RP11-93K22.13\|ENSG00000248243\|lincRNA | 1.77121221 | 4.13124171 | 1.2218384 | 1.60E-11 | 9.44E-11 | up |
| CTD-3128G10.6\|ENSG00000269680\|antisense | 1.76946659 | 3.75994848 | 1.08739837 | 1.57E-10 | 7.92E-10 | up |
| MIR222HG\|ENSG00000270069\|lincRNA | 1.75623467 | 4.46013778 | 1.34460265 | 3.26E-09 | 1.35E-08 | up |
| MIR4435-2HG\|ENSG00000172965\|lincRNA | 1.75249398 | 5.8278957 | 1.73356557 | 1.10E-22 | 6.75E-21 | up |
| TMEM147-AS1\|ENSG00000236144\|antisense | 1.75153228 | 6.54144549 | 1.9009919 | 4.36E-21 | 1.74E-19 | up |
| RP5-1159O4.1\|ENSG00000272894\|lincRNA | 1.74836926 | 4.0394779 | 1.20815892 | 9.62E-14 | 8.36E-13 | up |
| CTD-3065J16.9\|ENSG00000255224\|antisense | 1.74087058 | 3.80081954 | 1.12650158 | 1.94E-14 | 1.86E-13 | up |
| RP3-475N16.1\|ENSG00000231113\|antisense | 1.73702825 | 3.62087999 | 1.05971914 | 1.34E-16 | 1.90E-15 | up |
| RP11-443B20.1\|ENSG00000271936\|antisense | 1.73042132 | 3.57737301 | 1.04777721 | 2.05E-15 | 2.35E-14 | up |
| RP3-368A4.6\|ENSG00000271533\|sense_intronic | 1.71988288 | 8.75656362 | 2.3480545 | 4.73E-06 | 1.20E-05 | up |
| RP1-151F17.1\|ENSG00000229931\|antisense | 1.70800474 | 3.47073726 | 1.02293018 | 2.34E-08 | 8.47E-08 | up |
| CTD-2523D13.2\|ENSG00000254854\|antisense | 1.68365556 | 4.18630936 | 1.31408191 | 4.34E-07 | 1.30E-06 | up |
| LINC00920\|ENSG00000246898\|lincRNA | 1.68278564 | 7.27334247 | 2.1117671 | 2.82E-11 | 1.59E-10 | up |
| RP1-151F17.2\|ENSG00000272341\|lincRNA | 1.6710079 | 3.36715582 | 1.01081194 | 1.09E-07 | 3.61E-07 | up |
| RP11-713M15.2\|ENSG00000272502\|antisense | 1.65388149 | 13.1513778 | 2.99128618 | 2.31E-20 | 7.80E-19 | up |
| RP11-44F14.2\|ENSG00000261804\|lincRNA | 1.64181581 | 4.53594745 | 1.46611164 | 5.40E-11 | 2.92E-10 | up |
| RP11-326C3.2\|ENSG00000255026\|antisense | 1.62016688 | 22.870645 | 3.81928273 | 1.72E-15 | 2.00E-14 | up |
| LINC01558\|ENSG00000146521\|lincRNA | 1.6137443 | 4.55877838 | 1.49823528 | 3.20E-06 | 8.32E-06 | up |
| RP11-7K24.3\|ENSG00000261068\|lincRNA | 1.6078426 | 3.8559284 | 1.26195208 | 6.36E-08 | 2.19E-07 | up |
| RP11-390P2.4\|ENSG00000225177\|antisense | 1.59000999 | 3.75785606 | 1.24087398 | 1.78E-15 | 2.06E-14 | up |
| LINC01569\|ENSG00000262468\|lincRNA | 1.58499894 | 4.56758466 | 1.52694959 | 1.72E-17 | 2.84E-16 | up |
| RP11-74J13.9\|ENSG00000277662\|sense_intronic | 1.57457182 | 3.82789473 | 1.2815916 | 3.26E-12 | 2.20E-11 | up |
| CTD-2589H19.6\|ENSG00000271781\|antisense | 1.5686279 | 3.68627028 | 1.23265869 | 2.80E-05 | 6.24E-05 | up |
| RP11-631N16.4\|ENSG00000275180\|lincRNA | 1.56615746 | 4.10802397 | 1.39121533 | 3.58E-11 | 1.98E-10 | up |
| RP11-192H23.8\|ENSG00000264608\|sense_intronic | 1.56607414 | 3.13632601 | 1.00192302 | 1.64E-07 | 5.26E-07 | up |
| MFI2-AS1\|ENSG00000228109\|antisense | 1.56068315 | 6.81586866 | 2.12671986 | 6.00E-22 | 3.03E-20 | up |
| SLCO4A1-AS1\|ENSG00000232803\|antisense | 1.56014003 | 22.1487884 | 3.82748035 | 1.08E-18 | 2.33E-17 | up |
| RP11-435O5.2\|ENSG00000237857\|lincRNA | 1.53966265 | 6.0994128 | 1.98605608 | 1.14E-23 | 1.07E-21 | up |
| RP11-669E14.6\|ENSG00000262372\|antisense | 1.52418273 | 3.15119191 | 1.04786175 | 0.03800617 | 0.04980734 | up |
| RP11-1100L3.8\|ENSG00000259884\|lincRNA | 1.5175419 | 3.1862262 | 1.07011235 | 0.01109204 | 0.01597098 | up |
| RP11-108L7.15\|ENSG00000273162\|lincRNA | 1.51370561 | 3.66124988 | 1.27425159 | 1.14E-13 | 9.83E-13 | up |
| AC007405.6\|ENSG00000239467\|lincRNA | 1.50664432 | 6.66335806 | 2.14491055 | 1.69E-15 | 1.98E-14 | up |
| RP11-150O12.6\|ENSG00000253414\|sense_intronic | 1.49839232 | 18.9741237 | 3.66254595 | 9.58E-22 | 4.58E-20 | up |
| RP11-49I11.4\|ENSG00000274849\|sense_intronic | 1.49545382 | 3.0876287 | 1.04591591 | 6.10E-09 | 2.42E-08 | up |
| HM13-IT1\|ENSG00000235313\|sense_intronic | 1.49019111 | 4.50509591 | 1.59606046 | 2.70E-12 | 1.85E-11 | up |
| LINC00174\|ENSG00000179406\|lincRNA | 1.48657524 | 3.47489823 | 1.22497824 | 2.79E-12 | 1.90E-11 | up |
| RP11-44F14.8\|ENSG00000262714\|lincRNA | 1.4837843 | 3.88300382 | 1.38789174 | 2.95E-09 | 1.23E-08 | up |
| AC156455.1\|ENSG00000256546\|processed_transcript | 1.48358918 | 4.24142706 | 1.5154581 | 2.22E-06 | 5.91E-06 | up |
| CTD-2017F17.2\|ENSG00000274383\|antisense | 1.46922127 | 3.98477267 | 1.43944574 | 1.42E-07 | 4.62E-07 | up |
| GABPB1-AS1\|ENSG00000244879\|antisense | 1.46205207 | 3.52338495 | 1.26896741 | 2.09E-08 | 7.63E-08 | up |
| RP11-524D16__A.3\|ENSG00000261295\|antisense | 1.42568163 | 9.00094496 | 2.65842462 | 3.42E-12 | 2.30E-11 | up |
| RP11-293P20.2\|ENSG00000225315\|antisense | 1.41807362 | 3.35142841 | 1.24084368 | 0.0001437 | 0.00028866 | up |
| MGC32805\|ENSG00000250328\|antisense | 1.40991612 | 3.25591729 | 1.20745472 | 0.00011048 | 0.00022576 | up |
| XX-FW83563B9.5\|ENSG00000280195\|antisense | 1.40915444 | 2.85544108 | 1.01888388 | 3.43E-13 | 2.73E-12 | up |
| LA16c-358B7.3\|ENSG00000261505\|antisense | 1.40087168 | 2.86628818 | 1.03285885 | 1.23E-09 | 5.44E-09 | up |
| FOXP4-AS1\|ENSG00000234753\|antisense | 1.4006687 | 8.26261581 | 2.56048284 | 1.90E-22 | 1.11E-20 | up |
| LINC-PINT\|ENSG00000231721\|antisense | 1.36485166 | 4.48598803 | 1.71668161 | 9.94E-14 | 8.58E-13 | up |
| CELSR3-AS1\|ENSG00000228350\|antisense | 1.35314954 | 3.29027917 | 1.28188872 | 1.08E-08 | 4.13E-08 | up |
| PAN3-AS1\|ENSG00000261485\|antisense | 1.34906448 | 3.15953167 | 1.22775143 | 7.47E-10 | 3.38E-09 | up |
| MIR3142HG\|ENSG00000253522\|lincRNA | 1.34383027 | 5.01092996 | 1.89872745 | 6.78E-10 | 3.09E-09 | up |
| RP11-7O11.3\|ENSG00000237950\|antisense | 1.34060972 | 2.81804307 | 1.07180436 | 1.00E-07 | 3.34E-07 | up |
| FAM201A\|ENSG00000204860\|antisense | 1.32342463 | 3.2565035 | 1.29904774 | 1.21E-11 | 7.39E-11 | up |
| RP11-8P13.5\|ENSG00000274976\|sense_intronic | 1.32262887 | 2.95021925 | 1.15741387 | 1.01E-05 | 2.41E-05 | up |
| RP11-152N13.16\|ENSG00000272599\|antisense | 1.32193536 | 4.7126911 | 1.83389949 | 2.73E-18 | 5.31E-17 | up |
| RP4-563E14.1\|ENSG00000273759\|lincRNA | 1.31570453 | 4.55648735 | 1.79208652 | 8.49E-12 | 5.33E-11 | up |
| RP11-10N23.2\|ENSG00000253854\|antisense | 1.31347683 | 3.30970425 | 1.33331156 | 6.93E-07 | 2.01E-06 | up |
| RP11-324O2.3\|ENSG00000232934\|antisense | 1.3105028 | 2.74126232 | 1.06471995 | 0.01632201 | 0.02279921 | up |
| SCARNA9\|ENSG00000254911\|antisense | 1.29360485 | 17.0020937 | 3.71624352 | 9.23E-14 | 8.03E-13 | up |
| CTD-2649C14.2\|ENSG00000274460\|processed_transcript | 1.29159535 | 3.06774545 | 1.24802463 | 4.19E-09 | 1.71E-08 | up |
| RP11-260M2.1\|ENSG00000272807\|antisense | 1.28678231 | 3.37547867 | 1.3913241 | 1.68E-08 | 6.24E-08 | up |
| RP11-199F11.2\|ENSG00000262251\|sense_intronic | 1.27123423 | 4.13159685 | 1.70046961 | 1.96E-19 | 4.86E-18 | up |
| RP1-228H13.5\|ENSG00000260920\|sense_overlapping | 1.26958644 | 3.58768511 | 1.49869464 | 2.87E-18 | 5.53E-17 | up |
| LINC01503\|ENSG00000233901\|lincRNA | 1.26292317 | 2.68529843 | 1.08831556 | 1.64E-07 | 5.26E-07 | up |
| C1RL-AS1\|ENSG00000205885\|antisense | 1.25263263 | 2.55024285 | 1.02567127 | 9.03E-07 | 2.56E-06 | up |
| CTA-363E6.6\|ENSG00000260592\|antisense | 1.25100382 | 2.73736303 | 1.12970059 | 8.68E-07 | 2.47E-06 | up |
| FBXL19-AS1\|ENSG00000260852\|antisense | 1.23888224 | 2.56728896 | 1.05120663 | 7.62E-09 | 2.97E-08 | up |
| RP11-111M22.4\|ENSG00000272301\|lincRNA | 1.23705781 | 3.78019645 | 1.61154829 | 2.43E-10 | 1.19E-09 | up |
| RP11-245D16.4\|ENSG00000278238\|lincRNA | 1.23103258 | 3.16693735 | 1.36321939 | 3.69E-10 | 1.76E-09 | up |
| RP11-2B6.3\|ENSG00000269946\|lincRNA | 1.2226083 | 2.8979512 | 1.24507103 | 2.06E-08 | 7.54E-08 | up |
| RP11-273B20.1\|ENSG00000256967\|antisense | 1.21813907 | 3.40482638 | 1.48290239 | 2.95E-11 | 1.66E-10 | up |
| AC144652.1\|ENSG00000273117\|lincRNA | 1.21349673 | 3.15157597 | 1.37690321 | 5.73E-16 | 7.20E-15 | up |
| RP1-197B17.5\|ENSG00000276390\|sense_intronic | 1.20818357 | 2.75857014 | 1.19108099 | 1.37E-09 | 6.03E-09 | up |
| AF064858.8\|ENSG00000235888\|lincRNA | 1.18510014 | 4.07561344 | 1.78200825 | 0.00059671 | 0.00107518 | up |
| AC002116.7\|ENSG00000267698\|antisense | 1.18274099 | 2.9779305 | 1.33217591 | 7.30E-16 | 9.02E-15 | up |
| RP11-666O2.2\|ENSG00000275441\|antisense | 1.17919451 | 2.56135078 | 1.11910313 | 4.32E-08 | 1.51E-07 | up |
| CTA-984G1.5\|ENSG00000237015\|antisense | 1.17625636 | 2.52526722 | 1.10223354 | 8.73E-09 | 3.37E-08 | up |
| CTA-363E19.2\|ENSG00000272072\|antisense | 1.17425719 | 2.54826778 | 1.11776847 | 0.00684593 | 0.01021973 | up |
| KB-226F1.2\|ENSG00000272787\|lincRNA | 1.17121782 | 2.46745919 | 1.07501681 | 4.01E-08 | 1.41E-07 | up |
| AF064858.11\|ENSG00000237721\|lincRNA | 1.17108347 | 15.4505969 | 3.72174676 | 2.33E-07 | 7.29E-07 | up |
| DICER1-AS1\|ENSG00000235706\|antisense | 1.16658571 | 2.39363229 | 1.03690924 | 1.35E-11 | 8.14E-11 | up |
| CTD-2547G23.4\|ENSG00000274925\|lincRNA | 1.16542404 | 2.91775592 | 1.32400422 | 5.17E-11 | 2.81E-10 | up |
| AC093620.5\|ENSG00000241269\|antisense | 1.16359665 | 4.17329706 | 1.84259657 | 2.92E-13 | 2.34E-12 | up |
| RP11-650L12.2\|ENSG00000261762\|antisense | 1.15518032 | 2.5541003 | 1.14469711 | 1.21E-09 | 5.34E-09 | up |
| RP11-135A1.2\|ENSG00000232874\|antisense | 1.15470554 | 3.31428779 | 1.52117388 | 1.10E-08 | 4.18E-08 | up |
| CTD-2371O3.3\|ENSG00000268403\|antisense | 1.15202548 | 3.01446726 | 1.38773044 | 3.32E-13 | 2.65E-12 | up |
| RP4-694B14.8\|ENSG00000278383\|antisense | 1.14364728 | 3.58165699 | 1.64698501 | 5.20E-10 | 2.41E-09 | up |
| RP11-235E17.6\|ENSG00000262903\|antisense | 1.1408832 | 2.98832487 | 1.3891859 | 1.13E-10 | 5.79E-10 | up |
| LINC01138\|ENSG00000274020\|lincRNA | 1.13746436 | 2.54000056 | 1.15900747 | 1.62E-14 | 1.58E-13 | up |
| AL133243.1\|ENSG00000276334\|sense_intronic | 1.1357266 | 3.52360878 | 1.63343817 | 1.54E-06 | 4.23E-06 | up |
| RP11-649A18.4\|ENSG00000263786\|sense_intronic | 1.13531663 | 2.43244053 | 1.09930982 | 3.45E-05 | 7.61E-05 | up |
| RP11-473M20.9\|ENSG00000262370\|lincRNA | 1.12807281 | 6.80588229 | 2.59292201 | 1.50E-07 | 4.86E-07 | up |
| RP4-635E18.6\|ENSG00000230337\|antisense | 1.12518203 | 2.37059856 | 1.07509296 | 5.60E-09 | 2.24E-08 | up |
| CTD-2349P21.10\|ENSG00000265791\|sense_intronic | 1.1238628 | 3.98659078 | 1.8266896 | 2.02E-09 | 8.66E-09 | up |
| RP11-680A11.5\|ENSG00000257605\|antisense | 1.12238192 | 3.39028655 | 1.59484355 | 1.59E-21 | 7.15E-20 | up |
| RP5-855D21.3\|ENSG00000272812\|sense_intronic | 1.12108494 | 2.50789872 | 1.1615835 | 3.88E-08 | 1.37E-07 | up |
| OGFRP1\|ENSG00000182057\|lincRNA | 1.11607953 | 2.44655407 | 1.13231133 | 2.09E-15 | 2.39E-14 | up |
| RP11-203M5.7\|ENSG00000258515\|antisense | 1.11041663 | 2.51530443 | 1.17963194 | 4.28E-06 | 1.09E-05 | up |
| LINC01355\|ENSG00000261326\|lincRNA | 1.11013262 | 2.89850507 | 1.38457697 | 3.85E-09 | 1.58E-08 | up |
| CTD-2517M22.17\|ENSG00000265393\|antisense | 1.10397196 | 3.00265892 | 1.44353707 | 1.01E-07 | 3.35E-07 | up |
| RP11-523H24.3\|ENSG00000226352\|antisense | 1.09103357 | 2.73108064 | 1.32377643 | 1.67E-06 | 4.54E-06 | up |
| RP11-1275H24.1\|ENSG00000234432\|lincRNA | 1.08690018 | 3.06373418 | 1.49507168 | 3.26E-17 | 5.18E-16 | up |
| RP11-93H24.3\|ENSG00000273723\|lincRNA | 1.07627831 | 4.26688904 | 1.98713341 | 1.74E-16 | 2.41E-15 | up |
| PVT1\|ENSG00000249859\|lincRNA | 1.07221948 | 8.71379238 | 3.02270049 | 3.36E-26 | 1.27E-23 | up |
| ATP2A1-AS1\|ENSG00000260442\|antisense | 1.0669071 | 4.16443354 | 1.96468571 | 3.05E-15 | 3.37E-14 | up |
| LINC00853\|ENSG00000224805\|antisense | 1.06673609 | 2.71176864 | 1.3460308 | 1.49E-11 | 8.83E-11 | up |
| LINC01123\|ENSG00000204588\|lincRNA | 1.06528129 | 2.15842334 | 1.01874343 | 6.44E-07 | 1.88E-06 | up |
| RP5-1184F4.5\|ENSG00000236772\|antisense | 1.06012332 | 2.25983376 | 1.09198455 | 0.00032397 | 0.00061489 | up |
| RP11-172H24.4\|ENSG00000278291\|antisense | 1.05894183 | 2.91400992 | 1.46038244 | 2.34E-13 | 1.90E-12 | up |
| HCG16\|ENSG00000244349\|antisense | 1.05362767 | 2.2533996 | 1.09673804 | 1.81E-06 | 4.89E-06 | up |
| MAFG-AS1\|ENSG00000265688\|antisense | 1.05317543 | 9.96847076 | 3.24262644 | 1.18E-24 | 1.65E-22 | up |
| UBAC2-AS1\|ENSG00000228889\|lincRNA | 1.05194128 | 2.42128311 | 1.20271761 | 2.09E-12 | 1.46E-11 | up |
| CTD-2006C1.2\|ENSG00000219665\|processed_transcript | 1.0357993 | 2.28864981 | 1.14375224 | 7.77E-16 | 9.54E-15 | up |
| RP1-198K11.5\|ENSG00000275457\|antisense | 1.03415632 | 2.52021945 | 1.28509509 | 1.30E-13 | 1.10E-12 | up |
| RP11-248J18.2\|ENSG00000269906\|sense_intronic | 1.03340623 | 2.30003792 | 1.15425016 | 2.83E-06 | 7.41E-06 | up |
| RP11-337N6.3\|ENSG00000280374\|sense_intronic | 1.03284143 | 2.48566835 | 1.26701504 | 7.14E-09 | 2.80E-08 | up |
| AP001469.9\|ENSG00000239415\|antisense | 1.02764796 | 3.13327174 | 1.60832377 | 5.90E-20 | 1.72E-18 | up |
| AC008746.12\|ENSG00000267838\|lincRNA | 1.02694684 | 3.3123501 | 1.68949366 | 1.97E-13 | 1.63E-12 | up |
| SP2-AS1\|ENSG00000234494\|antisense | 1.02103581 | 2.09286588 | 1.0354464 | 9.77E-11 | 5.08E-10 | up |
| CTD-2555C10.3\|ENSG00000259230\|lincRNA | 1.02087607 | 2.40342485 | 1.23528396 | 1.44E-07 | 4.67E-07 | up |
| MMP25-AS1\|ENSG00000261971\|antisense | 1.01570082 | 2.81910922 | 1.47276386 | 1.55E-12 | 1.10E-11 | up |
| RP11-112J3.16\|ENSG00000227388\|lincRNA | 1.00504678 | 2.67547235 | 1.41253096 | 8.70E-14 | 7.63E-13 | up |
| RP3-337H4.8\|ENSG00000203362\|antisense | 1.00499014 | 2.02235017 | 1.00885148 | 1.80E-10 | 8.97E-10 | up |
| FMR1-IT1\|ENSG00000236337\|sense_intronic | 0.99536357 | 2.24198516 | 1.17148124 | 0.0013458 | 0.00226984 | up |
| RP4-681N20.5\|ENSG00000275582\|antisense | 0.99218972 | 2.2671774 | 1.19220936 | 0.00013458 | 0.00027118 | up |
| RP11-503E24.2\|ENSG00000254165\|antisense | 0.98481372 | 3.18419778 | 1.69300718 | 1.54E-05 | 3.58E-05 | up |
| RP5-1061H20.4\|ENSG00000177788\|lincRNA | 0.97866109 | 2.5176645 | 1.3632048 | 5.48E-12 | 3.58E-11 | up |
| NDUFB2-AS1\|ENSG00000240889\|antisense | 0.97070092 | 2.03380841 | 1.06708502 | 4.82E-17 | 7.39E-16 | up |
| LINC00115\|ENSG00000225880\|lincRNA | 0.97046732 | 1.98496276 | 1.03236041 | 3.25E-11 | 1.81E-10 | up |
| RP11-216L13.19\|ENSG00000273066\|processed_transcript | 0.96936353 | 3.71747067 | 1.93921164 | 5.90E-13 | 4.54E-12 | up |
| RP11-277P12.20\|ENSG00000245648\|antisense | 0.96880233 | 4.73525098 | 2.28916665 | 3.20E-08 | 1.14E-07 | up |
| NKILA\|ENSG00000278709\|antisense | 0.96615241 | 3.87803298 | 2.00500238 | 5.79E-12 | 3.76E-11 | up |
| C6orf99\|ENSG00000203711\|lincRNA | 0.96477774 | 2.13176732 | 1.14378145 | 3.23E-11 | 1.80E-10 | up |
| CTC-246B18.10\|ENSG00000269246\|lincRNA | 0.96055258 | 2.02919498 | 1.078971 | 0.00037478 | 0.00070127 | up |
| CTC-542B22.2\|ENSG00000263990\|lincRNA | 0.95625242 | 2.35963878 | 1.30310263 | 7.23E-08 | 2.47E-07 | up |
| RP11-386I14.4\|ENSG00000273338\|antisense | 0.95459379 | 4.46134636 | 2.22452031 | 7.38E-08 | 2.52E-07 | up |
| WNT5A-AS1\|ENSG00000244586\|antisense | 0.95310874 | 2.11091314 | 1.14715448 | 0.0016122 | 0.00268835 | up |
| CTC-435M10.12\|ENSG00000277744\|sense_intronic | 0.95152343 | 2.48898533 | 1.38724664 | 1.30E-11 | 7.87E-11 | up |
| DLEU2\|ENSG00000231607\|antisense | 0.95027359 | 3.50816774 | 1.88430289 | 4.85E-14 | 4.39E-13 | up |
| RP11-234K24.6\|ENSG00000278035\|lincRNA | 0.9419552 | 2.02178462 | 1.10189897 | 0.00287059 | 0.00458619 | up |
| ALG13-AS1\|ENSG00000229487\|antisense | 0.93684423 | 2.87187767 | 1.6161132 | 0.01628909 | 0.02275921 | up |
| LINC01232\|ENSG00000280734\|processed_transcript | 0.92924006 | 3.42623453 | 1.88250066 | 3.70E-20 | 1.16E-18 | up |
| BZRAP1-AS1\|ENSG00000265148\|antisense | 0.92500319 | 2.8350955 | 1.61586708 | 1.21E-11 | 7.39E-11 | up |
| RP11-524F11.1\|ENSG00000265511\|antisense | 0.92176407 | 1.91710671 | 1.05646121 | 4.50E-07 | 1.34E-06 | up |
| RP11-572O17.1\|ENSG00000270195\|lincRNA | 0.91764327 | 2.68300888 | 1.54784651 | 3.21E-14 | 2.99E-13 | up |
| RP11-275I14.4\|ENSG00000234478\|antisense | 0.91715998 | 2.48447929 | 1.43769821 | 1.82E-06 | 4.90E-06 | up |
| MCM3AP-AS1\|ENSG00000215424\|antisense | 0.90663152 | 1.93055113 | 1.09042454 | 3.73E-15 | 4.08E-14 | up |
| RP11-523H20.3\|ENSG00000272183\|antisense | 0.90307691 | 2.22198368 | 1.29892746 | 2.59E-10 | 1.26E-09 | up |
| RP13-638C3.3\|ENSG00000262147\|antisense | 0.90036272 | 2.56059029 | 1.50789821 | 2.50E-12 | 1.72E-11 | up |
| RP11-261P9.4\|ENSG00000254419\|sense_intronic | 0.89633601 | 3.04134839 | 1.76259953 | 1.92E-09 | 8.23E-09 | up |
| RP11-334C17.5\|ENSG00000262580\|antisense | 0.89488342 | 2.39812931 | 1.4221378 | 9.23E-12 | 5.75E-11 | up |
| RP4-616B8.5\|ENSG00000274825\|lincRNA | 0.8948834 | 2.35386947 | 1.3952627 | 9.10E-13 | 6.76E-12 | up |
| LA16c-306A4.1\|ENSG00000260022\|antisense | 0.88276373 | 2.68971792 | 1.60735562 | 1.55E-11 | 9.14E-11 | up |
| AC104667.3\|ENSG00000234949\|antisense | 0.88113234 | 2.34027426 | 1.40924699 | 1.23E-10 | 6.28E-10 | up |
| RP11-98D18.9\|ENSG00000203288\|antisense | 0.88058072 | 1.96872985 | 1.16073799 | 1.82E-09 | 7.82E-09 | up |
| RP11-1275H24.2\|ENSG00000272953\|lincRNA | 0.87188267 | 2.41594751 | 1.47038321 | 3.18E-10 | 1.53E-09 | up |
| CTD-2095E4.5\|ENSG00000277511\|lincRNA | 0.86927276 | 2.29910878 | 1.40319389 | 3.53E-09 | 1.46E-08 | up |
| RP11-351J23.1\|ENSG00000203688\|lincRNA | 0.84360982 | 3.46006422 | 2.03615102 | 1.13E-12 | 8.24E-12 | up |
| RP11-626G11.1\|ENSG00000261357\|lincRNA | 0.84292645 | 1.89093317 | 1.16561972 | 4.24E-07 | 1.27E-06 | up |
| RP6-114E22.1\|ENSG00000266869\|lincRNA | 0.83830132 | 2.64151785 | 1.65582636 | 0.00656769 | 0.00982928 | up |
| RP11-634H22.1\|ENSG00000273391\|antisense | 0.83600445 | 2.37543349 | 1.50660829 | 1.93E-14 | 1.85E-13 | up |
| RP11-272L14.2\|ENSG00000274204\|sense_intronic | 0.8277635 | 3.29910227 | 1.99478296 | 3.85E-07 | 1.16E-06 | up |
| RP11-11N5.3\|ENSG00000272218\|lincRNA | 0.82168324 | 6.23789954 | 2.92440607 | 0.01091557 | 0.01572804 | up |
| RP11-425M5.7\|ENSG00000276603\|sense_intronic | 0.81932711 | 3.70043652 | 2.17518401 | 4.99E-09 | 2.02E-08 | up |
| RP11-368I7.4\|ENSG00000260259\|lincRNA | 0.81828682 | 1.83174694 | 1.16254168 | 4.33E-11 | 2.38E-10 | up |
| CTB-119C2.1\|ENSG00000273237\|antisense | 0.81407549 | 1.93370189 | 1.24813091 | 0.00664685 | 0.00993934 | up |
| CASC8\|ENSG00000246228\|antisense | 0.81267795 | 3.42809313 | 2.07665064 | 2.19E-17 | 3.58E-16 | up |
| RP11-796E10.1\|ENSG00000270460\|lincRNA | 0.81179133 | 5.17834403 | 2.67330998 | 5.78E-20 | 1.69E-18 | up |
| CAPN10-AS1\|ENSG00000260942\|antisense | 0.80741348 | 2.17308823 | 1.42836718 | 2.65E-17 | 4.29E-16 | up |
| RP5-1028K7.2\|ENSG00000266088\|lincRNA | 0.80711717 | 2.30925967 | 1.51658037 | 1.71E-13 | 1.43E-12 | up |
| RP11-321F8.4\|ENSG00000257543\|lincRNA | 0.8008768 | 1.8445074 | 1.20358335 | 9.45E-07 | 2.67E-06 | up |
| AC115522.3\|ENSG00000268601\|lincRNA | 0.79933985 | 1.67478709 | 1.06709677 | 2.16E-06 | 5.74E-06 | up |
| FAM95C\|ENSG00000273036\|lincRNA | 0.79616204 | 1.88723747 | 1.24514198 | 5.04E-05 | 0.0001084 | up |
| ELFN1-AS1\|ENSG00000236081\|lincRNA | 0.78967138 | 27.9442022 | 5.14515277 | 5.01E-24 | 5.31E-22 | up |
| RP11-332H14.2\|ENSG00000272994\|lincRNA | 0.78918963 | 1.83827555 | 1.21990914 | 1.01E-11 | 6.27E-11 | up |
| CTD-2196E14.6\|ENSG00000260751\|sense_intronic | 0.78871099 | 1.928816 | 1.29014687 | 3.74E-10 | 1.77E-09 | up |
| AC114730.11\|ENSG00000235351\|antisense | 0.78450845 | 1.61924497 | 1.04546038 | 1.26E-05 | 2.97E-05 | up |
| AC002128.5\|ENSG00000271366\|antisense | 0.7843162 | 1.6997482 | 1.11581373 | 0.00044054 | 0.00081539 | up |
| RP11-396C23.2\|ENSG00000225518\|lincRNA | 0.78176149 | 2.82234377 | 1.8520933 | 1.64E-15 | 1.93E-14 | up |
| CTD-2527I21.14\|ENSG00000271032\|antisense | 0.78162496 | 3.53527402 | 2.1772736 | 4.48E-15 | 4.85E-14 | up |
| SH3PXD2A-AS1\|ENSG00000280693\|antisense | 0.77889976 | 6.87153304 | 3.14112243 | 7.65E-18 | 1.36E-16 | up |
| RP11-351C21.2\|ENSG00000274943\|sense_intronic | 0.77759865 | 2.80023908 | 1.84845238 | 1.68E-12 | 1.20E-11 | up |
| STAG3L5P-PVRIG2P-PILRB\|ENSG00000272752\|processed_transcript | 0.77588959 | 2.0748268 | 1.41906764 | 6.00E-13 | 4.60E-12 | up |
| RP11-309L24.4\|ENSG00000272899\|lincRNA | 0.77512648 | 4.18340745 | 2.43217488 | 1.73E-20 | 6.02E-19 | up |
| RP11-849F2.9\|ENSG00000269947\|lincRNA | 0.77476434 | 1.97477559 | 1.34985926 | 4.53E-14 | 4.12E-13 | up |
| AC073635.5\|ENSG00000250990\|antisense | 0.76956808 | 1.69088365 | 1.13565652 | 0.01760468 | 0.02441717 | up |
| LA16c-313D11.12\|ENSG00000261659\|processed_transcript | 0.76907495 | 1.83046126 | 1.25101113 | 1.64E-11 | 9.67E-11 | up |
| RP11-2E11.9\|ENSG00000270953\|antisense | 0.76827579 | 2.19034699 | 1.51146324 | 1.43E-11 | 8.58E-11 | up |
| RP11-539L10.2\|ENSG00000246526\|lincRNA | 0.76521892 | 1.57977802 | 1.0457774 | 2.27E-09 | 9.69E-09 | up |
| RP11-277B15.3\|ENSG00000272205\|lincRNA | 0.76097717 | 1.78153322 | 1.22719432 | 1.82E-09 | 7.82E-09 | up |
| AP006621.9\|ENSG00000269915\|antisense | 0.75916628 | 1.75237949 | 1.20682742 | 0.00013895 | 0.00027931 | up |
| RP11-415J8.3\|ENSG00000225313\|antisense | 0.75650455 | 1.72020941 | 1.18516353 | 1.03E-05 | 2.47E-05 | up |
| RP11-148K1.12\|ENSG00000244151\|antisense | 0.75564504 | 2.53145028 | 1.74418354 | 2.07E-16 | 2.82E-15 | up |
| RP11-305E6.4\|ENSG00000259994\|sense_overlapping | 0.75304487 | 2.06088311 | 1.45245495 | 2.72E-15 | 3.02E-14 | up |
| RP11-585P4.5\|ENSG00000257497\|antisense | 0.74963878 | 2.94548296 | 1.97423672 | 7.95E-15 | 8.20E-14 | up |
| AC012360.6\|ENSG00000238273\|antisense | 0.74701821 | 1.96041739 | 1.39194553 | 0.00279739 | 0.0044787 | up |
| RP11-727A23.5\|ENSG00000247137\|processed_transcript | 0.74612994 | 1.58466746 | 1.08668132 | 6.64E-08 | 2.29E-07 | up |
| LINC01124\|ENSG00000222033\|lincRNA | 0.74079204 | 4.65011762 | 2.65012671 | 1.60E-15 | 1.89E-14 | up |
| RP11-18H7.1\|ENSG00000242622\|lincRNA | 0.73998768 | 1.67436343 | 1.17803955 | 9.28E-11 | 4.86E-10 | up |
| LA16c-390E6.4\|ENSG00000260051\|antisense | 0.7384407 | 2.57680007 | 1.80302664 | 4.55E-12 | 3.01E-11 | up |
| RP4-724E16.2\|ENSG00000197670\|antisense | 0.73333222 | 1.64625435 | 1.16664841 | 1.23E-05 | 2.92E-05 | up |
| AC004066.3\|ENSG00000250522\|antisense | 0.73103229 | 1.47938721 | 1.01699266 | 0.0012217 | 0.00207771 | up |
| RP11-498C9.12\|ENSG00000264769\|antisense | 0.72609594 | 1.57435886 | 1.11653233 | 0.0009837 | 0.0017097 | up |
| RP3-339A18.6\|ENSG00000233250\|antisense | 0.72440221 | 2.03421156 | 1.48960688 | 4.22E-09 | 1.72E-08 | up |
| GATA2-AS1\|ENSG00000244300\|antisense | 0.72156496 | 5.61077297 | 2.95899835 | 3.37E-14 | 3.13E-13 | up |
| RP11-373D23.2\|ENSG00000270640\|sense_intronic | 0.72053581 | 4.52091064 | 2.64947137 | 1.18E-05 | 2.81E-05 | up |
| N4BP2L2-IT2\|ENSG00000281026\|sense_intronic | 0.71833594 | 2.44520602 | 1.76722542 | 3.10E-06 | 8.07E-06 | up |
| AC104088.1\|ENSG00000232555\|lincRNA | 0.71732887 | 2.55970536 | 1.83527116 | 4.68E-11 | 2.56E-10 | up |
| RP11-686G8.2\|ENSG00000258099\|antisense | 0.71529424 | 1.61528906 | 1.17518363 | 3.17E-11 | 1.77E-10 | up |
| RP11-278A23.2\|ENSG00000260671\|sense_intronic | 0.70598448 | 2.20739728 | 1.64463793 | 0.00666064 | 0.00995715 | up |
| RP11-496I9.1\|ENSG00000254815\|antisense | 0.70116731 | 2.34707488 | 1.74303324 | 1.19E-10 | 6.08E-10 | up |
| RP11-495P10.1\|ENSG00000231551\|lincRNA | 0.69680437 | 1.5247224 | 1.12972103 | 0.00534808 | 0.00811629 | up |
| RP11-572O6.1\|ENSG00000271973\|lincRNA | 0.69626918 | 1.55165938 | 1.15609482 | 3.50E-05 | 7.70E-05 | up |
| RP1-92O14.6\|ENSG00000229431\|antisense | 0.69433842 | 1.7206984 | 1.30928333 | 4.04E-10 | 1.90E-09 | up |
| RP11-218F10.3\|ENSG00000273449\|lincRNA | 0.69117492 | 2.00722998 | 1.53808314 | 1.73E-09 | 7.51E-09 | up |
| RP11-46F15.2\|ENSG00000238260\|antisense | 0.69064057 | 1.44586416 | 1.06592503 | 8.28E-06 | 2.01E-05 | up |
| RP11-216B9.9\|ENSG00000280758\|antisense | 0.69046021 | 1.46803986 | 1.08826096 | 3.33E-07 | 1.01E-06 | up |
| LINC01311\|ENSG00000260924\|antisense | 0.68700939 | 2.40808324 | 1.80948353 | 6.57E-19 | 1.47E-17 | up |
| RP11-480A16.1\|ENSG00000260261\|lincRNA | 0.68596684 | 2.02988825 | 1.56518957 | 1.73E-10 | 8.62E-10 | up |
| LINC00114\|ENSG00000223806\|lincRNA | 0.684676 | 2.27929362 | 1.73509344 | 2.58E-06 | 6.82E-06 | up |
| CTD-3138B18.6\|ENSG00000268201\|sense_intronic | 0.67912171 | 1.91913188 | 1.49871181 | 5.58E-06 | 1.40E-05 | up |
| GK-AS1\|ENSG00000243055\|antisense | 0.67764961 | 2.95884274 | 2.12642161 | 2.49E-05 | 5.61E-05 | up |
| CTC-1337H24.4\|ENSG00000275484\|lincRNA | 0.6731703 | 1.61198726 | 1.25979692 | 2.80E-08 | 1.00E-07 | up |
| RP11-29G8.3\|ENSG00000261553\|processed_transcript | 0.6673133 | 2.20208296 | 1.72243266 | 3.83E-15 | 4.18E-14 | up |
| AC016735.2\|ENSG00000231826\|lincRNA | 0.66475952 | 10.6370145 | 4.00011695 | 0.00028386 | 0.0005454 | up |
| AC005519.4\|ENSG00000258559\|sense_overlapping | 0.66299617 | 1.50525056 | 1.18293122 | 1.39E-07 | 4.53E-07 | up |
| SPRY4-IT1\|ENSG00000281881\|lincRNA | 0.66091879 | 1.4999336 | 1.18235371 | 0.00442163 | 0.00679984 | up |
| RP13-131K19.6\|ENSG00000272434\|antisense | 0.6603715 | 1.7841279 | 1.43386928 | 0.0007878 | 0.00138919 | up |
| LINC01094\|ENSG00000251442\|lincRNA | 0.65973291 | 1.53280417 | 1.21621941 | 0.00179751 | 0.00297583 | up |
| AC084117.3\|ENSG00000256006\|sense_intronic | 0.65968126 | 2.09686847 | 1.66839535 | 8.14E-06 | 1.98E-05 | up |
| CTD-3222D19.12\|ENSG00000269399\|lincRNA | 0.65739338 | 1.84632466 | 1.48982743 | 2.60E-17 | 4.23E-16 | up |
| RP11-529K1.2\|ENSG00000261777\|processed_transcript | 0.65661519 | 1.43647577 | 1.12941362 | 1.76E-08 | 6.49E-08 | up |
| RP11-244M2.1\|ENSG00000267374\|lincRNA | 0.6511443 | 1.53141801 | 1.23381894 | 7.58E-06 | 1.85E-05 | up |
| RP11-214N9.1\|ENSG00000271855\|lincRNA | 0.65052897 | 1.39755907 | 1.10322405 | 3.77E-10 | 1.79E-09 | up |
| XXbac-BPG252P9.9\|ENSG00000272540\|antisense | 0.65048708 | 1.40399234 | 1.10994275 | 3.62E-09 | 1.49E-08 | up |
| RP1-167A14.2\|ENSG00000227598\|antisense | 0.64929168 | 1.65774584 | 1.3522842 | 2.80E-08 | 1.00E-07 | up |
| LINC01235\|ENSG00000270547\|lincRNA | 0.64871271 | 1.9486851 | 1.58684938 | 5.43E-07 | 1.60E-06 | up |
| RP11-186N15.3\|ENSG00000228302\|antisense | 0.64679633 | 1.46353644 | 1.17807527 | 8.56E-12 | 5.37E-11 | up |
| RP13-942N8.1\|ENSG00000256092\|lincRNA | 0.64459421 | 1.69438182 | 1.39429589 | 3.67E-15 | 4.02E-14 | up |
| RP11-554E23.4\|ENSG00000257222\|antisense | 0.64158494 | 1.69901607 | 1.40498731 | 3.66E-11 | 2.02E-10 | up |
| CCAT1\|ENSG00000247844\|lincRNA | 0.64049289 | 18.3369106 | 4.83942422 | 2.06E-25 | 4.75E-23 | up |
| RP11-755F10.3\|ENSG00000254461\|antisense | 0.6403908 | 1.30750558 | 1.02979262 | 0.00083309 | 0.00146427 | up |
| RP11-324E6.10\|ENSG00000274191\|lincRNA | 0.63988018 | 1.50109496 | 1.23014157 | 1.02E-07 | 3.37E-07 | up |
| LL0XNC01-237H1.2\|ENSG00000239407\|lincRNA | 0.63457083 | 1.80067308 | 1.50468317 | 4.75E-09 | 1.93E-08 | up |
| RHOA-IT1\|ENSG00000235908\|sense_intronic | 0.63365009 | 1.30078549 | 1.03762479 | 0.00154178 | 0.00257903 | up |
| NCBP2-AS1\|ENSG00000225578\|antisense | 0.63275897 | 1.66185361 | 1.39306534 | 7.38E-07 | 2.13E-06 | up |
| RP13-270P17.1\|ENSG00000264235\|antisense | 0.63159459 | 1.43773456 | 1.18672663 | 9.05E-08 | 3.04E-07 | up |
| CTD-2537I9.13\|ENSG00000267096\|sense_intronic | 0.63024259 | 1.30052726 | 1.04511749 | 6.80E-06 | 1.68E-05 | up |
| RP1-102K2.8\|ENSG00000268812\|antisense | 0.6294587 | 2.29167701 | 1.8642201 | 3.92E-16 | 5.12E-15 | up |
| RP5-908M14.10\|ENSG00000275437\|sense_intronic | 0.62911443 | 2.32276434 | 1.88444842 | 4.45E-16 | 5.68E-15 | up |
| RP11-1020A11.1\|ENSG00000269894\|sense_intronic | 0.62879629 | 1.58596783 | 1.3346989 | 7.04E-07 | 2.04E-06 | up |
| RP11-358N2.2\|ENSG00000277692\|lincRNA | 0.62509445 | 1.39016581 | 1.15311087 | 1.00E-05 | 2.40E-05 | up |
| RP11-88E10.5\|ENSG00000274922\|lincRNA | 0.62485692 | 1.92359091 | 1.62220423 | 4.22E-10 | 1.98E-09 | up |
| RP11-680G24.6\|ENSG00000275910\|antisense | 0.62322318 | 1.50260689 | 1.26964683 | 0.00122933 | 0.00208935 | up |
| RP11-143J12.3\|ENSG00000263847\|antisense | 0.62001204 | 1.77851345 | 1.52030375 | 1.64E-05 | 3.80E-05 | up |
| RASAL2-AS1\|ENSG00000224687\|lincRNA | 0.61973794 | 1.31411501 | 1.08436136 | 2.92E-13 | 2.34E-12 | up |
| RP11-350J20.12\|ENSG00000273132\|antisense | 0.61810045 | 16.2605791 | 4.71739352 | 8.25E-22 | 4.05E-20 | up |
| RP11-568J23.5\|ENSG00000270184\|antisense | 0.61729617 | 1.55161786 | 1.32973855 | 8.44E-08 | 2.85E-07 | up |
| RP11-715J22.3\|ENSG00000260095\|antisense | 0.61682151 | 1.68120183 | 1.44656795 | 8.00E-10 | 3.62E-09 | up |
| RP11-295D4.4\|ENSG00000277170\|sense_intronic | 0.61336882 | 1.29586252 | 1.07908594 | 0.0002503 | 0.00048459 | up |
| CTD-3001H11.2\|ENSG00000277383\|antisense | 0.61297594 | 1.3442492 | 1.13289826 | 1.16E-07 | 3.81E-07 | up |
| RBPMS-AS1\|ENSG00000254109\|antisense | 0.6129236 | 1.91980204 | 1.6471784 | 2.92E-11 | 1.65E-10 | up |
| STX18-AS1\|ENSG00000247708\|antisense | 0.61200332 | 1.31847334 | 1.10725701 | 2.99E-16 | 4.00E-15 | up |
| RP1-315G1.3\|ENSG00000232412\|lincRNA | 0.6112376 | 1.589648 | 1.37890215 | 7.48E-07 | 2.15E-06 | up |
| RP11-79P5.9\|ENSG00000272525\|lincRNA | 0.61109617 | 1.44157989 | 1.23817945 | 9.49E-11 | 4.95E-10 | up |
| CTD-2521M24.6\|ENSG00000269481\|antisense | 0.61039952 | 1.48114124 | 1.27888348 | 1.33E-12 | 9.59E-12 | up |
| AATBC\|ENSG00000215458\|antisense | 0.61008846 | 1.28839136 | 1.07848056 | 2.78E-05 | 6.22E-05 | up |
| RP11-66N24.6\|ENSG00000274002\|lincRNA | 0.60952371 | 1.799526 | 1.56186269 | 5.52E-06 | 1.38E-05 | up |
| CTD-2325P2.4\|ENSG00000259038\|antisense | 0.60665049 | 1.74491159 | 1.52421646 | 2.87E-07 | 8.81E-07 | up |
| GAPLINC\|ENSG00000266835\|lincRNA | 0.60472523 | 1.96242556 | 1.69828625 | 3.52E-13 | 2.78E-12 | up |
| RP11-496H1.2\|ENSG00000273308\|lincRNA | 0.60216728 | 1.46786139 | 1.28547953 | 0.00011999 | 0.00024417 | up |
| RP11-762I7.4\|ENSG00000257509\|sense_intronic | 0.60028061 | 1.71517076 | 1.51464326 | 2.41E-06 | 6.38E-06 | up |
| LA16c-325D7.2\|ENSG00000263280\|lincRNA | 0.59887824 | 1.25685273 | 1.069481 | 2.51E-05 | 5.66E-05 | up |
| RP5-901A4.1\|ENSG00000255306\|antisense | 0.59597624 | 1.47999037 | 1.31226106 | 1.03E-10 | 5.35E-10 | up |
| GAS6-AS1\|ENSG00000233695\|antisense | 0.59490553 | 7.19542087 | 3.59634658 | 6.14E-20 | 1.78E-18 | up |
| RP11-38M8.1\|ENSG00000273297\|lincRNA | 0.59222721 | 1.3068252 | 1.14184351 | 0.00064417 | 0.00115481 | up |
| RP11-37B2.1\|ENSG00000251136\|lincRNA | 0.59192553 | 1.83451027 | 1.6319074 | 2.16E-13 | 1.77E-12 | up |
| RP11-545P7.9\|ENSG00000277566\|sense_intronic | 0.58853338 | 1.24650759 | 1.08269553 | 0.0090551 | 0.01324426 | up |
| LINC00630\|ENSG00000223546\|lincRNA | 0.58709879 | 1.40662229 | 1.26055979 | 6.95E-14 | 6.16E-13 | up |
| AP001628.6\|ENSG00000225218\|lincRNA | 0.58630911 | 2.35735411 | 2.0074351 | 8.12E-10 | 3.67E-09 | up |
| LL22NC03-2H8.5\|ENSG00000274422\|lincRNA | 0.58470242 | 1.33344374 | 1.18938249 | 1.36E-05 | 3.19E-05 | up |
| CH507-513H4.5\|ENSG00000281383\|lincRNA | 0.58414007 | 1.96915928 | 1.75319355 | 0.03697602 | 0.04855335 | up |
| PIK3IP1-AS1\|ENSG00000228839\|antisense | 0.58352364 | 1.46201175 | 1.32509189 | 2.21E-10 | 1.09E-09 | up |
| AP000692.9\|ENSG00000228107\|sense_overlapping | 0.58208257 | 1.52826492 | 1.39259894 | 1.98E-06 | 5.31E-06 | up |
| RP11-119J18.1\|ENSG00000250378\|lincRNA | 0.58168198 | 1.82145059 | 1.64678534 | 0.01132049 | 0.0162805 | up |
| LINC00893\|ENSG00000241769\|antisense | 0.58057237 | 1.25426193 | 1.11129085 | 1.03E-06 | 2.89E-06 | up |
| TRPM2-AS\|ENSG00000230061\|antisense | 0.58051449 | 6.34070844 | 3.44924006 | 9.13E-15 | 9.25E-14 | up |
| CTD-3099C6.9\|ENSG00000269825\|sense_intronic | 0.57915139 | 1.80400831 | 1.63919356 | 0.01446534 | 0.02038839 | up |
| RP11-121A14.2\|ENSG00000235204\|antisense | 0.57785365 | 1.89131317 | 1.7106122 | 5.46E-06 | 1.37E-05 | up |
| RP3-431P23.5\|ENSG00000269155\|lincRNA | 0.57719348 | 1.35335003 | 1.22940812 | 3.62E-05 | 7.95E-05 | up |
| CTD-2286N8.2\|ENSG00000267655\|sense_intronic | 0.57223288 | 1.16662403 | 1.02766538 | 5.68E-07 | 1.67E-06 | up |
| RP11-685N10.1\|ENSG00000255893\|antisense | 0.57094274 | 1.69477941 | 1.56967955 | 0.00755276 | 0.01119295 | up |
| RP11-1348G14.8\|ENSG00000275807\|antisense | 0.56923964 | 1.7364933 | 1.6090688 | 2.23E-14 | 2.11E-13 | up |
| RP11-242C19.2\|ENSG00000241886\|sense_intronic | 0.56740113 | 2.62307371 | 2.20881742 | 3.45E-05 | 7.61E-05 | up |
| RP11-492I21.1\|ENSG00000228686\|antisense | 0.56654379 | 1.77033995 | 1.64376705 | 0.00012232 | 0.00024806 | up |
| MIRLET7BHG\|ENSG00000197182\|lincRNA | 0.56605592 | 1.538654 | 1.44265235 | 1.55E-11 | 9.14E-11 | up |
| RP11-95P2.3\|ENSG00000263105\|lincRNA | 0.56406818 | 1.46005371 | 1.37207998 | 1.68E-07 | 5.38E-07 | up |
| RP11-727F15.9\|ENSG00000256690\|antisense | 0.56311436 | 2.19915804 | 1.96545145 | 3.89E-19 | 9.12E-18 | up |
| AP001469.7\|ENSG00000228137\|antisense | 0.55957229 | 1.45011941 | 1.37377527 | 7.80E-09 | 3.04E-08 | up |
| RP11-1029J19.4\|ENSG00000258919\|lincRNA | 0.55945776 | 6.73208802 | 3.58895294 | 1.25E-21 | 5.86E-20 | up |
| LINC01342\|ENSG00000223823\|lincRNA | 0.55842651 | 1.39302391 | 1.31878069 | 9.97E-08 | 3.33E-07 | up |
| KB-431C1.5\|ENSG00000272037\|antisense | 0.55762812 | 1.86167167 | 1.73922345 | 6.14E-09 | 2.44E-08 | up |
| OSGEPL1-AS1\|ENSG00000253559\|antisense | 0.55537212 | 1.14394901 | 1.04249608 | 3.77E-09 | 1.55E-08 | up |
| MATN1-AS1\|ENSG00000186056\|antisense | 0.55442802 | 1.19662326 | 1.10989695 | 1.51E-12 | 1.08E-11 | up |
| RP11-181K3.4\|ENSG00000224468\|antisense | 0.55414813 | 1.12637051 | 1.02333789 | 0.00547634 | 0.00829194 | up |
| RHPN1-AS1\|ENSG00000254389\|antisense | 0.55304082 | 2.48359212 | 2.16697037 | 3.36E-16 | 4.44E-15 | up |
| GS1-124K5.3\|ENSG00000223473\|lincRNA | 0.54918632 | 1.28994469 | 1.23194162 | 2.55E-07 | 7.90E-07 | up |
| FTX\|ENSG00000230590\|lincRNA | 0.54851952 | 3.01123682 | 2.45674131 | 1.77E-07 | 5.64E-07 | up |
| RP11-486O12.2\|ENSG00000247373\|lincRNA | 0.54670813 | 1.10511411 | 1.01535261 | 7.49E-14 | 6.62E-13 | up |
| RP11-146F11.5\|ENSG00000260167\|antisense | 0.54619378 | 1.16965434 | 1.09859746 | 2.47E-06 | 6.53E-06 | up |
| C10orf91\|ENSG00000180066\|lincRNA | 0.54576726 | 1.37970962 | 1.33800692 | 2.03E-10 | 1.01E-09 | up |
| MCCC1-AS1\|ENSG00000243368\|antisense | 0.54575528 | 1.41784208 | 1.37737075 | 7.67E-09 | 2.99E-08 | up |
| AC007249.3\|ENSG00000217258\|lincRNA | 0.54382851 | 1.45994582 | 1.42469113 | 3.73E-09 | 1.54E-08 | up |
| CTD-2278I10.4\|ENSG00000269815\|sense_intronic | 0.54264408 | 1.28142072 | 1.23966607 | 4.43E-08 | 1.55E-07 | up |
| LINC01473\|ENSG00000237877\|lincRNA | 0.54218003 | 2.18214314 | 2.00890185 | 3.15E-11 | 1.77E-10 | up |
| RP11-230F18.6\|ENSG00000276916\|sense_intronic | 0.54205429 | 1.51389276 | 1.48175376 | 0.00071295 | 0.00126611 | up |
| LINC00843\|ENSG00000178440\|lincRNA | 0.54097325 | 1.21664073 | 1.16927405 | 0.0142924 | 0.02017146 | up |
| RP5-1159O4.2\|ENSG00000272732\|lincRNA | 0.53824383 | 1.14462301 | 1.08854073 | 9.08E-09 | 3.49E-08 | up |
| CTD-3222D19.8\|ENSG00000279529\|antisense | 0.53819636 | 1.08946844 | 1.01741986 | 5.15E-09 | 2.07E-08 | up |
| CTD-2349P21.12\|ENSG00000276250\|antisense | 0.537234 | 1.22078955 | 1.18419199 | 9.96E-06 | 2.39E-05 | up |
| RP3-510D11.2\|ENSG00000234546\|lincRNA | 0.53672491 | 1.65757509 | 1.62681947 | 1.20E-11 | 7.31E-11 | up |
| CTD-2517M22.14\|ENSG00000255182\|processed_transcript | 0.53322189 | 1.27378922 | 1.25631866 | 1.61E-09 | 6.99E-09 | up |
| RP11-50B3.4\|ENSG00000254694\|antisense | 0.53193712 | 1.25899791 | 1.24294827 | 0.00057907 | 0.00104623 | up |
| RP11-314A20.2\|ENSG00000244184\|antisense | 0.53142782 | 1.45933793 | 1.45736833 | 5.27E-09 | 2.12E-08 | up |
| RP11-560J1.2\|ENSG00000271888\|lincRNA | 0.53086512 | 1.271538 | 1.26015733 | 1.79E-09 | 7.74E-09 | up |
| HCG15\|ENSG00000227214\|antisense | 0.52762974 | 1.26321161 | 1.25949856 | 1.69E-10 | 8.46E-10 | up |
| RP1-122P22.4\|ENSG00000268628\|lincRNA | 0.52745083 | 2.8514498 | 2.43458712 | 0.01849048 | 0.02557881 | up |
| CTA-217C2.2\|ENSG00000273243\|lincRNA | 0.5268449 | 2.45951362 | 2.22292285 | 7.18E-13 | 5.44E-12 | up |
| RP6-74O6.6\|ENSG00000272824\|lincRNA | 0.52517474 | 1.8277978 | 1.79923706 | 1.86E-07 | 5.88E-07 | up |
| CYP4F35P\|ENSG00000265787\|lincRNA | 0.5248735 | 1.40981123 | 1.42546033 | 0.00363473 | 0.00568536 | up |
| RP11-415I12.3\|ENSG00000249753\|antisense | 0.52412958 | 1.09199769 | 1.05897438 | 0.00219336 | 0.00358747 | up |
| CTB-50L17.9\|ENSG00000267769\|antisense | 0.52120006 | 1.41138301 | 1.4372004 | 2.12E-08 | 7.74E-08 | up |
| RP11-505K9.5\|ENSG00000274677\|sense_intronic | 0.52101797 | 1.37195855 | 1.39683185 | 4.19E-09 | 1.71E-08 | up |
| NARF-IT1\|ENSG00000266236\|sense_intronic | 0.51962649 | 1.12059417 | 1.10871703 | 4.51E-05 | 9.78E-05 | up |
| RP11-817I4.1\|ENSG00000274964\|sense_intronic | 0.51836416 | 2.37172282 | 2.19389753 | 2.99E-17 | 4.79E-16 | up |
| RP11-54O7.17\|ENSG00000272512\|lincRNA | 0.51820576 | 1.36170698 | 1.39381933 | 7.29E-07 | 2.11E-06 | up |
| LINC01605\|ENSG00000253161\|lincRNA | 0.51695978 | 5.3065362 | 3.35964653 | 6.29E-23 | 4.33E-21 | up |
| RP11-734K2.4\|ENSG00000270344\|antisense | 0.51626007 | 1.41210871 | 1.45168123 | 3.29E-18 | 6.22E-17 | up |
| AC137932.4\|ENSG00000268218\|antisense | 0.51489267 | 1.36997135 | 1.41180208 | 6.29E-13 | 4.81E-12 | up |
| RP11-356B19.11\|ENSG00000271833\|sense_intronic | 0.51435934 | 1.90452772 | 1.88858478 | 1.06E-13 | 9.11E-13 | up |
| RP5-823G15.5\|ENSG00000259723\|antisense | 0.51418641 | 1.83402011 | 1.83464609 | 0.00050939 | 0.00092889 | up |
| AC129492.6\|ENSG00000214999\|antisense | 0.51185321 | 1.40310878 | 1.45482484 | 0.00463196 | 0.00709858 | up |
| ABALON\|ENSG00000281376\|antisense | 0.50998684 | 2.33143594 | 2.19268686 | 7.01E-23 | 4.71E-21 | up |
| RP11-122K13.7\|ENSG00000226699\|antisense | 0.50947153 | 1.5143065 | 1.5715838 | 0.00058602 | 0.00105699 | up |
| RP11-130L8.2\|ENSG00000269976\|lincRNA | 0.50859966 | 1.26465664 | 1.31414334 | 0.00044347 | 0.00082054 | up |
| LA16c-361A3.3\|ENSG00000261207\|antisense | 0.50824629 | 1.17769284 | 1.21236362 | 8.84E-05 | 0.00018386 | up |
| HIF1A-AS2\|ENSG00000258667\|lincRNA | 0.50812043 | 4.92045014 | 3.27554792 | 0.00088527 | 0.00154657 | up |
| RP11-968A15.2\|ENSG00000257596\|antisense | 0.50760872 | 2.40147308 | 2.24213089 | 1.75E-15 | 2.02E-14 | up |
| LLNLR-268E12.1\|ENSG00000276445\|lincRNA | 0.50577996 | 1.19169671 | 1.23643533 | 9.47E-06 | 2.28E-05 | up |
| RP11-506M12.1\|ENSG00000242798\|antisense | 0.50418028 | 1.46844442 | 1.54227707 | 1.06E-10 | 5.44E-10 | up |
| LINC01410\|ENSG00000238113\|lincRNA | 0.50316917 | 1.06668245 | 1.08401531 | 0.02820183 | 0.03786674 | up |
| AC009404.2\|ENSG00000236255\|lincRNA | 0.50284369 | 1.64767269 | 1.71224778 | 1.12E-18 | 2.38E-17 | up |
| CTB-31N19.3\|ENSG00000261596\|sense_intronic | 0.50212621 | 1.27053609 | 1.33931542 | 0.0026582 | 0.00427911 | up |
| AC005387.3\|ENSG00000268938\|antisense | 0.50198846 | 1.17513785 | 1.22710389 | 2.51E-07 | 7.80E-07 | up |
| RP11-785D18.3\|ENSG00000277247\|antisense | 0.50111336 | 4.26976035 | 3.09094619 | 1.95E-11 | 1.13E-10 | up |
| LRP4-AS1\|ENSG00000247675\|antisense | 0.49863534 | 1.43964376 | 1.52965482 | 0.00055919 | 0.00101391 | up |
| RP11-981P6.1\|ENSG00000258302\|antisense | 0.49654729 | 1.09537358 | 1.14141997 | 0.00305883 | 0.00485474 | up |
| RP11-211G23.2\|ENSG00000260877\|lincRNA | 0.49513291 | 7.75189821 | 3.96866189 | 2.72E-16 | 3.65E-15 | up |
| RP11-423P10.2\|ENSG00000272054\|sense_intronic | 0.49336897 | 1.05053787 | 1.09038929 | 2.88E-05 | 6.41E-05 | up |
| RP1-63M2.7\|ENSG00000275223\|lincRNA | 0.49068879 | 1.34788256 | 1.4578146 | 6.65E-10 | 3.03E-09 | up |
| RP11-727F15.13\|ENSG00000269463\|sense_intronic | 0.4900868 | 1.25145496 | 1.35249719 | 0.00034853 | 0.00065703 | up |
| RP11-138A9.2\|ENSG00000273319\|lincRNA | 0.48928352 | 3.07861354 | 2.65353819 | 2.09E-05 | 4.76E-05 | up |
| LINC01556\|ENSG00000204709\|lincRNA | 0.48665946 | 1.35263696 | 1.47479018 | 0.00317129 | 0.00501822 | up |
| RP11-64D22.5\|ENSG00000250271\|lincRNA | 0.48483775 | 1.0929808 | 1.17269413 | 0.00155519 | 0.00259819 | up |
| RP4-777L9.2\|ENSG00000273998\|lincRNA | 0.48457454 | 1.09132282 | 1.17128741 | 2.29E-05 | 5.18E-05 | up |
| UNC5B-AS1\|ENSG00000237512\|antisense | 0.48389497 | 2.43682272 | 2.33223547 | 0.00050012 | 0.0009145 | up |
| UBE2R2-AS1\|ENSG00000235481\|antisense | 0.48383609 | 1.59163602 | 1.71792015 | 1.16E-07 | 3.80E-07 | up |
| SNHG22\|ENSG00000267322\|antisense | 0.48312366 | 2.05673562 | 2.08989195 | 1.07E-08 | 4.10E-08 | up |
| RP11-387M24.5\|ENSG00000272170\|antisense | 0.48276219 | 1.36618866 | 1.50077213 | 5.66E-09 | 2.26E-08 | up |
| CTC-510F12.4\|ENSG00000267174\|3prime_overlapping_ncrna | 0.48242482 | 1.13426693 | 1.23338415 | 1.48E-10 | 7.50E-10 | up |
| AC010761.9\|ENSG00000265474\|antisense | 0.48110788 | 1.20716914 | 1.32719549 | 2.34E-07 | 7.31E-07 | up |
| RP11-269F19.2\|ENSG00000225721\|antisense | 0.48013333 | 1.09022726 | 1.1831219 | 6.80E-12 | 4.34E-11 | up |
| RP11-166B2.8\|ENSG00000263307\|antisense | 0.4799595 | 1.14564342 | 1.25517351 | 1.79E-05 | 4.12E-05 | up |
| AP000704.5\|ENSG00000224790\|lincRNA | 0.47981917 | 1.07146151 | 1.15901733 | 1.04E-08 | 3.97E-08 | up |
| CTD-3051D23.4\|ENSG00000258593\|lincRNA | 0.47962737 | 1.07645458 | 1.16630156 | 1.33E-05 | 3.13E-05 | up |
| LINC00525\|ENSG00000146666\|antisense | 0.4748033 | 1.886212 | 1.99008998 | 3.14E-09 | 1.31E-08 | up |
| RP13-714J12.1\|ENSG00000274373\|lincRNA | 0.47451031 | 1.50678679 | 1.66696396 | 3.17E-11 | 1.77E-10 | up |
| RP4-616B8.4\|ENSG00000261431\|antisense | 0.47401108 | 1.36467548 | 1.52556524 | 0.00217557 | 0.00356057 | up |
| RP1-244F24.1\|ENSG00000271857\|antisense | 0.47289866 | 0.97357439 | 1.04176015 | 0.00059974 | 0.0010799 | up |
| RP11-500G10.5\|ENSG00000277218\|lincRNA | 0.47255286 | 1.23808183 | 1.38955905 | 9.55E-08 | 3.19E-07 | up |
| CTD-2035E11.5\|ENSG00000272144\|lincRNA | 0.47241521 | 0.99691957 | 1.07742169 | 9.48E-05 | 0.00019616 | up |
| AC093585.6\|ENSG00000229996\|antisense | 0.46911335 | 1.60799681 | 1.77725608 | 0.00296989 | 0.00472491 | up |
| RP11-137H2.4\|ENSG00000226659\|antisense | 0.46830607 | 1.1206084 | 1.25875858 | 0.0006912 | 0.00123161 | up |
| RP5-1054A22.4\|ENSG00000277829\|sense_intronic | 0.46810723 | 1.12378938 | 1.26346073 | 0.01981057 | 0.02722734 | up |
| RP11-186B7.4\|ENSG00000264772\|processed_transcript | 0.46767243 | 0.9401589 | 1.00740622 | 2.30E-15 | 2.58E-14 | up |
| RP11-295D4.1\|ENSG00000262712\|sense_intronic | 0.46762083 | 1.51143519 | 1.69250803 | 1.03E-17 | 1.76E-16 | up |
| RP11-214F16.8\|ENSG00000280710\|lincRNA | 0.46713333 | 1.22641362 | 1.39253935 | 5.59E-10 | 2.58E-09 | up |
| RP3-337H4.9\|ENSG00000271754\|antisense | 0.46650613 | 1.67523735 | 1.84439757 | 1.28E-13 | 1.09E-12 | up |
| RP1-68D18.4\|ENSG00000255443\|antisense | 0.46616461 | 3.16362091 | 2.76266536 | 2.30E-14 | 2.17E-13 | up |
| RP11-336K24.12\|ENSG00000273002\|antisense | 0.46478309 | 0.99812988 | 1.10266996 | 6.72E-08 | 2.31E-07 | up |
| RP11-540A21.2\|ENSG00000245522\|lincRNA | 0.4647177 | 1.01533507 | 1.12752941 | 1.56E-07 | 5.04E-07 | up |
| AC000111.6\|ENSG00000083622\|antisense | 0.46457032 | 2.44875703 | 2.39808076 | 2.51E-05 | 5.65E-05 | up |
| RP11-295H24.5\|ENSG00000276593\|sense_intronic | 0.46362275 | 0.9428467 | 1.02407185 | 0.00307709 | 0.0048808 | up |
| RP3-323P24.3\|ENSG00000226310\|antisense | 0.46184041 | 1.14274223 | 1.30703369 | 5.69E-05 | 0.0001213 | up |
| RP11-646I6.6\|ENSG00000270147\|lincRNA | 0.46126912 | 0.96463706 | 1.06437753 | 0.00041788 | 0.00077616 | up |
| RP11-831H9.3\|ENSG00000254964\|antisense | 0.46124732 | 1.36570742 | 1.56603601 | 0.02473546 | 0.03361258 | up |
| RP11-93B14.6\|ENSG00000167046\|antisense | 0.46096136 | 1.37795903 | 1.57981527 | 7.90E-06 | 1.92E-05 | up |
| RP11-2C24.4\|ENSG00000260899\|processed_transcript | 0.45873642 | 1.24953847 | 1.44565798 | 2.96E-09 | 1.24E-08 | up |
| RP11-69E11.8\|ENSG00000228060\|antisense | 0.45846943 | 1.51942451 | 1.72862756 | 0.00037395 | 0.00069997 | up |
| LINC01273\|ENSG00000231742\|lincRNA | 0.45796074 | 1.5394464 | 1.74911581 | 3.15E-10 | 1.52E-09 | up |
| CTD-2382E5.6\|ENSG00000278493\|sense_intronic | 0.45775332 | 1.16263747 | 1.34475906 | 9.58E-07 | 2.70E-06 | up |
| AC005785.2\|ENSG00000268189\|processed_transcript | 0.45756079 | 1.26713124 | 1.46953063 | 8.09E-15 | 8.31E-14 | up |
| AC007773.2\|ENSG00000267213\|antisense | 0.45682161 | 1.73801631 | 1.92773882 | 4.65E-16 | 5.92E-15 | up |
| RP11-304F15.3\|ENSG00000204584\|antisense | 0.45682087 | 1.07938065 | 1.24050328 | 2.32E-07 | 7.26E-07 | up |
| AP000487.6\|ENSG00000254604\|antisense | 0.45624313 | 1.56611685 | 1.77931711 | 5.57E-12 | 3.63E-11 | up |
| RP11-552M11.4\|ENSG00000243960\|sense_overlapping | 0.45610532 | 1.23305152 | 1.43479419 | 5.21E-11 | 2.82E-10 | up |
| RP11-435O5.4\|ENSG00000271659\|antisense | 0.45606832 | 2.2348365 | 2.29284744 | 1.25E-12 | 9.09E-12 | up |
| CTC-537E7.2\|ENSG00000248359\|lincRNA | 0.45422438 | 2.48003471 | 2.44888328 | 0.00051876 | 0.000945 | up |
| RP3-325F22.3\|ENSG00000234263\|antisense | 0.453746 | 1.41897593 | 1.64489328 | 5.65E-05 | 0.00012051 | up |
| CTC-518P12.6\|ENSG00000267484\|antisense | 0.452255 | 1.00076044 | 1.14588833 | 0.00046436 | 0.00085501 | up |
| RP11-758P17.3\|ENSG00000240211\|antisense | 0.45221337 | 1.1104352 | 1.29604965 | 1.57E-06 | 4.30E-06 | up |
| RP3-522D1.1\|ENSG00000224167\|lincRNA | 0.45214983 | 3.51859612 | 2.96012709 | 1.96E-17 | 3.23E-16 | up |
| AC133644.2\|ENSG00000280721\|lincRNA | 0.4517709 | 1.24299971 | 1.4601627 | 9.70E-11 | 5.05E-10 | up |
| RP11-82L18.2\|ENSG00000224945\|sense_intronic | 0.45104031 | 3.10010956 | 2.78099092 | 1.51E-19 | 3.90E-18 | up |
| RP11-396C23.4\|ENSG00000272562\|antisense | 0.44803821 | 0.95121187 | 1.08614494 | 0.01891913 | 0.02613088 | up |
| AC027601.1\|ENSG00000260005\|antisense | 0.44792848 | 1.03964713 | 1.21475362 | 2.47E-10 | 1.20E-09 | up |
| UBE2Q1-AS1\|ENSG00000229780\|antisense | 0.44626568 | 1.24407759 | 1.47910171 | 5.49E-08 | 1.91E-07 | up |
| RP4-583P15.16\|ENSG00000274501\|antisense | 0.44495404 | 1.24635789 | 1.48599017 | 0.00103268 | 0.00178605 | up |
| BCYRN1\|ENSG00000236824\|lincRNA | 0.44354561 | 1.14377858 | 1.36665343 | 2.81E-05 | 6.27E-05 | up |
| RP11-629O1.2\|ENSG00000261220\|lincRNA | 0.44154957 | 2.35450313 | 2.41477532 | 2.65E-14 | 2.50E-13 | up |
| RP11-950C14.3\|ENSG00000258646\|antisense | 0.44127763 | 0.93513497 | 1.08348799 | 0.00012635 | 0.00025573 | up |
| RP11-33N14.5\|ENSG00000276292\|antisense | 0.44119264 | 0.99770276 | 1.17720135 | 0.01279776 | 0.01825153 | up |
| RP11-155O18.6\|ENSG00000272100\|antisense | 0.44077379 | 1.18943729 | 1.43216887 | 2.33E-08 | 8.46E-08 | up |
| RP11-864J10.4\|ENSG00000258136\|antisense | 0.43784051 | 0.96560769 | 1.14103172 | 0.00091369 | 0.00159324 | up |
| RP11-98F14.11\|ENSG00000269125\|antisense | 0.43751271 | 1.31427706 | 1.58687261 | 0.00010085 | 0.00020762 | up |
| RP11-732A21.3\|ENSG00000277459\|lincRNA | 0.43550088 | 1.37738503 | 1.66118435 | 1.59E-12 | 1.13E-11 | up |
| RP11-525G13.2\|ENSG00000236364\|antisense | 0.43493322 | 1.1728457 | 1.43114743 | 5.19E-07 | 1.53E-06 | up |
| MIR17HG\|ENSG00000215417\|processed_transcript | 0.43436584 | 3.91551342 | 3.17221895 | 6.87E-20 | 1.93E-18 | up |
| RP11-139H15.5\|ENSG00000276533\|sense_intronic | 0.43295602 | 1.47330579 | 1.76676452 | 3.53E-10 | 1.68E-09 | up |
| NEBL-AS1\|ENSG00000231920\|antisense | 0.43245721 | 0.98213596 | 1.18336536 | 2.75E-08 | 9.87E-08 | up |
| LINC00346\|ENSG00000255874\|lincRNA | 0.43116952 | 2.0880458 | 2.27582627 | 5.39E-19 | 1.23E-17 | up |
| RP13-317D12.3\|ENSG00000255237\|antisense | 0.43114576 | 1.05735708 | 1.29421508 | 2.38E-11 | 1.36E-10 | up |
| AC093375.1\|ENSG00000226383\|lincRNA | 0.43022547 | 1.7321779 | 2.00942226 | 0.00011887 | 0.00024198 | up |
| LINC00365\|ENSG00000224511\|lincRNA | 0.42563257 | 1.29053084 | 1.60028415 | 7.42E-09 | 2.90E-08 | up |
| RP11-706O15.3\|ENSG00000234449\|lincRNA | 0.42485776 | 1.31613488 | 1.63125552 | 0.00308412 | 0.0048905 | up |
| RP11-214K3.19\|ENSG00000270061\|sense_intronic | 0.42406851 | 1.4077657 | 1.73103798 | 9.43E-05 | 0.00019518 | up |
| RP11-981G7.6\|ENSG00000272505\|lincRNA | 0.42403839 | 1.02711475 | 1.27633059 | 0.018221 | 0.02523896 | up |
| RP11-420A23.1\|ENSG00000251432\|lincRNA | 0.42343455 | 1.22439336 | 1.53185622 | 4.88E-12 | 3.22E-11 | up |
| CTD-3214K23.1\|ENSG00000271555\|sense_intronic | 0.42150817 | 1.17772775 | 1.4823736 | 0.00015236 | 0.00030447 | up |
| RP11-128M1.1\|ENSG00000226644\|lincRNA | 0.41925028 | 2.77411978 | 2.72614643 | 4.01E-14 | 3.69E-13 | up |
| RP5-1142A6.10\|ENSG00000278341\|antisense | 0.41898109 | 0.90721027 | 1.11455184 | 1.72E-09 | 7.47E-09 | up |
| AC092301.3\|ENSG00000269148\|antisense | 0.41774514 | 0.98714061 | 1.24063256 | 2.79E-05 | 6.24E-05 | up |
| RP11-197P3.5\|ENSG00000229587\|sense_intronic | 0.41719547 | 1.11277549 | 1.41536715 | 7.38E-05 | 0.00015517 | up |
| RP11-254F7.4\|ENSG00000272524\|lincRNA | 0.41678251 | 1.05201826 | 1.3357931 | 4.21E-06 | 1.08E-05 | up |
| RP11-368I7.6\|ENSG00000275734\|antisense | 0.41586764 | 0.88006499 | 1.08148565 | 2.50E-07 | 7.77E-07 | up |
| RP11-84C13.2\|ENSG00000270720\|lincRNA | 0.41540921 | 1.36882996 | 1.72033815 | 6.88E-14 | 6.12E-13 | up |
| RP11-996F15.4\|ENSG00000275476\|antisense | 0.41392187 | 1.19592625 | 1.53069805 | 0.0326435 | 0.04323756 | up |
| RP11-773H22.4\|ENSG00000266969\|antisense | 0.41346726 | 0.85519076 | 1.04847316 | 0.0051003 | 0.00776247 | up |
| RP11-127B20.3\|ENSG00000272677\|antisense | 0.41019181 | 1.00485199 | 1.29261241 | 5.00E-12 | 3.29E-11 | up |
| ZNF337-AS1\|ENSG00000213742\|antisense | 0.41006176 | 1.09263208 | 1.41389458 | 7.75E-13 | 5.84E-12 | up |
| AP006621.8\|ENSG00000255108\|antisense | 0.40834518 | 1.3478277 | 1.72277497 | 7.89E-05 | 0.00016532 | up |
| RP5-1112D6.7\|ENSG00000271789\|lincRNA | 0.40736815 | 1.54189094 | 1.92029563 | 2.18E-12 | 1.51E-11 | up |
| RP4-785G19.5\|ENSG00000250917\|sense_overlapping | 0.4063969 | 2.17417402 | 2.41950612 | 4.40E-16 | 5.64E-15 | up |
| RP11-394I13.2\|ENSG00000271151\|lincRNA | 0.40633392 | 1.44851143 | 1.83383337 | 3.25E-12 | 2.19E-11 | up |
| RP11-568A7.2\|ENSG00000228648\|lincRNA | 0.4062801 | 2.21638601 | 2.44766257 | 2.48E-08 | 8.95E-08 | up |
| RP11-1055B8.9\|ENSG00000275966\|lincRNA | 0.40543966 | 2.83789954 | 2.80726438 | 3.15E-11 | 1.77E-10 | up |
| RP11-77P6.2\|ENSG00000227896\|antisense | 0.40466456 | 0.99308935 | 1.29519701 | 1.38E-13 | 1.17E-12 | up |
| RP11-533E19.7\|ENSG00000272906\|lincRNA | 0.40425662 | 1.1505291 | 1.50895417 | 1.48E-11 | 8.80E-11 | up |
| CTD-2540F13.2\|ENSG00000267892\|antisense | 0.40297755 | 2.1189492 | 2.39457762 | 5.48E-15 | 5.79E-14 | up |
| RP11-626H12.2\|ENSG00000254605\|lincRNA | 0.40159497 | 1.37227286 | 1.77275428 | 6.84E-05 | 0.00014414 | up |
| RP11-214K3.21\|ENSG00000269997\|sense_intronic | 0.40145529 | 1.26697413 | 1.65807584 | 0.00038878 | 0.00072593 | up |
| AL133243.2\|ENSG00000276517\|sense_intronic | 0.40033349 | 1.12534239 | 1.4910898 | 2.04E-07 | 6.43E-07 | up |
| KCNIP2-AS1\|ENSG00000226009\|antisense | 0.39913094 | 0.98388818 | 1.30163225 | 3.66E-12 | 2.45E-11 | up |
| AC004471.9\|ENSG00000223461\|antisense | 0.39843404 | 1.29867287 | 1.70462528 | 3.76E-13 | 2.96E-12 | up |
| RP11-531A24.5\|ENSG00000253636\|antisense | 0.39806646 | 0.81360589 | 1.03132082 | 0.00112745 | 0.00192918 | up |
| UCKL1-AS1\|ENSG00000280213\|antisense | 0.39783067 | 1.38137371 | 1.79587727 | 4.77E-11 | 2.60E-10 | up |
| HS1BP3-IT1\|ENSG00000231948\|sense_intronic | 0.3968463 | 0.95392992 | 1.26530295 | 9.25E-05 | 0.00019145 | up |
| RP3-453C12.14\|ENSG00000275894\|sense_intronic | 0.39550142 | 2.33178693 | 2.55968119 | 5.05E-13 | 3.92E-12 | up |
| GCC2-AS1\|ENSG00000214184\|antisense | 0.39449985 | 0.96311347 | 1.28768104 | 3.63E-12 | 2.43E-11 | up |
| RP11-57H14.2\|ENSG00000233547\|antisense | 0.39385014 | 0.8484601 | 1.10720004 | 0.00390344 | 0.00607164 | up |
| RP5-963E22.6\|ENSG00000273821\|lincRNA | 0.39228961 | 1.59695866 | 2.02533595 | 3.17E-13 | 2.53E-12 | up |
| RP11-1029J19.5\|ENSG00000258404\|lincRNA | 0.39218316 | 3.86241866 | 3.29990506 | 3.36E-20 | 1.07E-18 | up |
| RP11-142E9.1\|ENSG00000276672\|sense_intronic | 0.38929795 | 1.63652286 | 2.07168711 | 1.15E-17 | 1.96E-16 | up |
| RP11-626G11.5\|ENSG00000261465\|antisense | 0.38921288 | 0.86302627 | 1.14884502 | 3.24E-07 | 9.83E-07 | up |
| RP11-388C12.5\|ENSG00000263321\|lincRNA | 0.38915029 | 1.10354227 | 1.50374257 | 3.74E-07 | 1.12E-06 | up |
| ZNF346-IT1\|ENSG00000251666\|sense_intronic | 0.38808954 | 1.02761832 | 1.40484306 | 7.67E-06 | 1.87E-05 | up |
| RP11-22L13.1\|ENSG00000272235\|lincRNA | 0.38573961 | 1.47730233 | 1.93726591 | 3.75E-09 | 1.54E-08 | up |
| RP11-96D1.5\|ENSG00000260891\|antisense | 0.38569921 | 1.43259744 | 1.89308519 | 0.00017123 | 0.00033958 | up |
| MIR181A2HG\|ENSG00000224020\|antisense | 0.38554504 | 1.80978278 | 2.23084525 | 1.27E-09 | 5.58E-09 | up |
| RP11-627K11.6\|ENSG00000275097\|3prime_overlapping_ncrna | 0.38445077 | 1.28169565 | 1.73718297 | 4.52E-09 | 1.84E-08 | up |
| DCUN1D2-AS\|ENSG00000233613\|antisense | 0.38366659 | 1.00355793 | 1.38719887 | 0.00035884 | 0.00067406 | up |
| RP11-438L19.1\|ENSG00000272800\|lincRNA | 0.38329427 | 1.29033446 | 1.75122073 | 1.61E-14 | 1.57E-13 | up |
| AC005618.6\|ENSG00000272070\|lincRNA | 0.38250266 | 0.91015109 | 1.25063628 | 0.00018804 | 0.00037139 | up |
| IBA57-AS1\|ENSG00000203684\|lincRNA | 0.38173905 | 0.92768023 | 1.28104083 | 3.40E-06 | 8.81E-06 | up |
| CMB9-94B1.2\|ENSG00000276505\|sense_intronic | 0.37857201 | 0.76051853 | 1.00641567 | 0.01451537 | 0.02044804 | up |
| RP11-7I15.4\|ENSG00000254675\|antisense | 0.37843846 | 1.4280104 | 1.91587588 | 8.95E-09 | 3.44E-08 | up |
| AC020594.5\|ENSG00000237133\|antisense | 0.37840986 | 0.77668066 | 1.03737186 | 0.00474285 | 0.00725384 | up |
| RP11-932O9.10\|ENSG00000269974\|lincRNA | 0.37733467 | 2.02045424 | 2.42076311 | 1.18E-11 | 7.22E-11 | up |
| RP11-395N3.2\|ENSG00000272622\|lincRNA | 0.37731358 | 0.8454729 | 1.16399449 | 0.00090286 | 0.00157591 | up |
| CTD-2192J16.21\|ENSG00000269560\|sense_intronic | 0.37489874 | 1.02942223 | 1.45726195 | 0.00066299 | 0.00118573 | up |
| CTA-126B4.7\|ENSG00000230107\|lincRNA | 0.37371044 | 2.8840927 | 2.94812478 | 2.14E-11 | 1.23E-10 | up |
| RP11-982M15.6\|ENSG00000256050\|lincRNA | 0.3716645 | 0.84458883 | 1.18424826 | 1.01E-07 | 3.37E-07 | up |
| RP11-431J24.2\|ENSG00000238178\|antisense | 0.37165321 | 10.8782088 | 4.87134015 | 9.22E-06 | 2.22E-05 | up |
| RP11-21M24.3\|ENSG00000260635\|antisense | 0.37133186 | 1.58643572 | 2.09500805 | 0.00698615 | 0.01040854 | up |
| RP11-23F23.2\|ENSG00000254480\|lincRNA | 0.37021313 | 0.96027197 | 1.375087 | 2.49E-05 | 5.61E-05 | up |
| RP11-383C5.5\|ENSG00000234134\|sense_intronic | 0.36951945 | 0.81127026 | 1.13453232 | 0.00122817 | 0.00208804 | up |
| RP4-584D14.6\|ENSG00000239377\|antisense | 0.36845224 | 1.1915792 | 1.69332533 | 1.63E-06 | 4.45E-06 | up |
| RP11-151N17.1\|ENSG00000259659\|sense_intronic | 0.36833031 | 1.36200815 | 1.88666333 | 1.18E-08 | 4.47E-08 | up |
| CASC19\|ENSG00000254166\|lincRNA | 0.36777945 | 12.2730608 | 5.0605104 | 5.04E-25 | 8.55E-23 | up |
| RP11-461A8.4\|ENSG00000263235\|sense_overlapping | 0.36393688 | 1.38528655 | 1.9284243 | 1.44E-16 | 2.04E-15 | up |
| AC087294.2\|ENSG00000235530\|antisense | 0.36357078 | 0.78117065 | 1.10340152 | 0.0270378 | 0.03644226 | up |
| LINC00628\|ENSG00000280924\|lincRNA | 0.36322856 | 0.9621688 | 1.40541238 | 4.31E-12 | 2.86E-11 | up |
| RP11-353N14.4\|ENSG00000262188\|lincRNA | 0.36281229 | 5.02250303 | 3.79111129 | 1.22E-20 | 4.46E-19 | up |
| CTD-2199O4.6\|ENSG00000272417\|lincRNA | 0.361549 | 0.96625572 | 1.41821388 | 0.00069514 | 0.0012378 | up |
| ERICD\|ENSG00000280303\|lincRNA | 0.36143369 | 0.939316 | 1.3778796 | 8.78E-14 | 7.68E-13 | up |
| AC097721.2\|ENSG00000228043\|lincRNA | 0.36121856 | 0.9263435 | 1.35867525 | 1.18E-09 | 5.20E-09 | up |
| RP11-552F3.10\|ENSG00000267342\|antisense | 0.36113592 | 0.95521954 | 1.40329043 | 7.01E-14 | 6.20E-13 | up |
| AC073333.8\|ENSG00000235837\|antisense | 0.36068396 | 1.41255691 | 1.96950183 | 1.95E-12 | 1.37E-11 | up |
| RP11-563N4.1\|ENSG00000272716\|lincRNA | 0.3593271 | 0.73427723 | 1.03102713 | 2.08E-06 | 5.54E-06 | up |
| RP11-723O4.2\|ENSG00000231305\|antisense | 0.35777519 | 0.77302272 | 1.11145747 | 2.31E-06 | 6.13E-06 | up |
| RP4-673M15.1\|ENSG00000272768\|antisense | 0.3571894 | 0.85631136 | 1.26144619 | 5.56E-06 | 1.39E-05 | up |
| LINC01012\|ENSG00000281706\|lincRNA | 0.35618484 | 0.98053923 | 1.46094924 | 3.41E-17 | 5.42E-16 | up |
| AC005387.2\|ENSG00000269191\|antisense | 0.35557822 | 0.93370038 | 1.3927927 | 3.75E-08 | 1.33E-07 | up |
| AC004837.5\|ENSG00000228554\|antisense | 0.35532555 | 1.97799059 | 2.47682223 | 2.64E-09 | 1.11E-08 | up |
| CTA-276F8.1\|ENSG00000276564\|lincRNA | 0.35403456 | 0.9942891 | 1.48977518 | 5.73E-08 | 1.99E-07 | up |
| AC092614.2\|ENSG00000227542\|lincRNA | 0.35322373 | 1.40161093 | 1.98843177 | 4.52E-17 | 7.00E-16 | up |
| RP11-723O4.9\|ENSG00000261159\|lincRNA | 0.35251767 | 1.31739854 | 1.90192437 | 0.00016114 | 0.00032112 | up |
| CTC-497E21.3\|ENSG00000254670\|lincRNA | 0.35210839 | 1.25163419 | 1.82972149 | 2.15E-06 | 5.73E-06 | up |
| RP11-485G7.5\|ENSG00000263080\|antisense | 0.35044159 | 0.76644128 | 1.12900124 | 1.03E-07 | 3.41E-07 | up |
| RP1-179N16.6\|ENSG00000246982\|antisense | 0.34996581 | 1.17840212 | 1.75154604 | 2.48E-12 | 1.71E-11 | up |
| RP11-67L3.4\|ENSG00000233203\|antisense | 0.34936257 | 0.72970039 | 1.06257917 | 7.93E-08 | 2.69E-07 | up |
| RP11-167N5.5\|ENSG00000267834\|lincRNA | 0.3491334 | 0.89413656 | 1.35671681 | 2.92E-07 | 8.93E-07 | up |
| MED4-AS1\|ENSG00000229111\|antisense | 0.34910452 | 0.96579522 | 1.46805828 | 3.03E-07 | 9.24E-07 | up |
| AC012314.8\|ENSG00000237017\|antisense | 0.34877845 | 0.72902255 | 1.06365255 | 1.35E-11 | 8.14E-11 | up |
| RP1-68D18.2\|ENSG00000251194\|sense_intronic | 0.34807421 | 1.91650252 | 2.46100909 | 1.07E-13 | 9.19E-13 | up |
| CTD-2302E22.6\|ENSG00000274015\|lincRNA | 0.34803819 | 1.04482511 | 1.58594397 | 1.50E-11 | 8.88E-11 | up |
| RNF139-AS1\|ENSG00000245149\|lincRNA | 0.34781047 | 1.04788507 | 1.59110722 | 4.81E-15 | 5.16E-14 | up |
| AP000487.5\|ENSG00000246889\|antisense | 0.34777697 | 0.91207594 | 1.39099157 | 5.29E-17 | 8.09E-16 | up |
| LINC01183\|ENSG00000230561\|lincRNA | 0.34719877 | 1.40301636 | 2.01469811 | 7.80E-18 | 1.37E-16 | up |
| RP11-497H17.1\|ENSG00000262663\|lincRNA | 0.34660325 | 1.52318264 | 2.13573184 | 8.22E-08 | 2.78E-07 | up |
| AP001412.1\|ENSG00000272948\|antisense | 0.34644653 | 0.87560541 | 1.33764818 | 4.44E-08 | 1.55E-07 | up |
| LINC01126\|ENSG00000279873\|lincRNA | 0.34376369 | 0.71811074 | 1.06278916 | 8.62E-05 | 0.00017967 | up |
| RP3-404F18.5\|ENSG00000237903\|sense_intronic | 0.34285269 | 0.92300407 | 1.42874818 | 0.02028898 | 0.02784149 | up |
| RP11-492E3.2\|ENSG00000227619\|antisense | 0.34207725 | 3.51439448 | 3.36088208 | 9.88E-20 | 2.65E-18 | up |
| AF064858.10\|ENSG00000237609\|lincRNA | 0.34176634 | 1.13347735 | 1.72967336 | 0.02474492 | 0.03361682 | up |
| RP11-504P24.9\|ENSG00000278467\|lincRNA | 0.3413999 | 1.142331 | 1.74244621 | 0.00130711 | 0.00220951 | up |
| RP11-506H20.1\|ENSG00000251307\|antisense | 0.34101607 | 1.85842013 | 2.44616505 | 3.89E-19 | 9.12E-18 | up |
| RP11-298I3.4\|ENSG00000258457\|antisense | 0.34043384 | 0.83549924 | 1.29526407 | 3.49E-10 | 1.67E-09 | up |
| LINC00471\|ENSG00000181798\|lincRNA | 0.34013484 | 0.87871442 | 1.36928757 | 3.10E-08 | 1.11E-07 | up |
| LINC00894\|ENSG00000235703\|antisense | 0.33979812 | 1.43600179 | 2.07930777 | 1.01E-12 | 7.41E-12 | up |
| RP11-972P1.11\|ENSG00000278112\|sense_intronic | 0.33902088 | 1.15207386 | 1.76478717 | 3.17E-10 | 1.52E-09 | up |
| AC142528.1\|ENSG00000235078\|antisense | 0.33859001 | 0.75214058 | 1.15146296 | 6.91E-09 | 2.71E-08 | up |
| CTD-2116N20.1\|ENSG00000250081\|lincRNA | 0.33802097 | 1.44645522 | 2.09733699 | 6.98E-10 | 3.18E-09 | up |
| U47924.29\|ENSG00000271969\|antisense | 0.33687925 | 1.1540466 | 1.77639798 | 4.88E-10 | 2.28E-09 | up |
| BACH1-IT1\|ENSG00000248476\|sense_intronic | 0.33494876 | 0.93442187 | 1.48013362 | 0.01317183 | 0.0187485 | up |
| RP11-264B17.4\|ENSG00000260367\|antisense | 0.33449949 | 0.81354574 | 1.28221944 | 1.53E-10 | 7.70E-10 | up |
| RP11-214K3.20\|ENSG00000269938\|sense_intronic | 0.334002 | 1.0429636 | 1.64276015 | 0.01545595 | 0.02166691 | up |
| LA16c-325D7.1\|ENSG00000263325\|antisense | 0.33338588 | 3.79344387 | 3.5082433 | 2.03E-18 | 4.05E-17 | up |
| AF064858.6\|ENSG00000205622\|lincRNA | 0.33313195 | 1.61945003 | 2.28133834 | 2.95E-09 | 1.23E-08 | up |
| CTB-31O20.8\|ENSG00000267141\|antisense | 0.33263539 | 0.97507177 | 1.55156673 | 1.88E-05 | 4.31E-05 | up |
| RP11-383J24.1\|ENSG00000253227\|lincRNA | 0.33249797 | 0.78053693 | 1.23112138 | 0.01974991 | 0.02715805 | up |
| RP6-65G23.5\|ENSG00000275630\|lincRNA | 0.33203424 | 0.76387083 | 1.20199668 | 0.00021556 | 0.00042211 | up |
| RP11-214O1.2\|ENSG00000266709\|lincRNA | 0.33026279 | 0.77612357 | 1.23267193 | 0.00130909 | 0.00221213 | up |
| AC107081.5\|ENSG00000236498\|antisense | 0.33006301 | 1.25468109 | 1.92650733 | 2.96E-15 | 3.28E-14 | up |
| CTD-2023M8.1\|ENSG00000248693\|lincRNA | 0.33003357 | 1.31169266 | 1.99074503 | 6.89E-07 | 2.00E-06 | up |
| CTB-61M7.2\|ENSG00000268734\|lincRNA | 0.32929898 | 1.35437359 | 2.04015579 | 0.00110042 | 0.00188964 | up |
| ZDHHC20-IT1\|ENSG00000236953\|sense_intronic | 0.32915207 | 1.6121549 | 2.29216418 | 2.70E-11 | 1.53E-10 | up |
| RP11-546J1.1\|ENSG00000270557\|lincRNA | 0.32894475 | 0.91395498 | 1.47427781 | 1.00E-10 | 5.20E-10 | up |
| GS1-293C5.1\|ENSG00000273138\|antisense | 0.32855561 | 0.85238857 | 1.37537367 | 0.02186609 | 0.02988967 | up |
| LINC00896\|ENSG00000236499\|lincRNA | 0.32763222 | 1.62670892 | 2.311807 | 7.11E-15 | 7.39E-14 | up |
| CTD-2047H16.2\|ENSG00000262979\|sense_intronic | 0.32753052 | 4.91485725 | 3.90744825 | 3.92E-19 | 9.17E-18 | up |
| DDX11-AS1\|ENSG00000245614\|antisense | 0.32748313 | 1.14679681 | 1.80811731 | 7.60E-20 | 2.11E-18 | up |
| RP11-108K3.1\|ENSG00000259240\|lincRNA | 0.32719207 | 1.53740047 | 2.23228332 | 1.28E-13 | 1.09E-12 | up |
| LINC00622\|ENSG00000260941\|sense_overlapping | 0.32642649 | 0.83852171 | 1.36109001 | 0.00024723 | 0.000479 | up |
| AC005624.2\|ENSG00000267201\|lincRNA | 0.32591657 | 0.75857939 | 1.21879746 | 0.00011614 | 0.00023697 | up |
| RP11-972P1.10\|ENSG00000274427\|sense_intronic | 0.32572068 | 1.71839238 | 2.39935229 | 3.12E-14 | 2.92E-13 | up |
| RP11-227H15.5\|ENSG00000231748\|antisense | 0.32569705 | 1.63785272 | 2.33020307 | 8.70E-12 | 5.45E-11 | up |
| RP1-27K12.4\|ENSG00000249379\|antisense | 0.32523979 | 0.76332245 | 1.23078887 | 0.00508159 | 0.00773622 | up |
| SLC16A1-AS1\|ENSG00000226419\|antisense | 0.32475253 | 1.34976279 | 2.05529321 | 2.10E-18 | 4.16E-17 | up |
| JARID2-AS1\|ENSG00000235488\|antisense | 0.32470506 | 0.99668867 | 1.61801307 | 2.06E-05 | 4.70E-05 | up |
| C5orf66\|ENSG00000224186\|antisense | 0.32442324 | 0.91830779 | 1.50110061 | 3.27E-20 | 1.05E-18 | up |
| RP11-478C19.2\|ENSG00000258210\|processed_transcript | 0.324279 | 0.85194151 | 1.39351879 | 4.21E-08 | 1.48E-07 | up |
| RP11-44N11.2\|ENSG00000272384\|lincRNA | 0.32390778 | 0.68857438 | 1.08802937 | 1.06E-06 | 2.97E-06 | up |
| SOX9-AS1\|ENSG00000234899\|processed_transcript | 0.32366441 | 0.7089475 | 1.13118005 | 3.85E-07 | 1.16E-06 | up |
| RP11-141M1.4\|ENSG00000277386\|sense_intronic | 0.32365439 | 1.02763074 | 1.66679599 | 0.00012419 | 0.00025147 | up |
| RP11-426A6.5\|ENSG00000226706\|antisense | 0.32318942 | 0.94230265 | 1.54381053 | 1.32E-12 | 9.53E-12 | up |
| RP11-596D21.1\|ENSG00000257831\|antisense | 0.32206272 | 2.0071767 | 2.63975406 | 4.49E-05 | 9.75E-05 | up |
| RP11-7F18.2\|ENSG00000271966\|lincRNA | 0.32161185 | 0.69604726 | 1.11386468 | 0.00014726 | 0.00029524 | up |
| RP11-128N14.5\|ENSG00000276573\|sense_intronic | 0.32124724 | 2.192552 | 2.77085508 | 1.69E-08 | 6.25E-08 | up |
| LINC01186\|ENSG00000236751\|lincRNA | 0.32123993 | 1.68175325 | 2.38824292 | 8.64E-17 | 1.27E-15 | up |
| RP11-89K10.1\|ENSG00000254286\|antisense | 0.31942423 | 0.89381376 | 1.48450051 | 1.92E-11 | 1.11E-10 | up |
| AC005306.3\|ENSG00000267283\|processed_transcript | 0.31926787 | 0.6784751 | 1.0875285 | 3.52E-06 | 9.09E-06 | up |
| RP11-794G24.1\|ENSG00000256443\|lincRNA | 0.31836589 | 1.11822007 | 1.81244648 | 3.46E-09 | 1.43E-08 | up |
| YEATS2-AS1\|ENSG00000233885\|antisense | 0.31822275 | 0.89382894 | 1.48996178 | 1.92E-12 | 1.35E-11 | up |
| RP11-1260E13.1\|ENSG00000262920\|antisense | 0.31790104 | 0.70318957 | 1.14533593 | 0.02198463 | 0.03004396 | up |
| RP13-349O20.2\|ENSG00000281091\|lincRNA | 0.31706484 | 1.09513052 | 1.78825301 | 5.76E-07 | 1.69E-06 | up |
| RP5-1157M23.2\|ENSG00000243224\|antisense | 0.31645191 | 0.8283582 | 1.38826848 | 2.75E-11 | 1.56E-10 | up |
| CH17-140K24.6\|ENSG00000279375\|antisense | 0.3155654 | 1.38897038 | 2.1380049 | 0.00356604 | 0.00559112 | up |
| RP11-35O15.1\|ENSG00000259424\|antisense | 0.3146028 | 0.78339169 | 1.31620232 | 0.00020556 | 0.00040403 | up |
| RP11-521I2.3\|ENSG00000260368\|sense_overlapping | 0.31422855 | 0.85343011 | 1.44145874 | 9.40E-10 | 4.21E-09 | up |
| AC007038.7\|ENSG00000229127\|antisense | 0.31339263 | 1.09992048 | 1.81135605 | 1.66E-07 | 5.32E-07 | up |
| LINC00431\|ENSG00000225760\|lincRNA | 0.31147582 | 0.81863557 | 1.39410118 | 0.00193017 | 0.00318252 | up |
| CTC-215O4.4\|ENSG00000266936\|antisense | 0.31145784 | 0.79228333 | 1.34697954 | 0.02871287 | 0.03849442 | up |
| RP1-191J18.66\|ENSG00000272476\|antisense | 0.31115702 | 0.79908273 | 1.36070209 | 1.49E-16 | 2.10E-15 | up |
| RP4-697K14.15\|ENSG00000275812\|antisense | 0.31076058 | 1.71069102 | 2.46070378 | 2.48E-10 | 1.21E-09 | up |
| AC127904.2\|ENSG00000230732\|sense_intronic | 0.3088378 | 1.2343935 | 1.99888112 | 0.00042246 | 0.00078431 | up |
| AF230666.2\|ENSG00000223697\|antisense | 0.30856969 | 0.65392568 | 1.08353031 | 0.00821346 | 0.01208761 | up |
| KB-1836B5.4\|ENSG00000272128\|lincRNA | 0.30813742 | 0.6512491 | 1.07963557 | 0.0090811 | 0.01327603 | up |
| AC010976.2\|ENSG00000231731\|antisense | 0.30788964 | 0.8747403 | 1.50644143 | 4.13E-08 | 1.45E-07 | up |
| RP11-768F21.1\|ENSG00000248636\|lincRNA | 0.30729928 | 0.65317186 | 1.08781823 | 3.63E-08 | 1.29E-07 | up |
| SLFNL1-AS1\|ENSG00000281207\|antisense | 0.30686248 | 0.64910365 | 1.08085664 | 2.35E-07 | 7.33E-07 | up |
| RP11-29B2.6\|ENSG00000276957\|lincRNA | 0.30579716 | 1.17612209 | 1.94339094 | 1.76E-07 | 5.61E-07 | up |
| RP11-806H10.4\|ENSG00000266970\|lincRNA | 0.30552727 | 0.66676439 | 1.12587592 | 2.93E-07 | 8.96E-07 | up |
| RP11-188P17.2\|ENSG00000272646\|lincRNA | 0.30446233 | 0.65371457 | 1.1023971 | 0.00025341 | 0.00049008 | up |
| CTC-537E7.3\|ENSG00000248884\|lincRNA | 0.30421388 | 0.95082797 | 1.64409835 | 0.03021338 | 0.04033535 | up |
| FALEC\|ENSG00000228126\|lincRNA | 0.30188627 | 0.87826595 | 1.54065274 | 2.96E-07 | 9.05E-07 | up |
| TGFB2-AS1\|ENSG00000232480\|antisense | 0.30140458 | 0.786976 | 1.38461829 | 0.03734435 | 0.0490006 | up |
| RP11-167P11.2\|ENSG00000270165\|sense_intronic | 0.30101544 | 0.61326084 | 1.02666336 | 0.00615491 | 0.0092402 | up |
| TM4SF1-AS1\|ENSG00000240541\|antisense | 0.3001614 | 1.19666835 | 1.99521299 | 6.21E-12 | 4.01E-11 | up |
| RP11-150O12.5\|ENSG00000254111\|lincRNA | 0.30011463 | 1.26434837 | 2.07480849 | 1.28E-07 | 4.18E-07 | up |
| RP11-303E16.5\|ENSG00000261141\|antisense | 0.29859869 | 0.83769366 | 1.48821494 | 6.72E-10 | 3.06E-09 | up |
| RP11-214K3.22\|ENSG00000270048\|sense_intronic | 0.29826211 | 1.08316759 | 1.86060387 | 0.00270736 | 0.004349 | up |
| OGFR-AS1\|ENSG00000229873\|antisense | 0.29678321 | 0.7128634 | 1.26421615 | 1.07E-05 | 2.55E-05 | up |
| C3orf35\|ENSG00000198590\|lincRNA | 0.29531141 | 0.7328876 | 1.31135487 | 2.07E-11 | 1.19E-10 | up |
| RP11-173B14.4\|ENSG00000228444\|sense_intronic | 0.29401089 | 1.32834601 | 2.17568947 | 8.16E-12 | 5.13E-11 | up |
| RP11-458D21.1\|ENSG00000233396\|lincRNA | 0.29384988 | 0.71905961 | 1.29103207 | 1.31E-07 | 4.30E-07 | up |
| RP11-800A3.2\|ENSG00000215841\|antisense | 0.29317695 | 0.8212928 | 1.48612496 | 0.00114585 | 0.00195687 | up |
| CTC-425O23.2\|ENSG00000205041\|sense_intronic | 0.29295473 | 0.7013398 | 1.25943588 | 0.00335059 | 0.00528458 | up |
| C9orf173-AS1\|ENSG00000275549\|antisense | 0.29272468 | 0.7469049 | 1.35138017 | 2.77E-05 | 6.19E-05 | up |
| RP11-488P3.1\|ENSG00000230439\|sense_intronic | 0.29256036 | 0.60613632 | 1.05090798 | 0.00012014 | 0.0002443 | up |
| CASC15\|ENSG00000272168\|lincRNA | 0.29163921 | 1.14859193 | 1.97760975 | 1.23E-11 | 7.49E-11 | up |
| LINC01376\|ENSG00000236204\|lincRNA | 0.29149869 | 0.72493441 | 1.3143611 | 8.87E-07 | 2.52E-06 | up |
| CTD-3065B20.2\|ENSG00000259212\|antisense | 0.29126904 | 0.88035017 | 1.59572512 | 0.0334272 | 0.04421097 | up |
| RP4-760C5.3\|ENSG00000231081\|lincRNA | 0.29109261 | 2.00498427 | 2.78404081 | 1.13E-11 | 6.94E-11 | up |
| AC010761.14\|ENSG00000267729\|antisense | 0.29093913 | 0.61228853 | 1.07349431 | 0.01073605 | 0.01549886 | up |
| CTD-3216D2.5\|ENSG00000272945\|sense_intronic | 0.29055626 | 2.33081927 | 3.0039477 | 8.15E-20 | 2.24E-18 | up |
| LINC00173\|ENSG00000196668\|processed_transcript | 0.29047052 | 0.77049163 | 1.40738752 | 0.00052818 | 0.00096149 | up |
| RP11-17G12.2\|ENSG00000256897\|antisense | 0.29017652 | 1.0055027 | 1.79291426 | 0.00035683 | 0.00067076 | up |
| RP11-350J20.5\|ENSG00000231760\|antisense | 0.28969545 | 0.81310135 | 1.48889818 | 6.72E-08 | 2.31E-07 | up |
| AC092171.2\|ENSG00000188365\|antisense | 0.28965154 | 1.27761385 | 2.14106164 | 2.64E-13 | 2.13E-12 | up |
| AC005540.3\|ENSG00000235852\|antisense | 0.28924481 | 0.83483828 | 1.52920567 | 5.74E-05 | 0.00012224 | up |
| RP11-661A12.9\|ENSG00000255050\|antisense | 0.28771284 | 1.5326238 | 2.41330211 | 3.24E-12 | 2.19E-11 | up |
| RP1-28H20.3\|ENSG00000271784\|lincRNA | 0.28674944 | 0.61822477 | 1.10834078 | 5.27E-05 | 0.00011304 | up |
| RP11-676J12.8\|ENSG00000262434\|antisense | 0.28673811 | 0.58890056 | 1.03829037 | 0.00773838 | 0.01142701 | up |
| KLHL7-AS1\|ENSG00000230658\|lincRNA | 0.28600797 | 0.64318529 | 1.1691791 | 0.00067447 | 0.00120464 | up |
| RP11-166B2.5\|ENSG00000261216\|antisense | 0.28574694 | 0.99103882 | 1.79420353 | 4.31E-06 | 1.10E-05 | up |
| RP4-597N16.4\|ENSG00000273416\|lincRNA | 0.2856079 | 0.83205078 | 1.54263571 | 7.93E-09 | 3.08E-08 | up |
| AC006273.5\|ENSG00000267530\|lincRNA | 0.28525769 | 2.09796373 | 2.87865205 | 3.04E-18 | 5.82E-17 | up |
| RP11-399K21.14\|ENSG00000272692\|lincRNA | 0.28523843 | 0.65640418 | 1.20241604 | 3.31E-05 | 7.31E-05 | up |
| RP11-152P23.2\|ENSG00000260350\|antisense | 0.28515476 | 0.73744868 | 1.37079756 | 1.49E-06 | 4.10E-06 | up |
| RP11-74C1.4\|ENSG00000232536\|sense_intronic | 0.28488918 | 0.91373457 | 1.6813743 | 7.63E-05 | 0.00016021 | up |
| RP11-20E24.1\|ENSG00000257526\|lincRNA | 0.28479083 | 0.92738803 | 1.70327043 | 3.78E-10 | 1.79E-09 | up |
| AC005062.2\|ENSG00000243004\|sense_overlapping | 0.28342691 | 0.7841121 | 1.46808317 | 2.06E-06 | 5.51E-06 | up |
| C9orf147\|ENSG00000230185\|antisense | 0.28297395 | 0.59149566 | 1.06369834 | 1.82E-08 | 6.69E-08 | up |
| RP11-20G13.2\|ENSG00000259760\|antisense | 0.28296581 | 1.32207981 | 2.22410962 | 5.69E-05 | 0.00012131 | up |
| LINC00244\|ENSG00000279418\|lincRNA | 0.28179842 | 0.70006555 | 1.31282648 | 0.03155563 | 0.0419239 | up |
| ADD3-AS1\|ENSG00000203876\|processed_transcript | 0.28141209 | 0.90985815 | 1.69295735 | 1.07E-10 | 5.52E-10 | up |
| KB-1836B5.1\|ENSG00000260949\|sense_overlapping | 0.28119687 | 0.8163356 | 1.53758183 | 1.18E-08 | 4.46E-08 | up |
| ALMS1-IT1\|ENSG00000230002\|sense_intronic | 0.2795438 | 1.14949297 | 2.03985141 | 3.18E-14 | 2.97E-13 | up |
| RP11-231E4.5\|ENSG00000276934\|sense_intronic | 0.2794971 | 0.91586922 | 1.71230828 | 3.35E-05 | 7.40E-05 | up |
| ZMIZ1-AS1\|ENSG00000224596\|antisense | 0.27898666 | 0.66917882 | 1.26219564 | 9.31E-10 | 4.18E-09 | up |
| RP5-940J5.3\|ENSG00000255966\|antisense | 0.27785984 | 0.64541407 | 1.21586771 | 0.0002828 | 0.00054376 | up |
| RP11-231L11.3\|ENSG00000248773\|sense_intronic | 0.27760435 | 1.44426519 | 2.37923359 | 0.0098074 | 0.01426702 | up |
| RP11-98D18.17\|ENSG00000269489\|lincRNA | 0.27754677 | 0.67440226 | 1.28087846 | 2.61E-05 | 5.86E-05 | up |
| CTD-3035D6.2\|ENSG00000258884\|lincRNA | 0.27715159 | 1.20262106 | 2.11743495 | 1.23E-07 | 4.05E-07 | up |
| MIR503HG\|ENSG00000223749\|lincRNA | 0.27715045 | 2.7427106 | 3.30686117 | 3.95E-20 | 1.22E-18 | up |
| CTD-2020K17.3\|ENSG00000233175\|antisense | 0.27709559 | 0.70360124 | 1.34437431 | 1.02E-08 | 3.90E-08 | up |
| LINC00571\|ENSG00000223685\|lincRNA | 0.2768479 | 0.65783072 | 1.2486228 | 0.00013015 | 0.00026254 | up |
| RP4-539M6.20\|ENSG00000273350\|sense_intronic | 0.27629351 | 1.15316332 | 2.06132329 | 5.15E-06 | 1.30E-05 | up |
| AC004076.5\|ENSG00000276449\|antisense | 0.27598656 | 0.75485059 | 1.45159309 | 3.68E-11 | 2.03E-10 | up |
| XXbac-BPG157A10.21\|ENSG00000272217\|lincRNA | 0.27570416 | 0.79631472 | 1.53021769 | 1.73E-07 | 5.51E-07 | up |
| CTD-2562G15.3\|ENSG00000276744\|sense_intronic | 0.2753252 | 0.84059786 | 1.61027914 | 2.28E-12 | 1.58E-11 | up |
| RP11-265E18.1\|ENSG00000272905\|lincRNA | 0.27442838 | 0.671454 | 1.29085887 | 0.00051737 | 0.00094279 | up |
| U47924.32\|ENSG00000275703\|lincRNA | 0.27235279 | 0.70796773 | 1.37820694 | 1.93E-06 | 5.18E-06 | up |
| RP11-15N24.4\|ENSG00000261533\|sense_overlapping | 0.27225996 | 0.56273342 | 1.04746682 | 1.43E-08 | 5.36E-08 | up |
| LINCR-0001\|ENSG00000253641\|lincRNA | 0.2720181 | 1.65957548 | 2.60903971 | 7.38E-06 | 1.81E-05 | up |
| RP11-351D16.3\|ENSG00000273008\|lincRNA | 0.27118702 | 0.69601707 | 1.35983457 | 7.34E-12 | 4.66E-11 | up |
| FSIP2-AS1\|ENSG00000231646\|antisense | 0.27095989 | 1.0560684 | 1.96255205 | 8.46E-07 | 2.41E-06 | up |
| RAET1E-AS1\|ENSG00000268592\|antisense | 0.26946435 | 1.62026811 | 2.58806622 | 1.40E-07 | 4.55E-07 | up |
| AC018766.6\|ENSG00000268047\|antisense | 0.26942282 | 1.09238028 | 2.01953122 | 5.19E-09 | 2.09E-08 | up |
| RP11-895M11.3\|ENSG00000258798\|lincRNA | 0.26905091 | 0.83212511 | 1.62892128 | 5.43E-07 | 1.60E-06 | up |
| RP11-968A15.8\|ENSG00000258344\|sense_overlapping | 0.26903889 | 0.80827879 | 1.58703824 | 0.00100624 | 0.00174545 | up |
| RP11-474G23.3\|ENSG00000273064\|antisense | 0.26832629 | 0.92679916 | 1.78826832 | 3.70E-14 | 3.41E-13 | up |
| RP11-867G23.12\|ENSG00000254756\|antisense | 0.26681888 | 0.64279645 | 1.26850123 | 2.75E-06 | 7.22E-06 | up |
| RP5-991G20.2\|ENSG00000259209\|antisense | 0.26582662 | 0.84831995 | 1.6741229 | 2.17E-08 | 7.92E-08 | up |
| RP11-344N10.5\|ENSG00000272630\|lincRNA | 0.26549185 | 0.61509055 | 1.21213122 | 2.25E-10 | 1.11E-09 | up |
| ZNF252P-AS1\|ENSG00000255559\|antisense | 0.26543242 | 0.66641959 | 1.32808619 | 3.54E-08 | 1.26E-07 | up |
| RP11-476H16.1\|ENSG00000277020\|antisense | 0.26534903 | 0.75810946 | 1.51451492 | 1.00E-07 | 3.34E-07 | up |
| SMG7-AS1\|ENSG00000232860\|processed_transcript | 0.26517574 | 0.61612539 | 1.21627523 | 2.68E-12 | 1.84E-11 | up |
| RP11-861E21.1\|ENSG00000267108\|lincRNA | 0.26487556 | 1.10834201 | 2.06501651 | 8.91E-06 | 2.15E-05 | up |
| RP11-278A23.1\|ENSG00000226180\|lincRNA | 0.26351126 | 0.69465656 | 1.39843528 | 6.85E-12 | 4.37E-11 | up |
| RP4-798P15.3\|ENSG00000254154\|processed_transcript | 0.2623846 | 0.69665054 | 1.4087521 | 2.23E-07 | 6.99E-07 | up |
| ARHGEF38-IT1\|ENSG00000249885\|sense_intronic | 0.2623736 | 1.84705511 | 2.81553243 | 3.53E-10 | 1.68E-09 | up |
| CTD-2105E13.15\|ENSG00000269275\|antisense | 0.26190744 | 0.55603883 | 1.08612856 | 0.00381692 | 0.00595103 | up |
| TH2LCRR\|ENSG00000223442\|antisense | 0.26183166 | 0.67491962 | 1.36607615 | 0.00574718 | 0.00867724 | up |
| RP11-91P24.6\|ENSG00000255449\|antisense | 0.26166726 | 0.65986743 | 1.33444281 | 0.00287487 | 0.00459165 | up |
| KIAA0196-AS1\|ENSG00000253167\|antisense | 0.26112788 | 1.01085786 | 1.95275177 | 2.82E-07 | 8.65E-07 | up |
| PRR7-AS1\|ENSG00000246334\|antisense | 0.26108861 | 1.74626112 | 2.74165787 | 2.07E-22 | 1.19E-20 | up |
| RP11-467C18.1\|ENSG00000270457\|lincRNA | 0.26108325 | 0.59456056 | 1.18731386 | 0.02784122 | 0.03744081 | up |
| AC005532.5\|ENSG00000230825\|processed_transcript | 0.2599826 | 0.59216173 | 1.18757618 | 0.0065751 | 0.00983759 | up |
| RP11-620J15.1\|ENSG00000257953\|antisense | 0.25963592 | 1.03481352 | 1.99480891 | 9.76E-05 | 0.00020147 | up |
| CTC-303L1.2\|ENSG00000270133\|sense_intronic | 0.25930461 | 0.53349103 | 1.04081617 | 0.0311515 | 0.04144932 | up |
| AC011513.4\|ENSG00000268833\|antisense | 0.25926086 | 1.74544726 | 2.75112044 | 1.81E-07 | 5.74E-07 | up |
| MYB-AS1\|ENSG00000236703\|antisense | 0.25771647 | 1.1093154 | 2.10581299 | 0.00113873 | 0.00194598 | up |
| LINC00216\|ENSG00000279636\|lincRNA | 0.25759867 | 1.21701981 | 2.24015562 | 0.00213172 | 0.00349419 | up |
| RP11-138H8.8\|ENSG00000274297\|sense_intronic | 0.25755838 | 1.42952583 | 2.47256531 | 0.00014899 | 0.00029848 | up |
| LINC01301\|ENSG00000251396\|lincRNA | 0.25745622 | 0.99685298 | 1.95305362 | 3.19E-09 | 1.33E-08 | up |
| RP11-290D2.3\|ENSG00000228886\|lincRNA | 0.25666066 | 2.47511123 | 3.26955925 | 5.88E-05 | 0.0001249 | up |
| RP11-624L4.1\|ENSG00000259345\|lincRNA | 0.25661443 | 0.86121456 | 1.74677041 | 4.77E-06 | 1.21E-05 | up |
| RP11-316M21.6\|ENSG00000227492\|antisense | 0.25650109 | 0.52079817 | 1.02175945 | 5.33E-06 | 1.34E-05 | up |
| RP11-153I24.4\|ENSG00000277246\|sense_intronic | 0.25614439 | 0.57395594 | 1.16398272 | 0.03572341 | 0.04700169 | up |
| RP11-714G18.1\|ENSG00000250410\|antisense | 0.25393597 | 0.55190096 | 1.11994466 | 0.00102611 | 0.00177662 | up |
| RP11-253M7.1\|ENSG00000259426\|antisense | 0.25354428 | 0.62480233 | 1.30116214 | 1.32E-13 | 1.12E-12 | up |
| CTB-25B13.9\|ENSG00000267092\|antisense | 0.25290419 | 1.03603907 | 2.03441557 | 2.94E-12 | 2.00E-11 | up |
| AC058791.1\|ENSG00000226380\|lincRNA | 0.25216304 | 2.35510524 | 3.22336282 | 2.17E-23 | 1.77E-21 | up |
| C9orf41-AS1\|ENSG00000203321\|antisense | 0.25155336 | 0.94059105 | 1.90270317 | 0.00134575 | 0.00226984 | up |
| RP11-338E21.2\|ENSG00000225195\|sense_intronic | 0.25146062 | 1.17895672 | 2.22910638 | 0.00178093 | 0.00295207 | up |
| RP11-399C16.3\|ENSG00000279623\|sense_intronic | 0.25112741 | 0.73683856 | 1.55292907 | 0.01855094 | 0.02564238 | up |
| RP11-190C22.8\|ENSG00000272662\|lincRNA | 0.25111932 | 0.70875273 | 1.49690937 | 2.79E-06 | 7.32E-06 | up |
| LINC01348\|ENSG00000280587\|lincRNA | 0.25068284 | 1.23675444 | 2.30262394 | 2.01E-11 | 1.16E-10 | up |
| MIR1539\|ENSG00000265496\|antisense | 0.24955645 | 0.55002462 | 1.14013001 | 0.00042673 | 0.00079094 | up |
| CTD-3214H19.6\|ENSG00000268204\|antisense | 0.24835223 | 1.07322259 | 2.11148971 | 1.97E-11 | 1.14E-10 | up |
| RP11-166B2.3\|ENSG00000261560\|sense_intronic | 0.24829431 | 1.0472343 | 2.07646113 | 9.02E-13 | 6.72E-12 | up |
| LINC00954\|ENSG00000228784\|lincRNA | 0.24810404 | 0.6565768 | 1.40401854 | 0.00463531 | 0.00710166 | up |
| RP11-214K3.18\|ENSG00000270095\|sense_intronic | 0.24709775 | 1.02490178 | 2.05233189 | 0.00491765 | 0.0074974 | up |
| LINC00337\|ENSG00000225077\|lincRNA | 0.24645848 | 0.55071594 | 1.15996375 | 3.48E-07 | 1.05E-06 | up |
| RP11-626G11.4\|ENSG00000260430\|lincRNA | 0.24640399 | 0.77218735 | 1.64792533 | 2.73E-07 | 8.41E-07 | up |
| HCG14\|ENSG00000224157\|antisense | 0.24555319 | 0.51622352 | 1.0719603 | 0.00629461 | 0.00944459 | up |
| AC024560.2\|ENSG00000236833\|lincRNA | 0.24530878 | 0.51854261 | 1.07986368 | 0.00242405 | 0.00393323 | up |
| RP11-410E4.1\|ENSG00000259915\|lincRNA | 0.24499518 | 0.92724984 | 1.92020477 | 0.01350625 | 0.01917937 | up |
| AC016722.4\|ENSG00000228925\|antisense | 0.24422911 | 0.59028192 | 1.273169 | 2.66E-06 | 7.02E-06 | up |
| CTA-268H5.12\|ENSG00000273353\|sense_intronic | 0.24387087 | 0.84260648 | 1.78874159 | 0.00181231 | 0.0029994 | up |
| RP1-10C16.1\|ENSG00000235736\|lincRNA | 0.2435515 | 1.76404207 | 2.85658622 | 1.10E-05 | 2.62E-05 | up |
| CTB-134F13.1\|ENSG00000260545\|antisense | 0.24349384 | 0.56827099 | 1.22269379 | 0.01176803 | 0.01689204 | up |
| RP11-316M1.3\|ENSG00000231073\|antisense | 0.24301931 | 0.53920683 | 1.14976785 | 0.00111948 | 0.00191678 | up |
| RP11-10J21.4\|ENSG00000253307\|antisense | 0.24291691 | 1.86261725 | 2.93879644 | 1.22E-16 | 1.75E-15 | up |
| LINC01091\|ENSG00000249464\|lincRNA | 0.24277714 | 0.87166884 | 1.84414755 | 5.90E-13 | 4.54E-12 | up |
| FLJ31356\|ENSG00000229951\|antisense | 0.24275627 | 0.51062509 | 1.07275583 | 1.49E-05 | 3.49E-05 | up |
| RP11-483F11.7\|ENSG00000229278\|antisense | 0.24257412 | 0.52941767 | 1.12598075 | 0.00586133 | 0.0088345 | up |
| PRSS51\|ENSG00000253649\|antisense | 0.24183043 | 2.28735079 | 3.24160994 | 3.04E-06 | 7.94E-06 | up |
| RP11-98D18.1\|ENSG00000227045\|antisense | 0.24182902 | 0.67451541 | 1.47986399 | 0.03670805 | 0.04822538 | up |
| CRYM-AS1\|ENSG00000189149\|lincRNA | 0.24163696 | 0.73292592 | 1.60082627 | 1.85E-11 | 1.08E-10 | up |
| RP11-563K23.1\|ENSG00000272619\|lincRNA | 0.24092426 | 1.24351298 | 2.36776998 | 0.00166712 | 0.00277296 | up |
| RP1-118J21.25\|ENSG00000261798\|antisense | 0.24069642 | 1.06399988 | 2.14421142 | 5.88E-16 | 7.37E-15 | up |
| RP11-243J16.7\|ENSG00000236559\|antisense | 0.24013662 | 2.34388766 | 3.28697609 | 0.01022499 | 0.01482567 | up |
| MANEA-AS1\|ENSG00000261366\|antisense | 0.23998119 | 0.56206523 | 1.22781625 | 0.00033865 | 0.00063999 | up |
| RP11-473O4.5\|ENSG00000254317\|antisense | 0.23925312 | 0.75363579 | 1.65532977 | 1.58E-05 | 3.67E-05 | up |
| LINC01356\|ENSG00000215866\|lincRNA | 0.23920223 | 1.69711587 | 2.8267823 | 2.97E-19 | 7.13E-18 | up |
| RP11-65L3.2\|ENSG00000270277\|antisense | 0.23804745 | 0.59389972 | 1.3189702 | 0.01503464 | 0.02111781 | up |
| RP11-435O5.7\|ENSG00000271384\|lincRNA | 0.23689169 | 0.67102064 | 1.50212952 | 4.41E-06 | 1.12E-05 | up |
| RP11-687E1.2\|ENSG00000276174\|sense_intronic | 0.23678976 | 0.86565426 | 1.87018424 | 0.01110919 | 0.01598529 | up |
| FOCAD-AS1\|ENSG00000227071\|lincRNA | 0.23586429 | 0.83478489 | 1.82344745 | 5.77E-08 | 2.00E-07 | up |
| RP13-638C3.2\|ENSG00000262652\|sense_intronic | 0.23563206 | 1.01430766 | 2.10588756 | 0.00179749 | 0.00297583 | up |
| RP5-981O7.2\|ENSG00000229591\|antisense | 0.2348033 | 0.78589198 | 1.74287837 | 3.48E-06 | 8.99E-06 | up |
| RP11-295D4.5\|ENSG00000277440\|sense_intronic | 0.23421149 | 0.66977035 | 1.51585469 | 2.74E-06 | 7.20E-06 | up |
| LA16c-425C2.1\|ENSG00000260954\|sense_intronic | 0.23310529 | 0.59105174 | 1.34230269 | 1.68E-05 | 3.88E-05 | up |
| CTC-499B15.5\|ENSG00000197332\|lincRNA | 0.23286301 | 0.48478812 | 1.05787284 | 2.85E-07 | 8.74E-07 | up |
| RP11-483H20.4\|ENSG00000234789\|antisense | 0.23269066 | 0.49379137 | 1.0854883 | 0.0015663 | 0.00261511 | up |
| RP3-426I6.5\|ENSG00000269971\|sense_intronic | 0.2321838 | 1.15163151 | 2.31033993 | 0.00205873 | 0.00338188 | up |
| RP4-539M6.21\|ENSG00000272689\|sense_intronic | 0.23186199 | 0.88487804 | 1.93221231 | 0.00840968 | 0.01234742 | up |
| RP11-462L8.1\|ENSG00000229656\|lincRNA | 0.23125334 | 0.63418378 | 1.45542677 | 2.17E-09 | 9.29E-09 | up |
| RP11-120K24.5\|ENSG00000269376\|sense_intronic | 0.23118294 | 0.94298439 | 2.02819898 | 0.01373634 | 0.0194644 | up |
| RP11-73M7.6\|ENSG00000235790\|antisense | 0.23026963 | 0.66141225 | 1.52222563 | 4.75E-07 | 1.41E-06 | up |
| RP11-43F13.4\|ENSG00000272347\|lincRNA | 0.22822759 | 1.06525324 | 2.22265132 | 2.51E-11 | 1.43E-10 | up |
| CTC-459F4.1\|ENSG00000261770\|lincRNA | 0.22775933 | 0.49483341 | 1.11943273 | 0.00020686 | 0.00040614 | up |
| RP3-449M8.9\|ENSG00000270000\|lincRNA | 0.22745935 | 0.7928166 | 1.80137845 | 3.22E-07 | 9.78E-07 | up |
| C2orf48\|ENSG00000163009\|lincRNA | 0.22668195 | 1.54742826 | 2.7711311 | 4.74E-19 | 1.08E-17 | up |
| FAM222A-AS1\|ENSG00000255650\|antisense | 0.22618358 | 3.19152565 | 3.81868012 | 2.33E-22 | 1.32E-20 | up |
| LLNLR-246C6.1\|ENSG00000273218\|lincRNA | 0.22604828 | 0.5449584 | 1.26951513 | 1.31E-08 | 4.93E-08 | up |
| RP11-485G7.6\|ENSG00000262703\|antisense | 0.22499678 | 0.84786277 | 1.91392644 | 5.58E-13 | 4.31E-12 | up |
| U73166.2\|ENSG00000230454\|lincRNA | 0.22473954 | 0.78719793 | 1.80847244 | 5.58E-13 | 4.31E-12 | up |
| LL0XNC01-250H12.3\|ENSG00000234405\|antisense | 0.22470096 | 0.47427424 | 1.07771521 | 0.0144775 | 0.02040012 | up |
| RP11-118M9.3\|ENSG00000251556\|antisense | 0.22423072 | 0.48367942 | 1.10906719 | 0.01658202 | 0.02312583 | up |
| CTD-2525I3.5\|ENSG00000269102\|antisense | 0.2235538 | 0.77792311 | 1.79900551 | 0.0001704 | 0.00033818 | up |
| CATIP-AS1\|ENSG00000225062\|processed_transcript | 0.22329425 | 0.56304049 | 1.33429259 | 2.84E-06 | 7.43E-06 | up |
| RP11-449J21.5\|ENSG00000267128\|antisense | 0.22322791 | 0.5489926 | 1.29826929 | 9.38E-07 | 2.65E-06 | up |
| RP11-96O20.5\|ENSG00000275709\|lincRNA | 0.22292264 | 0.63779215 | 1.51654323 | 1.58E-05 | 3.67E-05 | up |
| RP5-827C21.6\|ENSG00000273367\|antisense | 0.22276822 | 0.7299171 | 1.71218915 | 0.00357176 | 0.00559842 | up |
| RP11-186F10.2\|ENSG00000257761\|antisense | 0.22249625 | 2.14213485 | 3.26719638 | 6.94E-13 | 5.26E-12 | up |
| CTC-325J23.2\|ENSG00000249655\|antisense | 0.22239829 | 0.54373096 | 1.28974731 | 0.00012815 | 0.00025898 | up |
| RP5-956O18.2\|ENSG00000227006\|antisense | 0.22226097 | 0.79534422 | 1.83932476 | 0.00108118 | 0.00186083 | up |
| RP5-1085F17.4\|ENSG00000260536\|antisense | 0.22202628 | 1.4490306 | 2.7062857 | 1.86E-06 | 5.00E-06 | up |
| RP3-324O17.8\|ENSG00000278276\|lincRNA | 0.22156642 | 0.50901158 | 1.19995924 | 0.00188454 | 0.00310923 | up |
| SLC2A1-AS1\|ENSG00000227533\|lincRNA | 0.22068812 | 0.63928534 | 1.53445105 | 8.57E-08 | 2.89E-07 | up |
| LMO7-AS1\|ENSG00000261105\|antisense | 0.21913391 | 2.16055605 | 3.30151802 | 4.18E-18 | 7.67E-17 | up |
| RP11-72M17.1\|ENSG00000258561\|lincRNA | 0.21866043 | 0.86647403 | 1.98646437 | 1.13E-19 | 2.97E-18 | up |
| RP11-17J14.2\|ENSG00000266495\|antisense | 0.21825533 | 0.4953796 | 1.18251754 | 0.00035257 | 0.00066346 | up |
| RP11-2C24.7\|ENSG00000274678\|lincRNA | 0.21767825 | 0.62417411 | 1.51975126 | 1.33E-05 | 3.13E-05 | up |
| FTO-IT1\|ENSG00000260936\|sense_intronic | 0.21734508 | 0.62224266 | 1.51748987 | 0.00032293 | 0.00061335 | up |
| UBOX5-AS1\|ENSG00000235958\|antisense | 0.21707582 | 0.5754521 | 1.40649684 | 2.45E-07 | 7.62E-07 | up |
| RP11-66N24.4\|ENSG00000157306\|processed_transcript | 0.21704608 | 0.46025518 | 1.08443263 | 1.01E-07 | 3.36E-07 | up |
| RP11-500B12.1\|ENSG00000233569\|lincRNA | 0.21646535 | 1.09331729 | 2.33650416 | 0.00263714 | 0.00424908 | up |
| RP11-92G12.3\|ENSG00000260088\|lincRNA | 0.21642634 | 0.51891427 | 1.2616201 | 0.00413334 | 0.00639545 | up |
| FARP1-AS1\|ENSG00000231194\|antisense | 0.21629177 | 0.81572733 | 1.9151082 | 1.30E-05 | 3.06E-05 | up |
| RP4-564F22.7\|ENSG00000275401\|lincRNA | 0.21477135 | 0.74385528 | 1.79222042 | 4.23E-08 | 1.48E-07 | up |
| AC108463.1\|ENSG00000230499\|lincRNA | 0.21427166 | 0.89414392 | 2.06106604 | 8.50E-09 | 3.29E-08 | up |
| RP11-603J24.5\|ENSG00000258317\|antisense | 0.21352584 | 0.51186357 | 1.26134867 | 5.51E-07 | 1.62E-06 | up |
| RP11-837J7.4\|ENSG00000277299\|antisense | 0.21327067 | 0.49599507 | 1.21764022 | 0.00033446 | 0.00063275 | up |
| C1orf147\|ENSG00000162888\|antisense | 0.21321238 | 0.5472177 | 1.35982368 | 7.23E-08 | 2.47E-07 | up |
| RP11-57A19.2\|ENSG00000246465\|lincRNA | 0.21260937 | 1.15656924 | 2.44357457 | 1.43E-10 | 7.25E-10 | up |
| RP4-620E11.8\|ENSG00000273951\|sense_intronic | 0.21258958 | 1.02396928 | 2.26802968 | 2.10E-09 | 8.96E-09 | up |
| AC017002.1\|ENSG00000240350\|lincRNA | 0.21181671 | 0.66693201 | 1.6547233 | 7.61E-07 | 2.19E-06 | up |
| RP4-564F22.5\|ENSG00000224635\|lincRNA | 0.21155503 | 0.96276097 | 2.18614467 | 9.69E-11 | 5.05E-10 | up |
| RP11-776H12.1\|ENSG00000226476\|lincRNA | 0.21098505 | 3.90369739 | 4.20962857 | 1.45E-14 | 1.43E-13 | up |
| XXbac-B444P24.14\|ENSG00000273139\|lincRNA | 0.21097783 | 0.49790209 | 1.23877065 | 0.00254667 | 0.00412042 | up |
| RP11-845M18.6\|ENSG00000257829\|antisense | 0.21088702 | 0.58651228 | 1.47569098 | 1.60E-05 | 3.71E-05 | up |
| RP11-96D1.10\|ENSG00000263276\|sense_overlapping | 0.21050953 | 0.45501853 | 1.11203973 | 4.86E-06 | 1.23E-05 | up |
| AC005775.2\|ENSG00000266933\|antisense | 0.21014904 | 0.54987336 | 1.38768656 | 2.90E-09 | 1.22E-08 | up |
| RP13-890H12.2\|ENSG00000267288\|antisense | 0.20961048 | 1.0091304 | 2.26732987 | 2.70E-17 | 4.36E-16 | up |
| RP11-384C4.2\|ENSG00000229832\|sense_intronic | 0.20954807 | 0.64456123 | 1.62103616 | 0.00687144 | 0.01025493 | up |
| RP11-127I20.8\|ENSG00000275056\|sense_intronic | 0.20906573 | 0.72235927 | 1.78875996 | 1.12E-07 | 3.71E-07 | up |
| MESTIT1\|ENSG00000272701\|antisense | 0.20899605 | 0.45702468 | 1.12879642 | 1.40E-07 | 4.55E-07 | up |
| RP11-61K9.2\|ENSG00000223576\|antisense | 0.20892652 | 0.53338935 | 1.35219338 | 0.00574157 | 0.00867125 | up |
| RP11-497G19.1\|ENSG00000257883\|lincRNA | 0.20887816 | 2.40581304 | 3.525791 | 1.40E-05 | 3.29E-05 | up |
| RP11-255C15.3\|ENSG00000260743\|lincRNA | 0.2086424 | 0.51352717 | 1.29940828 | 2.20E-07 | 6.90E-07 | up |
| AC007285.6\|ENSG00000227014\|antisense | 0.20827721 | 0.51787319 | 1.3140939 | 0.00070437 | 0.00125256 | up |
| AC005954.3\|ENSG00000267138\|sense_intronic | 0.20789186 | 0.45222229 | 1.12119884 | 0.0082027 | 0.01207653 | up |
| AP000695.6\|ENSG00000230479\|antisense | 0.20774134 | 0.95116142 | 2.19490188 | 5.55E-11 | 2.99E-10 | up |
| LA16c-385E7.1\|ENSG00000260646\|lincRNA | 0.20761309 | 0.54164205 | 1.38344233 | 1.51E-05 | 3.53E-05 | up |
| RP11-843B15.4\|ENSG00000277342\|sense_intronic | 0.20674665 | 1.55167641 | 2.90789189 | 7.68E-17 | 1.14E-15 | up |
| C9orf163\|ENSG00000196366\|lincRNA | 0.20673158 | 0.83667064 | 2.016901 | 7.44E-18 | 1.33E-16 | up |
| RP4-816N1.7\|ENSG00000258092\|antisense | 0.2058826 | 0.97870475 | 2.24905178 | 2.51E-09 | 1.06E-08 | up |
| CTC-228N24.2\|ENSG00000250603\|lincRNA | 0.20574572 | 0.74361659 | 1.85369654 | 0.00702369 | 0.01045566 | up |
| RP5-890E16.2\|ENSG00000263412\|processed_transcript | 0.20539987 | 0.53743678 | 1.38765981 | 3.53E-09 | 1.46E-08 | up |
| ZNF341-AS1\|ENSG00000230753\|antisense | 0.20473807 | 0.51259386 | 1.32403678 | 4.45E-06 | 1.13E-05 | up |
| RP11-496D24.2\|ENSG00000261121\|lincRNA | 0.20444545 | 0.69001212 | 1.75490573 | 0.00123524 | 0.0020967 | up |
| RP11-285G1.14\|ENSG00000273363\|sense_intronic | 0.20428608 | 0.57475658 | 1.49236014 | 7.96E-06 | 1.94E-05 | up |
| RP11-402D21.2\|ENSG00000272817\|antisense | 0.2041383 | 0.41532621 | 1.02469805 | 0.00036598 | 0.00068626 | up |
| AC008781.7\|ENSG00000228737\|antisense | 0.2031561 | 0.62732443 | 1.62662307 | 0.00027487 | 0.00052928 | up |
| AC019186.1\|ENSG00000234584\|lincRNA | 0.20301606 | 0.47213891 | 1.21761751 | 0.00016288 | 0.00032435 | up |
| CTD-2192J16.26\|ENSG00000268945\|lincRNA | 0.20298814 | 0.66702007 | 1.71633476 | 6.21E-10 | 2.84E-09 | up |
| AC006160.5\|ENSG00000249502\|antisense | 0.20292767 | 0.50506296 | 1.31549768 | 0.00471204 | 0.00721089 | up |
| CTD-2026D20.2\|ENSG00000253347\|antisense | 0.20260685 | 0.65502318 | 1.692863 | 0.00014647 | 0.00029389 | up |
| RP11-103B5.4\|ENSG00000277840\|sense_intronic | 0.20232428 | 1.75508311 | 3.116798 | 3.20E-16 | 4.25E-15 | up |
| DLGAP4-AS1\|ENSG00000232907\|antisense | 0.20144186 | 0.76211934 | 1.91965343 | 1.32E-12 | 9.53E-12 | up |
| RP5-1057I20.5\|ENSG00000276691\|antisense | 0.20133913 | 0.80987164 | 2.0080657 | 1.00E-05 | 2.39E-05 | up |
| LINC01132\|ENSG00000227630\|lincRNA | 0.20098133 | 0.76114548 | 1.92111074 | 1.59E-18 | 3.25E-17 | up |
| RP11-128N14.4\|ENSG00000276704\|sense_intronic | 0.20093886 | 1.17877798 | 2.55246351 | 7.05E-06 | 1.74E-05 | up |
| RP4-622L5.7\|ENSG00000224066\|antisense | 0.20053119 | 0.56267564 | 1.48847684 | 6.27E-10 | 2.87E-09 | up |
| RP11-326C3.15\|ENSG00000270972\|lincRNA | 0.20000529 | 1.11672199 | 2.48116001 | 4.98E-05 | 0.00010706 | up |
| RP11-677I18.3\|ENSG00000254433\|antisense | 0.19991907 | 0.52442944 | 1.39133263 | 0.00229827 | 0.00374866 | up |
| DARS-AS1\|ENSG00000231890\|antisense | 0.19969708 | 0.4970067 | 1.31545208 | 2.31E-10 | 1.13E-09 | up |
| EIF2B5-AS1\|ENSG00000230215\|antisense | 0.19966263 | 1.06966177 | 2.42151843 | 0.00200777 | 0.00330123 | up |
| AL163953.2\|ENSG00000225680\|lincRNA | 0.19939823 | 3.21178519 | 4.00965091 | 1.64E-19 | 4.19E-18 | up |
| CTD-3195I5.4\|ENSG00000262358\|antisense | 0.1988502 | 0.52710427 | 1.40640639 | 0.00017603 | 0.00034883 | up |
| RP11-817I4.2\|ENSG00000276115\|sense_intronic | 0.19881085 | 0.86799024 | 2.12628229 | 4.56E-15 | 4.92E-14 | up |
| RP11-875O11.3\|ENSG00000253616\|antisense | 0.19786755 | 1.99219371 | 3.33175103 | 1.35E-19 | 3.53E-18 | up |
| CTD-2033A16.3\|ENSG00000262136\|antisense | 0.19781066 | 0.82248385 | 2.05586716 | 1.82E-09 | 7.84E-09 | up |
| CTB-40H15.4\|ENSG00000272057\|lincRNA | 0.19725862 | 0.52244162 | 1.40518151 | 0.00054009 | 0.00098217 | up |
| MGC45922\|ENSG00000180279\|lincRNA | 0.19675951 | 0.50733543 | 1.36650657 | 8.91E-07 | 2.52E-06 | up |
| CRNDE\|ENSG00000245694\|lincRNA | 0.19647359 | 6.40947673 | 5.02779925 | 8.91E-25 | 1.35E-22 | up |
| RP4-550H1.7\|ENSG00000277235\|lincRNA | 0.19630299 | 0.6417629 | 1.70895825 | 3.82E-05 | 8.35E-05 | up |
| RP11-973D8.5\|ENSG00000273973\|sense_intronic | 0.19625676 | 0.40165397 | 1.0332108 | 0.00313612 | 0.0049685 | up |
| RP5-1014D13.2\|ENSG00000279738\|sense_overlapping | 0.19603101 | 0.48535214 | 1.30795 | 7.95E-18 | 1.39E-16 | up |
| GAS8-AS1\|ENSG00000221819\|antisense | 0.19593646 | 0.92173535 | 2.2339667 | 2.23E-07 | 6.99E-07 | up |
| RP11-367H1.1\|ENSG00000273113\|lincRNA | 0.19539651 | 0.78454531 | 2.00545204 | 6.08E-11 | 3.27E-10 | up |
| BBOX1-AS1\|ENSG00000254560\|antisense | 0.19526499 | 6.99001815 | 5.16179099 | 1.83E-18 | 3.69E-17 | up |
| RP11-365P13.5\|ENSG00000277767\|lincRNA | 0.19499723 | 0.56877512 | 1.54440471 | 3.88E-08 | 1.37E-07 | up |
| RP11-44I10.3\|ENSG00000261267\|antisense | 0.19493944 | 1.29091474 | 2.7272958 | 0.00154474 | 0.00258317 | up |
| RP11-417F21.1\|ENSG00000250116\|antisense | 0.19476887 | 0.95041087 | 2.28678823 | 0.00431996 | 0.00665701 | up |
| STT3A-AS1\|ENSG00000254671\|antisense | 0.19474599 | 0.56915574 | 1.54722988 | 0.00772541 | 0.01141372 | up |
| RP11-261P13.6\|ENSG00000275485\|sense_intronic | 0.19428868 | 1.04201593 | 2.42310361 | 2.31E-11 | 1.32E-10 | up |
| RP11-566K11.7\|ENSG00000267048\|processed_transcript | 0.19393637 | 0.73749668 | 1.92705318 | 1.57E-09 | 6.83E-09 | up |
| RP11-200A1.1\|ENSG00000240006\|lincRNA | 0.19367893 | 0.50961668 | 1.39574548 | 0.03192099 | 0.04237746 | up |
| RP11-140H17.2\|ENSG00000274093\|sense_intronic | 0.19334835 | 3.56208859 | 4.20344903 | 0.01040437 | 0.01506929 | up |
| LINC00449\|ENSG00000203441\|antisense | 0.19334579 | 0.58172352 | 1.58915027 | 5.62E-10 | 2.59E-09 | up |
| STAM-AS1\|ENSG00000260589\|antisense | 0.19328694 | 0.5401122 | 1.48251497 | 1.25E-10 | 6.35E-10 | up |
| AC015987.1\|ENSG00000224746\|antisense | 0.19288859 | 0.66209875 | 1.77927861 | 3.52E-05 | 7.74E-05 | up |
| RP11-467D18.2\|ENSG00000237934\|antisense | 0.19266897 | 0.50364079 | 1.38627089 | 0.00451728 | 0.00693688 | up |
| RP11-248G5.9\|ENSG00000273523\|antisense | 0.19235014 | 0.44544602 | 1.21151577 | 0.00028292 | 0.00054378 | up |
| RP11-392O17.2\|ENSG00000277007\|lincRNA | 0.19112571 | 0.45132762 | 1.2396532 | 9.35E-05 | 0.00019355 | up |
| RP11-247A12.7\|ENSG00000268707\|lincRNA | 0.1900977 | 0.61583117 | 1.69579383 | 3.54E-05 | 7.78E-05 | up |
| LINC01424\|ENSG00000236519\|antisense | 0.18962094 | 0.61440814 | 1.69607905 | 5.63E-11 | 3.03E-10 | up |
| RP11-661A12.12\|ENSG00000254812\|antisense | 0.18892566 | 0.44193243 | 1.22600713 | 0.00130469 | 0.0022061 | up |
| RP11-4K16.2\|ENSG00000253972\|lincRNA | 0.18852164 | 1.08951037 | 2.53087793 | 4.00E-05 | 8.72E-05 | up |
| RP11-362K14.5\|ENSG00000269984\|antisense | 0.18833697 | 0.63283702 | 1.74851777 | 2.39E-07 | 7.44E-07 | up |
| RP11-598F7.5\|ENSG00000256694\|antisense | 0.18795011 | 1.36779075 | 2.86342584 | 4.79E-21 | 1.89E-19 | up |
| CYP4A22-AS1\|ENSG00000225506\|lincRNA | 0.18794961 | 1.01911371 | 2.43889723 | 1.39E-17 | 2.33E-16 | up |
| RP11-55K13.1\|ENSG00000260495\|antisense | 0.18723047 | 0.52809291 | 1.49597656 | 6.56E-09 | 2.59E-08 | up |
| RP3-508I15.18\|ENSG00000244491\|antisense | 0.18713654 | 0.58491851 | 1.64414433 | 0.00038103 | 0.00071246 | up |
| RP11-264B17.3\|ENSG00000261067\|processed_transcript | 0.18694249 | 0.39679705 | 1.08580676 | 1.70E-08 | 6.31E-08 | up |
| AP001619.2\|ENSG00000227698\|sense_intronic | 0.18682231 | 0.63645137 | 1.76838355 | 1.32E-05 | 3.11E-05 | up |
| LINC00562\|ENSG00000260388\|lincRNA | 0.18665511 | 0.55294298 | 1.5667557 | 2.10E-07 | 6.61E-07 | up |
| SRD5A3-AS1\|ENSG00000249700\|processed_transcript | 0.18649329 | 0.38066842 | 1.02941117 | 4.76E-07 | 1.41E-06 | up |
| PRC1-AS1\|ENSG00000258725\|antisense | 0.18598736 | 0.45473965 | 1.2898362 | 1.66E-05 | 3.84E-05 | up |
| RP11-863P13.3\|ENSG00000261327\|lincRNA | 0.18447169 | 0.89687214 | 2.28150291 | 9.66E-10 | 4.32E-09 | up |
| ARAP1-AS2\|ENSG00000245148\|antisense | 0.18419248 | 0.69354838 | 1.91278241 | 4.07E-05 | 8.85E-05 | up |
| RP11-462D18.4\|ENSG00000277373\|sense_intronic | 0.18415701 | 0.45373844 | 1.30092461 | 0.01357265 | 0.01925819 | up |
| LA16c-380H5.1\|ENSG00000262152\|processed_transcript | 0.1840458 | 0.61758851 | 1.7465811 | 1.07E-05 | 2.54E-05 | up |
| RP11-351J23.2\|ENSG00000272549\|lincRNA | 0.18263569 | 0.48346101 | 1.40443082 | 8.81E-05 | 0.00018336 | up |
| RP3-399L15.3\|ENSG00000228624\|antisense | 0.18174363 | 0.42739178 | 1.23365432 | 5.36E-12 | 3.51E-11 | up |
| C17orf82\|ENSG00000187013\|lincRNA | 0.18105123 | 0.65852865 | 1.86284825 | 1.10E-08 | 4.18E-08 | up |
| C17orf77\|ENSG00000182352\|antisense | 0.18066581 | 2.99557341 | 4.0514368 | 8.37E-07 | 2.39E-06 | up |
| RP11-120D5.1\|ENSG00000234129\|lincRNA | 0.18065904 | 0.56290079 | 1.63961123 | 2.56E-15 | 2.85E-14 | up |
| RP11-29H23.4\|ENSG00000232519\|antisense | 0.18059076 | 0.621324 | 1.78262172 | 1.68E-05 | 3.89E-05 | up |
| CTC-806A22.1\|ENSG00000250842\|lincRNA | 0.18044279 | 0.48379119 | 1.42284298 | 0.01389099 | 0.01964678 | up |
| RP11-449G16.1\|ENSG00000273035\|sense_intronic | 0.18024072 | 1.21733943 | 2.75573461 | 0.00544843 | 0.00825676 | up |
| RP5-1009E24.9\|ENSG00000274949\|processed_transcript | 0.1794304 | 0.51732165 | 1.52763724 | 1.75E-05 | 4.04E-05 | up |
| RP11-527N22.2\|ENSG00000253746\|lincRNA | 0.17921981 | 2.14979372 | 3.58439622 | 3.89E-17 | 6.10E-16 | up |
| RP11-619A14.2\|ENSG00000254933\|antisense | 0.17870951 | 1.346222 | 2.91322803 | 0.00039584 | 0.00073782 | up |
| RP11-537A6.9\|ENSG00000233144\|antisense | 0.17870811 | 0.76088528 | 2.09007383 | 5.13E-08 | 1.79E-07 | up |
| RP11-303E16.3\|ENSG00000260213\|antisense | 0.17823119 | 0.52937541 | 1.5705414 | 6.80E-15 | 7.12E-14 | up |
| RP11-282O18.7\|ENSG00000269980\|antisense | 0.17822769 | 0.43577148 | 1.28985026 | 0.01226629 | 0.0175455 | up |
| RP3-406P24.4\|ENSG00000272248\|lincRNA | 0.17743187 | 0.37155751 | 1.06632038 | 0.0246762 | 0.03354065 | up |
| RP11-1000B6.5\|ENSG00000244952\|lincRNA | 0.17740927 | 0.40440233 | 1.18870997 | 0.00011543 | 0.00023562 | up |
| RP11-13P5.2\|ENSG00000233682\|antisense | 0.17739112 | 0.62878391 | 1.82563055 | 1.40E-05 | 3.28E-05 | up |
| ODF2-AS1\|ENSG00000225951\|antisense | 0.17673472 | 0.67838167 | 1.94051173 | 2.27E-05 | 5.15E-05 | up |
| RP11-5G9.5\|ENSG00000274001\|sense_intronic | 0.17648982 | 1.23418156 | 2.80589776 | 2.30E-07 | 7.21E-07 | up |
| RP11-323F24.3\|ENSG00000276524\|lincRNA | 0.17617306 | 0.46725808 | 1.40722631 | 1.21E-05 | 2.86E-05 | up |
| RP11-757O6.1\|ENSG00000264222\|lincRNA | 0.1748734 | 0.88422735 | 2.33810653 | 1.41E-06 | 3.88E-06 | up |
| CASC21\|ENSG00000253929\|lincRNA | 0.17459775 | 21.6833297 | 6.95640755 | 3.24E-23 | 2.45E-21 | up |
| RP11-141B14.1\|ENSG00000270019\|lincRNA | 0.17450461 | 0.48454842 | 1.4733757 | 2.68E-05 | 6.02E-05 | up |
| RP11-21B21.4\|ENSG00000261744\|antisense | 0.17413182 | 0.74300852 | 2.09319888 | 7.50E-09 | 2.93E-08 | up |
| RP4-625H18.2\|ENSG00000228412\|lincRNA | 0.17403005 | 0.54342822 | 1.64275307 | 0.0281205 | 0.037771 | up |
| RP11-632F7.3\|ENSG00000250906\|antisense | 0.17299558 | 0.55503244 | 1.68183693 | 0.0014564 | 0.00244362 | up |
| RP11-10A14.4\|ENSG00000253426\|lincRNA | 0.17286026 | 1.02542972 | 2.56855047 | 9.18E-13 | 6.81E-12 | up |
| CTD-2012K14.6\|ENSG00000261386\|lincRNA | 0.17261749 | 0.45998238 | 1.41399993 | 7.24E-06 | 1.78E-05 | up |
| KLF7-IT1\|ENSG00000237892\|sense_intronic | 0.17247764 | 1.0884476 | 2.65779071 | 0.00084026 | 0.00147539 | up |
| RP11-640I15.1\|ENSG00000266261\|antisense | 0.17236436 | 0.4140149 | 1.2642212 | 0.00012114 | 0.00024614 | up |
| RP11-535C21.3\|ENSG00000236896\|antisense | 0.17223695 | 0.97006433 | 2.49368571 | 0.00714275 | 0.01062096 | up |
| RP11-60A8.1\|ENSG00000249743\|lincRNA | 0.1722125 | 2.29648641 | 3.73716651 | 5.19E-06 | 1.31E-05 | up |
| RP11-413M3.4\|ENSG00000227512\|antisense | 0.17185963 | 0.76328474 | 2.15099068 | 1.54E-08 | 5.72E-08 | up |
| TM4SF19-AS1\|ENSG00000235897\|antisense | 0.17144413 | 0.40507568 | 1.24045301 | 1.31E-07 | 4.30E-07 | up |
| LA16c-329F2.2\|ENSG00000275092\|sense_intronic | 0.17105634 | 1.02161686 | 2.57831075 | 3.80E-17 | 5.98E-16 | up |
| RP11-326C3.12\|ENSG00000255328\|lincRNA | 0.17082517 | 0.76026879 | 2.15398901 | 3.87E-08 | 1.37E-07 | up |
| RP11-57A1.1\|ENSG00000263680\|lincRNA | 0.17080539 | 0.93859088 | 2.45814297 | 7.86E-11 | 4.16E-10 | up |
| GK-IT1\|ENSG00000229331\|sense_intronic | 0.17079003 | 1.48309313 | 3.11831354 | 3.70E-06 | 9.51E-06 | up |
| RP13-216E22.5\|ENSG00000271199\|sense_overlapping | 0.1702458 | 1.45688719 | 3.09719808 | 2.95E-06 | 7.71E-06 | up |
| RP11-203B9.4\|ENSG00000226803\|antisense | 0.17019613 | 0.51801966 | 1.60580868 | 0.00024266 | 0.00047085 | up |
| HULC\|ENSG00000251164\|lincRNA | 0.1696635 | 53.214763 | 8.2930065 | 3.57E-07 | 1.08E-06 | up |
| RP11-174G6.1\|ENSG00000214772\|antisense | 0.16917976 | 0.66917892 | 1.98383504 | 0.00070249 | 0.00124963 | up |
| RP11-33I11.2\|ENSG00000254432\|antisense | 0.16871313 | 1.4137162 | 3.06684836 | 0.01668824 | 0.02325562 | up |
| MLK7-AS1\|ENSG00000238133\|antisense | 0.1686951 | 3.36630252 | 4.31867486 | 4.18E-18 | 7.67E-17 | up |
| CTC-268N12.2\|ENSG00000278638\|antisense | 0.16862146 | 0.63922521 | 1.92253612 | 5.73E-05 | 0.00012188 | up |
| KB-1980E6.3\|ENSG00000253633\|lincRNA | 0.1680228 | 0.46400957 | 1.46549757 | 0.00333253 | 0.00525765 | up |
| RP4-585I14.3\|ENSG00000278153\|lincRNA | 0.16755887 | 0.5615574 | 1.74476541 | 5.54E-06 | 1.39E-05 | up |
| LINC01564\|ENSG00000235899\|antisense | 0.16735117 | 0.8424216 | 2.33166378 | 5.97E-05 | 0.00012666 | up |
| RP4-607I7.1\|ENSG00000255521\|antisense | 0.16661386 | 0.52292119 | 1.65008516 | 6.11E-09 | 2.42E-08 | up |
| RP11-159F24.5\|ENSG00000248240\|antisense | 0.16648027 | 0.35217797 | 1.08095343 | 0.02977241 | 0.03978411 | up |
| LRP1-AS\|ENSG00000259125\|antisense | 0.16580371 | 0.48652447 | 1.55303605 | 1.14E-06 | 3.17E-06 | up |
| GRPEL2-AS1\|ENSG00000253618\|antisense | 0.16564477 | 0.34414901 | 1.05494074 | 0.02442284 | 0.03321331 | up |
| RP11-524F11.2\|ENSG00000264666\|antisense | 0.16530914 | 0.38218877 | 1.20911894 | 0.00103705 | 0.00179302 | up |
| RP11-343H5.6\|ENSG00000237605\|antisense | 0.16469386 | 0.46376975 | 1.49362193 | 0.00060264 | 0.00108438 | up |
| MIR34AHG\|ENSG00000228526\|lincRNA | 0.16429673 | 0.66360285 | 2.01401635 | 5.92E-08 | 2.05E-07 | up |
| RP11-552F3.4\|ENSG00000266980\|antisense | 0.16420691 | 0.37328519 | 1.18476344 | 0.00021568 | 0.00042221 | up |
| RP11-133N21.10\|ENSG00000257964\|antisense | 0.16357693 | 0.62099161 | 1.92460444 | 0.00044736 | 0.00082611 | up |
| RP4-694A7.2\|ENSG00000233589\|antisense | 0.16326562 | 0.96815244 | 2.56801322 | 3.48E-06 | 8.99E-06 | up |
| RP11-429J17.7\|ENSG00000254973\|lincRNA | 0.16273053 | 1.22623213 | 2.91367531 | 7.19E-21 | 2.76E-19 | up |
| TSPEAR-AS1\|ENSG00000235890\|antisense | 0.16259311 | 0.82021791 | 2.33474114 | 1.04E-08 | 3.98E-08 | up |
| RP11-91P24.7\|ENSG00000254459\|antisense | 0.16244434 | 0.41726336 | 1.3610128 | 0.0025062 | 0.00405783 | up |
| RP11-180O5.2\|ENSG00000226088\|antisense | 0.16163786 | 0.66088498 | 2.03163405 | 0.0037795 | 0.00589962 | up |
| RP11-415J8.5\|ENSG00000233246\|antisense | 0.1614623 | 0.49720789 | 1.62265189 | 0.00541914 | 0.00821471 | up |
| RP11-386G11.5\|ENSG00000257913\|antisense | 0.16053983 | 1.64242489 | 3.35482425 | 1.15E-23 | 1.07E-21 | up |
| RP11-155G14.6\|ENSG00000240758\|processed_transcript | 0.16049014 | 0.80555022 | 2.32748986 | 1.49E-12 | 1.07E-11 | up |
| SMPD5\|ENSG00000204791\|antisense | 0.16043088 | 0.64597136 | 2.00951831 | 2.23E-13 | 1.82E-12 | up |
| AP001610.5\|ENSG00000228318\|antisense | 0.15948096 | 0.33246707 | 1.05982727 | 0.02686241 | 0.03621506 | up |
| AC007285.7\|ENSG00000231519\|antisense | 0.15933847 | 0.9162093 | 2.52358262 | 1.54E-09 | 6.69E-09 | up |
| RP11-424I19.2\|ENSG00000259182\|lincRNA | 0.15922334 | 0.33936687 | 1.09179388 | 0.00075181 | 0.00132933 | up |
| RP3-522J7.7\|ENSG00000279345\|antisense | 0.1591582 | 0.43534015 | 1.4516816 | 0.00870785 | 0.01275852 | up |
| RP11-568A7.3\|ENSG00000231720\|lincRNA | 0.15898598 | 0.94786896 | 2.57578807 | 8.96E-06 | 2.16E-05 | up |
| CTD-2371O3.2\|ENSG00000254397\|antisense | 0.15865462 | 0.94450413 | 2.57366756 | 2.25E-07 | 7.04E-07 | up |
| RP6-74O6.2\|ENSG00000215859\|lincRNA | 0.15863406 | 0.33644327 | 1.08466073 | 8.31E-06 | 2.02E-05 | up |
| RP5-908M14.5\|ENSG00000233017\|antisense | 0.15849572 | 2.72685528 | 4.10472239 | 1.30E-18 | 2.72E-17 | up |
| RP11-809N8.4\|ENSG00000256448\|antisense | 0.1584484 | 0.46314543 | 1.54745217 | 0.00757844 | 0.01122473 | up |
| LINC01252\|ENSG00000247157\|lincRNA | 0.15797052 | 0.42303589 | 1.42112475 | 2.00E-05 | 4.56E-05 | up |
| RP11-66H6.4\|ENSG00000274038\|sense_intronic | 0.15764966 | 0.87810581 | 2.47767271 | 4.78E-08 | 1.67E-07 | up |
| AP001429.1\|ENSG00000270116\|sense_intronic | 0.15688051 | 2.6094484 | 4.05600688 | 8.36E-08 | 2.83E-07 | up |
| RP5-1011O1.2\|ENSG00000232498\|antisense | 0.15577161 | 3.09196363 | 4.31101916 | 6.47E-14 | 5.76E-13 | up |
| RP11-136K14.1\|ENSG00000232891\|sense_intronic | 0.15572223 | 0.78607406 | 2.33569034 | 0.01407644 | 0.01988785 | up |
| RP5-902P8.12\|ENSG00000260179\|lincRNA | 0.15525719 | 0.34324977 | 1.14459861 | 2.62E-07 | 8.11E-07 | up |
| ENO1-IT1\|ENSG00000236269\|sense_intronic | 0.1551619 | 0.81295314 | 2.38939782 | 2.55E-07 | 7.90E-07 | up |
| RP5-1009E24.8\|ENSG00000275491\|lincRNA | 0.15481003 | 1.4952332 | 3.27179965 | 6.17E-18 | 1.12E-16 | up |
| RP11-705O1.8\|ENSG00000263884\|lincRNA | 0.15470969 | 0.32529515 | 1.07218573 | 9.43E-06 | 2.27E-05 | up |
| RP11-30L15.6\|ENSG00000260955\|lincRNA | 0.15460891 | 0.57308261 | 1.89011961 | 0.00168634 | 0.00280228 | up |
| AC104654.2\|ENSG00000234362\|lincRNA | 0.15377813 | 0.50047234 | 1.70244001 | 0.00111877 | 0.00191622 | up |
| RP11-159H10.3\|ENSG00000246528\|antisense | 0.15376144 | 0.75318602 | 2.29231246 | 1.47E-10 | 7.43E-10 | up |
| LINC01426\|ENSG00000234380\|antisense | 0.15344248 | 0.6959695 | 2.18132614 | 1.78E-09 | 7.70E-09 | up |
| RP11-84A19.4\|ENSG00000269967\|lincRNA | 0.15326861 | 1.83498572 | 3.58163466 | 3.89E-06 | 9.98E-06 | up |
| AOAH-IT1\|ENSG00000230539\|sense_intronic | 0.15318545 | 0.83656142 | 2.44919223 | 0.00335175 | 0.00528484 | up |
| AC002331.1\|ENSG00000236481\|lincRNA | 0.15274325 | 1.73340871 | 3.50443132 | 6.14E-12 | 3.98E-11 | up |
| RP11-288L9.4\|ENSG00000225886\|antisense | 0.15273475 | 0.40108338 | 1.39287383 | 0.00410058 | 0.00635167 | up |
| RP11-91P24.5\|ENSG00000254691\|antisense | 0.15255095 | 0.5396343 | 1.82269091 | 0.00276566 | 0.00443593 | up |
| RP11-443B7.3\|ENSG00000258082\|lincRNA | 0.15220968 | 0.32694739 | 1.10299839 | 0.00488613 | 0.00745363 | up |
| GLYCTK-AS1\|ENSG00000242797\|processed_transcript | 0.15171159 | 0.40227943 | 1.40686664 | 0.00705794 | 0.01050369 | up |
| LLNLR-260G6.1\|ENSG00000275162\|sense_intronic | 0.15150944 | 0.58561418 | 1.95054281 | 0.00134816 | 0.0022731 | up |
| PRCAT47\|ENSG00000260896\|lincRNA | 0.15138826 | 0.35110776 | 1.21366055 | 0.01255904 | 0.01794003 | up |
| SCARNA15\|ENSG00000252690\|processed_transcript | 0.15031547 | 0.46946892 | 1.64303622 | 2.25E-10 | 1.11E-09 | up |
| RP11-527F13.1\|ENSG00000231842\|lincRNA | 0.15015637 | 0.9810214 | 2.70781892 | 0.00041036 | 0.00076273 | up |
| C1orf195\|ENSG00000204464\|lincRNA | 0.14959114 | 1.22224737 | 3.0304397 | 0.03215268 | 0.04265975 | up |
| RP13-554M15.8\|ENSG00000277186\|lincRNA | 0.14955169 | 0.37611949 | 1.33054688 | 0.00030279 | 0.00057905 | up |
| RP13-20L14.4\|ENSG00000264812\|sense_intronic | 0.14954808 | 0.35127561 | 1.23199398 | 0.00262833 | 0.00423875 | up |
| WI2-87327B8.2\|ENSG00000273812\|lincRNA | 0.1494653 | 0.96995907 | 2.69811326 | 8.31E-09 | 3.22E-08 | up |
| CTD-3105H18.7\|ENSG00000234848\|antisense | 0.14944103 | 0.47404999 | 1.6654629 | 0.00016765 | 0.00033298 | up |
| RP11-68I3.11\|ENSG00000265625\|sense_intronic | 0.14904761 | 0.57262461 | 1.94181647 | 0.02739697 | 0.03690758 | up |
| RP11-185E8.1\|ENSG00000240497\|lincRNA | 0.14871478 | 0.57223062 | 1.94404868 | 0.01110099 | 0.01597782 | up |
| LINC00412\|ENSG00000234772\|antisense | 0.14832765 | 0.79315149 | 2.41880893 | 7.54E-06 | 1.84E-05 | up |
| RP11-417O11.5\|ENSG00000225761\|sense_intronic | 0.1482524 | 1.07634144 | 2.86000847 | 0.00136494 | 0.00229919 | up |
| RP11-415J8.7\|ENSG00000270115\|lincRNA | 0.14809706 | 0.48442588 | 1.70973298 | 0.00276007 | 0.00442965 | up |
| AC073283.7\|ENSG00000225187\|antisense | 0.14803258 | 1.58288651 | 3.41857122 | 9.14E-15 | 9.25E-14 | up |
| LINC00176\|ENSG00000196421\|lincRNA | 0.14775063 | 0.97008879 | 2.71495253 | 8.89E-12 | 5.56E-11 | up |
| CTC-343N3.1\|ENSG00000183562\|antisense | 0.14746604 | 1.77830095 | 3.59204489 | 2.59E-06 | 6.83E-06 | up |
| AC093690.1\|ENSG00000223522\|antisense | 0.147298 | 0.461048 | 1.64617914 | 0.03420276 | 0.04516912 | up |
| CASK-AS1\|ENSG00000233033\|antisense | 0.14713934 | 0.43502045 | 1.56390023 | 7.78E-05 | 0.00016315 | up |
| RP11-616M22.7\|ENSG00000260710\|lincRNA | 0.14677956 | 1.60408055 | 3.4500236 | 2.22E-10 | 1.09E-09 | up |
| FAM83C-AS1\|ENSG00000235214\|antisense | 0.14639554 | 0.75805417 | 2.37242934 | 2.52E-08 | 9.08E-08 | up |
| RP4-569M23.5\|ENSG00000267882\|antisense | 0.14621493 | 0.49577188 | 1.7615858 | 6.52E-05 | 0.0001379 | up |
| RP11-492E3.51\|ENSG00000239353\|lincRNA | 0.14607602 | 0.53116787 | 1.86244857 | 3.75E-05 | 8.21E-05 | up |
| RP1-224A6.9\|ENSG00000271840\|lincRNA | 0.14589268 | 0.65277302 | 2.16167392 | 0.00291444 | 0.00464506 | up |
| FLJ21408\|ENSG00000245888\|antisense | 0.14579283 | 0.35448436 | 1.28180222 | 2.38E-09 | 1.01E-08 | up |
| AC079145.4\|ENSG00000227210\|antisense | 0.14560878 | 0.88964446 | 2.61113156 | 6.47E-11 | 3.47E-10 | up |
| LINC00618\|ENSG00000225163\|lincRNA | 0.14529961 | 0.95410351 | 2.71511501 | 2.12E-12 | 1.47E-11 | up |
| LINC01270\|ENSG00000203999\|lincRNA | 0.14525504 | 0.53559908 | 1.88256527 | 1.84E-14 | 1.78E-13 | up |
| RP11-380B4.3\|ENSG00000277151\|lincRNA | 0.14482931 | 0.45527598 | 1.65238772 | 0.00075289 | 0.00133081 | up |
| CTD-2165H16.4\|ENSG00000261360\|antisense | 0.14459007 | 0.36136598 | 1.32149224 | 1.11E-10 | 5.72E-10 | up |
| RP11-80H5.7\|ENSG00000240996\|lincRNA | 0.14371314 | 0.35043658 | 1.28596137 | 0.00360061 | 0.00563699 | up |
| RP11-697N18.4\|ENSG00000269924\|sense_intronic | 0.14326761 | 0.5155589 | 1.84742476 | 0.00090318 | 0.00157596 | up |
| RP4-534N18.2\|ENSG00000228634\|lincRNA | 0.14284044 | 1.13879423 | 2.99503072 | 0.00104725 | 0.00180888 | up |
| RP11-738B7.1\|ENSG00000242078\|lincRNA | 0.14277549 | 0.69858388 | 2.29068503 | 2.99E-07 | 9.14E-07 | up |
| RP11-96D1.3\|ENSG00000259797\|sense_intronic | 0.14252628 | 0.46233093 | 1.69769795 | 0.02366351 | 0.03223857 | up |
| RP11-70L8.4\|ENSG00000265194\|antisense | 0.14237749 | 1.13950858 | 3.00061881 | 1.70E-15 | 1.98E-14 | up |
| RP11-754B17.1\|ENSG00000255158\|antisense | 0.14215753 | 0.51899886 | 1.86824086 | 0.00613391 | 0.0092139 | up |
| RP11-138A9.1\|ENSG00000271204\|lincRNA | 0.14142944 | 2.31087581 | 4.03028533 | 1.39E-06 | 3.83E-06 | up |
| C15orf56\|ENSG00000176753\|processed_transcript | 0.14135352 | 0.38260012 | 1.43652949 | 4.13E-08 | 1.45E-07 | up |
| CTB-186G2.4\|ENSG00000267375\|antisense | 0.14095538 | 2.40823762 | 4.09466727 | 0.0005829 | 0.00105256 | up |
| RP11-27M24.2\|ENSG00000260310\|sense_intronic | 0.14057804 | 0.72504992 | 2.36670912 | 0.00231839 | 0.00377566 | up |
| RP11-69L16.4\|ENSG00000238221\|antisense | 0.14019388 | 0.42127672 | 1.58734481 | 0.03099745 | 0.04127543 | up |
| AC004231.2\|ENSG00000234477\|antisense | 0.1400604 | 1.05400558 | 2.91176146 | 2.60E-05 | 5.83E-05 | up |
| CTD-2410N18.4\|ENSG00000273345\|processed_transcript | 0.13942342 | 0.37658211 | 1.43349155 | 9.17E-07 | 2.59E-06 | up |
| LINC01572\|ENSG00000261008\|lincRNA | 0.13921894 | 0.59093972 | 2.08565547 | 3.32E-18 | 6.26E-17 | up |
| RP5-1107A17.3\|ENSG00000262298\|antisense | 0.1390871 | 0.39027837 | 1.48851494 | 2.27E-05 | 5.14E-05 | up |
| RP11-563J2.3\|ENSG00000212743\|lincRNA | 0.13907208 | 0.5312698 | 1.93361189 | 0.00012011 | 0.0002443 | up |
| RP11-133K1.11\|ENSG00000273786\|sense_intronic | 0.13900461 | 0.45497384 | 1.71065086 | 1.73E-06 | 4.70E-06 | up |
| CTD-2358C21.5\|ENSG00000276867\|lincRNA | 0.13880829 | 0.57397077 | 2.04788359 | 8.68E-07 | 2.47E-06 | up |
| SPATA3-AS1\|ENSG00000238062\|lincRNA | 0.13870794 | 0.77160336 | 2.47580907 | 7.37E-18 | 1.32E-16 | up |
| PTGES2-AS1\|ENSG00000232850\|lincRNA | 0.13853422 | 1.33111294 | 3.26431874 | 5.17E-22 | 2.71E-20 | up |
| LINC01545\|ENSG00000204904\|lincRNA | 0.13791899 | 0.44206275 | 1.68043006 | 6.20E-06 | 1.54E-05 | up |
| RP11-394O4.3\|ENSG00000253865\|antisense | 0.13788327 | 0.35182252 | 1.35140039 | 0.00099917 | 0.00173431 | up |
| RP11-752G15.8\|ENSG00000259442\|antisense | 0.1375437 | 0.43342111 | 1.65587939 | 0.0028781 | 0.00459403 | up |
| AC008440.10\|ENSG00000232324\|lincRNA | 0.13746202 | 0.77404921 | 2.49339223 | 3.94E-06 | 1.01E-05 | up |
| RP11-353N14.5\|ENSG00000262585\|lincRNA | 0.13734318 | 1.3087721 | 3.25235678 | 2.56E-20 | 8.53E-19 | up |
| ACTN1-AS1\|ENSG00000259062\|antisense | 0.13724221 | 0.27519327 | 1.00372096 | 6.55E-05 | 0.00013843 | up |
| RP11-313P13.5\|ENSG00000272843\|antisense | 0.13684674 | 0.88572923 | 2.69430466 | 7.91E-09 | 3.07E-08 | up |
| CTC-506B8.1\|ENSG00000249180\|antisense | 0.1368258 | 0.86450257 | 2.65952999 | 0.00291225 | 0.00464436 | up |
| CTC-453G23.5\|ENSG00000269534\|antisense | 0.13675657 | 0.50423384 | 1.88248287 | 6.11E-10 | 2.80E-09 | up |
| RP3-380B8.4\|ENSG00000233064\|lincRNA | 0.13667314 | 0.98083242 | 2.84327695 | 3.37E-06 | 8.73E-06 | up |
| RP11-445O16.3\|ENSG00000224799\|antisense | 0.1357905 | 0.45289969 | 1.73780898 | 2.09E-05 | 4.75E-05 | up |
| RP4-530I15.9\|ENSG00000232043\|antisense | 0.13549047 | 0.65936867 | 2.28289399 | 5.21E-09 | 2.09E-08 | up |
| RP11-212I21.2\|ENSG00000260135\|lincRNA | 0.13534516 | 0.57563759 | 2.08851748 | 0.00010628 | 0.00021812 | up |
| RP11-563J2.2\|ENSG00000215244\|lincRNA | 0.13533953 | 0.63663883 | 2.2338919 | 3.40E-10 | 1.63E-09 | up |
| RP11-502N13.2\|ENSG00000255649\|antisense | 0.1352105 | 0.65502315 | 2.27633876 | 9.11E-05 | 0.000189 | up |
| RP13-463N16.6\|ENSG00000242147\|lincRNA | 0.13449241 | 0.90433162 | 2.74932715 | 3.49E-12 | 2.34E-11 | up |
| FARSA-AS1\|ENSG00000266975\|antisense | 0.13435853 | 0.67459323 | 2.32792994 | 1.63E-05 | 3.77E-05 | up |
| RP11-245C17.2\|ENSG00000259654\|antisense | 0.13369828 | 0.29315095 | 1.13266281 | 0.01924969 | 0.02656667 | up |
| RP11-154J22.1\|ENSG00000259488\|antisense | 0.13369767 | 0.2837838 | 1.08581786 | 1.76E-06 | 4.76E-06 | up |
| RP11-578B16.1\|ENSG00000277223\|sense_intronic | 0.13361117 | 2.9986453 | 4.48819834 | 3.04E-12 | 2.06E-11 | up |
| RP11-616M22.11\|ENSG00000273551\|lincRNA | 0.13325083 | 0.33328544 | 1.32261375 | 9.19E-05 | 0.00019045 | up |
| RP11-386G11.10\|ENSG00000258017\|antisense | 0.13241457 | 0.74486366 | 2.49191446 | 5.01E-14 | 4.53E-13 | up |
| RP11-445P17.8\|ENSG00000224034\|lincRNA | 0.13225226 | 0.48809931 | 1.88388234 | 1.78E-05 | 4.10E-05 | up |
| GTF3C2-AS1\|ENSG00000234945\|antisense | 0.13221623 | 0.42430232 | 1.68219332 | 3.22E-11 | 1.80E-10 | up |
| FRGCA\|ENSG00000236663\|antisense | 0.1318992 | 1.32748208 | 3.33118473 | 1.02E-09 | 4.56E-09 | up |
| RP11-326C3.13\|ENSG00000270030\|lincRNA | 0.13178123 | 0.9737289 | 2.88537522 | 4.60E-10 | 2.15E-09 | up |
| RP11-327F22.6\|ENSG00000270120\|sense_intronic | 0.13167692 | 0.58073451 | 2.14087625 | 8.15E-05 | 0.00017033 | up |
| AC135050.5\|ENSG00000261124\|antisense | 0.13145188 | 0.43526902 | 1.72737254 | 2.49E-06 | 6.57E-06 | up |
| BLACAT1\|ENSG00000281406\|lincRNA | 0.13142645 | 6.17457909 | 5.55401329 | 1.01E-24 | 1.49E-22 | up |
| CTD-2600O9.2\|ENSG00000276166\|antisense | 0.13135586 | 0.39466836 | 1.58716027 | 8.32E-06 | 2.02E-05 | up |
| RP11-108M9.6\|ENSG00000272426\|lincRNA | 0.13130357 | 0.38546375 | 1.55368906 | 0.00047938 | 0.00088083 | up |
| RP5-1158E12.3\|ENSG00000231566\|lincRNA | 0.13116807 | 2.76033919 | 4.39535705 | 6.86E-20 | 1.93E-18 | up |
| CTB-58E17.9\|ENSG00000275665\|antisense | 0.13111358 | 0.42142153 | 1.68444684 | 7.06E-06 | 1.74E-05 | up |
| RP5-836N17.4\|ENSG00000275576\|sense_intronic | 0.13098036 | 1.75676313 | 3.74549723 | 6.44E-15 | 6.77E-14 | up |
| RP11-36I17.2\|ENSG00000275191\|sense_intronic | 0.13004801 | 0.69063571 | 2.40888066 | 7.57E-11 | 4.02E-10 | up |
| AC005534.8\|ENSG00000224903\|antisense | 0.1299527 | 0.82131216 | 2.65994406 | 2.21E-14 | 2.11E-13 | up |
| RP11-333J10.3\|ENSG00000277589\|sense_intronic | 0.12972241 | 0.69794381 | 2.42768317 | 0.0019653 | 0.00323543 | up |
| RP4-533D7.5\|ENSG00000227857\|antisense | 0.12961498 | 0.51500393 | 1.99035096 | 2.32E-05 | 5.26E-05 | up |
| RP11-546K22.1\|ENSG00000253844\|lincRNA | 0.12924997 | 1.96472986 | 3.92609513 | 5.54E-14 | 4.96E-13 | up |
| PRMT5-AS1\|ENSG00000237054\|antisense | 0.12903484 | 0.46285482 | 1.84279911 | 3.30E-16 | 4.37E-15 | up |
| LINC00441\|ENSG00000231473\|lincRNA | 0.12897955 | 0.29835313 | 1.20987861 | 6.47E-05 | 0.00013676 | up |
| RNF144A-AS1\|ENSG00000228203\|processed_transcript | 0.12874355 | 0.38050007 | 1.56339657 | 1.24E-07 | 4.06E-07 | up |
| CTD-2587H19.3\|ENSG00000269873\|lincRNA | 0.12857222 | 0.94910163 | 2.88398366 | 0.00046352 | 0.00085375 | up |
| RP5-1057I20.6\|ENSG00000274737\|sense_intronic | 0.12853573 | 0.44016822 | 1.77588559 | 0.00981596 | 0.01427556 | up |
| CTD-2147F2.1\|ENSG00000259485\|lincRNA | 0.12834209 | 7.94602019 | 5.95216621 | 6.17E-17 | 9.32E-16 | up |
| RP11-407N8.6\|ENSG00000274156\|lincRNA | 0.128088 | 0.43592206 | 1.76693486 | 0.00157295 | 0.00262455 | up |
| RP11-1C8.6\|ENSG00000266289\|lincRNA | 0.12783172 | 0.76646601 | 2.5839759 | 1.77E-11 | 1.03E-10 | up |
| RP11-424N24.2\|ENSG00000271937\|antisense | 0.12782423 | 0.27966511 | 1.12953893 | 0.02570957 | 0.03482024 | up |
| RP11-490B18.5\|ENSG00000276408\|lincRNA | 0.12782253 | 0.3883281 | 1.60313396 | 3.03E-07 | 9.25E-07 | up |
| RP11-598F7.6\|ENSG00000256540\|lincRNA | 0.12777894 | 0.87617508 | 2.77756909 | 1.10E-17 | 1.88E-16 | up |
| RP11-399K21.13\|ENSG00000273248\|lincRNA | 0.12754723 | 0.59981322 | 2.2334817 | 7.22E-07 | 2.09E-06 | up |
| RP11-94I2.4\|ENSG00000274372\|lincRNA | 0.12647759 | 0.4743734 | 1.90714138 | 2.75E-06 | 7.22E-06 | up |
| RP11-493L12.5\|ENSG00000257924\|lincRNA | 0.12593735 | 0.40540665 | 1.68666353 | 0.00014817 | 0.00029695 | up |
| RP1-90G24.6\|ENSG00000224050\|lincRNA | 0.12532404 | 1.01221541 | 3.01378125 | 3.56E-06 | 9.18E-06 | up |
| AC108676.1\|ENSG00000244675\|sense_overlapping | 0.12507585 | 0.73628333 | 2.55745781 | 0.00766503 | 0.01133714 | up |
| PLA2G4C-AS1\|ENSG00000269420\|antisense | 0.12476634 | 0.26431518 | 1.08303052 | 0.01384658 | 0.01959443 | up |
| RP11-473C18.3\|ENSG00000261687\|antisense | 0.12459203 | 0.40039924 | 1.68422745 | 2.39E-05 | 5.40E-05 | up |
| MIR3945HG\|ENSG00000251230\|lincRNA | 0.12397885 | 0.49714707 | 2.00357868 | 0.0033049 | 0.00521873 | up |
| AC097461.4\|ENSG00000234193\|antisense | 0.12397681 | 0.66936256 | 2.43271759 | 1.13E-05 | 2.68E-05 | up |
| RP13-46H24.1\|ENSG00000254739\|antisense | 0.12388844 | 0.79447042 | 2.68095191 | 1.55E-05 | 3.61E-05 | up |
| RP11-474P2.6\|ENSG00000275481\|lincRNA | 0.12369286 | 0.60991709 | 2.30185094 | 6.16E-09 | 2.44E-08 | up |
| CTC-250I14.3\|ENSG00000267512\|antisense | 0.12366707 | 0.3889884 | 1.65326574 | 9.92E-05 | 0.00020455 | up |
| RP13-977J11.8\|ENSG00000256312\|lincRNA | 0.12361599 | 0.48658915 | 1.97683876 | 0.00911361 | 0.01330888 | up |
| CRAT40\|ENSG00000263520\|lincRNA | 0.12283457 | 2.73744006 | 4.47803879 | 3.12E-07 | 9.52E-07 | up |
| AC005307.1\|ENSG00000260725\|lincRNA | 0.1226062 | 1.11262184 | 3.18185955 | 0.00379774 | 0.00592461 | up |
| RP11-147L13.2\|ENSG00000265100\|antisense | 0.1225953 | 0.27785269 | 1.18041654 | 0.00017979 | 0.00035603 | up |
| AC073641.2\|ENSG00000228802\|antisense | 0.12152554 | 0.34279853 | 1.49610134 | 0.00247068 | 0.00400644 | up |
| LINC01269\|ENSG00000258689\|lincRNA | 0.12142427 | 1.10337739 | 3.18379765 | 4.35E-11 | 2.39E-10 | up |
| LINC00323\|ENSG00000226496\|antisense | 0.12102142 | 0.31645797 | 1.38675146 | 0.00026973 | 0.00051995 | up |
| RP5-1186P10.2\|ENSG00000273069\|lincRNA | 0.12101847 | 0.51829118 | 2.09853557 | 2.05E-08 | 7.51E-08 | up |
| RP11-59D5__B.2\|ENSG00000236345\|antisense | 0.12090538 | 1.09746931 | 3.18223022 | 5.34E-15 | 5.67E-14 | up |
| RP11-20G13.3\|ENSG00000259590\|lincRNA | 0.12087199 | 1.02979287 | 3.09080237 | 1.96E-08 | 7.20E-08 | up |
| RP11-34F20.7\|ENSG00000274270\|sense_intronic | 0.12052305 | 0.51197008 | 2.08675044 | 2.07E-18 | 4.12E-17 | up |
| AP000593.7\|ENSG00000255843\|antisense | 0.12034155 | 0.49352453 | 2.03598696 | 4.60E-07 | 1.37E-06 | up |
| CTD-2240E14.4\|ENSG00000267387\|antisense | 0.1203068 | 0.40994514 | 1.76871263 | 4.03E-10 | 1.90E-09 | up |
| RP1-78O14.1\|ENSG00000257894\|lincRNA | 0.12012709 | 0.34895032 | 1.53846012 | 0.01250047 | 0.017866 | up |
| RP11-338K13.1\|ENSG00000273267\|antisense | 0.11999546 | 0.42874176 | 1.83712911 | 0.00033385 | 0.00063183 | up |
| RP3-324O17.7\|ENSG00000274364\|sense_intronic | 0.11996478 | 1.04849693 | 3.12763981 | 8.20E-06 | 1.99E-05 | up |
| RP4-593H12.1\|ENSG00000272854\|antisense | 0.11969255 | 0.30497372 | 1.34935158 | 0.00047156 | 0.00086706 | up |
| RP11-363N22.3\|ENSG00000205740\|processed_transcript | 0.11942199 | 0.2498706 | 1.06511271 | 0.00021788 | 0.00042587 | up |
| RP11-499O7.7\|ENSG00000224251\|antisense | 0.11901631 | 0.44170297 | 1.89191724 | 0.00043988 | 0.00081447 | up |
| CTD-2293H3.2\|ENSG00000267345\|antisense | 0.11894729 | 0.26142209 | 1.13605866 | 0.03437875 | 0.04537417 | up |
| AC144450.1\|ENSG00000228613\|antisense | 0.11885008 | 0.87102695 | 2.87357446 | 0.00033003 | 0.00062503 | up |
| UNQ6494\|ENSG00000237372\|lincRNA | 0.11842507 | 0.3106473 | 1.391303 | 5.60E-07 | 1.64E-06 | up |
| RP11-615I2.2\|ENSG00000260577\|antisense | 0.11783537 | 0.68972392 | 2.5492464 | 3.69E-20 | 1.16E-18 | up |
| RP11-121A14.3\|ENSG00000227200\|sense_intronic | 0.11779288 | 0.70979261 | 2.59114518 | 0.03265043 | 0.04323756 | up |
| AC069513.4\|ENSG00000229178\|lincRNA | 0.11706811 | 0.33339521 | 1.50988526 | 1.05E-07 | 3.47E-07 | up |
| AC118754.4\|ENSG00000229782\|antisense | 0.11628307 | 0.9744406 | 3.06693321 | 6.13E-10 | 2.81E-09 | up |
| RP4-761J14.8\|ENSG00000219410\|antisense | 0.11626605 | 0.26731547 | 1.20111342 | 1.94E-08 | 7.14E-08 | up |
| KB-1440D3.13\|ENSG00000272954\|sense_intronic | 0.11584758 | 0.93596461 | 3.01422612 | 1.66E-11 | 9.78E-11 | up |
| RP11-338N10.3\|ENSG00000269978\|lincRNA | 0.11568084 | 2.53142461 | 4.45172772 | 3.47E-13 | 2.76E-12 | up |
| LINC00624\|ENSG00000278811\|antisense | 0.11517314 | 0.51029963 | 2.14754032 | 3.63E-07 | 1.10E-06 | up |
| RP11-62H20.1\|ENSG00000261120\|antisense | 0.11514178 | 0.56697053 | 2.29986232 | 7.30E-07 | 2.11E-06 | up |
| RDH10-AS1\|ENSG00000250295\|antisense | 0.11513751 | 0.59290281 | 2.36443771 | 7.31E-06 | 1.79E-05 | up |
| RP11-428O18.6\|ENSG00000275294\|lincRNA | 0.11508466 | 0.51586721 | 2.16430414 | 9.62E-07 | 2.71E-06 | up |
| LINC01561\|ENSG00000177234\|lincRNA | 0.1149448 | 0.37131098 | 1.6916868 | 6.57E-05 | 0.00013861 | up |
| AC010524.4\|ENSG00000268157\|antisense | 0.11484701 | 0.53414056 | 2.21750614 | 0.01839061 | 0.02544731 | up |
| RP11-521D12.5\|ENSG00000243491\|lincRNA | 0.11481035 | 0.30649289 | 1.41660095 | 0.00322097 | 0.0050953 | up |
| RP4-655J12.4\|ENSG00000233154\|lincRNA | 0.11476415 | 0.29594249 | 1.36664483 | 3.03E-05 | 6.71E-05 | up |
| RP11-429J17.8\|ENSG00000214733\|antisense | 0.1146487 | 0.86079797 | 2.90845467 | 9.82E-14 | 8.50E-13 | up |
| AC019181.2\|ENSG00000233255\|antisense | 0.11449725 | 0.30804663 | 1.42783586 | 0.00661605 | 0.00989607 | up |
| RP11-469J4.3\|ENSG00000242578\|lincRNA | 0.11438318 | 0.32233788 | 1.49469889 | 0.00050067 | 0.0009152 | up |
| LINC01555\|ENSG00000180869\|lincRNA | 0.11432859 | 1.00980774 | 3.14282251 | 4.04E-13 | 3.17E-12 | up |
| RP11-142C4.6\|ENSG00000255240\|antisense | 0.11417803 | 0.37246812 | 1.70583193 | 0.0038692 | 0.00602191 | up |
| RP11-8L2.1\|ENSG00000250546\|processed_transcript | 0.11401413 | 0.34555953 | 1.59972163 | 0.00127849 | 0.00216457 | up |
| AC009410.1\|ENSG00000232023\|lincRNA | 0.11383231 | 1.85649745 | 4.02760138 | 0.00062072 | 0.00111502 | up |
| AF124730.4\|ENSG00000224649\|antisense | 0.11380288 | 0.65159565 | 2.51743997 | 0.00014496 | 0.00029096 | up |
| RP11-111M22.5\|ENSG00000271757\|lincRNA | 0.11330139 | 0.38996499 | 1.78317909 | 3.57E-06 | 9.20E-06 | up |
| RP11-80H5.2\|ENSG00000232936\|antisense | 0.11323619 | 0.34349214 | 1.60094194 | 3.28E-06 | 8.52E-06 | up |
| TFAP2A-AS1\|ENSG00000229950\|antisense | 0.11310304 | 1.77750451 | 3.97414356 | 1.13E-15 | 1.36E-14 | up |
| RP5-991G20.6\|ENSG00000279206\|lincRNA | 0.11242411 | 0.3194198 | 1.50650226 | 0.00770607 | 0.01138831 | up |
| AC114730.8\|ENSG00000215692\|antisense | 0.11181806 | 0.28768259 | 1.36332475 | 0.00095605 | 0.00166383 | up |
| RP11-484K9.4\|ENSG00000272844\|antisense | 0.11178922 | 0.36745157 | 1.71677302 | 2.06E-06 | 5.51E-06 | up |
| RNASEH2B-AS1\|ENSG00000233672\|antisense | 0.11158388 | 0.40613685 | 1.86383736 | 2.24E-08 | 8.13E-08 | up |
| TSPEAR-AS2\|ENSG00000182912\|antisense | 0.11150962 | 1.04024512 | 3.22168346 | 2.40E-12 | 1.66E-11 | up |
| RP4-539M6.22\|ENSG00000273428\|lincRNA | 0.11128357 | 0.2685658 | 1.27103505 | 0.03276998 | 0.04338503 | up |
| AC068831.16\|ENSG00000278514\|lincRNA | 0.11050881 | 0.2926026 | 1.40478115 | 0.00112817 | 0.0019298 | up |
| CTD-2619J13.19\|ENSG00000269473\|lincRNA | 0.11010368 | 0.34686395 | 1.6555073 | 1.71E-14 | 1.66E-13 | up |
| TTC3-AS1\|ENSG00000228677\|antisense | 0.10993244 | 0.40376909 | 1.87691334 | 0.00031854 | 0.00060587 | up |
| RP11-426C22.4\|ENSG00000259807\|lincRNA | 0.10976625 | 0.98720428 | 3.16891417 | 2.80E-13 | 2.26E-12 | up |
| RP11-443C10.1\|ENSG00000253163\|lincRNA | 0.10937622 | 0.57363839 | 2.39084241 | 1.19E-08 | 4.51E-08 | up |
| RP11-16K12.1\|ENSG00000177699\|antisense | 0.10840863 | 0.26903257 | 1.31130128 | 0.00262763 | 0.00423875 | up |
| RP11-393I2.4\|ENSG00000272008\|antisense | 0.1083922 | 0.23013875 | 1.08624303 | 6.70E-06 | 1.66E-05 | up |
| PLCG1-AS1\|ENSG00000226648\|antisense | 0.10832442 | 0.5419223 | 2.32272749 | 9.53E-10 | 4.26E-09 | up |
| RP13-49I15.6\|ENSG00000273980\|lincRNA | 0.1081855 | 0.28077546 | 1.37590972 | 0.00116596 | 0.00198931 | up |
| AC005307.3\|ENSG00000267243\|lincRNA | 0.10811333 | 0.43208077 | 1.99875664 | 0.02273568 | 0.03103833 | up |
| RP13-650J16.1\|ENSG00000264569\|antisense | 0.10806931 | 0.44902094 | 2.0548259 | 2.39E-05 | 5.41E-05 | up |
| RP11-167J8.3\|ENSG00000254972\|lincRNA | 0.10793818 | 0.35901884 | 1.73385424 | 0.03084708 | 0.04110618 | up |
| RP11-879F14.2\|ENSG00000267279\|lincRNA | 0.10759546 | 0.33744852 | 1.64905017 | 1.76E-09 | 7.64E-09 | up |
| CCDC144NL-AS1\|ENSG00000233098\|antisense | 0.10758068 | 0.32905344 | 1.61290285 | 7.86E-06 | 1.92E-05 | up |
| RP11-158H5.8\|ENSG00000274184\|lincRNA | 0.10753157 | 0.27266303 | 1.34235883 | 0.00441492 | 0.0067915 | up |
| RP11-54O7.11\|ENSG00000224969\|antisense | 0.10751675 | 0.35511941 | 1.7237428 | 0.00030368 | 0.00058012 | up |
| CTC-501O10.1\|ENSG00000267440\|lincRNA | 0.10706303 | 0.35404428 | 1.72546935 | 0.00037017 | 0.00069363 | up |
| RP11-54H7.4\|ENSG00000275216\|lincRNA | 0.10629309 | 7.67656393 | 6.17434103 | 1.09E-18 | 2.34E-17 | up |
| CTD-2357A8.3\|ENSG00000267123\|lincRNA | 0.10622294 | 1.28212307 | 3.59336754 | 2.52E-18 | 4.92E-17 | up |
| RP11-204P2.3\|ENSG00000235399\|antisense | 0.10621865 | 0.35586965 | 1.74431178 | 0.00046648 | 0.00085861 | up |
| RP11-435O5.6\|ENSG00000271314\|antisense | 0.10609338 | 0.80185758 | 2.91801142 | 0.00025572 | 0.00049419 | up |
| RP11-401P9.6\|ENSG00000205414\|antisense | 0.1058322 | 1.29994744 | 3.61860271 | 3.23E-06 | 8.39E-06 | up |
| AC092168.2\|ENSG00000232034\|sense_intronic | 0.10580775 | 1.91232723 | 4.17581225 | 1.09E-07 | 3.60E-07 | up |
| RP11-144F15.1\|ENSG00000257545\|antisense | 0.10566413 | 0.29873233 | 1.49936775 | 0.00291953 | 0.00465176 | up |
| RP4-536B24.4\|ENSG00000260498\|lincRNA | 0.10562152 | 0.36606734 | 1.79320522 | 5.48E-07 | 1.61E-06 | up |
| AC007966.1\|ENSG00000226747\|antisense | 0.1055544 | 0.67502923 | 2.67696324 | 6.69E-07 | 1.94E-06 | up |
| RP5-858B6.3\|ENSG00000223393\|antisense | 0.10538757 | 0.46256656 | 2.13395619 | 5.70E-11 | 3.07E-10 | up |
| RP4-635E18.7\|ENSG00000226849\|antisense | 0.10538261 | 0.25686483 | 1.28537251 | 2.62E-09 | 1.11E-08 | up |
| AC093382.1\|ENSG00000226276\|lincRNA | 0.10523992 | 0.33316734 | 1.66256491 | 0.00532473 | 0.00808548 | up |
| CTC-563A5.2\|ENSG00000249412\|antisense | 0.10503403 | 0.36448041 | 1.79498444 | 0.02921588 | 0.03910943 | up |
| RP11-667M19.2\|ENSG00000256603\|antisense | 0.10503376 | 0.26591121 | 1.34009145 | 0.00992739 | 0.01442178 | up |
| RP11-430G17.3\|ENSG00000271200\|lincRNA | 0.10499512 | 0.52645007 | 2.32597452 | 8.52E-09 | 3.29E-08 | up |
| RP11-158M2.5\|ENSG00000259416\|antisense | 0.10482492 | 0.36064326 | 1.78259074 | 0.00019924 | 0.00039247 | up |
| RP13-516M14.4\|ENSG00000265692\|lincRNA | 0.10476892 | 0.23082854 | 1.13961084 | 2.84E-05 | 6.33E-05 | up |
| RP11-495P10.7\|ENSG00000231196\|processed_transcript | 0.10390168 | 0.35112373 | 1.75676057 | 0.00609625 | 0.00915992 | up |
| RP11-278H7.4\|ENSG00000229960\|lincRNA | 0.10382278 | 1.67851024 | 4.01498641 | 1.61E-16 | 2.25E-15 | up |
| RP4-669H2.1\|ENSG00000231703\|antisense | 0.1037776 | 0.4045692 | 1.96289148 | 0.00274017 | 0.00439905 | up |
| AC079354.5\|ENSG00000231903\|antisense | 0.1033749 | 0.42536072 | 2.04080089 | 7.53E-06 | 1.84E-05 | up |
| RP13-991F5.2\|ENSG00000266445\|antisense | 0.10331215 | 0.29928517 | 1.5345108 | 0.00013599 | 0.00027368 | up |
| CTD-2330K9.2\|ENSG00000230698\|antisense | 0.10324654 | 0.38200129 | 1.88748402 | 5.01E-05 | 0.00010773 | up |
| RP11-700H6.4\|ENSG00000262006\|lincRNA | 0.10304051 | 0.29378139 | 1.51153143 | 0.00225343 | 0.00368259 | up |
| RP11-758N13.1\|ENSG00000259721\|lincRNA | 0.10301909 | 0.42176783 | 2.03353731 | 0.00023188 | 0.00045141 | up |
| Z69666.2\|ENSG00000228779\|antisense | 0.10276964 | 0.46342095 | 2.17290916 | 3.10E-06 | 8.06E-06 | up |
| RP11-106D4.2\|ENSG00000276337\|sense_intronic | 0.10266567 | 0.75547883 | 2.8794375 | 7.96E-19 | 1.76E-17 | up |
| CTC-498J12.1\|ENSG00000250237\|lincRNA | 0.10262985 | 0.49919055 | 2.28214018 | 1.17E-05 | 2.77E-05 | up |
| RP3-486I3.7\|ENSG00000237021\|lincRNA | 0.10252418 | 0.26895458 | 1.39139836 | 0.00045729 | 0.00084316 | up |
| RP11-165D6.1\|ENSG00000278462\|sense_intronic | 0.10229447 | 1.23065275 | 3.58862363 | 0.00056422 | 0.00102254 | up |
| MIRLET7DHG\|ENSG00000230262\|lincRNA | 0.10216546 | 0.21781737 | 1.09221151 | 0.00047483 | 0.00087277 | up |
| RP11-322D14.2\|ENSG00000260145\|antisense | 0.10170314 | 0.79135556 | 2.95996182 | 7.57E-09 | 2.96E-08 | up |
| AC091729.8\|ENSG00000226291\|antisense | 0.10150764 | 0.35951221 | 1.82445245 | 0.00230191 | 0.00375341 | up |
| RP11-425D17.2\|ENSG00000273989\|sense_intronic | 0.10134671 | 0.57830677 | 2.51253572 | 0.00380845 | 0.00593956 | up |
| RP11-23P13.6\|ENSG00000174171\|antisense | 0.1013146 | 2.02239425 | 4.31915021 | 6.97E-16 | 8.63E-15 | up |
| RP11-621L6.3\|ENSG00000273321\|lincRNA | 0.1011481 | 0.27691371 | 1.45296726 | 0.0159458 | 0.02231486 | up |
| RP11-642C5.1\|ENSG00000271156\|lincRNA | 0.10081885 | 0.64357917 | 2.67435227 | 0.0022536 | 0.00368259 | up |
| AC004854.4\|ENSG00000234183\|antisense | 0.10068558 | 0.37902541 | 1.9124375 | 3.48E-05 | 7.67E-05 | up |
| RP11-383G6.3\|ENSG00000244327\|sense_overlapping | 0.10033162 | 0.3533321 | 1.8162485 | 0.00299588 | 0.00476196 | up |
| RP11-613D13.5\|ENSG00000246250\|antisense | 0.10032255 | 0.54090996 | 2.43074256 | 0.00020535 | 0.00040377 | up |
| RP11-108K3.2\|ENSG00000259306\|lincRNA | 0.10014895 | 0.80664157 | 3.00978052 | 3.53E-13 | 2.79E-12 | up |
| LINC01594\|ENSG00000225328\|antisense | 0.09994078 | 2.32299429 | 4.53876829 | 2.07E-16 | 2.82E-15 | up |
| RP11-414H17.5\|ENSG00000213994\|antisense | 0.09985392 | 0.48691682 | 2.28578437 | 0.00353044 | 0.00554514 | up |
| RP11-1M18.1\|ENSG00000231217\|lincRNA | 0.09962743 | 0.5648112 | 2.50315373 | 1.93E-05 | 4.42E-05 | up |
| RP11-302F12.10\|ENSG00000250541\|antisense | 0.0986904 | 0.72666465 | 2.88030815 | 0.01445401 | 0.02037785 | up |
| CTD-2349P21.5\|ENSG00000263603\|lincRNA | 0.09862762 | 0.70898714 | 2.84569587 | 5.08E-09 | 2.05E-08 | up |
| CTC-550B14.6\|ENSG00000267149\|processed_transcript | 0.09862135 | 0.29605543 | 1.58589546 | 0.00021193 | 0.00041547 | up |
| AC009299.2\|ENSG00000235724\|antisense | 0.09845305 | 0.32831363 | 1.73756681 | 9.86E-05 | 0.00020348 | up |
| RP11-390N6.1\|ENSG00000257191\|antisense | 0.09836721 | 1.85424417 | 4.23650988 | 6.70E-12 | 4.28E-11 | up |
| IGFBP7-AS1\|ENSG00000245067\|antisense | 0.09798951 | 0.75182861 | 2.93970461 | 0.00012103 | 0.000246 | up |
| RP5-1120P11.1\|ENSG00000237686\|antisense | 0.09783065 | 2.68511393 | 4.77855297 | 1.27E-25 | 3.36E-23 | up |
| RP11-1143G9.5\|ENSG00000274979\|lincRNA | 0.09748525 | 1.75407469 | 4.16938245 | 3.49E-17 | 5.53E-16 | up |
| LINC00659\|ENSG00000228705\|antisense | 0.09709341 | 8.5799569 | 6.46545315 | 3.77E-24 | 4.54E-22 | up |
| CTD-3224K15.3\|ENSG00000272255\|lincRNA | 0.09682673 | 0.30067378 | 1.6347218 | 0.00419045 | 0.00647437 | up |
| RP11-182J23.1\|ENSG00000260070\|antisense | 0.09675399 | 0.33301901 | 1.78321145 | 1.49E-05 | 3.49E-05 | up |
| RP11-544I20.2\|ENSG00000214770\|antisense | 0.09657711 | 0.27334114 | 1.5009494 | 2.50E-11 | 1.42E-10 | up |
| MIR31HG\|ENSG00000171889\|sense_overlapping | 0.09654131 | 0.94027731 | 3.28386798 | 3.12E-10 | 1.51E-09 | up |
| CTD-2529P6.3\|ENSG00000269300\|antisense | 0.09653007 | 0.32597688 | 1.75571931 | 6.73E-05 | 0.00014182 | up |
| CTD-2587H19.2\|ENSG00000268496\|sense_intronic | 0.09625639 | 0.35590722 | 1.88654701 | 0.01738541 | 0.02416363 | up |
| AC011290.4\|ENSG00000227172\|antisense | 0.09610347 | 0.38538656 | 2.00364579 | 2.18E-05 | 4.95E-05 | up |
| RP11-439M11.1\|ENSG00000271947\|lincRNA | 0.09605256 | 0.30885671 | 1.6850417 | 0.00426798 | 0.00658457 | up |
| BIRC6-AS2\|ENSG00000279897\|antisense | 0.09585984 | 1.26783315 | 3.72529451 | 0.00063081 | 0.00113201 | up |
| RP11-838N2.5\|ENSG00000266578\|lincRNA | 0.0958257 | 0.34202254 | 1.83560691 | 0.01047136 | 0.01515389 | up |
| ADAM20P1\|ENSG00000259158\|lincRNA | 0.09580918 | 0.22168021 | 1.21024419 | 0.00882262 | 0.01291596 | up |
| RP11-551L14.4\|ENSG00000226472\|antisense | 0.0957727 | 0.27489642 | 1.5212018 | 0.00031638 | 0.00060198 | up |
| RP11-734K21.5\|ENSG00000261104\|lincRNA | 0.09571849 | 0.60192043 | 2.65270324 | 1.94E-11 | 1.12E-10 | up |
| RP11-331F9.3\|ENSG00000231393\|antisense | 0.09544542 | 0.30614546 | 1.68146946 | 0.00635207 | 0.00952811 | up |
| EPHA1-AS1\|ENSG00000229153\|antisense | 0.09476511 | 0.32424795 | 1.77466952 | 0.00196166 | 0.00323143 | up |
| RP11-134N1.2\|ENSG00000256084\|lincRNA | 0.09468892 | 1.92329535 | 4.34424096 | 3.73E-05 | 8.17E-05 | up |
| AC092687.5\|ENSG00000234818\|antisense | 0.09456729 | 0.48089643 | 2.34631304 | 0.01617435 | 0.02261678 | up |
| CTD-3064M3.7\|ENSG00000271959\|antisense | 0.09449148 | 0.46899133 | 2.31130504 | 1.28E-10 | 6.51E-10 | up |
| RP11-956J14.1\|ENSG00000237061\|sense_intronic | 0.09446555 | 0.77557124 | 3.03739912 | 0.00074914 | 0.00132505 | up |
| RP11-403P17.2\|ENSG00000260650\|antisense | 0.0943528 | 0.34253424 | 1.86011099 | 0.00833844 | 0.01225453 | up |
| RP1-127H14.3\|ENSG00000255692\|antisense | 0.0940267 | 0.38082825 | 2.01799812 | 0.00056983 | 0.001032 | up |
| LINC00299\|ENSG00000236790\|lincRNA | 0.09371467 | 0.23021445 | 1.29663158 | 2.03E-05 | 4.64E-05 | up |
| CSNK1G2-AS1\|ENSG00000180846\|antisense | 0.09355999 | 0.34668801 | 1.8896743 | 0.00012341 | 0.00024998 | up |
| RP11-508N22.12\|ENSG00000272983\|lincRNA | 0.09354421 | 0.2228937 | 1.25263557 | 0.03141402 | 0.04175389 | up |
| RP11-855A2.3\|ENSG00000266717\|antisense | 0.09336769 | 0.47407796 | 2.34412898 | 0.0002608 | 0.00050382 | up |
| XX-C00717C00720L.1\|ENSG00000279182\|antisense | 0.09295581 | 0.19690252 | 1.08286463 | 0.01677813 | 0.02337473 | up |
| CDIPT-AS1\|ENSG00000214725\|antisense | 0.09259335 | 0.73456433 | 2.98790831 | 1.98E-08 | 7.27E-08 | up |
| AE000662.93\|ENSG00000259054\|lincRNA | 0.09220083 | 0.45474667 | 2.30221143 | 7.73E-05 | 0.00016212 | up |
| RP11-643C9.2\|ENSG00000260679\|lincRNA | 0.09190679 | 0.33766256 | 1.87733888 | 0.0010022 | 0.001739 | up |
| CTD-2349P21.6\|ENSG00000265443\|lincRNA | 0.09169075 | 0.95719399 | 3.38396319 | 3.67E-05 | 8.05E-05 | up |
| RP3-393E18.2\|ENSG00000237927\|lincRNA | 0.09163581 | 0.29142164 | 1.66912462 | 6.03E-05 | 0.00012797 | up |
| RP11-212I21.4\|ENSG00000261997\|lincRNA | 0.09148576 | 0.42214937 | 2.2061345 | 3.34E-05 | 7.37E-05 | up |
| RP11-354K1.2\|ENSG00000228792\|lincRNA | 0.09123983 | 0.39480162 | 2.11339227 | 3.32E-06 | 8.60E-06 | up |
| KB-1562D12.1\|ENSG00000254024\|antisense | 0.09107697 | 0.45426589 | 2.31837875 | 5.71E-05 | 0.00012164 | up |
| AC072062.1\|ENSG00000229267\|antisense | 0.09093512 | 0.2860156 | 1.65318442 | 0.00093523 | 0.00162973 | up |
| LINC01152\|ENSG00000256124\|lincRNA | 0.09015919 | 0.27932592 | 1.63140303 | 0.00277329 | 0.00444548 | up |
| PROX1-AS1\|ENSG00000230461\|processed_transcript | 0.09002395 | 0.43870534 | 2.28487157 | 3.17E-13 | 2.53E-12 | up |
| RP11-2E11.5\|ENSG00000259920\|lincRNA | 0.08994313 | 0.18022067 | 1.00267947 | 0.00066659 | 0.00119137 | up |
| RP4-669P10.16\|ENSG00000232710\|lincRNA | 0.08962468 | 0.37146057 | 2.05124114 | 4.54E-07 | 1.35E-06 | up |
| AC004593.3\|ENSG00000235669\|antisense | 0.08945099 | 2.10787873 | 4.55855068 | 1.14E-12 | 8.33E-12 | up |
| ZFPM2-AS1\|ENSG00000251003\|processed_transcript | 0.08941799 | 0.49066744 | 2.45610855 | 5.81E-09 | 2.31E-08 | up |
| RP4-782L23.2\|ENSG00000226883\|antisense | 0.08926254 | 0.61315416 | 2.780123 | 0.00022827 | 0.00044503 | up |
| RPS6KA2-IT1\|ENSG00000232082\|sense_intronic | 0.08909768 | 0.34799953 | 1.96562564 | 0.00227389 | 0.00371117 | up |
| RP11-429J17.5\|ENSG00000254548\|antisense | 0.08908846 | 2.4180401 | 4.76245582 | 7.49E-18 | 1.33E-16 | up |
| RP11-108O10.2\|ENSG00000254990\|lincRNA | 0.08902247 | 0.21847323 | 1.29521511 | 0.02029419 | 0.02784149 | up |
| LURAP1L-AS1\|ENSG00000235448\|antisense | 0.08857057 | 0.45806294 | 2.3706466 | 0.00010798 | 0.00022134 | up |
| RP3-322G13.7\|ENSG00000234832\|antisense | 0.08843527 | 0.39283557 | 2.15123188 | 1.09E-09 | 4.82E-09 | up |
| AFAP1-AS1\|ENSG00000272620\|antisense | 0.08842112 | 6.52247055 | 6.20488368 | 1.42E-07 | 4.62E-07 | up |
| CTD-2024F21.1\|ENSG00000277173\|sense_intronic | 0.08811036 | 0.36643834 | 2.05618696 | 6.45E-07 | 1.88E-06 | up |
| AL133493.2\|ENSG00000233922\|lincRNA | 0.08803318 | 0.76279486 | 3.11517578 | 9.97E-08 | 3.33E-07 | up |
| CTC-508F8.1\|ENSG00000263331\|antisense | 0.08781411 | 0.9513025 | 3.43737945 | 0.00028129 | 0.00054144 | up |
| RP11-517C16.4\|ENSG00000261243\|antisense | 0.08780622 | 0.34671027 | 1.9813356 | 0.00029233 | 0.00056064 | up |
| RP11-378E13.4\|ENSG00000273965\|antisense | 0.08774116 | 0.26795847 | 1.61068373 | 0.00316243 | 0.00500569 | up |
| KIAA1614-AS1\|ENSG00000232586\|antisense | 0.08739272 | 0.17859142 | 1.0310778 | 0.00487644 | 0.00744099 | up |
| ERVH48-1\|ENSG00000233056\|lincRNA | 0.08733899 | 0.63498253 | 2.86201909 | 0.01963064 | 0.02700806 | up |
| AC078883.4\|ENSG00000226963\|antisense | 0.08693369 | 0.82688031 | 3.24969126 | 0.00050961 | 0.00092897 | up |
| LINC00880\|ENSG00000243629\|lincRNA | 0.08683981 | 0.26590528 | 1.61448396 | 3.49E-12 | 2.34E-11 | up |
| AC004691.5\|ENSG00000229263\|antisense | 0.08681589 | 0.46530319 | 2.42214012 | 0.01624567 | 0.02270454 | up |
| CTD-2369P2.4\|ENSG00000267105\|antisense | 0.08663609 | 1.27317159 | 3.87731489 | 2.12E-11 | 1.22E-10 | up |
| RP5-851M4.1\|ENSG00000277901\|lincRNA | 0.08653978 | 0.37681017 | 2.12240255 | 0.00022914 | 0.0004464 | up |
| RP11-804A23.2\|ENSG00000255959\|antisense | 0.08643146 | 0.25468354 | 1.55907737 | 5.60E-05 | 0.00011959 | up |
| RP11-96K19.4\|ENSG00000261654\|sense_overlapping | 0.08633204 | 0.18592046 | 1.10671757 | 0.0353532 | 0.04654926 | up |
| AC007879.7\|ENSG00000229647\|lincRNA | 0.08597437 | 0.3285906 | 1.93431264 | 4.18E-08 | 1.47E-07 | up |
| RP11-325E5.1\|ENSG00000259656\|lincRNA | 0.0858956 | 0.26993408 | 1.65195093 | 2.06E-05 | 4.71E-05 | up |
| RP11-83B20.1\|ENSG00000214650\|lincRNA | 0.08585944 | 0.46030739 | 2.42254901 | 1.26E-05 | 2.98E-05 | up |
| RP11-320N7.2\|ENSG00000256969\|lincRNA | 0.08573803 | 0.67182768 | 2.97008407 | 0.00094181 | 0.00164013 | up |
| RP11-388M20.6\|ENSG00000260304\|antisense | 0.08511906 | 0.43902929 | 2.366763 | 0.00655167 | 0.00980808 | up |
| RP11-45M22.3\|ENSG00000263624\|antisense | 0.08509679 | 0.39136199 | 2.201327 | 0.00124789 | 0.00211615 | up |
| RP13-516M14.2\|ENSG00000264548\|antisense | 0.08508206 | 0.31978216 | 1.9101626 | 6.35E-12 | 4.09E-11 | up |
| RP11-219B4.3\|ENSG00000254208\|antisense | 0.08469193 | 0.59114559 | 2.80321716 | 5.67E-09 | 2.26E-08 | up |
| AC012360.4\|ENSG00000235319\|lincRNA | 0.08452777 | 0.34331141 | 2.02202052 | 4.39E-07 | 1.31E-06 | up |
| RP11-44K6.4\|ENSG00000254287\|antisense | 0.08438355 | 0.49612996 | 2.5556844 | 0.00017218 | 0.00034134 | up |
| EIF1AX-AS1\|ENSG00000225037\|antisense | 0.08418418 | 1.30569607 | 3.95512611 | 0.00360668 | 0.00564482 | up |
| RP11-419J16.1\|ENSG00000265554\|lincRNA | 0.08412818 | 1.92175923 | 4.51369472 | 1.44E-09 | 6.30E-09 | up |
| AC093732.1\|ENSG00000233845\|antisense | 0.08406372 | 1.6056776 | 4.25555509 | 7.87E-14 | 6.93E-13 | up |
| RP11-452J21.2\|ENSG00000249234\|antisense | 0.08395477 | 0.49780837 | 2.56790621 | 0.01495003 | 0.02100454 | up |
| BANCR\|ENSG00000278910\|lincRNA | 0.08358797 | 0.32939526 | 1.9784526 | 0.00067004 | 0.00119713 | up |
| KCNMB2-AS1\|ENSG00000237978\|antisense | 0.08357768 | 1.68865137 | 4.33660998 | 5.00E-09 | 2.02E-08 | up |
| RP11-734K21.2\|ENSG00000242136\|processed_transcript | 0.08344598 | 0.62095929 | 2.89558427 | 3.76E-09 | 1.54E-08 | up |
| AP000695.4\|ENSG00000233818\|antisense | 0.0834284 | 0.64446852 | 2.94949942 | 1.33E-16 | 1.90E-15 | up |
| IFT74-AS1\|ENSG00000234676\|antisense | 0.08335922 | 0.21717672 | 1.38145577 | 0.02332136 | 0.03179695 | up |
| RP5-994D16.11\|ENSG00000274386\|lincRNA | 0.08334907 | 0.173617 | 1.05867023 | 0.00822169 | 0.01209636 | up |
| RP11-182J1.5\|ENSG00000256278\|antisense | 0.08327028 | 0.24521873 | 1.55819561 | 0.00015115 | 0.00030235 | up |
| LL22NC03-N14H11.1\|ENSG00000272872\|sense_intronic | 0.08318652 | 0.88451692 | 3.4104681 | 4.60E-07 | 1.37E-06 | up |
| C8orf37-AS1\|ENSG00000253773\|lincRNA | 0.08300069 | 0.23228732 | 1.48471515 | 3.79E-05 | 8.28E-05 | up |
| CTB-174O21.2\|ENSG00000269487\|antisense | 0.08283673 | 0.42251414 | 2.35065706 | 1.80E-07 | 5.72E-07 | up |
| RP11-245P10.6\|ENSG00000270104\|lincRNA | 0.08242865 | 0.19336524 | 1.23011074 | 0.02031661 | 0.02785782 | up |
| RP11-234K24.3\|ENSG00000232406\|antisense | 0.08206698 | 0.61776541 | 2.91218527 | 1.55E-07 | 5.01E-07 | up |
| AP000266.7\|ENSG00000232623\|antisense | 0.08152714 | 0.36078653 | 2.1457931 | 0.01260802 | 0.0180003 | up |
| LINC01146\|ENSG00000258867\|lincRNA | 0.08151194 | 0.50948021 | 2.64394282 | 5.75E-08 | 1.99E-07 | up |
| RASGRF2-AS1\|ENSG00000251450\|antisense | 0.08125923 | 0.17578196 | 1.11318346 | 0.00332089 | 0.00524242 | up |
| KB-1507C5.3\|ENSG00000253263\|lincRNA | 0.08088085 | 0.27225995 | 1.75111467 | 0.03720757 | 0.0488332 | up |
| AC137932.6\|ENSG00000261253\|antisense | 0.08063454 | 0.51267904 | 2.6685861 | 2.98E-10 | 1.44E-09 | up |
| PROSER2-AS1\|ENSG00000225778\|antisense | 0.08013997 | 0.17955932 | 1.1638667 | 0.00108769 | 0.0018702 | up |
| RSF1-IT1\|ENSG00000255409\|sense_intronic | 0.08011987 | 0.39145612 | 2.28861859 | 0.01484385 | 0.02086088 | up |
| CTD-2006K23.1\|ENSG00000261222\|lincRNA | 0.07993284 | 0.32885985 | 2.04061267 | 0.0025975 | 0.00419542 | up |
| RP11-176D17.3\|ENSG00000228470\|sense_overlapping | 0.07979248 | 0.33769156 | 2.08138138 | 0.00015366 | 0.00030691 | up |
| RP11-478J18.2\|ENSG00000274895\|antisense | 0.07978454 | 0.45458929 | 2.51038262 | 3.65E-13 | 2.88E-12 | up |
| RP11-256I23.1\|ENSG00000268896\|antisense | 0.07970995 | 0.33741749 | 2.08170298 | 0.00022124 | 0.00043197 | up |
| RP11-351M8.1\|ENSG00000259649\|antisense | 0.0796902 | 0.68012402 | 3.09332364 | 1.60E-05 | 3.71E-05 | up |
| DEPDC1-AS1\|ENSG00000234264\|antisense | 0.0792241 | 0.20613287 | 1.37956326 | 0.0002636 | 0.00050887 | up |
| CTD-2568A17.1\|ENSG00000261341\|lincRNA | 0.07888553 | 0.22295685 | 1.49893195 | 0.01071398 | 0.0154712 | up |
| PLS3-AS1\|ENSG00000271826\|antisense | 0.07882316 | 0.27612236 | 1.80861624 | 1.00E-06 | 2.81E-06 | up |
| CTC-276P9.4\|ENSG00000277619\|antisense | 0.07882169 | 0.16226149 | 1.04165606 | 0.00234319 | 0.00381488 | up |
| RP1-71H24.1\|ENSG00000257452\|antisense | 0.07876967 | 0.39162643 | 2.31376607 | 1.55E-05 | 3.62E-05 | up |
| RP11-528I4.2\|ENSG00000272969\|antisense | 0.07852329 | 0.22204438 | 1.49965564 | 2.16E-05 | 4.91E-05 | up |
| CTD-2024P10.2\|ENSG00000271874\|lincRNA | 0.07838804 | 0.20803201 | 1.40810016 | 0.00918102 | 0.01339994 | up |
| RP11-131N11.4\|ENSG00000254271\|lincRNA | 0.07822807 | 0.2495244 | 1.67342271 | 0.0156321 | 0.02189898 | up |
| CTA-246H3.12\|ENSG00000272942\|lincRNA | 0.07818711 | 0.33425744 | 2.09595707 | 0.00231404 | 0.00377089 | up |
| RP1-41C23.4\|ENSG00000278546\|lincRNA | 0.07811682 | 0.61361946 | 2.97363921 | 2.25E-10 | 1.11E-09 | up |
| RP11-711D18.2\|ENSG00000258435\|lincRNA | 0.07810064 | 0.22892095 | 1.55144324 | 8.71E-05 | 0.00018144 | up |
| RP11-353N14.2\|ENSG00000262772\|lincRNA | 0.07778412 | 1.22166317 | 3.97322705 | 5.44E-22 | 2.80E-20 | up |
| RP11-42O4.2\|ENSG00000278367\|antisense | 0.0777793 | 0.64224002 | 3.04565447 | 3.79E-12 | 2.53E-11 | up |
| RP11-307P5.1\|ENSG00000227681\|lincRNA | 0.07770221 | 0.55894174 | 2.84667042 | 0.00197799 | 0.00325429 | up |
| RP11-677M24.1\|ENSG00000277945\|sense_intronic | 0.07769123 | 0.83762025 | 3.43047268 | 9.65E-07 | 2.72E-06 | up |
| CTD-2619J13.13\|ENSG00000268307\|lincRNA | 0.07759304 | 0.72555957 | 3.225095 | 8.64E-07 | 2.46E-06 | up |
| RP4-545L17.12\|ENSG00000274269\|processed_transcript | 0.07732361 | 0.55166309 | 2.83480665 | 0.0003476 | 0.00065551 | up |
| RP11-17E2.2\|ENSG00000250039\|antisense | 0.0772729 | 0.19007762 | 1.29855431 | 0.00444148 | 0.00682751 | up |
| DLEU7-AS1\|ENSG00000237152\|antisense | 0.07720323 | 0.68356511 | 3.14634561 | 3.12E-15 | 3.44E-14 | up |
| AC011997.1\|ENSG00000222017\|antisense | 0.07691994 | 0.23401104 | 1.60514699 | 0.00240399 | 0.00390667 | up |
| CTC-344H19.4\|ENSG00000268401\|lincRNA | 0.07691777 | 0.34970493 | 2.18474938 | 2.81E-07 | 8.62E-07 | up |
| RP11-338N10.2\|ENSG00000270035\|lincRNA | 0.07667802 | 0.65652244 | 3.09795932 | 2.64E-08 | 9.50E-08 | up |
| RP11-1299A16.3\|ENSG00000260641\|antisense | 0.07667012 | 0.34036395 | 2.15034185 | 8.19E-11 | 4.33E-10 | up |
| LINC01485\|ENSG00000254211\|lincRNA | 0.07640533 | 0.92405644 | 3.59623588 | 2.79E-09 | 1.17E-08 | up |
| RP11-194N12.2\|ENSG00000267222\|sense_intronic | 0.07633685 | 0.31779752 | 2.05765633 | 0.01203059 | 0.0172316 | up |
| AC078852.2\|ENSG00000253399\|sense_intronic | 0.07631432 | 0.49215458 | 2.68908576 | 0.00034454 | 0.00065066 | up |
| DKFZp434J0226\|ENSG00000268460\|processed_transcript | 0.07630856 | 0.16020398 | 1.06999312 | 0.02876914 | 0.0385601 | up |
| RP11-358B23.7\|ENSG00000278829\|sense_intronic | 0.07619352 | 0.54895284 | 2.84894194 | 0.0230509 | 0.03144438 | up |
| AC006273.4\|ENSG00000272473\|lincRNA | 0.07608701 | 0.3016102 | 1.98696319 | 1.39E-07 | 4.54E-07 | up |
| AC078852.1\|ENSG00000254094\|sense_intronic | 0.0759594 | 0.32932205 | 2.11619874 | 0.00027287 | 0.00052561 | up |
| C2-AS1\|ENSG00000281756\|antisense | 0.07586295 | 0.82161695 | 3.43699859 | 3.61E-15 | 3.97E-14 | up |
| RP11-465L10.10\|ENSG00000204044\|antisense | 0.07580843 | 0.41791871 | 2.46279221 | 6.54E-10 | 2.99E-09 | up |
| RP11-346D19.1\|ENSG00000236347\|lincRNA | 0.07517549 | 1.00885465 | 3.74631215 | 1.36E-13 | 1.16E-12 | up |
| RP11-1148O4.2\|ENSG00000265394\|antisense | 0.07486027 | 0.52102531 | 2.79908124 | 0.00060897 | 0.00109504 | up |
| LINC01615\|ENSG00000223485\|lincRNA | 0.07474633 | 0.92530777 | 3.62985864 | 4.57E-17 | 7.07E-16 | up |
| AC073257.2\|ENSG00000237614\|lincRNA | 0.07471693 | 0.44485234 | 2.57381953 | 1.44E-07 | 4.68E-07 | up |
| IGBP1-AS2\|ENSG00000220925\|antisense | 0.07442042 | 0.43750805 | 2.55553903 | 1.87E-05 | 4.30E-05 | up |
| RP11-145A3.1\|ENSG00000227496\|antisense | 0.07409964 | 0.243384 | 1.71569599 | 0.00087386 | 0.00152782 | up |
| RP11-956E11.1\|ENSG00000257507\|lincRNA | 0.07393329 | 0.19391211 | 1.39110689 | 0.01457992 | 0.02052806 | up |
| RP4-616B8.6\|ENSG00000277581\|sense_intronic | 0.07374303 | 0.49366969 | 2.74296757 | 2.07E-09 | 8.85E-09 | up |
| LINC01191\|ENSG00000234199\|lincRNA | 0.07368345 | 0.21208071 | 1.52520089 | 0.00032359 | 0.00061439 | up |
| CTC-455F18.1\|ENSG00000253348\|antisense | 0.07334484 | 0.7982199 | 3.44401888 | 0.00135111 | 0.00227661 | up |
| RP11-709D24.6\|ENSG00000261442\|antisense | 0.07316721 | 0.23164403 | 1.66264039 | 0.00235886 | 0.00383921 | up |
| RP1-69D17.4\|ENSG00000226149\|lincRNA | 0.07284367 | 0.5440722 | 2.90092258 | 0.00086636 | 0.00151669 | up |
| RP11-54O7.16\|ENSG00000272438\|lincRNA | 0.07282593 | 0.5242088 | 2.84761736 | 0.00236198 | 0.00384312 | up |
| CTB-43E15.2\|ENSG00000253428\|lincRNA | 0.07243016 | 0.60666112 | 3.06622848 | 6.02E-10 | 2.77E-09 | up |
| XXbac-BPG308K3.5\|ENSG00000225173\|lincRNA | 0.0723555 | 0.73659102 | 3.34768928 | 3.13E-13 | 2.51E-12 | up |
| RP11-700H6.2\|ENSG00000251665\|lincRNA | 0.0719553 | 0.18551678 | 1.36637693 | 0.00013014 | 0.00026254 | up |
| PTPRG-AS1\|ENSG00000241472\|processed_transcript | 0.07186289 | 0.3083806 | 2.10139323 | 0.00065457 | 0.00117106 | up |
| RP11-392O1.4\|ENSG00000267747\|antisense | 0.07184437 | 0.99309042 | 3.78897814 | 1.72E-12 | 1.22E-11 | up |
| DENND5B-AS1\|ENSG00000255867\|antisense | 0.07163066 | 0.30268522 | 2.07916906 | 0.01973988 | 0.02715129 | up |
| AC010148.1\|ENSG00000235726\|processed_transcript | 0.07153452 | 0.28647163 | 2.00168081 | 1.03E-11 | 6.35E-11 | up |
| CDC42-IT1\|ENSG00000230068\|sense_intronic | 0.07144771 | 0.70393606 | 3.30048466 | 0.0005348 | 0.00097289 | up |
| RP11-49O14.2\|ENSG00000236095\|sense_intronic | 0.07126542 | 0.68570925 | 3.26632282 | 7.58E-06 | 1.85E-05 | up |
| RP11-402G3.5\|ENSG00000230054\|lincRNA | 0.07117694 | 0.171683 | 1.27026539 | 0.01343729 | 0.01909168 | up |
| CTB-60B18.10\|ENSG00000268655\|lincRNA | 0.07105901 | 0.33079376 | 2.21884262 | 0.0001632 | 0.00032486 | up |
| RP5-963E22.4\|ENSG00000231977\|lincRNA | 0.07099798 | 0.26751678 | 1.91377957 | 0.01391207 | 0.0196661 | up |
| LACTB2-AS1\|ENSG00000246366\|antisense | 0.07097772 | 0.24833288 | 1.80683724 | 2.04E-08 | 7.47E-08 | up |
| RP11-363N22.2\|ENSG00000229869\|antisense | 0.07083245 | 0.31364627 | 2.14665602 | 0.00012772 | 0.00025822 | up |
| RP11-540O11.4\|ENSG00000259521\|antisense | 0.07066103 | 0.31337135 | 2.14888656 | 0.00190046 | 0.00313452 | up |
| PCAT1\|ENSG00000253438\|lincRNA | 0.07060098 | 0.40827626 | 2.53178563 | 2.02E-16 | 2.76E-15 | up |
| RP11-436D10.3\|ENSG00000238280\|antisense | 0.07045607 | 0.16966328 | 1.26787854 | 0.01923508 | 0.02655342 | up |
| LA16c-352F7.1\|ENSG00000278716\|lincRNA | 0.07016551 | 0.68927425 | 3.29624415 | 1.38E-06 | 3.80E-06 | up |
| RP4-680D5.2\|ENSG00000237301\|antisense | 0.06988082 | 0.15688328 | 1.16672308 | 3.47E-05 | 7.64E-05 | up |
| RP11-431K24.1\|ENSG00000238290\|lincRNA | 0.06972586 | 0.24674192 | 1.82323715 | 4.05E-09 | 1.66E-08 | up |
| RP4-541C22.5\|ENSG00000255202\|antisense | 0.06972309 | 0.18144406 | 1.37981646 | 0.03806838 | 0.04987654 | up |
| RP11-861E21.2\|ENSG00000267199\|antisense | 0.06970499 | 0.23680898 | 1.76438991 | 2.41E-08 | 8.70E-08 | up |
| DUXAP8\|ENSG00000206195\|processed_transcript | 0.06958391 | 1.12720472 | 4.01785192 | 5.60E-16 | 7.05E-15 | up |
| RP3-439F8.1\|ENSG00000234869\|antisense | 0.06953917 | 0.32510846 | 2.2250234 | 1.94E-14 | 1.86E-13 | up |
| RP11-276E17.2\|ENSG00000234142\|lincRNA | 0.06950538 | 0.2569824 | 1.886473 | 0.00015511 | 0.00030969 | up |
| RP11-436K8.1\|ENSG00000231252\|lincRNA | 0.06930489 | 1.03733867 | 3.90378611 | 4.74E-14 | 4.30E-13 | up |
| RP11-91I20.2\|ENSG00000254869\|lincRNA | 0.06922866 | 0.34457106 | 2.31536012 | 6.17E-06 | 1.53E-05 | up |
| AC007405.4\|ENSG00000234350\|lincRNA | 0.06897178 | 0.31100844 | 2.17287561 | 4.97E-13 | 3.86E-12 | up |
| RP11-177H13.2\|ENSG00000253837\|processed_transcript | 0.0687628 | 0.28283743 | 2.04027296 | 1.72E-07 | 5.49E-07 | up |
| RP11-452H21.1\|ENSG00000254420\|antisense | 0.06867862 | 0.50427222 | 2.87626985 | 5.88E-07 | 1.72E-06 | up |
| RP1-276N6.2\|ENSG00000230234\|lincRNA | 0.06866995 | 5.6146779 | 6.3533806 | 2.32E-16 | 3.14E-15 | up |
| AP000253.1\|ENSG00000234509\|lincRNA | 0.06860142 | 0.14389468 | 1.06870301 | 0.00512845 | 0.0077986 | up |
| RP11-535A19.2\|ENSG00000255507\|antisense | 0.06858115 | 0.28108508 | 2.03512297 | 5.19E-12 | 3.41E-11 | up |
| HOTAIR\|ENSG00000228630\|antisense | 0.0682835 | 0.85556046 | 3.64726096 | 0.00124472 | 0.00211212 | up |
| RP11-44N11.1\|ENSG00000253372\|sense_intronic | 0.06827271 | 0.6649759 | 3.28392111 | 0.0142904 | 0.02017146 | up |
| RP11-303E16.6\|ENSG00000261838\|antisense | 0.06821118 | 0.51510743 | 2.91679329 | 0.00011702 | 0.00023841 | up |
| LINC01285\|ENSG00000203650\|lincRNA | 0.06780334 | 0.21075744 | 1.63615536 | 5.18E-06 | 1.30E-05 | up |
| CTD-2529O21.2\|ENSG00000261978\|antisense | 0.06778965 | 0.34883028 | 2.36338832 | 0.00110882 | 0.00190037 | up |
| RP1-90G24.11\|ENSG00000273325\|lincRNA | 0.06763314 | 0.67731071 | 3.32401565 | 3.53E-06 | 9.11E-06 | up |
| LINC00941\|ENSG00000235884\|lincRNA | 0.06759747 | 1.90279336 | 4.81500583 | 1.26E-15 | 1.50E-14 | up |
| RP11-151A6.4\|ENSG00000224356\|sense_intronic | 0.0675818 | 0.24107043 | 1.83474801 | 3.83E-08 | 1.36E-07 | up |
| RP11-583F2.7\|ENSG00000280299\|processed_transcript | 0.06739392 | 0.28063828 | 2.05802159 | 3.61E-05 | 7.94E-05 | up |
| RP11-659E9.2\|ENSG00000240915\|antisense | 0.06729401 | 0.21144468 | 1.65173027 | 0.01697663 | 0.02363264 | up |
| CTC-348L5.1\|ENSG00000253687\|antisense | 0.06721766 | 0.95212584 | 3.8242401 | 9.25E-08 | 3.10E-07 | up |
| AC005592.2\|ENSG00000231185\|antisense | 0.06684462 | 0.70398642 | 3.39666419 | 3.63E-23 | 2.64E-21 | up |
| RP11-381N20.1\|ENSG00000251526\|lincRNA | 0.06681655 | 0.2381376 | 1.83351811 | 0.00298254 | 0.00474219 | up |
| RP11-33B1.3\|ENSG00000250950\|lincRNA | 0.06676689 | 0.31841406 | 2.25369924 | 0.00248087 | 0.00402051 | up |
| RP13-16H11.5\|ENSG00000235843\|antisense | 0.06672752 | 0.341262 | 2.35452594 | 0.00482862 | 0.00737439 | up |
| FAM157C\|ENSG00000260528\|lincRNA | 0.06654983 | 0.19645611 | 1.56170018 | 6.49E-09 | 2.56E-08 | up |
| GATM-AS1\|ENSG00000275672\|antisense | 0.06642542 | 0.14958334 | 1.17114219 | 0.01665828 | 0.02321997 | up |
| RP11-462G2.2\|ENSG00000224349\|antisense | 0.06619323 | 0.26821895 | 2.0186556 | 0.00036082 | 0.00067729 | up |
| DSCR9\|ENSG00000230366\|lincRNA | 0.06610348 | 0.25175607 | 1.9292284 | 1.06E-10 | 5.45E-10 | up |
| RP11-486M23.1\|ENSG00000253632\|lincRNA | 0.0660937 | 0.27169881 | 2.03942355 | 0.00158147 | 0.00263795 | up |
| CTD-2049O4.1\|ENSG00000261329\|antisense | 0.06608981 | 0.2956101 | 2.16119589 | 0.00020271 | 0.00039887 | up |
| CTB-35F21.1\|ENSG00000249526\|lincRNA | 0.06585314 | 0.37153577 | 2.49617707 | 6.56E-05 | 0.00013847 | up |
| RP11-282K24.3\|ENSG00000253284\|sense_intronic | 0.06537522 | 0.69346992 | 3.4070176 | 0.00299717 | 0.00476259 | up |
| RP11-362F19.1\|ENSG00000248810\|lincRNA | 0.065257 | 1.86175497 | 4.83438672 | 0.00101964 | 0.00176637 | up |
| RP5-837J1.4\|ENSG00000267457\|lincRNA | 0.06505109 | 0.82922336 | 3.6721156 | 0.00022666 | 0.00044205 | up |
| RP11-686O6.1\|ENSG00000272966\|lincRNA | 0.06476437 | 0.33039864 | 2.35093554 | 2.72E-05 | 6.08E-05 | up |
| RP13-895J2.2\|ENSG00000256943\|antisense | 0.06468119 | 0.2824465 | 2.12655944 | 0.00090728 | 0.00158259 | up |
| AC009120.11\|ENSG00000275236\|lincRNA | 0.06466792 | 0.22526925 | 1.80052828 | 6.75E-06 | 1.67E-05 | up |
| RP11-10A14.9\|ENSG00000279949\|lincRNA | 0.06447108 | 0.57588579 | 3.15905862 | 3.40E-09 | 1.41E-08 | up |
| RP11-14D22.1\|ENSG00000244564\|lincRNA | 0.06444795 | 0.32775929 | 2.34643039 | 0.00050425 | 0.00092078 | up |
| RP11-84A19.3\|ENSG00000254545\|antisense | 0.06430133 | 0.31800711 | 2.30613864 | 4.68E-05 | 0.00010118 | up |
| RP11-63A1.1\|ENSG00000261886\|lincRNA | 0.06415585 | 0.31409679 | 2.29155651 | 9.34E-06 | 2.25E-05 | up |
| RP11-192H23.7\|ENSG00000264044\|antisense | 0.06401353 | 0.38110118 | 2.57372524 | 1.26E-08 | 4.76E-08 | up |
| WASIR2\|ENSG00000231439\|lincRNA | 0.06395211 | 0.56587186 | 3.14541154 | 1.75E-09 | 7.60E-09 | up |
| LINC00958\|ENSG00000251381\|lincRNA | 0.06367789 | 0.44875417 | 2.81706103 | 3.96E-07 | 1.19E-06 | up |
| AC007461.2\|ENSG00000226101\|lincRNA | 0.06340579 | 0.22755742 | 1.84354408 | 0.00030327 | 0.00057954 | up |
| AC022819.3\|ENSG00000259921\|sense_intronic | 0.06327987 | 0.14110892 | 1.15699061 | 0.00126359 | 0.00214139 | up |
| AC123023.1\|ENSG00000236452\|lincRNA | 0.06327246 | 5.70219715 | 6.49379639 | 4.28E-20 | 1.29E-18 | up |
| CH507-154B10.2\|ENSG00000280018\|lincRNA | 0.06320677 | 0.12763218 | 1.0138412 | 0.01505399 | 0.02113379 | up |
| DIAPH2-AS1\|ENSG00000236256\|antisense | 0.06308759 | 0.81506293 | 3.69148324 | 9.09E-10 | 4.09E-09 | up |
| RP1-90J4.1\|ENSG00000257906\|lincRNA | 0.06305095 | 0.48523958 | 2.94410718 | 2.43E-07 | 7.56E-07 | up |
| LUCAT1\|ENSG00000248323\|lincRNA | 0.06295538 | 1.0773818 | 4.09705606 | 1.53E-15 | 1.81E-14 | up |
| LINC00240\|ENSG00000224843\|lincRNA | 0.06285568 | 0.1508933 | 1.26341381 | 6.10E-06 | 1.52E-05 | up |
| RP11-151A6.6\|ENSG00000280169\|antisense | 0.06285422 | 0.19942164 | 1.66574053 | 0.00795377 | 0.01172822 | up |
| CTD-2008P7.10\|ENSG00000267644\|lincRNA | 0.06284258 | 0.25910604 | 2.04372827 | 0.00030875 | 0.00058874 | up |
| CTC-529G1.1\|ENSG00000254391\|antisense | 0.0628151 | 0.23068539 | 1.87674341 | 0.00646555 | 0.00969285 | up |
| KCNQ1OT1\|ENSG00000269821\|antisense | 0.06240384 | 0.67619979 | 3.43774292 | 1.17E-16 | 1.69E-15 | up |
| RP11-44N22.3\|ENSG00000272702\|processed_transcript | 0.06227465 | 0.32854035 | 2.39935369 | 1.68E-16 | 2.34E-15 | up |
| RP11-697E22.2\|ENSG00000277501\|antisense | 0.06227066 | 0.15469011 | 1.31275642 | 0.00926097 | 0.01350548 | up |
| RP11-123B3.2\|ENSG00000235939\|antisense | 0.06201298 | 0.4679168 | 2.91560995 | 1.16E-06 | 3.22E-06 | up |
| RP11-319G9.3\|ENSG00000260816\|sense_intronic | 0.06192722 | 0.37295414 | 2.59035263 | 0.0038867 | 0.00604737 | up |
| AC083884.8\|ENSG00000232729\|processed_transcript | 0.06171031 | 0.75708499 | 3.6168719 | 2.85E-08 | 1.02E-07 | up |
| RP11-2E11.6\|ENSG00000270823\|antisense | 0.06161202 | 0.26750861 | 2.11830171 | 2.33E-09 | 9.91E-09 | up |
| RP11-184M15.1\|ENSG00000248187\|lincRNA | 0.0614956 | 0.18430197 | 1.58351639 | 0.00140388 | 0.00235954 | up |
| RP11-620J15.2\|ENSG00000245651\|lincRNA | 0.0612517 | 0.14358326 | 1.22906565 | 0.00364812 | 0.00570294 | up |
| RP11-493L12.3\|ENSG00000257925\|antisense | 0.06098245 | 0.59292079 | 3.28137332 | 8.42E-09 | 3.26E-08 | up |
| LINC00867\|ENSG00000232139\|lincRNA | 0.0606515 | 0.29736312 | 2.29361041 | 0.00024543 | 0.00047587 | up |
| AC002401.1\|ENSG00000236472\|antisense | 0.06061587 | 0.24243518 | 1.99983156 | 0.00254933 | 0.00412264 | up |
| AC004549.6\|ENSG00000229893\|antisense | 0.06048549 | 0.18302602 | 1.59738778 | 0.00012836 | 0.00025921 | up |
| EXTL3-AS1\|ENSG00000246339\|antisense | 0.06046283 | 0.32087584 | 2.40789465 | 6.56E-15 | 6.88E-14 | up |
| RP11-79O8.1\|ENSG00000271991\|lincRNA | 0.06043474 | 0.24406917 | 2.01384011 | 1.51E-05 | 3.53E-05 | up |
| RP11-643M14.1\|ENSG00000259251\|antisense | 0.06032042 | 0.17184033 | 1.51035025 | 2.02E-05 | 4.60E-05 | up |
| RP11-680H20.2\|ENSG00000250519\|lincRNA | 0.06027322 | 0.25926158 | 2.10481948 | 0.02746372 | 0.03698811 | up |
| LMCD1-AS1\|ENSG00000227110\|antisense | 0.06014147 | 0.22598873 | 1.90981872 | 1.02E-14 | 1.02E-13 | up |
| KIZ-AS1\|ENSG00000232712\|antisense | 0.05982065 | 0.22780075 | 1.92905706 | 0.0163391 | 0.02281706 | up |
| AC159540.1\|ENSG00000230606\|lincRNA | 0.05968058 | 0.15050559 | 1.3344837 | 0.00061517 | 0.00110581 | up |
| EHMT1-IT1\|ENSG00000281796\|sense_intronic | 0.05967409 | 0.34299712 | 2.52301999 | 0.0018337 | 0.003031 | up |
| RP11-127L20.3\|ENSG00000228261\|lincRNA | 0.05957278 | 0.5927965 | 3.31481174 | 8.71E-08 | 2.93E-07 | up |
| AC005042.4\|ENSG00000204380\|antisense | 0.05914739 | 0.28216251 | 2.25413994 | 1.17E-11 | 7.17E-11 | up |
| RP11-95H3.1\|ENSG00000259847\|lincRNA | 0.05913513 | 0.2065883 | 1.8046711 | 0.00119576 | 0.00203621 | up |
| RP11-1055B8.2\|ENSG00000263154\|lincRNA | 0.05860975 | 1.00653058 | 4.10210643 | 3.17E-07 | 9.66E-07 | up |
| AC006460.2\|ENSG00000228509\|antisense | 0.0584964 | 0.52324813 | 3.16107547 | 0.01215582 | 0.01740157 | up |
| RP11-1012E15.2\|ENSG00000259868\|processed_transcript | 0.05843437 | 0.1777387 | 1.60486883 | 0.00057549 | 0.00104047 | up |
| RP11-45L9.1\|ENSG00000251175\|antisense | 0.05784956 | 0.47898915 | 3.04961517 | 0.00038436 | 0.00071792 | up |
| AC104809.4\|ENSG00000233392\|processed_transcript | 0.05783562 | 0.83108508 | 3.84496587 | 6.86E-05 | 0.00014449 | up |
| AJ006998.2\|ENSG00000229425\|lincRNA | 0.05763327 | 0.15086282 | 1.38826361 | 0.01227708 | 0.01755619 | up |
| LINC01136\|ENSG00000233791\|lincRNA | 0.05727375 | 0.12737982 | 1.15319087 | 0.0003265 | 0.00061925 | up |
| CTA-390C10.9\|ENSG00000272798\|lincRNA | 0.05725631 | 0.34002955 | 2.57015362 | 5.55E-05 | 0.00011856 | up |
| THOC7-AS1\|ENSG00000240549\|antisense | 0.05718336 | 0.4663941 | 3.02788212 | 0.02295365 | 0.03131977 | up |
| RP11-456D7.1\|ENSG00000263787\|antisense | 0.05717487 | 0.33016146 | 2.52971852 | 0.00016468 | 0.00032758 | up |
| RP11-321P16.1\|ENSG00000276923\|lincRNA | 0.05707085 | 0.30319646 | 2.40942703 | 5.55E-05 | 0.00011856 | up |
| RP11-659E9.4\|ENSG00000253121\|antisense | 0.05702485 | 0.50229964 | 3.13888547 | 6.12E-06 | 1.52E-05 | up |
| RP11-114F3.4\|ENSG00000277895\|antisense | 0.05662969 | 0.26553163 | 2.2292532 | 5.57E-10 | 2.57E-09 | up |
| RP11-383I23.2\|ENSG00000273374\|lincRNA | 0.05651618 | 0.37236676 | 2.71998834 | 2.03E-13 | 1.67E-12 | up |
| CTB-193M12.3\|ENSG00000262380\|sense_intronic | 0.05644767 | 0.35745986 | 2.66279546 | 0.00119175 | 0.00203134 | up |
| RP11-278L15.2\|ENSG00000243885\|lincRNA | 0.05633942 | 0.37534393 | 2.73599666 | 7.07E-10 | 3.22E-09 | up |
| RP11-229P13.15\|ENSG00000236394\|antisense | 0.05601175 | 0.37622532 | 2.74779558 | 2.33E-08 | 8.44E-08 | up |
| AC005757.6\|ENSG00000267044\|antisense | 0.05597309 | 0.3855442 | 2.78409109 | 0.003856 | 0.00600489 | up |
| AC007204.2\|ENSG00000267268\|lincRNA | 0.05593167 | 0.12990951 | 1.2157698 | 0.02784223 | 0.03744081 | up |
| AC004156.3\|ENSG00000267666\|lincRNA | 0.0558452 | 0.12144058 | 1.12074529 | 0.00277529 | 0.00444734 | up |
| CTD-3032J10.3\|ENSG00000269480\|antisense | 0.05576087 | 0.17252908 | 1.62951452 | 0.00116551 | 0.00198917 | up |
| RP11-540O11.6\|ENSG00000259617\|antisense | 0.05558457 | 0.60856194 | 3.45264782 | 0.00123409 | 0.00209542 | up |
| RP11-181B11.1\|ENSG00000254370\|sense_intronic | 0.05531494 | 0.21405452 | 1.95223718 | 0.00761897 | 0.01127846 | up |
| RP1-90G24.10\|ENSG00000242082\|antisense | 0.05530243 | 0.4504357 | 3.02590634 | 7.84E-07 | 2.25E-06 | up |
| AC002306.1\|ENSG00000259242\|antisense | 0.05516629 | 0.26885767 | 2.28498386 | 0.00288222 | 0.00459924 | up |
| AF127577.8\|ENSG00000235277\|antisense | 0.05452901 | 1.56880023 | 4.846494 | 1.68E-07 | 5.37E-07 | up |
| RP11-727A23.7\|ENSG00000254551\|antisense | 0.0544204 | 0.14945348 | 1.45747707 | 0.02111632 | 0.02890204 | up |
| RP11-401O9.4\|ENSG00000273388\|antisense | 0.05431254 | 0.296443 | 2.44839756 | 0.00050773 | 0.0009265 | up |
| RP11-417E7.2\|ENSG00000261039\|lincRNA | 0.05426747 | 1.01908828 | 4.23104756 | 3.96E-08 | 1.40E-07 | up |
| ISM1-AS1\|ENSG00000226263\|antisense | 0.0542572 | 0.27867578 | 2.36070104 | 0.0009142 | 0.00159361 | up |
| CTA-339C12.1\|ENSG00000259294\|antisense | 0.05402457 | 0.69048448 | 3.6759213 | 4.36E-07 | 1.30E-06 | up |
| RP11-353N14.1\|ENSG00000262768\|antisense | 0.05389246 | 1.25077452 | 4.53659459 | 1.54E-16 | 2.16E-15 | up |
| RP11-265N7.1\|ENSG00000259450\|lincRNA | 0.05366665 | 0.25194402 | 2.23100536 | 4.91E-05 | 0.00010564 | up |
| HSD52\|ENSG00000224609\|processed_transcript | 0.05361833 | 0.45701308 | 3.09143717 | 0.00479667 | 0.00732982 | up |
| TERC\|ENSG00000270141\|lincRNA | 0.05357196 | 0.591546 | 3.46494051 | 0.0006075 | 0.00109276 | up |
| RP5-1029F21.2\|ENSG00000262905\|antisense | 0.05355141 | 0.39140609 | 2.86966966 | 3.12E-06 | 8.12E-06 | up |
| RP11-85G18.6\|ENSG00000262412\|lincRNA | 0.05338646 | 0.13062061 | 1.2908368 | 0.01332148 | 0.01894238 | up |
| RP11-25H12.1\|ENSG00000249413\|lincRNA | 0.05309067 | 0.44156926 | 3.05610957 | 3.58E-05 | 7.87E-05 | up |
| RP5-892K4.1\|ENSG00000271806\|antisense | 0.05306154 | 0.14845662 | 1.48430308 | 0.00843529 | 0.0123797 | up |
| RP11-68I3.7\|ENSG00000264647\|antisense | 0.05288145 | 0.65061845 | 3.62097814 | 1.93E-09 | 8.26E-09 | up |
| GS1-600G8.5\|ENSG00000235385\|lincRNA | 0.05266476 | 0.3918098 | 2.89524357 | 4.67E-09 | 1.90E-08 | up |
| RP11-615I2.1\|ENSG00000260084\|antisense | 0.0526007 | 0.19060125 | 1.8574038 | 0.00564223 | 0.00853318 | up |
| RP11-517H2.6\|ENSG00000272980\|processed_transcript | 0.05256472 | 0.20558047 | 1.96753655 | 0.00044782 | 0.00082657 | up |
| CTD-2256P15.1\|ENSG00000248968\|antisense | 0.05230252 | 0.3162461 | 2.59609539 | 2.28E-10 | 1.12E-09 | up |
| CTD-2529O21.1\|ENSG00000262172\|sense_intronic | 0.05219199 | 1.2539855 | 4.58654841 | 2.91E-19 | 7.01E-18 | up |
| RP11-219B4.7\|ENSG00000260493\|antisense | 0.05217581 | 0.19702345 | 1.91691445 | 2.51E-06 | 6.64E-06 | up |
| SMARCA5-AS1\|ENSG00000245112\|antisense | 0.05216198 | 0.17624271 | 1.75649302 | 0.00157195 | 0.00262371 | up |
| EGOT\|ENSG00000235947\|lincRNA | 0.05203045 | 0.43879352 | 3.07611405 | 8.82E-09 | 3.40E-08 | up |
| RP11-181K12.2\|ENSG00000251687\|antisense | 0.05177689 | 0.35577046 | 2.78056648 | 5.97E-06 | 1.49E-05 | up |
| CTA-315H11.2\|ENSG00000274080\|sense_intronic | 0.05173247 | 1.26459226 | 4.61145855 | 2.09E-22 | 1.19E-20 | up |
| CTB-46B19.2\|ENSG00000249593\|antisense | 0.05150055 | 0.39287192 | 2.93139943 | 2.76E-06 | 7.25E-06 | up |
| LINC01549\|ENSG00000232560\|lincRNA | 0.05102956 | 0.73428695 | 3.84693881 | 2.98E-06 | 7.77E-06 | up |
| EPN2-AS1\|ENSG00000235397\|antisense | 0.05098823 | 0.37552903 | 2.88068841 | 0.00260359 | 0.00420269 | up |
| AC073326.3\|ENSG00000228540\|sense_intronic | 0.05069012 | 0.58285051 | 3.5233494 | 4.48E-06 | 1.14E-05 | up |
| L29074.3\|ENSG00000227083\|antisense | 0.05068753 | 0.26899598 | 2.40788187 | 0.00014723 | 0.00029524 | up |
| LA16c-444G7.2\|ENSG00000260420\|lincRNA | 0.05066064 | 0.36452056 | 2.84706306 | 0.00080513 | 0.00141794 | up |
| RP4-813D12.3\|ENSG00000226308\|lincRNA | 0.05065952 | 0.31428777 | 2.63318083 | 0.00030305 | 0.00057932 | up |
| CTC-273B12.10\|ENSG00000269814\|lincRNA | 0.05029341 | 0.58632118 | 3.54324986 | 8.25E-15 | 8.46E-14 | up |
| CTC-573N18.1\|ENSG00000250509\|lincRNA | 0.0502356 | 0.26939697 | 2.42295172 | 0.00102622 | 0.00177662 | up |
| RP11-366L20.2\|ENSG00000197301\|antisense | 0.0502328 | 0.68202878 | 3.76313114 | 4.55E-13 | 3.57E-12 | up |
| LA16c-312E8.2\|ENSG00000260132\|antisense | 0.05007656 | 0.34997796 | 2.80505674 | 2.56E-05 | 5.77E-05 | up |
| LINC00282\|ENSG00000281106\|lincRNA | 0.05002877 | 0.14055399 | 1.49029437 | 0.00429053 | 0.00661357 | up |
| RP11-583F2.2\|ENSG00000265912\|lincRNA | 0.05002387 | 0.28708403 | 2.52078446 | 8.73E-05 | 0.00018179 | up |
| RP5-1101C3.1\|ENSG00000240499\|antisense | 0.04998107 | 0.34978495 | 2.80701463 | 0.00058394 | 0.00105396 | up |
| CTB-114C7.4\|ENSG00000250274\|lincRNA | 0.04984019 | 0.23417509 | 2.23220602 | 5.09E-06 | 1.28E-05 | up |
| RP4-794I6.4\|ENSG00000277287\|lincRNA | 0.04955923 | 0.22380233 | 2.17499932 | 1.45E-09 | 6.34E-09 | up |
| RP11-290L1.5\|ENSG00000257329\|lincRNA | 0.04954969 | 0.81721075 | 4.04376031 | 9.01E-08 | 3.03E-07 | up |
| AC064834.1\|ENSG00000224099\|lincRNA | 0.04934324 | 13.8141978 | 8.12908363 | 1.63E-17 | 2.71E-16 | up |
| RP11-290L1.3\|ENSG00000257453\|antisense | 0.04917387 | 0.67414625 | 3.77709772 | 1.39E-14 | 1.37E-13 | up |
| RP11-728K20.2\|ENSG00000260555\|lincRNA | 0.04910223 | 0.30284648 | 2.62472613 | 1.48E-05 | 3.47E-05 | up |
| RP13-895J2.6\|ENSG00000277011\|lincRNA | 0.04901689 | 0.43135824 | 3.13753571 | 0.00179205 | 0.00296865 | up |
| RP11-484L8.1\|ENSG00000267764\|antisense | 0.04900954 | 0.30053988 | 2.61642202 | 0.03054071 | 0.04071838 | up |
| DGCR5\|ENSG00000237517\|antisense | 0.04889418 | 0.22857079 | 2.2249065 | 7.95E-07 | 2.28E-06 | up |
| AC007128.1\|ENSG00000229970\|antisense | 0.04873873 | 1.82725514 | 5.22846568 | 2.93E-22 | 1.63E-20 | up |
| RP11-334A14.8\|ENSG00000235563\|antisense | 0.04868089 | 0.28193714 | 2.53394596 | 2.58E-07 | 8.00E-07 | up |
| RP11-181E10.3\|ENSG00000271590\|lincRNA | 0.04867832 | 0.41460381 | 3.09038226 | 5.04E-09 | 2.04E-08 | up |
| CTD-2527I21.15\|ENSG00000179066\|lincRNA | 0.04851408 | 0.63179272 | 3.70297596 | 0.00010166 | 0.00020919 | up |
| RP11-320G24.1\|ENSG00000236393\|lincRNA | 0.0484998 | 0.19290805 | 1.9918627 | 1.48E-11 | 8.81E-11 | up |
| RP1-137D17.1\|ENSG00000226194\|lincRNA | 0.04846524 | 0.17883641 | 1.88361805 | 1.67E-05 | 3.87E-05 | up |
| AC004237.1\|ENSG00000230612\|antisense | 0.04844577 | 0.22742764 | 2.23096511 | 1.22E-06 | 3.38E-06 | up |
| RP11-401P9.5\|ENSG00000260249\|antisense | 0.04840356 | 0.1597356 | 1.72250067 | 2.77E-05 | 6.20E-05 | up |
| LA16c-358B7.4\|ENSG00000274751\|antisense | 0.04803001 | 0.1404488 | 1.54803641 | 1.55E-07 | 5.01E-07 | up |
| RP11-806L2.2\|ENSG00000266456\|antisense | 0.04790731 | 0.18716454 | 1.96598949 | 4.33E-07 | 1.29E-06 | up |
| RP11-394B2.6\|ENSG00000260156\|antisense | 0.04766315 | 0.30601248 | 2.68264434 | 1.03E-06 | 2.89E-06 | up |
| AC005363.11\|ENSG00000277602\|lincRNA | 0.04758755 | 0.50515642 | 3.40807422 | 3.32E-08 | 1.18E-07 | up |
| EVX1-AS\|ENSG00000253405\|antisense | 0.04733817 | 1.44781377 | 4.9347283 | 3.90E-11 | 2.14E-10 | up |
| RP11-434E6.4\|ENSG00000277559\|lincRNA | 0.04732009 | 0.64704024 | 3.7733306 | 0.00020598 | 0.00040471 | up |
| RP11-728G15.1\|ENSG00000256008\|antisense | 0.0472895 | 0.5870436 | 3.63387587 | 1.71E-11 | 1.00E-10 | up |
| LINC01484\|ENSG00000253686\|lincRNA | 0.0471626 | 0.25795174 | 2.4513861 | 9.74E-13 | 7.21E-12 | up |
| RP11-54A9.1\|ENSG00000257219\|lincRNA | 0.04682488 | 0.23935427 | 2.35380026 | 3.97E-10 | 1.88E-09 | up |
| PCBP3-OT1\|ENSG00000280604\|sense_overlapping | 0.04680585 | 0.18483765 | 1.9814979 | 0.00110456 | 0.00189552 | up |
| RP11-141O11.2\|ENSG00000249352\|lincRNA | 0.04671171 | 0.17763009 | 1.92701996 | 0.00047126 | 0.00086681 | up |
| LINC01585\|ENSG00000245479\|sense_overlapping | 0.04643633 | 0.12446552 | 1.42242016 | 0.00783091 | 0.01155991 | up |
| CTD-2653D5.1\|ENSG00000255438\|antisense | 0.04631501 | 0.27023238 | 2.54464898 | 1.17E-07 | 3.84E-07 | up |
| RP11-10A14.5\|ENSG00000248538\|lincRNA | 0.04582863 | 1.16439062 | 4.6671821 | 8.09E-16 | 9.88E-15 | up |
| LINC01021\|ENSG00000250337\|lincRNA | 0.04579926 | 1.47725134 | 5.01144737 | 4.30E-06 | 1.09E-05 | up |
| AC003104.1\|ENSG00000236194\|sense_intronic | 0.04577999 | 0.63792833 | 3.80060541 | 0.00018306 | 0.00036183 | up |
| RP11-544A12.8\|ENSG00000246851\|antisense | 0.04550748 | 0.37210545 | 3.03153582 | 0.0006482 | 0.00116045 | up |
| AC012363.4\|ENSG00000224789\|antisense | 0.04529371 | 1.14728161 | 4.66276501 | 1.68E-12 | 1.20E-11 | up |
| RP11-464D20.6\|ENSG00000264546\|sense_intronic | 0.04528806 | 0.31749874 | 2.80954829 | 0.00081376 | 0.00143266 | up |
| RP11-89K21.1\|ENSG00000259439\|lincRNA | 0.04483623 | 1.11656078 | 4.63825295 | 3.26E-08 | 1.16E-07 | up |
| LINC00524\|ENSG00000259023\|lincRNA | 0.04475303 | 0.74321808 | 4.05372847 | 1.78E-06 | 4.82E-06 | up |
| RP3-329E20.2\|ENSG00000236936\|antisense | 0.044749 | 0.37279513 | 3.05845568 | 0.00944323 | 0.01376749 | up |
| AC004221.2\|ENSG00000267778\|lincRNA | 0.044701 | 0.14484512 | 1.69613205 | 0.00287677 | 0.0045933 | up |
| RP11-336A10.2\|ENSG00000226647\|antisense | 0.04436777 | 0.7153694 | 4.0111044 | 0.02063933 | 0.0282857 | up |
| CTA-941F9.10\|ENSG00000280383\|sense_overlapping | 0.04428338 | 0.19459942 | 2.13567014 | 2.58E-12 | 1.77E-11 | up |
| RP11-135J2.3\|ENSG00000223842\|lincRNA | 0.04402972 | 0.31626152 | 2.84456842 | 0.0017577 | 0.00291539 | up |
| RP4-671O14.6\|ENSG00000280434\|sense_overlapping | 0.04386589 | 0.19353601 | 2.14143046 | 1.08E-05 | 2.57E-05 | up |
| DKFZP434K028\|ENSG00000124915\|lincRNA | 0.04370504 | 0.29277851 | 2.74393816 | 8.31E-08 | 2.81E-07 | up |
| AC112721.2\|ENSG00000222032\|lincRNA | 0.04369102 | 0.68347176 | 3.96747297 | 4.12E-09 | 1.69E-08 | up |
| RP11-818F20.5\|ENSG00000257732\|antisense | 0.04338044 | 0.18563465 | 2.0973496 | 0.00072386 | 0.00128291 | up |
| TSSC1-IT1\|ENSG00000224885\|sense_intronic | 0.04314692 | 0.53840874 | 3.64137231 | 1.77E-10 | 8.83E-10 | up |
| RP11-402G3.3\|ENSG00000230601\|lincRNA | 0.04288311 | 0.16997673 | 1.98685578 | 0.0040604 | 0.00629546 | up |
| FAM83A-AS1\|ENSG00000204949\|antisense | 0.04267401 | 0.20362756 | 2.25450339 | 0.00055405 | 0.00100617 | up |
| LINC01614\|ENSG00000230838\|lincRNA | 0.04255918 | 3.02831477 | 6.15290089 | 1.38E-17 | 2.32E-16 | up |
| RP11-157F20.3\|ENSG00000272202\|antisense | 0.04241911 | 0.23749654 | 2.48512033 | 4.95E-11 | 2.69E-10 | up |
| RP11-725P16.2\|ENSG00000272769\|lincRNA | 0.04239916 | 0.11746634 | 1.47013966 | 0.00119393 | 0.00203375 | up |
| RP11-13J8.1\|ENSG00000232719\|lincRNA | 0.04229509 | 0.13171001 | 1.63880294 | 0.00143466 | 0.0024105 | up |
| RP1-170O19.17\|ENSG00000253308\|lincRNA | 0.04223172 | 2.20555914 | 5.7066737 | 1.00E-10 | 5.18E-10 | up |
| RP11-121C6.5\|ENSG00000276272\|lincRNA | 0.04212229 | 0.20071187 | 2.2524702 | 6.77E-09 | 2.66E-08 | up |
| FLJ16779\|ENSG00000275620\|antisense | 0.04167692 | 0.33728731 | 3.01665744 | 1.66E-08 | 6.17E-08 | up |
| AC019118.4\|ENSG00000226649\|lincRNA | 0.04157416 | 0.28956007 | 2.80010369 | 1.85E-05 | 4.26E-05 | up |
| ASAP1-IT2\|ENSG00000280543\|sense_intronic | 0.04152741 | 0.31923265 | 2.94247227 | 0.00286501 | 0.00458004 | up |
| RP11-212P7.3\|ENSG00000273184\|processed_transcript | 0.04149585 | 0.14539925 | 1.80898098 | 7.79E-05 | 0.0001633 | up |
| AC104113.3\|ENSG00000233847\|antisense | 0.04139418 | 0.37466676 | 3.17810823 | 8.69E-05 | 0.00018098 | up |
| TTLL10-AS1\|ENSG00000205231\|antisense | 0.0413668 | 0.12346053 | 1.57750476 | 0.01417644 | 0.0200238 | up |
| RP11-405F3.5\|ENSG00000261633\|antisense | 0.04129209 | 0.5756567 | 3.80127148 | 5.63E-05 | 0.00012008 | up |
| RP11-266A24.1\|ENSG00000255418\|lincRNA | 0.04113686 | 0.13584391 | 1.72344632 | 0.00257618 | 0.00416224 | up |
| RP5-907D15.4\|ENSG00000268941\|lincRNA | 0.04104746 | 0.55207862 | 3.74950877 | 2.82E-14 | 2.64E-13 | up |
| RP4-676L2.1\|ENSG00000260253\|sense_overlapping | 0.04097891 | 0.3053431 | 2.89747776 | 0.00031192 | 0.00059434 | up |
| CTB-50L17.5\|ENSG00000267255\|antisense | 0.04092193 | 0.42088468 | 3.36247881 | 8.69E-10 | 3.91E-09 | up |
| RP11-255P5.2\|ENSG00000272542\|lincRNA | 0.04082927 | 0.19828404 | 2.27989305 | 0.00416271 | 0.00643457 | up |
| RP11-982M15.8\|ENSG00000260792\|lincRNA | 0.04072109 | 0.10959848 | 1.42837974 | 0.00089978 | 0.00157106 | up |
| VAC14-AS1\|ENSG00000214353\|antisense | 0.04056268 | 0.4718959 | 3.54024364 | 2.37E-23 | 1.87E-21 | up |
| RP5-1025A1.3\|ENSG00000275358\|lincRNA | 0.04053622 | 0.25984303 | 2.68035693 | 0.00068131 | 0.00121562 | up |
| RP11-354M1.2\|ENSG00000260798\|antisense | 0.03986224 | 0.23087211 | 2.53399922 | 2.53E-05 | 5.70E-05 | up |
| RP11-829H16.3\|ENSG00000258525\|antisense | 0.03978554 | 0.15746545 | 1.98471944 | 0.00437295 | 0.00673475 | up |
| RP11-422N16.3\|ENSG00000248050\|antisense | 0.03948068 | 0.13721646 | 1.79723475 | 0.00276515 | 0.00443593 | up |
| CASC11\|ENSG00000249375\|lincRNA | 0.03928864 | 0.13597249 | 1.79113048 | 1.49E-10 | 7.55E-10 | up |
| AP000640.2\|ENSG00000255355\|processed_transcript | 0.0391086 | 0.12848851 | 1.71608159 | 0.00022854 | 0.0004454 | up |
| RP1-20B11.2\|ENSG00000230433\|antisense | 0.03907477 | 0.42756031 | 3.45181875 | 1.76E-06 | 4.77E-06 | up |
| AC017002.2\|ENSG00000224959\|lincRNA | 0.03897193 | 0.70826269 | 4.18377743 | 0.00032177 | 0.00061158 | up |
| RP4-591N18.2\|ENSG00000232564\|antisense | 0.03894545 | 0.60785351 | 3.96419701 | 0.0036914 | 0.0057672 | up |
| RP11-568J23.6\|ENSG00000270159\|antisense | 0.03889837 | 0.30204165 | 2.95696585 | 9.12E-06 | 2.20E-05 | up |
| MACC1-AS1\|ENSG00000228598\|antisense | 0.03876942 | 1.42401243 | 5.19889875 | 0.00104126 | 0.00179971 | up |
| AP000679.2\|ENSG00000176984\|antisense | 0.03864508 | 0.1084705 | 1.48894602 | 0.00400791 | 0.00621953 | up |
| RP11-51J9.4\|ENSG00000253708\|antisense | 0.03851654 | 0.21921882 | 2.50882147 | 1.45E-05 | 3.40E-05 | up |
| RP11-10N16.3\|ENSG00000232298\|antisense | 0.03827039 | 0.18776544 | 2.29463106 | 0.00012134 | 0.00024642 | up |
| RP11-1008C21.1\|ENSG00000259225\|lincRNA | 0.03821773 | 0.60427006 | 3.98287967 | 1.31E-14 | 1.30E-13 | up |
| HOXC-AS2\|ENSG00000250133\|processed_transcript | 0.03782143 | 0.28233133 | 2.90011338 | 0.00164785 | 0.00274262 | up |
| TLX1NB\|ENSG00000236311\|processed_transcript | 0.03771479 | 0.17183503 | 2.18782205 | 4.82E-05 | 0.00010406 | up |
| RP11-434D9.1\|ENSG00000249364\|lincRNA | 0.03762646 | 0.10984149 | 1.54560352 | 0.00163255 | 0.00271802 | up |
| RP11-240G22.5\|ENSG00000274554\|antisense | 0.03742271 | 0.29418838 | 2.97475431 | 3.64E-06 | 9.36E-06 | up |
| RP11-303E16.7\|ENSG00000245059\|lincRNA | 0.03700656 | 0.15705922 | 2.08545561 | 0.00011484 | 0.0002345 | up |
| RP11-310P5.1\|ENSG00000249650\|antisense | 0.0365812 | 0.7130431 | 4.28481481 | 3.95E-14 | 3.64E-13 | up |
| RP4-811H24.9\|ENSG00000233775\|lincRNA | 0.03629172 | 0.15785348 | 2.1208737 | 0.00012174 | 0.00024697 | up |
| EWSAT1\|ENSG00000212766\|lincRNA | 0.03620078 | 0.1207007 | 1.73734124 | 1.09E-05 | 2.59E-05 | up |
| DSG1-AS1\|ENSG00000266729\|antisense | 0.03598517 | 0.28819913 | 3.00159178 | 9.61E-08 | 3.21E-07 | up |
| LEF1-AS1\|ENSG00000232021\|processed_transcript | 0.03592928 | 0.21521017 | 2.58251427 | 3.25E-18 | 6.17E-17 | up |
| RP5-1063M23.2\|ENSG00000236908\|lincRNA | 0.03584075 | 0.16335005 | 2.18829423 | 4.21E-06 | 1.07E-05 | up |
| RP11-242J7.1\|ENSG00000254233\|lincRNA | 0.03546161 | 0.16036047 | 2.17698869 | 0.03125568 | 0.04155665 | up |
| LINC00460\|ENSG00000233532\|lincRNA | 0.03542035 | 5.34707025 | 7.2380262 | 4.23E-24 | 4.88E-22 | up |
| AC108142.1\|ENSG00000177822\|antisense | 0.03525877 | 0.19629108 | 2.47694067 | 8.64E-06 | 2.09E-05 | up |
| AC092198.1\|ENSG00000223486\|lincRNA | 0.03497451 | 0.26994376 | 2.94828315 | 1.04E-08 | 3.98E-08 | up |
| RP11-79M19.2\|ENSG00000236426\|antisense | 0.03494756 | 0.22947258 | 2.71505813 | 0.01089018 | 0.01569572 | up |
| RP11-93G5.1\|ENSG00000274317\|lincRNA | 0.03391556 | 0.1555987 | 2.19781094 | 0.00040552 | 0.00075426 | up |
| RP11-322E11.2\|ENSG00000275512\|lincRNA | 0.03364383 | 0.13832864 | 2.03968605 | 2.78E-07 | 8.56E-07 | up |
| LINC01010\|ENSG00000236700\|lincRNA | 0.03349364 | 0.15180475 | 2.18025795 | 0.00293433 | 0.00467253 | up |
| LINC01271\|ENSG00000233077\|lincRNA | 0.03332942 | 0.13481483 | 2.01611127 | 2.18E-07 | 6.86E-07 | up |
| RP11-90D4.3\|ENSG00000256101\|lincRNA | 0.0328309 | 0.16486347 | 2.3281456 | 6.06E-05 | 0.00012843 | up |
| RP3-388N13.5\|ENSG00000264448\|lincRNA | 0.03274191 | 0.24395382 | 2.89739756 | 3.28E-10 | 1.57E-09 | up |
| RP11-123K3.9\|ENSG00000276727\|antisense | 0.03265804 | 0.1976942 | 2.59776032 | 2.26E-06 | 6.00E-06 | up |
| AC007277.3\|ENSG00000213981\|antisense | 0.03262091 | 1.42327625 | 5.44727501 | 7.97E-15 | 8.21E-14 | up |
| RP11-535A19.1\|ENSG00000254814\|antisense | 0.03257437 | 0.28982902 | 3.1533928 | 2.31E-09 | 9.86E-09 | up |
| CTD-3060P21.1\|ENSG00000262884\|antisense | 0.03227258 | 0.13855346 | 2.10206205 | 0.00837039 | 0.01229807 | up |
| RP11-874J12.4\|ENSG00000266401\|antisense | 0.0321517 | 0.55683152 | 4.11427394 | 1.41E-11 | 8.42E-11 | up |
| AC137723.5\|ENSG00000235296\|antisense | 0.03203515 | 0.47781413 | 3.8987218 | 7.11E-11 | 3.80E-10 | up |
| RP11-297P16.4\|ENSG00000250920\|lincRNA | 0.03200502 | 13.0101525 | 8.66712395 | 3.43E-05 | 7.56E-05 | up |
| RP11-180C16.1\|ENSG00000273489\|antisense | 0.03152481 | 0.15457594 | 2.29375606 | 3.86E-09 | 1.58E-08 | up |
| LRRC2-AS1\|ENSG00000268324\|antisense | 0.03151626 | 0.15634637 | 2.31057737 | 3.64E-09 | 1.50E-08 | up |
| MPRIP-AS1\|ENSG00000225442\|antisense | 0.03146325 | 0.42194995 | 3.74533225 | 0.00241882 | 0.00392716 | up |
| MYCNOS\|ENSG00000233718\|antisense | 0.03145518 | 0.14573429 | 2.21197088 | 0.00048023 | 0.00088208 | up |
| AC112721.1\|ENSG00000222022\|lincRNA | 0.03130979 | 0.38578736 | 3.6231202 | 1.10E-07 | 3.64E-07 | up |
| CTB-113P19.1\|ENSG00000249035\|antisense | 0.03123341 | 0.50069661 | 4.00277465 | 8.61E-09 | 3.32E-08 | up |
| ST7-OT4\|ENSG00000214188\|sense_intronic | 0.03115477 | 0.51923689 | 4.05886799 | 5.45E-10 | 2.52E-09 | up |
| AC008746.3\|ENSG00000227407\|antisense | 0.03111962 | 0.24596227 | 2.98254095 | 1.28E-05 | 3.03E-05 | up |
| MRPL23-AS1\|ENSG00000226416\|antisense | 0.0308375 | 0.46527164 | 3.91531552 | 8.17E-05 | 0.00017068 | up |
| RP11-170M17.1\|ENSG00000228566\|lincRNA | 0.03079047 | 0.61500605 | 4.32004468 | 7.43E-13 | 5.61E-12 | up |
| RP11-510M2.5\|ENSG00000260015\|sense_intronic | 0.03062962 | 2.25300916 | 6.20078204 | 3.33E-07 | 1.01E-06 | up |
| RP3-340N1.2\|ENSG00000227066\|lincRNA | 0.03059649 | 0.13602951 | 2.15248164 | 0.00783734 | 0.01156619 | up |
| RP11-680F20.10\|ENSG00000260209\|lincRNA | 0.03055694 | 0.15306067 | 2.3245317 | 0.02661128 | 0.03592377 | up |
| FIRRE\|ENSG00000213468\|lincRNA | 0.03038176 | 1.98327953 | 6.02853877 | 1.91E-20 | 6.56E-19 | up |
| LINC01433\|ENSG00000230176\|antisense | 0.03023642 | 0.42072796 | 3.79852864 | 2.23E-15 | 2.52E-14 | up |
| RP3-416H24.1\|ENSG00000257671\|antisense | 0.03005342 | 0.50787149 | 4.07886255 | 4.69E-17 | 7.23E-16 | up |
| HMMR-AS1\|ENSG00000251018\|antisense | 0.03004321 | 0.1233933 | 2.03815311 | 0.00204004 | 0.00335221 | up |
| RP11-717D12.1\|ENSG00000214773\|sense_intronic | 0.02980629 | 0.37932673 | 3.66975215 | 0.00449414 | 0.00690334 | up |
| RP11-231I16.1\|ENSG00000257279\|antisense | 0.02958803 | 0.27620487 | 3.22265345 | 1.14E-09 | 5.07E-09 | up |
| TBL1XR1-AS1\|ENSG00000231310\|antisense | 0.02941605 | 0.77709579 | 4.7234172 | 3.65E-06 | 9.40E-06 | up |
| RP11-674E16.4\|ENSG00000214919\|sense_intronic | 0.02881168 | 0.21321313 | 2.88757062 | 3.24E-10 | 1.55E-09 | up |
| RP11-93K22.6\|ENSG00000250643\|antisense | 0.02851867 | 0.31561621 | 3.46819267 | 8.88E-06 | 2.15E-05 | up |
| RBAKDN\|ENSG00000273313\|lincRNA | 0.02834307 | 0.37416254 | 3.72259712 | 2.11E-06 | 5.61E-06 | up |
| FMR1-AS1\|ENSG00000268066\|processed_transcript | 0.02818779 | 0.10989461 | 1.96297864 | 1.00E-05 | 2.40E-05 | up |
| RP1-27K12.2\|ENSG00000231683\|lincRNA | 0.02817108 | 0.40239968 | 3.83634233 | 0.00354028 | 0.00555566 | up |
| AC004988.1\|ENSG00000203446\|antisense | 0.02811734 | 0.55055261 | 4.29134868 | 7.42E-13 | 5.61E-12 | up |
| CTD-2553C6.1\|ENSG00000267650\|antisense | 0.02798596 | 0.18126009 | 2.6952863 | 0.00022019 | 0.00043008 | up |
| LA16c-313D11.9\|ENSG00000260394\|antisense | 0.02777765 | 0.14681906 | 2.40204305 | 2.84E-07 | 8.73E-07 | up |
| RP11-54O7.1\|ENSG00000230699\|lincRNA | 0.02774798 | 0.23106236 | 3.05782779 | 9.89E-07 | 2.78E-06 | up |
| PHACTR2-AS1\|ENSG00000235740\|antisense | 0.02727671 | 0.64532865 | 4.56429252 | 0.0294137 | 0.03933452 | up |
| CFAP44-AS1\|ENSG00000243849\|antisense | 0.02721983 | 0.28130602 | 3.3694106 | 0.00259899 | 0.00419654 | up |
| RP11-114H23.2\|ENSG00000258088\|lincRNA | 0.02709501 | 0.55130661 | 4.34675598 | 2.28E-10 | 1.12E-09 | up |
| RP11-575L7.4\|ENSG00000231616\|antisense | 0.02688351 | 0.13602552 | 2.33908402 | 0.0030184 | 0.00479489 | up |
| AC012531.25\|ENSG00000260597\|lincRNA | 0.02662406 | 0.21939341 | 3.04271753 | 0.03479804 | 0.04586385 | up |
| RP11-54O7.18\|ENSG00000273443\|lincRNA | 0.02659284 | 0.2307509 | 3.11722672 | 6.84E-06 | 1.69E-05 | up |
| RP4-621N11.2\|ENSG00000269846\|antisense | 0.02656404 | 0.10896279 | 2.0362892 | 1.43E-05 | 3.35E-05 | up |
| CTB-181H17.1\|ENSG00000272219\|sense_intronic | 0.02634309 | 0.44358709 | 4.07372109 | 0.00806562 | 0.01188653 | up |
| AC007163.6\|ENSG00000230408\|antisense | 0.02625352 | 0.17372807 | 2.72624789 | 0.0001806 | 0.0003575 | up |
| RP11-109M17.2\|ENSG00000270372\|lincRNA | 0.02622753 | 11.6351336 | 8.79319002 | 2.31E-10 | 1.13E-09 | up |
| AC144450.2\|ENSG00000203635\|lincRNA | 0.02611111 | 0.56470638 | 4.43476507 | 4.71E-09 | 1.91E-08 | up |
| AC006262.5\|ENSG00000268621\|lincRNA | 0.02593493 | 2.07345152 | 6.32099412 | 6.15E-15 | 6.49E-14 | up |
| LINC01276\|ENSG00000226917\|lincRNA | 0.02573994 | 0.1820038 | 2.82188798 | 5.85E-05 | 0.00012444 | up |
| LINC00922\|ENSG00000261742\|lincRNA | 0.02570259 | 0.19425002 | 2.9179291 | 2.13E-10 | 1.05E-09 | up |
| CTD-2171N6.1\|ENSG00000267013\|lincRNA | 0.02568162 | 0.31875804 | 3.63365381 | 3.36E-12 | 2.26E-11 | up |
| RP11-50D9.3\|ENSG00000261668\|antisense | 0.02508379 | 0.15840836 | 2.65882108 | 1.27E-05 | 3.00E-05 | up |
| RP1-40E16.9\|ENSG00000230269\|lincRNA | 0.02495739 | 0.35702908 | 3.83850262 | 0.0031069 | 0.00492514 | up |
| RP13-487P22.1\|ENSG00000261529\|sense_intronic | 0.02493195 | 0.34661857 | 3.79728154 | 0.01060884 | 0.01533192 | up |
| RP11-513G11.4\|ENSG00000225742\|lincRNA | 0.02483036 | 0.13842941 | 2.47897333 | 0.00023686 | 0.00046009 | up |
| RP11-141J13.5\|ENSG00000261863\|lincRNA | 0.0248173 | 0.94450537 | 5.25014098 | 1.90E-13 | 1.57E-12 | up |
| DBET\|ENSG00000281591\|lincRNA | 0.02464251 | 0.27423488 | 3.47619104 | 3.59E-05 | 7.89E-05 | up |
| RP11-338N10.1\|ENSG00000270171\|lincRNA | 0.02454941 | 0.11628502 | 2.24390489 | 0.02334321 | 0.03181855 | up |
| RP11-573D15.2\|ENSG00000232233\|lincRNA | 0.02442495 | 0.15966865 | 2.7086533 | 1.60E-08 | 5.95E-08 | up |
| KCNQ1-AS1\|ENSG00000229414\|antisense | 0.02423124 | 0.12484169 | 2.3651598 | 0.00215514 | 0.00352931 | up |
| RP11-304L19.12\|ENSG00000259780\|lincRNA | 0.02408096 | 0.13481094 | 2.4849727 | 6.86E-06 | 1.69E-05 | up |
| CTC-137K3.1\|ENSG00000270137\|sense_intronic | 0.0239789 | 0.30474564 | 3.66776851 | 8.29E-06 | 2.01E-05 | up |
| PDX1-AS1\|ENSG00000247381\|antisense | 0.02382594 | 1.04802407 | 5.45899464 | 1.14E-20 | 4.21E-19 | up |
| RP11-126O1.4\|ENSG00000267476\|antisense | 0.0238134 | 1.09520389 | 5.52328227 | 2.61E-07 | 8.08E-07 | up |
| RP5-856G1.1\|ENSG00000270937\|lincRNA | 0.02379213 | 0.26255903 | 3.46408577 | 1.99E-05 | 4.54E-05 | up |
| RP1-232P20.1\|ENSG00000269985\|antisense | 0.02373727 | 0.36839537 | 3.95602917 | 0.00515403 | 0.00783075 | up |
| IGF2-AS\|ENSG00000099869\|antisense | 0.02367226 | 0.37691983 | 3.99298825 | 0.00014934 | 0.00029908 | up |
| AC016738.3\|ENSG00000230140\|antisense | 0.02360886 | 0.170544 | 2.85274348 | 0.00036876 | 0.00069124 | up |
| RP11-120K18.2\|ENSG00000260757\|sense_overlapping | 0.02344787 | 0.21878754 | 3.22200179 | 2.83E-08 | 1.01E-07 | up |
| LINC00858\|ENSG00000229404\|lincRNA | 0.02310485 | 1.81622345 | 6.29660201 | 1.07E-16 | 1.54E-15 | up |
| LINC00885\|ENSG00000224652\|lincRNA | 0.02308201 | 0.15486912 | 2.7462085 | 9.81E-05 | 0.00020258 | up |
| PCAT14\|ENSG00000280623\|lincRNA | 0.02293328 | 1.63936666 | 6.15955203 | 3.76E-05 | 8.23E-05 | up |
| LINC00862\|ENSG00000203721\|lincRNA | 0.02283685 | 0.19101867 | 3.06427828 | 2.83E-08 | 1.01E-07 | up |
| SLC7A11-AS1\|ENSG00000250033\|processed_transcript | 0.02269066 | 0.34875148 | 3.94202913 | 3.52E-09 | 1.46E-08 | up |
| RP11-434D9.2\|ENSG00000249894\|lincRNA | 0.02227934 | 0.2112818 | 3.24539013 | 1.06E-05 | 2.53E-05 | up |
| PROX1-AS1\|ENSG00000272167\|antisense | 0.02204531 | 0.26458428 | 3.58518372 | 0.0001018 | 0.00020942 | up |
| DDC-AS1\|ENSG00000226122\|antisense | 0.0218136 | 0.17808493 | 3.0292654 | 5.48E-05 | 0.00011725 | up |
| RP11-524C21.2\|ENSG00000261568\|lincRNA | 0.02140302 | 0.10880702 | 2.34588513 | 0.00010848 | 0.0002222 | up |
| CARS-AS1\|ENSG00000247473\|antisense | 0.02128274 | 0.17490435 | 3.03881014 | 1.76E-06 | 4.76E-06 | up |
| RP4-594A5.1\|ENSG00000272328\|lincRNA | 0.02096927 | 1.04213444 | 5.635121 | 4.36E-19 | 1.01E-17 | up |
| LINC00939\|ENSG00000249267\|lincRNA | 0.02096196 | 0.11244323 | 2.42335118 | 0.00453905 | 0.00696745 | up |
| DLGAP1-AS5\|ENSG00000261520\|antisense | 0.02034911 | 1.26530084 | 5.95837062 | 0.00709547 | 0.01055658 | up |
| RP11-161I6.2\|ENSG00000263745\|lincRNA | 0.02021961 | 0.432224 | 4.41795248 | 8.41E-16 | 1.03E-14 | up |
| RP11-635N19.3\|ENSG00000269989\|sense_intronic | 0.02015316 | 0.67259527 | 5.06066042 | 2.38E-08 | 8.61E-08 | up |
| RP4-598P13.1\|ENSG00000248458\|antisense | 0.0198534 | 0.53715303 | 4.75787514 | 0.00015197 | 0.00030388 | up |
| RP11-544M22.8\|ENSG00000228917\|antisense | 0.01945289 | 0.17921909 | 3.20366817 | 5.15E-06 | 1.30E-05 | up |
| LINC00887\|ENSG00000214145\|lincRNA | 0.01918769 | 0.11096433 | 2.53184311 | 3.78E-08 | 1.34E-07 | up |
| RP11-474D1.3\|ENSG00000214039\|lincRNA | 0.0191353 | 12.8131316 | 9.38717062 | 7.67E-20 | 2.12E-18 | up |
| AC007750.5\|ENSG00000236841\|antisense | 0.0191206 | 0.23752282 | 3.63486681 | 2.10E-12 | 1.47E-11 | up |
| RP11-126H7.4\|ENSG00000204049\|antisense | 0.01905877 | 0.26029055 | 3.77159608 | 1.37E-05 | 3.23E-05 | up |
| ITPK1-AS1\|ENSG00000258730\|antisense | 0.01870807 | 0.26530123 | 3.82589883 | 0.01567189 | 0.02194893 | up |
| AC142293.3\|ENSG00000249942\|antisense | 0.0186214 | 1.20287089 | 6.0133762 | 3.88E-12 | 2.59E-11 | up |
| LINC01411\|ENSG00000249306\|lincRNA | 0.0184931 | 2.34866002 | 6.98870722 | 8.06E-17 | 1.19E-15 | up |
| AC005592.1\|ENSG00000236714\|lincRNA | 0.0182813 | 0.10747341 | 2.55553932 | 8.28E-05 | 0.00017292 | up |
| RP11-142O6.1\|ENSG00000266371\|sense_intronic | 0.01812708 | 0.45868069 | 4.6612719 | 0.02624648 | 0.03549301 | up |
| AC005256.1\|ENSG00000267073\|lincRNA | 0.01785739 | 2.71856262 | 7.25017899 | 1.87E-17 | 3.09E-16 | up |
| RP4-784A16.3\|ENSG00000225030\|sense_intronic | 0.01751121 | 0.53428367 | 4.93125545 | 1.41E-07 | 4.58E-07 | up |
| CTC-529P8.1\|ENSG00000250072\|lincRNA | 0.01746374 | 0.17256274 | 3.30468638 | 1.98E-12 | 1.39E-11 | up |
| LINC01169\|ENSG00000259471\|lincRNA | 0.01741615 | 1.56172175 | 6.48656807 | 1.65E-10 | 8.28E-10 | up |
| RP11-264E20.1\|ENSG00000255465\|lincRNA | 0.01738342 | 0.12294734 | 2.82225698 | 1.15E-07 | 3.79E-07 | up |
| CTD-2147F2.2\|ENSG00000259664\|lincRNA | 0.01712175 | 0.49368062 | 4.84967639 | 1.40E-08 | 5.26E-08 | up |
| RP11-817J15.2\|ENSG00000256916\|antisense | 0.0170969 | 1.08818207 | 5.99204168 | 4.13E-14 | 3.78E-13 | up |
| RP1-29C18.10\|ENSG00000212939\|lincRNA | 0.01692247 | 0.15276307 | 3.17428377 | 0.0028963 | 0.0046203 | up |
| RP11-276H1.3\|ENSG00000175604\|antisense | 0.01684295 | 0.12470855 | 2.8883433 | 0.01173299 | 0.01684631 | up |
| AL773572.7\|ENSG00000225745\|antisense | 0.01676499 | 0.94038879 | 5.80973405 | 1.18E-11 | 7.20E-11 | up |
| CTD-3064M3.4\|ENSG00000244998\|antisense | 0.01659699 | 0.26056743 | 3.97266367 | 5.44E-13 | 4.21E-12 | up |
| SMCR5\|ENSG00000226746\|antisense | 0.01658245 | 0.2004765 | 3.59570414 | 0.0317314 | 0.04214687 | up |
| RP1-79C4.4\|ENSG00000271811\|lincRNA | 0.01653668 | 0.41629624 | 4.65386931 | 1.80E-09 | 7.77E-09 | up |
| CTD-2090I13.1\|ENSG00000234277\|lincRNA | 0.01646608 | 0.12044156 | 2.87076439 | 0.00717104 | 0.01066003 | up |
| RP11-60L3.1\|ENSG00000259264\|antisense | 0.01644404 | 0.6589867 | 5.32461259 | 4.59E-15 | 4.93E-14 | up |
| RP1-78B3.1\|ENSG00000279652\|lincRNA | 0.01636881 | 0.30166889 | 4.20394426 | 1.10E-06 | 3.08E-06 | up |
| RP11-16C1.2\|ENSG00000264853\|sense_intronic | 0.01610199 | 0.53595153 | 5.05679162 | 0.02086692 | 0.02856806 | up |
| MYO16-AS1\|ENSG00000236242\|antisense | 0.01605834 | 0.58527419 | 5.18771764 | 9.98E-07 | 2.80E-06 | up |
| COL4A2-AS2\|ENSG00000224821\|antisense | 0.01596095 | 0.1989301 | 3.63964305 | 3.64E-05 | 7.98E-05 | up |
| RP1-140K8.5\|ENSG00000260604\|lincRNA | 0.01579923 | 0.40264166 | 4.6715705 | 9.87E-18 | 1.70E-16 | up |
| RP11-400N13.3\|ENSG00000232679\|lincRNA | 0.01569301 | 3.97373302 | 7.98422875 | 1.58E-20 | 5.55E-19 | up |
| LINC01267\|ENSG00000251576\|lincRNA | 0.01566078 | 0.1732756 | 3.46784023 | 9.55E-08 | 3.19E-07 | up |
| RP11-47I22.2\|ENSG00000250548\|lincRNA | 0.0155645 | 0.12230792 | 2.97418673 | 9.05E-11 | 4.75E-10 | up |
| RP11-469H8.6\|ENSG00000257588\|antisense | 0.01547777 | 1.2620806 | 6.34946259 | 8.94E-20 | 2.42E-18 | up |
| AC018359.1\|ENSG00000226320\|lincRNA | 0.0153044 | 2.15932595 | 7.14049044 | 1.96E-19 | 4.86E-18 | up |
| STEAP2-AS1\|ENSG00000227646\|processed_transcript | 0.01512782 | 0.15414814 | 3.34904174 | 1.23E-08 | 4.63E-08 | up |
| CTD-2542L18.1\|ENSG00000260417\|lincRNA | 0.01476473 | 0.27427677 | 4.21540568 | 0.0009372 | 0.00163263 | up |
| RP1-142L7.9\|ENSG00000270661\|lincRNA | 0.01460712 | 0.29010751 | 4.31184396 | 2.07E-06 | 5.52E-06 | up |
| RP11-547D24.1\|ENSG00000233542\|antisense | 0.01438289 | 0.56357056 | 5.2921707 | 4.17E-22 | 2.23E-20 | up |
| INHBA-AS1\|ENSG00000224116\|antisense | 0.01390404 | 0.1472237 | 3.40443428 | 8.33E-11 | 4.40E-10 | up |
| CCAT2\|ENSG00000280997\|lincRNA | 0.01385594 | 0.56383852 | 5.34670559 | 9.11E-10 | 4.09E-09 | up |
| RP11-159D23.2\|ENSG00000257472\|lincRNA | 0.01383785 | 0.23009022 | 4.05550763 | 5.86E-08 | 2.03E-07 | up |
| RP11-114H23.1\|ENSG00000258077\|lincRNA | 0.01375298 | 0.74780315 | 5.76484293 | 3.88E-18 | 7.25E-17 | up |
| CLDN10-AS1\|ENSG00000223392\|antisense | 0.01367729 | 1.3155128 | 6.58769956 | 2.71E-12 | 1.85E-11 | up |
| DLX6-AS1\|ENSG00000231764\|antisense | 0.01362244 | 0.3219168 | 4.56263132 | 8.96E-05 | 0.00018612 | up |
| LINC00658\|ENSG00000226995\|lincRNA | 0.01321838 | 0.19195778 | 3.86017194 | 1.65E-05 | 3.81E-05 | up |
| RP3-323A16.1\|ENSG00000279184\|antisense | 0.01317755 | 0.14516002 | 3.46149008 | 7.23E-18 | 1.30E-16 | up |
| RP5-884M6.1\|ENSG00000228742\|lincRNA | 0.01305359 | 1.66246916 | 6.99273702 | 4.07E-25 | 7.76E-23 | up |
| SND1-IT1\|ENSG00000279078\|lincRNA | 0.01300315 | 0.68655782 | 5.72244805 | 0.00454781 | 0.0069777 | up |
| RP11-416I2.1\|ENSG00000260328\|lincRNA | 0.01286183 | 0.34261375 | 4.73541475 | 5.57E-19 | 1.26E-17 | up |
| RP11-96B2.1\|ENSG00000255325\|lincRNA | 0.01284787 | 0.34683779 | 4.75466015 | 6.31E-07 | 1.84E-06 | up |
| CRAT37\|ENSG00000258551\|lincRNA | 0.01283782 | 0.73312843 | 5.83559432 | 1.16E-08 | 4.40E-08 | up |
| OSBPL10-AS1\|ENSG00000232490\|antisense | 0.01281205 | 0.23488502 | 4.19638141 | 0.00048723 | 0.0008937 | up |
| LINC01234\|ENSG00000249550\|lincRNA | 0.01244152 | 3.04142103 | 7.93343946 | 1.91E-16 | 2.63E-15 | up |
| RP11-53M11.3\|ENSG00000254142\|lincRNA | 0.01171737 | 0.91961446 | 6.29430893 | 1.38E-16 | 1.96E-15 | up |
| RP11-865I6.2\|ENSG00000254337\|lincRNA | 0.01168273 | 0.46601101 | 5.3179151 | 7.12E-08 | 2.44E-07 | up |
| RP11-30O15.1\|ENSG00000224049\|lincRNA | 0.01141702 | 0.31575293 | 4.78953767 | 1.05E-11 | 6.49E-11 | up |
| AC005264.2\|ENSG00000267551\|antisense | 0.01132861 | 0.15325361 | 3.75787846 | 9.22E-15 | 9.31E-14 | up |
| RP11-115D19.1\|ENSG00000251095\|antisense | 0.01110854 | 1.25168613 | 6.81606042 | 1.80E-07 | 5.72E-07 | up |
| LINC01511\|ENSG00000250584\|lincRNA | 0.01092629 | 0.47789521 | 5.45081895 | 2.18E-11 | 1.25E-10 | up |
| ABCA9-AS1\|ENSG00000231749\|antisense | 0.01075479 | 0.27442471 | 4.67335952 | 1.59E-07 | 5.13E-07 | up |
| AC068580.7\|ENSG00000230834\|lincRNA | 0.01047833 | 1.29484041 | 6.94922212 | 5.76E-10 | 2.65E-09 | up |
| LEMD1-AS1\|ENSG00000226235\|antisense | 0.0103544 | 0.18060715 | 4.12453984 | 3.88E-10 | 1.83E-09 | up |
| AL162759.1\|ENSG00000235269\|lincRNA | 0.01019399 | 0.55224079 | 5.75950646 | 3.62E-11 | 2.00E-10 | up |
| RP5-1120P11.3\|ENSG00000231881\|lincRNA | 0.00988728 | 0.20248159 | 4.35607365 | 1.22E-10 | 6.21E-10 | up |
| PCAT2\|ENSG00000253264\|lincRNA | 0.00988042 | 0.75242676 | 6.25083477 | 4.48E-13 | 3.51E-12 | up |
| RP11-332K15.1\|ENSG00000232524\|antisense | 0.00941169 | 0.34539008 | 5.19762978 | 2.68E-10 | 1.30E-09 | up |
| RP11-474D1.4\|ENSG00000261650\|lincRNA | 0.00892591 | 4.54376272 | 8.99167256 | 5.76E-17 | 8.75E-16 | up |
| C15orf54\|ENSG00000175746\|lincRNA | 0.0087808 | 0.18248502 | 4.37728178 | 9.15E-08 | 3.07E-07 | up |
| CTD-2015G9.2\|ENSG00000261175\|lincRNA | 0.00876897 | 0.20862113 | 4.57233405 | 2.99E-07 | 9.15E-07 | up |
| WT1-AS\|ENSG00000183242\|antisense | 0.00844409 | 0.115243 | 3.77059391 | 0.00115639 | 0.00197424 | up |
| RP11-454P21.1\|ENSG00000249937\|lincRNA | 0.00838057 | 0.39079135 | 5.54320606 | 7.84E-12 | 4.95E-11 | up |
| RP11-138J23.1\|ENSG00000251026\|lincRNA | 0.00795852 | 3.84459401 | 8.91611604 | 2.96E-21 | 1.22E-19 | up |
| DNAH17-AS1\|ENSG00000267432\|processed_transcript | 0.00760783 | 0.12953894 | 4.08975656 | 4.58E-13 | 3.58E-12 | up |
| RP11-815M8.1\|ENSG00000238042\|lincRNA | 0.00757115 | 0.92804754 | 6.93754217 | 8.51E-17 | 1.25E-15 | up |
| RP11-167H9.4\|ENSG00000243944\|antisense | 0.00753975 | 1.10888444 | 7.20037644 | 1.09E-15 | 1.31E-14 | up |
| LINC00698\|ENSG00000244342\|lincRNA | 0.00747279 | 0.18013034 | 4.59125034 | 2.48E-13 | 2.01E-12 | up |
| CTA-414D7.1\|ENSG00000279175\|sense_intronic | 0.00683608 | 0.11238638 | 4.03915421 | 3.61E-09 | 1.49E-08 | up |
| LINC01429\|ENSG00000227964\|lincRNA | 0.00677497 | 0.15957162 | 4.5578461 | 9.40E-07 | 2.65E-06 | up |
| RP11-264E20.2\|ENSG00000270160\|lincRNA | 0.00655021 | 0.1226847 | 4.22726984 | 1.47E-08 | 5.47E-08 | up |
| FEZF1-AS1\|ENSG00000230316\|antisense | 0.00638493 | 4.68029999 | 9.5177157 | 8.98E-22 | 4.33E-20 | up |
| ARNTL2-AS1\|ENSG00000245311\|antisense | 0.00634779 | 0.29027126 | 5.51500264 | 8.86E-08 | 2.98E-07 | up |
| RP11-191N8.2\|ENSG00000227925\|lincRNA | 0.00620595 | 0.73886425 | 6.89551258 | 4.27E-10 | 2.00E-09 | up |
| AC007099.1\|ENSG00000231172\|antisense | 0.00596333 | 2.34511242 | 8.6193229 | 5.86E-22 | 2.99E-20 | up |
| LINC00592\|ENSG00000258279\|lincRNA | 0.00548586 | 0.12558151 | 4.51676253 | 5.97E-09 | 2.37E-08 | up |
| RP11-404O13.1\|ENSG00000231666\|lincRNA | 0.00527175 | 0.14282072 | 4.75977851 | 2.60E-10 | 1.26E-09 | up |
| RP11-742B18.1\|ENSG00000249001\|antisense | 0.00516872 | 0.27036821 | 5.70897564 | 7.68E-08 | 2.61E-07 | up |
| CTC-327F10.4\|ENSG00000251320\|antisense | 0.00494756 | 0.62625902 | 6.98389895 | 2.23E-08 | 8.09E-08 | up |
| AC133785.1\|ENSG00000233221\|antisense | 0.0048324 | 0.14391465 | 4.89633112 | 1.36E-10 | 6.91E-10 | up |
| RP11-254F7.1\|ENSG00000260476\|lincRNA | 0.0045616 | 0.35797641 | 6.29418068 | 3.21E-12 | 2.17E-11 | up |
| RP11-400N13.2\|ENSG00000228437\|lincRNA | 0.00417036 | 2.18601973 | 9.03391878 | 5.42E-11 | 2.93E-10 | up |
| LINC01050\|ENSG00000271216\|lincRNA | 0.00405928 | 0.9199613 | 7.82420472 | 4.57E-15 | 4.93E-14 | up |
| AP000697.6\|ENSG00000224269\|antisense | 0.00393417 | 1.08378567 | 8.10580509 | 1.80E-16 | 2.49E-15 | up |
| RP11-401P9.1\|ENSG00000260029\|antisense | 0.00387288 | 1.38994484 | 8.48740403 | 1.12E-07 | 3.71E-07 | up |
| LINC01593\|ENSG00000231221\|lincRNA | 0.00376056 | 0.95546512 | 7.98911057 | 4.60E-11 | 2.52E-10 | up |
| RP11-143E21.3\|ENSG00000276122\|lincRNA | 0.00347952 | 2.82497499 | 9.66513287 | 4.03E-16 | 5.25E-15 | up |
| AC010967.2\|ENSG00000228033\|lincRNA | 0.00292049 | 0.47481482 | 7.34501059 | 4.74E-11 | 2.59E-10 | up |
| CTD-2034I21.2\|ENSG00000263082\|lincRNA | 0.00280194 | 0.34964549 | 6.96332529 | 3.45E-07 | 1.04E-06 | up |
| NPSR1-AS1\|ENSG00000197085\|processed_transcript | 0.0028002 | 0.50103867 | 7.48325016 | 1.91E-19 | 4.79E-18 | up |
| LINC01602\|ENSG00000205293\|lincRNA | 0.00256404 | 0.61848047 | 7.91416833 | 8.61E-13 | 6.44E-12 | up |
| AC079466.1\|ENSG00000266976\|lincRNA | 0.00248905 | 0.72562536 | 8.18748416 | 1.70E-10 | 8.52E-10 | up |
| RP11-197K6.1\|ENSG00000223812\|lincRNA | 0.00173575 | 2.07253749 | 10.2216233 | 2.25E-11 | 1.29E-10 | up |
| RP11-142A22.4\|ENSG00000272727\|lincRNA | 0.00157456 | 0.25923739 | 7.36317923 | 2.39E-09 | 1.01E-08 | up |
| RP11-135D11.2\|ENSG00000235140\|lincRNA | 0.81296314 | 0.04532491 | -4.1648138 | 6.35E-43 | 3.36E-39 | down |
| RP11-1090M7.1\|ENSG00000265489\|antisense | 0.77562234 | 0.05518229 | -3.8130772 | 1.97E-24 | 2.61E-22 | down |
| XXyac-YM21GA2.7\|ENSG00000214888\|antisense | 1.8570654 | 0.05622859 | -5.0455771 | 7.31E-31 | 4.85E-28 | down |
| RP11-481J13.1\|ENSG00000272180\|lincRNA | 0.51591008 | 0.06680544 | -2.9490822 | 1.47E-11 | 8.76E-11 | down |
| RP11-646E18.2\|ENSG00000238837\|lincRNA | 0.46593583 | 0.07099041 | -2.7144353 | 4.79E-24 | 5.19E-22 | down |
| RP11-17M24.1\|ENSG00000265356\|sense_intronic | 0.94238671 | 0.07702038 | -3.6130071 | 1.53E-35 | 2.03E-32 | down |
| RP11-654D12.2\|ENSG00000258216\|lincRNA | 0.57492787 | 0.07833058 | -2.8757335 | 8.02E-23 | 5.18E-21 | down |
| RP11-232L2.2\|ENSG00000251599\|antisense | 0.68167848 | 0.07877164 | -3.1133432 | 9.15E-23 | 5.78E-21 | down |
| AC004947.2\|ENSG00000233760\|lincRNA | 0.58850648 | 0.07995323 | -2.8798301 | 1.53E-23 | 1.35E-21 | down |
| BVES-AS1\|ENSG00000203808\|antisense | 0.59412971 | 0.0804085 | -2.8853581 | 6.23E-20 | 1.80E-18 | down |
| LINC00974\|ENSG00000226629\|lincRNA | 1.87441337 | 0.08068497 | -4.5379953 | 8.40E-30 | 4.95E-27 | down |
| MRGPRF-AS1\|ENSG00000256508\|antisense | 0.37430729 | 0.08072239 | -2.2131824 | 9.86E-13 | 7.27E-12 | down |
| PCAT18\|ENSG00000265369\|lincRNA | 0.48695309 | 0.0817177 | -2.5750623 | 5.37E-25 | 8.63E-23 | down |
| AC104024.1\|ENSG00000230709\|lincRNA | 0.37397827 | 0.0817852 | -2.1930428 | 1.03E-18 | 2.24E-17 | down |
| MEF2C-AS1\|ENSG00000248309\|antisense | 0.40546324 | 0.08180604 | -2.3092918 | 2.53E-21 | 1.07E-19 | down |
| UG0898H09\|ENSG00000274956\|lincRNA | 0.52743941 | 0.08265281 | -2.6738696 | 1.27E-19 | 3.32E-18 | down |
| RP5-1096D14.3\|ENSG00000256706\|antisense | 0.37584268 | 0.08268448 | -2.1844404 | 1.95E-14 | 1.86E-13 | down |
| LINC01013\|ENSG00000228495\|lincRNA | 0.3081929 | 0.08319972 | -1.8891831 | 1.40E-20 | 5.04E-19 | down |
| HLX-AS1\|ENSG00000257551\|antisense | 0.30974718 | 0.08477493 | -1.8693816 | 2.15E-15 | 2.44E-14 | down |
| RP11-249C24.11\|ENSG00000259923\|processed_transcript | 0.58420182 | 0.08494648 | -2.7818409 | 2.55E-36 | 6.75E-33 | down |
| RP11-441F2.5\|ENSG00000272372\|antisense | 0.72914871 | 0.08560085 | -3.0905161 | 2.07E-18 | 4.12E-17 | down |
| LINC01571\|ENSG00000260057\|lincRNA | 0.43316867 | 0.08688506 | -2.3177488 | 5.69E-28 | 2.51E-25 | down |
| RP11-863P13.4\|ENSG00000205037\|lincRNA | 0.31252584 | 0.08788579 | -1.8302737 | 3.15E-18 | 6.01E-17 | down |
| RP11-359E19.2\|ENSG00000253665\|lincRNA | 0.41708244 | 0.08794288 | -2.2456939 | 1.92E-14 | 1.85E-13 | down |
| CH17-437K3.1\|ENSG00000227082\|lincRNA | 0.2683832 | 0.08901665 | -1.5921473 | 3.46E-11 | 1.93E-10 | down |
| AC116035.1\|ENSG00000227260\|lincRNA | 0.83858414 | 0.09030425 | -3.2150897 | 1.37E-31 | 1.03E-28 | down |
| RP11-570H19.2\|ENSG00000227167\|lincRNA | 0.25460781 | 0.09054111 | -1.4916318 | 5.45E-17 | 8.31E-16 | down |
| TARID\|ENSG00000227954\|antisense | 0.31870469 | 0.09077957 | -1.8117806 | 2.64E-21 | 1.10E-19 | down |
| RP11-49G2.3\|ENSG00000274624\|sense_intronic | 0.25828341 | 0.09082317 | -1.5078227 | 1.37E-11 | 8.26E-11 | down |
| RP11-88I18.3\|ENSG00000229019\|sense_intronic | 0.32971567 | 0.09124246 | -1.8534452 | 3.31E-23 | 2.47E-21 | down |
| AC018647.3\|ENSG00000227544\|lincRNA | 0.23347747 | 0.09193823 | -1.3445465 | 5.52E-09 | 2.21E-08 | down |
| RP11-342A1.1\|ENSG00000250252\|lincRNA | 0.31473637 | 0.09279234 | -1.7620663 | 1.48E-19 | 3.85E-18 | down |
| RP11-2N1.2\|ENSG00000267774\|antisense | 1.27243713 | 0.09339952 | -3.7680354 | 1.50E-24 | 2.04E-22 | down |
| AF131217.1\|ENSG00000232855\|lincRNA | 0.3303176 | 0.09348575 | -1.8210355 | 4.10E-19 | 9.54E-18 | down |
| RP4-735C1.4\|ENSG00000241720\|antisense | 0.27272957 | 0.09381814 | -1.5395323 | 7.75E-15 | 8.01E-14 | down |
| LINC01354\|ENSG00000231768\|lincRNA | 0.39665609 | 0.09587434 | -2.048672 | 4.01E-17 | 6.25E-16 | down |
| CTD-2135D7.5\|ENSG00000262097\|lincRNA | 0.55892606 | 0.09769193 | -2.5163461 | 1.07E-22 | 6.66E-21 | down |
| LINC01502\|ENSG00000237339\|lincRNA | 0.19833117 | 0.09809854 | -1.0156078 | 0.00820366 | 0.01207653 | down |
| FGF13-AS1\|ENSG00000226031\|antisense | 0.27849882 | 0.09881328 | -1.4948943 | 3.51E-11 | 1.95E-10 | down |
| RP11-963H4.5\|ENSG00000266114\|lincRNA | 0.32445542 | 0.09885391 | -1.7146504 | 2.77E-20 | 9.13E-19 | down |
| AC078941.1\|ENSG00000236780\|lincRNA | 0.41418896 | 0.09904932 | -2.0640701 | 5.54E-05 | 0.00011851 | down |
| LINC01022\|ENSG00000232715\|antisense | 0.26968647 | 0.0992526 | -1.4421064 | 9.30E-11 | 4.87E-10 | down |
| AC007392.4\|ENSG00000237179\|lincRNA | 0.34378085 | 0.09993202 | -1.7824702 | 9.30E-16 | 1.13E-14 | down |
| CASC18\|ENSG00000257859\|lincRNA | 0.4801813 | 0.10020993 | -2.2605537 | 7.29E-15 | 7.56E-14 | down |
| KB-1448A5.1\|ENSG00000253105\|lincRNA | 0.34252848 | 0.10078908 | -1.7648846 | 2.50E-15 | 2.79E-14 | down |
| GAS1RR\|ENSG00000226237\|lincRNA | 0.41953334 | 0.10085449 | -2.0565101 | 1.28E-12 | 9.26E-12 | down |
| RP11-416N2.3\|ENSG00000226431\|sense_intronic | 0.38571989 | 0.10220232 | -1.9161256 | 1.03E-16 | 1.49E-15 | down |
| LINC00402\|ENSG00000235532\|lincRNA | 0.32767992 | 0.10416349 | -1.6534376 | 4.25E-18 | 7.76E-17 | down |
| RP11-326I11.4\|ENSG00000271538\|lincRNA | 0.27373675 | 0.10430235 | -1.3920175 | 6.09E-13 | 4.66E-12 | down |
| RP11-167B3.3\|ENSG00000277987\|lincRNA | 0.25218174 | 0.10506669 | -1.2631584 | 3.44E-06 | 8.89E-06 | down |
| CTA-929C8.7\|ENSG00000223726\|lincRNA | 0.49777819 | 0.10517792 | -2.2426711 | 1.33E-18 | 2.76E-17 | down |
| RP13-514E23.1\|ENSG00000261496\|sense_overlapping | 0.37211922 | 0.10557674 | -1.8174729 | 1.69E-19 | 4.29E-18 | down |
| RP11-885N19.6\|ENSG00000237419\|lincRNA | 0.5198304 | 0.10651975 | -2.2869201 | 6.82E-15 | 7.12E-14 | down |
| RP11-71E19.1\|ENSG00000250934\|lincRNA | 0.28864761 | 0.10691289 | -1.4328735 | 6.78E-11 | 3.63E-10 | down |
| RP11-807H22.7\|ENSG00000204971\|antisense | 0.24546721 | 0.10696083 | -1.1984478 | 1.62E-05 | 3.77E-05 | down |
| RP11-109D9.4\|ENSG00000261447\|lincRNA | 0.21524939 | 0.10706689 | -1.0074967 | 7.38E-10 | 3.34E-09 | down |
| RP11-429O1.1\|ENSG00000263317\|lincRNA | 0.39878972 | 0.1077354 | -1.8881359 | 1.97E-13 | 1.63E-12 | down |
| RP11-89B16.1\|ENSG00000249706\|sense_intronic | 0.24618438 | 0.10791522 | -1.189841 | 2.36E-12 | 1.63E-11 | down |
| FRMD6-AS2\|ENSG00000258537\|lincRNA | 0.68342864 | 0.10800392 | -2.661707 | 4.41E-25 | 8.06E-23 | down |
| RP11-117D22.2\|ENSG00000230138\|lincRNA | 0.24547514 | 0.11018397 | -1.1556627 | 6.29E-09 | 2.49E-08 | down |
| RP11-420N3.3\|ENSG00000279877\|processed_transcript | 0.30801336 | 0.11137229 | -1.4676025 | 4.37E-16 | 5.62E-15 | down |
| RP11-434H14.1\|ENSG00000257277\|antisense | 0.97596649 | 0.11254055 | -3.1163868 | 1.78E-33 | 1.57E-30 | down |
| RP11-264B14.1\|ENSG00000267207\|antisense | 0.30212523 | 0.11337686 | -1.4140205 | 2.46E-25 | 5.22E-23 | down |
| CHL1-AS2\|ENSG00000224318\|processed_transcript | 0.70793538 | 0.11537016 | -2.6173476 | 2.21E-21 | 9.43E-20 | down |
| LINC00693\|ENSG00000228214\|antisense | 0.28296074 | 0.11539371 | -1.2940374 | 6.30E-12 | 4.06E-11 | down |
| CTD-3116E22.8\|ENSG00000277587\|lincRNA | 0.27639097 | 0.11578826 | -1.2552215 | 4.30E-07 | 1.28E-06 | down |
| RP3-368B9.2\|ENSG00000250681\|lincRNA | 0.32742278 | 0.11628402 | -1.4935019 | 6.27E-06 | 1.56E-05 | down |
| RP11-298E9.6\|ENSG00000226762\|lincRNA | 0.34870438 | 0.11661513 | -1.5802495 | 3.95E-20 | 1.22E-18 | down |
| RP11-542M13.3\|ENSG00000268804\|lincRNA | 0.35652754 | 0.11722503 | -1.6047328 | 3.84E-15 | 4.18E-14 | down |
| CTB-186H2.3\|ENSG00000275431\|antisense | 0.29988174 | 0.1192861 | -1.3299678 | 6.80E-16 | 8.46E-15 | down |
| RP11-863P13.5\|ENSG00000261193\|lincRNA | 0.2862159 | 0.12100558 | -1.2420302 | 5.22E-14 | 4.70E-13 | down |
| RP11-124N19.3\|ENSG00000262198\|lincRNA | 0.76487833 | 0.12227205 | -2.6451356 | 1.16E-22 | 7.08E-21 | down |
| CTD-2089N3.1\|ENSG00000250360\|lincRNA | 0.54358485 | 0.12244141 | -2.1504137 | 2.80E-14 | 2.63E-13 | down |
| RP11-396O20.1\|ENSG00000254695\|lincRNA | 1.31530331 | 0.12361999 | -3.4114115 | 2.70E-24 | 3.40E-22 | down |
| AP001058.3\|ENSG00000232698\|lincRNA | 0.28600065 | 0.12388549 | -1.2070113 | 6.71E-08 | 2.31E-07 | down |
| CTC-558O2.2\|ENSG00000254192\|antisense | 0.46146176 | 0.12600026 | -1.8727845 | 7.96E-18 | 1.39E-16 | down |
| RP11-361L15.4\|ENSG00000265408\|processed_transcript | 0.47474684 | 0.12635776 | -1.9096442 | 1.86E-15 | 2.14E-14 | down |
| RP11-542B15.1\|ENSG00000203585\|lincRNA | 0.36553804 | 0.12701699 | -1.5250001 | 1.22E-14 | 1.22E-13 | down |
| RP11-1134I14.8\|ENSG00000255366\|lincRNA | 0.30480549 | 0.12734867 | -1.259105 | 2.50E-12 | 1.72E-11 | down |
| AC090044.2\|ENSG00000224239\|lincRNA | 0.44035744 | 0.12805678 | -1.7818914 | 5.91E-06 | 1.47E-05 | down |
| CTD-2298J14.2\|ENSG00000258636\|lincRNA | 0.26422796 | 0.12846194 | -1.0404421 | 2.07E-08 | 7.57E-08 | down |
| AC006116.24\|ENSG00000267776\|sense_intronic | 0.27318914 | 0.12851972 | -1.0879103 | 5.94E-12 | 3.85E-11 | down |
| RP11-350G8.5\|ENSG00000228013\|antisense | 0.43986058 | 0.12880066 | -1.7719063 | 3.56E-20 | 1.13E-18 | down |
| AC007392.3\|ENSG00000232046\|lincRNA | 0.39536052 | 0.13017496 | -1.6027169 | 9.12E-12 | 5.70E-11 | down |
| AC109309.4\|ENSG00000224513\|antisense | 0.61816516 | 0.1305999 | -2.2428385 | 2.26E-19 | 5.55E-18 | down |
| ADAMTS9-AS2\|ENSG00000241684\|antisense | 0.56393619 | 0.13169007 | -2.0983854 | 4.23E-19 | 9.79E-18 | down |
| LINC01537\|ENSG00000227467\|lincRNA | 0.51260793 | 0.13199922 | -1.9573264 | 6.41E-18 | 1.15E-16 | down |
| AC002398.12\|ENSG00000267328\|antisense | 1.40698714 | 0.13214287 | -3.4124386 | 9.34E-17 | 1.36E-15 | down |
| LRRC3-AS1\|ENSG00000229356\|antisense | 0.27333532 | 0.13253508 | -1.0442976 | 0.02007464 | 0.02756169 | down |
| TMEM5-AS1\|ENSG00000255850\|antisense | 0.28621325 | 0.13277545 | -1.1081021 | 0.00050132 | 0.00091575 | down |
| RP11-268P4.4\|ENSG00000257023\|antisense | 0.40094692 | 0.13292053 | -1.5928473 | 2.75E-05 | 6.16E-05 | down |
| AC010136.2\|ENSG00000223923\|antisense | 0.30254038 | 0.13302497 | -1.1854306 | 1.47E-11 | 8.79E-11 | down |
| RP11-384P7.7\|ENSG00000260947\|lincRNA | 0.92820413 | 0.13486968 | -2.7828761 | 4.19E-20 | 1.27E-18 | down |
| LINC00877\|ENSG00000241163\|lincRNA | 0.30983537 | 0.13525444 | -1.1958259 | 2.67E-12 | 1.83E-11 | down |
| LINC00473\|ENSG00000223414\|lincRNA | 0.34402737 | 0.13643855 | -1.334272 | 0.00118203 | 0.00201606 | down |
| RP11-384F7.2\|ENSG00000239268\|lincRNA | 0.53659774 | 0.13696977 | -1.9699835 | 7.19E-36 | 1.27E-32 | down |
| RP11-470M17.2\|ENSG00000254143\|lincRNA | 0.55230716 | 0.13698728 | -2.0114289 | 1.66E-10 | 8.33E-10 | down |
| AC073316.1\|ENSG00000236708\|antisense | 0.34027845 | 0.13704026 | -1.312116 | 3.07E-11 | 1.73E-10 | down |
| AF064860.7\|ENSG00000231713\|lincRNA | 0.54840684 | 0.13760407 | -1.9947235 | 1.05E-12 | 7.69E-12 | down |
| RP11-259O2.1\|ENSG00000248994\|lincRNA | 0.29663924 | 0.1378175 | -1.1059504 | 3.21E-14 | 2.99E-13 | down |
| RP11-109E24.2\|ENSG00000260519\|antisense | 0.40318232 | 0.13825638 | -1.5440863 | 1.87E-12 | 1.32E-11 | down |
| RP11-573G6.6\|ENSG00000261671\|lincRNA | 0.36418312 | 0.13844942 | -1.3953051 | 2.23E-13 | 1.82E-12 | down |
| RP11-350N15.3\|ENSG00000254981\|antisense | 0.32802288 | 0.13896247 | -1.2391011 | 1.38E-05 | 3.24E-05 | down |
| RP11-543C4.1\|ENSG00000247970\|lincRNA | 0.33804792 | 0.13926291 | -1.2794167 | 1.36E-14 | 1.35E-13 | down |
| RP11-239H6.2\|ENSG00000227803\|antisense | 0.45583866 | 0.1392846 | -1.7104876 | 6.96E-16 | 8.63E-15 | down |
| RP11-299L17.3\|ENSG00000258675\|lincRNA | 0.28554998 | 0.14008989 | -1.0273905 | 4.24E-08 | 1.49E-07 | down |
| RP11-440I14.2\|ENSG00000250596\|lincRNA | 0.4709605 | 0.14160441 | -1.7337399 | 1.37E-18 | 2.85E-17 | down |
| RP11-203E8.1\|ENSG00000253891\|lincRNA | 0.28589903 | 0.14256842 | -1.0038513 | 1.87E-05 | 4.29E-05 | down |
| RP11-863P13.6\|ENSG00000260166\|lincRNA | 0.59104715 | 0.14259759 | -2.0513236 | 1.74E-19 | 4.40E-18 | down |
| RP11-900F13.2\|ENSG00000278011\|lincRNA | 0.34748076 | 0.14269674 | -1.2839807 | 5.13E-09 | 2.07E-08 | down |
| AC092652.1\|ENSG00000231758\|antisense | 0.87040316 | 0.14385428 | -2.5970757 | 3.03E-35 | 3.21E-32 | down |
| RP11-710C12.1\|ENSG00000271474\|antisense | 0.40781214 | 0.14426897 | -1.4991437 | 2.64E-19 | 6.42E-18 | down |
| CADM3-AS1\|ENSG00000225670\|antisense | 0.51650977 | 0.14435714 | -1.8391532 | 6.41E-17 | 9.62E-16 | down |
| RP11-342A23.2\|ENSG00000274128\|sense_intronic | 0.62055425 | 0.14445374 | -2.1029498 | 1.34E-15 | 1.59E-14 | down |
| LINC01055\|ENSG00000235366\|lincRNA | 0.68223949 | 0.1449429 | -2.2347936 | 4.24E-16 | 5.49E-15 | down |
| AF064860.5\|ENSG00000225330\|sense_overlapping | 0.3977654 | 0.14579492 | -1.4479773 | 2.14E-08 | 7.81E-08 | down |
| RP11-266K4.14\|ENSG00000275367\|lincRNA | 0.29993324 | 0.14691231 | -1.0296862 | 2.89E-09 | 1.21E-08 | down |
| RP11-387A1.5\|ENSG00000272729\|antisense | 0.42691559 | 0.14712117 | -1.536946 | 2.16E-13 | 1.77E-12 | down |
| RP3-422G23.4\|ENSG00000262543\|lincRNA | 1.48079155 | 0.14823026 | -3.3204566 | 4.01E-26 | 1.33E-23 | down |
| RP11-536C5.2\|ENSG00000225279\|antisense | 0.52175268 | 0.15128409 | -1.7861059 | 1.24E-14 | 1.23E-13 | down |
| RP11-344P13.6\|ENSG00000272583\|lincRNA | 0.37729842 | 0.15209874 | -1.3106979 | 1.30E-12 | 9.44E-12 | down |
| LYPLAL1-AS1\|ENSG00000228063\|antisense | 0.41538832 | 0.15220419 | -1.4484525 | 4.57E-06 | 1.16E-05 | down |
| RP11-307B6.3\|ENSG00000223774\|antisense | 1.21410896 | 0.15296318 | -2.9886416 | 8.74E-15 | 8.88E-14 | down |
| AC016995.3\|ENSG00000231367\|lincRNA | 0.63387623 | 0.15320496 | -2.0487381 | 2.75E-08 | 9.87E-08 | down |
| RP11-400K9.3\|ENSG00000254204\|lincRNA | 0.74703138 | 0.15562546 | -2.2630908 | 8.80E-13 | 6.57E-12 | down |
| RP11-521M14.1\|ENSG00000253888\|antisense | 0.34197769 | 0.15690254 | -1.1240335 | 5.35E-09 | 2.14E-08 | down |
| RP11-182J1.1\|ENSG00000254414\|antisense | 0.41485734 | 0.15849061 | -1.3882179 | 3.16E-11 | 1.77E-10 | down |
| CCDC13-AS1\|ENSG00000173811\|antisense | 0.32330915 | 0.16027558 | -1.0123597 | 3.12E-09 | 1.30E-08 | down |
| CYP1B1-AS1\|ENSG00000232973\|antisense | 0.41526918 | 0.16041831 | -1.372208 | 1.24E-18 | 2.62E-17 | down |
| RP11-108M9.3\|ENSG00000228549\|lincRNA | 0.48136031 | 0.16064136 | -1.5832738 | 4.18E-09 | 1.71E-08 | down |
| RP11-713P17.3\|ENSG00000204241\|lincRNA | 0.45059848 | 0.16086784 | -1.4859665 | 4.52E-09 | 1.84E-08 | down |
| RP11-820I16.3\|ENSG00000266955\|antisense | 0.33906336 | 0.16143978 | -1.0705588 | 1.43E-12 | 1.03E-11 | down |
| LINC01140\|ENSG00000267272\|lincRNA | 0.49800036 | 0.16278263 | -1.6132 | 3.05E-19 | 7.29E-18 | down |
| RP11-640L9.1\|ENSG00000279727\|lincRNA | 0.62859568 | 0.16301892 | -1.9470929 | 1.55E-19 | 3.99E-18 | down |
| LINC01018\|ENSG00000250056\|lincRNA | 0.44076579 | 0.16302334 | -1.4349337 | 1.71E-10 | 8.55E-10 | down |
| RP11-367J11.3\|ENSG00000245468\|lincRNA | 0.396969 | 0.16310452 | -1.2832296 | 8.50E-15 | 8.66E-14 | down |
| RP3-400B16.1\|ENSG00000234817\|lincRNA | 0.4715064 | 0.16343929 | -1.5285225 | 4.46E-17 | 6.93E-16 | down |
| RP11-855A2.5\|ENSG00000266176\|lincRNA | 2.42460623 | 0.16355765 | -3.8898794 | 5.43E-29 | 2.62E-26 | down |
| RP11-281P23.2\|ENSG00000249631\|lincRNA | 0.39686795 | 0.16515907 | -1.2648029 | 8.05E-12 | 5.07E-11 | down |
| RP11-64C12.8\|ENSG00000267069\|lincRNA | 0.44532116 | 0.16577593 | -1.4256116 | 0.00284398 | 0.00454916 | down |
| LINC00955\|ENSG00000216560\|processed_transcript | 1.44677983 | 0.16583329 | -3.1250398 | 1.86E-11 | 1.08E-10 | down |
| RERG-IT1\|ENSG00000256650\|sense_intronic | 0.54633151 | 0.16634287 | -1.7156167 | 2.98E-11 | 1.68E-10 | down |
| HCG23\|ENSG00000228962\|antisense | 0.91137849 | 0.1665348 | -2.4522267 | 3.13E-20 | 1.01E-18 | down |
| AP000230.1\|ENSG00000273492\|lincRNA | 0.37606002 | 0.16791679 | -1.1632164 | 6.37E-12 | 4.09E-11 | down |
| RP11-380P13.1\|ENSG00000250137\|antisense | 1.31461006 | 0.16856173 | -2.963286 | 5.16E-25 | 8.55E-23 | down |
| RP11-295M18.6\|ENSG00000272823\|lincRNA | 0.49150476 | 0.16859906 | -1.5436088 | 1.17E-13 | 9.98E-13 | down |
| LINC01352\|ENSG00000238078\|lincRNA | 0.60388591 | 0.17266895 | -1.8062673 | 9.27E-17 | 1.35E-15 | down |
| RP11-753H16.3\|ENSG00000258137\|antisense | 1.80838178 | 0.17286164 | -3.3870097 | 2.41E-09 | 1.02E-08 | down |
| AP000472.2\|ENSG00000233215\|lincRNA | 1.33317417 | 0.17350377 | -2.9418264 | 4.78E-25 | 8.44E-23 | down |
| RP11-374M1.5\|ENSG00000235659\|lincRNA | 0.61654464 | 0.17538424 | -1.8136862 | 1.09E-11 | 6.71E-11 | down |
| RP1-193H18.3\|ENSG00000267653\|lincRNA | 0.49285858 | 0.17574537 | -1.4876871 | 2.38E-18 | 4.67E-17 | down |
| RP11-526A4.1\|ENSG00000234828\|lincRNA | 0.67350382 | 0.17670487 | -1.9303443 | 6.90E-19 | 1.54E-17 | down |
| VLDLR-AS1\|ENSG00000236404\|antisense | 0.47587408 | 0.17700126 | -1.4268202 | 1.52E-09 | 6.62E-09 | down |
| XXbac-BPG13B8.10\|ENSG00000229274\|lincRNA | 1.32103236 | 0.1773037 | -2.8973712 | 6.40E-25 | 9.99E-23 | down |
| RP11-725G5.2\|ENSG00000258630\|lincRNA | 0.67764657 | 0.17843091 | -1.9251675 | 5.34E-18 | 9.69E-17 | down |
| RP11-960L18.1\|ENSG00000261218\|lincRNA | 0.44284843 | 0.17849388 | -1.3109383 | 1.99E-11 | 1.15E-10 | down |
| RP11-867O8.5\|ENSG00000255478\|antisense | 0.96777876 | 0.17948971 | -2.4307762 | 4.38E-16 | 5.62E-15 | down |
| AC009133.17\|ENSG00000260719\|antisense | 0.94005745 | 0.18104917 | -2.3763674 | 4.08E-20 | 1.25E-18 | down |
| RP11-365O16.3\|ENSG00000232628\|sense_intronic | 0.4275016 | 0.18219746 | -1.230427 | 2.72E-09 | 1.15E-08 | down |
| LINC01399\|ENSG00000233080\|lincRNA | 0.50702095 | 0.18221039 | -1.4764401 | 1.24E-07 | 4.06E-07 | down |
| RP11-685G9.2\|ENSG00000259514\|antisense | 0.48224549 | 0.1831548 | -1.3967042 | 1.67E-10 | 8.38E-10 | down |
| RP11-554A11.5\|ENSG00000261276\|antisense | 0.70708975 | 0.18608224 | -1.925953 | 2.11E-07 | 6.65E-07 | down |
| RP11-202A13.1\|ENSG00000243832\|lincRNA | 2.03892265 | 0.18635651 | -3.4516699 | 3.78E-26 | 1.33E-23 | down |
| RP11-474N24.6\|ENSG00000272799\|lincRNA | 0.43903287 | 0.18652448 | -1.234964 | 1.24E-10 | 6.30E-10 | down |
| RP11-779O18.3\|ENSG00000253736\|antisense | 0.66933259 | 0.18732042 | -1.8372151 | 1.46E-16 | 2.06E-15 | down |
| LINC01197\|ENSG00000248441\|lincRNA | 0.41853935 | 0.18912061 | -1.1460567 | 9.76E-13 | 7.22E-12 | down |
| CTC-558O2.1\|ENSG00000254042\|antisense | 0.74559071 | 0.1904784 | -1.9687565 | 1.02E-18 | 2.21E-17 | down |
| AC007556.3\|ENSG00000235321\|antisense | 1.34571669 | 0.19515149 | -2.7857084 | 7.93E-26 | 2.40E-23 | down |
| OSTN-AS1\|ENSG00000233308\|antisense | 1.15899595 | 0.19605896 | -2.563516 | 3.73E-18 | 7.02E-17 | down |
| RP11-94C24.13\|ENSG00000275897\|lincRNA | 0.90213095 | 0.20010934 | -2.1725483 | 5.02E-20 | 1.49E-18 | down |
| RP11-432I5.8\|ENSG00000261513\|antisense | 0.7156115 | 0.20122833 | -1.8303431 | 2.10E-12 | 1.47E-11 | down |
| LINC01394\|ENSG00000281809\|lincRNA | 0.68296255 | 0.20314991 | -1.7492617 | 1.96E-14 | 1.87E-13 | down |
| RP11-225H22.4\|ENSG00000234699\|antisense | 0.45345679 | 0.20540629 | -1.1424848 | 1.98E-09 | 8.50E-09 | down |
| LL22NC03-75H12.2\|ENSG00000218357\|lincRNA | 0.53222806 | 0.20545764 | -1.3732036 | 4.65E-12 | 3.07E-11 | down |
| CTD-3193O13.1\|ENSG00000260500\|antisense | 0.72459417 | 0.20622632 | -1.8129447 | 7.82E-16 | 9.57E-15 | down |
| CH17-360D5.1\|ENSG00000264717\|lincRNA | 0.62966406 | 0.20688423 | -1.6057587 | 2.81E-11 | 1.59E-10 | down |
| RBMS3-AS3\|ENSG00000235904\|antisense | 0.89798175 | 0.2071343 | -2.1161197 | 5.52E-21 | 2.17E-19 | down |
| LINC01336\|ENSG00000250889\|lincRNA | 0.48917026 | 0.2085175 | -1.2301682 | 3.55E-09 | 1.47E-08 | down |
| FLJ31104\|ENSG00000227908\|antisense | 0.51776222 | 0.21607825 | -1.2607358 | 1.21E-14 | 1.21E-13 | down |
| CTC-378H22.2\|ENSG00000259436\|antisense | 0.57037148 | 0.22261404 | -1.3573573 | 6.19E-14 | 5.52E-13 | down |
| LINC00092\|ENSG00000225194\|lincRNA | 1.16082971 | 0.22314902 | -2.379077 | 4.18E-24 | 4.88E-22 | down |
| RP11-354E11.2\|ENSG00000233968\|antisense | 0.61721743 | 0.2293523 | -1.4282134 | 3.59E-14 | 3.32E-13 | down |
| RP4-668J24.2\|ENSG00000261730\|antisense | 0.8445425 | 0.22996732 | -1.8767412 | 1.00E-18 | 2.19E-17 | down |
| RP11-209E8.1\|ENSG00000259237\|lincRNA | 5.21532132 | 0.23010363 | -4.5024005 | 4.46E-24 | 4.93E-22 | down |
| RP11-678G14.3\|ENSG00000268555\|lincRNA | 2.25601887 | 0.23061407 | -3.2902267 | 1.58E-20 | 5.55E-19 | down |
| HID1-AS1\|ENSG00000263586\|antisense | 0.53638345 | 0.23202698 | -1.2089722 | 7.25E-11 | 3.87E-10 | down |
| CTD-2308L22.1\|ENSG00000272944\|antisense | 0.61281678 | 0.23315945 | -1.3941389 | 1.69E-14 | 1.65E-13 | down |
| MAFTRR\|ENSG00000261390\|lincRNA | 0.47842281 | 0.23337263 | -1.0356508 | 3.15E-10 | 1.52E-09 | down |
| AP001627.1\|ENSG00000225731\|antisense | 2.01515667 | 0.2353249 | -3.0981661 | 1.60E-17 | 2.67E-16 | down |
| AC025165.8\|ENSG00000224713\|antisense | 0.53432042 | 0.2362686 | -1.1772773 | 5.52E-07 | 1.62E-06 | down |
| RP11-326K13.5\|ENSG00000274275\|sense_intronic | 0.60565529 | 0.23666009 | -1.3556805 | 2.55E-13 | 2.06E-12 | down |
| RP11-834C11.8\|ENSG00000248576\|antisense | 0.53709272 | 0.23738625 | -1.1779348 | 1.07E-14 | 1.07E-13 | down |
| RP11-489O18.1\|ENSG00000253988\|lincRNA | 0.57246386 | 0.23765311 | -1.2683273 | 2.44E-11 | 1.39E-10 | down |
| RP11-747D18.1\|ENSG00000239205\|lincRNA | 2.97305522 | 0.24004535 | -3.6305674 | 5.65E-23 | 3.99E-21 | down |
| RP11-430C7.4\|ENSG00000240710\|antisense | 0.80347804 | 0.24339285 | -1.7229718 | 4.81E-17 | 7.38E-16 | down |
| CTA-150C2.13\|ENSG00000235209\|lincRNA | 1.49270692 | 0.24365056 | -2.6150455 | 3.78E-20 | 1.18E-18 | down |
| ZFY-AS1\|ENSG00000233070\|antisense | 0.55330982 | 0.24409269 | -1.1806585 | 0.00755659 | 0.01119549 | down |
| IFNG-AS1\|ENSG00000255733\|antisense | 0.52732298 | 0.24625646 | -1.0985253 | 7.72E-08 | 2.62E-07 | down |
| AC004791.2\|ENSG00000267453\|lincRNA | 0.6688658 | 0.24653061 | -1.43995 | 2.94E-07 | 9.01E-07 | down |
| RP5-965F6.2\|ENSG00000231246\|lincRNA | 1.14824026 | 0.24902899 | -2.2050389 | 2.15E-21 | 9.28E-20 | down |
| RP11-126O1.6\|ENSG00000275418\|lincRNA | 1.10009746 | 0.24954335 | -2.140269 | 6.73E-17 | 1.01E-15 | down |
| RP1-161P9.5\|ENSG00000271392\|sense_intronic | 0.70975809 | 0.24962787 | -1.5075484 | 2.11E-15 | 2.40E-14 | down |
| CTB-118N6.2\|ENSG00000249167\|lincRNA | 1.28843527 | 0.24978638 | -2.3668533 | 8.67E-23 | 5.54E-21 | down |
| CTD-2541M15.3\|ENSG00000271743\|lincRNA | 0.51623397 | 0.25154331 | -1.0372182 | 6.76E-13 | 5.15E-12 | down |
| RP11-320N21.2\|ENSG00000254248\|antisense | 1.33203734 | 0.25420607 | -2.3895642 | 4.67E-16 | 5.92E-15 | down |
| FOXD3-AS1\|ENSG00000230798\|antisense | 1.22585411 | 0.25431638 | -2.269091 | 1.01E-17 | 1.73E-16 | down |
| CTC-756D1.2\|ENSG00000253390\|lincRNA | 0.53925282 | 0.25589743 | -1.0753962 | 2.31E-09 | 9.86E-09 | down |
| AJ011932.1\|ENSG00000274248\|lincRNA | 0.63370507 | 0.25679328 | -1.3032041 | 1.47E-08 | 5.48E-08 | down |
| RP11-459C13.1\|ENSG00000263312\|lincRNA | 1.33208586 | 0.2583229 | -2.3664396 | 6.60E-21 | 2.56E-19 | down |
| LINC01550\|ENSG00000246223\|lincRNA | 0.62930579 | 0.26459931 | -1.2499519 | 6.42E-16 | 8.00E-15 | down |
| RP4-758J18.7\|ENSG00000225905\|antisense | 0.59165998 | 0.2649405 | -1.1590999 | 4.43E-07 | 1.32E-06 | down |
| RP11-567C2.1\|ENSG00000257194\|lincRNA | 1.43821688 | 0.2650885 | -2.4397353 | 9.78E-19 | 2.15E-17 | down |
| RP11-805I24.3\|ENSG00000268505\|lincRNA | 1.15700523 | 0.26557722 | -2.1231921 | 7.67E-23 | 5.08E-21 | down |
| DKFZp779M0652\|ENSG00000205106\|lincRNA | 0.93259596 | 0.26564929 | -1.8117293 | 4.30E-16 | 5.55E-15 | down |
| RP11-167N24.6\|ENSG00000258247\|lincRNA | 5.78522663 | 0.26611264 | -4.4422645 | 1.48E-18 | 3.04E-17 | down |
| MIR497HG\|ENSG00000267532\|antisense | 1.1904588 | 0.26664318 | -2.1585354 | 9.61E-21 | 3.64E-19 | down |
| RP11-733O18.1\|ENSG00000236120\|lincRNA | 1.67161869 | 0.27022298 | -2.6290235 | 3.67E-22 | 2.03E-20 | down |
| LINC00930\|ENSG00000258647\|lincRNA | 0.56760385 | 0.27130173 | -1.0649861 | 8.54E-15 | 8.69E-14 | down |
| RP11-359E10.1\|ENSG00000270607\|lincRNA | 0.75888206 | 0.27221754 | -1.4791157 | 8.87E-07 | 2.52E-06 | down |
| AP001347.6\|ENSG00000224905\|antisense | 0.55621205 | 0.27432547 | -1.0197464 | 0.00034517 | 0.00065139 | down |
| C20orf166-AS1\|ENSG00000174403\|antisense | 2.189098 | 0.27570254 | -2.9891521 | 2.13E-20 | 7.30E-19 | down |
| RP11-305L7.6\|ENSG00000229694\|lincRNA | 1.30617304 | 0.27819828 | -2.2311606 | 3.84E-22 | 2.10E-20 | down |
| AC074363.1\|ENSG00000228222\|lincRNA | 2.22854589 | 0.27887082 | -2.9984338 | 1.70E-25 | 4.30E-23 | down |
| AC003991.3\|ENSG00000228113\|antisense | 0.58104461 | 0.27995289 | -1.0534649 | 1.60E-13 | 1.34E-12 | down |
| AL122127.25\|ENSG00000244620\|lincRNA | 0.94037504 | 0.2801058 | -1.7472644 | 2.70E-14 | 2.54E-13 | down |
| RP11-320N21.1\|ENSG00000245080\|antisense | 1.31697392 | 0.28142376 | -2.2264107 | 1.07E-15 | 1.29E-14 | down |
| AC007182.6\|ENSG00000224721\|antisense | 5.25076351 | 0.28211418 | -4.2181762 | 2.00E-25 | 4.75E-23 | down |
| CASC16\|ENSG00000249231\|lincRNA | 0.70429566 | 0.2826795 | -1.3170139 | 1.01E-12 | 7.41E-12 | down |
| RP5-1125N11.2\|ENSG00000223382\|lincRNA | 0.85702596 | 0.28506229 | -1.5880617 | 0.00063913 | 0.00114653 | down |
| RP11-844P9.2\|ENSG00000248596\|lincRNA | 0.57263103 | 0.28533674 | -1.0049403 | 2.73E-12 | 1.86E-11 | down |
| RP11-384O8.1\|ENSG00000267034\|lincRNA | 0.72661557 | 0.28549158 | -1.3477441 | 2.04E-07 | 6.43E-07 | down |
| U62631.5\|ENSG00000269553\|sense_intronic | 0.83111495 | 0.29183115 | -1.5099142 | 6.80E-08 | 2.34E-07 | down |
| RP1-170O19.23\|ENSG00000272801\|processed_transcript | 0.61251462 | 0.2925046 | -1.066285 | 1.11E-06 | 3.11E-06 | down |
| RP11-1069G10.1\|ENSG00000259370\|antisense | 1.04477972 | 0.29446353 | -1.8270379 | 2.07E-17 | 3.39E-16 | down |
| ADAMTS9-AS1\|ENSG00000241158\|antisense | 1.85451467 | 0.29804801 | -2.637425 | 4.98E-15 | 5.33E-14 | down |
| AC106869.2\|ENSG00000226087\|lincRNA | 3.75920753 | 0.29889804 | -3.6527032 | 1.35E-26 | 5.51E-24 | down |
| HAND2-AS1\|ENSG00000237125\|antisense | 5.26949761 | 0.30026278 | -4.1333679 | 1.63E-19 | 4.17E-18 | down |
| RP11-527N22.1\|ENSG00000253123\|processed_transcript | 0.61341746 | 0.30173978 | -1.0235643 | 2.11E-10 | 1.04E-09 | down |
| RP11-12M5.3\|ENSG00000229407\|antisense | 0.92779252 | 0.30291919 | -1.6148693 | 8.58E-13 | 6.43E-12 | down |
| CTD-2054N24.2\|ENSG00000259363\|lincRNA | 0.76980836 | 0.30441948 | -1.3384387 | 1.83E-12 | 1.29E-11 | down |
| RP11-713N11.6\|ENSG00000276842\|antisense | 0.69651065 | 0.31088628 | -1.1637585 | 6.21E-12 | 4.01E-11 | down |
| XXyac-YM21GA2.4\|ENSG00000234460\|lincRNA | 1.29664702 | 0.31117983 | -2.0589653 | 2.07E-19 | 5.10E-18 | down |
| LINC01160\|ENSG00000231346\|lincRNA | 0.70120859 | 0.31155434 | -1.1703599 | 4.61E-05 | 9.98E-05 | down |
| AF186192.1\|ENSG00000254533\|lincRNA | 0.9328893 | 0.31421541 | -1.569952 | 1.47E-16 | 2.08E-15 | down |
| RP11-129J12.1\|ENSG00000228778\|lincRNA | 1.26084716 | 0.31530777 | -1.9995608 | 2.50E-16 | 3.39E-15 | down |
| RP11-285G1.9\|ENSG00000223462\|lincRNA | 1.89540272 | 0.3159606 | -2.5846878 | 1.20E-22 | 7.22E-21 | down |
| LINC01351\|ENSG00000237457\|lincRNA | 1.93440957 | 0.31979579 | -2.5966704 | 2.90E-29 | 1.54E-26 | down |
| RP11-367F23.1\|ENSG00000228216\|lincRNA | 2.2133481 | 0.32181441 | -2.7819295 | 2.10E-05 | 4.79E-05 | down |
| RP11-13K12.1\|ENSG00000267506\|lincRNA | 0.88295208 | 0.32977856 | -1.4208375 | 2.54E-10 | 1.23E-09 | down |
| PP14571\|ENSG00000218416\|processed_transcript | 0.79015682 | 0.33259295 | -1.2483814 | 1.85E-10 | 9.21E-10 | down |
| AC096579.13\|ENSG00000240040\|lincRNA | 0.96858149 | 0.33342041 | -1.538531 | 7.75E-13 | 5.84E-12 | down |
| RP11-285E9.6\|ENSG00000263718\|lincRNA | 0.73969029 | 0.34189239 | -1.113379 | 7.51E-12 | 4.76E-11 | down |
| RP11-375H19.2\|ENSG00000203307\|antisense | 0.99749087 | 0.34581962 | -1.5282839 | 1.92E-11 | 1.11E-10 | down |
| DLG1-AS1\|ENSG00000227375\|antisense | 0.72553349 | 0.34774626 | -1.0610072 | 1.99E-13 | 1.64E-12 | down |
| LINC01135\|ENSG00000234807\|lincRNA | 1.04614868 | 0.34927545 | -1.5826508 | 3.08E-19 | 7.32E-18 | down |
| RP11-404E16.1\|ENSG00000250734\|antisense | 1.01658963 | 0.35580377 | -1.5145837 | 2.02E-06 | 5.43E-06 | down |
| AC004637.1\|ENSG00000267304\|antisense | 1.09992002 | 0.35642465 | -1.6257296 | 1.24E-10 | 6.33E-10 | down |
| RP11-1000B6.3\|ENSG00000261064\|lincRNA | 0.82063633 | 0.36439456 | -1.1712416 | 3.80E-11 | 2.09E-10 | down |
| AC002451.3\|ENSG00000231170\|antisense | 0.76389146 | 0.36550859 | -1.0634624 | 7.49E-11 | 3.97E-10 | down |
| RP4-800J21.3\|ENSG00000218018\|antisense | 0.84048444 | 0.36618209 | -1.1986599 | 6.34E-08 | 2.19E-07 | down |
| RP11-70D24.2\|ENSG00000278058\|lincRNA | 0.94761531 | 0.36821956 | -1.3637353 | 5.41E-13 | 4.19E-12 | down |
| AC145124.2\|ENSG00000255495\|antisense | 0.77635426 | 0.36890272 | -1.0734747 | 2.51E-10 | 1.22E-09 | down |
| GS1-204I12.4\|ENSG00000261729\|lincRNA | 1.19472651 | 0.37144367 | -1.6854651 | 9.14E-12 | 5.71E-11 | down |
| LINC00940\|ENSG00000235049\|lincRNA | 1.91932769 | 0.37232324 | -2.3659735 | 4.69E-19 | 1.08E-17 | down |
| RP11-404P21.3\|ENSG00000258793\|antisense | 0.78907954 | 0.37340253 | -1.079439 | 5.31E-14 | 4.77E-13 | down |
| RP11-182J1.17\|ENSG00000275120\|antisense | 0.83302477 | 0.37402923 | -1.1552084 | 9.47E-10 | 4.24E-09 | down |
| LINC00892\|ENSG00000233093\|processed_transcript | 0.81780262 | 0.37489278 | -1.1252746 | 2.55E-11 | 1.45E-10 | down |
| LINC01475\|ENSG00000257582\|lincRNA | 1.56099629 | 0.37589948 | -2.0540483 | 8.63E-18 | 1.50E-16 | down |
| CTD-2269F5.1\|ENSG00000250320\|antisense | 1.65093553 | 0.37867242 | -2.1242615 | 1.49E-21 | 6.83E-20 | down |
| RP11-230B22.1\|ENSG00000235545\|antisense | 0.94978578 | 0.37991406 | -1.3219291 | 5.29E-18 | 9.63E-17 | down |
| RP11-131H24.4\|ENSG00000258987\|antisense | 2.56222384 | 0.38613194 | -2.7302307 | 9.24E-24 | 8.91E-22 | down |
| RP1-35C21.1\|ENSG00000224968\|lincRNA | 6.72024738 | 0.38670963 | -4.1191917 | 2.51E-24 | 3.25E-22 | down |
| RP11-1260E13.4\|ENSG00000262061\|processed_transcript | 0.88969157 | 0.38976657 | -1.1906949 | 5.93E-13 | 4.56E-12 | down |
| RP4-633O19__A.1\|ENSG00000223695\|lincRNA | 1.0123314 | 0.39203823 | -1.3686154 | 8.33E-13 | 6.26E-12 | down |
| RP11-148O21.2\|ENSG00000255354\|antisense | 1.19471908 | 0.39514683 | -1.5962107 | 3.05E-12 | 2.07E-11 | down |
| RP11-616M22.3\|ENSG00000261294\|antisense | 0.91428764 | 0.39616423 | -1.2065495 | 5.36E-09 | 2.15E-08 | down |
| RP11-597M12.2\|ENSG00000277621\|antisense | 0.89187764 | 0.3984104 | -1.1625905 | 7.96E-08 | 2.70E-07 | down |
| RP5-887A10.1\|ENSG00000234184\|lincRNA | 1.59986983 | 0.40317265 | -1.9884848 | 3.64E-16 | 4.79E-15 | down |
| RP1-117B12.4\|ENSG00000253102\|antisense | 2.15355926 | 0.40415766 | -2.4137329 | 1.23E-22 | 7.35E-21 | down |
| RP11-875O11.1\|ENSG00000245025\|antisense | 1.04770698 | 0.40643974 | -1.3661219 | 1.68E-14 | 1.63E-13 | down |
| CTC-296K1.4\|ENSG00000267405\|lincRNA | 2.92550393 | 0.4090612 | -2.8382965 | 2.75E-21 | 1.14E-19 | down |
| RP11-514D23.2\|ENSG00000268754\|lincRNA | 1.16715721 | 0.41383687 | -1.4958648 | 7.65E-17 | 1.14E-15 | down |
| RP11-798K3.2\|ENSG00000259347\|lincRNA | 1.6429687 | 0.42180123 | -1.9616698 | 3.10E-10 | 1.50E-09 | down |
| RP11-89K11.1\|ENSG00000259658\|lincRNA | 0.93804355 | 0.42230591 | -1.1513665 | 1.83E-07 | 5.82E-07 | down |
| RP11-1070N10.3\|ENSG00000258572\|lincRNA | 1.10077485 | 0.42434718 | -1.3752024 | 9.47E-12 | 5.88E-11 | down |
| RP11-616M22.5\|ENSG00000260182\|sense_intronic | 1.35840868 | 0.42613132 | -1.6725476 | 1.54E-07 | 4.99E-07 | down |
| AC093627.8\|ENSG00000242611\|lincRNA | 2.6283487 | 0.42709377 | -2.6215319 | 9.05E-14 | 7.89E-13 | down |
| RP11-251M1.1\|ENSG00000228401\|antisense | 1.08594449 | 0.4282558 | -1.3424057 | 2.71E-16 | 3.65E-15 | down |
| RP11-89C3.4\|ENSG00000271584\|lincRNA | 0.9884696 | 0.43550227 | -1.1825164 | 6.96E-08 | 2.39E-07 | down |
| ZNF582-AS1\|ENSG00000267454\|lincRNA | 1.27406309 | 0.43869718 | -1.5381394 | 2.77E-17 | 4.46E-16 | down |
| LINC00702\|ENSG00000233117\|lincRNA | 1.05565051 | 0.43940733 | -1.2645015 | 8.92E-14 | 7.79E-13 | down |
| LINC01215\|ENSG00000271856\|lincRNA | 0.98972154 | 0.44237276 | -1.1617601 | 8.28E-08 | 2.80E-07 | down |
| RP11-456K23.1\|ENSG00000267414\|lincRNA | 1.1353502 | 0.4435722 | -1.3558965 | 2.85E-17 | 4.57E-16 | down |
| AF127936.5\|ENSG00000226751\|lincRNA | 1.3075017 | 0.44954509 | -1.5402751 | 1.99E-14 | 1.90E-13 | down |
| CTD-2554C21.2\|ENSG00000267640\|lincRNA | 1.02219718 | 0.44983282 | -1.1842127 | 1.62E-10 | 8.13E-10 | down |
| CTC-297N7.9\|ENSG00000264016\|lincRNA | 2.90891834 | 0.45521296 | -2.6758693 | 5.35E-24 | 5.41E-22 | down |
| RP4-651E10.4\|ENSG00000236915\|antisense | 1.91181522 | 0.46123813 | -2.0513594 | 1.47E-14 | 1.45E-13 | down |
| RP11-448G15.1\|ENSG00000250413\|antisense | 1.52762345 | 0.46446471 | -1.7176481 | 2.45E-15 | 2.74E-14 | down |
| RP11-13K12.5\|ENSG00000267466\|lincRNA | 2.03103379 | 0.46479799 | -2.1275385 | 4.31E-14 | 3.94E-13 | down |
| LINC00582\|ENSG00000229228\|antisense | 2.07732423 | 0.46925982 | -2.1462676 | 9.15E-18 | 1.59E-16 | down |
| CTD-2256P15.5\|ENSG00000271715\|lincRNA | 1.22145402 | 0.47071787 | -1.375665 | 3.62E-12 | 2.42E-11 | down |
| CTB-41I6.1\|ENSG00000266389\|lincRNA | 1.12226496 | 0.47122095 | -1.2519377 | 5.49E-12 | 3.58E-11 | down |
| RP11-293M10.6\|ENSG00000259319\|antisense | 1.36026419 | 0.47748324 | -1.5103649 | 4.02E-23 | 2.88E-21 | down |
| RP11-245G13.2\|ENSG00000271952\|lincRNA | 3.13536748 | 0.48126712 | -2.7037248 | 5.81E-23 | 4.05E-21 | down |
| MCPH1-AS1\|ENSG00000249898\|antisense | 1.07474432 | 0.48916424 | -1.1356026 | 1.49E-17 | 2.49E-16 | down |
| RP11-1336O20.2\|ENSG00000249690\|antisense | 1.38121111 | 0.48977718 | -1.4957364 | 6.81E-15 | 7.12E-14 | down |
| RP11-2I17.4\|ENSG00000260173\|antisense | 3.3813288 | 0.49630411 | -2.768294 | 2.12E-21 | 9.28E-20 | down |
| AC002511.3\|ENSG00000232680\|lincRNA | 1.52211411 | 0.49691557 | -1.6150039 | 6.27E-15 | 6.61E-14 | down |
| RP11-449D8.1\|ENSG00000265485\|lincRNA | 1.05828341 | 0.49735456 | -1.0893794 | 1.97E-12 | 1.38E-11 | down |
| CTD-2588E21.1\|ENSG00000277763\|lincRNA | 1.81889817 | 0.50421891 | -1.8509427 | 9.69E-21 | 3.64E-19 | down |
| AL161668.5\|ENSG00000258604\|lincRNA | 1.14240157 | 0.5053477 | -1.1767216 | 7.47E-12 | 4.74E-11 | down |
| RP11-867G23.10\|ENSG00000254510\|processed_transcript | 3.37368274 | 0.51568031 | -2.7097754 | 2.25E-15 | 2.54E-14 | down |
| ZNF790-AS1\|ENSG00000267254\|antisense | 1.03788161 | 0.51588768 | -1.008513 | 2.44E-14 | 2.30E-13 | down |
| RP11-102N12.3\|ENSG00000273472\|lincRNA | 1.33486001 | 0.51896287 | -1.3629852 | 1.58E-16 | 2.21E-15 | down |
| RP11-887P2.5\|ENSG00000258274\|antisense | 4.50786719 | 0.52005213 | -3.1157169 | 3.69E-14 | 3.41E-13 | down |
| RP11-472N13.3\|ENSG00000237797\|lincRNA | 1.10512759 | 0.52839162 | -1.0645335 | 6.86E-08 | 2.35E-07 | down |
| LINC00390\|ENSG00000226519\|lincRNA | 1.53036016 | 0.53071945 | -1.5278499 | 1.38E-12 | 9.91E-12 | down |
| RP11-6O2.3\|ENSG00000261616\|antisense | 3.64141176 | 0.54242792 | -2.7469946 | 2.08E-15 | 2.38E-14 | down |
| AF001548.6\|ENSG00000263065\|antisense | 4.51218659 | 0.55222611 | -3.0304957 | 1.58E-13 | 1.32E-12 | down |
| RP11-227H15.4\|ENSG00000229261\|antisense | 1.71362959 | 0.55725641 | -1.6206421 | 1.40E-13 | 1.18E-12 | down |
| SFTA1P\|ENSG00000225383\|lincRNA | 2.51407588 | 0.56472184 | -2.1544159 | 4.11E-18 | 7.59E-17 | down |
| PGM5-AS1\|ENSG00000224958\|antisense | 11.9548943 | 0.57039437 | -4.3894978 | 2.31E-25 | 5.10E-23 | down |
| RP11-467J12.4\|ENSG00000261550\|antisense | 3.4332566 | 0.5844452 | -2.554438 | 1.27E-18 | 2.66E-17 | down |
| RP11-412D9.4\|ENSG00000260329\|antisense | 1.41247632 | 0.59274037 | -1.2527544 | 2.38E-06 | 6.32E-06 | down |
| RP11-63P12.7\|ENSG00000235523\|lincRNA | 2.10331327 | 0.59306014 | -1.8264134 | 2.13E-10 | 1.05E-09 | down |
| KIAA0125\|ENSG00000226777\|lincRNA | 2.10215255 | 0.59762854 | -1.8145464 | 6.37E-17 | 9.59E-16 | down |
| CTC-436P18.3\|ENSG00000249279\|lincRNA | 2.89418102 | 0.61251334 | -2.240342 | 1.00E-19 | 2.67E-18 | down |
| RP11-37L2.1\|ENSG00000234535\|lincRNA | 1.44770634 | 0.62151237 | -1.219914 | 9.74E-14 | 8.45E-13 | down |
| NOVA1-AS1\|ENSG00000257842\|antisense | 1.40931479 | 0.62546305 | -1.1719973 | 1.80E-12 | 1.27E-11 | down |
| CTC-296K1.3\|ENSG00000267505\|lincRNA | 4.17720464 | 0.62616402 | -2.7379253 | 5.42E-20 | 1.59E-18 | down |
| B3GALT5-AS1\|ENSG00000184809\|antisense | 11.1253448 | 0.65627519 | -4.0834053 | 6.24E-19 | 1.40E-17 | down |
| A2M-AS1\|ENSG00000245105\|antisense | 1.6211265 | 0.65937154 | -1.2978332 | 1.14E-10 | 5.83E-10 | down |
| AC020571.3\|ENSG00000229056\|antisense | 1.33038177 | 0.66187277 | -1.0072145 | 1.43E-09 | 6.29E-09 | down |
| RP11-367G18.1\|ENSG00000230943\|lincRNA | 1.98040566 | 0.66406841 | -1.5763922 | 6.54E-08 | 2.25E-07 | down |
| RP13-497K6.1\|ENSG00000249453\|antisense | 5.69436714 | 0.66871074 | -3.0900813 | 1.78E-23 | 1.52E-21 | down |
| RP11-396F22.1\|ENSG00000257718\|antisense | 1.59582371 | 0.67277069 | -1.2461145 | 7.39E-15 | 7.65E-14 | down |
| GNG12-AS1\|ENSG00000232284\|antisense | 1.46975071 | 0.68506956 | -1.1012491 | 1.75E-15 | 2.02E-14 | down |
| EPB41L4A-AS2\|ENSG00000278921\|lincRNA | 1.73294632 | 0.70587461 | -1.2957431 | 2.65E-16 | 3.57E-15 | down |
| CA3-AS1\|ENSG00000253549\|antisense | 4.99174658 | 0.72187092 | -2.7897319 | 3.45E-23 | 2.54E-21 | down |
| CTD-2306A12.1\|ENSG00000273972\|lincRNA | 1.8995954 | 0.73273462 | -1.3743295 | 5.83E-12 | 3.79E-11 | down |
| RP11-296O14.3\|ENSG00000203739\|antisense | 1.77710165 | 0.73462007 | -1.274456 | 1.04E-18 | 2.25E-17 | down |
| RP5-1185I7.1\|ENSG00000232756\|antisense | 1.92061346 | 0.73608849 | -1.3836161 | 3.20E-13 | 2.55E-12 | down |
| RP11-532F6.3\|ENSG00000272463\|lincRNA | 3.71217972 | 0.74954168 | -2.308186 | 1.54E-21 | 6.99E-20 | down |
| TMEM220-AS1\|ENSG00000263400\|antisense | 4.15328517 | 0.76734573 | -2.4363043 | 4.10E-25 | 7.76E-23 | down |
| RP11-310P5.2\|ENSG00000250385\|antisense | 2.15415032 | 0.77654411 | -1.4719791 | 0.00011045 | 0.00022576 | down |
| AC144831.1\|ENSG00000261888\|lincRNA | 2.57444485 | 0.7818711 | -1.7192587 | 6.87E-20 | 1.93E-18 | down |
| RP11-963H4.3\|ENSG00000263508\|lincRNA | 2.95117582 | 0.78317268 | -1.9138875 | 1.74E-20 | 6.02E-19 | down |
| RP11-498E2.9\|ENSG00000269970\|sense_intronic | 2.82960192 | 0.79275179 | -1.835658 | 6.39E-16 | 7.99E-15 | down |
| CTC-428G20.6\|ENSG00000271797\|antisense | 2.21045362 | 0.83415537 | -1.4059544 | 1.13E-21 | 5.37E-20 | down |
| RP11-116O18.1\|ENSG00000266968\|sense_intronic | 3.45909836 | 0.83832402 | -2.0448162 | 5.74E-06 | 1.43E-05 | down |
| AC073283.4\|ENSG00000234690\|lincRNA | 3.97494452 | 0.84379174 | -2.2359759 | 6.71E-23 | 4.56E-21 | down |
| LINC00865\|ENSG00000232229\|lincRNA | 1.69192247 | 0.84509835 | -1.0014723 | 6.50E-10 | 2.97E-09 | down |
| RP11-341G23.3\|ENSG00000257766\|antisense | 1.98523424 | 0.86612615 | -1.1966602 | 2.31E-13 | 1.88E-12 | down |
| RP11-203J24.9\|ENSG00000257524\|processed_transcript | 3.26006724 | 0.87952439 | -1.8901062 | 3.90E-06 | 9.99E-06 | down |
| C5orf66-AS1\|ENSG00000249082\|lincRNA | 2.44990442 | 0.88681198 | -1.4660253 | 5.26E-15 | 5.61E-14 | down |
| LINC00704\|ENSG00000231298\|lincRNA | 1.79690864 | 0.89398069 | -1.0072015 | 1.38E-11 | 8.31E-11 | down |
| RP11-731D1.4\|ENSG00000270265\|lincRNA | 2.25282213 | 0.89897393 | -1.3253822 | 2.74E-14 | 2.57E-13 | down |
| FGF14-AS2\|ENSG00000272143\|lincRNA | 3.12622467 | 0.90875076 | -1.7824649 | 3.87E-18 | 7.25E-17 | down |
| TINCR\|ENSG00000223573\|lincRNA | 5.44880827 | 0.91045723 | -2.5812776 | 1.22E-23 | 1.12E-21 | down |
| RP11-344B5.2\|ENSG00000224307\|lincRNA | 2.17535859 | 0.91828633 | -1.2442373 | 1.46E-13 | 1.23E-12 | down |
| RP11-400K9.4\|ENSG00000237807\|lincRNA | 4.18632854 | 0.94684176 | -2.1444903 | 2.12E-15 | 2.41E-14 | down |
| RP11-356O9.2\|ENSG00000259087\|lincRNA | 10.6873445 | 0.9542876 | -3.4853355 | 3.16E-24 | 3.89E-22 | down |
| RP11-507K2.3\|ENSG00000258789\|antisense | 2.33608268 | 0.95981903 | -1.283257 | 5.17E-14 | 4.67E-13 | down |
| KB-68A7.1\|ENSG00000274225\|lincRNA | 2.25669441 | 0.97741452 | -1.2071686 | 1.35E-06 | 3.73E-06 | down |
| RP11-1399P15.1\|ENSG00000273445\|antisense | 2.6813351 | 1.01265473 | -1.4048092 | 5.20E-14 | 4.69E-13 | down |
| RP11-325F22.2\|ENSG00000237513\|lincRNA | 3.1378614 | 1.01294522 | -1.6312255 | 1.24E-18 | 2.62E-17 | down |
| RP11-425D10.10\|ENSG00000260273\|antisense | 2.73423816 | 1.01633184 | -1.4277674 | 2.46E-13 | 1.99E-12 | down |
| ERVH-1\|ENSG00000251292\|lincRNA | 2.76913193 | 1.02848849 | -1.4289081 | 2.04E-15 | 2.35E-14 | down |
| RP11-524H19.2\|ENSG00000224984\|antisense | 3.36141369 | 1.03887819 | -1.6940416 | 1.68E-15 | 1.97E-14 | down |
| LINC00996\|ENSG00000242258\|lincRNA | 2.72164272 | 1.04998857 | -1.3741041 | 5.43E-15 | 5.76E-14 | down |
| CDKN2B-AS1\|ENSG00000240498\|antisense | 29.1484507 | 1.05748248 | -4.7847135 | 8.65E-26 | 2.41E-23 | down |
| RP11-554A11.4\|ENSG00000261625\|sense_overlapping | 3.63535982 | 1.05853865 | -1.7800242 | 3.70E-10 | 1.76E-09 | down |
| AC006129.2\|ENSG00000268027\|lincRNA | 2.55708486 | 1.06971518 | -1.2572733 | 1.74E-13 | 1.45E-12 | down |
| RP11-284N8.3\|ENSG00000259834\|lincRNA | 4.03109586 | 1.07631814 | -1.9050675 | 2.85E-19 | 6.91E-18 | down |
| AC083900.1\|ENSG00000225111\|sense_intronic | 3.07905079 | 1.09804881 | -1.4875435 | 1.57E-14 | 1.55E-13 | down |
| RP11-396O20.2\|ENSG00000254645\|lincRNA | 25.5839355 | 1.12033707 | -4.5132334 | 1.83E-23 | 1.54E-21 | down |
| RP11-77K12.10\|ENSG00000273971\|lincRNA | 3.77320392 | 1.12085597 | -1.7511892 | 2.90E-12 | 1.98E-11 | down |
| AC002511.2\|ENSG00000233214\|lincRNA | 3.24090698 | 1.14819887 | -1.4970251 | 7.91E-14 | 6.96E-13 | down |
| CTC-510F12.7\|ENSG00000273733\|antisense | 2.50757417 | 1.1546412 | -1.1188478 | 1.72E-08 | 6.37E-08 | down |
| ZNF667-AS1\|ENSG00000166770\|lincRNA | 3.78020385 | 1.25303439 | -1.593038 | 3.60E-15 | 3.97E-14 | down |
| ID2-AS1\|ENSG00000235092\|antisense | 2.81738844 | 1.28169069 | -1.1363103 | 4.38E-13 | 3.44E-12 | down |
| RP11-542M13.2\|ENSG00000269667\|lincRNA | 11.3245116 | 1.28273447 | -3.1421544 | 1.38E-23 | 1.24E-21 | down |
| RP11-35P15.1\|ENSG00000270403\|lincRNA | 10.90902 | 1.29444851 | -3.075112 | 5.33E-24 | 5.41E-22 | down |
| RP11-95I16.6\|ENSG00000278484\|lincRNA | 3.77690395 | 1.29572643 | -1.5434429 | 6.02E-17 | 9.12E-16 | down |
| RP11-774O3.3\|ENSG00000251615\|lincRNA | 2.76527134 | 1.30644995 | -1.0817692 | 1.14E-14 | 1.15E-13 | down |
| TRPC7-AS1\|ENSG00000248211\|antisense | 4.6922222 | 1.31934513 | -1.8304493 | 2.69E-20 | 8.90E-19 | down |
| RP11-900F13.3\|ENSG00000281333\|antisense | 2.756605 | 1.350524 | -1.0293733 | 6.40E-12 | 4.11E-11 | down |
| RTCA-AS1\|ENSG00000224616\|antisense | 2.80253938 | 1.39974216 | -1.0015735 | 9.47E-12 | 5.88E-11 | down |
| RP11-102C16.3\|ENSG00000227907\|antisense | 3.20906826 | 1.40731647 | -1.1892077 | 8.44E-15 | 8.63E-14 | down |
| RP11-290F5.1\|ENSG00000249096\|lincRNA | 3.93574577 | 1.41949957 | -1.4712546 | 6.82E-13 | 5.19E-12 | down |
| AC104699.1\|ENSG00000224220\|antisense | 5.32869377 | 1.41971169 | -1.9081839 | 2.29E-15 | 2.58E-14 | down |
| RP11-968O1.5\|ENSG00000256139\|sense_overlapping | 3.13715522 | 1.42510682 | -1.1383869 | 1.69E-16 | 2.34E-15 | down |
| RP11-321G12.1\|ENSG00000259459\|lincRNA | 8.4916065 | 1.43246882 | -2.5675338 | 3.31E-21 | 1.34E-19 | down |
| AC144831.3\|ENSG00000274370\|lincRNA | 4.98147932 | 1.43382333 | -1.796707 | 4.15E-21 | 1.67E-19 | down |
| RP11-440I14.3\|ENSG00000251584\|lincRNA | 2.88122253 | 1.4389419 | -1.0016727 | 4.70E-09 | 1.91E-08 | down |
| RP11-96D1.6\|ENSG00000261469\|sense_intronic | 8.95614028 | 1.54533817 | -2.5349545 | 2.21E-23 | 1.78E-21 | down |
| MBNL1-AS1\|ENSG00000229619\|antisense | 8.35100651 | 1.57009115 | -2.4111018 | 2.36E-18 | 4.66E-17 | down |
| TP73-AS1\|ENSG00000227372\|antisense | 3.98839529 | 1.57068899 | -1.3444109 | 3.12E-16 | 4.17E-15 | down |
| RP11-616M22.12\|ENSG00000277010\|antisense | 3.42028873 | 1.57090288 | -1.1225241 | 2.02E-12 | 1.41E-11 | down |
| RP11-677M14.3\|ENSG00000250073\|antisense | 5.41867605 | 1.60356411 | -1.7566584 | 5.22E-22 | 2.71E-20 | down |
| AL928768.3\|ENSG00000253701\|lincRNA | 16.0956688 | 1.62608488 | -3.3071981 | 2.56E-23 | 2.00E-21 | down |
| RP11-1060J15.4\|ENSG00000256377\|antisense | 5.27562377 | 1.74643651 | -1.5949275 | 1.00E-15 | 1.21E-14 | down |
| NALT1\|ENSG00000237886\|antisense | 3.97435126 | 1.88555366 | -1.0757312 | 0.02001827 | 0.02749141 | down |
| RP13-616I3.1\|ENSG00000272989\|lincRNA | 4.05721824 | 1.94773188 | -1.0586958 | 9.25E-10 | 4.15E-09 | down |
| RP11-6N17.3\|ENSG00000266601\|antisense | 3.93543366 | 1.95294895 | -1.0108684 | 6.22E-13 | 4.76E-12 | down |
| KBTBD11-OT1\|ENSG00000253696\|lincRNA | 4.47573913 | 1.97576161 | -1.1797171 | 4.29E-16 | 5.55E-15 | down |
| RP11-502I4.3\|ENSG00000270964\|lincRNA | 4.16874537 | 2.04006375 | -1.030999 | 9.07E-17 | 1.33E-15 | down |
| RP11-514F3.5\|ENSG00000255186\|sense_intronic | 4.91618189 | 2.05852821 | -1.2559251 | 1.70E-13 | 1.42E-12 | down |
| UBXN10-AS1\|ENSG00000225986\|antisense | 12.9377559 | 2.06116164 | -2.6500579 | 5.16E-09 | 2.08E-08 | down |
| XXbac-B476C20.9\|ENSG00000225335\|antisense | 9.47571668 | 2.08121766 | -2.1868072 | 3.12E-23 | 2.40E-21 | down |
| RP11-166D19.1\|ENSG00000255248\|sense_overlapping | 4.27324045 | 2.09664797 | -1.0272458 | 1.62E-05 | 3.77E-05 | down |
| C14orf132\|ENSG00000227051\|lincRNA | 7.12538707 | 2.16866942 | -1.7161582 | 4.02E-18 | 7.46E-17 | down |
| RP11-680F8.1\|ENSG00000256802\|antisense | 4.39070886 | 2.18789142 | -1.0049127 | 8.50E-11 | 4.48E-10 | down |
| DPP10-AS1\|ENSG00000235026\|antisense | 10.0043995 | 2.1956174 | -2.187936 | 1.40E-18 | 2.88E-17 | down |
| RP11-1260E13.2\|ENSG00000262294\|antisense | 4.62121483 | 2.24083128 | -1.0442381 | 1.20E-11 | 7.36E-11 | down |
| RP5-1057J7.7\|ENSG00000271420\|lincRNA | 5.06112051 | 2.34858264 | -1.1076665 | 4.36E-15 | 4.74E-14 | down |
| RP11-834C11.4\|ENSG00000250742\|lincRNA | 5.64158602 | 2.35491045 | -1.2604286 | 2.05E-16 | 2.81E-15 | down |
| RP5-1148A21.3\|ENSG00000266680\|antisense | 6.61381534 | 2.49176315 | -1.4083158 | 9.88E-20 | 2.65E-18 | down |
| AP001610.9\|ENSG00000232806\|lincRNA | 6.10582732 | 2.55001484 | -1.2596811 | 4.70E-10 | 2.20E-09 | down |
| RP11-25K19.1\|ENSG00000167912\|antisense | 6.17517132 | 2.76480043 | -1.1593038 | 1.60E-14 | 1.56E-13 | down |
| SERTAD4-AS1\|ENSG00000203706\|antisense | 8.34334181 | 3.12969343 | -1.414604 | 5.07E-07 | 1.50E-06 | down |
| B4GALT1-AS1\|ENSG00000233554\|antisense | 9.45496105 | 3.35488919 | -1.4948064 | 7.42E-22 | 3.68E-20 | down |
| RP11-528A4.2\|ENSG00000229155\|lincRNA | 11.4937417 | 3.48544855 | -1.7214323 | 3.01E-20 | 9.86E-19 | down |
| FLJ22763\|ENSG00000241224\|lincRNA | 11.4199577 | 3.65861051 | -1.6421896 | 4.40E-15 | 4.77E-14 | down |
| LINC01082\|ENSG00000269186\|lincRNA | 19.5913684 | 3.74079483 | -2.3888014 | 4.35E-24 | 4.90E-22 | down |
| RP11-800A3.4\|ENSG00000260401\|sense_overlapping | 9.83715366 | 4.28056309 | -1.2004404 | 3.29E-14 | 3.05E-13 | down |
| AC053503.6\|ENSG00000234638\|antisense | 9.99576507 | 4.31852794 | -1.2107774 | 2.54E-09 | 1.07E-08 | down |
| SMIM2-AS1\|ENSG00000227258\|antisense | 9.01224883 | 4.49354615 | -1.0040327 | 4.84E-11 | 2.64E-10 | down |
| RP11-490M8.1\|ENSG00000260025\|lincRNA | 9.3122162 | 4.55589153 | -1.0313912 | 1.78E-18 | 3.64E-17 | down |
| FENDRR\|ENSG00000268388\|lincRNA | 16.7730527 | 5.06718036 | -1.7268902 | 2.71E-18 | 5.28E-17 | down |
| PDCD4-AS1\|ENSG00000203497\|antisense | 11.7244068 | 5.11814248 | -1.1958227 | 1.85E-21 | 8.15E-20 | down |
| RP11-160O5.1\|ENSG00000263470\|lincRNA | 14.1615511 | 5.44563454 | -1.3788072 | 6.62E-21 | 2.56E-19 | down |
| RP11-703I16.1\|ENSG00000267480\|antisense | 12.3571803 | 5.84354149 | -1.0804347 | 1.21E-17 | 2.05E-16 | down |
| USP30-AS1\|ENSG00000256262\|antisense | 14.6953109 | 6.51653018 | -1.17318 | 5.69E-10 | 2.62E-09 | down |
| RP1-278O22.1\|ENSG00000224961\|lincRNA | 27.5249363 | 6.68035493 | -2.0427426 | 7.44E-20 | 2.08E-18 | down |
| CTD-3157E16.2\|ENSG00000276855\|lincRNA | 16.7968572 | 6.71567699 | -1.3225866 | 9.26E-18 | 1.60E-16 | down |
| HAGLR\|ENSG00000224189\|antisense | 24.9971717 | 7.0601279 | -1.8239986 | 1.48E-20 | 5.28E-19 | down |
| RP11-452I5.2\|ENSG00000266036\|antisense | 26.7211047 | 7.50682296 | -1.8317053 | 8.81E-16 | 1.07E-14 | down |
| AC009014.3\|ENSG00000271824\|lincRNA | 21.5514773 | 7.56123641 | -1.5110927 | 5.51E-16 | 6.96E-15 | down |
| CTD-3157E16.1\|ENSG00000265519\|lincRNA | 22.8418512 | 7.7090119 | -1.5670617 | 4.76E-20 | 1.42E-18 | down |
| AC092580.4\|ENSG00000235576\|lincRNA | 15.8390252 | 7.78501594 | -1.0247117 | 5.24E-10 | 2.43E-09 | down |
| CTC-490G23.2\|ENSG00000231412\|lincRNA | 39.3388015 | 7.98188523 | -2.3011516 | 2.72E-11 | 1.54E-10 | down |
| RP11-349K16.1\|ENSG00000256643\|lincRNA | 41.014382 | 8.75790903 | -2.2274715 | 4.61E-22 | 2.45E-20 | down |
| RP11-394O4.5\|ENSG00000269936\|lincRNA | 36.4211184 | 9.80470466 | -1.8932291 | 1.54E-07 | 4.96E-07 | down |
| LINC00483\|ENSG00000167117\|processed_transcript | 25.4845285 | 9.88712899 | -1.3659981 | 7.80E-18 | 1.37E-16 | down |
| MIR22HG\|ENSG00000186594\|lincRNA | 32.70033 | 11.5570513 | -1.5005318 | 1.75E-21 | 7.80E-20 | down |
| AP000439.3\|ENSG00000255774\|lincRNA | 38.8884234 | 16.5759424 | -1.2302499 | 5.15E-15 | 5.51E-14 | down |
| RP11-519G16.5\|ENSG00000259342\|antisense | 54.7628505 | 19.9348501 | -1.4579048 | 3.39E-16 | 4.47E-15 | down |
| INAFM2\|ENSG00000259330\|antisense | 66.3175476 | 28.8698214 | -1.1998285 | 8.60E-13 | 6.44E-12 | down |
| LINC01133\|ENSG00000224259\|lincRNA | 84.6738831 | 30.5203353 | -1.4721462 | 3.91E-18 | 7.27E-17 | down |
| LINC00675\|ENSG00000263429\|lincRNA | 119.633386 | 38.2862658 | -1.6437212 | 1.28E-22 | 7.51E-21 | down |
| PP7080\|ENSG00000188242\|antisense | 286.553977 | 38.3307645 | -2.9022322 | 2.23E-14 | 2.11E-13 | down |
| ABHD11-AS1\|ENSG00000225969\|antisense | 88.1001915 | 39.0241703 | -1.1747772 | 4.50E-14 | 4.09E-13 | down |
