# Supplementary material for: A 7-lncRNA signature associated with the prognosis of colon adenocarcinoma
Source: PeerJ. 2020 Apr 10;8:e8877. doi: 10.7717/peerj.8877 (PMC7153553; doi:10.7717/peerj.8877)
Supplement: Table S2 [file peerj-08-8877-s002.docx]

| id | HR | HR.95L | HR.95H | pvalue |
| --- | --- | --- | --- | --- |
| AC005532.5\|ENSG00000230825\|processed_transcript | 1.80165449 | 1.43256256 | 2.26584094 | 4.82E-07 |
| RP11-399K21.14\|ENSG00000272692\|lincRNA | 1.78699051 | 1.39271146 | 2.29289063 | 5.01E-06 |
| AC073283.7\|ENSG00000225187\|antisense | 1.24240521 | 1.1310626 | 1.36470845 | 5.87E-06 |
| AC009404.2\|ENSG00000236255\|lincRNA | 1.79853398 | 1.3682841 | 2.36407372 | 2.58E-05 |
| AC019118.4\|ENSG00000226649\|lincRNA | 5.41080562 | 2.43595445 | 12.0186227 | 3.38E-05 |
| LINC00282\|ENSG00000281106\|lincRNA | 14.9945361 | 3.82222694 | 58.8233292 | 0.00010334 |
| ACTN1-AS1\|ENSG00000259062\|antisense | 7.4772181 | 2.663637 | 20.9896433 | 0.0001333 |
| RP4-564F22.7\|ENSG00000275401\|lincRNA | 2.04400089 | 1.41446551 | 2.95372322 | 0.00014125 |
| RP4-564F22.5\|ENSG00000224635\|lincRNA | 1.66368546 | 1.27411561 | 2.17236906 | 0.00018423 |
| AP006621.6\|ENSG00000255142\|lincRNA | 1.10559823 | 1.04650165 | 1.16803203 | 0.00034142 |
| RP11-273B20.1\|ENSG00000256967\|antisense | 1.1946437 | 1.08366106 | 1.31699258 | 0.00035019 |
| MIRLET7BHG\|ENSG00000197182\|lincRNA | 1.12840215 | 1.05580487 | 1.20599123 | 0.00037019 |
| RP4-694A7.2\|ENSG00000233589\|antisense | 1.18806664 | 1.07857015 | 1.30867923 | 0.00047738 |
| RP11-167H9.4\|ENSG00000243944\|antisense | 1.21552061 | 1.08802404 | 1.35795745 | 0.00055612 |
| SNHG17\|ENSG00000196756\|processed_transcript | 1.01396535 | 1.00598469 | 1.02200932 | 0.00058178 |
| AC007461.2\|ENSG00000226101\|lincRNA | 4.52206131 | 1.8842531 | 10.8525965 | 0.00072925 |
| TSPEAR-AS1\|ENSG00000235890\|antisense | 1.39582451 | 1.14998389 | 1.69422031 | 0.00074155 |
| AC006273.5\|ENSG00000267530\|lincRNA | 1.19841727 | 1.07764828 | 1.3327205 | 0.00083835 |
| LRP4-AS1\|ENSG00000247675\|antisense | 1.12929805 | 1.05082946 | 1.21362614 | 0.00093528 |
| FALEC\|ENSG00000228126\|lincRNA | 1.76708229 | 1.25689195 | 2.48436616 | 0.00105538 |
| RP11-400N13.2\|ENSG00000228437\|lincRNA | 1.05413239 | 1.02138018 | 1.08793486 | 0.00106185 |
| RP4-816N1.7\|ENSG00000258092\|antisense | 1.48381766 | 1.16871843 | 1.88387108 | 0.00119502 |
| CTC-435M10.12\|ENSG00000277744\|sense_intronic | 1.25339244 | 1.09115904 | 1.43974668 | 0.00140547 |
| RP6-74O6.6\|ENSG00000272824\|lincRNA | 1.20764965 | 1.07510112 | 1.35654 | 0.00146894 |
| RP11-476H16.1\|ENSG00000277020\|antisense | 1.58706431 | 1.19300817 | 2.11127901 | 0.00151456 |
| AC018766.4\|ENSG00000267896\|antisense | 1.0890142 | 1.0322026 | 1.14895267 | 0.0018121 |
| RP11-278A23.1\|ENSG00000226180\|lincRNA | 1.91177011 | 1.2714328 | 2.8746033 | 0.0018463 |
| RP11-148K1.12\|ENSG00000244151\|antisense | 1.24348809 | 1.08240541 | 1.42854296 | 0.00207937 |
| AC012363.4\|ENSG00000224789\|antisense | 1.35497325 | 1.11613625 | 1.64491789 | 0.00213685 |
| MMP25-AS1\|ENSG00000261971\|antisense | 1.13202754 | 1.04477459 | 1.2265673 | 0.00244332 |
| LINC00862\|ENSG00000203721\|lincRNA | 26.6987856 | 3.18938729 | 223.499089 | 0.00244698 |
| AC011997.1\|ENSG00000222017\|antisense | 2.8156941 | 1.43563739 | 5.52237867 | 0.0025942 |
| CTD-2020K17.3\|ENSG00000233175\|antisense | 1.70498838 | 1.2046561 | 2.4131247 | 0.0026078 |
| RP11-680A11.5\|ENSG00000257605\|antisense | 1.32715061 | 1.10360747 | 1.59597392 | 0.00263388 |
| AC024560.2\|ENSG00000236833\|lincRNA | 1.54563788 | 1.16372591 | 2.05288587 | 0.00263766 |
| CTC-273B12.10\|ENSG00000269814\|lincRNA | 1.42018388 | 1.12853388 | 1.78720578 | 0.0027808 |
| AC007879.7\|ENSG00000229647\|lincRNA | 2.73416559 | 1.413263 | 5.28964633 | 0.00281469 |
| RP11-310P5.1\|ENSG00000249650\|antisense | 1.22581505 | 1.07196377 | 1.40174751 | 0.00292476 |
| RP5-956O18.2\|ENSG00000227006\|antisense | 1.52757436 | 1.15353919 | 2.02289045 | 0.00310874 |
| RP11-336K24.12\|ENSG00000273002\|antisense | 1.67676493 | 1.18835918 | 2.36590138 | 0.00325703 |
| RP11-152N13.16\|ENSG00000272599\|antisense | 1.09173946 | 1.0296649 | 1.15755626 | 0.0032955 |
| AC027601.1\|ENSG00000260005\|antisense | 1.33621221 | 1.10067016 | 1.62215996 | 0.00339586 |
| RP11-829H16.3\|ENSG00000258525\|antisense | 6.93402937 | 1.88795934 | 25.4670544 | 0.00352981 |
| PRR7-AS1\|ENSG00000246334\|antisense | 1.38395504 | 1.11211707 | 1.72223914 | 0.00358674 |
| CTD-2293H3.2\|ENSG00000267345\|antisense | 4.48413741 | 1.63261146 | 12.3161504 | 0.00360453 |
| RP11-568A7.3\|ENSG00000231720\|lincRNA | 1.17649252 | 1.05292062 | 1.31456695 | 0.00409482 |
| RP11-186F10.2\|ENSG00000257761\|antisense | 1.15927128 | 1.04783762 | 1.28255551 | 0.00415438 |
| RP11-785D18.3\|ENSG00000277247\|antisense | 1.02055087 | 1.00643379 | 1.03486597 | 0.00420518 |
| AC002306.1\|ENSG00000259242\|antisense | 2.28329 | 1.29214037 | 4.03471119 | 0.00447869 |
| HOTAIR\|ENSG00000228630\|antisense | 1.08393632 | 1.02527934 | 1.14594912 | 0.0045188 |
| CTD-2529P6.3\|ENSG00000269300\|antisense | 1.95712544 | 1.22939614 | 3.11562716 | 0.00464694 |
| RP11-700H6.4\|ENSG00000262006\|lincRNA | 1.9551604 | 1.22785473 | 3.11327725 | 0.0047312 |
| LINC01433\|ENSG00000230176\|antisense | 2.10412953 | 1.25566184 | 3.52591832 | 0.0047382 |
| CTD-3128G10.6\|ENSG00000269680\|antisense | 1.1901615 | 1.0547136 | 1.34300382 | 0.00474122 |
| RP11-462L8.1\|ENSG00000229656\|lincRNA | 1.80269695 | 1.19202967 | 2.72620419 | 0.00523324 |
| RP11-351M8.1\|ENSG00000259649\|antisense | 2.28856393 | 1.27714041 | 4.10097809 | 0.00540368 |
| RP11-727F15.13\|ENSG00000269463\|sense_intronic | 1.5299088 | 1.13370521 | 2.06457632 | 0.00542587 |
| RP4-594A5.1\|ENSG00000272328\|lincRNA | 1.35969351 | 1.09449163 | 1.68915538 | 0.00551022 |
| RP11-383I23.2\|ENSG00000273374\|lincRNA | 2.23131926 | 1.26373322 | 3.93974421 | 0.00565898 |
| CTD-2547G23.4\|ENSG00000274925\|lincRNA | 1.23352169 | 1.06270725 | 1.43179201 | 0.00578535 |
| CCDC144NL-AS1\|ENSG00000233098\|antisense | 1.43090065 | 1.10823337 | 1.84751399 | 0.00599259 |
| RP11-429J17.8\|ENSG00000214733\|antisense | 1.37964411 | 1.0958239 | 1.73697423 | 0.00616894 |
| TMEM147-AS1\|ENSG00000236144\|antisense | 1.10034511 | 1.02755456 | 1.17829204 | 0.00617458 |
| FLJ21408\|ENSG00000245888\|antisense | 4.14922867 | 1.49414929 | 11.5223416 | 0.00632311 |
| LINC00843\|ENSG00000178440\|lincRNA | 1.87341404 | 1.19319925 | 2.94140328 | 0.00638376 |
| LINC00244\|ENSG00000279418\|lincRNA | 1.81456525 | 1.18103072 | 2.78794362 | 0.00654155 |
| RP11-153I24.4\|ENSG00000277246\|sense_intronic | 2.14537392 | 1.23575328 | 3.72455355 | 0.00668645 |
| RP4-761J14.8\|ENSG00000219410\|antisense | 7.49469705 | 1.73675449 | 32.3422132 | 0.00693578 |
| RP11-114H23.2\|ENSG00000258088\|lincRNA | 1.34765969 | 1.08464497 | 1.67445265 | 0.00707165 |
| RP11-568A7.2\|ENSG00000228648\|lincRNA | 1.12198196 | 1.03146718 | 1.22043972 | 0.00732085 |
| CTC-573N18.1\|ENSG00000250509\|lincRNA | 1.22396105 | 1.05551237 | 1.41929236 | 0.00747036 |
| AC010761.9\|ENSG00000265474\|antisense | 1.47107312 | 1.10706483 | 1.9547691 | 0.00778587 |
| RP11-434E6.4\|ENSG00000277559\|lincRNA | 1.24365462 | 1.05791488 | 1.46200496 | 0.00823829 |
| RP1-142L7.9\|ENSG00000270661\|lincRNA | 10.1131673 | 1.81604065 | 56.3182069 | 0.00826662 |
| RP11-401P9.5\|ENSG00000260249\|antisense | 5.14149017 | 1.51907025 | 17.4020399 | 0.00848671 |
| RP11-296O14.3\|ENSG00000203739\|antisense | 1.79899144 | 1.15997346 | 2.79003813 | 0.00872214 |
| CTD-2562G15.3\|ENSG00000276744\|sense_intronic | 1.92564703 | 1.17909827 | 3.14487485 | 0.00883785 |
| BBOX1-AS1\|ENSG00000254560\|antisense | 1.04181386 | 1.01033405 | 1.07427452 | 0.00887821 |
| AP000593.7\|ENSG00000255843\|antisense | 2.0284852 | 1.19376647 | 3.44686528 | 0.00893006 |
| RP13-463N16.6\|ENSG00000242147\|lincRNA | 1.38844416 | 1.08534273 | 1.77619209 | 0.00900953 |
| OSGEPL1-AS1\|ENSG00000253559\|antisense | 1.40999686 | 1.08724135 | 1.82856471 | 0.00957997 |
| CTB-58E17.9\|ENSG00000275665\|antisense | 1.92436884 | 1.17227978 | 3.15896895 | 0.00963923 |
| AC005785.2\|ENSG00000268189\|processed_transcript | 1.60048255 | 1.11963983 | 2.28782895 | 0.00988387 |
| ALMS1-IT1\|ENSG00000230002\|sense_intronic | 1.2360294 | 1.05146912 | 1.45298483 | 0.01022173 |
| INE1\|ENSG00000224975\|sense_intronic | 1.14281713 | 1.03186936 | 1.26569412 | 0.01040548 |
| TSPEAR-AS2\|ENSG00000182912\|antisense | 1.12572437 | 1.02760838 | 1.23320848 | 0.01091852 |
| CTC-425O23.2\|ENSG00000205041\|sense_intronic | 1.78632057 | 1.14215768 | 2.79378343 | 0.01100738 |
| CTD-2196E14.6\|ENSG00000260751\|sense_intronic | 1.39390191 | 1.07864788 | 1.80129455 | 0.01112672 |
| RP11-626H12.2\|ENSG00000254605\|lincRNA | 1.09108553 | 1.0199331 | 1.16720168 | 0.0112897 |
| RP1-170O19.17\|ENSG00000253308\|lincRNA | 1.03413191 | 1.0075434 | 1.06142208 | 0.01155509 |
| BX470102.3\|ENSG00000238279\|antisense | 1.02164524 | 1.00477994 | 1.03879361 | 0.01168755 |
| ALG13-AS1\|ENSG00000229487\|antisense | 1.15812563 | 1.03312095 | 1.29825552 | 0.01176545 |
| AC007128.1\|ENSG00000229970\|antisense | 1.20637524 | 1.04234429 | 1.3962193 | 0.01186475 |
| RP11-727F15.9\|ENSG00000256690\|antisense | 1.25673708 | 1.05176827 | 1.50165026 | 0.01188372 |
| LA16c-352F7.1\|ENSG00000278716\|lincRNA | 1.15647267 | 1.0325799 | 1.29523055 | 0.01191998 |
| RP11-429J17.5\|ENSG00000254548\|antisense | 1.06648055 | 1.01419455 | 1.12146211 | 0.01208994 |
| LL22NC03-N14H11.1\|ENSG00000272872\|sense_intronic | 1.17306293 | 1.03546313 | 1.32894798 | 0.01216255 |
| AC137932.4\|ENSG00000268218\|antisense | 1.51597836 | 1.09507481 | 2.09866063 | 0.01216632 |
| RP5-1011O1.2\|ENSG00000232498\|antisense | 1.07231376 | 1.0153493 | 1.13247413 | 0.01217923 |
| RP11-42O4.2\|ENSG00000278367\|antisense | 1.46868736 | 1.08716658 | 1.98409572 | 0.01226126 |
| RP11-332H14.2\|ENSG00000272994\|lincRNA | 1.39106108 | 1.07434746 | 1.8011407 | 0.0122795 |
| AC005387.3\|ENSG00000268938\|antisense | 1.35642806 | 1.06838516 | 1.72212902 | 0.01231134 |
| GAS6-AS1\|ENSG00000233695\|antisense | 1.03577364 | 1.0076041 | 1.06473071 | 0.01247434 |
| NALT1\|ENSG00000237886\|antisense | 1.17782545 | 1.03586274 | 1.33924385 | 0.01250174 |
| RP5-1056H1.2\|ENSG00000278192\|lincRNA | 1.05912897 | 1.01227047 | 1.10815657 | 0.01283908 |
| PAN3-AS1\|ENSG00000261485\|antisense | 1.12069689 | 1.02448444 | 1.22594494 | 0.01284098 |
| RP11-430C7.4\|ENSG00000240710\|antisense | 2.09533588 | 1.16865974 | 3.75680986 | 0.01302201 |
| DCUN1D2-AS\|ENSG00000233613\|antisense | 1.3043597 | 1.05688587 | 1.60978046 | 0.01330904 |
| RP11-211G23.2\|ENSG00000260877\|lincRNA | 1.03515913 | 1.00714913 | 1.06394812 | 0.01355108 |
| RP11-783K16.13\|ENSG00000257086\|lincRNA | 1.15270755 | 1.02943295 | 1.29074429 | 0.01379201 |
| RP11-435O5.4\|ENSG00000271659\|antisense | 1.13586391 | 1.02605299 | 1.25742708 | 0.01405891 |
| CTD-3222D19.8\|ENSG00000279529\|antisense | 1.64966531 | 1.10555002 | 2.46157621 | 0.01423201 |
| CTC-550B14.6\|ENSG00000267149\|processed_transcript | 2.54445713 | 1.20004535 | 5.3950145 | 0.01486986 |
| AC006042.6\|ENSG00000227719\|antisense | 1.03947251 | 1.00755634 | 1.07239967 | 0.01497079 |
| PCAT6\|ENSG00000228288\|antisense | 1.06414143 | 1.0121604 | 1.11879204 | 0.01497434 |
| RP5-1063M23.2\|ENSG00000236908\|lincRNA | 2.69692873 | 1.20938567 | 6.01414815 | 0.01532657 |
| AC118754.4\|ENSG00000229782\|antisense | 1.29001166 | 1.04926889 | 1.5859901 | 0.0156787 |
| RP11-867G23.12\|ENSG00000254756\|antisense | 1.59362173 | 1.09093983 | 2.32792876 | 0.0159474 |
| IBA57-AS1\|ENSG00000203684\|lincRNA | 1.24122802 | 1.04115254 | 1.47975148 | 0.01596798 |
| LINC01555\|ENSG00000180869\|lincRNA | 0.58180216 | 0.37320758 | 0.90698519 | 0.01680549 |
| RDH10-AS1\|ENSG00000250295\|antisense | 3.67029547 | 1.26185174 | 10.6756352 | 0.01699056 |
| RP11-10A14.5\|ENSG00000248538\|lincRNA | 1.12663656 | 1.02118367 | 1.24297908 | 0.0174052 |
| RHOA-IT1\|ENSG00000235908\|sense_intronic | 1.31339874 | 1.04794707 | 1.646091 | 0.01795688 |
| RP11-383J24.1\|ENSG00000253227\|lincRNA | 1.16177516 | 1.02555727 | 1.31608595 | 0.01844459 |
| UNC5B-AS1\|ENSG00000237512\|antisense | 1.02349633 | 1.00389545 | 1.04347991 | 0.01857061 |
| LINC01356\|ENSG00000215866\|lincRNA | 1.3187416 | 1.04735602 | 1.66044724 | 0.01859519 |
| CTB-50L17.9\|ENSG00000267769\|antisense | 1.22255557 | 1.03370781 | 1.44590387 | 0.01891443 |
| C1RL-AS1\|ENSG00000205885\|antisense | 1.22855127 | 1.03405415 | 1.45963172 | 0.01924335 |
| RP11-167N5.5\|ENSG00000267834\|lincRNA | 1.47778623 | 1.06412973 | 2.0522424 | 0.01975619 |
| RP11-148O21.2\|ENSG00000255354\|antisense | 1.17635481 | 1.02571921 | 1.34911253 | 0.02016902 |
| RP11-363N22.3\|ENSG00000205740\|processed_transcript | 3.44843117 | 1.21255341 | 9.80713712 | 0.02026676 |
| RP11-316M21.6\|ENSG00000227492\|antisense | 2.2746554 | 1.13574722 | 4.5556415 | 0.02038557 |
| FENDRR\|ENSG00000268388\|lincRNA | 0.87520759 | 0.78193302 | 0.97960862 | 0.02043405 |
| AC009133.17\|ENSG00000260719\|antisense | 2.60607269 | 1.15844412 | 5.86270394 | 0.02058487 |
| ELFN1-AS1\|ENSG00000236081\|lincRNA | 1.01001989 | 1.00153152 | 1.0185802 | 0.02059322 |
| RP4-584D14.6\|ENSG00000239377\|antisense | 1.15004394 | 1.02152674 | 1.29472976 | 0.02076513 |
| RP11-367H1.1\|ENSG00000273113\|lincRNA | 1.41202389 | 1.0508614 | 1.89731156 | 0.02207298 |
| LINC00152\|ENSG00000222041\|lincRNA | 1.07151017 | 1.00994967 | 1.13682304 | 0.02214223 |
| RP4-673M15.1\|ENSG00000272768\|antisense | 1.28989639 | 1.03707048 | 1.60435837 | 0.02219699 |
| LINC01410\|ENSG00000238113\|lincRNA | 1.34584261 | 1.04338396 | 1.73597871 | 0.02219793 |
| CTB-25B13.9\|ENSG00000267092\|antisense | 1.33843336 | 1.04218981 | 1.71888445 | 0.0223885 |
| AC114730.11\|ENSG00000235351\|antisense | 1.268145 | 1.03399699 | 1.55531569 | 0.0225501 |
| MIR181A2HG\|ENSG00000224020\|antisense | 1.23126306 | 1.0292553 | 1.47291806 | 0.02288566 |
| RP11-122K13.7\|ENSG00000226699\|antisense | 1.09869252 | 1.01311257 | 1.19150161 | 0.02291647 |
| RP1-63M2.7\|ENSG00000275223\|lincRNA | 1.30521584 | 1.03744563 | 1.64209895 | 0.02297999 |
| MCF2L-AS1\|ENSG00000235280\|antisense | 1.01583373 | 1.0021361 | 1.0297186 | 0.02332764 |
| RP11-143J12.3\|ENSG00000263847\|antisense | 1.23105074 | 1.02853344 | 1.4734435 | 0.02340496 |
| RP11-7I15.4\|ENSG00000254675\|antisense | 1.20665561 | 1.02563872 | 1.41962052 | 0.02349836 |
| NKILA\|ENSG00000278709\|antisense | 1.06501598 | 1.00844327 | 1.12476237 | 0.02370497 |
| RP11-563N4.1\|ENSG00000272716\|lincRNA | 1.75982538 | 1.07609621 | 2.87798185 | 0.02430964 |
| EVX1-AS\|ENSG00000253405\|antisense | 1.04282631 | 1.00536636 | 1.08168202 | 0.02465888 |
| RP11-449D8.1\|ENSG00000265485\|lincRNA | 0.35537697 | 0.14400688 | 0.87699137 | 0.02478356 |
| U47924.29\|ENSG00000271969\|antisense | 1.25602052 | 1.02855576 | 1.53378903 | 0.02534061 |
| AC004076.5\|ENSG00000276449\|antisense | 1.8969154 | 1.08214291 | 3.32515047 | 0.02537619 |
| ASMTL-AS1\|ENSG00000236017\|antisense | 1.0527396 | 1.00635492 | 1.10126223 | 0.02538448 |
| RP11-268J15.5\|ENSG00000116883\|antisense | 1.03375835 | 1.00402848 | 1.06436853 | 0.02574749 |
| AP000640.2\|ENSG00000255355\|processed_transcript | 3.86765215 | 1.17686314 | 12.710682 | 0.02586498 |
| RP11-368I7.6\|ENSG00000275734\|antisense | 1.47979119 | 1.04661693 | 2.09224778 | 0.0265678 |
| RP11-290L1.3\|ENSG00000257453\|antisense | 1.25189886 | 1.02628953 | 1.52710392 | 0.02669718 |
| LENG8-AS1\|ENSG00000226696\|antisense | 1.05895619 | 1.00662013 | 1.11401329 | 0.02675212 |
| CTD-2349P21.10\|ENSG00000265791\|sense_intronic | 1.00670533 | 1.00076953 | 1.01267634 | 0.02676629 |
| RP11-495P10.1\|ENSG00000231551\|lincRNA | 1.56712958 | 1.05295694 | 2.33237942 | 0.02680751 |
| RP11-235E17.6\|ENSG00000262903\|antisense | 1.13543695 | 1.01375304 | 1.27172695 | 0.02808237 |
| RP1-102K2.8\|ENSG00000268812\|antisense | 1.1373139 | 1.01364077 | 1.27607623 | 0.0284784 |
| RP11-245P10.6\|ENSG00000270104\|lincRNA | 2.62518837 | 1.10455693 | 6.23925645 | 0.02888108 |
| RP11-35O15.1\|ENSG00000259424\|antisense | 1.27693702 | 1.02535658 | 1.59024497 | 0.02898921 |
| CTD-2147F2.2\|ENSG00000259664\|lincRNA | 2.20903972 | 1.08456004 | 4.49938805 | 0.02899083 |
| MCM3AP-AS1\|ENSG00000215424\|antisense | 1.49975917 | 1.04145838 | 2.15973832 | 0.02938486 |
| RP13-977J11.8\|ENSG00000256312\|lincRNA | 1.31410252 | 1.02702106 | 1.68143137 | 0.02985801 |
| LINC00174\|ENSG00000179406\|lincRNA | 1.1245014 | 1.01109277 | 1.25063044 | 0.03051415 |
| RP11-29H23.4\|ENSG00000232519\|antisense | 1.5122861 | 1.03928402 | 2.20056233 | 0.03067172 |
| CTD-2371O3.3\|ENSG00000268403\|antisense | 1.1394356 | 1.01214122 | 1.28273946 | 0.03080156 |
| RP11-815M8.1\|ENSG00000238042\|lincRNA | 1.33678393 | 1.02566896 | 1.74226905 | 0.03175559 |
| RP11-393I2.4\|ENSG00000272008\|antisense | 6.38714113 | 1.17464461 | 34.7301402 | 0.03185093 |
| AP001469.9\|ENSG00000239415\|antisense | 1.20622293 | 1.01569216 | 1.43249481 | 0.03255989 |
| CTD-2600O9.2\|ENSG00000276166\|antisense | 1.84729535 | 1.04993653 | 3.25019658 | 0.03325362 |
| MIR503HG\|ENSG00000223749\|lincRNA | 1.07006 | 1.00529591 | 1.13899639 | 0.03352152 |
| RP11-79O8.1\|ENSG00000271991\|lincRNA | 3.29767482 | 1.09471098 | 9.93381765 | 0.03393867 |
| MIR4435-2HG\|ENSG00000172965\|lincRNA | 1.12044791 | 1.00863448 | 1.24465657 | 0.03398531 |
| XXbac-B476C20.9\|ENSG00000225335\|antisense | 0.70653841 | 0.51204591 | 0.97490578 | 0.03445762 |
| RP11-20I20.4\|ENSG00000273179\|antisense | 1.06017024 | 1.00426245 | 1.11919045 | 0.03452878 |
| LINC01234\|ENSG00000249550\|lincRNA | 1.05179743 | 1.00367279 | 1.10222958 | 0.03456789 |
| RP11-502I4.3\|ENSG00000270964\|lincRNA | 1.2664164 | 1.01713068 | 1.57679887 | 0.03470053 |
| LA16c-325D7.2\|ENSG00000263280\|lincRNA | 1.29555113 | 1.01754213 | 1.64951671 | 0.03563474 |
| RP1-41C23.4\|ENSG00000278546\|lincRNA | 1.44952317 | 1.02386111 | 2.05215083 | 0.03635758 |
| AC011513.4\|ENSG00000268833\|antisense | 1.08534124 | 1.00513219 | 1.17195093 | 0.03655977 |
| RP11-108K3.1\|ENSG00000259240\|lincRNA | 1.14606626 | 1.00837803 | 1.30255503 | 0.03682279 |
| RP11-480A16.1\|ENSG00000260261\|lincRNA | 1.21886359 | 1.01217305 | 1.46776131 | 0.03683517 |
| RP11-177H13.2\|ENSG00000253837\|processed_transcript | 10.3186184 | 1.15105948 | 92.5007671 | 0.03700744 |
| CMB9-94B1.2\|ENSG00000276505\|sense_intronic | 1.29915851 | 1.01567501 | 1.66176465 | 0.03717826 |
| RP11-260M2.1\|ENSG00000272807\|antisense | 1.18655804 | 1.00969822 | 1.3943968 | 0.03778649 |
| GABPB1-AS1\|ENSG00000244879\|antisense | 1.07051988 | 1.00384335 | 1.14162514 | 0.03781307 |
| RP11-438L19.1\|ENSG00000272800\|lincRNA | 1.54647842 | 1.02466802 | 2.33401986 | 0.03789396 |
| LINC01235\|ENSG00000270547\|lincRNA | 1.07368339 | 1.00395065 | 1.14825965 | 0.03798249 |
| LINC00865\|ENSG00000232229\|lincRNA | 1.33143536 | 1.01586099 | 1.74504203 | 0.03808142 |
| RP11-351D16.3\|ENSG00000273008\|lincRNA | 1.63230376 | 1.02600763 | 2.59687696 | 0.03860774 |
| TMEM191A\|ENSG00000226287\|processed_transcript | 1.04066763 | 1.00203572 | 1.08078893 | 0.03889174 |
| CTC-268N12.2\|ENSG00000278638\|antisense | 2.16508346 | 1.04006167 | 4.50702736 | 0.03892594 |
| RP11-626G11.4\|ENSG00000260430\|lincRNA | 1.34837542 | 1.01525078 | 1.79080511 | 0.03896977 |
| RP11-798K3.2\|ENSG00000259347\|lincRNA | 1.40172174 | 1.01655001 | 1.9328354 | 0.03938912 |
| CTD-2349P21.6\|ENSG00000265443\|lincRNA | 1.02486025 | 1.00113546 | 1.04914727 | 0.03988599 |
| CELSR3-AS1\|ENSG00000228350\|antisense | 1.12045884 | 1.00517412 | 1.24896572 | 0.04006068 |
| RP4-536B24.4\|ENSG00000260498\|lincRNA | 1.62813898 | 1.02176023 | 2.59438218 | 0.04031354 |
| RP11-843B15.4\|ENSG00000277342\|sense_intronic | 1.34369774 | 1.01278484 | 1.78273167 | 0.04055677 |
| LINC00996\|ENSG00000242258\|lincRNA | 0.6269712 | 0.40088665 | 0.98055868 | 0.04075585 |
| CTD-2256P15.1\|ENSG00000248968\|antisense | 2.55552362 | 1.04006753 | 6.27911245 | 0.0407942 |
| RP11-563J2.3\|ENSG00000212743\|lincRNA | 2.70400299 | 1.03954381 | 7.03350075 | 0.04140237 |
| RP11-50D9.3\|ENSG00000261668\|antisense | 0.00121941 | 1.91E-06 | 0.77864828 | 0.04176212 |
| AF064858.10\|ENSG00000237609\|lincRNA | 1.17041579 | 1.00582398 | 1.36194121 | 0.04184436 |
| CTD-2537I9.13\|ENSG00000267096\|sense_intronic | 1.19920449 | 1.00672472 | 1.42848525 | 0.04184623 |
| CTD-2035E11.5\|ENSG00000272144\|lincRNA | 1.38836692 | 1.01103569 | 1.9065229 | 0.04258173 |
| RP11-137H2.4\|ENSG00000226659\|antisense | 1.28762391 | 1.00840743 | 1.64415225 | 0.04265237 |
| RP11-805I24.3\|ENSG00000268505\|lincRNA | 0.17114699 | 0.03104924 | 0.94338187 | 0.04267393 |
| RP3-431P23.5\|ENSG00000269155\|lincRNA | 1.17678886 | 1.00538555 | 1.37741389 | 0.04267901 |
| AC009410.1\|ENSG00000232023\|lincRNA | 1.08937289 | 1.00278008 | 1.18344323 | 0.0427997 |
| LINC00894\|ENSG00000235703\|antisense | 1.33718448 | 1.00929268 | 1.77159943 | 0.04292817 |
| RP5-1057I20.5\|ENSG00000276691\|antisense | 1.29185462 | 1.00817785 | 1.65535115 | 0.04293432 |
| RP11-114H23.1\|ENSG00000258077\|lincRNA | 1.24056129 | 1.00629232 | 1.52936904 | 0.04351785 |
| RP11-972P1.11\|ENSG00000278112\|sense_intronic | 1.40014284 | 1.00977591 | 1.94142082 | 0.04355987 |
| RP11-365P13.5\|ENSG00000277767\|lincRNA | 1.6669299 | 1.01331152 | 2.74215306 | 0.04421644 |
| AP000697.6\|ENSG00000224269\|antisense | 1.21984686 | 1.00514933 | 1.48040328 | 0.04422375 |
| CTD-2349P21.9\|ENSG00000266490\|lincRNA | 1.00467269 | 1.00010464 | 1.00926161 | 0.04496684 |
| RP11-499O7.7\|ENSG00000224251\|antisense | 1.61617214 | 1.00982123 | 2.58660868 | 0.04542525 |
| CTD-2228K2.7\|ENSG00000225138\|processed_transcript | 1.00970789 | 1.00018713 | 1.01931927 | 0.0456437 |
| CAPN10-AS1\|ENSG00000260942\|antisense | 1.26909981 | 1.00396639 | 1.60425123 | 0.04625367 |
| YEATS2-AS1\|ENSG00000233885\|antisense | 1.75254415 | 1.00598546 | 3.05313658 | 0.0475874 |
| RP11-568J23.5\|ENSG00000270184\|antisense | 1.23315246 | 1.00186187 | 1.51783896 | 0.04798332 |
| AC074363.1\|ENSG00000228222\|lincRNA | 0.38618868 | 0.15020022 | 0.9929526 | 0.04830876 |
| RP11-63A1.1\|ENSG00000261886\|lincRNA | 1.8776291 | 1.00367511 | 3.51258192 | 0.04867321 |
| LINC01138\|ENSG00000274020\|lincRNA | 1.20079393 | 1.00088823 | 1.44062645 | 0.04889339 |
| PLA2G4C-AS1\|ENSG00000269420\|antisense | 2.10599653 | 1.00284575 | 4.42263563 | 0.04912896 |
| RP4-533D7.5\|ENSG00000227857\|antisense | 1.58580643 | 1.00117196 | 2.51183826 | 0.0494194 |
| LRRC3-AS1\|ENSG00000229356\|antisense | 2.07285301 | 1.00147785 | 4.2903791 | 0.04953673 |
