# Supplementary material for: A 7-lncRNA signature associated with the prognosis of colon adenocarcinoma
Source: PeerJ. 2020 Apr 10;8:e8877. doi: 10.7717/peerj.8877 (PMC7153553; doi:10.7717/peerj.8877)
Supplement: Table S3 — Abbreviations: lncRNA, long noncoding RNA; mRNA, messenger RNA. [file peerj-08-8877-s003.docx]

| **lncRNA** | **mRNA** | **cor** | **pvalue** |
| --- | --- | --- | --- |
| AC007879.7 | TPRN | -0.449 | 6.57E-27 |
| AC007879.7 | HDHD3 | -0.444 | 2.86E-26 |
| AC007879.7 | PPP1R14D | -0.437 | 2.21E-25 |
| AC007879.7 | TST | -0.436 | 2.83E-25 |
| AC007879.7 | CDX1 | -0.434 | 4.88E-25 |
| AC007879.7 | BDH1 | -0.432 | 9.69E-25 |
| AC007879.7 | TMEM54 | -0.43 | 1.46E-24 |
| AC007879.7 | MPST | -0.426 | 4.37E-24 |
| AC007879.7 | TJP3 | -0.417 | 4.34E-23 |
| AC007879.7 | LGALS4 | -0.411 | 2.03E-22 |
| AC007879.7 | ACADS | -0.408 | 5.10E-22 |
| AC007879.7 | HSD11B2 | -0.404 | 1.28E-21 |
| AC007879.7 | TTC38 | -0.403 | 1.69E-21 |
| AC007879.7 | SELENBP1 | -0.402 | 2.13E-21 |
| AC007879.7 | GPA33 | -0.402 | 2.23E-21 |
| AC007879.7 | SMIM22 | -0.401 | 2.49E-21 |
| AC007879.7 | HADH | -0.395 | 1.29E-20 |
| AC007879.7 | FAM195A | -0.394 | 1.58E-20 |
| AC007879.7 | FAAH | -0.392 | 2.69E-20 |
| AC007879.7 | ESRRA | -0.391 | 2.99E-20 |
| AC007879.7 | UQCRC1 | -0.391 | 3.28E-20 |
| AC007879.7 | IHH | -0.391 | 3.43E-20 |
| AC007879.7 | DDC | -0.389 | 5.08E-20 |
| AC007879.7 | STAP2 | -0.388 | 5.88E-20 |
| AC007879.7 | PHGR1 | -0.387 | 7.48E-20 |
| AC007879.7 | CHCHD10 | -0.386 | 1.04E-19 |
| AC007879.7 | COX5A | -0.385 | 1.34E-19 |
| AC007879.7 | MGAT4B | -0.384 | 1.82E-19 |
| AC007879.7 | TMPRSS2 | -0.382 | 2.63E-19 |
| AC007879.7 | CDHR5 | -0.381 | 3.33E-19 |
| AC007879.7 | PLEKHJ1 | -0.379 | 5.56E-19 |
| AC007879.7 | COX5B | -0.378 | 7.43E-19 |
| AC007879.7 | CBLC | -0.376 | 1.15E-18 |
| AC007879.7 | SHD | -0.375 | 1.29E-18 |
| AC007879.7 | MMP15 | -0.375 | 1.43E-18 |
| AC007879.7 | ST14 | -0.373 | 2.21E-18 |
| AC007879.7 | CLDN3 | -0.372 | 2.72E-18 |
| AC007879.7 | EPS8L3 | -0.371 | 3.45E-18 |
| AC007879.7 | B3GNT8 | -0.369 | 4.65E-18 |
| AC007879.7 | ANKRD9 | -0.368 | 6.17E-18 |
| AC007879.7 | PRSS3 | -0.368 | 6.96E-18 |
| AC007879.7 | COQ9 | -0.367 | 7.75E-18 |
| AC007879.7 | AP1M2 | -0.367 | 7.93E-18 |
| AC007879.7 | ALKBH7 | -0.366 | 9.94E-18 |
| AC007879.7 | SHROOM1 | -0.365 | 1.27E-17 |
| AC007879.7 | CISD3 | -0.365 | 1.34E-17 |
| AC007879.7 | LLGL2 | -0.363 | 1.97E-17 |
| AC007879.7 | ELMO3 | -0.362 | 2.12E-17 |
| AC007879.7 | KDF1 | -0.362 | 2.36E-17 |
| AC007879.7 | NR2F6 | -0.362 | 2.41E-17 |
| AC007879.7 | CC2D1A | -0.361 | 2.67E-17 |
| AC007879.7 | PDHA1 | -0.359 | 4.11E-17 |
| AC007879.7 | CLDN7 | -0.359 | 4.18E-17 |
| AC007879.7 | ACSS2 | -0.359 | 4.29E-17 |
| AC007879.7 | C10orf99 | -0.359 | 4.71E-17 |
| AC007879.7 | PLEKHG6 | -0.359 | 4.92E-17 |
| AC007879.7 | CLCN2 | -0.358 | 5.72E-17 |
| AC007879.7 | PRSS8 | -0.357 | 6.74E-17 |
| AC007879.7 | COX4I1 | -0.357 | 7.10E-17 |
| AC007879.7 | ATP5G1 | -0.356 | 7.63E-17 |
| AC007879.7 | FOXD2 | -0.356 | 8.29E-17 |
| AC007879.7 | DHRS11 | -0.356 | 8.70E-17 |
| AC007879.7 | CRB3 | -0.356 | 8.80E-17 |
| AC007879.7 | NDUFV1 | -0.355 | 1.13E-16 |
| AC007879.7 | AIFM3 | -0.354 | 1.27E-16 |
| AC007879.7 | PEX11A | -0.353 | 1.47E-16 |
| AC007879.7 | CES3 | -0.352 | 1.82E-16 |
| AC007879.7 | VIPR1 | -0.352 | 1.82E-16 |
| AC007879.7 | SLC44A4 | -0.351 | 2.54E-16 |
| AC007879.7 | SMDT1 | -0.351 | 2.63E-16 |
| AC007879.7 | GPT | -0.351 | 2.64E-16 |
| AC007879.7 | SLC25A10 | -0.35 | 2.88E-16 |
| AC007879.7 | GGT6 | -0.35 | 3.24E-16 |
| AC007879.7 | PKP3 | -0.349 | 3.54E-16 |
| AC007879.7 | C6orf136 | -0.349 | 3.56E-16 |
| AC007879.7 | INPP5J | -0.348 | 4.02E-16 |
| AC007879.7 | CAMSAP3 | -0.347 | 5.71E-16 |
| AC007879.7 | SLC39A5 | -0.347 | 5.82E-16 |
| AC007879.7 | EPHX2 | -0.347 | 5.88E-16 |
| AC007879.7 | TTC22 | -0.347 | 5.91E-16 |
| AC007879.7 | ASL | -0.346 | 6.59E-16 |
| AC007879.7 | CDX2 | -0.346 | 6.98E-16 |
| AC007879.7 | USH1C | -0.346 | 7.20E-16 |
| AC007879.7 | TMC4 | -0.345 | 7.68E-16 |
| AC007879.7 | SLC25A5 | -0.345 | 7.74E-16 |
| AC007879.7 | MPND | -0.345 | 7.76E-16 |
| AC007879.7 | COMTD1 | -0.345 | 7.97E-16 |
| AC007879.7 | PXMP2 | -0.344 | 9.70E-16 |
| AC007879.7 | AMN | -0.344 | 9.85E-16 |
| AC007879.7 | ALDH2 | -0.344 | 1.08E-15 |
| AC007879.7 | UQCR10 | -0.344 | 1.11E-15 |
| AC007879.7 | ECHS1 | -0.343 | 1.15E-15 |
| AC007879.7 | ETHE1 | -0.343 | 1.15E-15 |
| AC007879.7 | SLC25A1 | -0.343 | 1.24E-15 |
| AC007879.7 | SGK2 | -0.343 | 1.36E-15 |
| AC007879.7 | MYO1A | -0.342 | 1.44E-15 |
| AC007879.7 | SOWAHB | -0.342 | 1.47E-15 |
| AC007879.7 | MYO7B | -0.342 | 1.56E-15 |
| AC007879.7 | MISP | -0.342 | 1.63E-15 |
| AC007879.7 | UQCRQ | -0.341 | 1.80E-15 |
| AC007879.7 | PDZD3 | -0.34 | 2.25E-15 |
| AC007879.7 | TXN2 | -0.34 | 2.38E-15 |
| AC007879.7 | TMEM125 | -0.339 | 2.63E-15 |
| AC007879.7 | PRRG2 | -0.339 | 2.75E-15 |
| AC007879.7 | ENHO | -0.338 | 3.03E-15 |
| AC007879.7 | PRR15 | -0.338 | 3.21E-15 |
| AC007879.7 | CPTP | -0.338 | 3.21E-15 |
| AC007879.7 | CKMT1A | -0.337 | 3.75E-15 |
| AC007879.7 | DGAT1 | -0.337 | 3.87E-15 |
| AC007879.7 | TMEM53 | -0.337 | 4.38E-15 |
| AC007879.7 | ID1 | -0.336 | 4.68E-15 |
| AC007879.7 | ECI1 | -0.335 | 5.59E-15 |
| AC007879.7 | COA3 | -0.335 | 5.61E-15 |
| AC007879.7 | PWWP2B | -0.335 | 6.41E-15 |
| AC007879.7 | CKB | -0.334 | 6.82E-15 |
| AC007879.7 | NAT2 | -0.333 | 8.25E-15 |
| AC007879.7 | GJB1 | -0.333 | 8.52E-15 |
| AC007879.7 | ACO2 | -0.333 | 8.88E-15 |
| AC007879.7 | ARHGEF16 | -0.333 | 8.91E-15 |
| AC007879.7 | PCK2 | -0.333 | 9.44E-15 |
| AC007879.7 | ENDOG | -0.333 | 9.84E-15 |
| AC007879.7 | VPS51 | -0.332 | 9.95E-15 |
| AC007879.7 | ATP5D | -0.332 | 9.98E-15 |
| AC007879.7 | BAIAP2L2 | -0.332 | 1.07E-14 |
| AC007879.7 | CEBPA | -0.332 | 1.11E-14 |
| AC007879.7 | ATG4D | -0.332 | 1.15E-14 |
| AC007879.7 | TMEM238 | -0.331 | 1.33E-14 |
| AC007879.7 | CCDC64B | -0.331 | 1.41E-14 |
| AC007879.7 | GLOD5 | -0.331 | 1.42E-14 |
| AC007879.7 | IL17RE | -0.329 | 1.99E-14 |
| AC007879.7 | CYP2J2 | -0.329 | 2.02E-14 |
| AC007879.7 | C1orf210 | -0.329 | 2.10E-14 |
| AC007879.7 | GALE | -0.328 | 2.16E-14 |
| AC007879.7 | ZNF768 | -0.328 | 2.55E-14 |
| AC007879.7 | PPP1R16A | -0.327 | 2.69E-14 |
| AC007879.7 | ILVBL | -0.327 | 2.76E-14 |
| AC007879.7 | NDUFB10 | -0.327 | 2.91E-14 |
| AC007879.7 | SLC9A3R1 | -0.326 | 3.31E-14 |
| AC007879.7 | MOGAT2 | -0.325 | 4.12E-14 |
| AC007879.7 | DOK4 | -0.325 | 4.17E-14 |
| AC007879.7 | EPS8L2 | -0.325 | 4.50E-14 |
| AC007879.7 | AURKAIP1 | -0.324 | 5.29E-14 |
| AC007879.7 | AOC1 | -0.324 | 5.39E-14 |
| AC007879.7 | DECR2 | -0.323 | 5.54E-14 |
| AC007879.7 | ASB13 | -0.323 | 5.87E-14 |
| AC007879.7 | SUCLG1 | -0.322 | 7.25E-14 |
| AC007879.7 | MARVELD3 | -0.322 | 7.63E-14 |
| AC007879.7 | CACFD1 | -0.322 | 7.90E-14 |
| AC007879.7 | COX6B1 | -0.321 | 8.02E-14 |
| AC007879.7 | ELF3 | -0.32 | 9.68E-14 |
| AC007879.7 | FAM3D | -0.32 | 1.05E-13 |
| AC007879.7 | TUFM | -0.32 | 1.06E-13 |
| AC007879.7 | OVOL1 | -0.32 | 1.10E-13 |
| AC007879.7 | NUDT16L1 | -0.319 | 1.28E-13 |
| AC007879.7 | PPP1R1B | -0.319 | 1.32E-13 |
| AC007879.7 | PRDX5 | -0.319 | 1.32E-13 |
| AC007879.7 | COX6A1 | -0.318 | 1.44E-13 |
| AC007879.7 | COX8A | -0.318 | 1.46E-13 |
| AC007879.7 | OPLAH | -0.318 | 1.52E-13 |
| AC007879.7 | MRPL12 | -0.317 | 1.71E-13 |
| AC007879.7 | NDUFA2 | -0.317 | 2.00E-13 |
| AC007879.7 | ACOT11 | -0.316 | 2.02E-13 |
| AC007879.7 | SOWAHA | -0.316 | 2.03E-13 |
| AC007879.7 | SIRT6 | -0.316 | 2.16E-13 |
| AC007879.7 | MRPL41 | -0.316 | 2.30E-13 |
| AC007879.7 | ZNF524 | -0.316 | 2.33E-13 |
| AC007879.7 | SLC25A6 | -0.315 | 2.47E-13 |
| AC007879.7 | ABHD14B | -0.315 | 2.56E-13 |
| AC007879.7 | NDUFS3 | -0.315 | 2.59E-13 |
| AC007879.7 | QARS | -0.315 | 2.68E-13 |
| AC007879.7 | CYP4F12 | -0.314 | 3.20E-13 |
| AC007879.7 | ECSIT | -0.314 | 3.38E-13 |
| AC007879.7 | FUK | -0.313 | 3.76E-13 |
| AC007879.7 | CDC42EP5 | -0.313 | 3.95E-13 |
| AC007879.7 | SLC37A4 | -0.312 | 4.47E-13 |
| AC007879.7 | C2orf82 | -0.312 | 4.59E-13 |
| AC007879.7 | HMGCS2 | -0.312 | 4.78E-13 |
| AC007879.7 | CLRN3 | -0.312 | 4.84E-13 |
| AC007879.7 | ZNF784 | -0.311 | 5.20E-13 |
| AC007879.7 | FN3K | -0.311 | 5.51E-13 |
| AC007879.7 | HNF1B | -0.311 | 5.72E-13 |
| AC007879.7 | HIGD2A | -0.31 | 6.01E-13 |
| AC007879.7 | RASSF7 | -0.31 | 6.35E-13 |
| AC007879.7 | METTL7B | -0.31 | 6.70E-13 |
| AC007879.7 | ILDR1 | -0.309 | 7.26E-13 |
| AC007879.7 | DNPH1 | -0.309 | 7.56E-13 |
| AC007879.7 | ATP5G3 | -0.309 | 8.00E-13 |
| AC007879.7 | BTNL3 | -0.309 | 8.32E-13 |
| AC007879.7 | STARD10 | -0.309 | 8.42E-13 |
| AC007879.7 | C19orf70 | -0.309 | 8.49E-13 |
| AC007879.7 | SLC26A6 | -0.308 | 8.91E-13 |
| AC007879.7 | LDHD | -0.308 | 8.93E-13 |
| AC007879.7 | RAB40C | -0.308 | 9.91E-13 |
| AC007879.7 | PRAP1 | -0.307 | 1.03E-12 |
| AC007879.7 | PIGZ | -0.307 | 1.11E-12 |
| AC007879.7 | RNF186 | -0.307 | 1.13E-12 |
| AC007879.7 | CYC1 | -0.307 | 1.18E-12 |
| AC007879.7 | CKMT1B | -0.306 | 1.24E-12 |
| AC007879.7 | PAQR8 | -0.306 | 1.25E-12 |
| AC007879.7 | C8orf82 | -0.306 | 1.27E-12 |
| AC007879.7 | NDUFS6 | -0.306 | 1.34E-12 |
| AC007879.7 | ZDHHC12 | -0.306 | 1.37E-12 |
| AC007879.7 | LAMTOR4 | -0.306 | 1.40E-12 |
| AC007879.7 | ATPIF1 | -0.306 | 1.44E-12 |
| AC007879.7 | CASP6 | -0.305 | 1.53E-12 |
| AC007879.7 | TMEM45B | -0.305 | 1.58E-12 |
| AC007879.7 | PICK1 | -0.305 | 1.73E-12 |
| AC007879.7 | SCO2 | -0.304 | 1.80E-12 |
| AC007879.7 | MRPS34 | -0.304 | 1.81E-12 |
| AC007879.7 | NR1I2 | -0.304 | 1.84E-12 |
| AC007879.7 | QTRT1 | -0.304 | 1.95E-12 |
| AC007879.7 | ZNF787 | -0.304 | 2.01E-12 |
| AC007879.7 | ANKS4B | -0.304 | 2.03E-12 |
| AC007879.7 | ATP5B | -0.303 | 2.08E-12 |
| AC007879.7 | NRARP | -0.303 | 2.20E-12 |
| AC007879.7 | NDUFB7 | -0.303 | 2.32E-12 |
| AC007879.7 | CHP2 | -0.303 | 2.44E-12 |
| AC007879.7 | EPB41L4B | -0.302 | 2.48E-12 |
| AC007879.7 | GFER | -0.302 | 2.58E-12 |
| AC007879.7 | PFKL | -0.302 | 2.58E-12 |
| AC007879.7 | RPS6KA1 | -0.302 | 2.69E-12 |
| AC007879.7 | AGMAT | -0.302 | 2.69E-12 |
| AC007879.7 | TMEM82 | -0.302 | 2.74E-12 |
| AC007879.7 | TEX264 | -0.302 | 2.84E-12 |
| AC007879.7 | PRR15L | -0.302 | 2.87E-12 |
| AC007879.7 | SCAND1 | -0.301 | 3.05E-12 |
| AC007879.7 | NDUFB11 | -0.301 | 3.32E-12 |
| AC007879.7 | NDUFA3 | -0.301 | 3.34E-12 |
| AC007879.7 | MAP2K2 | -0.301 | 3.43E-12 |
| AC007879.7 | MYH2 | 0.301 | 2.92E-12 |
| AC007879.7 | PPP1R42 | 0.301 | 2.93E-12 |
| AC007879.7 | CRYAB | 0.301 | 2.96E-12 |
| AC007879.7 | TET2 | 0.301 | 2.98E-12 |
| AC007879.7 | JAM2 | 0.301 | 3.01E-12 |
| AC007879.7 | LATS1 | 0.301 | 3.01E-12 |
| AC007879.7 | AC007040.11 | 0.301 | 3.01E-12 |
| AC007879.7 | SPIRE1 | 0.301 | 3.04E-12 |
| AC007879.7 | MAP3K2 | 0.301 | 3.08E-12 |
| AC007879.7 | ADAM30 | 0.301 | 3.10E-12 |
| AC007879.7 | LRCH3 | 0.301 | 3.12E-12 |
| AC007879.7 | SLF2 | 0.301 | 3.12E-12 |
| AC007879.7 | NBPF12 | 0.301 | 3.12E-12 |
| AC007879.7 | PTPRG | 0.301 | 3.16E-12 |
| AC007879.7 | HIGD1C | 0.301 | 3.21E-12 |
| AC007879.7 | BMP2K | 0.301 | 3.22E-12 |
| AC007879.7 | RAP2B | 0.301 | 3.24E-12 |
| AC007879.7 | FBXW2 | 0.301 | 3.26E-12 |
| AC007879.7 | RLF | 0.301 | 3.27E-12 |
| AC007879.7 | FAM13C | 0.301 | 3.31E-12 |
| AC007879.7 | PRSS37 | 0.301 | 3.31E-12 |
| AC007879.7 | GAPT | 0.301 | 3.32E-12 |
| AC007879.7 | PHYHIP | 0.301 | 3.38E-12 |
| AC007879.7 | SDR9C7 | 0.302 | 2.47E-12 |
| AC007879.7 | IGFL1 | 0.302 | 2.48E-12 |
| AC007879.7 | PHC1 | 0.302 | 2.53E-12 |
| AC007879.7 | SCN1B | 0.302 | 2.56E-12 |
| AC007879.7 | REST | 0.302 | 2.57E-12 |
| AC007879.7 | PLS3 | 0.302 | 2.58E-12 |
| AC007879.7 | KIF3A | 0.302 | 2.59E-12 |
| AC007879.7 | CEP350 | 0.302 | 2.62E-12 |
| AC007879.7 | REM1 | 0.302 | 2.63E-12 |
| AC007879.7 | SERPINB12 | 0.302 | 2.64E-12 |
| AC007879.7 | SLC7A7 | 0.302 | 2.65E-12 |
| AC007879.7 | IFNA5 | 0.302 | 2.66E-12 |
| AC007879.7 | PRSS35 | 0.302 | 2.70E-12 |
| AC007879.7 | ATF7 | 0.302 | 2.70E-12 |
| AC007879.7 | ZBTB34 | 0.302 | 2.72E-12 |
| AC007879.7 | DNAJC10 | 0.302 | 2.73E-12 |
| AC007879.7 | MEPE | 0.302 | 2.74E-12 |
| AC007879.7 | DNAH12 | 0.302 | 2.75E-12 |
| AC007879.7 | CPXM2 | 0.302 | 2.76E-12 |
| AC007879.7 | CARD6 | 0.302 | 2.79E-12 |
| AC007879.7 | SLC16A2 | 0.302 | 2.83E-12 |
| AC007879.7 | TMED7-TICAM2 | 0.302 | 2.85E-12 |
| AC007879.7 | FCN3 | 0.302 | 2.87E-12 |
| AC007879.7 | TMPRSS11D | 0.302 | 2.89E-12 |
| AC007879.7 | ZNF618 | 0.302 | 2.90E-12 |
| AC007879.7 | ZNF229 | 0.303 | 2.07E-12 |
| AC007879.7 | SEC22B | 0.303 | 2.07E-12 |
| AC007879.7 | TSPAN2 | 0.303 | 2.08E-12 |
| AC007879.7 | SLITRK2 | 0.303 | 2.08E-12 |
| AC007879.7 | NGF | 0.303 | 2.09E-12 |
| AC007879.7 | PATE4 | 0.303 | 2.10E-12 |
| AC007879.7 | CCDC62 | 0.303 | 2.11E-12 |
| AC007879.7 | POLN | 0.303 | 2.13E-12 |
| AC007879.7 | CCDC67 | 0.303 | 2.13E-12 |
| AC007879.7 | MTMR2 | 0.303 | 2.15E-12 |
| AC007879.7 | CETN1 | 0.303 | 2.15E-12 |
| AC007879.7 | SLC36A3 | 0.303 | 2.18E-12 |
| AC007879.7 | TSPAN18 | 0.303 | 2.18E-12 |
| AC007879.7 | ZBTB8A | 0.303 | 2.21E-12 |
| AC007879.7 | ZNF385A | 0.303 | 2.21E-12 |
| AC007879.7 | CCDC172 | 0.303 | 2.25E-12 |
| AC007879.7 | PCDHB10 | 0.303 | 2.25E-12 |
| AC007879.7 | C8orf88 | 0.303 | 2.26E-12 |
| AC007879.7 | CORO1C | 0.303 | 2.27E-12 |
| AC007879.7 | RICTOR | 0.303 | 2.27E-12 |
| AC007879.7 | ITSN1 | 0.303 | 2.27E-12 |
| AC007879.7 | AC005477.1 | 0.303 | 2.27E-12 |
| AC007879.7 | NDST3 | 0.303 | 2.28E-12 |
| AC007879.7 | NAP1L5 | 0.303 | 2.33E-12 |
| AC007879.7 | OSBPL10 | 0.303 | 2.33E-12 |
| AC007879.7 | CCBE1 | 0.303 | 2.34E-12 |
| AC007879.7 | KLHL2 | 0.303 | 2.34E-12 |
| AC007879.7 | FAM205C | 0.303 | 2.35E-12 |
| AC007879.7 | DLGAP2 | 0.303 | 2.35E-12 |
| AC007879.7 | KPNA1 | 0.303 | 2.36E-12 |
| AC007879.7 | OR13D1 | 0.303 | 2.39E-12 |
| AC007879.7 | INPP5B | 0.303 | 2.39E-12 |
| AC007879.7 | TNKS2 | 0.303 | 2.42E-12 |
| AC007879.7 | SLC13A4 | 0.303 | 2.43E-12 |
| AC007879.7 | DCHS1 | 0.304 | 1.76E-12 |
| AC007879.7 | OR51M1 | 0.304 | 1.79E-12 |
| AC007879.7 | POMK | 0.304 | 1.79E-12 |
| AC007879.7 | NEK1 | 0.304 | 1.80E-12 |
| AC007879.7 | GOLGA8N | 0.304 | 1.80E-12 |
| AC007879.7 | WTAP | 0.304 | 1.87E-12 |
| AC007879.7 | AL138751.1 | 0.304 | 1.88E-12 |
| AC007879.7 | PLEKHA8 | 0.304 | 1.88E-12 |
| AC007879.7 | STYX | 0.304 | 1.89E-12 |
| AC007879.7 | PPP4R1 | 0.304 | 1.89E-12 |
| AC007879.7 | RGS19 | 0.304 | 1.90E-12 |
| AC007879.7 | THBS4 | 0.304 | 1.90E-12 |
| AC007879.7 | ADORA2A | 0.304 | 1.90E-12 |
| AC007879.7 | ATP5L2 | 0.304 | 1.91E-12 |
| AC007879.7 | GIMAP8 | 0.304 | 1.92E-12 |
| AC007879.7 | CHORDC1 | 0.304 | 1.93E-12 |
| AC007879.7 | CDK15 | 0.304 | 1.93E-12 |
| AC007879.7 | SLC9C2 | 0.304 | 1.95E-12 |
| AC007879.7 | AC011380.1 | 0.304 | 1.95E-12 |
| AC007879.7 | BLOC1S6 | 0.304 | 1.97E-12 |
| AC007879.7 | PLA2G5 | 0.304 | 1.98E-12 |
| AC007879.7 | PAX1 | 0.304 | 2.00E-12 |
| AC007879.7 | IMPAD1 | 0.304 | 2.00E-12 |
| AC007879.7 | SLC15A4 | 0.304 | 2.03E-12 |
| AC007879.7 | GYPA | 0.304 | 2.04E-12 |
| AC007879.7 | SLC12A5 | 0.304 | 2.05E-12 |
| AC007879.7 | MMP12 | 0.305 | 1.46E-12 |
| AC007879.7 | EIF5A2 | 0.305 | 1.46E-12 |
| AC007879.7 | FAM120C | 0.305 | 1.46E-12 |
| AC007879.7 | PRKAB2 | 0.305 | 1.49E-12 |
| AC007879.7 | CALB2 | 0.305 | 1.51E-12 |
| AC007879.7 | TMEM95 | 0.305 | 1.53E-12 |
| AC007879.7 | ANKRD36B | 0.305 | 1.54E-12 |
| AC007879.7 | GPR132 | 0.305 | 1.54E-12 |
| AC007879.7 | TTL | 0.305 | 1.56E-12 |
| AC007879.7 | NBPF10 | 0.305 | 1.56E-12 |
| AC007879.7 | LEUTX | 0.305 | 1.57E-12 |
| AC007879.7 | MS4A6A | 0.305 | 1.58E-12 |
| AC007879.7 | RNF146 | 0.305 | 1.59E-12 |
| AC007879.7 | TIMM23B | 0.305 | 1.60E-12 |
| AC007879.7 | ZNF436 | 0.305 | 1.61E-12 |
| AC007879.7 | C8B | 0.305 | 1.61E-12 |
| AC007879.7 | TANK | 0.305 | 1.62E-12 |
| AC007879.7 | USP37 | 0.305 | 1.62E-12 |
| AC007879.7 | CASP8AP2 | 0.305 | 1.64E-12 |
| AC007879.7 | PPM1K | 0.305 | 1.65E-12 |
| AC007879.7 | DHX36 | 0.305 | 1.66E-12 |
| AC007879.7 | VPS8 | 0.305 | 1.68E-12 |
| AC007879.7 | AL928654.7 | 0.305 | 1.68E-12 |
| AC007879.7 | ITK | 0.305 | 1.68E-12 |
| AC007879.7 | LDHAL6A | 0.305 | 1.69E-12 |
| AC007879.7 | SHISA2 | 0.305 | 1.70E-12 |
| AC007879.7 | FAM106A | 0.306 | 1.23E-12 |
| AC007879.7 | ZNF788 | 0.306 | 1.24E-12 |
| AC007879.7 | 11-Sep | 0.306 | 1.24E-12 |
| AC007879.7 | PIP5K1A | 0.306 | 1.25E-12 |
| AC007879.7 | PRELP | 0.306 | 1.26E-12 |
| AC007879.7 | LCTL | 0.306 | 1.27E-12 |
| AC007879.7 | BAG2 | 0.306 | 1.27E-12 |
| AC007879.7 | ESCO1 | 0.306 | 1.28E-12 |
| AC007879.7 | CAP2 | 0.306 | 1.29E-12 |
| AC007879.7 | ZNF430 | 0.306 | 1.30E-12 |
| AC007879.7 | TMEM217 | 0.306 | 1.30E-12 |
| AC007879.7 | FOXN2 | 0.306 | 1.30E-12 |
| AC007879.7 | AWAT1 | 0.306 | 1.31E-12 |
| AC007879.7 | DDX4 | 0.306 | 1.32E-12 |
| AC007879.7 | C2orf73 | 0.306 | 1.32E-12 |
| AC007879.7 | DENND4A | 0.306 | 1.33E-12 |
| AC007879.7 | AMN1 | 0.306 | 1.35E-12 |
| AC007879.7 | TRPV5 | 0.306 | 1.36E-12 |
| AC007879.7 | L3HYPDH | 0.306 | 1.36E-12 |
| AC007879.7 | SPATA8 | 0.306 | 1.36E-12 |
| AC007879.7 | GABRQ | 0.306 | 1.37E-12 |
| AC007879.7 | SIAH3 | 0.306 | 1.39E-12 |
| AC007879.7 | CILP | 0.306 | 1.41E-12 |
| AC007879.7 | SYNJ1 | 0.306 | 1.41E-12 |
| AC007879.7 | CYP4A22 | 0.306 | 1.43E-12 |
| AC007879.7 | OR2D2 | 0.306 | 1.44E-12 |
| AC007879.7 | SLC1A2 | 0.306 | 1.45E-12 |
| AC007879.7 | KIAA1551 | 0.306 | 1.45E-12 |
| AC007879.7 | DOCK9 | 0.307 | 1.02E-12 |
| AC007879.7 | DNAJB6 | 0.307 | 1.03E-12 |
| AC007879.7 | GATAD2B | 0.307 | 1.06E-12 |
| AC007879.7 | COL27A1 | 0.307 | 1.06E-12 |
| AC007879.7 | USP50 | 0.307 | 1.07E-12 |
| AC007879.7 | HVCN1 | 0.307 | 1.07E-12 |
| AC007879.7 | RP11-219A15.1 | 0.307 | 1.07E-12 |
| AC007879.7 | BBOX1 | 0.307 | 1.07E-12 |
| AC007879.7 | NAGK | 0.307 | 1.08E-12 |
| AC007879.7 | C7orf25 | 0.307 | 1.09E-12 |
| AC007879.7 | PROX2 | 0.307 | 1.09E-12 |
| AC007879.7 | SPAG17 | 0.307 | 1.09E-12 |
| AC007879.7 | C9orf72 | 0.307 | 1.10E-12 |
| AC007879.7 | CRABP2 | 0.307 | 1.11E-12 |
| AC007879.7 | TAS2R46 | 0.307 | 1.13E-12 |
| AC007879.7 | MAST4 | 0.307 | 1.14E-12 |
| AC007879.7 | CCR5 | 0.307 | 1.15E-12 |
| AC007879.7 | CEP57L1 | 0.307 | 1.16E-12 |
| AC007879.7 | MOG | 0.307 | 1.16E-12 |
| AC007879.7 | LMOD2 | 0.307 | 1.17E-12 |
| AC007879.7 | TAS2R42 | 0.307 | 1.17E-12 |
| AC007879.7 | EMILIN1 | 0.307 | 1.18E-12 |
| AC007879.7 | RNF165 | 0.307 | 1.20E-12 |
| AC007879.7 | LST1 | 0.307 | 1.20E-12 |
| AC007879.7 | OR2A2 | 0.308 | 8.57E-13 |
| AC007879.7 | RCBTB2 | 0.308 | 8.61E-13 |
| AC007879.7 | TSC22D3 | 0.308 | 8.74E-13 |
| AC007879.7 | LEPR | 0.308 | 8.75E-13 |
| AC007879.7 | ARHGAP20 | 0.308 | 8.78E-13 |
| AC007879.7 | LYPD1 | 0.308 | 8.87E-13 |
| AC007879.7 | OR5F1 | 0.308 | 8.94E-13 |
| AC007879.7 | LY86 | 0.308 | 8.95E-13 |
| AC007879.7 | PPP1R3C | 0.308 | 9.07E-13 |
| AC007879.7 | MAP3K3 | 0.308 | 9.10E-13 |
| AC007879.7 | ARR3 | 0.308 | 9.16E-13 |
| AC007879.7 | TACR1 | 0.308 | 9.21E-13 |
| AC007879.7 | HAMP | 0.308 | 9.21E-13 |
| AC007879.7 | GADD45B | 0.308 | 9.30E-13 |
| AC007879.7 | ZNF555 | 0.308 | 9.63E-13 |
| AC007879.7 | CHRM5 | 0.308 | 9.69E-13 |
| AC007879.7 | RC3H2 | 0.308 | 9.74E-13 |
| AC007879.7 | ADAM29 | 0.308 | 9.94E-13 |
| AC007879.7 | CYP2E1 | 0.308 | 9.95E-13 |
| AC007879.7 | TTC7B | 0.308 | 9.96E-13 |
| AC007879.7 | VEZT | 0.308 | 9.99E-13 |
| AC007879.7 | SAMHD1 | 0.308 | 1.00E-12 |
| AC007879.7 | RP3-403A15.5 | 0.309 | 7.35E-13 |
| AC007879.7 | RERG | 0.309 | 7.35E-13 |
| AC007879.7 | IQCJ-SCHIP1 | 0.309 | 7.36E-13 |
| AC007879.7 | OVCH2 | 0.309 | 7.42E-13 |
| AC007879.7 | GAPVD1 | 0.309 | 7.45E-13 |
| AC007879.7 | EMP3 | 0.309 | 7.45E-13 |
| AC007879.7 | CD28 | 0.309 | 7.45E-13 |
| AC007879.7 | C15orf53 | 0.309 | 7.46E-13 |
| AC007879.7 | DNM1 | 0.309 | 7.52E-13 |
| AC007879.7 | GRAMD1B | 0.309 | 7.53E-13 |
| AC007879.7 | CLCF1 | 0.309 | 7.54E-13 |
| AC007879.7 | INSIG2 | 0.309 | 7.64E-13 |
| AC007879.7 | TBC1D8 | 0.309 | 7.68E-13 |
| AC007879.7 | KIAA0040 | 0.309 | 7.88E-13 |
| AC007879.7 | TMCO5A | 0.309 | 7.93E-13 |
| AC007879.7 | MEI4 | 0.309 | 8.00E-13 |
| AC007879.7 | HIVEP1 | 0.309 | 8.03E-13 |
| AC007879.7 | FP325331.1 | 0.309 | 8.04E-13 |
| AC007879.7 | ECT2L | 0.309 | 8.06E-13 |
| AC007879.7 | C18orf63 | 0.309 | 8.11E-13 |
| AC007879.7 | TRIM22 | 0.309 | 8.12E-13 |
| AC007879.7 | SAMD9L | 0.309 | 8.13E-13 |
| AC007879.7 | PSG5 | 0.309 | 8.14E-13 |
| AC007879.7 | KDSR | 0.309 | 8.38E-13 |
| AC007879.7 | PTPDC1 | 0.309 | 8.42E-13 |
| AC007879.7 | JMJD1C | 0.309 | 8.42E-13 |
| AC007879.7 | ZNF573 | 0.309 | 8.47E-13 |
| AC007879.7 | RGS8 | 0.31 | 5.99E-13 |
| AC007879.7 | GJA4 | 0.31 | 6.05E-13 |
| AC007879.7 | TPH2 | 0.31 | 6.10E-13 |
| AC007879.7 | CHRNA3 | 0.31 | 6.14E-13 |
| AC007879.7 | RAB11FIP2 | 0.31 | 6.27E-13 |
| AC007879.7 | TRIM51 | 0.31 | 6.35E-13 |
| AC007879.7 | KIF3C | 0.31 | 6.46E-13 |
| AC007879.7 | OR52B2 | 0.31 | 6.47E-13 |
| AC007879.7 | ZNF407 | 0.31 | 6.48E-13 |
| AC007879.7 | NECAB1 | 0.31 | 6.53E-13 |
| AC007879.7 | CFHR5 | 0.31 | 6.57E-13 |
| AC007879.7 | FNDC9 | 0.31 | 6.58E-13 |
| AC007879.7 | DCLK1 | 0.31 | 6.62E-13 |
| AC007879.7 | RNMT | 0.31 | 6.73E-13 |
| AC007879.7 | AC055733.1 | 0.31 | 6.92E-13 |
| AC007879.7 | ACAN | 0.31 | 6.99E-13 |
| AC007879.7 | C9orf131 | 0.311 | 5.01E-13 |
| AC007879.7 | MYH1 | 0.311 | 5.07E-13 |
| AC007879.7 | WDR91 | 0.311 | 5.08E-13 |
| AC007879.7 | TMC3 | 0.311 | 5.08E-13 |
| AC007879.7 | ERO1B | 0.311 | 5.10E-13 |
| AC007879.7 | C10orf113 | 0.311 | 5.13E-13 |
| AC007879.7 | CCDC178 | 0.311 | 5.26E-13 |
| AC007879.7 | SEMA3A | 0.311 | 5.26E-13 |
| AC007879.7 | PDE4DIP | 0.311 | 5.39E-13 |
| AC007879.7 | CLUL1 | 0.311 | 5.43E-13 |
| AC007879.7 | OVCH1 | 0.311 | 5.43E-13 |
| AC007879.7 | RCBTB1 | 0.311 | 5.45E-13 |
| AC007879.7 | SDS | 0.311 | 5.51E-13 |
| AC007879.7 | UCP1 | 0.311 | 5.52E-13 |
| AC007879.7 | CFL2 | 0.311 | 5.58E-13 |
| AC007879.7 | ERRFI1 | 0.311 | 5.59E-13 |
| AC007879.7 | PATL2 | 0.311 | 5.62E-13 |
| AC007879.7 | CMKLR1 | 0.311 | 5.62E-13 |
| AC007879.7 | XIRP2 | 0.311 | 5.63E-13 |
| AC007879.7 | AC092881.1 | 0.311 | 5.70E-13 |
| AC007879.7 | CFHR2 | 0.311 | 5.72E-13 |
| AC007879.7 | TAS2R3 | 0.311 | 5.76E-13 |
| AC007879.7 | TRERF1 | 0.311 | 5.79E-13 |
| AC007879.7 | SYT3 | 0.311 | 5.84E-13 |
| AC007879.7 | TMOD2 | 0.311 | 5.85E-13 |
| AC007879.7 | KCNK12 | 0.311 | 5.87E-13 |
| AC007879.7 | METTL21C | 0.311 | 5.90E-13 |
| AC007879.7 | MSANTD3-TMEFF1 | 0.311 | 5.94E-13 |
| AC007879.7 | CFAP206 | 0.312 | 4.18E-13 |
| AC007879.7 | AC012363.2 | 0.312 | 4.19E-13 |
| AC007879.7 | B3GAT2 | 0.312 | 4.21E-13 |
| AC007879.7 | RASSF5 | 0.312 | 4.32E-13 |
| AC007879.7 | CCDC40 | 0.312 | 4.35E-13 |
| AC007879.7 | ZC3H11A | 0.312 | 4.39E-13 |
| AC007879.7 | FSHB | 0.312 | 4.40E-13 |
| AC007879.7 | GSG1 | 0.312 | 4.44E-13 |
| AC007879.7 | WDR7 | 0.312 | 4.46E-13 |
| AC007879.7 | CLIP1 | 0.312 | 4.48E-13 |
| AC007879.7 | LAMB2 | 0.312 | 4.52E-13 |
| AC007879.7 | SPIN2A | 0.312 | 4.57E-13 |
| AC007879.7 | ZSCAN1 | 0.312 | 4.59E-13 |
| AC007879.7 | FZD1 | 0.312 | 4.62E-13 |
| AC007879.7 | IL5 | 0.312 | 4.70E-13 |
| AC007879.7 | FBXO11 | 0.312 | 4.70E-13 |
| AC007879.7 | TPD52L3 | 0.312 | 4.78E-13 |
| AC007879.7 | VAX2 | 0.312 | 4.84E-13 |
| AC007879.7 | ZSWIM4 | 0.312 | 4.85E-13 |
| AC007879.7 | TAS2R30 | 0.312 | 4.86E-13 |
| AC007879.7 | SP100 | 0.312 | 4.87E-13 |
| AC007879.7 | OR14A2 | 0.312 | 4.90E-13 |
| AC007879.7 | SEC14L2 | 0.312 | 4.90E-13 |
| AC007879.7 | TCP11L1 | 0.312 | 4.99E-13 |
| AC007879.7 | GNA12 | 0.313 | 3.49E-13 |
| AC007879.7 | ERC2 | 0.313 | 3.56E-13 |
| AC007879.7 | DPYS | 0.313 | 3.57E-13 |
| AC007879.7 | AC006994.3 | 0.313 | 3.57E-13 |
| AC007879.7 | COPS8 | 0.313 | 3.57E-13 |
| AC007879.7 | SIGLEC10 | 0.313 | 3.58E-13 |
| AC007879.7 | EGR3 | 0.313 | 3.63E-13 |
| AC007879.7 | OPRK1 | 0.313 | 3.65E-13 |
| AC007879.7 | ZNF292 | 0.313 | 3.66E-13 |
| AC007879.7 | TSNAX-DISC1 | 0.313 | 3.70E-13 |
| AC007879.7 | UVRAG | 0.313 | 3.77E-13 |
| AC007879.7 | UHRF1BP1 | 0.313 | 3.78E-13 |
| AC007879.7 | TRDMT1 | 0.313 | 3.83E-13 |
| AC007879.7 | RNASE9 | 0.313 | 3.84E-13 |
| AC007879.7 | EID2B | 0.313 | 3.92E-13 |
| AC007879.7 | C7orf60 | 0.313 | 3.93E-13 |
| AC007879.7 | HSPB2 | 0.313 | 3.94E-13 |
| AC007879.7 | ZNF611 | 0.313 | 3.96E-13 |
| AC007879.7 | KLHL20 | 0.313 | 3.97E-13 |
| AC007879.7 | SPATA22 | 0.313 | 4.00E-13 |
| AC007879.7 | OR9A4 | 0.313 | 4.01E-13 |
| AC007879.7 | SELP | 0.313 | 4.03E-13 |
| AC007879.7 | STXBP4 | 0.313 | 4.07E-13 |
| AC007879.7 | SYPL2 | 0.313 | 4.16E-13 |
| AC007879.7 | FAM65C | 0.314 | 2.91E-13 |
| AC007879.7 | TMEM247 | 0.314 | 3.03E-13 |
| AC007879.7 | ADAD1 | 0.314 | 3.04E-13 |
| AC007879.7 | OR13G1 | 0.314 | 3.07E-13 |
| AC007879.7 | IFT81 | 0.314 | 3.09E-13 |
| AC007879.7 | ZFP36L1 | 0.314 | 3.13E-13 |
| AC007879.7 | ZNF750 | 0.314 | 3.20E-13 |
| AC007879.7 | GATS | 0.314 | 3.21E-13 |
| AC007879.7 | BPI | 0.314 | 3.21E-13 |
| AC007879.7 | OR8S1 | 0.314 | 3.23E-13 |
| AC007879.7 | DST | 0.314 | 3.29E-13 |
| AC007879.7 | NBPF20 | 0.314 | 3.32E-13 |
| AC007879.7 | STARD6 | 0.314 | 3.39E-13 |
| AC007879.7 | SGCZ | 0.314 | 3.42E-13 |
| AC007879.7 | SMIM13 | 0.314 | 3.43E-13 |
| AC007879.7 | PIWIL3 | 0.314 | 3.44E-13 |
| AC007879.7 | DNAH10OS | 0.314 | 3.47E-13 |
| AC007879.7 | GBP6 | 0.315 | 2.45E-13 |
| AC007879.7 | SPIN1 | 0.315 | 2.49E-13 |
| AC007879.7 | ODF3 | 0.315 | 2.52E-13 |
| AC007879.7 | EXOC5 | 0.315 | 2.53E-13 |
| AC007879.7 | ITGB8 | 0.315 | 2.59E-13 |
| AC007879.7 | SPDYA | 0.315 | 2.60E-13 |
| AC007879.7 | S100A9 | 0.315 | 2.62E-13 |
| AC007879.7 | P2RY6 | 0.315 | 2.66E-13 |
| AC007879.7 | SAV1 | 0.315 | 2.68E-13 |
| AC007879.7 | BOLL | 0.315 | 2.70E-13 |
| AC007879.7 | STEAP2 | 0.315 | 2.75E-13 |
| AC007879.7 | CCDC179 | 0.315 | 2.76E-13 |
| AC007879.7 | OR5V1 | 0.315 | 2.78E-13 |
| AC007879.7 | TMEM239 | 0.315 | 2.80E-13 |
| AC007879.7 | ZNF641 | 0.315 | 2.81E-13 |
| AC007879.7 | DCP2 | 0.315 | 2.82E-13 |
| AC007879.7 | ELMOD1 | 0.315 | 2.85E-13 |
| AC007879.7 | PLPPR2 | 0.315 | 2.87E-13 |
| AC007879.7 | UBQLN3 | 0.315 | 2.89E-13 |
| AC007879.7 | TAS2R14 | 0.316 | 2.02E-13 |
| AC007879.7 | SPI1 | 0.316 | 2.02E-13 |
| AC007879.7 | BLOC1S5-TXNDC5 | 0.316 | 2.03E-13 |
| AC007879.7 | CRY1 | 0.316 | 2.07E-13 |
| AC007879.7 | C1orf145 | 0.316 | 2.08E-13 |
| AC007879.7 | SHPRH | 0.316 | 2.08E-13 |
| AC007879.7 | MC2R | 0.316 | 2.09E-13 |
| AC007879.7 | EFCAB1 | 0.316 | 2.09E-13 |
| AC007879.7 | BSPH1 | 0.316 | 2.12E-13 |
| AC007879.7 | GPR34 | 0.316 | 2.14E-13 |
| AC007879.7 | TDRD15 | 0.316 | 2.14E-13 |
| AC007879.7 | RUFY3 | 0.316 | 2.15E-13 |
| AC007879.7 | P4HA1 | 0.316 | 2.16E-13 |
| AC007879.7 | PRKCDBP | 0.316 | 2.22E-13 |
| AC007879.7 | TMCC1 | 0.316 | 2.23E-13 |
| AC007879.7 | S100G | 0.316 | 2.23E-13 |
| AC007879.7 | ALS2CR11 | 0.316 | 2.26E-13 |
| AC007879.7 | CXCL9 | 0.316 | 2.34E-13 |
| AC007879.7 | CLIC2 | 0.316 | 2.35E-13 |
| AC007879.7 | SIGLECL1 | 0.316 | 2.36E-13 |
| AC007879.7 | KCNJ6 | 0.316 | 2.36E-13 |
| AC007879.7 | TLR5 | 0.316 | 2.38E-13 |
| AC007879.7 | FKBP10 | 0.316 | 2.40E-13 |
| AC007879.7 | USP34 | 0.316 | 2.41E-13 |
| AC007879.7 | DENND2C | 0.317 | 1.68E-13 |
| AC007879.7 | PLA2G4C | 0.317 | 1.70E-13 |
| AC007879.7 | MS4A5 | 0.317 | 1.70E-13 |
| AC007879.7 | KIAA1586 | 0.317 | 1.71E-13 |
| AC007879.7 | EXTL2 | 0.317 | 1.72E-13 |
| AC007879.7 | TAS2R19 | 0.317 | 1.75E-13 |
| AC007879.7 | ZNF451 | 0.317 | 1.79E-13 |
| AC007879.7 | SMIM18 | 0.317 | 1.80E-13 |
| AC007879.7 | CLASP1 | 0.317 | 1.80E-13 |
| AC007879.7 | CRLF2 | 0.317 | 1.81E-13 |
| AC007879.7 | FHL5 | 0.317 | 1.81E-13 |
| AC007879.7 | DEFB108B | 0.317 | 1.86E-13 |
| AC007879.7 | TIGIT | 0.317 | 1.87E-13 |
| AC007879.7 | PGM3 | 0.317 | 1.90E-13 |
| AC007879.7 | HSPA13 | 0.317 | 1.92E-13 |
| AC007879.7 | PREX1 | 0.317 | 1.93E-13 |
| AC007879.7 | SLC25A30 | 0.317 | 1.98E-13 |
| AC007879.7 | PLEKHH2 | 0.317 | 1.99E-13 |
| AC007879.7 | IFNK | 0.317 | 2.01E-13 |
| AC007879.7 | EFCAB12 | 0.317 | 2.01E-13 |
| AC007879.7 | NAALAD2 | 0.318 | 1.42E-13 |
| AC007879.7 | VAMP4 | 0.318 | 1.43E-13 |
| AC007879.7 | ZNF805 | 0.318 | 1.45E-13 |
| AC007879.7 | CC2D2A | 0.318 | 1.45E-13 |
| AC007879.7 | LDHAL6B | 0.318 | 1.46E-13 |
| AC007879.7 | CRLF3 | 0.318 | 1.46E-13 |
| AC007879.7 | CRYBG3 | 0.318 | 1.46E-13 |
| AC007879.7 | PLEKHD1 | 0.318 | 1.46E-13 |
| AC007879.7 | PATE1 | 0.318 | 1.47E-13 |
| AC007879.7 | RNGTT | 0.318 | 1.47E-13 |
| AC007879.7 | DBX2 | 0.318 | 1.48E-13 |
| AC007879.7 | GNAI2 | 0.318 | 1.48E-13 |
| AC007879.7 | SRGAP1 | 0.318 | 1.48E-13 |
| AC007879.7 | AC069063.2 | 0.318 | 1.49E-13 |
| AC007879.7 | INTU | 0.318 | 1.49E-13 |
| AC007879.7 | TAS2R43 | 0.318 | 1.49E-13 |
| AC007879.7 | DCDC2B | 0.318 | 1.53E-13 |
| AC007879.7 | GYS2 | 0.318 | 1.56E-13 |
| AC007879.7 | AIF1 | 0.318 | 1.56E-13 |
| AC007879.7 | CLIP3 | 0.318 | 1.56E-13 |
| AC007879.7 | RNF169 | 0.318 | 1.57E-13 |
| AC007879.7 | USH2A | 0.318 | 1.58E-13 |
| AC007879.7 | TMEM212 | 0.318 | 1.58E-13 |
| AC007879.7 | GM2A | 0.318 | 1.62E-13 |
| AC007879.7 | GCNT7 | 0.318 | 1.62E-13 |
| AC007879.7 | ANO2 | 0.318 | 1.64E-13 |
| AC007879.7 | AC018902.1 | 0.318 | 1.67E-13 |
| AC007879.7 | SH3BP5 | 0.319 | 1.17E-13 |
| AC007879.7 | ARNTL | 0.319 | 1.17E-13 |
| AC007879.7 | CHRNB3 | 0.319 | 1.21E-13 |
| AC007879.7 | DIXDC1 | 0.319 | 1.23E-13 |
| AC007879.7 | GXYLT2 | 0.319 | 1.23E-13 |
| AC007879.7 | SLC30A7 | 0.319 | 1.26E-13 |
| AC007879.7 | FAM198A | 0.319 | 1.27E-13 |
| AC007879.7 | UPK1B | 0.319 | 1.28E-13 |
| AC007879.7 | RP11-310N16.1 | 0.319 | 1.31E-13 |
| AC007879.7 | GCKR | 0.319 | 1.32E-13 |
| AC007879.7 | EFCAB3 | 0.319 | 1.33E-13 |
| AC007879.7 | TOPAZ1 | 0.319 | 1.36E-13 |
| AC007879.7 | HFM1 | 0.319 | 1.39E-13 |
| AC007879.7 | 14-Sep | 0.32 | 9.67E-14 |
| AC007879.7 | LRRD1 | 0.32 | 9.77E-14 |
| AC007879.7 | SP4 | 0.32 | 1.00E-13 |
| AC007879.7 | TUBA1A | 0.32 | 1.01E-13 |
| AC007879.7 | MALT1 | 0.32 | 1.02E-13 |
| AC007879.7 | UNC45B | 0.32 | 1.02E-13 |
| AC007879.7 | TMEM204 | 0.32 | 1.04E-13 |
| AC007879.7 | C5orf42 | 0.32 | 1.04E-13 |
| AC007879.7 | DNAJB7 | 0.32 | 1.05E-13 |
| AC007879.7 | ZFP82 | 0.32 | 1.05E-13 |
| AC007879.7 | RRAGD | 0.32 | 1.06E-13 |
| AC007879.7 | STX6 | 0.32 | 1.08E-13 |
| AC007879.7 | LIPI | 0.32 | 1.11E-13 |
| AC007879.7 | MFAP3 | 0.32 | 1.11E-13 |
| AC007879.7 | TMPRSS11B | 0.32 | 1.12E-13 |
| AC007879.7 | PPP1R12A | 0.32 | 1.12E-13 |
| AC007879.7 | PTCHD3 | 0.32 | 1.14E-13 |
| AC007879.7 | ADGRD1 | 0.32 | 1.15E-13 |
| AC007879.7 | PDLIM4 | 0.321 | 8.19E-14 |
| AC007879.7 | DIRAS3 | 0.321 | 8.22E-14 |
| AC007879.7 | ANKRD36 | 0.321 | 8.22E-14 |
| AC007879.7 | ARG1 | 0.321 | 8.30E-14 |
| AC007879.7 | OSBPL3 | 0.321 | 8.34E-14 |
| AC007879.7 | TAS2R31 | 0.321 | 8.47E-14 |
| AC007879.7 | KCNAB3 | 0.321 | 8.52E-14 |
| AC007879.7 | CSF1 | 0.321 | 8.59E-14 |
| AC007879.7 | NRG3 | 0.321 | 8.61E-14 |
| AC007879.7 | PIK3C3 | 0.321 | 8.69E-14 |
| AC007879.7 | RPS6KC1 | 0.321 | 8.75E-14 |
| AC007879.7 | GABPB1 | 0.321 | 8.78E-14 |
| AC007879.7 | TAAR8 | 0.321 | 8.89E-14 |
| AC007879.7 | ZNF625 | 0.321 | 8.96E-14 |
| AC007879.7 | RAPH1 | 0.321 | 9.03E-14 |
| AC007879.7 | RNF212B | 0.321 | 9.08E-14 |
| AC007879.7 | KCND1 | 0.321 | 9.23E-14 |
| AC007879.7 | SHANK3 | 0.321 | 9.29E-14 |
| AC007879.7 | VGLL4 | 0.321 | 9.35E-14 |
| AC007879.7 | NTRK1 | 0.321 | 9.42E-14 |
| AC007879.7 | ATP13A5 | 0.321 | 9.58E-14 |
| AC007879.7 | POU5F2 | 0.321 | 9.61E-14 |
| AC007879.7 | ANKRD55 | 0.322 | 6.68E-14 |
| AC007879.7 | ANKRD66 | 0.322 | 6.79E-14 |
| AC007879.7 | C5orf49 | 0.322 | 6.89E-14 |
| AC007879.7 | KRT6A | 0.322 | 6.93E-14 |
| AC007879.7 | MAGEH1 | 0.322 | 6.96E-14 |
| AC007879.7 | FOXO1 | 0.322 | 7.00E-14 |
| AC007879.7 | TAAR9 | 0.322 | 7.01E-14 |
| AC007879.7 | CGB7 | 0.322 | 7.04E-14 |
| AC007879.7 | ZNF98 | 0.322 | 7.08E-14 |
| AC007879.7 | ZNF286B | 0.322 | 7.10E-14 |
| AC007879.7 | TEX13A | 0.322 | 7.25E-14 |
| AC007879.7 | LEF1 | 0.322 | 7.25E-14 |
| AC007879.7 | PLEKHM3 | 0.322 | 7.27E-14 |
| AC007879.7 | CCDC168 | 0.322 | 7.41E-14 |
| AC007879.7 | UBXN7 | 0.322 | 7.42E-14 |
| AC007879.7 | RBM43 | 0.322 | 7.56E-14 |
| AC007879.7 | CADM1 | 0.322 | 7.61E-14 |
| AC007879.7 | DDX47 | 0.322 | 7.76E-14 |
| AC007879.7 | TRIM38 | 0.322 | 7.76E-14 |
| AC007879.7 | PLEKHG1 | 0.322 | 7.77E-14 |
| AC007879.7 | TVP23A | 0.322 | 7.92E-14 |
| AC007879.7 | ARHGEF33 | 0.322 | 7.94E-14 |
| AC007879.7 | GRASP | 0.322 | 7.94E-14 |
| AC007879.7 | RBM44 | 0.322 | 7.95E-14 |
| AC007879.7 | HCFC2 | 0.323 | 5.75E-14 |
| AC007879.7 | RUFY2 | 0.323 | 5.78E-14 |
| AC007879.7 | PDLIM5 | 0.323 | 5.85E-14 |
| AC007879.7 | CTD-2116N17.1 | 0.323 | 5.88E-14 |
| AC007879.7 | CERKL | 0.323 | 5.91E-14 |
| AC007879.7 | LHX8 | 0.323 | 5.94E-14 |
| AC007879.7 | OR52J3 | 0.323 | 5.98E-14 |
| AC007879.7 | PHC3 | 0.323 | 6.05E-14 |
| AC007879.7 | SBK3 | 0.323 | 6.06E-14 |
| AC007879.7 | KLHDC8A | 0.323 | 6.08E-14 |
| AC007879.7 | NME9 | 0.323 | 6.12E-14 |
| AC007879.7 | PCDHB2 | 0.323 | 6.15E-14 |
| AC007879.7 | GRM7 | 0.323 | 6.27E-14 |
| AC007879.7 | ANKRD36C | 0.323 | 6.39E-14 |
| AC007879.7 | C9orf153 | 0.323 | 6.41E-14 |
| AC007879.7 | ERVW-1 | 0.323 | 6.42E-14 |
| AC007879.7 | HS3ST2 | 0.323 | 6.42E-14 |
| AC007879.7 | GPATCH2 | 0.323 | 6.43E-14 |
| AC007879.7 | DPPA3 | 0.323 | 6.48E-14 |
| AC007879.7 | FSD2 | 0.323 | 6.49E-14 |
| AC007879.7 | SLC4A7 | 0.323 | 6.53E-14 |
| AC007879.7 | F5 | 0.323 | 6.54E-14 |
| AC007879.7 | HOXC5 | 0.324 | 4.61E-14 |
| AC007879.7 | PLAUR | 0.324 | 4.70E-14 |
| AC007879.7 | TAS2R8 | 0.324 | 4.86E-14 |
| AC007879.7 | S1PR5 | 0.324 | 4.87E-14 |
| AC007879.7 | CCDC144A | 0.324 | 4.88E-14 |
| AC007879.7 | AC010642.1 | 0.324 | 4.90E-14 |
| AC007879.7 | MR1 | 0.324 | 4.93E-14 |
| AC007879.7 | FAM219A | 0.324 | 4.95E-14 |
| AC007879.7 | PSTPIP1 | 0.324 | 4.97E-14 |
| AC007879.7 | RNF144A | 0.324 | 4.99E-14 |
| AC007879.7 | ANKRD12 | 0.324 | 5.00E-14 |
| AC007879.7 | C9orf3 | 0.324 | 5.05E-14 |
| AC007879.7 | PTPN22 | 0.324 | 5.08E-14 |
| AC007879.7 | SENP7 | 0.324 | 5.09E-14 |
| AC007879.7 | HSPA12B | 0.324 | 5.13E-14 |
| AC007879.7 | SLC5A4 | 0.324 | 5.16E-14 |
| AC007879.7 | ACSM6 | 0.324 | 5.21E-14 |
| AC007879.7 | AC124312.1 | 0.324 | 5.27E-14 |
| AC007879.7 | CBWD3 | 0.324 | 5.28E-14 |
| AC007879.7 | CHST10 | 0.324 | 5.28E-14 |
| AC007879.7 | SEMA6B | 0.324 | 5.28E-14 |
| AC007879.7 | TAGLN | 0.324 | 5.28E-14 |
| AC007879.7 | CCDC82 | 0.324 | 5.34E-14 |
| AC007879.7 | MARK3 | 0.324 | 5.35E-14 |
| AC007879.7 | ANKRD30BL | 0.324 | 5.37E-14 |
| AC007879.7 | TRAPPC6B | 0.324 | 5.38E-14 |
| AC007879.7 | VMO1 | 0.324 | 5.44E-14 |
| AC007879.7 | KLF9 | 0.324 | 5.44E-14 |
| AC007879.7 | ZKSCAN8 | 0.325 | 3.77E-14 |
| AC007879.7 | TCAF2 | 0.325 | 3.78E-14 |
| AC007879.7 | RP11-437B10.1 | 0.325 | 3.79E-14 |
| AC007879.7 | CBWD5 | 0.325 | 3.82E-14 |
| AC007879.7 | OSTN | 0.325 | 3.83E-14 |
| AC007879.7 | C21orf91 | 0.325 | 3.84E-14 |
| AC007879.7 | RP11-613M10.9 | 0.325 | 3.85E-14 |
| AC007879.7 | DMXL2 | 0.325 | 3.85E-14 |
| AC007879.7 | SLFN14 | 0.325 | 3.88E-14 |
| AC007879.7 | RNF113B | 0.325 | 3.89E-14 |
| AC007879.7 | HCRTR1 | 0.325 | 3.90E-14 |
| AC007879.7 | FBLN2 | 0.325 | 3.97E-14 |
| AC007879.7 | LGI2 | 0.325 | 3.99E-14 |
| AC007879.7 | TMEM169 | 0.325 | 4.04E-14 |
| AC007879.7 | TMEM30A | 0.325 | 4.17E-14 |
| AC007879.7 | RD3L | 0.325 | 4.23E-14 |
| AC007879.7 | RP11-574K11.31 | 0.325 | 4.24E-14 |
| AC007879.7 | ZBTB8B | 0.325 | 4.24E-14 |
| AC007879.7 | CCP110 | 0.325 | 4.29E-14 |
| AC007879.7 | TCF15 | 0.325 | 4.33E-14 |
| AC007879.7 | BMP1 | 0.325 | 4.38E-14 |
| AC007879.7 | PHIP | 0.325 | 4.40E-14 |
| AC007879.7 | RP11-136C24.3 | 0.325 | 4.42E-14 |
| AC007879.7 | CNPY4 | 0.325 | 4.46E-14 |
| AC007879.7 | OXTR | 0.325 | 4.46E-14 |
| AC007879.7 | FAM129A | 0.325 | 4.49E-14 |
| AC007879.7 | USP29 | 0.325 | 4.50E-14 |
| AC007879.7 | SLC16A10 | 0.325 | 4.51E-14 |
| AC007879.7 | KLHL28 | 0.325 | 4.55E-14 |
| AC007879.7 | FOLH1 | 0.326 | 3.15E-14 |
| AC007879.7 | TMEM244 | 0.326 | 3.16E-14 |
| AC007879.7 | ZC3H10 | 0.326 | 3.20E-14 |
| AC007879.7 | CCDC181 | 0.326 | 3.23E-14 |
| AC007879.7 | SPINK13 | 0.326 | 3.24E-14 |
| AC007879.7 | PKD2L2 | 0.326 | 3.25E-14 |
| AC007879.7 | AC016549.1 | 0.326 | 3.29E-14 |
| AC007879.7 | LIPK | 0.326 | 3.33E-14 |
| AC007879.7 | C4orf17 | 0.326 | 3.37E-14 |
| AC007879.7 | RPS6KA2 | 0.326 | 3.37E-14 |
| AC007879.7 | ZRSR1 | 0.326 | 3.42E-14 |
| AC007879.7 | CSF1R | 0.326 | 3.50E-14 |
| AC007879.7 | CPEB4 | 0.326 | 3.50E-14 |
| AC007879.7 | SETD7 | 0.326 | 3.53E-14 |
| AC007879.7 | ZNF221 | 0.326 | 3.55E-14 |
| AC007879.7 | C16orf45 | 0.326 | 3.59E-14 |
| AC007879.7 | VTN | 0.326 | 3.65E-14 |
| AC007879.7 | ELF2 | 0.326 | 3.65E-14 |
| AC007879.7 | SARNP | 0.326 | 3.66E-14 |
| AC007879.7 | SPINK6 | 0.326 | 3.71E-14 |
| AC007879.7 | PATE2 | 0.326 | 3.75E-14 |
| AC007879.7 | SOCS4 | 0.326 | 3.77E-14 |
| AC007879.7 | PDC | 0.327 | 2.58E-14 |
| AC007879.7 | FOXC1 | 0.327 | 2.64E-14 |
| AC007879.7 | SPINK7 | 0.327 | 2.65E-14 |
| AC007879.7 | FLG2 | 0.327 | 2.69E-14 |
| AC007879.7 | RGS22 | 0.327 | 2.69E-14 |
| AC007879.7 | KIR3DX1 | 0.327 | 2.71E-14 |
| AC007879.7 | ELK4 | 0.327 | 2.73E-14 |
| AC007879.7 | NPR2 | 0.327 | 2.80E-14 |
| AC007879.7 | SYT9 | 0.327 | 2.83E-14 |
| AC007879.7 | KRTAP24-1 | 0.327 | 2.84E-14 |
| AC007879.7 | OMG | 0.327 | 2.88E-14 |
| AC007879.7 | F2RL3 | 0.327 | 2.92E-14 |
| AC007879.7 | MEMO1 | 0.327 | 3.03E-14 |
| AC007879.7 | PLK2 | 0.327 | 3.04E-14 |
| AC007879.7 | C1orf216 | 0.327 | 3.05E-14 |
| AC007879.7 | ZNF660 | 0.327 | 3.08E-14 |
| AC007879.7 | RAX | 0.327 | 3.10E-14 |
| AC007879.7 | SERPINB10 | 0.327 | 3.10E-14 |
| AC007879.7 | FBXO32 | 0.327 | 3.10E-14 |
| AC007879.7 | METTL11B | 0.328 | 2.14E-14 |
| AC007879.7 | SMARCD3 | 0.328 | 2.17E-14 |
| AC007879.7 | PARVG | 0.328 | 2.18E-14 |
| AC007879.7 | SYT15 | 0.328 | 2.25E-14 |
| AC007879.7 | C8A | 0.328 | 2.25E-14 |
| AC007879.7 | LRRC63 | 0.328 | 2.27E-14 |
| AC007879.7 | ZNF280D | 0.328 | 2.28E-14 |
| AC007879.7 | KLC1 | 0.328 | 2.28E-14 |
| AC007879.7 | ARHGAP30 | 0.328 | 2.35E-14 |
| AC007879.7 | CFAP57 | 0.328 | 2.37E-14 |
| AC007879.7 | TIGD7 | 0.328 | 2.38E-14 |
| AC007879.7 | TXNDC8 | 0.328 | 2.40E-14 |
| AC007879.7 | TAS2R13 | 0.328 | 2.40E-14 |
| AC007879.7 | NAMPT | 0.328 | 2.41E-14 |
| AC007879.7 | TRAF1 | 0.328 | 2.45E-14 |
| AC007879.7 | SPDYE5 | 0.328 | 2.47E-14 |
| AC007879.7 | AP4E1 | 0.328 | 2.51E-14 |
| AC007879.7 | RGL1 | 0.328 | 2.52E-14 |
| AC007879.7 | BTK | 0.328 | 2.56E-14 |
| AC007879.7 | F13A1 | 0.328 | 2.57E-14 |
| AC007879.7 | CFAP43 | 0.329 | 1.78E-14 |
| AC007879.7 | OCLM | 0.329 | 1.79E-14 |
| AC007879.7 | TEAD1 | 0.329 | 1.80E-14 |
| AC007879.7 | ZNF80 | 0.329 | 1.81E-14 |
| AC007879.7 | ZSWIM2 | 0.329 | 1.82E-14 |
| AC007879.7 | C10orf128 | 0.329 | 1.83E-14 |
| AC007879.7 | USP49 | 0.329 | 1.85E-14 |
| AC007879.7 | UGGT2 | 0.329 | 1.86E-14 |
| AC007879.7 | ANO6 | 0.329 | 1.87E-14 |
| AC007879.7 | AC013286.1 | 0.329 | 1.88E-14 |
| AC007879.7 | AL365202.1 | 0.329 | 1.96E-14 |
| AC007879.7 | UBE2QL1 | 0.329 | 1.99E-14 |
| AC007879.7 | SETX | 0.329 | 1.99E-14 |
| AC007879.7 | GRK4 | 0.329 | 2.02E-14 |
| AC007879.7 | FAM114A2 | 0.329 | 2.02E-14 |
| AC007879.7 | USP51 | 0.329 | 2.02E-14 |
| AC007879.7 | TSPYL4 | 0.329 | 2.07E-14 |
| AC007879.7 | TTN | 0.329 | 2.07E-14 |
| AC007879.7 | DNAH17 | 0.33 | 1.45E-14 |
| AC007879.7 | SDCCAG8 | 0.33 | 1.46E-14 |
| AC007879.7 | ZNF671 | 0.33 | 1.47E-14 |
| AC007879.7 | PAM | 0.33 | 1.48E-14 |
| AC007879.7 | BNIP3L | 0.33 | 1.50E-14 |
| AC007879.7 | GHRL | 0.33 | 1.51E-14 |
| AC007879.7 | ACSL4 | 0.33 | 1.54E-14 |
| AC007879.7 | PAK3 | 0.33 | 1.55E-14 |
| AC007879.7 | ARRDC3 | 0.33 | 1.57E-14 |
| AC007879.7 | SERPINI2 | 0.33 | 1.59E-14 |
| AC007879.7 | SLC7A2 | 0.33 | 1.60E-14 |
| AC007879.7 | IKZF4 | 0.33 | 1.61E-14 |
| AC007879.7 | GTF2A1L | 0.33 | 1.64E-14 |
| AC007879.7 | S1PR2 | 0.33 | 1.64E-14 |
| AC007879.7 | AC016577.1 | 0.33 | 1.67E-14 |
| AC007879.7 | PDLIM7 | 0.33 | 1.67E-14 |
| AC007879.7 | BVES | 0.33 | 1.70E-14 |
| AC007879.7 | UHRF2 | 0.33 | 1.71E-14 |
| AC007879.7 | SPATA16 | 0.33 | 1.72E-14 |
| AC007879.7 | KLHDC1 | 0.33 | 1.73E-14 |
| AC007879.7 | ATP2B4 | 0.33 | 1.74E-14 |
| AC007879.7 | RP11-872D17.8 | 0.33 | 1.75E-14 |
| AC007879.7 | HOXC4 | 0.331 | 1.21E-14 |
| AC007879.7 | OR2C3 | 0.331 | 1.23E-14 |
| AC007879.7 | ESAM | 0.331 | 1.23E-14 |
| AC007879.7 | ATXN3 | 0.331 | 1.25E-14 |
| AC007879.7 | A4GNT | 0.331 | 1.27E-14 |
| AC007879.7 | FSHR | 0.331 | 1.28E-14 |
| AC007879.7 | IL11 | 0.331 | 1.30E-14 |
| AC007879.7 | U51561.1 | 0.331 | 1.30E-14 |
| AC007879.7 | OR7A5 | 0.331 | 1.32E-14 |
| AC007879.7 | GJB6 | 0.331 | 1.33E-14 |
| AC007879.7 | AHNAK2 | 0.331 | 1.35E-14 |
| AC007879.7 | SCIMP | 0.331 | 1.36E-14 |
| AC007879.7 | TMEM200C | 0.331 | 1.36E-14 |
| AC007879.7 | TMEM110-MUSTN1 | 0.331 | 1.38E-14 |
| AC007879.7 | AL596220.1 | 0.331 | 1.41E-14 |
| AC007879.7 | OR2B11 | 0.331 | 1.42E-14 |
| AC007879.7 | GOLGA8K | 0.331 | 1.43E-14 |
| AC007879.7 | PAPOLG | 0.332 | 9.86E-15 |
| AC007879.7 | WASF1 | 0.332 | 9.88E-15 |
| AC007879.7 | MKLN1 | 0.332 | 1.03E-14 |
| AC007879.7 | MAP3K7 | 0.332 | 1.04E-14 |
| AC007879.7 | OR4L1 | 0.332 | 1.10E-14 |
| AC007879.7 | PCDH15 | 0.332 | 1.10E-14 |
| AC007879.7 | SHCBP1L | 0.332 | 1.10E-14 |
| AC007879.7 | AIDA | 0.332 | 1.10E-14 |
| AC007879.7 | IL24 | 0.332 | 1.11E-14 |
| AC007879.7 | SLC9B1 | 0.332 | 1.11E-14 |
| AC007879.7 | GNG2 | 0.332 | 1.12E-14 |
| AC007879.7 | TRIM61 | 0.332 | 1.13E-14 |
| AC007879.7 | NME7 | 0.332 | 1.14E-14 |
| AC007879.7 | GULP1 | 0.332 | 1.15E-14 |
| AC007879.7 | PXDC1 | 0.332 | 1.16E-14 |
| AC007879.7 | ZNF560 | 0.332 | 1.18E-14 |
| AC007879.7 | HIPK3 | 0.333 | 8.13E-15 |
| AC007879.7 | PSG4 | 0.333 | 8.20E-15 |
| AC007879.7 | TANC1 | 0.333 | 8.27E-15 |
| AC007879.7 | IL21R | 0.333 | 8.28E-15 |
| AC007879.7 | SYNE3 | 0.333 | 8.30E-15 |
| AC007879.7 | LRCH1 | 0.333 | 8.43E-15 |
| AC007879.7 | PP2D1 | 0.333 | 8.47E-15 |
| AC007879.7 | BLID | 0.333 | 8.50E-15 |
| AC007879.7 | COL4A4 | 0.333 | 8.54E-15 |
| AC007879.7 | UCHL1 | 0.333 | 8.55E-15 |
| AC007879.7 | OR56B1 | 0.333 | 8.56E-15 |
| AC007879.7 | ZNF236 | 0.333 | 8.59E-15 |
| AC007879.7 | LRRC25 | 0.333 | 8.69E-15 |
| AC007879.7 | CSF2RA | 0.333 | 8.72E-15 |
| AC007879.7 | ITPRIPL2 | 0.333 | 8.84E-15 |
| AC007879.7 | KCNH1 | 0.333 | 8.85E-15 |
| AC007879.7 | ARIH1 | 0.333 | 8.90E-15 |
| AC007879.7 | ASB14 | 0.333 | 8.93E-15 |
| AC007879.7 | TBX19 | 0.333 | 9.16E-15 |
| AC007879.7 | TREML1 | 0.333 | 9.30E-15 |
| AC007879.7 | CCSAP | 0.333 | 9.37E-15 |
| AC007879.7 | ANKRD1 | 0.333 | 9.70E-15 |
| AC007879.7 | AGBL3 | 0.333 | 9.78E-15 |
| AC007879.7 | SNAPC1 | 0.334 | 7.01E-15 |
| AC007879.7 | RASSF9 | 0.334 | 7.02E-15 |
| AC007879.7 | SLC12A6 | 0.334 | 7.16E-15 |
| AC007879.7 | PRKD3 | 0.334 | 7.26E-15 |
| AC007879.7 | HPS5 | 0.334 | 7.26E-15 |
| AC007879.7 | FBLN5 | 0.334 | 7.28E-15 |
| AC007879.7 | TTC28 | 0.334 | 7.29E-15 |
| AC007879.7 | TXLNB | 0.334 | 7.35E-15 |
| AC007879.7 | USP15 | 0.334 | 7.52E-15 |
| AC007879.7 | GLRA3 | 0.334 | 7.72E-15 |
| AC007879.7 | SH3RF3 | 0.334 | 7.79E-15 |
| AC007879.7 | FRMD7 | 0.334 | 8.03E-15 |
| AC007879.7 | KCNRG | 0.334 | 8.04E-15 |
| AC007879.7 | ZNF286A | 0.335 | 5.54E-15 |
| AC007879.7 | PBOV1 | 0.335 | 5.54E-15 |
| AC007879.7 | MYOF | 0.335 | 5.58E-15 |
| AC007879.7 | TIAM1 | 0.335 | 5.61E-15 |
| AC007879.7 | PTPRE | 0.335 | 5.62E-15 |
| AC007879.7 | ALOX15B | 0.335 | 5.67E-15 |
| AC007879.7 | ARHGAP25 | 0.335 | 5.73E-15 |
| AC007879.7 | AOC3 | 0.335 | 5.76E-15 |
| AC007879.7 | IQCJ | 0.335 | 5.79E-15 |
| AC007879.7 | USP6 | 0.335 | 5.86E-15 |
| AC007879.7 | NFAT5 | 0.335 | 6.03E-15 |
| AC007879.7 | CYBRD1 | 0.335 | 6.08E-15 |
| AC007879.7 | PRL | 0.335 | 6.09E-15 |
| AC007879.7 | ROCK1 | 0.335 | 6.10E-15 |
| AC007879.7 | GNS | 0.335 | 6.13E-15 |
| AC007879.7 | HRNR | 0.335 | 6.19E-15 |
| AC007879.7 | CD248 | 0.335 | 6.20E-15 |
| AC007879.7 | CD300A | 0.335 | 6.27E-15 |
| AC007879.7 | RGAG4 | 0.335 | 6.29E-15 |
| AC007879.7 | ATP8B3 | 0.335 | 6.31E-15 |
| AC007879.7 | OTOA | 0.335 | 6.34E-15 |
| AC007879.7 | SNRK | 0.335 | 6.43E-15 |
| AC007879.7 | IL10RA | 0.335 | 6.54E-15 |
| AC007879.7 | PLCL1 | 0.335 | 6.66E-15 |
| AC007879.7 | APOC1 | 0.336 | 4.52E-15 |
| AC007879.7 | SPATA9 | 0.336 | 4.60E-15 |
| AC007879.7 | VCL | 0.336 | 4.63E-15 |
| AC007879.7 | PRH1 | 0.336 | 4.65E-15 |
| AC007879.7 | PLEKHA3 | 0.336 | 4.66E-15 |
| AC007879.7 | CLEC14A | 0.336 | 4.78E-15 |
| AC007879.7 | FAM153B | 0.336 | 4.85E-15 |
| AC007879.7 | ARID4B | 0.336 | 4.85E-15 |
| AC007879.7 | PPT1 | 0.336 | 4.95E-15 |
| AC007879.7 | FAM19A1 | 0.336 | 5.01E-15 |
| AC007879.7 | OAZ2 | 0.336 | 5.11E-15 |
| AC007879.7 | C12orf42 | 0.336 | 5.17E-15 |
| AC007879.7 | SLC35F4 | 0.336 | 5.21E-15 |
| AC007879.7 | XAF1 | 0.336 | 5.21E-15 |
| AC007879.7 | VPS13B | 0.336 | 5.22E-15 |
| AC007879.7 | CEP128 | 0.336 | 5.24E-15 |
| AC007879.7 | MRAP | 0.336 | 5.30E-15 |
| AC007879.7 | PRR4 | 0.336 | 5.32E-15 |
| AC007879.7 | PTPN5 | 0.336 | 5.42E-15 |
| AC007879.7 | ASZ1 | 0.336 | 5.47E-15 |
| AC007879.7 | DGKH | 0.337 | 3.71E-15 |
| AC007879.7 | CTLA4 | 0.337 | 3.83E-15 |
| AC007879.7 | SPON2 | 0.337 | 3.85E-15 |
| AC007879.7 | ETV5 | 0.337 | 3.92E-15 |
| AC007879.7 | TLL1 | 0.337 | 3.92E-15 |
| AC007879.7 | MYBPH | 0.337 | 3.94E-15 |
| AC007879.7 | FPGT-TNNI3K | 0.337 | 3.97E-15 |
| AC007879.7 | C3 | 0.337 | 3.97E-15 |
| AC007879.7 | SYCE1 | 0.337 | 4.15E-15 |
| AC007879.7 | TPBGL | 0.337 | 4.28E-15 |
| AC007879.7 | N4BP2 | 0.337 | 4.39E-15 |
| AC007879.7 | IGF1 | 0.337 | 4.49E-15 |
| AC007879.7 | ZBTB20 | 0.338 | 3.03E-15 |
| AC007879.7 | MEIOC | 0.338 | 3.04E-15 |
| AC007879.7 | KCNJ5 | 0.338 | 3.05E-15 |
| AC007879.7 | RAB2B | 0.338 | 3.09E-15 |
| AC007879.7 | A4GALT | 0.338 | 3.20E-15 |
| AC007879.7 | CYP2A6 | 0.338 | 3.21E-15 |
| AC007879.7 | ARID5B | 0.338 | 3.22E-15 |
| AC007879.7 | KIF17 | 0.338 | 3.28E-15 |
| AC007879.7 | LRRTM2 | 0.338 | 3.32E-15 |
| AC007879.7 | ZNF720 | 0.338 | 3.34E-15 |
| AC007879.7 | CCDC173 | 0.338 | 3.34E-15 |
| AC007879.7 | LRRC55 | 0.338 | 3.36E-15 |
| AC007879.7 | SEL1L2 | 0.338 | 3.39E-15 |
| AC007879.7 | PODXL | 0.338 | 3.39E-15 |
| AC007879.7 | MPRIP | 0.338 | 3.41E-15 |
| AC007879.7 | CLRN1 | 0.338 | 3.41E-15 |
| AC007879.7 | NKAIN4 | 0.338 | 3.41E-15 |
| AC007879.7 | AC092835.2 | 0.338 | 3.46E-15 |
| AC007879.7 | PLET1 | 0.338 | 3.47E-15 |
| AC007879.7 | IMPG1 | 0.338 | 3.59E-15 |
| AC007879.7 | SERPINH1 | 0.338 | 3.60E-15 |
| AC007879.7 | BHLHE22 | 0.338 | 3.69E-15 |
| AC007879.7 | WDR41 | 0.339 | 2.50E-15 |
| AC007879.7 | CLEC4C | 0.339 | 2.51E-15 |
| AC007879.7 | DCC | 0.339 | 2.56E-15 |
| AC007879.7 | SEC14L1 | 0.339 | 2.56E-15 |
| AC007879.7 | POLK | 0.339 | 2.59E-15 |
| AC007879.7 | TSPAN16 | 0.339 | 2.61E-15 |
| AC007879.7 | CASP4 | 0.339 | 2.64E-15 |
| AC007879.7 | STAT4 | 0.339 | 2.66E-15 |
| AC007879.7 | SLC22A24 | 0.339 | 2.78E-15 |
| AC007879.7 | RP11-385D13.1 | 0.339 | 2.78E-15 |
| AC007879.7 | VASH2 | 0.339 | 2.79E-15 |
| AC007879.7 | MYH13 | 0.339 | 2.80E-15 |
| AC007879.7 | ARHGAP23 | 0.339 | 2.80E-15 |
| AC007879.7 | FOXS1 | 0.339 | 2.88E-15 |
| AC007879.7 | NUMBL | 0.339 | 2.91E-15 |
| AC007879.7 | CSMD1 | 0.339 | 2.96E-15 |
| AC007879.7 | TRAF3IP3 | 0.339 | 3.02E-15 |
| AC007879.7 | MMRN2 | 0.339 | 3.02E-15 |
| AC007879.7 | BATF3 | 0.34 | 2.08E-15 |
| AC007879.7 | TRPV2 | 0.34 | 2.16E-15 |
| AC007879.7 | HSD17B14 | 0.34 | 2.17E-15 |
| AC007879.7 | GGCX | 0.34 | 2.19E-15 |
| AC007879.7 | ASB15 | 0.34 | 2.19E-15 |
| AC007879.7 | TCEAL3 | 0.34 | 2.24E-15 |
| AC007879.7 | C4orf45 | 0.34 | 2.26E-15 |
| AC007879.7 | ERICH6 | 0.34 | 2.27E-15 |
| AC007879.7 | AHR | 0.34 | 2.28E-15 |
| AC007879.7 | IL12RB2 | 0.34 | 2.30E-15 |
| AC007879.7 | ZNF624 | 0.34 | 2.32E-15 |
| AC007879.7 | POLI | 0.34 | 2.41E-15 |
| AC007879.7 | SULT6B1 | 0.34 | 2.42E-15 |
| AC007879.7 | KRTAP5-7 | 0.34 | 2.44E-15 |
| AC007879.7 | ERVFRD-1 | 0.34 | 2.46E-15 |
| AC007879.7 | ADAM20 | 0.34 | 2.48E-15 |
| AC007879.7 | LHFPL2 | 0.341 | 1.68E-15 |
| AC007879.7 | REV3L | 0.341 | 1.68E-15 |
| AC007879.7 | TMEM108 | 0.341 | 1.72E-15 |
| AC007879.7 | TSKS | 0.341 | 1.72E-15 |
| AC007879.7 | COL14A1 | 0.341 | 1.74E-15 |
| AC007879.7 | RP11-849F2.7 | 0.341 | 1.74E-15 |
| AC007879.7 | IDS | 0.341 | 1.74E-15 |
| AC007879.7 | SWAP70 | 0.341 | 1.75E-15 |
| AC007879.7 | CD69 | 0.341 | 1.78E-15 |
| AC007879.7 | RELT | 0.341 | 1.80E-15 |
| AC007879.7 | SAMD14 | 0.341 | 1.81E-15 |
| AC007879.7 | A1BG | 0.341 | 1.85E-15 |
| AC007879.7 | SLC24A3 | 0.341 | 1.92E-15 |
| AC007879.7 | CD300C | 0.341 | 1.93E-15 |
| AC007879.7 | NLGN4Y | 0.341 | 1.94E-15 |
| AC007879.7 | CNTN1 | 0.341 | 1.94E-15 |
| AC007879.7 | KIAA1328 | 0.341 | 1.99E-15 |
| AC007879.7 | SH2B3 | 0.341 | 1.99E-15 |
| AC007879.7 | RGCC | 0.341 | 2.02E-15 |
| AC007879.7 | ARHGAP10 | 0.342 | 1.41E-15 |
| AC007879.7 | FBN2 | 0.342 | 1.42E-15 |
| AC007879.7 | C12orf50 | 0.342 | 1.42E-15 |
| AC007879.7 | ATP1A4 | 0.342 | 1.43E-15 |
| AC007879.7 | HAS2 | 0.342 | 1.44E-15 |
| AC007879.7 | ISLR | 0.342 | 1.51E-15 |
| AC007879.7 | MYCBP2 | 0.342 | 1.52E-15 |
| AC007879.7 | ITGB2 | 0.342 | 1.52E-15 |
| AC007879.7 | ZNF519 | 0.342 | 1.54E-15 |
| AC007879.7 | TROVE2 | 0.342 | 1.57E-15 |
| AC007879.7 | C7orf62 | 0.342 | 1.57E-15 |
| AC007879.7 | PDZD2 | 0.342 | 1.59E-15 |
| AC007879.7 | DSTYK | 0.342 | 1.60E-15 |
| AC007879.7 | PCDHA9 | 0.342 | 1.62E-15 |
| AC007879.7 | WDR47 | 0.342 | 1.62E-15 |
| AC007879.7 | NKAPL | 0.342 | 1.65E-15 |
| AC007879.7 | HAL | 0.343 | 1.11E-15 |
| AC007879.7 | LDOC1 | 0.343 | 1.11E-15 |
| AC007879.7 | ZNF568 | 0.343 | 1.12E-15 |
| AC007879.7 | PINLYP | 0.343 | 1.12E-15 |
| AC007879.7 | FKBP15 | 0.343 | 1.13E-15 |
| AC007879.7 | TRAM2 | 0.343 | 1.14E-15 |
| AC007879.7 | BTG4 | 0.343 | 1.15E-15 |
| AC007879.7 | ZNF676 | 0.343 | 1.17E-15 |
| AC007879.7 | GUCY2F | 0.343 | 1.18E-15 |
| AC007879.7 | DNAJC18 | 0.343 | 1.18E-15 |
| AC007879.7 | VEPH1 | 0.343 | 1.20E-15 |
| AC007879.7 | RECQL | 0.343 | 1.21E-15 |
| AC007879.7 | MEIS2 | 0.343 | 1.24E-15 |
| AC007879.7 | TCEB3B | 0.343 | 1.24E-15 |
| AC007879.7 | TRAPPC3L | 0.343 | 1.25E-15 |
| AC007879.7 | ZNF728 | 0.343 | 1.28E-15 |
| AC007879.7 | ELMO1 | 0.343 | 1.30E-15 |
| AC007879.7 | STOM | 0.343 | 1.32E-15 |
| AC007879.7 | GDPD4 | 0.343 | 1.34E-15 |
| AC007879.7 | ETV3L | 0.343 | 1.34E-15 |
| AC007879.7 | REC114 | 0.343 | 1.35E-15 |
| AC007879.7 | PARVA | 0.343 | 1.35E-15 |
| AC007879.7 | CXorf21 | 0.344 | 9.11E-16 |
| AC007879.7 | C9orf84 | 0.344 | 9.11E-16 |
| AC007879.7 | OPRL1 | 0.344 | 9.12E-16 |
| AC007879.7 | PCDHGA2 | 0.344 | 9.14E-16 |
| AC007879.7 | PTPRQ | 0.344 | 9.29E-16 |
| AC007879.7 | MGP | 0.344 | 9.35E-16 |
| AC007879.7 | C17orf47 | 0.344 | 9.38E-16 |
| AC007879.7 | SMURF2 | 0.344 | 9.55E-16 |
| AC007879.7 | CBLB | 0.344 | 9.65E-16 |
| AC007879.7 | TAGAP | 0.344 | 9.73E-16 |
| AC007879.7 | SPARCL1 | 0.344 | 1.00E-15 |
| AC007879.7 | INPP5F | 0.344 | 1.01E-15 |
| AC007879.7 | DLG2 | 0.344 | 1.02E-15 |
| AC007879.7 | C20orf141 | 0.344 | 1.06E-15 |
| AC007879.7 | SHISA4 | 0.344 | 1.06E-15 |
| AC007879.7 | HSD17B6 | 0.344 | 1.09E-15 |
| AC007879.7 | LETM2 | 0.344 | 1.10E-15 |
| AC007879.7 | GLIS1 | 0.344 | 1.11E-15 |
| AC007879.7 | ARHGEF25 | 0.345 | 7.51E-16 |
| AC007879.7 | OR11A1 | 0.345 | 7.74E-16 |
| AC007879.7 | ZMAT3 | 0.345 | 7.83E-16 |
| AC007879.7 | CELA2B | 0.345 | 7.84E-16 |
| AC007879.7 | SMIM10 | 0.345 | 8.01E-16 |
| AC007879.7 | FGD5 | 0.345 | 8.25E-16 |
| AC007879.7 | PARP15 | 0.345 | 8.30E-16 |
| AC007879.7 | GRIK1 | 0.345 | 8.48E-16 |
| AC007879.7 | TSPYL6 | 0.345 | 8.53E-16 |
| AC007879.7 | CEP19 | 0.345 | 8.53E-16 |
| AC007879.7 | LGALS1 | 0.345 | 8.56E-16 |
| AC007879.7 | JAK3 | 0.345 | 8.59E-16 |
| AC007879.7 | INTS6 | 0.345 | 8.64E-16 |
| AC007879.7 | SLC12A1 | 0.345 | 8.76E-16 |
| AC007879.7 | PURA | 0.345 | 8.79E-16 |
| AC007879.7 | DUSP10 | 0.345 | 9.01E-16 |
| AC007879.7 | LYG2 | 0.345 | 9.05E-16 |
| AC007879.7 | CYTIP | 0.345 | 9.08E-16 |
| AC007879.7 | SMCO3 | 0.346 | 6.31E-16 |
| AC007879.7 | IL12B | 0.346 | 6.33E-16 |
| AC007879.7 | CTIF | 0.346 | 6.41E-16 |
| AC007879.7 | SASH1 | 0.346 | 6.47E-16 |
| AC007879.7 | TXNDC15 | 0.346 | 6.55E-16 |
| AC007879.7 | TIMP1 | 0.346 | 6.65E-16 |
| AC007879.7 | MYO15A | 0.346 | 6.73E-16 |
| AC007879.7 | SNCA | 0.346 | 6.82E-16 |
| AC007879.7 | LRRC4 | 0.346 | 6.89E-16 |
| AC007879.7 | FAM217A | 0.346 | 6.93E-16 |
| AC007879.7 | GABRR3 | 0.346 | 7.00E-16 |
| AC007879.7 | PHLDB1 | 0.346 | 7.03E-16 |
| AC007879.7 | CFAP161 | 0.346 | 7.06E-16 |
| AC007879.7 | GSC | 0.346 | 7.27E-16 |
| AC007879.7 | CFAP69 | 0.347 | 5.07E-16 |
| AC007879.7 | GCM2 | 0.347 | 5.14E-16 |
| AC007879.7 | CR1L | 0.347 | 5.16E-16 |
| AC007879.7 | MED12L | 0.347 | 5.22E-16 |
| AC007879.7 | SNURF | 0.347 | 5.25E-16 |
| AC007879.7 | ARNTL2 | 0.347 | 5.26E-16 |
| AC007879.7 | SUSD5 | 0.347 | 5.27E-16 |
| AC007879.7 | FAM216B | 0.347 | 5.31E-16 |
| AC007879.7 | SERPINB11 | 0.347 | 5.40E-16 |
| AC007879.7 | KCTD20 | 0.347 | 5.47E-16 |
| AC007879.7 | PIK3CA | 0.347 | 5.55E-16 |
| AC007879.7 | HTR1F | 0.347 | 5.81E-16 |
| AC007879.7 | APBB1IP | 0.348 | 4.08E-16 |
| AC007879.7 | GPX7 | 0.348 | 4.11E-16 |
| AC007879.7 | PLA2G7 | 0.348 | 4.12E-16 |
| AC007879.7 | TAS2R50 | 0.348 | 4.22E-16 |
| AC007879.7 | CSRNP2 | 0.348 | 4.24E-16 |
| AC007879.7 | POPDC3 | 0.348 | 4.25E-16 |
| AC007879.7 | RP5-1021I20.4 | 0.348 | 4.49E-16 |
| AC007879.7 | KCNK13 | 0.348 | 4.54E-16 |
| AC007879.7 | TMEM263 | 0.348 | 4.56E-16 |
| AC007879.7 | RNF115 | 0.348 | 4.56E-16 |
| AC007879.7 | CREB1 | 0.348 | 4.59E-16 |
| AC007879.7 | CNTLN | 0.348 | 4.67E-16 |
| AC007879.7 | DNAH11 | 0.348 | 4.73E-16 |
| AC007879.7 | CDH8 | 0.349 | 3.28E-16 |
| AC007879.7 | FTO | 0.349 | 3.29E-16 |
| AC007879.7 | HCK | 0.349 | 3.31E-16 |
| AC007879.7 | OR7C1 | 0.349 | 3.32E-16 |
| AC007879.7 | DOK3 | 0.349 | 3.38E-16 |
| AC007879.7 | TDRD9 | 0.349 | 3.40E-16 |
| AC007879.7 | C18orf54 | 0.349 | 3.40E-16 |
| AC007879.7 | PVRIG | 0.349 | 3.42E-16 |
| AC007879.7 | PDGFRA | 0.349 | 3.43E-16 |
| AC007879.7 | RASAL2 | 0.349 | 3.43E-16 |
| AC007879.7 | OR52N4 | 0.349 | 3.57E-16 |
| AC007879.7 | CCDC39 | 0.349 | 3.57E-16 |
| AC007879.7 | ATL3 | 0.349 | 3.65E-16 |
| AC007879.7 | PATE3 | 0.349 | 3.67E-16 |
| AC007879.7 | CTSB | 0.349 | 3.68E-16 |
| AC007879.7 | TNFRSF8 | 0.349 | 3.74E-16 |
| AC007879.7 | SYTL3 | 0.349 | 3.90E-16 |
| AC007879.7 | PIKFYVE | 0.349 | 3.95E-16 |
| AC007879.7 | C3orf30 | 0.35 | 2.65E-16 |
| AC007879.7 | RNF24 | 0.35 | 2.71E-16 |
| AC007879.7 | ANKRD50 | 0.35 | 2.76E-16 |
| AC007879.7 | GJE1 | 0.35 | 2.78E-16 |
| AC007879.7 | ARHGAP19-SLIT1 | 0.35 | 2.80E-16 |
| AC007879.7 | MEGF10 | 0.35 | 2.85E-16 |
| AC007879.7 | OTULIN | 0.35 | 2.87E-16 |
| AC007879.7 | NBPF26 | 0.35 | 2.88E-16 |
| AC007879.7 | ARMCX1 | 0.35 | 3.05E-16 |
| AC007879.7 | FILIP1 | 0.35 | 3.05E-16 |
| AC007879.7 | TMTC3 | 0.35 | 3.20E-16 |
| AC007879.7 | RP5-1052I5.2 | 0.351 | 2.17E-16 |
| AC007879.7 | FAM124A | 0.351 | 2.18E-16 |
| AC007879.7 | BPGM | 0.351 | 2.19E-16 |
| AC007879.7 | ANGPTL4 | 0.351 | 2.21E-16 |
| AC007879.7 | DIRC3 | 0.351 | 2.23E-16 |
| AC007879.7 | SNED1 | 0.351 | 2.28E-16 |
| AC007879.7 | NCKAP1L | 0.351 | 2.29E-16 |
| AC007879.7 | WDFY4 | 0.351 | 2.35E-16 |
| AC007879.7 | MBNL2 | 0.351 | 2.37E-16 |
| AC007879.7 | EPHA3 | 0.351 | 2.37E-16 |
| AC007879.7 | SLC26A10 | 0.351 | 2.39E-16 |
| AC007879.7 | BTBD18 | 0.351 | 2.41E-16 |
| AC007879.7 | ANKAR | 0.351 | 2.42E-16 |
| AC007879.7 | BEND2 | 0.351 | 2.48E-16 |
| AC007879.7 | PDE6C | 0.351 | 2.57E-16 |
| AC007879.7 | COL18A1 | 0.351 | 2.59E-16 |
| AC007879.7 | LRP12 | 0.351 | 2.61E-16 |
| AC007879.7 | EDARADD | 0.351 | 2.62E-16 |
| AC007879.7 | BICD2 | 0.352 | 1.75E-16 |
| AC007879.7 | TMEM39A | 0.352 | 1.75E-16 |
| AC007879.7 | PARP8 | 0.352 | 1.77E-16 |
| AC007879.7 | C17orf105 | 0.352 | 1.78E-16 |
| AC007879.7 | BBIP1 | 0.352 | 1.79E-16 |
| AC007879.7 | MRVI1 | 0.352 | 1.87E-16 |
| AC007879.7 | CCPG1 | 0.352 | 1.92E-16 |
| AC007879.7 | MAP4K5 | 0.352 | 1.94E-16 |
| AC007879.7 | PXT1 | 0.352 | 2.00E-16 |
| AC007879.7 | FCRL6 | 0.352 | 2.01E-16 |
| AC007879.7 | GNB5 | 0.352 | 2.04E-16 |
| AC007879.7 | PIK3AP1 | 0.352 | 2.12E-16 |
| AC007879.7 | OR1L4 | 0.353 | 1.42E-16 |
| AC007879.7 | ATP8A2 | 0.353 | 1.48E-16 |
| AC007879.7 | LRP2BP | 0.353 | 1.49E-16 |
| AC007879.7 | PLXND1 | 0.353 | 1.52E-16 |
| AC007879.7 | VWC2L | 0.353 | 1.53E-16 |
| AC007879.7 | TMEM119 | 0.353 | 1.58E-16 |
| AC007879.7 | RP11-468E2.4 | 0.353 | 1.62E-16 |
| AC007879.7 | MDGA2 | 0.353 | 1.64E-16 |
| AC007879.7 | C10orf131 | 0.353 | 1.64E-16 |
| AC007879.7 | EVL | 0.353 | 1.65E-16 |
| AC007879.7 | TIAM2 | 0.353 | 1.67E-16 |
| AC007879.7 | WSB1 | 0.353 | 1.69E-16 |
| AC007879.7 | PNRC1 | 0.353 | 1.70E-16 |
| AC007879.7 | PEAR1 | 0.354 | 1.19E-16 |
| AC007879.7 | FAM43B | 0.354 | 1.21E-16 |
| AC007879.7 | HTRA3 | 0.354 | 1.22E-16 |
| AC007879.7 | RP3-382I10.7 | 0.354 | 1.24E-16 |
| AC007879.7 | KLF8 | 0.354 | 1.27E-16 |
| AC007879.7 | PRB4 | 0.354 | 1.31E-16 |
| AC007879.7 | C7orf34 | 0.354 | 1.31E-16 |
| AC007879.7 | CXCR4 | 0.354 | 1.33E-16 |
| AC007879.7 | ACSL1 | 0.354 | 1.33E-16 |
| AC007879.7 | KANSL1L | 0.354 | 1.33E-16 |
| AC007879.7 | RBPJ | 0.354 | 1.36E-16 |
| AC007879.7 | ZDHHC17 | 0.354 | 1.38E-16 |
| AC007879.7 | RASGRP1 | 0.354 | 1.40E-16 |
| AC007879.7 | TEK | 0.355 | 9.46E-17 |
| AC007879.7 | GRID1 | 0.355 | 9.62E-17 |
| AC007879.7 | GSAP | 0.355 | 9.65E-17 |
| AC007879.7 | GIMAP4 | 0.355 | 9.73E-17 |
| AC007879.7 | SLC8A1 | 0.355 | 9.79E-17 |
| AC007879.7 | C5orf47 | 0.355 | 9.96E-17 |
| AC007879.7 | SAR1A | 0.355 | 1.02E-16 |
| AC007879.7 | CD83 | 0.355 | 1.02E-16 |
| AC007879.7 | SLC36A4 | 0.355 | 1.03E-16 |
| AC007879.7 | SP110 | 0.355 | 1.03E-16 |
| AC007879.7 | SLC41A1 | 0.355 | 1.06E-16 |
| AC007879.7 | HACE1 | 0.355 | 1.07E-16 |
| AC007879.7 | LRRC10 | 0.355 | 1.07E-16 |
| AC007879.7 | ABLIM3 | 0.355 | 1.07E-16 |
| AC007879.7 | NAP1L3 | 0.355 | 1.10E-16 |
| AC007879.7 | CEP97 | 0.355 | 1.11E-16 |
| AC007879.7 | RASSF4 | 0.355 | 1.14E-16 |
| AC007879.7 | DDI1 | 0.356 | 7.80E-17 |
| AC007879.7 | SS18 | 0.356 | 7.86E-17 |
| AC007879.7 | HLX | 0.356 | 8.05E-17 |
| AC007879.7 | CRMP1 | 0.356 | 8.06E-17 |
| AC007879.7 | NOTCH2NL | 0.356 | 8.15E-17 |
| AC007879.7 | RHOH | 0.356 | 8.16E-17 |
| AC007879.7 | GIMAP5 | 0.356 | 8.55E-17 |
| AC007879.7 | ZBTB37 | 0.356 | 8.62E-17 |
| AC007879.7 | TRH | 0.356 | 8.77E-17 |
| AC007879.7 | BAALC | 0.356 | 8.81E-17 |
| AC007879.7 | MTMR9 | 0.356 | 8.97E-17 |
| AC007879.7 | CCDC71L | 0.356 | 9.06E-17 |
| AC007879.7 | CEP135 | 0.356 | 9.15E-17 |
| AC007879.7 | TSHR | 0.356 | 9.26E-17 |
| AC007879.7 | PCDHGC4 | 0.357 | 6.19E-17 |
| AC007879.7 | SIX1 | 0.357 | 6.30E-17 |
| AC007879.7 | KCNH7 | 0.357 | 6.30E-17 |
| AC007879.7 | NKIRAS1 | 0.357 | 6.43E-17 |
| AC007879.7 | IFI16 | 0.357 | 6.53E-17 |
| AC007879.7 | HACD4 | 0.357 | 6.66E-17 |
| AC007879.7 | SPDYE1 | 0.357 | 6.73E-17 |
| AC007879.7 | ZBTB46 | 0.357 | 6.77E-17 |
| AC007879.7 | VASN | 0.357 | 6.97E-17 |
| AC007879.7 | ANKUB1 | 0.357 | 7.01E-17 |
| AC007879.7 | TYW5 | 0.357 | 7.23E-17 |
| AC007879.7 | TVP23C | 0.357 | 7.52E-17 |
| AC007879.7 | TBXA2R | 0.357 | 7.53E-17 |
| AC007879.7 | PCDHB15 | 0.358 | 5.02E-17 |
| AC007879.7 | AP1S2 | 0.358 | 5.10E-17 |
| AC007879.7 | KCNAB1 | 0.358 | 5.16E-17 |
| AC007879.7 | EMSY | 0.358 | 5.41E-17 |
| AC007879.7 | XKR5 | 0.358 | 5.45E-17 |
| AC007879.7 | KLHL6 | 0.358 | 5.51E-17 |
| AC007879.7 | SPDYE4 | 0.358 | 5.63E-17 |
| AC007879.7 | PDE6H | 0.358 | 5.65E-17 |
| AC007879.7 | PRDM1 | 0.358 | 5.67E-17 |
| AC007879.7 | CCDC50 | 0.358 | 5.73E-17 |
| AC007879.7 | GLI1 | 0.358 | 5.84E-17 |
| AC007879.7 | SPRY1 | 0.358 | 5.88E-17 |
| AC007879.7 | MAK | 0.358 | 5.89E-17 |
| AC007879.7 | CDC42EP3 | 0.358 | 5.89E-17 |
| AC007879.7 | KCNC3 | 0.358 | 5.90E-17 |
| AC007879.7 | EPG5 | 0.358 | 5.93E-17 |
| AC007879.7 | SHE | 0.358 | 6.05E-17 |
| AC007879.7 | ROBO4 | 0.358 | 6.05E-17 |
| AC007879.7 | ROBO1 | 0.359 | 4.09E-17 |
| AC007879.7 | FAT4 | 0.359 | 4.16E-17 |
| AC007879.7 | ATP10D | 0.359 | 4.20E-17 |
| AC007879.7 | ZNF781 | 0.359 | 4.21E-17 |
| AC007879.7 | ZIM2 | 0.359 | 4.21E-17 |
| AC007879.7 | FGD6 | 0.359 | 4.22E-17 |
| AC007879.7 | LRCH2 | 0.359 | 4.24E-17 |
| AC007879.7 | FMNL1 | 0.359 | 4.25E-17 |
| AC007879.7 | CAPRIN2 | 0.359 | 4.27E-17 |
| AC007879.7 | ARHGAP22 | 0.359 | 4.31E-17 |
| AC007879.7 | DPEP2 | 0.359 | 4.32E-17 |
| AC007879.7 | PRKG1 | 0.359 | 4.36E-17 |
| AC007879.7 | ZNF449 | 0.359 | 4.43E-17 |
| AC007879.7 | PAPOLB | 0.359 | 4.47E-17 |
| AC007879.7 | SIGLEC7 | 0.359 | 4.50E-17 |
| AC007879.7 | ZIK1 | 0.359 | 4.61E-17 |
| AC007879.7 | ARHGAP28 | 0.359 | 4.64E-17 |
| AC007879.7 | PEAK1 | 0.359 | 4.67E-17 |
| AC007879.7 | COL6A2 | 0.359 | 4.78E-17 |
| AC007879.7 | HMGB4 | 0.359 | 4.87E-17 |
| AC007879.7 | PARD6G | 0.359 | 4.88E-17 |
| AC007879.7 | FAM20C | 0.359 | 4.89E-17 |
| AC007879.7 | HIGD1B | 0.359 | 4.90E-17 |
| AC007879.7 | SVEP1 | 0.359 | 4.90E-17 |
| AC007879.7 | SLC12A4 | 0.359 | 4.92E-17 |
| AC007879.7 | REL | 0.36 | 3.24E-17 |
| AC007879.7 | THEGL | 0.36 | 3.25E-17 |
| AC007879.7 | FGF10 | 0.36 | 3.27E-17 |
| AC007879.7 | LRRC17 | 0.36 | 3.28E-17 |
| AC007879.7 | ADAMTS1 | 0.36 | 3.32E-17 |
| AC007879.7 | AWAT2 | 0.36 | 3.37E-17 |
| AC007879.7 | FRG2C | 0.36 | 3.42E-17 |
| AC007879.7 | MBD5 | 0.36 | 3.46E-17 |
| AC007879.7 | NYAP2 | 0.36 | 3.47E-17 |
| AC007879.7 | NABP1 | 0.36 | 3.56E-17 |
| AC007879.7 | TCEAL7 | 0.36 | 3.57E-17 |
| AC007879.7 | SIRPA | 0.36 | 3.59E-17 |
| AC007879.7 | LRRC32 | 0.36 | 3.70E-17 |
| AC007879.7 | TP63 | 0.36 | 3.71E-17 |
| AC007879.7 | VSIG4 | 0.36 | 3.79E-17 |
| AC007879.7 | HAS1 | 0.36 | 3.82E-17 |
| AC007879.7 | RAB3B | 0.36 | 3.86E-17 |
| AC007879.7 | STX11 | 0.36 | 3.91E-17 |
| AC007879.7 | TAOK1 | 0.36 | 3.93E-17 |
| AC007879.7 | FAM205A | 0.36 | 3.94E-17 |
| AC007879.7 | ST8SIA1 | 0.361 | 2.61E-17 |
| AC007879.7 | PRKAR1A | 0.361 | 2.65E-17 |
| AC007879.7 | SNAI1 | 0.361 | 2.67E-17 |
| AC007879.7 | OMD | 0.361 | 2.70E-17 |
| AC007879.7 | FAT2 | 0.361 | 2.73E-17 |
| AC007879.7 | AASS | 0.361 | 2.73E-17 |
| AC007879.7 | PTPRC | 0.361 | 2.74E-17 |
| AC007879.7 | MAP3K19 | 0.361 | 2.74E-17 |
| AC007879.7 | CCDC30 | 0.361 | 2.77E-17 |
| AC007879.7 | RP11-286N22.8 | 0.361 | 2.82E-17 |
| AC007879.7 | TRIM23 | 0.361 | 2.84E-17 |
| AC007879.7 | ZSCAN30 | 0.361 | 2.98E-17 |
| AC007879.7 | MCAM | 0.361 | 3.17E-17 |
| AC007879.7 | AL023806.1 | 0.361 | 3.17E-17 |
| AC007879.7 | CACNB4 | 0.361 | 3.17E-17 |
| AC007879.7 | BTN2A2 | 0.362 | 2.09E-17 |
| AC007879.7 | APELA | 0.362 | 2.12E-17 |
| AC007879.7 | PNMAL2 | 0.362 | 2.14E-17 |
| AC007879.7 | MATR3 | 0.362 | 2.16E-17 |
| AC007879.7 | STAT2 | 0.362 | 2.19E-17 |
| AC007879.7 | BACH1 | 0.362 | 2.25E-17 |
| AC007879.7 | CPLX4 | 0.362 | 2.26E-17 |
| AC007879.7 | EVI2B | 0.362 | 2.29E-17 |
| AC007879.7 | FAM168A | 0.362 | 2.32E-17 |
| AC007879.7 | IL18RAP | 0.362 | 2.45E-17 |
| AC007879.7 | RGS18 | 0.363 | 1.69E-17 |
| AC007879.7 | CHRNE | 0.363 | 1.70E-17 |
| AC007879.7 | ZNF366 | 0.363 | 1.73E-17 |
| AC007879.7 | FADS1 | 0.363 | 1.75E-17 |
| AC007879.7 | BBS9 | 0.363 | 1.78E-17 |
| AC007879.7 | ZNF438 | 0.363 | 1.80E-17 |
| AC007879.7 | MEF2C | 0.363 | 1.80E-17 |
| AC007879.7 | FEZ1 | 0.363 | 1.83E-17 |
| AC007879.7 | MCFD2 | 0.363 | 1.88E-17 |
| AC007879.7 | PSG3 | 0.363 | 1.90E-17 |
| AC007879.7 | LHCGR | 0.363 | 1.93E-17 |
| AC007879.7 | RP11-507M3.1 | 0.363 | 1.93E-17 |
| AC007879.7 | TNFAIP3 | 0.363 | 1.96E-17 |
| AC007879.7 | STARD9 | 0.363 | 2.00E-17 |
| AC007879.7 | GOLGA8M | 0.363 | 2.03E-17 |
| AC007879.7 | WDR49 | 0.363 | 2.07E-17 |
| AC007879.7 | USP44 | 0.364 | 1.38E-17 |
| AC007879.7 | OGFRL1 | 0.364 | 1.41E-17 |
| AC007879.7 | ZNF484 | 0.364 | 1.42E-17 |
| AC007879.7 | PCDHB12 | 0.364 | 1.46E-17 |
| AC007879.7 | DNAJB5 | 0.364 | 1.46E-17 |
| AC007879.7 | CDH20 | 0.364 | 1.47E-17 |
| AC007879.7 | IQSEC3 | 0.364 | 1.48E-17 |
| AC007879.7 | RDX | 0.364 | 1.49E-17 |
| AC007879.7 | C16orf78 | 0.364 | 1.52E-17 |
| AC007879.7 | PCDHGB7 | 0.364 | 1.60E-17 |
| AC007879.7 | TMPRSS11F | 0.364 | 1.60E-17 |
| AC007879.7 | ZNF418 | 0.364 | 1.63E-17 |
| AC007879.7 | IL6ST | 0.364 | 1.65E-17 |
| AC007879.7 | SMCO2 | 0.365 | 1.09E-17 |
| AC007879.7 | MEF2A | 0.365 | 1.10E-17 |
| AC007879.7 | ZNF135 | 0.365 | 1.13E-17 |
| AC007879.7 | SSPN | 0.365 | 1.13E-17 |
| AC007879.7 | ITPR1 | 0.365 | 1.15E-17 |
| AC007879.7 | ADGRA2 | 0.365 | 1.16E-17 |
| AC007879.7 | YAF2 | 0.365 | 1.17E-17 |
| AC007879.7 | LOXL4 | 0.365 | 1.20E-17 |
| AC007879.7 | UBASH3B | 0.365 | 1.24E-17 |
| AC007879.7 | ZNF331 | 0.365 | 1.24E-17 |
| AC007879.7 | PRSS55 | 0.365 | 1.25E-17 |
| AC007879.7 | PRG4 | 0.365 | 1.30E-17 |
| AC007879.7 | MN1 | 0.365 | 1.30E-17 |
| AC007879.7 | DCLK3 | 0.365 | 1.34E-17 |
| AC007879.7 | MDGA1 | 0.366 | 9.12E-18 |
| AC007879.7 | MS4A4A | 0.366 | 9.13E-18 |
| AC007879.7 | ACSS3 | 0.366 | 9.13E-18 |
| AC007879.7 | CLEC12A | 0.366 | 9.21E-18 |
| AC007879.7 | PTAFR | 0.366 | 9.47E-18 |
| AC007879.7 | ZNF546 | 0.366 | 9.53E-18 |
| AC007879.7 | CCL4L2 | 0.366 | 9.68E-18 |
| AC007879.7 | CRYBA1 | 0.366 | 9.80E-18 |
| AC007879.7 | CYLD | 0.366 | 1.02E-17 |
| AC007879.7 | SHOX2 | 0.366 | 1.03E-17 |
| AC007879.7 | THSD1 | 0.366 | 1.05E-17 |
| AC007879.7 | PBX3 | 0.366 | 1.05E-17 |
| AC007879.7 | TXNDC2 | 0.367 | 7.03E-18 |
| AC007879.7 | AKAP2 | 0.367 | 7.21E-18 |
| AC007879.7 | GPR52 | 0.367 | 7.48E-18 |
| AC007879.7 | CEP112 | 0.367 | 7.62E-18 |
| AC007879.7 | ALS2 | 0.367 | 7.86E-18 |
| AC007879.7 | BACE1 | 0.367 | 7.90E-18 |
| AC007879.7 | RBMS2 | 0.367 | 7.96E-18 |
| AC007879.7 | HEATR9 | 0.367 | 8.10E-18 |
| AC007879.7 | LSAMP | 0.367 | 8.11E-18 |
| AC007879.7 | OLAH | 0.367 | 8.13E-18 |
| AC007879.7 | CDR1 | 0.367 | 8.15E-18 |
| AC007879.7 | C15orf43 | 0.367 | 8.28E-18 |
| AC007879.7 | IKZF2 | 0.367 | 8.32E-18 |
| AC007879.7 | FCRLB | 0.367 | 8.51E-18 |
| AC007879.7 | MS4A4E | 0.367 | 8.57E-18 |
| AC007879.7 | RGS20 | 0.367 | 8.66E-18 |
| AC007879.7 | SEC24D | 0.368 | 5.62E-18 |
| AC007879.7 | PCDHGC5 | 0.368 | 5.67E-18 |
| AC007879.7 | C11orf65 | 0.368 | 5.68E-18 |
| AC007879.7 | SLC43A3 | 0.368 | 5.74E-18 |
| AC007879.7 | CPXM1 | 0.368 | 6.02E-18 |
| AC007879.7 | KLF12 | 0.368 | 6.05E-18 |
| AC007879.7 | AGRP | 0.368 | 6.46E-18 |
| AC007879.7 | IGSF6 | 0.368 | 6.46E-18 |
| AC007879.7 | CBL | 0.368 | 6.69E-18 |
| AC007879.7 | MYH8 | 0.368 | 6.70E-18 |
| AC007879.7 | KIAA1210 | 0.368 | 6.80E-18 |
| AC007879.7 | ZNF483 | 0.369 | 4.62E-18 |
| AC007879.7 | FRY | 0.369 | 4.63E-18 |
| AC007879.7 | NFAM1 | 0.369 | 4.68E-18 |
| AC007879.7 | ZNF732 | 0.369 | 4.71E-18 |
| AC007879.7 | EHD2 | 0.369 | 4.75E-18 |
| AC007879.7 | MID2 | 0.369 | 4.77E-18 |
| AC007879.7 | ST3GAL1 | 0.369 | 4.78E-18 |
| AC007879.7 | SESTD1 | 0.369 | 4.97E-18 |
| AC007879.7 | PCNXL4 | 0.369 | 5.08E-18 |
| AC007879.7 | CILP2 | 0.369 | 5.09E-18 |
| AC007879.7 | RTL1 | 0.369 | 5.29E-18 |
| AC007879.7 | SOX7 | 0.369 | 5.40E-18 |
| AC007879.7 | PLPP7 | 0.369 | 5.40E-18 |
| AC007879.7 | STX2 | 0.369 | 5.43E-18 |
| AC007879.7 | WTIP | 0.369 | 5.55E-18 |
| AC007879.7 | BBX | 0.369 | 5.59E-18 |
| AC007879.7 | SH3PXD2A | 0.37 | 3.66E-18 |
| AC007879.7 | RGS1 | 0.37 | 3.84E-18 |
| AC007879.7 | CSRP3 | 0.37 | 3.99E-18 |
| AC007879.7 | FGF12 | 0.37 | 4.02E-18 |
| AC007879.7 | PNMAL1 | 0.37 | 4.04E-18 |
| AC007879.7 | RP11-111H13.1 | 0.37 | 4.08E-18 |
| AC007879.7 | ACAD11 | 0.37 | 4.17E-18 |
| AC007879.7 | WDFY3 | 0.37 | 4.21E-18 |
| AC007879.7 | RAB23 | 0.37 | 4.35E-18 |
| AC007879.7 | NPR3 | 0.37 | 4.40E-18 |
| AC007879.7 | KIAA1024 | 0.37 | 4.40E-18 |
| AC007879.7 | TPBG | 0.37 | 4.43E-18 |
| AC007879.7 | FGF2 | 0.371 | 2.93E-18 |
| AC007879.7 | GABARAPL1 | 0.371 | 3.01E-18 |
| AC007879.7 | DNAJB4 | 0.371 | 3.02E-18 |
| AC007879.7 | SH2D4B | 0.371 | 3.13E-18 |
| AC007879.7 | CDK17 | 0.371 | 3.14E-18 |
| AC007879.7 | TMTC1 | 0.371 | 3.21E-18 |
| AC007879.7 | MAMLD1 | 0.371 | 3.25E-18 |
| AC007879.7 | TRIB2 | 0.371 | 3.28E-18 |
| AC007879.7 | C16orf52 | 0.371 | 3.30E-18 |
| AC007879.7 | IL1B | 0.371 | 3.33E-18 |
| AC007879.7 | PRDM6 | 0.371 | 3.42E-18 |
| AC007879.7 | IGDCC4 | 0.371 | 3.48E-18 |
| AC007879.7 | ZNF410 | 0.371 | 3.48E-18 |
| AC007879.7 | HOXC9 | 0.372 | 2.34E-18 |
| AC007879.7 | GAS7 | 0.372 | 2.36E-18 |
| AC007879.7 | PLEKHO1 | 0.372 | 2.56E-18 |
| AC007879.7 | SLC9C1 | 0.372 | 2.58E-18 |
| AC007879.7 | HCLS1 | 0.372 | 2.65E-18 |
| AC007879.7 | ELOVL2 | 0.372 | 2.77E-18 |
| AC007879.7 | SNX18 | 0.372 | 2.82E-18 |
| AC007879.7 | NFATC1 | 0.373 | 1.84E-18 |
| AC007879.7 | C1R | 0.373 | 1.86E-18 |
| AC007879.7 | KLF7 | 0.373 | 1.90E-18 |
| AC007879.7 | SNTB2 | 0.373 | 1.94E-18 |
| AC007879.7 | EGFL6 | 0.373 | 1.99E-18 |
| AC007879.7 | GADL1 | 0.373 | 1.99E-18 |
| AC007879.7 | SHC4 | 0.373 | 2.06E-18 |
| AC007879.7 | SYT14 | 0.373 | 2.09E-18 |
| AC007879.7 | KIAA1549L | 0.373 | 2.22E-18 |
| AC007879.7 | C2orf74 | 0.373 | 2.22E-18 |
| AC007879.7 | NCKAP5L | 0.373 | 2.27E-18 |
| AC007879.7 | LCP1 | 0.374 | 1.51E-18 |
| AC007879.7 | PM20D1 | 0.374 | 1.53E-18 |
| AC007879.7 | C17orf107 | 0.374 | 1.55E-18 |
| AC007879.7 | OR2A5 | 0.374 | 1.56E-18 |
| AC007879.7 | LILRA5 | 0.374 | 1.59E-18 |
| AC007879.7 | BOC | 0.374 | 1.63E-18 |
| AC007879.7 | USHBP1 | 0.374 | 1.65E-18 |
| AC007879.7 | CACNA2D3 | 0.374 | 1.65E-18 |
| AC007879.7 | CTC-273B12.10 | 0.374 | 1.65E-18 |
| AC007879.7 | AC009404.2 | 0.374 | 1.65E-18 |
| AC007879.7 | AC073283.7 | 0.374 | 1.65E-18 |
| AC007879.7 | RP11-167H9.4 | 0.374 | 1.65E-18 |
| AC007879.7 | AC007879.7 | 0.374 | 1.65E-18 |
| AC007879.7 | RP4-816N1.7 | 0.374 | 1.65E-18 |
| AC007879.7 | RP11-400N13.2 | 0.374 | 1.65E-18 |
| AC007879.7 | FMO1 | 0.374 | 1.69E-18 |
| AC007879.7 | MAP2 | 0.374 | 1.72E-18 |
| AC007879.7 | ZNF699 | 0.374 | 1.74E-18 |
| AC007879.7 | C12orf40 | 0.374 | 1.75E-18 |
| AC007879.7 | CTD-2370N5.3 | 0.374 | 1.76E-18 |
| AC007879.7 | CDO1 | 0.374 | 1.79E-18 |
| AC007879.7 | SCEL | 0.374 | 1.82E-18 |
| AC007879.7 | NLGN4X | 0.375 | 1.19E-18 |
| AC007879.7 | MFAP2 | 0.375 | 1.20E-18 |
| AC007879.7 | GGN | 0.375 | 1.22E-18 |
| AC007879.7 | NUDT11 | 0.375 | 1.24E-18 |
| AC007879.7 | SORCS2 | 0.375 | 1.25E-18 |
| AC007879.7 | ZNF474 | 0.375 | 1.27E-18 |
| AC007879.7 | FAM19A2 | 0.375 | 1.27E-18 |
| AC007879.7 | TNS1 | 0.375 | 1.30E-18 |
| AC007879.7 | SOST | 0.375 | 1.34E-18 |
| AC007879.7 | ZNF812P | 0.375 | 1.36E-18 |
| AC007879.7 | SPATC1 | 0.375 | 1.36E-18 |
| AC007879.7 | CRYGN | 0.375 | 1.39E-18 |
| AC007879.7 | DSPP | 0.375 | 1.42E-18 |
| AC007879.7 | KCNQ5 | 0.375 | 1.45E-18 |
| AC007879.7 | RP11-248J23.7 | 0.376 | 9.88E-19 |
| AC007879.7 | UGCG | 0.376 | 9.89E-19 |
| AC007879.7 | NR4A3 | 0.376 | 9.95E-19 |
| AC007879.7 | COLQ | 0.376 | 1.00E-18 |
| AC007879.7 | ACTA2 | 0.376 | 1.02E-18 |
| AC007879.7 | NAB1 | 0.376 | 1.02E-18 |
| AC007879.7 | DNAL1 | 0.376 | 1.04E-18 |
| AC007879.7 | TMEM106A | 0.376 | 1.04E-18 |
| AC007879.7 | PHTF1 | 0.376 | 1.06E-18 |
| AC007879.7 | SEMA7A | 0.376 | 1.11E-18 |
| AC007879.7 | SYNC | 0.376 | 1.12E-18 |
| AC007879.7 | EFNB3 | 0.376 | 1.13E-18 |
| AC007879.7 | PICALM | 0.376 | 1.15E-18 |
| AC007879.7 | ITGA4 | 0.376 | 1.17E-18 |
| AC007879.7 | ADD2 | 0.377 | 7.50E-19 |
| AC007879.7 | SLC15A5 | 0.377 | 7.52E-19 |
| AC007879.7 | SLC38A2 | 0.377 | 7.70E-19 |
| AC007879.7 | MS4A6E | 0.377 | 8.20E-19 |
| AC007879.7 | F13B | 0.377 | 8.22E-19 |
| AC007879.7 | IL10 | 0.377 | 8.35E-19 |
| AC007879.7 | APLNR | 0.377 | 8.43E-19 |
| AC007879.7 | RCN3 | 0.377 | 8.48E-19 |
| AC007879.7 | RIT1 | 0.377 | 8.53E-19 |
| AC007879.7 | TDO2 | 0.377 | 9.17E-19 |
| AC007879.7 | SLFN12L | 0.377 | 9.22E-19 |
| AC007879.7 | RAG2 | 0.377 | 9.23E-19 |
| AC007879.7 | RAB34 | 0.377 | 9.26E-19 |
| AC007879.7 | MYO1F | 0.378 | 5.99E-19 |
| AC007879.7 | BBOF1 | 0.378 | 6.27E-19 |
| AC007879.7 | MAF | 0.378 | 6.35E-19 |
| AC007879.7 | CDKL5 | 0.378 | 6.52E-19 |
| AC007879.7 | BMPR2 | 0.378 | 6.61E-19 |
| AC007879.7 | NUDT3 | 0.378 | 6.62E-19 |
| AC007879.7 | PRCD | 0.378 | 6.69E-19 |
| AC007879.7 | DNAJC24 | 0.378 | 6.85E-19 |
| AC007879.7 | SFXN3 | 0.378 | 6.87E-19 |
| AC007879.7 | INPP4A | 0.378 | 6.93E-19 |
| AC007879.7 | RBFOX2 | 0.378 | 7.02E-19 |
| AC007879.7 | RP11-382A20.3 | 0.378 | 7.11E-19 |
| AC007879.7 | IGFBP7 | 0.378 | 7.32E-19 |
| AC007879.7 | DOCK8 | 0.378 | 7.44E-19 |
| AC007879.7 | DRAM1 | 0.379 | 4.82E-19 |
| AC007879.7 | PCDHGA3 | 0.379 | 4.87E-19 |
| AC007879.7 | FAM160B1 | 0.379 | 4.91E-19 |
| AC007879.7 | KCNK2 | 0.379 | 4.95E-19 |
| AC007879.7 | MIB1 | 0.379 | 5.01E-19 |
| AC007879.7 | CAV1 | 0.379 | 5.06E-19 |
| AC007879.7 | ANXA5 | 0.379 | 5.08E-19 |
| AC007879.7 | CCDC3 | 0.379 | 5.58E-19 |
| AC007879.7 | PIK3R6 | 0.379 | 5.63E-19 |
| AC007879.7 | FAM196A | 0.379 | 5.71E-19 |
| AC007879.7 | ARHGEF6 | 0.379 | 5.75E-19 |
| AC007879.7 | MSANTD3 | 0.379 | 5.78E-19 |
| AC007879.7 | FMO2 | 0.379 | 5.80E-19 |
| AC007879.7 | GABRR2 | 0.38 | 3.86E-19 |
| AC007879.7 | FOXC2 | 0.38 | 3.91E-19 |
| AC007879.7 | PCDHA10 | 0.38 | 3.98E-19 |
| AC007879.7 | PRRX2 | 0.38 | 4.12E-19 |
| AC007879.7 | TMPRSS12 | 0.38 | 4.15E-19 |
| AC007879.7 | EMCN | 0.38 | 4.22E-19 |
| AC007879.7 | TAL1 | 0.38 | 4.42E-19 |
| AC007879.7 | FER1L5 | 0.38 | 4.61E-19 |
| AC007879.7 | SCARF2 | 0.38 | 4.62E-19 |
| AC007879.7 | RAB12 | 0.381 | 3.01E-19 |
| AC007879.7 | CHRD | 0.381 | 3.08E-19 |
| AC007879.7 | PTGS2 | 0.381 | 3.55E-19 |
| AC007879.7 | IFIT1B | 0.381 | 3.61E-19 |
| AC007879.7 | SGCE | 0.381 | 3.63E-19 |
| AC007879.7 | APBB2 | 0.381 | 3.63E-19 |
| AC007879.7 | CD274 | 0.381 | 3.67E-19 |
| AC007879.7 | GNG11 | 0.381 | 3.69E-19 |
| AC007879.7 | GPR21 | 0.381 | 3.70E-19 |
| AC007879.7 | DTHD1 | 0.381 | 3.71E-19 |
| AC007879.7 | PLN | 0.382 | 2.47E-19 |
| AC007879.7 | CD53 | 0.382 | 2.48E-19 |
| AC007879.7 | EPB42 | 0.382 | 2.56E-19 |
| AC007879.7 | EFEMP1 | 0.382 | 2.56E-19 |
| AC007879.7 | TMEM200B | 0.382 | 2.62E-19 |
| AC007879.7 | RSPO3 | 0.382 | 2.64E-19 |
| AC007879.7 | ENTHD1 | 0.382 | 2.66E-19 |
| AC007879.7 | GPR180 | 0.382 | 2.66E-19 |
| AC007879.7 | ASB3 | 0.382 | 2.68E-19 |
| AC007879.7 | SOX5 | 0.382 | 2.75E-19 |
| AC007879.7 | IL2RA | 0.382 | 2.76E-19 |
| AC007879.7 | ANKRD34A | 0.382 | 2.87E-19 |
| AC007879.7 | SV2B | 0.382 | 2.93E-19 |
| AC007879.7 | ATM | 0.382 | 2.95E-19 |
| AC007879.7 | KERA | 0.382 | 2.96E-19 |
| AC007879.7 | SAMD8 | 0.383 | 1.93E-19 |
| AC007879.7 | FSTL3 | 0.383 | 1.99E-19 |
| AC007879.7 | DKK2 | 0.383 | 1.99E-19 |
| AC007879.7 | WNT9B | 0.383 | 2.02E-19 |
| AC007879.7 | CYTH4 | 0.383 | 2.13E-19 |
| AC007879.7 | SELL | 0.383 | 2.15E-19 |
| AC007879.7 | HIP1 | 0.383 | 2.23E-19 |
| AC007879.7 | GPR137B | 0.383 | 2.25E-19 |
| AC007879.7 | PCOLCE | 0.383 | 2.27E-19 |
| AC007879.7 | RANBP9 | 0.383 | 2.29E-19 |
| AC007879.7 | MXRA7 | 0.383 | 2.34E-19 |
| AC007879.7 | VNN3 | 0.384 | 1.50E-19 |
| AC007879.7 | FYB | 0.384 | 1.53E-19 |
| AC007879.7 | PHF21A | 0.384 | 1.55E-19 |
| AC007879.7 | C4orf22 | 0.384 | 1.68E-19 |
| AC007879.7 | TBCEL | 0.384 | 1.68E-19 |
| AC007879.7 | FCHSD2 | 0.384 | 1.74E-19 |
| AC007879.7 | CC2D2B | 0.384 | 1.74E-19 |
| AC007879.7 | BDNF | 0.384 | 1.80E-19 |
| AC007879.7 | MMP9 | 0.385 | 1.20E-19 |
| AC007879.7 | TNFSF14 | 0.385 | 1.20E-19 |
| AC007879.7 | SLC39A6 | 0.385 | 1.22E-19 |
| AC007879.7 | ZBED6 | 0.385 | 1.23E-19 |
| AC007879.7 | CMTM2 | 0.385 | 1.29E-19 |
| AC007879.7 | ABI2 | 0.385 | 1.34E-19 |
| AC007879.7 | CD34 | 0.385 | 1.38E-19 |
| AC007879.7 | ADCY4 | 0.385 | 1.43E-19 |
| AC007879.7 | DOCK2 | 0.385 | 1.44E-19 |
| AC007879.7 | CFLAR | 0.385 | 1.44E-19 |
| AC007879.7 | ROR2 | 0.385 | 1.45E-19 |
| AC007879.7 | CSF2RB | 0.385 | 1.45E-19 |
| AC007879.7 | C4orf50 | 0.385 | 1.46E-19 |
| AC007879.7 | UACA | 0.385 | 1.47E-19 |
| AC007879.7 | TBATA | 0.386 | 9.35E-20 |
| AC007879.7 | PRR29 | 0.386 | 9.58E-20 |
| AC007879.7 | DPYSL4 | 0.386 | 9.62E-20 |
| AC007879.7 | GPR183 | 0.386 | 9.79E-20 |
| AC007879.7 | NDEL1 | 0.386 | 9.87E-20 |
| AC007879.7 | AGO3 | 0.386 | 1.01E-19 |
| AC007879.7 | MAP3K8 | 0.386 | 1.09E-19 |
| AC007879.7 | SERPING1 | 0.386 | 1.14E-19 |
| AC007879.7 | FHL3 | 0.387 | 7.50E-20 |
| AC007879.7 | PRTG | 0.387 | 7.71E-20 |
| AC007879.7 | RGS2 | 0.387 | 7.95E-20 |
| AC007879.7 | C8orf48 | 0.387 | 7.97E-20 |
| AC007879.7 | GPR173 | 0.387 | 8.78E-20 |
| AC007879.7 | OR6B1 | 0.387 | 8.86E-20 |
| AC007879.7 | SLC26A5 | 0.388 | 5.96E-20 |
| AC007879.7 | ZCCHC18 | 0.388 | 6.00E-20 |
| AC007879.7 | GIPC3 | 0.388 | 6.21E-20 |
| AC007879.7 | PAEP | 0.388 | 6.33E-20 |
| AC007879.7 | CLEC2D | 0.388 | 6.47E-20 |
| AC007879.7 | SUCNR1 | 0.388 | 7.07E-20 |
| AC007879.7 | KIAA1614 | 0.388 | 7.12E-20 |
| AC007879.7 | FNDC3B | 0.388 | 7.16E-20 |
| AC007879.7 | MEIKIN | 0.388 | 7.18E-20 |
| AC007879.7 | ALS2CR12 | 0.388 | 7.20E-20 |
| AC007879.7 | IQCG | 0.389 | 4.58E-20 |
| AC007879.7 | MMP1 | 0.389 | 4.79E-20 |
| AC007879.7 | ZNF843 | 0.389 | 4.81E-20 |
| AC007879.7 | RASGRF2 | 0.389 | 4.87E-20 |
| AC007879.7 | SLC10A6 | 0.389 | 4.87E-20 |
| AC007879.7 | ADAP2 | 0.389 | 5.04E-20 |
| AC007879.7 | ADPRH | 0.389 | 5.06E-20 |
| AC007879.7 | NXPE3 | 0.389 | 5.14E-20 |
| AC007879.7 | KRT79 | 0.389 | 5.17E-20 |
| AC007879.7 | ADAM23 | 0.389 | 5.27E-20 |
| AC007879.7 | EFEMP2 | 0.389 | 5.60E-20 |
| AC007879.7 | SIGLEC14 | 0.389 | 5.67E-20 |
| AC007879.7 | GPR78 | 0.389 | 5.68E-20 |
| AC007879.7 | WDR26 | 0.39 | 3.67E-20 |
| AC007879.7 | IFFO1 | 0.39 | 3.71E-20 |
| AC007879.7 | ENTPD7 | 0.39 | 3.74E-20 |
| AC007879.7 | ZDHHC15 | 0.39 | 3.84E-20 |
| AC007879.7 | CXCR2 | 0.39 | 4.02E-20 |
| AC007879.7 | MYOZ3 | 0.39 | 4.13E-20 |
| AC007879.7 | PTGIS | 0.39 | 4.25E-20 |
| AC007879.7 | LOXL1 | 0.39 | 4.30E-20 |
| AC007879.7 | CHI3L1 | 0.39 | 4.33E-20 |
| AC007879.7 | KIAA0825 | 0.39 | 4.34E-20 |
| AC007879.7 | FMO3 | 0.391 | 2.90E-20 |
| AC007879.7 | CATSPER1 | 0.391 | 2.96E-20 |
| AC007879.7 | PTPRN | 0.391 | 3.05E-20 |
| AC007879.7 | ARHGAP15 | 0.391 | 3.10E-20 |
| AC007879.7 | PCDHB3 | 0.391 | 3.15E-20 |
| AC007879.7 | GML | 0.391 | 3.19E-20 |
| AC007879.7 | DEGS1 | 0.391 | 3.23E-20 |
| AC007879.7 | RASGRP4 | 0.391 | 3.26E-20 |
| AC007879.7 | STRADA | 0.391 | 3.39E-20 |
| AC007879.7 | BNIP2 | 0.391 | 3.42E-20 |
| AC007879.7 | DMP1 | 0.391 | 3.43E-20 |
| AC007879.7 | CLNK | 0.392 | 2.25E-20 |
| AC007879.7 | ABCG4 | 0.392 | 2.31E-20 |
| AC007879.7 | ZNF382 | 0.392 | 2.32E-20 |
| AC007879.7 | SNX10 | 0.392 | 2.34E-20 |
| AC007879.7 | CTSL | 0.392 | 2.35E-20 |
| AC007879.7 | IQUB | 0.392 | 2.39E-20 |
| AC007879.7 | FAM234B | 0.392 | 2.42E-20 |
| AC007879.7 | IGSF21 | 0.392 | 2.45E-20 |
| AC007879.7 | FER | 0.392 | 2.54E-20 |
| AC007879.7 | FBXO15 | 0.392 | 2.62E-20 |
| AC007879.7 | ZNF705A | 0.393 | 1.81E-20 |
| AC007879.7 | TUB | 0.393 | 1.87E-20 |
| AC007879.7 | MXRA8 | 0.393 | 1.90E-20 |
| AC007879.7 | DPYD | 0.393 | 1.96E-20 |
| AC007879.7 | MYOZ2 | 0.393 | 2.00E-20 |
| AC007879.7 | FAM228A | 0.393 | 2.03E-20 |
| AC007879.7 | BEAN1 | 0.393 | 2.09E-20 |
| AC007879.7 | LILRB1 | 0.393 | 2.15E-20 |
| AC007879.7 | NOTCH2 | 0.393 | 2.18E-20 |
| AC007879.7 | KLHL38 | 0.394 | 1.39E-20 |
| AC007879.7 | FCGR3B | 0.394 | 1.43E-20 |
| AC007879.7 | CD72 | 0.394 | 1.43E-20 |
| AC007879.7 | OPN5 | 0.394 | 1.43E-20 |
| AC007879.7 | JAM3 | 0.394 | 1.45E-20 |
| AC007879.7 | PIAS3 | 0.394 | 1.51E-20 |
| AC007879.7 | TTBK2 | 0.394 | 1.58E-20 |
| AC007879.7 | FAM229B | 0.394 | 1.59E-20 |
| AC007879.7 | TMEM86A | 0.394 | 1.62E-20 |
| AC007879.7 | TRIM6 | 0.394 | 1.65E-20 |
| AC007879.7 | AMTN | 0.394 | 1.74E-20 |
| AC007879.7 | FLT4 | 0.395 | 1.09E-20 |
| AC007879.7 | DRAXIN | 0.395 | 1.13E-20 |
| AC007879.7 | FGF11 | 0.395 | 1.21E-20 |
| AC007879.7 | ZNF354C | 0.395 | 1.26E-20 |
| AC007879.7 | LAMP5 | 0.395 | 1.34E-20 |
| AC007879.7 | TET1 | 0.395 | 1.34E-20 |
| AC007879.7 | SV2A | 0.395 | 1.38E-20 |
| AC007879.7 | CEP295NL | 0.396 | 9.25E-21 |
| AC007879.7 | ANGPT1 | 0.396 | 9.88E-21 |
| AC007879.7 | ZSCAN23 | 0.396 | 9.98E-21 |
| AC007879.7 | PTX3 | 0.396 | 1.00E-20 |
| AC007879.7 | LAMB4 | 0.396 | 1.02E-20 |
| AC007879.7 | SLFN5 | 0.396 | 1.03E-20 |
| AC007879.7 | GPR135 | 0.397 | 7.20E-21 |
| AC007879.7 | BHMT2 | 0.397 | 7.34E-21 |
| AC007879.7 | NIN | 0.397 | 7.46E-21 |
| AC007879.7 | TRPC4 | 0.397 | 8.23E-21 |
| AC007879.7 | IL31RA | 0.397 | 8.46E-21 |
| AC007879.7 | OSBPL8 | 0.398 | 5.22E-21 |
| AC007879.7 | KIAA1755 | 0.398 | 5.59E-21 |
| AC007879.7 | CXCL5 | 0.398 | 5.61E-21 |
| AC007879.7 | SLC24A4 | 0.398 | 5.96E-21 |
| AC007879.7 | PTGFR | 0.398 | 6.13E-21 |
| AC007879.7 | PRKCH | 0.398 | 6.35E-21 |
| AC007879.7 | PODNL1 | 0.398 | 6.51E-21 |
| AC007879.7 | CYYR1 | 0.398 | 6.53E-21 |
| AC007879.7 | PCDHB6 | 0.398 | 6.63E-21 |
| AC007879.7 | GLRB | 0.399 | 4.08E-21 |
| AC007879.7 | NLGN2 | 0.399 | 4.12E-21 |
| AC007879.7 | ARL13B | 0.399 | 4.16E-21 |
| AC007879.7 | C1QTNF3 | 0.399 | 4.34E-21 |
| AC007879.7 | PCDHB7 | 0.399 | 4.46E-21 |
| AC007879.7 | CCL4 | 0.399 | 4.56E-21 |
| AC007879.7 | HCAR3 | 0.399 | 4.58E-21 |
| AC007879.7 | SUMO4 | 0.399 | 4.62E-21 |
| AC007879.7 | C4orf47 | 0.399 | 4.68E-21 |
| AC007879.7 | ICAM1 | 0.399 | 4.86E-21 |
| AC007879.7 | CD300LB | 0.399 | 4.91E-21 |
| AC007879.7 | CCDC7 | 0.399 | 4.96E-21 |
| AC007879.7 | HIVEP3 | 0.399 | 5.14E-21 |
| AC007879.7 | POU2F2 | 0.4 | 3.25E-21 |
| AC007879.7 | HAPLN3 | 0.4 | 3.25E-21 |
| AC007879.7 | LAT2 | 0.4 | 3.41E-21 |
| AC007879.7 | TPTE2 | 0.4 | 3.48E-21 |
| AC007879.7 | SCUBE3 | 0.4 | 3.55E-21 |
| AC007879.7 | SNX31 | 0.4 | 3.58E-21 |
| AC007879.7 | IL1RL1 | 0.4 | 3.62E-21 |
| AC007879.7 | SCARF1 | 0.4 | 3.63E-21 |
| AC007879.7 | ADAM8 | 0.4 | 3.73E-21 |
| AC007879.7 | C1orf54 | 0.4 | 3.89E-21 |
| AC007879.7 | DDHD1 | 0.4 | 3.94E-21 |
| AC007879.7 | ZNF697 | 0.4 | 4.06E-21 |
| AC007879.7 | ZDBF2 | 0.401 | 2.52E-21 |
| AC007879.7 | SNX29 | 0.401 | 2.58E-21 |
| AC007879.7 | GPR137C | 0.401 | 2.59E-21 |
| AC007879.7 | ARSB | 0.401 | 2.61E-21 |
| AC007879.7 | GPATCH2L | 0.401 | 2.72E-21 |
| AC007879.7 | AKAP12 | 0.401 | 2.89E-21 |
| AC007879.7 | BACH2 | 0.401 | 2.90E-21 |
| AC007879.7 | ADARB1 | 0.401 | 3.08E-21 |
| AC007879.7 | SOCS5 | 0.402 | 1.99E-21 |
| AC007879.7 | ELL2 | 0.402 | 2.01E-21 |
| AC007879.7 | ALDH1A3 | 0.402 | 2.05E-21 |
| AC007879.7 | FAM65B | 0.402 | 2.09E-21 |
| AC007879.7 | FAM182B | 0.402 | 2.13E-21 |
| AC007879.7 | ZNF804A | 0.402 | 2.19E-21 |
| AC007879.7 | ST8SIA2 | 0.402 | 2.20E-21 |
| AC007879.7 | NUP210L | 0.402 | 2.24E-21 |
| AC007879.7 | ANKDD1A | 0.402 | 2.24E-21 |
| AC007879.7 | SLN | 0.402 | 2.32E-21 |
| AC007879.7 | ECSCR | 0.402 | 2.32E-21 |
| AC007879.7 | COL6A6 | 0.402 | 2.33E-21 |
| AC007879.7 | ZNF99 | 0.402 | 2.45E-21 |
| AC007879.7 | STON1-GTF2A1L | 0.402 | 2.48E-21 |
| AC007879.7 | CNTNAP1 | 0.403 | 1.52E-21 |
| AC007879.7 | NPC1 | 0.403 | 1.61E-21 |
| AC007879.7 | FCER1G | 0.403 | 1.62E-21 |
| AC007879.7 | FGFR1 | 0.403 | 1.64E-21 |
| AC007879.7 | ANKRD44 | 0.403 | 1.64E-21 |
| AC007879.7 | ADAMTS7 | 0.403 | 1.71E-21 |
| AC007879.7 | NPL | 0.403 | 1.74E-21 |
| AC007879.7 | FCN1 | 0.403 | 1.74E-21 |
| AC007879.7 | GLIPR1 | 0.403 | 1.75E-21 |
| AC007879.7 | NXPH2 | 0.403 | 1.87E-21 |
| AC007879.7 | GRM5 | 0.404 | 1.26E-21 |
| AC007879.7 | HTRA4 | 0.404 | 1.27E-21 |
| AC007879.7 | TLR8 | 0.404 | 1.44E-21 |
| AC007879.7 | OLFML1 | 0.404 | 1.50E-21 |
| AC007879.7 | RBMS3 | 0.405 | 9.29E-22 |
| AC007879.7 | IGFBP3 | 0.405 | 9.96E-22 |
| AC007879.7 | BMPR1B | 0.405 | 1.02E-21 |
| AC007879.7 | S100PBP | 0.405 | 1.07E-21 |
| AC007879.7 | GPR65 | 0.405 | 1.10E-21 |
| AC007879.7 | CPZ | 0.405 | 1.15E-21 |
| AC007879.7 | CCDC184 | 0.405 | 1.17E-21 |
| AC007879.7 | LAMA2 | 0.406 | 7.23E-22 |
| AC007879.7 | MAP9 | 0.406 | 7.48E-22 |
| AC007879.7 | PDLIM3 | 0.406 | 7.53E-22 |
| AC007879.7 | ADAMTS18 | 0.406 | 7.55E-22 |
| AC007879.7 | S1PR1 | 0.406 | 7.63E-22 |
| AC007879.7 | OSTM1 | 0.406 | 7.66E-22 |
| AC007879.7 | CCL18 | 0.406 | 8.31E-22 |
| AC007879.7 | GLIS2 | 0.406 | 8.33E-22 |
| AC007879.7 | THPO | 0.406 | 8.50E-22 |
| AC007879.7 | WNT5A | 0.406 | 8.78E-22 |
| AC007879.7 | CERS5 | 0.407 | 5.58E-22 |
| AC007879.7 | FBXL13 | 0.407 | 5.69E-22 |
| AC007879.7 | SYT16 | 0.407 | 5.88E-22 |
| AC007879.7 | NR3C1 | 0.407 | 6.24E-22 |
| AC007879.7 | TWIST2 | 0.407 | 6.52E-22 |
| AC007879.7 | CSGALNACT1 | 0.407 | 6.52E-22 |
| AC007879.7 | LIX1L | 0.407 | 6.58E-22 |
| AC007879.7 | ZNF333 | 0.407 | 6.63E-22 |
| AC007879.7 | TAS2R40 | 0.407 | 6.73E-22 |
| AC007879.7 | FAM19A5 | 0.407 | 6.88E-22 |
| AC007879.7 | PDE1A | 0.408 | 4.31E-22 |
| AC007879.7 | HOXC8 | 0.408 | 4.55E-22 |
| AC007879.7 | MFGE8 | 0.408 | 4.55E-22 |
| AC007879.7 | NID1 | 0.408 | 4.70E-22 |
| AC007879.7 | SSH1 | 0.408 | 4.74E-22 |
| AC007879.7 | CDKL3 | 0.408 | 4.88E-22 |
| AC007879.7 | KCNJ1 | 0.408 | 4.97E-22 |
| AC007879.7 | ABCA6 | 0.408 | 5.01E-22 |
| AC007879.7 | CDH5 | 0.408 | 5.11E-22 |
| AC007879.7 | PTHLH | 0.408 | 5.30E-22 |
| AC007879.7 | ARRDC5 | 0.408 | 5.34E-22 |
| AC007879.7 | TDRD6 | 0.409 | 3.41E-22 |
| AC007879.7 | HIVEP2 | 0.409 | 3.42E-22 |
| AC007879.7 | TPM4 | 0.409 | 3.55E-22 |
| AC007879.7 | SERPINB9 | 0.409 | 3.65E-22 |
| AC007879.7 | PDE4B | 0.409 | 3.83E-22 |
| AC007879.7 | CYBB | 0.409 | 3.92E-22 |
| AC007879.7 | TWSG1 | 0.409 | 4.05E-22 |
| AC007879.7 | FHAD1 | 0.409 | 4.24E-22 |
| AC007879.7 | ABCA1 | 0.409 | 4.30E-22 |
| AC007879.7 | ADAM17 | 0.41 | 2.65E-22 |
| AC007879.7 | CXCR1 | 0.41 | 2.67E-22 |
| AC007879.7 | SYDE1 | 0.41 | 2.77E-22 |
| AC007879.7 | CLEC1B | 0.41 | 2.85E-22 |
| AC007879.7 | HK3 | 0.41 | 2.88E-22 |
| AC007879.7 | LINGO2 | 0.41 | 2.90E-22 |
| AC007879.7 | PCDH9 | 0.41 | 3.03E-22 |
| AC007879.7 | PRR34 | 0.41 | 3.06E-22 |
| AC007879.7 | RNF144B | 0.41 | 3.09E-22 |
| AC007879.7 | MRO | 0.41 | 3.10E-22 |
| AC007879.7 | PTRF | 0.41 | 3.23E-22 |
| AC007879.7 | MAP3K7CL | 0.41 | 3.26E-22 |
| AC007879.7 | SLC16A7 | 0.41 | 3.33E-22 |
| AC007879.7 | FAM155A | 0.41 | 3.34E-22 |
| AC007879.7 | TBC1D19 | 0.411 | 2.03E-22 |
| AC007879.7 | ARHGEF17 | 0.411 | 2.06E-22 |
| AC007879.7 | RASSF2 | 0.411 | 2.09E-22 |
| AC007879.7 | MFAP5 | 0.411 | 2.12E-22 |
| AC007879.7 | IL7R | 0.411 | 2.12E-22 |
| AC007879.7 | CAMK2A | 0.411 | 2.21E-22 |
| AC007879.7 | MACF1 | 0.411 | 2.26E-22 |
| AC007879.7 | B3GALNT1 | 0.411 | 2.32E-22 |
| AC007879.7 | HGF | 0.411 | 2.32E-22 |
| AC007879.7 | AXL | 0.411 | 2.55E-22 |
| AC007879.7 | PTGER3 | 0.412 | 1.64E-22 |
| AC007879.7 | C12orf54 | 0.412 | 1.74E-22 |
| AC007879.7 | CETP | 0.412 | 1.75E-22 |
| AC007879.7 | NUDT10 | 0.412 | 1.78E-22 |
| AC007879.7 | PCDHB5 | 0.412 | 1.80E-22 |
| AC007879.7 | ETV1 | 0.412 | 1.83E-22 |
| AC007879.7 | DNAJC5B | 0.412 | 1.85E-22 |
| AC007879.7 | PZP | 0.412 | 1.86E-22 |
| AC007879.7 | PCNX | 0.412 | 1.90E-22 |
| AC007879.7 | GAB3 | 0.412 | 1.92E-22 |
| AC007879.7 | ASAP1 | 0.412 | 1.94E-22 |
| AC007879.7 | TREM2 | 0.412 | 1.97E-22 |
| AC007879.7 | CCL3L3 | 0.413 | 1.25E-22 |
| AC007879.7 | CXCL6 | 0.413 | 1.26E-22 |
| AC007879.7 | CTD-3088G3.8 | 0.413 | 1.32E-22 |
| AC007879.7 | RECK | 0.413 | 1.36E-22 |
| AC007879.7 | ZBED2 | 0.413 | 1.39E-22 |
| AC007879.7 | PALLD | 0.413 | 1.46E-22 |
| AC007879.7 | CD226 | 0.413 | 1.52E-22 |
| AC007879.7 | TUBB6 | 0.414 | 9.66E-23 |
| AC007879.7 | LBH | 0.414 | 1.01E-22 |
| AC007879.7 | DCN | 0.414 | 1.04E-22 |
| AC007879.7 | ZNF267 | 0.414 | 1.05E-22 |
| AC007879.7 | PTPN12 | 0.414 | 1.07E-22 |
| AC007879.7 | FAM198B | 0.415 | 7.50E-23 |
| AC007879.7 | GBP5 | 0.415 | 7.75E-23 |
| AC007879.7 | KLF17 | 0.415 | 7.89E-23 |
| AC007879.7 | CNRIP1 | 0.415 | 8.09E-23 |
| AC007879.7 | AMIGO2 | 0.415 | 8.13E-23 |
| AC007879.7 | ARHGEF15 | 0.415 | 8.21E-23 |
| AC007879.7 | MICU3 | 0.415 | 8.28E-23 |
| AC007879.7 | TANC2 | 0.415 | 8.62E-23 |
| AC007879.7 | SPTB | 0.415 | 8.98E-23 |
| AC007879.7 | KDR | 0.415 | 9.11E-23 |
| AC007879.7 | CMYA5 | 0.415 | 9.15E-23 |
| AC007879.7 | FBXL2 | 0.415 | 9.19E-23 |
| AC007879.7 | C10orf90 | 0.416 | 5.56E-23 |
| AC007879.7 | TMEM136 | 0.416 | 6.23E-23 |
| AC007879.7 | AAED1 | 0.416 | 6.39E-23 |
| AC007879.7 | TIE1 | 0.416 | 6.42E-23 |
| AC007879.7 | CFAP61 | 0.416 | 6.57E-23 |
| AC007879.7 | NEMP2 | 0.416 | 6.81E-23 |
| AC007879.7 | PFKFB3 | 0.416 | 6.83E-23 |
| AC007879.7 | DISC1 | 0.416 | 6.86E-23 |
| AC007879.7 | ACVR1 | 0.416 | 7.00E-23 |
| AC007879.7 | TGFB2 | 0.416 | 7.13E-23 |
| AC007879.7 | COL6A1 | 0.417 | 4.25E-23 |
| AC007879.7 | GIT2 | 0.417 | 4.56E-23 |
| AC007879.7 | ANGPT4 | 0.417 | 4.83E-23 |
| AC007879.7 | MAS1 | 0.417 | 4.90E-23 |
| AC007879.7 | COL13A1 | 0.417 | 5.06E-23 |
| AC007879.7 | TMX3 | 0.417 | 5.21E-23 |
| AC007879.7 | RETN | 0.418 | 3.42E-23 |
| AC007879.7 | CHI3L2 | 0.418 | 3.60E-23 |
| AC007879.7 | FAM127C | 0.418 | 3.67E-23 |
| AC007879.7 | MS4A7 | 0.418 | 3.68E-23 |
| AC007879.7 | HTR1B | 0.418 | 3.71E-23 |
| AC007879.7 | CSDC2 | 0.418 | 3.90E-23 |
| AC007879.7 | DLG4 | 0.418 | 4.10E-23 |
| AC007879.7 | FAM180A | 0.418 | 4.15E-23 |
| AC007879.7 | MYO16 | 0.418 | 4.21E-23 |
| AC007879.7 | ADAMTSL4 | 0.419 | 2.74E-23 |
| AC007879.7 | STAC | 0.419 | 2.83E-23 |
| AC007879.7 | COL25A1 | 0.419 | 2.86E-23 |
| AC007879.7 | UCN2 | 0.419 | 2.88E-23 |
| AC007879.7 | TNFRSF9 | 0.419 | 2.91E-23 |
| AC007879.7 | S100A12 | 0.419 | 2.93E-23 |
| AC007879.7 | MOXD1 | 0.419 | 2.93E-23 |
| AC007879.7 | SSC5D | 0.419 | 2.95E-23 |
| AC007879.7 | HIF1A | 0.419 | 3.27E-23 |
| AC007879.7 | LYST | 0.42 | 2.01E-23 |
| AC007879.7 | G0S2 | 0.42 | 2.08E-23 |
| AC007879.7 | DCBLD2 | 0.42 | 2.10E-23 |
| AC007879.7 | LTBP1 | 0.42 | 2.11E-23 |
| AC007879.7 | SLC6A16 | 0.42 | 2.11E-23 |
| AC007879.7 | KCNA7 | 0.42 | 2.12E-23 |
| AC007879.7 | PXDNL | 0.42 | 2.12E-23 |
| AC007879.7 | LAPTM5 | 0.42 | 2.13E-23 |
| AC007879.7 | IL1RN | 0.42 | 2.25E-23 |
| AC007879.7 | CDC42SE1 | 0.42 | 2.48E-23 |
| AC007879.7 | ARMCX2 | 0.421 | 1.61E-23 |
| AC007879.7 | MRC1 | 0.421 | 1.63E-23 |
| AC007879.7 | EMILIN2 | 0.421 | 1.72E-23 |
| AC007879.7 | TNFSF8 | 0.421 | 1.76E-23 |
| AC007879.7 | AMPD3 | 0.421 | 1.78E-23 |
| AC007879.7 | PLEKHO2 | 0.421 | 1.80E-23 |
| AC007879.7 | DOCK10 | 0.421 | 1.81E-23 |
| AC007879.7 | F2R | 0.421 | 1.85E-23 |
| AC007879.7 | CAMSAP2 | 0.421 | 1.87E-23 |
| AC007879.7 | GPR161 | 0.421 | 1.89E-23 |
| AC007879.7 | ADGRE3 | 0.421 | 1.93E-23 |
| AC007879.7 | TM4SF18 | 0.422 | 1.28E-23 |
| AC007879.7 | CYTL1 | 0.422 | 1.30E-23 |
| AC007879.7 | SLC24A2 | 0.422 | 1.36E-23 |
| AC007879.7 | TMEM47 | 0.422 | 1.38E-23 |
| AC007879.7 | TMEM26 | 0.422 | 1.38E-23 |
| AC007879.7 | LAIR1 | 0.422 | 1.43E-23 |
| AC007879.7 | TUSC3 | 0.422 | 1.46E-23 |
| AC007879.7 | FIGN | 0.422 | 1.48E-23 |
| AC007879.7 | CCL3 | 0.423 | 8.76E-24 |
| AC007879.7 | GREB1 | 0.423 | 9.11E-24 |
| AC007879.7 | SH3PXD2B | 0.423 | 9.29E-24 |
| AC007879.7 | PCDHGA9 | 0.423 | 9.35E-24 |
| AC007879.7 | PMP22 | 0.423 | 1.02E-23 |
| AC007879.7 | ITGA1 | 0.423 | 1.03E-23 |
| AC007879.7 | CLEC12B | 0.423 | 1.12E-23 |
| AC007879.7 | NBPF19 | 0.424 | 6.79E-24 |
| AC007879.7 | AGPAT4 | 0.424 | 6.80E-24 |
| AC007879.7 | C6orf10 | 0.424 | 7.21E-24 |
| AC007879.7 | GYPE | 0.424 | 7.64E-24 |
| AC007879.7 | EID3 | 0.424 | 7.68E-24 |
| AC007879.7 | TFPI | 0.424 | 7.73E-24 |
| AC007879.7 | CD33 | 0.424 | 7.87E-24 |
| AC007879.7 | AOC2 | 0.424 | 8.04E-24 |
| AC007879.7 | STARD8 | 0.424 | 8.35E-24 |
| AC007879.7 | FRMD4A | 0.424 | 8.42E-24 |
| AC007879.7 | LY96 | 0.424 | 8.42E-24 |
| AC007879.7 | VCAM1 | 0.424 | 8.53E-24 |
| AC007879.7 | PRICKLE2 | 0.425 | 5.18E-24 |
| AC007879.7 | WNT2 | 0.425 | 5.37E-24 |
| AC007879.7 | COL4A2 | 0.425 | 5.73E-24 |
| AC007879.7 | IMPG2 | 0.425 | 5.77E-24 |
| AC007879.7 | RIMKLB | 0.425 | 5.84E-24 |
| AC007879.7 | FLI1 | 0.425 | 6.37E-24 |
| AC007879.7 | CCR8 | 0.425 | 6.37E-24 |
| AC007879.7 | SOGA3 | 0.425 | 6.48E-24 |
| AC007879.7 | CEP85L | 0.426 | 4.05E-24 |
| AC007879.7 | TRPC3 | 0.426 | 4.17E-24 |
| AC007879.7 | C1S | 0.426 | 4.25E-24 |
| AC007879.7 | KDELC1 | 0.426 | 4.32E-24 |
| AC007879.7 | MAP4K4 | 0.426 | 4.41E-24 |
| AC007879.7 | C1QTNF6 | 0.426 | 4.47E-24 |
| AC007879.7 | SACS | 0.426 | 4.51E-24 |
| AC007879.7 | CA5B | 0.426 | 4.66E-24 |
| AC007879.7 | KATNAL1 | 0.426 | 4.70E-24 |
| AC007879.7 | CSRP2 | 0.426 | 4.71E-24 |
| AC007879.7 | PEA15 | 0.426 | 4.84E-24 |
| AC007879.7 | CREB5 | 0.427 | 3.03E-24 |
| AC007879.7 | RORA | 0.427 | 3.05E-24 |
| AC007879.7 | EFS | 0.427 | 3.07E-24 |
| AC007879.7 | LURAP1 | 0.427 | 3.14E-24 |
| AC007879.7 | PANX1 | 0.427 | 3.31E-24 |
| AC007879.7 | SERPINB2 | 0.427 | 3.33E-24 |
| AC007879.7 | MILR1 | 0.427 | 3.58E-24 |
| AC007879.7 | MDFIC | 0.428 | 2.33E-24 |
| AC007879.7 | MROH9 | 0.428 | 2.64E-24 |
| AC007879.7 | ANXA1 | 0.428 | 2.70E-24 |
| AC007879.7 | PLEK | 0.428 | 2.72E-24 |
| AC007879.7 | NREP | 0.429 | 1.76E-24 |
| AC007879.7 | CLEC4A | 0.429 | 1.77E-24 |
| AC007879.7 | PRAM1 | 0.429 | 1.79E-24 |
| AC007879.7 | BICD1 | 0.429 | 1.87E-24 |
| AC007879.7 | MYCT1 | 0.429 | 1.91E-24 |
| AC007879.7 | AC069368.3 | 0.429 | 1.94E-24 |
| AC007879.7 | COMP | 0.429 | 1.95E-24 |
| AC007879.7 | COLEC12 | 0.429 | 1.96E-24 |
| AC007879.7 | NEXN | 0.429 | 1.97E-24 |
| AC007879.7 | TECTA | 0.429 | 1.99E-24 |
| AC007879.7 | NAIP | 0.43 | 1.33E-24 |
| AC007879.7 | GPR4 | 0.43 | 1.46E-24 |
| AC007879.7 | SLFN12 | 0.43 | 1.56E-24 |
| AC007879.7 | SLC16A6 | 0.43 | 1.68E-24 |
| AC007879.7 | HEYL | 0.431 | 1.10E-24 |
| AC007879.7 | PDGFB | 0.431 | 1.13E-24 |
| AC007879.7 | EOGT | 0.431 | 1.16E-24 |
| AC007879.7 | CHST3 | 0.431 | 1.25E-24 |
| AC007879.7 | APBA2 | 0.431 | 1.28E-24 |
| AC007879.7 | SLAMF8 | 0.432 | 8.56E-25 |
| AC007879.7 | FLG | 0.432 | 8.70E-25 |
| AC007879.7 | DIO2 | 0.432 | 8.72E-25 |
| AC007879.7 | TRPV4 | 0.432 | 8.75E-25 |
| AC007879.7 | CXCL8 | 0.432 | 9.11E-25 |
| AC007879.7 | NETO1 | 0.432 | 9.23E-25 |
| AC007879.7 | ALPL | 0.432 | 9.38E-25 |
| AC007879.7 | CLEC6A | 0.432 | 9.43E-25 |
| AC007879.7 | CNIH3 | 0.433 | 5.88E-25 |
| AC007879.7 | HECTD2 | 0.433 | 6.04E-25 |
| AC007879.7 | ARHGAP24 | 0.433 | 6.50E-25 |
| AC007879.7 | ZNF626 | 0.433 | 6.91E-25 |
| AC007879.7 | P3H1 | 0.433 | 7.12E-25 |
| AC007879.7 | ATP8B2 | 0.433 | 7.15E-25 |
| AC007879.7 | HCAR2 | 0.433 | 7.24E-25 |
| AC007879.7 | ETS1 | 0.433 | 7.32E-25 |
| AC007879.7 | RAB7B | 0.433 | 7.47E-25 |
| AC007879.7 | LRRC70 | 0.433 | 7.50E-25 |
| AC007879.7 | NOVA2 | 0.434 | 4.62E-25 |
| AC007879.7 | RIC1 | 0.434 | 5.05E-25 |
| AC007879.7 | ADAMTS14 | 0.434 | 5.14E-25 |
| AC007879.7 | SMARCA1 | 0.434 | 5.26E-25 |
| AC007879.7 | TLR1 | 0.434 | 5.44E-25 |
| AC007879.7 | KLRG1 | 0.434 | 5.59E-25 |
| AC007879.7 | CR1 | 0.434 | 5.76E-25 |
| AC007879.7 | GPR182 | 0.435 | 3.38E-25 |
| AC007879.7 | C3AR1 | 0.435 | 3.44E-25 |
| AC007879.7 | ADAM19 | 0.435 | 3.54E-25 |
| AC007879.7 | SLAMF9 | 0.435 | 3.64E-25 |
| AC007879.7 | FAM171B | 0.435 | 3.88E-25 |
| AC007879.7 | ZNF677 | 0.435 | 3.98E-25 |
| AC007879.7 | GPNMB | 0.435 | 4.34E-25 |
| AC007879.7 | SIX4 | 0.436 | 2.60E-25 |
| AC007879.7 | MCF2L2 | 0.436 | 2.67E-25 |
| AC007879.7 | C1orf101 | 0.436 | 2.68E-25 |
| AC007879.7 | OSM | 0.436 | 2.78E-25 |
| AC007879.7 | DCBLD1 | 0.436 | 2.80E-25 |
| AC007879.7 | NCAM2 | 0.436 | 2.82E-25 |
| AC007879.7 | MEIS1 | 0.436 | 3.01E-25 |
| AC007879.7 | HTR2B | 0.436 | 3.14E-25 |
| AC007879.7 | BNC1 | 0.436 | 3.19E-25 |
| AC007879.7 | GUCY1A2 | 0.436 | 3.21E-25 |
| AC007879.7 | ST8SIA4 | 0.437 | 2.00E-25 |
| AC007879.7 | PALM2 | 0.437 | 2.09E-25 |
| AC007879.7 | SDK1 | 0.437 | 2.13E-25 |
| AC007879.7 | MSRB3 | 0.437 | 2.33E-25 |
| AC007879.7 | RAB8B | 0.437 | 2.40E-25 |
| AC007879.7 | JAZF1 | 0.437 | 2.43E-25 |
| AC007879.7 | C3orf80 | 0.437 | 2.45E-25 |
| AC007879.7 | ZNF454 | 0.437 | 2.47E-25 |
| AC007879.7 | PDE1B | 0.438 | 1.46E-25 |
| AC007879.7 | ITGA10 | 0.438 | 1.48E-25 |
| AC007879.7 | SHANK1 | 0.438 | 1.53E-25 |
| AC007879.7 | NOTCH3 | 0.438 | 1.68E-25 |
| AC007879.7 | CATIP | 0.438 | 1.79E-25 |
| AC007879.7 | CEACAM4 | 0.438 | 1.81E-25 |
| AC007879.7 | MARCO | 0.438 | 1.84E-25 |
| AC007879.7 | FAM151B | 0.438 | 1.91E-25 |
| AC007879.7 | SLC35G2 | 0.439 | 1.17E-25 |
| AC007879.7 | SERPINF1 | 0.439 | 1.22E-25 |
| AC007879.7 | CNTN6 | 0.439 | 1.32E-25 |
| AC007879.7 | ABL2 | 0.44 | 8.57E-26 |
| AC007879.7 | MSN | 0.44 | 8.72E-26 |
| AC007879.7 | GEM | 0.44 | 8.89E-26 |
| AC007879.7 | LRRC4C | 0.44 | 8.89E-26 |
| AC007879.7 | HAVCR2 | 0.44 | 9.73E-26 |
| AC007879.7 | C11orf88 | 0.44 | 9.83E-26 |
| AC007879.7 | GJC1 | 0.44 | 1.01E-25 |
| AC007879.7 | FPR3 | 0.44 | 1.04E-25 |
| AC007879.7 | RUNX1T1 | 0.441 | 6.73E-26 |
| AC007879.7 | SIRPB1 | 0.441 | 6.73E-26 |
| AC007879.7 | MCF2 | 0.441 | 7.13E-26 |
| AC007879.7 | DLEU7 | 0.441 | 7.44E-26 |
| AC007879.7 | MSC | 0.442 | 4.83E-26 |
| AC007879.7 | LILRA1 | 0.442 | 4.83E-26 |
| AC007879.7 | TEX14 | 0.442 | 5.07E-26 |
| AC007879.7 | NLRP1 | 0.442 | 5.21E-26 |
| AC007879.7 | CARD8 | 0.442 | 5.26E-26 |
| AC007879.7 | CD163 | 0.442 | 5.31E-26 |
| AC007879.7 | LAMC1 | 0.442 | 6.07E-26 |
| AC007879.7 | RORB | 0.443 | 3.69E-26 |
| AC007879.7 | PCDHGA4 | 0.443 | 3.74E-26 |
| AC007879.7 | IL6 | 0.443 | 4.36E-26 |
| AC007879.7 | SOD2 | 0.443 | 4.53E-26 |
| AC007879.7 | ITGB3 | 0.444 | 2.90E-26 |
| AC007879.7 | SLIT2 | 0.444 | 2.98E-26 |
| AC007879.7 | ADAMTS9 | 0.444 | 3.21E-26 |
| AC007879.7 | CDYL2 | 0.444 | 3.29E-26 |
| AC007879.7 | OPN1SW | 0.445 | 2.09E-26 |
| AC007879.7 | CD80 | 0.445 | 2.12E-26 |
| AC007879.7 | ITGB1 | 0.445 | 2.22E-26 |
| AC007879.7 | FGF5 | 0.445 | 2.35E-26 |
| AC007879.7 | CCL2 | 0.445 | 2.55E-26 |
| AC007879.7 | THEMIS2 | 0.446 | 1.69E-26 |
| AC007879.7 | CLIP4 | 0.446 | 1.70E-26 |
| AC007879.7 | DCSTAMP | 0.446 | 1.91E-26 |
| AC007879.7 | PROK2 | 0.446 | 1.92E-26 |
| AC007879.7 | EBF1 | 0.447 | 1.23E-26 |
| AC007879.7 | SPG20 | 0.447 | 1.26E-26 |
| AC007879.7 | KCNT2 | 0.447 | 1.26E-26 |
| AC007879.7 | PIK3R5 | 0.447 | 1.29E-26 |
| AC007879.7 | PABPC5 | 0.447 | 1.42E-26 |
| AC007879.7 | AMPH | 0.447 | 1.45E-26 |
| AC007879.7 | DYNC2H1 | 0.447 | 1.48E-26 |
| AC007879.7 | YPEL4 | 0.448 | 9.02E-27 |
| AC007879.7 | DKK3 | 0.448 | 9.31E-27 |
| AC007879.7 | DYSF | 0.448 | 9.59E-27 |
| AC007879.7 | PI15 | 0.448 | 9.87E-27 |
| AC007879.7 | ARHGAP29 | 0.448 | 1.05E-26 |
| AC007879.7 | SLITRK4 | 0.448 | 1.10E-26 |
| AC007879.7 | FST | 0.449 | 7.53E-27 |
| AC007879.7 | RANBP3L | 0.449 | 7.53E-27 |
| AC007879.7 | TNR | 0.449 | 8.15E-27 |
| AC007879.7 | ENPEP | 0.45 | 4.77E-27 |
| AC007879.7 | SOCS3 | 0.45 | 5.41E-27 |
| AC007879.7 | PIP4K2A | 0.451 | 3.61E-27 |
| AC007879.7 | XIRP1 | 0.451 | 3.62E-27 |
| AC007879.7 | COL7A1 | 0.451 | 3.74E-27 |
| AC007879.7 | ADAMTS10 | 0.451 | 4.06E-27 |
| AC007879.7 | PECAM1 | 0.451 | 4.09E-27 |
| AC007879.7 | GGT5 | 0.451 | 4.21E-27 |
| AC007879.7 | PADI4 | 0.451 | 4.41E-27 |
| AC007879.7 | THBD | 0.451 | 4.50E-27 |
| AC007879.7 | FZD4 | 0.451 | 4.50E-27 |
| AC007879.7 | SRGN | 0.451 | 4.59E-27 |
| AC007879.7 | EGR2 | 0.452 | 2.79E-27 |
| AC007879.7 | RFTN1 | 0.452 | 3.23E-27 |
| AC007879.7 | AMOTL1 | 0.452 | 3.25E-27 |
| AC007879.7 | TPRG1 | 0.452 | 3.28E-27 |
| AC007879.7 | SYT11 | 0.452 | 3.49E-27 |
| AC007879.7 | PDE10A | 0.453 | 2.04E-27 |
| AC007879.7 | LILRA2 | 0.453 | 2.30E-27 |
| AC007879.7 | RASSF8 | 0.453 | 2.39E-27 |
| AC007879.7 | SAMSN1 | 0.453 | 2.51E-27 |
| AC007879.7 | C20orf194 | 0.454 | 1.59E-27 |
| AC007879.7 | LILRB2 | 0.454 | 1.61E-27 |
| AC007879.7 | COL16A1 | 0.454 | 1.64E-27 |
| AC007879.7 | PRPH2 | 0.454 | 1.67E-27 |
| AC007879.7 | ALOX5AP | 0.454 | 1.73E-27 |
| AC007879.7 | IGLON5 | 0.454 | 1.81E-27 |
| AC007879.7 | VSTM4 | 0.454 | 1.81E-27 |
| AC007879.7 | TSPYL5 | 0.454 | 1.82E-27 |
| AC007879.7 | GJA5 | 0.454 | 1.85E-27 |
| AC007879.7 | C10orf55 | 0.454 | 1.90E-27 |
| AC007879.7 | IBSP | 0.454 | 1.92E-27 |
| AC007879.7 | S100A8 | 0.455 | 1.10E-27 |
| AC007879.7 | EVI2A | 0.455 | 1.11E-27 |
| AC007879.7 | RGS16 | 0.455 | 1.11E-27 |
| AC007879.7 | AJAP1 | 0.455 | 1.11E-27 |
| AC007879.7 | SPTA1 | 0.455 | 1.18E-27 |
| AC007879.7 | SLC35B4 | 0.455 | 1.19E-27 |
| AC007879.7 | CYP19A1 | 0.455 | 1.24E-27 |
| AC007879.7 | TNFSF13B | 0.455 | 1.27E-27 |
| AC007879.7 | NAV1 | 0.455 | 1.32E-27 |
| AC007879.7 | SIRPD | 0.455 | 1.43E-27 |
| AC007879.7 | DCUN1D3 | 0.456 | 8.77E-28 |
| AC007879.7 | EYA4 | 0.456 | 9.02E-28 |
| AC007879.7 | ENOX1 | 0.456 | 9.36E-28 |
| AC007879.7 | RNF180 | 0.456 | 1.03E-27 |
| AC007879.7 | SYNE1 | 0.456 | 1.07E-27 |
| AC007879.7 | ZEB1 | 0.457 | 6.17E-28 |
| AC007879.7 | WWTR1 | 0.457 | 7.80E-28 |
| AC007879.7 | WDFY2 | 0.458 | 4.44E-28 |
| AC007879.7 | LILRB4 | 0.458 | 4.52E-28 |
| AC007879.7 | SFRP2 | 0.458 | 4.57E-28 |
| AC007879.7 | VASH1 | 0.458 | 4.72E-28 |
| AC007879.7 | SCGB3A2 | 0.458 | 4.93E-28 |
| AC007879.7 | ELOVL4 | 0.458 | 5.43E-28 |
| AC007879.7 | FKBP7 | 0.458 | 5.52E-28 |
| AC007879.7 | RGS17 | 0.458 | 5.53E-28 |
| AC007879.7 | MAP1A | 0.458 | 5.55E-28 |
| AC007879.7 | NOTCH4 | 0.458 | 5.80E-28 |
| AC007879.7 | ATXN1 | 0.459 | 3.30E-28 |
| AC007879.7 | SLC38A6 | 0.459 | 3.47E-28 |
| AC007879.7 | KCNQ3 | 0.459 | 3.81E-28 |
| AC007879.7 | CABYR | 0.459 | 3.99E-28 |
| AC007879.7 | CLEC2B | 0.46 | 2.52E-28 |
| AC007879.7 | SPHK1 | 0.46 | 2.60E-28 |
| AC007879.7 | ZNF25 | 0.46 | 2.75E-28 |
| AC007879.7 | FAM63B | 0.46 | 2.78E-28 |
| AC007879.7 | SFRP4 | 0.46 | 2.80E-28 |
| AC007879.7 | CD86 | 0.46 | 2.89E-28 |
| AC007879.7 | CFH | 0.46 | 3.09E-28 |
| AC007879.7 | C8orf34 | 0.461 | 1.79E-28 |
| AC007879.7 | FBXL7 | 0.461 | 1.88E-28 |
| AC007879.7 | CAV2 | 0.461 | 2.03E-28 |
| AC007879.7 | MAFB | 0.461 | 2.15E-28 |
| AC007879.7 | TICAM2 | 0.461 | 2.15E-28 |
| AC007879.7 | PDGFRL | 0.461 | 2.19E-28 |
| AC007879.7 | C9orf47 | 0.461 | 2.22E-28 |
| AC007879.7 | PCDH7 | 0.461 | 2.33E-28 |
| AC007879.7 | GPR68 | 0.461 | 2.39E-28 |
| AC007879.7 | CORIN | 0.462 | 1.32E-28 |
| AC007879.7 | HOPX | 0.462 | 1.49E-28 |
| AC007879.7 | MAGEL2 | 0.462 | 1.55E-28 |
| AC007879.7 | IGFL3 | 0.462 | 1.60E-28 |
| AC007879.7 | CLIC4 | 0.462 | 1.66E-28 |
| AC007879.7 | MRC2 | 0.462 | 1.68E-28 |
| AC007879.7 | IGFBP5 | 0.462 | 1.73E-28 |
| AC007879.7 | EHD3 | 0.463 | 1.00E-28 |
| AC007879.7 | TM4SF19 | 0.463 | 1.09E-28 |
| AC007879.7 | ATP8B4 | 0.463 | 1.12E-28 |
| AC007879.7 | CDH6 | 0.463 | 1.25E-28 |
| AC007879.7 | MEOX2 | 0.463 | 1.26E-28 |
| AC007879.7 | TFEC | 0.463 | 1.30E-28 |
| AC007879.7 | TRPC1 | 0.464 | 7.73E-29 |
| AC007879.7 | SYNDIG1 | 0.464 | 8.45E-29 |
| AC007879.7 | SIGLEC9 | 0.465 | 6.06E-29 |
| AC007879.7 | SLFN11 | 0.465 | 6.06E-29 |
| AC007879.7 | SAMD4A | 0.465 | 6.61E-29 |
| AC007879.7 | PLEKHG2 | 0.466 | 4.30E-29 |
| AC007879.7 | 4-Sep | 0.466 | 4.69E-29 |
| AC007879.7 | CDH13 | 0.466 | 4.69E-29 |
| AC007879.7 | OSCAR | 0.466 | 4.77E-29 |
| AC007879.7 | SBSN | 0.466 | 5.07E-29 |
| AC007879.7 | HOOK3 | 0.466 | 5.26E-29 |
| AC007879.7 | LIMS1 | 0.467 | 3.49E-29 |
| AC007879.7 | C10orf10 | 0.468 | 2.11E-29 |
| AC007879.7 | PURG | 0.468 | 2.23E-29 |
| AC007879.7 | CACNA2D1 | 0.468 | 2.24E-29 |
| AC007879.7 | AF165138.7 | 0.468 | 2.25E-29 |
| AC007879.7 | RARB | 0.468 | 2.46E-29 |
| AC007879.7 | SFMBT2 | 0.468 | 2.50E-29 |
| AC007879.7 | GLI2 | 0.468 | 2.53E-29 |
| AC007879.7 | FAM101B | 0.468 | 2.63E-29 |
| AC007879.7 | CFAP54 | 0.468 | 2.74E-29 |
| AC007879.7 | ADGRL2 | 0.468 | 2.79E-29 |
| AC007879.7 | ZNF385D | 0.469 | 1.72E-29 |
| AC007879.7 | CXorf36 | 0.469 | 1.75E-29 |
| AC007879.7 | NME8 | 0.469 | 1.82E-29 |
| AC007879.7 | MEFV | 0.469 | 1.83E-29 |
| AC007879.7 | FYN | 0.469 | 1.95E-29 |
| AC007879.7 | FGR | 0.469 | 1.96E-29 |
| AC007879.7 | CTSK | 0.47 | 1.18E-29 |
| AC007879.7 | DPYSL3 | 0.47 | 1.24E-29 |
| AC007879.7 | ZFP92 | 0.47 | 1.25E-29 |
| AC007879.7 | ADGRG3 | 0.47 | 1.27E-29 |
| AC007879.7 | SCHIP1 | 0.47 | 1.32E-29 |
| AC007879.7 | SLC46A2 | 0.47 | 1.51E-29 |
| AC007879.7 | PPFIBP1 | 0.471 | 8.89E-30 |
| AC007879.7 | PHTF2 | 0.471 | 8.91E-30 |
| AC007879.7 | DENND5A | 0.471 | 9.59E-30 |
| AC007879.7 | COPZ2 | 0.471 | 1.07E-29 |
| AC007879.7 | NXN | 0.472 | 6.23E-30 |
| AC007879.7 | CCR1 | 0.472 | 6.89E-30 |
| AC007879.7 | RXFP1 | 0.472 | 7.28E-30 |
| AC007879.7 | WIPF1 | 0.472 | 7.46E-30 |
| AC007879.7 | CERCAM | 0.472 | 7.47E-30 |
| AC007879.7 | THBS1 | 0.472 | 7.77E-30 |
| AC007879.7 | LRRK2 | 0.472 | 8.08E-30 |
| AC007879.7 | MATN3 | 0.472 | 8.12E-30 |
| AC007879.7 | EGFLAM | 0.473 | 4.47E-30 |
| AC007879.7 | EPYC | 0.473 | 4.64E-30 |
| AC007879.7 | CASC10 | 0.473 | 4.76E-30 |
| AC007879.7 | PILRA | 0.473 | 4.86E-30 |
| AC007879.7 | CYP27C1 | 0.473 | 5.00E-30 |
| AC007879.7 | DLC1 | 0.473 | 5.25E-30 |
| AC007879.7 | COL8A2 | 0.473 | 5.82E-30 |
| AC007879.7 | FUT11 | 0.473 | 5.94E-30 |
| AC007879.7 | SRSF12 | 0.474 | 3.59E-30 |
| AC007879.7 | KIFC3 | 0.474 | 3.78E-30 |
| AC007879.7 | TGFBR1 | 0.475 | 2.52E-30 |
| AC007879.7 | KCNJ8 | 0.475 | 2.59E-30 |
| AC007879.7 | SLC6A17 | 0.475 | 2.65E-30 |
| AC007879.7 | MMP14 | 0.475 | 2.76E-30 |
| AC007879.7 | FAM49A | 0.475 | 2.78E-30 |
| AC007879.7 | CASS4 | 0.475 | 2.88E-30 |
| AC007879.7 | CLMP | 0.475 | 3.09E-30 |
| AC007879.7 | TRPC6 | 0.475 | 3.20E-30 |
| AC007879.7 | ERG | 0.476 | 1.93E-30 |
| AC007879.7 | 1-Mar | 0.477 | 1.31E-30 |
| AC007879.7 | DPY19L2 | 0.477 | 1.67E-30 |
| AC007879.7 | PPP1R18 | 0.478 | 1.20E-30 |
| AC007879.7 | ESR1 | 0.479 | 6.88E-31 |
| AC007879.7 | ARMC9 | 0.479 | 7.06E-31 |
| AC007879.7 | CREM | 0.479 | 7.53E-31 |
| AC007879.7 | RUNX1 | 0.479 | 7.61E-31 |
| AC007879.7 | FGF14 | 0.479 | 8.81E-31 |
| AC007879.7 | PHACTR1 | 0.48 | 4.77E-31 |
| AC007879.7 | THY1 | 0.48 | 4.94E-31 |
| AC007879.7 | FAT3 | 0.48 | 5.15E-31 |
| AC007879.7 | ZNF208 | 0.48 | 5.20E-31 |
| AC007879.7 | MNDA | 0.48 | 5.66E-31 |
| AC007879.7 | GALNT15 | 0.48 | 6.15E-31 |
| AC007879.7 | TRIM46 | 0.48 | 6.26E-31 |
| AC007879.7 | ITGAM | 0.48 | 6.44E-31 |
| AC007879.7 | AEBP1 | 0.481 | 3.64E-31 |
| AC007879.7 | KMO | 0.481 | 3.75E-31 |
| AC007879.7 | TLR6 | 0.481 | 3.77E-31 |
| AC007879.7 | GUCY1A3 | 0.481 | 3.86E-31 |
| AC007879.7 | RFTN2 | 0.481 | 3.94E-31 |
| AC007879.7 | FIBIN | 0.481 | 4.26E-31 |
| AC007879.7 | FLT1 | 0.481 | 4.60E-31 |
| AC007879.7 | CCIN | 0.481 | 4.70E-31 |
| AC007879.7 | STK32B | 0.482 | 2.58E-31 |
| AC007879.7 | MEX3B | 0.482 | 2.77E-31 |
| AC007879.7 | SDC2 | 0.482 | 2.77E-31 |
| AC007879.7 | FERMT2 | 0.482 | 2.83E-31 |
| AC007879.7 | DDR2 | 0.482 | 3.30E-31 |
| AC007879.7 | ARHGAP31 | 0.482 | 3.35E-31 |
| AC007879.7 | BCL6 | 0.483 | 1.85E-31 |
| AC007879.7 | RHOJ | 0.483 | 1.94E-31 |
| AC007879.7 | TPST1 | 0.483 | 1.99E-31 |
| AC007879.7 | CPT1C | 0.483 | 2.06E-31 |
| AC007879.7 | PREX2 | 0.483 | 2.13E-31 |
| AC007879.7 | LTBP2 | 0.483 | 2.15E-31 |
| AC007879.7 | ZCCHC5 | 0.483 | 2.20E-31 |
| AC007879.7 | FBLN7 | 0.483 | 2.48E-31 |
| AC007879.7 | CRISPLD1 | 0.484 | 1.37E-31 |
| AC007879.7 | IKBIP | 0.484 | 1.40E-31 |
| AC007879.7 | CHST15 | 0.484 | 1.47E-31 |
| AC007879.7 | TIMP2 | 0.484 | 1.58E-31 |
| AC007879.7 | CD109 | 0.484 | 1.65E-31 |
| AC007879.7 | PPEF1 | 0.485 | 9.65E-32 |
| AC007879.7 | SLA | 0.485 | 9.66E-32 |
| AC007879.7 | FCGR2B | 0.485 | 1.06E-31 |
| AC007879.7 | C5AR2 | 0.485 | 1.07E-31 |
| AC007879.7 | SELE | 0.485 | 1.09E-31 |
| AC007879.7 | BEST1 | 0.485 | 1.28E-31 |
| AC007879.7 | WDPCP | 0.486 | 6.84E-32 |
| AC007879.7 | ZNF281 | 0.486 | 8.77E-32 |
| AC007879.7 | PRND | 0.486 | 8.81E-32 |
| AC007879.7 | CALD1 | 0.487 | 4.96E-32 |
| AC007879.7 | ANGPT2 | 0.487 | 5.32E-32 |
| AC007879.7 | GDF6 | 0.487 | 5.33E-32 |
| AC007879.7 | FAM110B | 0.487 | 5.73E-32 |
| AC007879.7 | NRIP3 | 0.487 | 6.13E-32 |
| AC007879.7 | FMNL3 | 0.488 | 3.65E-32 |
| AC007879.7 | HTRA1 | 0.488 | 3.78E-32 |
| AC007879.7 | WWC2 | 0.488 | 4.03E-32 |
| AC007879.7 | DYRK3 | 0.488 | 4.42E-32 |
| AC007879.7 | SRGAP2C | 0.488 | 4.49E-32 |
| AC007879.7 | NCF2 | 0.489 | 2.77E-32 |
| AC007879.7 | MPDZ | 0.489 | 2.80E-32 |
| AC007879.7 | CCDC8 | 0.489 | 3.22E-32 |
| AC007879.7 | STON1 | 0.489 | 3.26E-32 |
| AC007879.7 | LCP2 | 0.49 | 2.26E-32 |
| AC007879.7 | TSHZ3 | 0.49 | 2.36E-32 |
| AC007879.7 | LOXL2 | 0.491 | 1.30E-32 |
| AC007879.7 | CCDC80 | 0.491 | 1.65E-32 |
| AC007879.7 | LHFP | 0.492 | 9.39E-33 |
| AC007879.7 | MAP1B | 0.492 | 1.05E-32 |
| AC007879.7 | P2RX7 | 0.492 | 1.25E-32 |
| AC007879.7 | TBX15 | 0.493 | 6.67E-33 |
| AC007879.7 | TREML4 | 0.493 | 7.38E-33 |
| AC007879.7 | MMP2 | 0.493 | 8.00E-33 |
| AC007879.7 | LRRC15 | 0.493 | 8.13E-33 |
| AC007879.7 | KIRREL | 0.493 | 8.89E-33 |
| AC007879.7 | WNK3 | 0.494 | 4.75E-33 |
| AC007879.7 | KLHL4 | 0.494 | 4.84E-33 |
| AC007879.7 | ZNF154 | 0.494 | 4.90E-33 |
| AC007879.7 | PIEZO2 | 0.494 | 5.12E-33 |
| AC007879.7 | KLRD1 | 0.494 | 5.33E-33 |
| AC007879.7 | MMP8 | 0.494 | 5.45E-33 |
| AC007879.7 | MCEMP1 | 0.494 | 5.81E-33 |
| AC007879.7 | FPR1 | 0.494 | 6.53E-33 |
| AC007879.7 | MURC | 0.495 | 3.46E-33 |
| AC007879.7 | PYGO1 | 0.495 | 3.51E-33 |
| AC007879.7 | PTPRM | 0.495 | 3.72E-33 |
| AC007879.7 | ANKRD6 | 0.495 | 3.75E-33 |
| AC007879.7 | LCA5 | 0.495 | 3.87E-33 |
| AC007879.7 | HSD11B1 | 0.495 | 4.08E-33 |
| AC007879.7 | GLIS3 | 0.495 | 4.10E-33 |
| AC007879.7 | ALDH1L2 | 0.495 | 4.19E-33 |
| AC007879.7 | TM6SF1 | 0.495 | 4.27E-33 |
| AC007879.7 | GUCY1B3 | 0.495 | 4.36E-33 |
| AC007879.7 | ITGAV | 0.495 | 4.61E-33 |
| AC007879.7 | PTGIR | 0.496 | 2.52E-33 |
| AC007879.7 | MS4A14 | 0.496 | 2.64E-33 |
| AC007879.7 | RGS4 | 0.496 | 2.93E-33 |
| AC007879.7 | CD84 | 0.496 | 3.24E-33 |
| AC007879.7 | LDB2 | 0.497 | 1.71E-33 |
| AC007879.7 | PCDH12 | 0.497 | 1.72E-33 |
| AC007879.7 | RASGRP3 | 0.497 | 1.77E-33 |
| AC007879.7 | DZIP1 | 0.497 | 1.92E-33 |
| AC007879.7 | IRAK3 | 0.497 | 2.04E-33 |
| AC007879.7 | DACT1 | 0.498 | 1.34E-33 |
| AC007879.7 | PRKD1 | 0.498 | 1.35E-33 |
| AC007879.7 | MYH10 | 0.498 | 1.70E-33 |
| AC007879.7 | ITGBL1 | 0.499 | 9.02E-34 |
| AC007879.7 | TMEM233 | 0.499 | 1.04E-33 |
| AC007879.7 | MITF | 0.499 | 1.08E-33 |
| AC007879.7 | EBF3 | 0.499 | 1.12E-33 |
| AC007879.7 | ARL4C | 0.5 | 7.45E-34 |
| AC007879.7 | FCGR1B | 0.5 | 8.02E-34 |
| AC007879.7 | MPP4 | 0.5 | 8.46E-34 |
| AC007879.7 | PLAU | 0.5 | 8.47E-34 |
| AC007879.7 | PHLDB2 | 0.501 | 4.40E-34 |
| AC007879.7 | UBE2E2 | 0.501 | 4.76E-34 |
| AC007879.7 | FAM124B | 0.501 | 5.26E-34 |
| AC007879.7 | SRGAP2 | 0.501 | 5.36E-34 |
| AC007879.7 | MCTP1 | 0.502 | 3.08E-34 |
| AC007879.7 | FNDC4 | 0.502 | 3.83E-34 |
| AC007879.7 | CHSY1 | 0.503 | 2.47E-34 |
| AC007879.7 | HS3ST3B1 | 0.503 | 2.98E-34 |
| AC007879.7 | MEIS3 | 0.504 | 1.57E-34 |
| AC007879.7 | FILIP1L | 0.504 | 1.99E-34 |
| AC007879.7 | KYNU | 0.504 | 2.02E-34 |
| AC007879.7 | TNC | 0.504 | 2.16E-34 |
| AC007879.7 | LAYN | 0.505 | 1.17E-34 |
| AC007879.7 | C1orf162 | 0.505 | 1.32E-34 |
| AC007879.7 | DOK6 | 0.505 | 1.36E-34 |
| AC007879.7 | RBMS1 | 0.506 | 8.41E-35 |
| AC007879.7 | CCL7 | 0.506 | 8.71E-35 |
| AC007879.7 | NID2 | 0.506 | 9.27E-35 |
| AC007879.7 | RHOQ | 0.506 | 9.54E-35 |
| AC007879.7 | FLRT2 | 0.506 | 9.89E-35 |
| AC007879.7 | STEAP1B | 0.507 | 5.57E-35 |
| AC007879.7 | CLEC7A | 0.508 | 4.20E-35 |
| AC007879.7 | BCL2A1 | 0.508 | 4.76E-35 |
| AC007879.7 | CYR61 | 0.508 | 4.93E-35 |
| AC007879.7 | RNASE2 | 0.508 | 5.20E-35 |
| AC007879.7 | MCHR1 | 0.509 | 2.75E-35 |
| AC007879.7 | C14orf37 | 0.509 | 2.84E-35 |
| AC007879.7 | CALCRL | 0.509 | 3.14E-35 |
| AC007879.7 | CSF3R | 0.509 | 3.18E-35 |
| AC007879.7 | HEG1 | 0.509 | 3.51E-35 |
| AC007879.7 | GRIA3 | 0.509 | 3.59E-35 |
| AC007879.7 | PALMD | 0.509 | 3.72E-35 |
| AC007879.7 | PLXDC1 | 0.51 | 2.05E-35 |
| AC007879.7 | KLHL5 | 0.51 | 2.13E-35 |
| AC007879.7 | LAMA4 | 0.51 | 2.29E-35 |
| AC007879.7 | LRRC8C | 0.511 | 1.30E-35 |
| AC007879.7 | BASP1 | 0.511 | 1.43E-35 |
| AC007879.7 | MAP3K12 | 0.511 | 1.53E-35 |
| AC007879.7 | GPR141 | 0.511 | 1.66E-35 |
| AC007879.7 | HMCN1 | 0.511 | 1.72E-35 |
| AC007879.7 | HHIPL1 | 0.511 | 1.81E-35 |
| AC007879.7 | COL5A3 | 0.512 | 9.18E-36 |
| AC007879.7 | GPR1 | 0.512 | 1.01E-35 |
| AC007879.7 | SGCD | 0.512 | 1.15E-35 |
| AC007879.7 | EDNRA | 0.512 | 1.28E-35 |
| AC007879.7 | PLXNC1 | 0.513 | 6.82E-36 |
| AC007879.7 | MMP13 | 0.513 | 7.98E-36 |
| AC007879.7 | S1PR3 | 0.513 | 7.98E-36 |
| AC007879.7 | ATP10A | 0.513 | 8.33E-36 |
| AC007879.7 | BCL6B | 0.514 | 4.75E-36 |
| AC007879.7 | CMTM3 | 0.514 | 4.87E-36 |
| AC007879.7 | GUCA1A | 0.515 | 3.80E-36 |
| AC007879.7 | MSR1 | 0.516 | 2.21E-36 |
| AC007879.7 | SGTB | 0.516 | 2.23E-36 |
| AC007879.7 | ZPLD1 | 0.516 | 2.45E-36 |
| AC007879.7 | KIAA1462 | 0.516 | 2.55E-36 |
| AC007879.7 | FCGR3A | 0.516 | 2.63E-36 |
| AC007879.7 | CFAP58 | 0.516 | 2.72E-36 |
| AC007879.7 | ANGPTL2 | 0.516 | 2.78E-36 |
| AC007879.7 | VIM | 0.516 | 3.04E-36 |
| AC007879.7 | MYO5A | 0.517 | 1.67E-36 |
| AC007879.7 | IL1R1 | 0.517 | 1.87E-36 |
| AC007879.7 | B4GALNT1 | 0.518 | 1.11E-36 |
| AC007879.7 | MEDAG | 0.519 | 7.61E-37 |
| AC007879.7 | GRM2 | 0.519 | 7.92E-37 |
| AC007879.7 | FCGR1A | 0.519 | 1.04E-36 |
| AC007879.7 | BEND6 | 0.52 | 5.27E-37 |
| AC007879.7 | ADGRL4 | 0.52 | 5.31E-37 |
| AC007879.7 | ITPRIP | 0.52 | 5.34E-37 |
| AC007879.7 | BGN | 0.52 | 6.54E-37 |
| AC007879.7 | PRICKLE1 | 0.521 | 3.47E-37 |
| AC007879.7 | NTNG2 | 0.521 | 3.82E-37 |
| AC007879.7 | CRISPLD2 | 0.521 | 4.10E-37 |
| AC007879.7 | PALM2-AKAP2 | 0.521 | 4.32E-37 |
| AC007879.7 | CD93 | 0.521 | 4.39E-37 |
| AC007879.7 | ASPN | 0.521 | 4.81E-37 |
| AC007879.7 | ZFPM2 | 0.522 | 3.06E-37 |
| AC007879.7 | PDCD1LG2 | 0.522 | 3.30E-37 |
| AC007879.7 | GPR84 | 0.523 | 1.68E-37 |
| AC007879.7 | TMEM45A | 0.523 | 2.04E-37 |
| AC007879.7 | SLC22A16 | 0.523 | 2.10E-37 |
| AC007879.7 | CLEC1A | 0.523 | 2.20E-37 |
| AC007879.7 | COL4A1 | 0.524 | 1.15E-37 |
| AC007879.7 | PKD2 | 0.524 | 1.49E-37 |
| AC007879.7 | NHSL2 | 0.524 | 1.61E-37 |
| AC007879.7 | PLXDC2 | 0.524 | 1.65E-37 |
| AC007879.7 | LOXL3 | 0.525 | 8.31E-38 |
| AC007879.7 | CHST1 | 0.525 | 8.37E-38 |
| AC007879.7 | KIF26B | 0.525 | 8.63E-38 |
| AC007879.7 | TMEM52B | 0.525 | 8.73E-38 |
| AC007879.7 | ZEB2 | 0.525 | 8.96E-38 |
| AC007879.7 | COL1A1 | 0.525 | 9.15E-38 |
| AC007879.7 | PLXNA4 | 0.525 | 9.61E-38 |
| AC007879.7 | ISM1 | 0.525 | 1.07E-37 |
| AC007879.7 | FAM126A | 0.526 | 6.71E-38 |
| AC007879.7 | GLT8D2 | 0.526 | 6.84E-38 |
| AC007879.7 | PDGFRB | 0.526 | 6.86E-38 |
| AC007879.7 | C5AR1 | 0.526 | 7.09E-38 |
| AC007879.7 | ADAMTS4 | 0.526 | 7.63E-38 |
| AC007879.7 | AKT3 | 0.527 | 4.20E-38 |
| AC007879.7 | SIGLEC5 | 0.527 | 4.39E-38 |
| AC007879.7 | EVC2 | 0.527 | 4.82E-38 |
| AC007879.7 | SRGAP2B | 0.528 | 2.57E-38 |
| AC007879.7 | NRK | 0.528 | 3.14E-38 |
| AC007879.7 | EVC | 0.528 | 3.25E-38 |
| AC007879.7 | GLI3 | 0.53 | 1.21E-38 |
| AC007879.7 | NUAK1 | 0.53 | 1.37E-38 |
| AC007879.7 | TCF4 | 0.53 | 1.61E-38 |
| AC007879.7 | FPR2 | 0.53 | 1.72E-38 |
| AC007879.7 | ZNF532 | 0.531 | 8.22E-39 |
| AC007879.7 | SPP1 | 0.531 | 9.73E-39 |
| AC007879.7 | LATS2 | 0.531 | 1.03E-38 |
| AC007879.7 | CALU | 0.532 | 7.28E-39 |
| AC007879.7 | DSEL | 0.532 | 7.46E-39 |
| AC007879.7 | CCDC81 | 0.532 | 7.46E-39 |
| AC007879.7 | IL1RAP | 0.533 | 3.97E-39 |
| AC007879.7 | PDGFC | 0.533 | 4.80E-39 |
| AC007879.7 | LUM | 0.534 | 2.98E-39 |
| AC007879.7 | DIRC1 | 0.534 | 3.17E-39 |
| AC007879.7 | STEAP4 | 0.534 | 3.29E-39 |
| AC007879.7 | CYP46A1 | 0.534 | 3.38E-39 |
| AC007879.7 | NLRC4 | 0.534 | 3.46E-39 |
| AC007879.7 | ADGRE2 | 0.534 | 3.61E-39 |
| AC007879.7 | CLEC4E | 0.535 | 2.51E-39 |
| AC007879.7 | FAM196B | 0.536 | 1.58E-39 |
| AC007879.7 | ZNF521 | 0.536 | 1.67E-39 |
| AC007879.7 | QKI | 0.537 | 8.42E-40 |
| AC007879.7 | SYNPO2L | 0.537 | 9.40E-40 |
| AC007879.7 | DOCK4 | 0.537 | 1.02E-39 |
| AC007879.7 | BNC2 | 0.537 | 1.13E-39 |
| AC007879.7 | LIPN | 0.538 | 5.98E-40 |
| AC007879.7 | TRO | 0.538 | 6.71E-40 |
| AC007879.7 | ARL10 | 0.538 | 7.31E-40 |
| AC007879.7 | GREM1 | 0.538 | 7.47E-40 |
| AC007879.7 | ZNF365 | 0.538 | 7.82E-40 |
| AC007879.7 | JAKMIP2 | 0.539 | 3.81E-40 |
| AC007879.7 | ENTPD1 | 0.539 | 4.72E-40 |
| AC007879.7 | GPC6 | 0.539 | 5.38E-40 |
| AC007879.7 | NRP2 | 0.541 | 1.76E-40 |
| AC007879.7 | GPX8 | 0.541 | 2.00E-40 |
| AC007879.7 | NNMT | 0.541 | 2.04E-40 |
| AC007879.7 | PDPN | 0.541 | 2.04E-40 |
| AC007879.7 | TSHZ2 | 0.541 | 2.11E-40 |
| AC007879.7 | SOX11 | 0.542 | 1.18E-40 |
| AC007879.7 | TWIST1 | 0.542 | 1.18E-40 |
| AC007879.7 | TRPS1 | 0.542 | 1.46E-40 |
| AC007879.7 | CEP170 | 0.543 | 9.09E-41 |
| AC007879.7 | ADAMTS2 | 0.543 | 9.35E-41 |
| AC007879.7 | ANOS1 | 0.543 | 1.03E-40 |
| AC007879.7 | RAI14 | 0.544 | 5.45E-41 |
| AC007879.7 | ELK3 | 0.544 | 5.65E-41 |
| AC007879.7 | ADAMTS16 | 0.544 | 5.93E-41 |
| AC007879.7 | MXRA5 | 0.544 | 7.13E-41 |
| AC007879.7 | DGKI | 0.544 | 7.65E-41 |
| AC007879.7 | FGF7 | 0.545 | 3.74E-41 |
| AC007879.7 | AMZ1 | 0.545 | 4.13E-41 |
| AC007879.7 | OLFML2B | 0.546 | 2.43E-41 |
| AC007879.7 | MOV10L1 | 0.547 | 1.55E-41 |
| AC007879.7 | TENM3 | 0.547 | 1.58E-41 |
| AC007879.7 | RUNX2 | 0.547 | 2.00E-41 |
| AC007879.7 | CCDC36 | 0.547 | 2.02E-41 |
| AC007879.7 | NLRP3 | 0.548 | 1.19E-41 |
| AC007879.7 | GJA1 | 0.548 | 1.23E-41 |
| AC007879.7 | ABCC9 | 0.548 | 1.41E-41 |
| AC007879.7 | PCDHGA12 | 0.548 | 1.49E-41 |
| AC007879.7 | GLT1D1 | 0.549 | 7.04E-42 |
| AC007879.7 | MLLT11 | 0.549 | 7.35E-42 |
| AC007879.7 | ZNF667 | 0.549 | 8.25E-42 |
| AC007879.7 | MRAS | 0.549 | 8.55E-42 |
| AC007879.7 | TGFB3 | 0.549 | 9.63E-42 |
| AC007879.7 | TNFSF4 | 0.55 | 5.33E-42 |
| AC007879.7 | GNB4 | 0.553 | 1.51E-42 |
| AC007879.7 | LILRA6 | 0.553 | 1.56E-42 |
| AC007879.7 | ECM2 | 0.553 | 1.86E-42 |
| AC007879.7 | FSTL1 | 0.554 | 9.36E-43 |
| AC007879.7 | FKBP14 | 0.554 | 1.06E-42 |
| AC007879.7 | ITGA11 | 0.554 | 1.07E-42 |
| AC007879.7 | CAMK1G | 0.554 | 1.14E-42 |
| AC007879.7 | CSMD2 | 0.554 | 1.19E-42 |
| AC007879.7 | MMP16 | 0.556 | 4.68E-43 |
| AC007879.7 | GPR85 | 0.556 | 4.79E-43 |
| AC007879.7 | GSDMC | 0.556 | 5.94E-43 |
| AC007879.7 | VEGFC | 0.558 | 1.85E-43 |
| AC007879.7 | BTBD19 | 0.558 | 1.89E-43 |
| AC007879.7 | NAV3 | 0.558 | 2.49E-43 |
| AC007879.7 | HRH2 | 0.559 | 1.45E-43 |
| AC007879.7 | PTPN14 | 0.559 | 1.63E-43 |
| AC007879.7 | LILRB3 | 0.56 | 7.96E-44 |
| AC007879.7 | FCGR2A | 0.561 | 5.72E-44 |
| AC007879.7 | GAS1 | 0.561 | 6.59E-44 |
| AC007879.7 | COL1A2 | 0.561 | 6.99E-44 |
| AC007879.7 | LZTS1 | 0.562 | 3.19E-44 |
| AC007879.7 | STC1 | 0.562 | 3.49E-44 |
| AC007879.7 | PXDN | 0.562 | 3.79E-44 |
| AC007879.7 | CHN1 | 0.564 | 1.39E-44 |
| AC007879.7 | SIRPB2 | 0.564 | 1.85E-44 |
| AC007879.7 | KCNE1 | 0.564 | 1.96E-44 |
| AC007879.7 | FCAR | 0.565 | 1.01E-44 |
| AC007879.7 | ADGRF5 | 0.566 | 6.24E-45 |
| AC007879.7 | ADAMTS5 | 0.566 | 6.94E-45 |
| AC007879.7 | TLR2 | 0.566 | 7.92E-45 |
| AC007879.7 | THSD7A | 0.566 | 8.07E-45 |
| AC007879.7 | COL5A1 | 0.567 | 4.36E-45 |
| AC007879.7 | ANTXR1 | 0.567 | 5.44E-45 |
| AC007879.7 | COL6A3 | 0.568 | 3.18E-45 |
| AC007879.7 | ST3GAL6 | 0.568 | 3.42E-45 |
| AC007879.7 | AFAP1L1 | 0.568 | 3.61E-45 |
| AC007879.7 | NRP1 | 0.569 | 1.79E-45 |
| AC007879.7 | CSGALNACT2 | 0.569 | 2.43E-45 |
| AC007879.7 | BICC1 | 0.57 | 1.22E-45 |
| AC007879.7 | CYP1B1 | 0.57 | 1.38E-45 |
| AC007879.7 | ITGAX | 0.57 | 1.57E-45 |
| AC007879.7 | MCC | 0.571 | 7.10E-46 |
| AC007879.7 | ARSI | 0.571 | 7.43E-46 |
| AC007879.7 | PLPP4 | 0.571 | 9.59E-46 |
| AC007879.7 | CTGF | 0.571 | 9.68E-46 |
| AC007879.7 | COL22A1 | 0.572 | 5.06E-46 |
| AC007879.7 | FNDC1 | 0.572 | 6.10E-46 |
| AC007879.7 | PPFIA2 | 0.572 | 6.16E-46 |
| AC007879.7 | DZIP1L | 0.572 | 6.54E-46 |
| AC007879.7 | ZFHX4 | 0.574 | 2.58E-46 |
| AC007879.7 | CDK14 | 0.575 | 1.20E-46 |
| AC007879.7 | CTHRC1 | 0.575 | 1.46E-46 |
| AC007879.7 | CCDC88A | 0.575 | 1.77E-46 |
| AC007879.7 | COL15A1 | 0.577 | 5.34E-47 |
| AC007879.7 | ITGA5 | 0.577 | 5.34E-47 |
| AC007879.7 | PCDH17 | 0.577 | 5.49E-47 |
| AC007879.7 | FBN1 | 0.577 | 5.58E-47 |
| AC007879.7 | LOXHD1 | 0.577 | 7.14E-47 |
| AC007879.7 | PAPPA | 0.578 | 3.07E-47 |
| AC007879.7 | OSMR | 0.579 | 2.19E-47 |
| AC007879.7 | ZNF469 | 0.579 | 2.71E-47 |
| AC007879.7 | DAZL | 0.579 | 2.74E-47 |
| AC007879.7 | CHST11 | 0.58 | 1.33E-47 |
| AC007879.7 | NLRP12 | 0.58 | 1.34E-47 |
| AC007879.7 | FRMD6 | 0.582 | 5.98E-48 |
| AC007879.7 | COL3A1 | 0.582 | 6.34E-48 |
| AC007879.7 | ADAMTS6 | 0.582 | 6.53E-48 |
| AC007879.7 | FAM20A | 0.583 | 4.18E-48 |
| AC007879.7 | FN1 | 0.584 | 2.16E-48 |
| AC007879.7 | CDH11 | 0.584 | 2.33E-48 |
| AC007879.7 | AQP9 | 0.585 | 1.29E-48 |
| AC007879.7 | TCTEX1D1 | 0.585 | 1.31E-48 |
| AC007879.7 | SLC6A1 | 0.585 | 1.33E-48 |
| AC007879.7 | DOK5 | 0.585 | 1.47E-48 |
| AC007879.7 | CLEC4D | 0.585 | 1.75E-48 |
| AC007879.7 | HECW2 | 0.586 | 8.10E-49 |
| AC007879.7 | CDH2 | 0.586 | 9.39E-49 |
| AC007879.7 | SNAI2 | 0.586 | 1.01E-48 |
| AC007879.7 | LPAR4 | 0.586 | 1.25E-48 |
| AC007879.7 | HECW1 | 0.586 | 1.28E-48 |
| AC007879.7 | CYP7B1 | 0.587 | 7.97E-49 |
| AC007879.7 | FAM26E | 0.588 | 3.49E-49 |
| AC007879.7 | NALCN | 0.588 | 3.50E-49 |
| AC007879.7 | PRR16 | 0.589 | 2.08E-49 |
| AC007879.7 | DSE | 0.591 | 8.80E-50 |
| AC007879.7 | TENM4 | 0.592 | 6.45E-50 |
| AC007879.7 | COL12A1 | 0.596 | 1.16E-50 |
| AC007879.7 | SPARC | 0.597 | 6.03E-51 |
| AC007879.7 | THBS2 | 0.598 | 2.98E-51 |
| AC007879.7 | NTM | 0.598 | 3.26E-51 |
| AC007879.7 | SLC1A3 | 0.598 | 4.17E-51 |
| AC007879.7 | SLC2A3 | 0.599 | 1.94E-51 |
| AC007879.7 | PABPC4L | 0.599 | 2.30E-51 |
| AC007879.7 | APCDD1L | 0.599 | 2.78E-51 |
| AC007879.7 | AVPR1A | 0.603 | 3.30E-52 |
| AC007879.7 | HS3ST3A1 | 0.604 | 1.73E-52 |
| AC007879.7 | HTR2A | 0.604 | 1.96E-52 |
| AC007879.7 | C5orf46 | 0.605 | 1.10E-52 |
| AC007879.7 | LMCD1 | 0.605 | 1.17E-52 |
| AC007879.7 | RAB31 | 0.605 | 1.51E-52 |
| AC007879.7 | RFX8 | 0.607 | 4.32E-53 |
| AC007879.7 | ADAMTS12 | 0.607 | 4.51E-53 |
| AC007879.7 | GPR176 | 0.608 | 2.22E-53 |
| AC007879.7 | PLPPR4 | 0.61 | 1.24E-53 |
| AC007879.7 | COL10A1 | 0.611 | 7.29E-54 |
| AC007879.7 | ERMN | 0.612 | 3.01E-54 |
| AC007879.7 | SULF1 | 0.612 | 3.33E-54 |
| AC007879.7 | VGLL3 | 0.612 | 4.92E-54 |
| AC007879.7 | DFNA5 | 0.613 | 2.34E-54 |
| AC007879.7 | EBF2 | 0.614 | 1.14E-54 |
| AC007879.7 | MMP19 | 0.614 | 1.16E-54 |
| AC007879.7 | ADAMTS3 | 0.614 | 1.38E-54 |
| AC007879.7 | SLC11A1 | 0.614 | 1.54E-54 |
| AC007879.7 | COL11A1 | 0.614 | 1.67E-54 |
| AC007879.7 | SPOCD1 | 0.616 | 4.39E-55 |
| AC007879.7 | CD300E | 0.616 | 4.86E-55 |
| AC007879.7 | COL8A1 | 0.616 | 5.41E-55 |
| AC007879.7 | FGF1 | 0.617 | 2.58E-55 |
| AC007879.7 | SPOCK1 | 0.619 | 1.08E-55 |
| AC007879.7 | POSTN | 0.619 | 1.28E-55 |
| AC007879.7 | KCND2 | 0.62 | 7.35E-56 |
| AC007879.7 | CHSY3 | 0.622 | 2.24E-56 |
| AC007879.7 | SGIP1 | 0.623 | 1.21E-56 |
| AC007879.7 | COL5A2 | 0.624 | 9.38E-57 |
| AC007879.7 | SERPINE1 | 0.625 | 3.86E-57 |
| AC007879.7 | TREM1 | 0.626 | 3.61E-57 |
| AC007879.7 | CCDC102B | 0.627 | 1.55E-57 |
| AC007879.7 | VCAN | 0.629 | 5.76E-58 |
| AC007879.7 | OLR1 | 0.629 | 7.36E-58 |
| AC007879.7 | RNF175 | 0.631 | 2.42E-58 |
| AC007879.7 | TNFAIP6 | 0.632 | 1.25E-58 |
| AC007879.7 | GFPT2 | 0.637 | 6.01E-60 |
| AC007879.7 | WISP1 | 0.642 | 5.07E-61 |
| AC007879.7 | COL24A1 | 0.642 | 5.88E-61 |
| AC007879.7 | INHBA | 0.647 | 3.03E-62 |
| AC007879.7 | CLEC5A | 0.648 | 1.72E-62 |
| AC007879.7 | KCNE4 | 0.65 | 3.75E-63 |
| AC007879.7 | PRRX1 | 0.654 | 5.14E-64 |
| AC007879.7 | BCAT1 | 0.655 | 2.39E-64 |
| AC007879.7 | ST6GALNAC5 | 0.655 | 2.97E-64 |
| AC007879.7 | LOX | 0.657 | 7.29E-65 |
| AC007879.7 | FAP | 0.658 | 5.28E-65 |
| AC007879.7 | P4HA3 | 0.663 | 2.36E-66 |
| AC007879.7 | SPRED3 | 0.669 | 7.57E-68 |
| AC007879.7 | NOX4 | 0.671 | 1.41E-68 |
| AC007879.7 | IQCA1 | 0.68 | 4.65E-71 |
| AC007879.7 | KCNJ15 | 0.681 | 3.13E-71 |
| AC007879.7 | ALPK2 | 0.688 | 3.45E-73 |
| AC007879.7 | ADAM12 | 0.689 | 1.46E-73 |
| AC009404.2 | ACADS | -0.509 | 3.41E-35 |
| AC009404.2 | RILP | -0.508 | 3.99E-35 |
| AC009404.2 | CLDN7 | -0.501 | 4.93E-34 |
| AC009404.2 | ATP6V0D1 | -0.486 | 7.14E-32 |
| AC009404.2 | MPDU1 | -0.455 | 1.42E-27 |
| AC009404.2 | AKR7A2 | -0.455 | 1.27E-27 |
| AC009404.2 | RHOC | -0.454 | 1.78E-27 |
| AC009404.2 | ETHE1 | -0.452 | 3.09E-27 |
| AC009404.2 | TST | -0.448 | 8.84E-27 |
| AC009404.2 | FUCA1 | -0.448 | 9.21E-27 |
| AC009404.2 | C1orf210 | -0.446 | 1.56E-26 |
| AC009404.2 | TSPAN1 | -0.44 | 8.30E-26 |
| AC009404.2 | TMEM54 | -0.437 | 2.18E-25 |
| AC009404.2 | UQCR10 | -0.435 | 4.25E-25 |
| AC009404.2 | MYL6 | -0.432 | 8.00E-25 |
| AC009404.2 | SLC25A11 | -0.43 | 1.50E-24 |
| AC009404.2 | LDHD | -0.43 | 1.41E-24 |
| AC009404.2 | HMGCL | -0.429 | 2.27E-24 |
| AC009404.2 | MAPK3 | -0.425 | 6.50E-24 |
| AC009404.2 | CLDN23 | -0.421 | 1.89E-23 |
| AC009404.2 | PGM1 | -0.421 | 1.49E-23 |
| AC009404.2 | PFN1 | -0.42 | 2.21E-23 |
| AC009404.2 | UQCRC1 | -0.418 | 3.60E-23 |
| AC009404.2 | CA2 | -0.411 | 2.09E-22 |
| AC009404.2 | GUCA2B | -0.41 | 3.29E-22 |
| AC009404.2 | CYSTM1 | -0.409 | 4.29E-22 |
| AC009404.2 | SMDT1 | -0.409 | 3.96E-22 |
| AC009404.2 | VAMP8 | -0.409 | 3.69E-22 |
| AC009404.2 | ITM2C | -0.408 | 5.50E-22 |
| AC009404.2 | PXMP2 | -0.408 | 5.27E-22 |
| AC009404.2 | PINK1 | -0.406 | 8.52E-22 |
| AC009404.2 | BLOC1S1 | -0.405 | 1.10E-21 |
| AC009404.2 | PLCD1 | -0.403 | 1.75E-21 |
| AC009404.2 | MGST3 | -0.401 | 2.85E-21 |
| AC009404.2 | BLVRB | -0.4 | 3.72E-21 |
| AC009404.2 | ATP5B | -0.399 | 4.47E-21 |
| AC009404.2 | PRADC1 | -0.398 | 6.10E-21 |
| AC009404.2 | COX5A | -0.397 | 7.02E-21 |
| AC009404.2 | ATP5A1 | -0.395 | 1.31E-20 |
| AC009404.2 | PSMB10 | -0.394 | 1.70E-20 |
| AC009404.2 | B3GALT4 | -0.394 | 1.63E-20 |
| AC009404.2 | CD63 | -0.394 | 1.66E-20 |
| AC009404.2 | LGALS4 | -0.393 | 1.90E-20 |
| AC009404.2 | CAPNS1 | -0.393 | 1.77E-20 |
| AC009404.2 | AKR7A3 | -0.391 | 3.42E-20 |
| AC009404.2 | FXYD3 | -0.39 | 4.47E-20 |
| AC009404.2 | NAA38 | -0.389 | 5.18E-20 |
| AC009404.2 | SQRDL | -0.389 | 5.68E-20 |
| AC009404.2 | CFD | -0.386 | 1.16E-19 |
| AC009404.2 | DPM3 | -0.386 | 1.07E-19 |
| AC009404.2 | CLTB | -0.385 | 1.31E-19 |
| AC009404.2 | C11orf86 | -0.385 | 1.44E-19 |
| AC009404.2 | CA4 | -0.384 | 1.85E-19 |
| AC009404.2 | ECHS1 | -0.383 | 2.12E-19 |
| AC009404.2 | MED11 | -0.383 | 1.95E-19 |
| AC009404.2 | TRAPPC1 | -0.383 | 1.98E-19 |
| AC009404.2 | SECTM1 | -0.383 | 1.93E-19 |
| AC009404.2 | SDCBP2 | -0.382 | 2.41E-19 |
| AC009404.2 | DCTN2 | -0.382 | 2.74E-19 |
| AC009404.2 | CA7 | -0.381 | 3.29E-19 |
| AC009404.2 | OTOP2 | -0.381 | 3.09E-19 |
| AC009404.2 | RABAC1 | -0.381 | 3.36E-19 |
| AC009404.2 | TSPO | -0.38 | 3.76E-19 |
| AC009404.2 | C12orf57 | -0.38 | 4.30E-19 |
| AC009404.2 | MUL1 | -0.38 | 4.09E-19 |
| AC009404.2 | SLC22A18AS | -0.379 | 5.88E-19 |
| AC009404.2 | SULT1A2 | -0.378 | 6.89E-19 |
| AC009404.2 | TMEM82 | -0.378 | 6.44E-19 |
| AC009404.2 | TMEM102 | -0.377 | 8.55E-19 |
| AC009404.2 | NDUFB10 | -0.377 | 9.26E-19 |
| AC009404.2 | NDUFA6 | -0.377 | 8.35E-19 |
| AC009404.2 | TCN2 | -0.376 | 1.12E-18 |
| AC009404.2 | OCEL1 | -0.376 | 1.10E-18 |
| AC009404.2 | ACAA2 | -0.375 | 1.41E-18 |
| AC009404.2 | BEST4 | -0.373 | 2.27E-18 |
| AC009404.2 | ZG16 | -0.372 | 2.62E-18 |
| AC009404.2 | TMEM120A | -0.372 | 2.71E-18 |
| AC009404.2 | SMIM22 | -0.37 | 3.90E-18 |
| AC009404.2 | COX8A | -0.368 | 5.99E-18 |
| AC009404.2 | RPS6KA1 | -0.367 | 7.26E-18 |
| AC009404.2 | BORCS6 | -0.367 | 7.45E-18 |
| AC009404.2 | SRI | -0.367 | 8.35E-18 |
| AC009404.2 | SERTAD1 | -0.366 | 9.30E-18 |
| AC009404.2 | NDUFA2 | -0.366 | 8.89E-18 |
| AC009404.2 | ITPKA | -0.366 | 9.63E-18 |
| AC009404.2 | SCO2 | -0.366 | 9.17E-18 |
| AC009404.2 | IL10RB | -0.366 | 9.57E-18 |
| AC009404.2 | ADTRP | -0.366 | 9.37E-18 |
| AC009404.2 | BTD | -0.366 | 9.25E-18 |
| AC009404.2 | IMPA2 | -0.365 | 1.14E-17 |
| AC009404.2 | C10orf54 | -0.364 | 1.37E-17 |
| AC009404.2 | UBB | -0.363 | 1.83E-17 |
| AC009404.2 | DNASE1L3 | -0.363 | 2.08E-17 |
| AC009404.2 | ATP2A3 | -0.363 | 2.00E-17 |
| AC009404.2 | TMEM125 | -0.362 | 2.19E-17 |
| AC009404.2 | GLTP | -0.362 | 2.50E-17 |
| AC009404.2 | SDHB | -0.362 | 2.26E-17 |
| AC009404.2 | UROD | -0.361 | 3.12E-17 |
| AC009404.2 | SLC44A4 | -0.361 | 2.79E-17 |
| AC009404.2 | SMPD1 | -0.361 | 3.02E-17 |
| AC009404.2 | CHCHD10 | -0.361 | 3.13E-17 |
| AC009404.2 | CDKN1A | -0.36 | 3.63E-17 |
| AC009404.2 | TPRG1L | -0.36 | 3.32E-17 |
| AC009404.2 | PYY | -0.36 | 3.27E-17 |
| AC009404.2 | MMP28 | -0.36 | 3.85E-17 |
| AC009404.2 | ADH1C | -0.36 | 3.85E-17 |
| AC009404.2 | SH3BGRL3 | -0.36 | 3.73E-17 |
| AC009404.2 | CIB1 | -0.359 | 4.32E-17 |
| AC009404.2 | TEX11 | -0.359 | 4.43E-17 |
| AC009404.2 | ITLN1 | -0.359 | 4.37E-17 |
| AC009404.2 | MT1F | -0.359 | 4.37E-17 |
| AC009404.2 | GBA | -0.357 | 7.05E-17 |
| AC009404.2 | CDC42EP5 | -0.357 | 6.42E-17 |
| AC009404.2 | SFN | -0.357 | 6.76E-17 |
| AC009404.2 | ORMDL2 | -0.356 | 8.65E-17 |
| AC009404.2 | TMEM53 | -0.356 | 9.11E-17 |
| AC009404.2 | ALKBH7 | -0.356 | 8.72E-17 |
| AC009404.2 | SERINC2 | -0.356 | 8.13E-17 |
| AC009404.2 | CTSD | -0.355 | 1.10E-16 |
| AC009404.2 | AOC1 | -0.354 | 1.24E-16 |
| AC009404.2 | FBXW5 | -0.353 | 1.42E-16 |
| AC009404.2 | PPP1CA | -0.353 | 1.44E-16 |
| AC009404.2 | CLCA4 | -0.353 | 1.49E-16 |
| AC009404.2 | HLA-C | -0.353 | 1.56E-16 |
| AC009404.2 | GSN | -0.353 | 1.52E-16 |
| AC009404.2 | COX4I1 | -0.351 | 2.60E-16 |
| AC009404.2 | ENHO | -0.351 | 2.23E-16 |
| AC009404.2 | ECI1 | -0.351 | 2.36E-16 |
| AC009404.2 | GUCA2A | -0.351 | 2.55E-16 |
| AC009404.2 | HSD17B2 | -0.35 | 2.87E-16 |
| AC009404.2 | TMEM37 | -0.35 | 3.05E-16 |
| AC009404.2 | CPT2 | -0.35 | 2.68E-16 |
| AC009404.2 | NAT1 | -0.35 | 3.03E-16 |
| AC009404.2 | TXN2 | -0.35 | 2.80E-16 |
| AC009404.2 | ACO2 | -0.35 | 2.72E-16 |
| AC009404.2 | MSRA | -0.349 | 3.73E-16 |
| AC009404.2 | AKR1B10 | -0.347 | 5.92E-16 |
| AC009404.2 | LRP10 | -0.347 | 5.50E-16 |
| AC009404.2 | TMIGD1 | -0.347 | 5.27E-16 |
| AC009404.2 | ANO10 | -0.347 | 5.30E-16 |
| AC009404.2 | ALPI | -0.347 | 5.52E-16 |
| AC009404.2 | HADH | -0.346 | 6.80E-16 |
| AC009404.2 | VSIG2 | -0.346 | 6.46E-16 |
| AC009404.2 | VILL | -0.345 | 7.67E-16 |
| AC009404.2 | KLF4 | -0.345 | 8.50E-16 |
| AC009404.2 | SDHA | -0.345 | 8.22E-16 |
| AC009404.2 | MS4A12 | -0.345 | 7.64E-16 |
| AC009404.2 | SLC25A20 | -0.345 | 8.37E-16 |
| AC009404.2 | EMC6 | -0.344 | 9.75E-16 |
| AC009404.2 | ENDOG | -0.344 | 9.32E-16 |
| AC009404.2 | TMEM171 | -0.344 | 9.53E-16 |
| AC009404.2 | SLC30A10 | -0.343 | 1.35E-15 |
| AC009404.2 | USP2 | -0.343 | 1.28E-15 |
| AC009404.2 | BAK1 | -0.343 | 1.30E-15 |
| AC009404.2 | CFL1 | -0.342 | 1.49E-15 |
| AC009404.2 | CPTP | -0.342 | 1.42E-15 |
| AC009404.2 | SMPDL3A | -0.342 | 1.44E-15 |
| AC009404.2 | SLC25A5 | -0.342 | 1.58E-15 |
| AC009404.2 | GDPD2 | -0.341 | 1.84E-15 |
| AC009404.2 | KDF1 | -0.341 | 1.98E-15 |
| AC009404.2 | NDRG2 | -0.341 | 1.90E-15 |
| AC009404.2 | HECTD3 | -0.34 | 2.26E-15 |
| AC009404.2 | CLDN5 | -0.34 | 2.26E-15 |
| AC009404.2 | RTCB | -0.34 | 2.44E-15 |
| AC009404.2 | TTC22 | -0.339 | 2.89E-15 |
| AC009404.2 | RETSAT | -0.339 | 2.74E-15 |
| AC009404.2 | SDF4 | -0.339 | 2.89E-15 |
| AC009404.2 | MT1G | -0.339 | 2.82E-15 |
| AC009404.2 | 2-Mar | -0.338 | 3.09E-15 |
| AC009404.2 | TNFSF13 | -0.338 | 3.43E-15 |
| AC009404.2 | RNF167 | -0.337 | 4.49E-15 |
| AC009404.2 | BSG | -0.336 | 5.40E-15 |
| AC009404.2 | GRN | -0.336 | 4.73E-15 |
| AC009404.2 | CST3 | -0.336 | 5.31E-15 |
| AC009404.2 | LGALS3 | -0.335 | 6.49E-15 |
| AC009404.2 | TMEM61 | -0.335 | 5.60E-15 |
| AC009404.2 | NUDT18 | -0.335 | 5.64E-15 |
| AC009404.2 | UQCRQ | -0.335 | 5.87E-15 |
| AC009404.2 | MPC1 | -0.334 | 6.97E-15 |
| AC009404.2 | TMEM256 | -0.334 | 6.74E-15 |
| AC009404.2 | CCDC107 | -0.334 | 7.78E-15 |
| AC009404.2 | SST | -0.334 | 7.07E-15 |
| AC009404.2 | TMEM219 | -0.334 | 6.89E-15 |
| AC009404.2 | RAB1B | -0.334 | 7.18E-15 |
| AC009404.2 | ATPIF1 | -0.334 | 8.01E-15 |
| AC009404.2 | SERF2 | -0.333 | 9.56E-15 |
| AC009404.2 | SLC4A4 | -0.333 | 9.22E-15 |
| AC009404.2 | ACVRL1 | -0.333 | 9.04E-15 |
| AC009404.2 | KRT19 | -0.332 | 1.18E-14 |
| AC009404.2 | TNFRSF1A | -0.332 | 1.10E-14 |
| AC009404.2 | CRYL1 | -0.332 | 1.09E-14 |
| AC009404.2 | INPP5K | -0.332 | 1.16E-14 |
| AC009404.2 | SMPDL3B | -0.332 | 1.17E-14 |
| AC009404.2 | UGT1A10 | -0.332 | 1.15E-14 |
| AC009404.2 | CHMP6 | -0.332 | 1.10E-14 |
| AC009404.2 | UGT1A8 | -0.332 | 1.14E-14 |
| AC009404.2 | PIGR | -0.331 | 1.32E-14 |
| AC009404.2 | PLA2G10 | -0.331 | 1.38E-14 |
| AC009404.2 | BMP3 | -0.331 | 1.33E-14 |
| AC009404.2 | CCL28 | -0.331 | 1.27E-14 |
| AC009404.2 | P2RX4 | -0.331 | 1.28E-14 |
| AC009404.2 | PQLC1 | -0.331 | 1.26E-14 |
| AC009404.2 | MIEF2 | -0.331 | 1.42E-14 |
| AC009404.2 | DAO | -0.331 | 1.36E-14 |
| AC009404.2 | COX6A1 | -0.331 | 1.25E-14 |
| AC009404.2 | SCARA5 | -0.331 | 1.27E-14 |
| AC009404.2 | SPIB | -0.331 | 1.29E-14 |
| AC009404.2 | MED18 | -0.33 | 1.69E-14 |
| AC009404.2 | SCGN | -0.33 | 1.73E-14 |
| AC009404.2 | SLC25A1 | -0.329 | 1.98E-14 |
| AC009404.2 | TWF2 | -0.329 | 1.91E-14 |
| AC009404.2 | GPRC5C | -0.328 | 2.52E-14 |
| AC009404.2 | ST6GALNAC6 | -0.328 | 2.16E-14 |
| AC009404.2 | BRINP3 | -0.328 | 2.33E-14 |
| AC009404.2 | AVPI1 | -0.328 | 2.19E-14 |
| AC009404.2 | BAD | -0.328 | 2.21E-14 |
| AC009404.2 | MYO1C | -0.328 | 2.38E-14 |
| AC009404.2 | KRT8 | -0.328 | 2.36E-14 |
| AC009404.2 | MRPL41 | -0.328 | 2.52E-14 |
| AC009404.2 | ZDHHC12 | -0.328 | 2.29E-14 |
| AC009404.2 | LRRC26 | -0.327 | 2.69E-14 |
| AC009404.2 | CHGA | -0.326 | 3.49E-14 |
| AC009404.2 | PRRG2 | -0.326 | 3.53E-14 |
| AC009404.2 | MAL | -0.326 | 3.12E-14 |
| AC009404.2 | TMEM59 | -0.326 | 3.69E-14 |
| AC009404.2 | NDUFS2 | -0.326 | 3.18E-14 |
| AC009404.2 | MT1H | -0.326 | 3.39E-14 |
| AC009404.2 | RHOG | -0.325 | 3.87E-14 |
| AC009404.2 | COX5B | -0.325 | 4.40E-14 |
| AC009404.2 | AQP8 | -0.325 | 3.97E-14 |
| AC009404.2 | PAFAH2 | -0.324 | 4.72E-14 |
| AC009404.2 | GIPC1 | -0.324 | 4.85E-14 |
| AC009404.2 | MPND | -0.324 | 5.16E-14 |
| AC009404.2 | CLEC3B | -0.324 | 4.71E-14 |
| AC009404.2 | VSTM2A | -0.324 | 4.97E-14 |
| AC009404.2 | CD177 | -0.324 | 5.48E-14 |
| AC009404.2 | CHMP2A | -0.324 | 4.77E-14 |
| AC009404.2 | MT1M | -0.324 | 5.42E-14 |
| AC009404.2 | GGT6 | -0.324 | 5.45E-14 |
| AC009404.2 | SIAE | -0.324 | 5.03E-14 |
| AC009404.2 | SESN2 | -0.323 | 5.71E-14 |
| AC009404.2 | DHRS7C | -0.323 | 5.62E-14 |
| AC009404.2 | HSD3B2 | -0.323 | 5.53E-14 |
| AC009404.2 | UGT2B15 | -0.323 | 6.05E-14 |
| AC009404.2 | TNK1 | -0.322 | 7.43E-14 |
| AC009404.2 | CD52 | -0.322 | 7.61E-14 |
| AC009404.2 | MFSD5 | -0.322 | 7.26E-14 |
| AC009404.2 | CLCA1 | -0.322 | 7.03E-14 |
| AC009404.2 | MYL12B | -0.322 | 6.81E-14 |
| AC009404.2 | NDUFS3 | -0.322 | 7.53E-14 |
| AC009404.2 | JCHAIN | -0.321 | 8.34E-14 |
| AC009404.2 | ECH1 | -0.321 | 8.78E-14 |
| AC009404.2 | COX14 | -0.321 | 9.43E-14 |
| AC009404.2 | GSTZ1 | -0.32 | 1.16E-13 |
| AC009404.2 | BTNL8 | -0.32 | 1.08E-13 |
| AC009404.2 | AURKAIP1 | -0.32 | 1.04E-13 |
| AC009404.2 | ATP5F1 | -0.32 | 1.05E-13 |
| AC009404.2 | HSD11B2 | -0.32 | 1.01E-13 |
| AC009404.2 | ALDOA | -0.32 | 1.15E-13 |
| AC009404.2 | LAMTOR4 | -0.32 | 1.04E-13 |
| AC009404.2 | PSMB6 | -0.32 | 1.15E-13 |
| AC009404.2 | PHGR1 | -0.319 | 1.36E-13 |
| AC009404.2 | TPSAB1 | -0.319 | 1.23E-13 |
| AC009404.2 | CRAT | -0.319 | 1.38E-13 |
| AC009404.2 | HRCT1 | -0.319 | 1.36E-13 |
| AC009404.2 | ACOT4 | -0.319 | 1.37E-13 |
| AC009404.2 | SLC27A4 | -0.319 | 1.21E-13 |
| AC009404.2 | ATP5G3 | -0.319 | 1.33E-13 |
| AC009404.2 | ABCG2 | -0.319 | 1.18E-13 |
| AC009404.2 | NBL1 | -0.319 | 1.36E-13 |
| AC009404.2 | CES2 | -0.318 | 1.41E-13 |
| AC009404.2 | GSKIP | -0.318 | 1.68E-13 |
| AC009404.2 | PDLIM1 | -0.318 | 1.46E-13 |
| AC009404.2 | ENTPD8 | -0.318 | 1.68E-13 |
| AC009404.2 | SLC9A3R1 | -0.318 | 1.56E-13 |
| AC009404.2 | HCST | -0.317 | 1.74E-13 |
| AC009404.2 | CDK2AP2 | -0.317 | 1.90E-13 |
| AC009404.2 | PKIB | -0.317 | 1.91E-13 |
| AC009404.2 | CTSS | -0.317 | 1.83E-13 |
| AC009404.2 | NUDT16L1 | -0.317 | 1.83E-13 |
| AC009404.2 | CACFD1 | -0.317 | 1.78E-13 |
| AC009404.2 | ANPEP | -0.317 | 1.74E-13 |
| AC009404.2 | PRSS36 | -0.316 | 2.31E-13 |
| AC009404.2 | CBR1 | -0.316 | 2.40E-13 |
| AC009404.2 | OTOP3 | -0.316 | 2.10E-13 |
| AC009404.2 | HIGD2A | -0.316 | 2.15E-13 |
| AC009404.2 | ZBTB7C | -0.316 | 2.04E-13 |
| AC009404.2 | SMIM12 | -0.316 | 2.33E-13 |
| AC009404.2 | MT2A | -0.316 | 2.24E-13 |
| AC009404.2 | GUCD1 | -0.316 | 2.17E-13 |
| AC009404.2 | SNX17 | -0.315 | 2.55E-13 |
| AC009404.2 | TSTD1 | -0.315 | 2.89E-13 |
| AC009404.2 | MARVELD3 | -0.315 | 2.54E-13 |
| AC009404.2 | SF3B5 | -0.315 | 2.59E-13 |
| AC009404.2 | HBB | -0.315 | 2.83E-13 |
| AC009404.2 | CAPZB | -0.315 | 2.52E-13 |
| AC009404.2 | AMPD1 | -0.314 | 3.47E-13 |
| AC009404.2 | CYBA | -0.314 | 2.97E-13 |
| AC009404.2 | FOLR2 | -0.314 | 3.21E-13 |
| AC009404.2 | SLC25A34 | -0.314 | 3.13E-13 |
| AC009404.2 | CRB3 | -0.313 | 3.50E-13 |
| AC009404.2 | LAMTOR1 | -0.313 | 3.80E-13 |
| AC009404.2 | RARRES3 | -0.313 | 3.62E-13 |
| AC009404.2 | TXNDC17 | -0.313 | 4.10E-13 |
| AC009404.2 | RRAS | -0.313 | 3.93E-13 |
| AC009404.2 | IFI35 | -0.312 | 4.95E-13 |
| AC009404.2 | B2M | -0.312 | 4.24E-13 |
| AC009404.2 | CHP2 | -0.312 | 4.76E-13 |
| AC009404.2 | FIS1 | -0.312 | 4.63E-13 |
| AC009404.2 | STAP2 | -0.312 | 4.67E-13 |
| AC009404.2 | TMEM220 | -0.312 | 4.76E-13 |
| AC009404.2 | NAAA | -0.311 | 5.54E-13 |
| AC009404.2 | MALL | -0.311 | 5.29E-13 |
| AC009404.2 | VAMP5 | -0.311 | 5.06E-13 |
| AC009404.2 | C14orf142 | -0.311 | 5.96E-13 |
| AC009404.2 | DHRS9 | -0.311 | 5.60E-13 |
| AC009404.2 | PLPP1 | -0.311 | 5.76E-13 |
| AC009404.2 | PTRHD1 | -0.311 | 5.51E-13 |
| AC009404.2 | GCNT3 | -0.311 | 5.83E-13 |
| AC009404.2 | IGFBP6 | -0.311 | 5.19E-13 |
| AC009404.2 | ARSA | -0.31 | 6.23E-13 |
| AC009404.2 | COX7A2 | -0.31 | 6.47E-13 |
| AC009404.2 | RNF19B | -0.31 | 6.07E-13 |
| AC009404.2 | C1orf115 | -0.31 | 6.96E-13 |
| AC009404.2 | CANT1 | -0.31 | 6.89E-13 |
| AC009404.2 | PSME1 | -0.31 | 6.67E-13 |
| AC009404.2 | PRSS3 | -0.31 | 6.82E-13 |
| AC009404.2 | RASD2 | -0.31 | 6.00E-13 |
| AC009404.2 | NR1H4 | -0.309 | 7.59E-13 |
| AC009404.2 | CORO1B | -0.309 | 8.39E-13 |
| AC009404.2 | AHCYL2 | -0.309 | 8.48E-13 |
| AC009404.2 | FCGBP | -0.309 | 8.32E-13 |
| AC009404.2 | ASL | -0.309 | 7.36E-13 |
| AC009404.2 | B3GNT7 | -0.309 | 7.86E-13 |
| AC009404.2 | BCAR3 | -0.309 | 7.51E-13 |
| AC009404.2 | TMEM45B | -0.308 | 9.18E-13 |
| AC009404.2 | RNASEK-C17orf49 | -0.308 | 8.93E-13 |
| AC009404.2 | CHP1 | -0.308 | 8.98E-13 |
| AC009404.2 | IFI27 | -0.308 | 9.26E-13 |
| AC009404.2 | SLC52A1 | -0.308 | 9.70E-13 |
| AC009404.2 | PTGDR2 | -0.308 | 9.32E-13 |
| AC009404.2 | RNASE1 | -0.308 | 9.25E-13 |
| AC009404.2 | GPT | -0.308 | 8.86E-13 |
| AC009404.2 | PDLIM2 | -0.307 | 1.02E-12 |
| AC009404.2 | PLAC8 | -0.307 | 1.15E-12 |
| AC009404.2 | SCAMP2 | -0.306 | 1.26E-12 |
| AC009404.2 | MVP | -0.306 | 1.29E-12 |
| AC009404.2 | MRPL54 | -0.306 | 1.41E-12 |
| AC009404.2 | DHRS4 | -0.306 | 1.38E-12 |
| AC009404.2 | STBD1 | -0.306 | 1.41E-12 |
| AC009404.2 | GPA33 | -0.306 | 1.24E-12 |
| AC009404.2 | TP53I3 | -0.305 | 1.60E-12 |
| AC009404.2 | B4GALNT2 | -0.305 | 1.48E-12 |
| AC009404.2 | CLDN3 | -0.305 | 1.53E-12 |
| AC009404.2 | MGAT1 | -0.305 | 1.65E-12 |
| AC009404.2 | CLEC10A | -0.305 | 1.57E-12 |
| AC009404.2 | UQCRFS1 | -0.304 | 1.75E-12 |
| AC009404.2 | GFER | -0.304 | 2.05E-12 |
| AC009404.2 | PSMB8 | -0.304 | 1.94E-12 |
| AC009404.2 | SCNN1B | -0.304 | 1.74E-12 |
| AC009404.2 | TACO1 | -0.304 | 1.99E-12 |
| AC009404.2 | LHPP | -0.304 | 1.88E-12 |
| AC009404.2 | ACP2 | -0.304 | 1.76E-12 |
| AC009404.2 | B3GNT8 | -0.303 | 2.23E-12 |
| AC009404.2 | MRPL34 | -0.303 | 2.08E-12 |
| AC009404.2 | SLC51B | -0.303 | 2.38E-12 |
| AC009404.2 | ZBTB7B | -0.303 | 2.36E-12 |
| AC009404.2 | IL17RC | -0.303 | 2.25E-12 |
| AC009404.2 | PYCARD | -0.303 | 2.07E-12 |
| AC009404.2 | HLA-A | -0.302 | 2.57E-12 |
| AC009404.2 | HADHA | -0.302 | 2.84E-12 |
| AC009404.2 | TMEM106C | -0.302 | 2.89E-12 |
| AC009404.2 | NAGA | -0.302 | 2.79E-12 |
| AC009404.2 | GFRA2 | -0.302 | 2.61E-12 |
| AC009404.2 | NDUFB7 | -0.302 | 2.51E-12 |
| AC009404.2 | HMOX1 | -0.302 | 2.47E-12 |
| AC009404.2 | CCR10 | -0.301 | 3.12E-12 |
| AC009404.2 | ZNF688 | -0.301 | 3.20E-12 |
| AC009404.2 | TMEM72 | -0.301 | 3.26E-12 |
| AC009404.2 | CDHR5 | -0.301 | 3.27E-12 |
| AC009404.2 | ELANE | -0.301 | 3.27E-12 |
| AC009404.2 | CITED2 | -0.301 | 3.25E-12 |
| AC009404.2 | GNB2 | -0.301 | 3.16E-12 |
| AC009404.2 | HADHB | -0.301 | 3.16E-12 |
| AC009404.2 | ARRB1 | -0.301 | 2.93E-12 |
| AC009404.2 | OR2AE1 | 0.301 | 3.11E-12 |
| AC009404.2 | ZBTB1 | 0.301 | 3.19E-12 |
| AC009404.2 | SRGAP3 | 0.301 | 3.25E-12 |
| AC009404.2 | SLF1 | 0.301 | 3.39E-12 |
| AC009404.2 | SENP1 | 0.301 | 3.29E-12 |
| AC009404.2 | BTRC | 0.301 | 3.13E-12 |
| AC009404.2 | OR5P3 | 0.301 | 3.12E-12 |
| AC009404.2 | TMCO5A | 0.301 | 3.16E-12 |
| AC009404.2 | HMGXB3 | 0.301 | 3.31E-12 |
| AC009404.2 | GVQW2 | 0.301 | 3.30E-12 |
| AC009404.2 | MAP4K4 | 0.301 | 3.22E-12 |
| AC009404.2 | SARM1 | 0.301 | 2.96E-12 |
| AC009404.2 | SOCS5 | 0.301 | 3.03E-12 |
| AC009404.2 | FBXO38 | 0.301 | 3.27E-12 |
| AC009404.2 | GOLGA6L4 | 0.301 | 3.41E-12 |
| AC009404.2 | CTAGE6 | 0.301 | 3.09E-12 |
| AC009404.2 | ERICH1 | 0.301 | 3.23E-12 |
| AC009404.2 | TRMT2B | 0.301 | 3.15E-12 |
| AC009404.2 | HACE1 | 0.301 | 2.91E-12 |
| AC009404.2 | SP1 | 0.301 | 3.25E-12 |
| AC009404.2 | ECT2 | 0.301 | 3.14E-12 |
| AC009404.2 | NPVF | 0.301 | 3.34E-12 |
| AC009404.2 | PARD3B | 0.301 | 3.36E-12 |
| AC009404.2 | KIAA0355 | 0.301 | 3.20E-12 |
| AC009404.2 | AC021106.1 | 0.301 | 3.27E-12 |
| AC009404.2 | TFAP4 | 0.302 | 2.66E-12 |
| AC009404.2 | TADA2A | 0.302 | 2.89E-12 |
| AC009404.2 | CLEC18A | 0.302 | 2.57E-12 |
| AC009404.2 | PLGLB2 | 0.302 | 2.47E-12 |
| AC009404.2 | CENPO | 0.302 | 2.60E-12 |
| AC009404.2 | ZDHHC21 | 0.302 | 2.75E-12 |
| AC009404.2 | ZNF644 | 0.302 | 2.56E-12 |
| AC009404.2 | RNF8 | 0.302 | 2.70E-12 |
| AC009404.2 | CNOT6 | 0.302 | 2.53E-12 |
| AC009404.2 | ITGB8 | 0.302 | 2.65E-12 |
| AC009404.2 | ZNF749 | 0.302 | 2.87E-12 |
| AC009404.2 | SPRED3 | 0.302 | 2.72E-12 |
| AC009404.2 | PIP4K2B | 0.302 | 2.48E-12 |
| AC009404.2 | TXNRD3 | 0.302 | 2.54E-12 |
| AC009404.2 | C4orf51 | 0.302 | 2.80E-12 |
| AC009404.2 | GTF2A1L | 0.302 | 2.73E-12 |
| AC009404.2 | SULT6B1 | 0.302 | 2.66E-12 |
| AC009404.2 | OR2A2 | 0.302 | 2.83E-12 |
| AC009404.2 | SPACA7 | 0.302 | 2.54E-12 |
| AC009404.2 | ZNF740 | 0.302 | 2.47E-12 |
| AC009404.2 | NME8 | 0.302 | 2.75E-12 |
| AC009404.2 | FAM208A | 0.302 | 2.45E-12 |
| AC009404.2 | MGAT4D | 0.302 | 2.86E-12 |
| AC009404.2 | ZCCHC14 | 0.302 | 2.58E-12 |
| AC009404.2 | TRIM24 | 0.302 | 2.91E-12 |
| AC009404.2 | OR2H2 | 0.303 | 2.25E-12 |
| AC009404.2 | SPIN4 | 0.303 | 2.16E-12 |
| AC009404.2 | POLR3G | 0.303 | 2.31E-12 |
| AC009404.2 | GVQW1 | 0.303 | 2.07E-12 |
| AC009404.2 | TBCK | 0.303 | 2.14E-12 |
| AC009404.2 | CYP3A43 | 0.303 | 2.09E-12 |
| AC009404.2 | CHEK1 | 0.303 | 2.08E-12 |
| AC009404.2 | C1orf234 | 0.303 | 2.20E-12 |
| AC009404.2 | PIK3CA | 0.303 | 2.23E-12 |
| AC009404.2 | TRIM45 | 0.303 | 2.33E-12 |
| AC009404.2 | C5 | 0.303 | 2.23E-12 |
| AC009404.2 | GRM7 | 0.303 | 2.35E-12 |
| AC009404.2 | PLK4 | 0.303 | 2.23E-12 |
| AC009404.2 | WWOX | 0.303 | 2.16E-12 |
| AC009404.2 | YIPF7 | 0.303 | 2.19E-12 |
| AC009404.2 | EIF5B | 0.303 | 2.16E-12 |
| AC009404.2 | VWA2 | 0.303 | 2.12E-12 |
| AC009404.2 | TBC1D30 | 0.303 | 2.21E-12 |
| AC009404.2 | PACRGL | 0.303 | 2.31E-12 |
| AC009404.2 | SART3 | 0.303 | 2.22E-12 |
| AC009404.2 | LIN37 | 0.303 | 2.15E-12 |
| AC009404.2 | ELF2 | 0.303 | 2.27E-12 |
| AC009404.2 | AF165138.7 | 0.303 | 2.19E-12 |
| AC009404.2 | CMSS1 | 0.303 | 2.36E-12 |
| AC009404.2 | EXOSC3 | 0.303 | 2.07E-12 |
| AC009404.2 | INO80 | 0.303 | 2.08E-12 |
| AC009404.2 | INPP5F | 0.303 | 2.33E-12 |
| AC009404.2 | AWAT1 | 0.303 | 2.43E-12 |
| AC009404.2 | CYP4A22 | 0.303 | 2.41E-12 |
| AC009404.2 | ASUN | 0.303 | 2.14E-12 |
| AC009404.2 | S100A7L2 | 0.303 | 2.22E-12 |
| AC009404.2 | TMPRSS12 | 0.304 | 1.86E-12 |
| AC009404.2 | BUB1B | 0.304 | 1.99E-12 |
| AC009404.2 | ALX4 | 0.304 | 2.00E-12 |
| AC009404.2 | ZC2HC1B | 0.304 | 1.92E-12 |
| AC009404.2 | KAT7 | 0.304 | 2.03E-12 |
| AC009404.2 | PEX5L | 0.304 | 1.84E-12 |
| AC009404.2 | KDM2B | 0.304 | 2.02E-12 |
| AC009404.2 | ACOT6 | 0.304 | 1.76E-12 |
| AC009404.2 | CAV3 | 0.304 | 1.89E-12 |
| AC009404.2 | PGAP1 | 0.304 | 2.05E-12 |
| AC009404.2 | ZNF92 | 0.304 | 1.98E-12 |
| AC009404.2 | FAM84B | 0.304 | 1.99E-12 |
| AC009404.2 | KCTD7 | 0.304 | 1.92E-12 |
| AC009404.2 | TMEM30C | 0.304 | 1.84E-12 |
| AC009404.2 | CEBPZ | 0.304 | 1.95E-12 |
| AC009404.2 | GPBP1 | 0.304 | 2.05E-12 |
| AC009404.2 | TRIM73 | 0.304 | 2.00E-12 |
| AC009404.2 | HLCS | 0.305 | 1.55E-12 |
| AC009404.2 | PZP | 0.305 | 1.55E-12 |
| AC009404.2 | KSR1 | 0.305 | 1.48E-12 |
| AC009404.2 | SCD | 0.305 | 1.66E-12 |
| AC009404.2 | ZNF345 | 0.305 | 1.46E-12 |
| AC009404.2 | SDCCAG8 | 0.305 | 1.54E-12 |
| AC009404.2 | ZNF311 | 0.305 | 1.72E-12 |
| AC009404.2 | FOXN2 | 0.305 | 1.65E-12 |
| AC009404.2 | SULT1A3 | 0.305 | 1.69E-12 |
| AC009404.2 | ZNF620 | 0.305 | 1.52E-12 |
| AC009404.2 | SHANK2 | 0.305 | 1.60E-12 |
| AC009404.2 | YLPM1 | 0.305 | 1.67E-12 |
| AC009404.2 | LRRC10 | 0.305 | 1.63E-12 |
| AC009404.2 | HIVEP2 | 0.305 | 1.72E-12 |
| AC009404.2 | ACCSL | 0.305 | 1.47E-12 |
| AC009404.2 | RFC3 | 0.305 | 1.54E-12 |
| AC009404.2 | TAS2R7 | 0.305 | 1.64E-12 |
| AC009404.2 | PRR14L | 0.305 | 1.58E-12 |
| AC009404.2 | PTRH2 | 0.305 | 1.52E-12 |
| AC009404.2 | LCOR | 0.305 | 1.56E-12 |
| AC009404.2 | MASTL | 0.305 | 1.58E-12 |
| AC009404.2 | SLC4A8 | 0.306 | 1.23E-12 |
| AC009404.2 | RP11-111K18.1 | 0.306 | 1.34E-12 |
| AC009404.2 | BCAN | 0.306 | 1.44E-12 |
| AC009404.2 | MAN2A2 | 0.306 | 1.37E-12 |
| AC009404.2 | PTBP3 | 0.306 | 1.40E-12 |
| AC009404.2 | RHPN1 | 0.306 | 1.44E-12 |
| AC009404.2 | EDNRA | 0.306 | 1.32E-12 |
| AC009404.2 | C6orf10 | 0.306 | 1.23E-12 |
| AC009404.2 | ZNF660 | 0.306 | 1.31E-12 |
| AC009404.2 | EIF2S3 | 0.306 | 1.35E-12 |
| AC009404.2 | PLEKHA1 | 0.306 | 1.22E-12 |
| AC009404.2 | FBXO47 | 0.306 | 1.31E-12 |
| AC009404.2 | ERCC4 | 0.306 | 1.43E-12 |
| AC009404.2 | C1orf194 | 0.306 | 1.34E-12 |
| AC009404.2 | ATF7 | 0.306 | 1.29E-12 |
| AC009404.2 | ATAD3B | 0.306 | 1.28E-12 |
| AC009404.2 | FUS | 0.306 | 1.44E-12 |
| AC009404.2 | SRSF12 | 0.306 | 1.29E-12 |
| AC009404.2 | TAF1B | 0.306 | 1.26E-12 |
| AC009404.2 | MAN2C1 | 0.306 | 1.24E-12 |
| AC009404.2 | FLT1 | 0.306 | 1.23E-12 |
| AC009404.2 | ZC3H7A | 0.307 | 1.07E-12 |
| AC009404.2 | HERC2 | 0.307 | 1.15E-12 |
| AC009404.2 | DEFB116 | 0.307 | 1.09E-12 |
| AC009404.2 | ICE2 | 0.307 | 1.09E-12 |
| AC009404.2 | RNF217 | 0.307 | 1.10E-12 |
| AC009404.2 | ZFAND1 | 0.307 | 1.14E-12 |
| AC009404.2 | MTERF3 | 0.307 | 1.03E-12 |
| AC009404.2 | OR13D1 | 0.307 | 1.15E-12 |
| AC009404.2 | PDS5A | 0.307 | 1.11E-12 |
| AC009404.2 | COL12A1 | 0.307 | 1.13E-12 |
| AC009404.2 | RXFP2 | 0.307 | 1.10E-12 |
| AC009404.2 | ZXDA | 0.307 | 1.18E-12 |
| AC009404.2 | MIOS | 0.307 | 1.14E-12 |
| AC009404.2 | GZF1 | 0.307 | 1.07E-12 |
| AC009404.2 | WAPL | 0.307 | 1.02E-12 |
| AC009404.2 | CTB-50L17.14 | 0.307 | 1.07E-12 |
| AC009404.2 | FREM3 | 0.307 | 1.08E-12 |
| AC009404.2 | PAPD4 | 0.307 | 1.18E-12 |
| AC009404.2 | PAWR | 0.307 | 1.04E-12 |
| AC009404.2 | ZNF763 | 0.307 | 1.19E-12 |
| AC009404.2 | CLDN24 | 0.307 | 1.11E-12 |
| AC009404.2 | C4orf17 | 0.307 | 1.14E-12 |
| AC009404.2 | TRIP12 | 0.307 | 1.04E-12 |
| AC009404.2 | SMCO3 | 0.307 | 1.20E-12 |
| AC009404.2 | EVI5 | 0.307 | 1.08E-12 |
| AC009404.2 | RNF168 | 0.308 | 9.65E-13 |
| AC009404.2 | LENEP | 0.308 | 9.17E-13 |
| AC009404.2 | ATHL1 | 0.308 | 8.53E-13 |
| AC009404.2 | HEXIM2 | 0.308 | 8.72E-13 |
| AC009404.2 | TSNARE1 | 0.308 | 9.83E-13 |
| AC009404.2 | DDX18 | 0.308 | 9.72E-13 |
| AC009404.2 | MAPKBP1 | 0.308 | 9.50E-13 |
| AC009404.2 | RAD54L | 0.308 | 8.69E-13 |
| AC009404.2 | TMEM56-RWDD3 | 0.308 | 9.15E-13 |
| AC009404.2 | KIDINS220 | 0.308 | 8.93E-13 |
| AC009404.2 | OPN5 | 0.308 | 9.21E-13 |
| AC009404.2 | CDH4 | 0.308 | 8.99E-13 |
| AC009404.2 | TTF1 | 0.308 | 8.74E-13 |
| AC009404.2 | CDC7 | 0.308 | 9.79E-13 |
| AC009404.2 | FOXD4L5 | 0.308 | 9.13E-13 |
| AC009404.2 | GUCY1A2 | 0.308 | 9.56E-13 |
| AC009404.2 | MSH4 | 0.308 | 9.43E-13 |
| AC009404.2 | TNKS2 | 0.308 | 9.94E-13 |
| AC009404.2 | ZNF346 | 0.308 | 9.10E-13 |
| AC009404.2 | PARD6B | 0.308 | 8.66E-13 |
| AC009404.2 | TIGD7 | 0.308 | 9.10E-13 |
| AC009404.2 | FAM219A | 0.308 | 1.02E-12 |
| AC009404.2 | LPIN1 | 0.308 | 9.53E-13 |
| AC009404.2 | ZSCAN5A | 0.308 | 9.86E-13 |
| AC009404.2 | C2orf66 | 0.308 | 9.09E-13 |
| AC009404.2 | OR5K3 | 0.309 | 8.52E-13 |
| AC009404.2 | RYR2 | 0.309 | 7.91E-13 |
| AC009404.2 | ALG10 | 0.309 | 7.45E-13 |
| AC009404.2 | PSMG4 | 0.309 | 7.20E-13 |
| AC009404.2 | DKC1 | 0.309 | 7.96E-13 |
| AC009404.2 | LIPI | 0.309 | 8.14E-13 |
| AC009404.2 | NHS | 0.309 | 7.77E-13 |
| AC009404.2 | CTRL | 0.309 | 7.61E-13 |
| AC009404.2 | ZSCAN25 | 0.309 | 8.01E-13 |
| AC009404.2 | PABPN1 | 0.309 | 7.37E-13 |
| AC009404.2 | MCM9 | 0.309 | 7.90E-13 |
| AC009404.2 | DSCC1 | 0.309 | 8.48E-13 |
| AC009404.2 | SLC35G6 | 0.309 | 7.76E-13 |
| AC009404.2 | RBM17 | 0.309 | 7.21E-13 |
| AC009404.2 | OR52I1 | 0.309 | 7.22E-13 |
| AC009404.2 | TRMU | 0.309 | 7.96E-13 |
| AC009404.2 | HCN3 | 0.309 | 7.75E-13 |
| AC009404.2 | RAB3GAP2 | 0.309 | 7.34E-13 |
| AC009404.2 | ARMCX5 | 0.309 | 7.91E-13 |
| AC009404.2 | SCP2D1 | 0.309 | 8.32E-13 |
| AC009404.2 | KCNJ1 | 0.309 | 7.44E-13 |
| AC009404.2 | PHC1 | 0.309 | 8.08E-13 |
| AC009404.2 | HAUS5 | 0.309 | 8.41E-13 |
| AC009404.2 | PPFIBP1 | 0.309 | 7.80E-13 |
| AC009404.2 | DSC1 | 0.309 | 8.00E-13 |
| AC009404.2 | ZIM2 | 0.309 | 7.43E-13 |
| AC009404.2 | RCN1 | 0.309 | 7.16E-13 |
| AC009404.2 | TERF1 | 0.309 | 7.95E-13 |
| AC009404.2 | FAM111B | 0.309 | 7.32E-13 |
| AC009404.2 | DRICH1 | 0.31 | 6.82E-13 |
| AC009404.2 | CHD9 | 0.31 | 6.41E-13 |
| AC009404.2 | TRIM77 | 0.31 | 7.13E-13 |
| AC009404.2 | TCTN1 | 0.31 | 6.32E-13 |
| AC009404.2 | NSUN2 | 0.31 | 6.71E-13 |
| AC009404.2 | SLFN14 | 0.31 | 6.73E-13 |
| AC009404.2 | AKNAD1 | 0.31 | 6.47E-13 |
| AC009404.2 | EPC2 | 0.31 | 6.33E-13 |
| AC009404.2 | C10orf12 | 0.31 | 6.67E-13 |
| AC009404.2 | SMLR1 | 0.31 | 6.94E-13 |
| AC009404.2 | CKAP2 | 0.31 | 6.97E-13 |
| AC009404.2 | DEPDC4 | 0.31 | 6.48E-13 |
| AC009404.2 | POMK | 0.31 | 6.90E-13 |
| AC009404.2 | PDPR | 0.31 | 7.14E-13 |
| AC009404.2 | C10orf62 | 0.31 | 6.34E-13 |
| AC009404.2 | TAF4B | 0.311 | 5.47E-13 |
| AC009404.2 | ILKAP | 0.311 | 5.21E-13 |
| AC009404.2 | GTF2F2 | 0.311 | 5.33E-13 |
| AC009404.2 | AL023806.1 | 0.311 | 5.60E-13 |
| AC009404.2 | SLC5A4 | 0.311 | 5.86E-13 |
| AC009404.2 | DONSON | 0.311 | 5.21E-13 |
| AC009404.2 | NAA15 | 0.311 | 5.80E-13 |
| AC009404.2 | DIAPH3 | 0.311 | 5.02E-13 |
| AC009404.2 | LYSMD4 | 0.311 | 4.99E-13 |
| AC009404.2 | ZNF3 | 0.311 | 5.30E-13 |
| AC009404.2 | IPO9 | 0.311 | 5.58E-13 |
| AC009404.2 | TTLL5 | 0.311 | 5.26E-13 |
| AC009404.2 | ZSCAN23 | 0.311 | 5.60E-13 |
| AC009404.2 | BORCS5 | 0.311 | 5.73E-13 |
| AC009404.2 | GLTSCR1L | 0.311 | 5.22E-13 |
| AC009404.2 | DCAF13 | 0.311 | 5.74E-13 |
| AC009404.2 | RP11-697E2.6 | 0.311 | 5.58E-13 |
| AC009404.2 | NPEPL1 | 0.311 | 5.87E-13 |
| AC009404.2 | TRA2B | 0.312 | 4.84E-13 |
| AC009404.2 | ATP6V1G3 | 0.312 | 4.24E-13 |
| AC009404.2 | SPINK8 | 0.312 | 4.28E-13 |
| AC009404.2 | TSNAX-DISC1 | 0.312 | 4.74E-13 |
| AC009404.2 | NRIP2 | 0.312 | 4.24E-13 |
| AC009404.2 | AC092835.2 | 0.312 | 4.45E-13 |
| AC009404.2 | TWISTNB | 0.312 | 4.93E-13 |
| AC009404.2 | SLC22A14 | 0.312 | 4.63E-13 |
| AC009404.2 | ZNF410 | 0.312 | 4.30E-13 |
| AC009404.2 | GOSR1 | 0.312 | 4.77E-13 |
| AC009404.2 | SYCE2 | 0.312 | 4.64E-13 |
| AC009404.2 | ANKIB1 | 0.312 | 4.23E-13 |
| AC009404.2 | TCFL5 | 0.312 | 4.81E-13 |
| AC009404.2 | MROH7 | 0.312 | 4.77E-13 |
| AC009404.2 | KIAA1841 | 0.312 | 4.75E-13 |
| AC009404.2 | CLEC1B | 0.312 | 4.20E-13 |
| AC009404.2 | OR52B6 | 0.312 | 4.52E-13 |
| AC009404.2 | YAF2 | 0.313 | 4.16E-13 |
| AC009404.2 | NIPSNAP3B | 0.313 | 3.65E-13 |
| AC009404.2 | RQCD1 | 0.313 | 4.13E-13 |
| AC009404.2 | KIAA0586 | 0.313 | 3.64E-13 |
| AC009404.2 | ZNF585B | 0.313 | 3.86E-13 |
| AC009404.2 | NXF1 | 0.313 | 3.76E-13 |
| AC009404.2 | IPO7 | 0.313 | 3.68E-13 |
| AC009404.2 | FKBP15 | 0.313 | 4.14E-13 |
| AC009404.2 | STK3 | 0.313 | 3.98E-13 |
| AC009404.2 | ANKRD13B | 0.313 | 3.97E-13 |
| AC009404.2 | ZBTB18 | 0.313 | 3.64E-13 |
| AC009404.2 | OR8I2 | 0.313 | 4.06E-13 |
| AC009404.2 | KIAA0825 | 0.313 | 3.96E-13 |
| AC009404.2 | TCF24 | 0.313 | 3.57E-13 |
| AC009404.2 | C2orf83 | 0.313 | 3.63E-13 |
| AC009404.2 | HYDIN | 0.313 | 3.88E-13 |
| AC009404.2 | HEATR5B | 0.313 | 4.04E-13 |
| AC009404.2 | APBB2 | 0.313 | 4.08E-13 |
| AC009404.2 | NRDE2 | 0.313 | 3.63E-13 |
| AC009404.2 | RNF212B | 0.313 | 3.64E-13 |
| AC009404.2 | MET | 0.313 | 3.52E-13 |
| AC009404.2 | ZNF836 | 0.313 | 4.10E-13 |
| AC009404.2 | TATDN1 | 0.313 | 3.71E-13 |
| AC009404.2 | PMS2 | 0.313 | 4.02E-13 |
| AC009404.2 | ESYT3 | 0.314 | 3.39E-13 |
| AC009404.2 | TRIM44 | 0.314 | 3.31E-13 |
| AC009404.2 | NIN | 0.314 | 3.15E-13 |
| AC009404.2 | SLC35E2 | 0.314 | 3.35E-13 |
| AC009404.2 | ATXN1 | 0.314 | 2.92E-13 |
| AC009404.2 | RRAGB | 0.314 | 2.91E-13 |
| AC009404.2 | ZNF746 | 0.314 | 3.37E-13 |
| AC009404.2 | NAV2 | 0.314 | 3.00E-13 |
| AC009404.2 | CBX3 | 0.314 | 3.07E-13 |
| AC009404.2 | FBXW8 | 0.314 | 3.01E-13 |
| AC009404.2 | PRRT2 | 0.314 | 3.35E-13 |
| AC009404.2 | WHSC1L1 | 0.314 | 2.93E-13 |
| AC009404.2 | CHTOP | 0.314 | 3.02E-13 |
| AC009404.2 | TRNT1 | 0.314 | 3.23E-13 |
| AC009404.2 | KIAA2012 | 0.314 | 3.06E-13 |
| AC009404.2 | LCAT | 0.314 | 3.45E-13 |
| AC009404.2 | MAP3K19 | 0.314 | 3.02E-13 |
| AC009404.2 | FAM192A | 0.314 | 3.37E-13 |
| AC009404.2 | C9orf72 | 0.314 | 3.13E-13 |
| AC009404.2 | HP1BP3 | 0.314 | 3.41E-13 |
| AC009404.2 | CMYA5 | 0.314 | 3.29E-13 |
| AC009404.2 | RPF2 | 0.314 | 3.44E-13 |
| AC009404.2 | ZKSCAN3 | 0.314 | 2.96E-13 |
| AC009404.2 | KCTD13 | 0.314 | 2.99E-13 |
| AC009404.2 | TROVE2 | 0.315 | 2.62E-13 |
| AC009404.2 | ZNF800 | 0.315 | 2.53E-13 |
| AC009404.2 | EXOSC8 | 0.315 | 2.61E-13 |
| AC009404.2 | GPR179 | 0.315 | 2.83E-13 |
| AC009404.2 | TAS2R38 | 0.315 | 2.74E-13 |
| AC009404.2 | MTRNR2L4 | 0.315 | 2.53E-13 |
| AC009404.2 | CFAP97 | 0.315 | 2.59E-13 |
| AC009404.2 | BLID | 0.315 | 2.82E-13 |
| AC009404.2 | RGPD8 | 0.315 | 2.53E-13 |
| AC009404.2 | OR4F15 | 0.315 | 2.78E-13 |
| AC009404.2 | PAPD5 | 0.315 | 2.88E-13 |
| AC009404.2 | STIL | 0.315 | 2.61E-13 |
| AC009404.2 | GNL2 | 0.315 | 2.78E-13 |
| AC009404.2 | POLK | 0.315 | 2.60E-13 |
| AC009404.2 | CCSER2 | 0.315 | 2.65E-13 |
| AC009404.2 | RP11-795F19.5 | 0.315 | 2.68E-13 |
| AC009404.2 | ASB15 | 0.315 | 2.83E-13 |
| AC009404.2 | KCNE1 | 0.315 | 2.47E-13 |
| AC009404.2 | SLC22A10 | 0.316 | 2.26E-13 |
| AC009404.2 | C20orf173 | 0.316 | 2.22E-13 |
| AC009404.2 | EWSR1 | 0.316 | 2.41E-13 |
| AC009404.2 | DTNB | 0.316 | 2.11E-13 |
| AC009404.2 | C22orf29 | 0.316 | 2.04E-13 |
| AC009404.2 | ARMC3 | 0.316 | 2.30E-13 |
| AC009404.2 | URB2 | 0.316 | 2.36E-13 |
| AC009404.2 | ZNF850 | 0.316 | 2.32E-13 |
| AC009404.2 | FANCC | 0.316 | 2.16E-13 |
| AC009404.2 | KIAA1033 | 0.316 | 2.39E-13 |
| AC009404.2 | THAP6 | 0.316 | 2.09E-13 |
| AC009404.2 | FNDC3B | 0.316 | 2.28E-13 |
| AC009404.2 | AC008522.1 | 0.316 | 2.27E-13 |
| AC009404.2 | USP9X | 0.316 | 2.12E-13 |
| AC009404.2 | OR1L8 | 0.316 | 2.35E-13 |
| AC009404.2 | NEDD1 | 0.316 | 2.16E-13 |
| AC009404.2 | ZNF770 | 0.317 | 1.91E-13 |
| AC009404.2 | CEP112 | 0.317 | 1.91E-13 |
| AC009404.2 | CCDC122 | 0.317 | 2.00E-13 |
| AC009404.2 | NCAPD3 | 0.317 | 1.85E-13 |
| AC009404.2 | WDR12 | 0.317 | 1.83E-13 |
| AC009404.2 | ZNF551 | 0.317 | 1.85E-13 |
| AC009404.2 | ERICH6 | 0.317 | 1.86E-13 |
| AC009404.2 | AL138751.1 | 0.317 | 1.80E-13 |
| AC009404.2 | RP11-561B11.2 | 0.317 | 1.81E-13 |
| AC009404.2 | PDZD8 | 0.317 | 1.91E-13 |
| AC009404.2 | GRM3 | 0.317 | 1.83E-13 |
| AC009404.2 | ZNF724P | 0.317 | 1.73E-13 |
| AC009404.2 | EPC1 | 0.317 | 1.84E-13 |
| AC009404.2 | KRTAP5-7 | 0.317 | 1.77E-13 |
| AC009404.2 | IGIP | 0.317 | 1.88E-13 |
| AC009404.2 | NPPA | 0.317 | 1.78E-13 |
| AC009404.2 | POLR2D | 0.317 | 1.98E-13 |
| AC009404.2 | SPIN3 | 0.317 | 1.97E-13 |
| AC009404.2 | ANKRD42 | 0.317 | 1.96E-13 |
| AC009404.2 | TFCP2 | 0.317 | 1.96E-13 |
| AC009404.2 | PALD1 | 0.317 | 2.01E-13 |
| AC009404.2 | GOLGA4 | 0.317 | 1.84E-13 |
| AC009404.2 | CAD | 0.317 | 1.94E-13 |
| AC009404.2 | C16orf87 | 0.317 | 1.84E-13 |
| AC009404.2 | ZNF550 | 0.317 | 1.81E-13 |
| AC009404.2 | LRRC7 | 0.317 | 1.70E-13 |
| AC009404.2 | MKL2 | 0.317 | 1.72E-13 |
| AC009404.2 | PTAR1 | 0.317 | 1.75E-13 |
| AC009404.2 | FOXJ3 | 0.317 | 1.75E-13 |
| AC009404.2 | C5orf42 | 0.317 | 1.78E-13 |
| AC009404.2 | CEP44 | 0.318 | 1.43E-13 |
| AC009404.2 | MCM10 | 0.318 | 1.44E-13 |
| AC009404.2 | CTD-2370N5.3 | 0.318 | 1.43E-13 |
| AC009404.2 | KPNA1 | 0.318 | 1.53E-13 |
| AC009404.2 | PROSER3 | 0.318 | 1.51E-13 |
| AC009404.2 | ENAH | 0.318 | 1.45E-13 |
| AC009404.2 | TRIM13 | 0.318 | 1.42E-13 |
| AC009404.2 | ERCC3 | 0.318 | 1.43E-13 |
| AC009404.2 | KIFC2 | 0.318 | 1.58E-13 |
| AC009404.2 | IFIT1B | 0.318 | 1.46E-13 |
| AC009404.2 | KLHL23 | 0.318 | 1.57E-13 |
| AC009404.2 | SLC38A1 | 0.318 | 1.55E-13 |
| AC009404.2 | SLC6A1 | 0.318 | 1.65E-13 |
| AC009404.2 | MTERF4 | 0.318 | 1.66E-13 |
| AC009404.2 | HERC1 | 0.318 | 1.61E-13 |
| AC009404.2 | ZBTB21 | 0.318 | 1.62E-13 |
| AC009404.2 | DGKD | 0.318 | 1.46E-13 |
| AC009404.2 | UBR4 | 0.318 | 1.41E-13 |
| AC009404.2 | CEP57 | 0.318 | 1.45E-13 |
| AC009404.2 | GDF11 | 0.318 | 1.55E-13 |
| AC009404.2 | SACS | 0.318 | 1.40E-13 |
| AC009404.2 | RFWD3 | 0.318 | 1.47E-13 |
| AC009404.2 | SUZ12 | 0.318 | 1.55E-13 |
| AC009404.2 | DHX33 | 0.318 | 1.40E-13 |
| AC009404.2 | RCBTB1 | 0.318 | 1.56E-13 |
| AC009404.2 | DHODH | 0.318 | 1.44E-13 |
| AC009404.2 | OSGIN2 | 0.318 | 1.65E-13 |
| AC009404.2 | ZNF460 | 0.318 | 1.48E-13 |
| AC009404.2 | KNOP1 | 0.318 | 1.57E-13 |
| AC009404.2 | GRIPAP1 | 0.318 | 1.40E-13 |
| AC009404.2 | CLASP2 | 0.319 | 1.25E-13 |
| AC009404.2 | HECW2 | 0.319 | 1.20E-13 |
| AC009404.2 | CCDC144A | 0.319 | 1.22E-13 |
| AC009404.2 | GTF3C3 | 0.319 | 1.17E-13 |
| AC009404.2 | PPP1R42 | 0.319 | 1.21E-13 |
| AC009404.2 | MCF2 | 0.319 | 1.26E-13 |
| AC009404.2 | ZUFSP | 0.319 | 1.26E-13 |
| AC009404.2 | SLC7A1 | 0.319 | 1.38E-13 |
| AC009404.2 | TOP2A | 0.319 | 1.17E-13 |
| AC009404.2 | ZNF124 | 0.319 | 1.32E-13 |
| AC009404.2 | LCN12 | 0.319 | 1.18E-13 |
| AC009404.2 | RAD51B | 0.319 | 1.22E-13 |
| AC009404.2 | RB1CC1 | 0.319 | 1.29E-13 |
| AC009404.2 | NUP205 | 0.319 | 1.24E-13 |
| AC009404.2 | AC004158.1 | 0.319 | 1.18E-13 |
| AC009404.2 | C1orf27 | 0.319 | 1.32E-13 |
| AC009404.2 | CST11 | 0.319 | 1.39E-13 |
| AC009404.2 | GPR180 | 0.319 | 1.18E-13 |
| AC009404.2 | CCDC154 | 0.319 | 1.38E-13 |
| AC009404.2 | PLXNA1 | 0.319 | 1.24E-13 |
| AC009404.2 | MED23 | 0.319 | 1.27E-13 |
| AC009404.2 | PDZD9 | 0.319 | 1.22E-13 |
| AC009404.2 | BAZ1A | 0.319 | 1.19E-13 |
| AC009404.2 | SPATA12 | 0.319 | 1.18E-13 |
| AC009404.2 | SMCO2 | 0.32 | 1.12E-13 |
| AC009404.2 | SRPK1 | 0.32 | 1.01E-13 |
| AC009404.2 | KIAA0753 | 0.32 | 1.14E-13 |
| AC009404.2 | ITSN2 | 0.32 | 1.03E-13 |
| AC009404.2 | MCMDC2 | 0.32 | 1.07E-13 |
| AC009404.2 | FLVCR1 | 0.32 | 1.07E-13 |
| AC009404.2 | GUF1 | 0.32 | 1.02E-13 |
| AC009404.2 | SNAPC4 | 0.32 | 9.79E-14 |
| AC009404.2 | USH2A | 0.32 | 1.06E-13 |
| AC009404.2 | ANKRD28 | 0.32 | 1.02E-13 |
| AC009404.2 | CERS5 | 0.32 | 1.15E-13 |
| AC009404.2 | IDI2 | 0.32 | 1.16E-13 |
| AC009404.2 | RRP1B | 0.32 | 9.72E-14 |
| AC009404.2 | OMG | 0.32 | 1.04E-13 |
| AC009404.2 | CEP120 | 0.32 | 1.09E-13 |
| AC009404.2 | WDR36 | 0.32 | 1.03E-13 |
| AC009404.2 | CCNE2 | 0.32 | 1.14E-13 |
| AC009404.2 | TAS2R8 | 0.32 | 1.02E-13 |
| AC009404.2 | RAPGEF5 | 0.32 | 9.64E-14 |
| AC009404.2 | INPP4A | 0.32 | 9.98E-14 |
| AC009404.2 | RFXAP | 0.32 | 1.04E-13 |
| AC009404.2 | MPHOSPH10 | 0.32 | 1.08E-13 |
| AC009404.2 | AGBL2 | 0.321 | 9.09E-14 |
| AC009404.2 | NFYB | 0.321 | 8.20E-14 |
| AC009404.2 | PCDHGA11 | 0.321 | 9.35E-14 |
| AC009404.2 | ACSBG1 | 0.321 | 8.27E-14 |
| AC009404.2 | NFATC2IP | 0.321 | 9.34E-14 |
| AC009404.2 | TEX38 | 0.321 | 8.04E-14 |
| AC009404.2 | LCORL | 0.321 | 8.43E-14 |
| AC009404.2 | GPAM | 0.321 | 8.48E-14 |
| AC009404.2 | YAP1 | 0.321 | 8.96E-14 |
| AC009404.2 | C19orf71 | 0.321 | 8.87E-14 |
| AC009404.2 | URI1 | 0.321 | 8.12E-14 |
| AC009404.2 | OR1J2 | 0.321 | 8.31E-14 |
| AC009404.2 | RFC1 | 0.321 | 8.69E-14 |
| AC009404.2 | ARMC2 | 0.321 | 8.59E-14 |
| AC009404.2 | ARL13B | 0.321 | 8.16E-14 |
| AC009404.2 | GGNBP2 | 0.321 | 8.39E-14 |
| AC009404.2 | OR6A2 | 0.321 | 9.16E-14 |
| AC009404.2 | CDKL5 | 0.321 | 8.08E-14 |
| AC009404.2 | FARP1 | 0.321 | 8.29E-14 |
| AC009404.2 | CLOCK | 0.321 | 8.04E-14 |
| AC009404.2 | ZNF732 | 0.321 | 8.35E-14 |
| AC009404.2 | RPAIN | 0.321 | 9.39E-14 |
| AC009404.2 | GHRL | 0.321 | 9.56E-14 |
| AC009404.2 | GPALPP1 | 0.321 | 8.09E-14 |
| AC009404.2 | SCAF11 | 0.321 | 9.63E-14 |
| AC009404.2 | NCAPG2 | 0.321 | 9.17E-14 |
| AC009404.2 | PCP2 | 0.321 | 8.05E-14 |
| AC009404.2 | CEP192 | 0.321 | 9.03E-14 |
| AC009404.2 | C18orf63 | 0.322 | 7.04E-14 |
| AC009404.2 | IL5 | 0.322 | 7.10E-14 |
| AC009404.2 | MYO7A | 0.322 | 7.01E-14 |
| AC009404.2 | OR2AG2 | 0.322 | 6.77E-14 |
| AC009404.2 | C21orf62 | 0.322 | 7.58E-14 |
| AC009404.2 | KIF18B | 0.322 | 6.73E-14 |
| AC009404.2 | ZNF280D | 0.322 | 7.61E-14 |
| AC009404.2 | RSRC1 | 0.322 | 7.99E-14 |
| AC009404.2 | PPFIA1 | 0.322 | 7.38E-14 |
| AC009404.2 | SYNJ2 | 0.322 | 7.91E-14 |
| AC009404.2 | RIOK1 | 0.322 | 7.62E-14 |
| AC009404.2 | ARMCX4 | 0.322 | 6.72E-14 |
| AC009404.2 | OR51I2 | 0.322 | 7.10E-14 |
| AC009404.2 | EML4 | 0.322 | 6.81E-14 |
| AC009404.2 | ZNF260 | 0.322 | 6.73E-14 |
| AC009404.2 | RNASE9 | 0.322 | 7.03E-14 |
| AC009404.2 | REST | 0.322 | 6.83E-14 |
| AC009404.2 | SET | 0.322 | 6.76E-14 |
| AC009404.2 | BCLAF1 | 0.322 | 6.82E-14 |
| AC009404.2 | KIF18A | 0.322 | 6.79E-14 |
| AC009404.2 | CCDC67 | 0.322 | 7.89E-14 |
| AC009404.2 | RCL1 | 0.322 | 7.84E-14 |
| AC009404.2 | ZNF138 | 0.323 | 6.58E-14 |
| AC009404.2 | LIMD1 | 0.323 | 6.24E-14 |
| AC009404.2 | SCHIP1 | 0.323 | 5.57E-14 |
| AC009404.2 | CEP170 | 0.323 | 6.51E-14 |
| AC009404.2 | UBE2F-SCLY | 0.323 | 6.44E-14 |
| AC009404.2 | EEA1 | 0.323 | 5.81E-14 |
| AC009404.2 | DDTL | 0.323 | 5.50E-14 |
| AC009404.2 | ZNF254 | 0.323 | 6.18E-14 |
| AC009404.2 | SLC25A32 | 0.323 | 6.47E-14 |
| AC009404.2 | TRHR | 0.323 | 6.41E-14 |
| AC009404.2 | PLCB1 | 0.323 | 6.07E-14 |
| AC009404.2 | LRCH1 | 0.323 | 5.79E-14 |
| AC009404.2 | RLF | 0.323 | 5.89E-14 |
| AC009404.2 | ARMC12 | 0.323 | 5.74E-14 |
| AC009404.2 | ALG10B | 0.323 | 5.68E-14 |
| AC009404.2 | ZNF564 | 0.323 | 6.45E-14 |
| AC009404.2 | PPP6R3 | 0.323 | 5.89E-14 |
| AC009404.2 | PRDM2 | 0.323 | 5.83E-14 |
| AC009404.2 | TUBGCP3 | 0.323 | 6.02E-14 |
| AC009404.2 | ZNF563 | 0.323 | 6.23E-14 |
| AC009404.2 | CLDN1 | 0.323 | 5.63E-14 |
| AC009404.2 | TCAP | 0.323 | 6.21E-14 |
| AC009404.2 | MAP3K12 | 0.323 | 6.49E-14 |
| AC009404.2 | ZNF682 | 0.323 | 5.76E-14 |
| AC009404.2 | C9orf43 | 0.323 | 5.88E-14 |
| AC009404.2 | SLC10A1 | 0.323 | 6.43E-14 |
| AC009404.2 | SIAH1 | 0.324 | 4.77E-14 |
| AC009404.2 | ADAMTS9 | 0.324 | 5.10E-14 |
| AC009404.2 | OR52W1 | 0.324 | 5.06E-14 |
| AC009404.2 | SESTD1 | 0.324 | 5.27E-14 |
| AC009404.2 | CEP63 | 0.324 | 4.75E-14 |
| AC009404.2 | IGF1R | 0.324 | 5.17E-14 |
| AC009404.2 | KIAA1257 | 0.324 | 4.66E-14 |
| AC009404.2 | SPAG8 | 0.324 | 4.86E-14 |
| AC009404.2 | OR1N1 | 0.324 | 4.68E-14 |
| AC009404.2 | CCDC70 | 0.324 | 5.21E-14 |
| AC009404.2 | OR2G2 | 0.324 | 4.63E-14 |
| AC009404.2 | N4BP2 | 0.324 | 5.05E-14 |
| AC009404.2 | LRP11 | 0.324 | 5.32E-14 |
| AC009404.2 | NFKBID | 0.324 | 5.36E-14 |
| AC009404.2 | EGLN2 | 0.324 | 5.43E-14 |
| AC009404.2 | DDX5 | 0.324 | 5.10E-14 |
| AC009404.2 | BRD8 | 0.324 | 5.40E-14 |
| AC009404.2 | CDH6 | 0.324 | 4.56E-14 |
| AC009404.2 | PHKG1 | 0.324 | 4.65E-14 |
| AC009404.2 | PLXNB1 | 0.325 | 4.11E-14 |
| AC009404.2 | DNM3 | 0.325 | 4.28E-14 |
| AC009404.2 | EXOC8 | 0.325 | 4.33E-14 |
| AC009404.2 | C3orf17 | 0.325 | 4.14E-14 |
| AC009404.2 | TGM1 | 0.325 | 4.13E-14 |
| AC009404.2 | SLC25A14 | 0.325 | 4.44E-14 |
| AC009404.2 | HRNR | 0.325 | 3.81E-14 |
| AC009404.2 | RBPJ | 0.325 | 4.54E-14 |
| AC009404.2 | POGLUT1 | 0.325 | 4.06E-14 |
| AC009404.2 | SRRM1 | 0.325 | 4.37E-14 |
| AC009404.2 | RPL7L1 | 0.325 | 3.94E-14 |
| AC009404.2 | KBTBD6 | 0.325 | 4.13E-14 |
| AC009404.2 | NFE2L3 | 0.325 | 3.89E-14 |
| AC009404.2 | LCA5L | 0.325 | 4.38E-14 |
| AC009404.2 | PTCD3 | 0.325 | 4.35E-14 |
| AC009404.2 | FAM106A | 0.325 | 3.91E-14 |
| AC009404.2 | LETMD1 | 0.325 | 4.05E-14 |
| AC009404.2 | ZNF253 | 0.325 | 4.20E-14 |
| AC009404.2 | FSD2 | 0.325 | 4.03E-14 |
| AC009404.2 | STX2 | 0.325 | 4.04E-14 |
| AC009404.2 | ZNF69 | 0.325 | 3.84E-14 |
| AC009404.2 | PIBF1 | 0.326 | 3.76E-14 |
| AC009404.2 | ZNF154 | 0.326 | 3.39E-14 |
| AC009404.2 | UBR3 | 0.326 | 3.43E-14 |
| AC009404.2 | IAPP | 0.326 | 3.75E-14 |
| AC009404.2 | NUP62CL | 0.326 | 3.69E-14 |
| AC009404.2 | KRI1 | 0.326 | 3.62E-14 |
| AC009404.2 | NOLC1 | 0.326 | 3.25E-14 |
| AC009404.2 | MTRF1 | 0.326 | 3.23E-14 |
| AC009404.2 | LRRC37A3 | 0.326 | 3.28E-14 |
| AC009404.2 | OR1L3 | 0.326 | 3.74E-14 |
| AC009404.2 | PHF3 | 0.326 | 3.17E-14 |
| AC009404.2 | RNF149 | 0.326 | 3.12E-14 |
| AC009404.2 | SLC9B1 | 0.326 | 3.73E-14 |
| AC009404.2 | SMAD5 | 0.326 | 3.44E-14 |
| AC009404.2 | TAS2R41 | 0.326 | 3.12E-14 |
| AC009404.2 | SPTBN2 | 0.326 | 3.64E-14 |
| AC009404.2 | DDX51 | 0.326 | 3.27E-14 |
| AC009404.2 | TCOF1 | 0.326 | 3.39E-14 |
| AC009404.2 | ERN1 | 0.326 | 3.63E-14 |
| AC009404.2 | LGR5 | 0.327 | 2.63E-14 |
| AC009404.2 | DDX52 | 0.327 | 2.93E-14 |
| AC009404.2 | CDK10 | 0.327 | 3.05E-14 |
| AC009404.2 | SYT16 | 0.327 | 2.88E-14 |
| AC009404.2 | CTD-2583A14.10 | 0.327 | 2.88E-14 |
| AC009404.2 | ZNF77 | 0.327 | 3.05E-14 |
| AC009404.2 | EXOSC2 | 0.327 | 3.09E-14 |
| AC009404.2 | OR56A1 | 0.327 | 3.02E-14 |
| AC009404.2 | DROSHA | 0.327 | 2.77E-14 |
| AC009404.2 | PLSCR2 | 0.327 | 2.92E-14 |
| AC009404.2 | SKA3 | 0.327 | 3.03E-14 |
| AC009404.2 | PPHLN1 | 0.327 | 2.72E-14 |
| AC009404.2 | KIAA1429 | 0.327 | 3.06E-14 |
| AC009404.2 | C12orf71 | 0.327 | 2.86E-14 |
| AC009404.2 | ANKRD62 | 0.327 | 2.78E-14 |
| AC009404.2 | MST1 | 0.327 | 2.63E-14 |
| AC009404.2 | NANP | 0.327 | 2.94E-14 |
| AC009404.2 | ERV3-1 | 0.327 | 3.08E-14 |
| AC009404.2 | SLC5A3 | 0.327 | 2.78E-14 |
| AC009404.2 | ZFP69 | 0.327 | 2.78E-14 |
| AC009404.2 | FUBP3 | 0.327 | 2.76E-14 |
| AC009404.2 | CNOT4 | 0.327 | 2.73E-14 |
| AC009404.2 | BBX | 0.327 | 2.81E-14 |
| AC009404.2 | PHF12 | 0.328 | 2.34E-14 |
| AC009404.2 | HSPH1 | 0.328 | 2.58E-14 |
| AC009404.2 | ZNF286B | 0.328 | 2.48E-14 |
| AC009404.2 | FZD3 | 0.328 | 2.33E-14 |
| AC009404.2 | SCAF8 | 0.328 | 2.25E-14 |
| AC009404.2 | PRR11 | 0.328 | 2.41E-14 |
| AC009404.2 | ZNF215 | 0.328 | 2.55E-14 |
| AC009404.2 | ZXDB | 0.328 | 2.46E-14 |
| AC009404.2 | VTI1A | 0.328 | 2.17E-14 |
| AC009404.2 | ZNF404 | 0.328 | 2.32E-14 |
| AC009404.2 | PDP1 | 0.328 | 2.55E-14 |
| AC009404.2 | CFAP161 | 0.328 | 2.48E-14 |
| AC009404.2 | DCUN1D4 | 0.328 | 2.42E-14 |
| AC009404.2 | TMEM33 | 0.328 | 2.54E-14 |
| AC009404.2 | NUP98 | 0.328 | 2.48E-14 |
| AC009404.2 | UPP2 | 0.328 | 2.49E-14 |
| AC009404.2 | ITGA2 | 0.328 | 2.33E-14 |
| AC009404.2 | HIST2H2BF | 0.328 | 2.42E-14 |
| AC009404.2 | MEPE | 0.328 | 2.56E-14 |
| AC009404.2 | OR6V1 | 0.328 | 2.28E-14 |
| AC009404.2 | SGOL2 | 0.328 | 2.56E-14 |
| AC009404.2 | SLC26A1 | 0.329 | 2.12E-14 |
| AC009404.2 | RPE | 0.329 | 1.79E-14 |
| AC009404.2 | PDP2 | 0.329 | 2.12E-14 |
| AC009404.2 | SGOL1 | 0.329 | 1.77E-14 |
| AC009404.2 | KIF23 | 0.329 | 2.08E-14 |
| AC009404.2 | CENPC | 0.329 | 1.95E-14 |
| AC009404.2 | TMPRSS11A | 0.329 | 1.97E-14 |
| AC009404.2 | AL049872.1 | 0.329 | 1.80E-14 |
| AC009404.2 | HOXB3 | 0.329 | 1.93E-14 |
| AC009404.2 | KDM5A | 0.329 | 2.04E-14 |
| AC009404.2 | RNF216 | 0.329 | 1.93E-14 |
| AC009404.2 | INTS4 | 0.329 | 1.91E-14 |
| AC009404.2 | RNF43 | 0.329 | 1.95E-14 |
| AC009404.2 | CD3EAP | 0.329 | 1.82E-14 |
| AC009404.2 | GATAD1 | 0.33 | 1.64E-14 |
| AC009404.2 | ZNF233 | 0.33 | 1.59E-14 |
| AC009404.2 | MED17 | 0.33 | 1.67E-14 |
| AC009404.2 | CNGB3 | 0.33 | 1.59E-14 |
| AC009404.2 | METTL2B | 0.33 | 1.68E-14 |
| AC009404.2 | ADGRF2 | 0.33 | 1.64E-14 |
| AC009404.2 | SUV39H2 | 0.33 | 1.68E-14 |
| AC009404.2 | CKAP2L | 0.33 | 1.59E-14 |
| AC009404.2 | DNM1L | 0.33 | 1.72E-14 |
| AC009404.2 | TRIM27 | 0.33 | 1.68E-14 |
| AC009404.2 | MROH9 | 0.33 | 1.74E-14 |
| AC009404.2 | NFX1 | 0.33 | 1.48E-14 |
| AC009404.2 | LRRTM2 | 0.33 | 1.64E-14 |
| AC009404.2 | KIAA1109 | 0.33 | 1.60E-14 |
| AC009404.2 | C12orf42 | 0.33 | 1.57E-14 |
| AC009404.2 | KLHDC10 | 0.33 | 1.50E-14 |
| AC009404.2 | FAM205A | 0.33 | 1.53E-14 |
| AC009404.2 | LRRC27 | 0.33 | 1.49E-14 |
| AC009404.2 | IQUB | 0.33 | 1.72E-14 |
| AC009404.2 | HOXA2 | 0.33 | 1.58E-14 |
| AC009404.2 | NCOA6 | 0.33 | 1.54E-14 |
| AC009404.2 | EPB41L5 | 0.33 | 1.73E-14 |
| AC009404.2 | DGKI | 0.331 | 1.27E-14 |
| AC009404.2 | GGA2 | 0.331 | 1.37E-14 |
| AC009404.2 | OR14A2 | 0.331 | 1.20E-14 |
| AC009404.2 | MPHOSPH8 | 0.331 | 1.33E-14 |
| AC009404.2 | C2orf78 | 0.331 | 1.27E-14 |
| AC009404.2 | AGFG1 | 0.331 | 1.30E-14 |
| AC009404.2 | ZNF554 | 0.331 | 1.39E-14 |
| AC009404.2 | ZNF555 | 0.331 | 1.31E-14 |
| AC009404.2 | FBXO45 | 0.331 | 1.31E-14 |
| AC009404.2 | HJURP | 0.331 | 1.43E-14 |
| AC009404.2 | ARFGEF1 | 0.331 | 1.20E-14 |
| AC009404.2 | CWC25 | 0.331 | 1.28E-14 |
| AC009404.2 | PFN4 | 0.331 | 1.27E-14 |
| AC009404.2 | CEP68 | 0.331 | 1.40E-14 |
| AC009404.2 | TNFSF15 | 0.331 | 1.28E-14 |
| AC009404.2 | ERCC5 | 0.331 | 1.37E-14 |
| AC009404.2 | NPIPB8 | 0.331 | 1.20E-14 |
| AC009404.2 | ASCC3 | 0.332 | 1.12E-14 |
| AC009404.2 | ZNF286A | 0.332 | 1.01E-14 |
| AC009404.2 | NOL12 | 0.332 | 1.02E-14 |
| AC009404.2 | PIGM | 0.332 | 1.10E-14 |
| AC009404.2 | PAPOLG | 0.332 | 9.95E-15 |
| AC009404.2 | PLEKHM3 | 0.332 | 1.15E-14 |
| AC009404.2 | DDIAS | 0.332 | 1.13E-14 |
| AC009404.2 | RP11-613M10.9 | 0.332 | 1.18E-14 |
| AC009404.2 | ZSCAN30 | 0.332 | 1.00E-14 |
| AC009404.2 | DNAJB13 | 0.332 | 1.15E-14 |
| AC009404.2 | S100G | 0.332 | 1.06E-14 |
| AC009404.2 | FANCD2 | 0.332 | 1.06E-14 |
| AC009404.2 | SLCO5A1 | 0.332 | 1.13E-14 |
| AC009404.2 | C3orf30 | 0.332 | 1.16E-14 |
| AC009404.2 | CST9 | 0.332 | 1.02E-14 |
| AC009404.2 | PPP1R32 | 0.333 | 8.91E-15 |
| AC009404.2 | ALKBH8 | 0.333 | 8.20E-15 |
| AC009404.2 | ATG16L1 | 0.333 | 8.16E-15 |
| AC009404.2 | UGGT1 | 0.333 | 9.19E-15 |
| AC009404.2 | CXorf23 | 0.333 | 9.56E-15 |
| AC009404.2 | IQCB1 | 0.333 | 9.67E-15 |
| AC009404.2 | CCND1 | 0.333 | 8.71E-15 |
| AC009404.2 | ZNF227 | 0.333 | 8.19E-15 |
| AC009404.2 | PLEKHH1 | 0.333 | 8.60E-15 |
| AC009404.2 | ESM1 | 0.333 | 8.34E-15 |
| AC009404.2 | DDX46 | 0.333 | 9.29E-15 |
| AC009404.2 | EFCAB8 | 0.333 | 8.98E-15 |
| AC009404.2 | AC013461.1 | 0.333 | 9.65E-15 |
| AC009404.2 | DGCR8 | 0.333 | 9.48E-15 |
| AC009404.2 | SKIL | 0.333 | 8.19E-15 |
| AC009404.2 | CHMP4A | 0.333 | 8.86E-15 |
| AC009404.2 | SETX | 0.333 | 9.71E-15 |
| AC009404.2 | SPTBN5 | 0.333 | 9.61E-15 |
| AC009404.2 | CCDC82 | 0.333 | 8.32E-15 |
| AC009404.2 | TAS2R39 | 0.333 | 9.26E-15 |
| AC009404.2 | RP11-385D13.1 | 0.333 | 9.15E-15 |
| AC009404.2 | IFNA5 | 0.333 | 8.94E-15 |
| AC009404.2 | MLLT4 | 0.333 | 9.25E-15 |
| AC009404.2 | DCAF10 | 0.334 | 6.87E-15 |
| AC009404.2 | E2F7 | 0.334 | 7.87E-15 |
| AC009404.2 | ARID5B | 0.334 | 7.15E-15 |
| AC009404.2 | ZNF547 | 0.334 | 6.81E-15 |
| AC009404.2 | DIP2A | 0.334 | 6.66E-15 |
| AC009404.2 | SRSF1 | 0.334 | 6.87E-15 |
| AC009404.2 | SMURF2 | 0.334 | 6.84E-15 |
| AC009404.2 | ZBED3 | 0.334 | 7.59E-15 |
| AC009404.2 | FAM199X | 0.334 | 7.49E-15 |
| AC009404.2 | UNKL | 0.334 | 7.64E-15 |
| AC009404.2 | ABCC10 | 0.334 | 7.95E-15 |
| AC009404.2 | MRRF | 0.334 | 7.60E-15 |
| AC009404.2 | SYDE2 | 0.334 | 7.15E-15 |
| AC009404.2 | GDAP1L1 | 0.334 | 8.08E-15 |
| AC009404.2 | C12orf66 | 0.334 | 7.76E-15 |
| AC009404.2 | TRIT1 | 0.334 | 6.77E-15 |
| AC009404.2 | TMEM41A | 0.334 | 7.13E-15 |
| AC009404.2 | DIAPH2 | 0.334 | 8.08E-15 |
| AC009404.2 | EFCAB3 | 0.334 | 7.61E-15 |
| AC009404.2 | TBC1D16 | 0.335 | 5.68E-15 |
| AC009404.2 | PURB | 0.335 | 6.53E-15 |
| AC009404.2 | GJA9 | 0.335 | 6.25E-15 |
| AC009404.2 | SNX27 | 0.335 | 5.75E-15 |
| AC009404.2 | MAP3K9 | 0.335 | 5.82E-15 |
| AC009404.2 | IMMP2L | 0.335 | 5.58E-15 |
| AC009404.2 | UCP3 | 0.335 | 6.10E-15 |
| AC009404.2 | ARHGAP33 | 0.335 | 6.59E-15 |
| AC009404.2 | SMG7 | 0.335 | 6.58E-15 |
| AC009404.2 | AMZ1 | 0.335 | 5.99E-15 |
| AC009404.2 | GPSM2 | 0.335 | 5.82E-15 |
| AC009404.2 | TGFBRAP1 | 0.335 | 6.50E-15 |
| AC009404.2 | ORC3 | 0.335 | 6.62E-15 |
| AC009404.2 | NPIPB3 | 0.335 | 5.54E-15 |
| AC009404.2 | BMS1 | 0.335 | 5.69E-15 |
| AC009404.2 | KIN | 0.335 | 6.12E-15 |
| AC009404.2 | TECTB | 0.335 | 6.04E-15 |
| AC009404.2 | BTBD18 | 0.335 | 6.01E-15 |
| AC009404.2 | NUP153 | 0.335 | 6.48E-15 |
| AC009404.2 | WNK1 | 0.336 | 5.44E-15 |
| AC009404.2 | ZNF567 | 0.336 | 4.76E-15 |
| AC009404.2 | ZNF112 | 0.336 | 4.70E-15 |
| AC009404.2 | ERCC6L | 0.336 | 5.33E-15 |
| AC009404.2 | RBM12 | 0.336 | 5.44E-15 |
| AC009404.2 | METTL21A | 0.336 | 4.54E-15 |
| AC009404.2 | CFAP58 | 0.336 | 4.86E-15 |
| AC009404.2 | CD200R1L | 0.336 | 5.32E-15 |
| AC009404.2 | GOLGA8N | 0.336 | 4.69E-15 |
| AC009404.2 | ZRANB1 | 0.336 | 4.77E-15 |
| AC009404.2 | CEP126 | 0.337 | 4.23E-15 |
| AC009404.2 | OR9A4 | 0.337 | 3.90E-15 |
| AC009404.2 | ZNF441 | 0.337 | 4.45E-15 |
| AC009404.2 | ZNF225 | 0.337 | 4.45E-15 |
| AC009404.2 | LRRC74A | 0.337 | 4.05E-15 |
| AC009404.2 | MOV10L1 | 0.337 | 3.81E-15 |
| AC009404.2 | RHD | 0.337 | 4.45E-15 |
| AC009404.2 | GPR21 | 0.337 | 3.86E-15 |
| AC009404.2 | CHD1 | 0.337 | 3.72E-15 |
| AC009404.2 | CNTD1 | 0.337 | 3.70E-15 |
| AC009404.2 | PCDHGC4 | 0.337 | 3.94E-15 |
| AC009404.2 | RECQL5 | 0.337 | 4.14E-15 |
| AC009404.2 | PGBD1 | 0.337 | 4.41E-15 |
| AC009404.2 | ZNF862 | 0.337 | 4.09E-15 |
| AC009404.2 | CCDC77 | 0.337 | 4.11E-15 |
| AC009404.2 | ZSCAN32 | 0.337 | 4.00E-15 |
| AC009404.2 | COX20 | 0.337 | 4.18E-15 |
| AC009404.2 | PHF21A | 0.337 | 3.90E-15 |
| AC009404.2 | WDR90 | 0.338 | 3.47E-15 |
| AC009404.2 | RTEL1-TNFRSF6B | 0.338 | 3.26E-15 |
| AC009404.2 | AC069499.1 | 0.338 | 3.66E-15 |
| AC009404.2 | ZNF20 | 0.338 | 3.18E-15 |
| AC009404.2 | ABCC1 | 0.338 | 3.18E-15 |
| AC009404.2 | EEF1E1 | 0.338 | 3.54E-15 |
| AC009404.2 | NUFIP1 | 0.338 | 3.24E-15 |
| AC009404.2 | ZNF382 | 0.338 | 3.59E-15 |
| AC009404.2 | ARL10 | 0.338 | 3.16E-15 |
| AC009404.2 | DICER1 | 0.338 | 3.61E-15 |
| AC009404.2 | MEX3A | 0.338 | 3.45E-15 |
| AC009404.2 | GUCA1B | 0.338 | 3.52E-15 |
| AC009404.2 | NPHP4 | 0.338 | 3.48E-15 |
| AC009404.2 | AASS | 0.338 | 3.25E-15 |
| AC009404.2 | DDX21 | 0.338 | 3.20E-15 |
| AC009404.2 | GCSAM | 0.339 | 2.79E-15 |
| AC009404.2 | CDK5RAP2 | 0.339 | 2.96E-15 |
| AC009404.2 | IMMP1L | 0.339 | 2.97E-15 |
| AC009404.2 | NXPH1 | 0.339 | 2.87E-15 |
| AC009404.2 | PDC | 0.339 | 2.91E-15 |
| AC009404.2 | B3GALNT2 | 0.339 | 2.82E-15 |
| AC009404.2 | MYO16 | 0.339 | 2.96E-15 |
| AC009404.2 | SFT2D2 | 0.339 | 2.73E-15 |
| AC009404.2 | KCNAB3 | 0.339 | 2.85E-15 |
| AC009404.2 | SPATA5 | 0.339 | 2.71E-15 |
| AC009404.2 | SLC22A15 | 0.339 | 2.97E-15 |
| AC009404.2 | OR10A4 | 0.339 | 2.82E-15 |
| AC009404.2 | COA1 | 0.339 | 2.77E-15 |
| AC009404.2 | LNPEP | 0.339 | 2.84E-15 |
| AC009404.2 | SLTM | 0.339 | 2.52E-15 |
| AC009404.2 | ZNF214 | 0.339 | 2.62E-15 |
| AC009404.2 | USP50 | 0.34 | 2.08E-15 |
| AC009404.2 | RP11-38C17.1 | 0.34 | 2.13E-15 |
| AC009404.2 | LDHAL6B | 0.34 | 2.41E-15 |
| AC009404.2 | TMCC1 | 0.34 | 2.36E-15 |
| AC009404.2 | PRH2 | 0.34 | 2.10E-15 |
| AC009404.2 | ZNF177 | 0.34 | 2.34E-15 |
| AC009404.2 | DDX42 | 0.34 | 2.26E-15 |
| AC009404.2 | YTHDC1 | 0.34 | 2.16E-15 |
| AC009404.2 | CHRNA10 | 0.34 | 2.12E-15 |
| AC009404.2 | ZNF354A | 0.34 | 2.22E-15 |
| AC009404.2 | TTC32 | 0.34 | 2.33E-15 |
| AC009404.2 | TSPYL6 | 0.34 | 2.40E-15 |
| AC009404.2 | MAT2A | 0.34 | 2.22E-15 |
| AC009404.2 | PHKA2 | 0.34 | 2.35E-15 |
| AC009404.2 | ALG1L2 | 0.341 | 1.96E-15 |
| AC009404.2 | DNAJC10 | 0.341 | 1.78E-15 |
| AC009404.2 | FSHR | 0.341 | 1.68E-15 |
| AC009404.2 | ODF2L | 0.341 | 1.84E-15 |
| AC009404.2 | CSTF3 | 0.341 | 1.77E-15 |
| AC009404.2 | DHX34 | 0.341 | 1.87E-15 |
| AC009404.2 | IFNK | 0.341 | 1.71E-15 |
| AC009404.2 | PARP6 | 0.341 | 1.85E-15 |
| AC009404.2 | TMEM14EP | 0.341 | 1.91E-15 |
| AC009404.2 | LSM11 | 0.341 | 1.87E-15 |
| AC009404.2 | AC073657.1 | 0.341 | 1.88E-15 |
| AC009404.2 | BCL11B | 0.341 | 1.93E-15 |
| AC009404.2 | UTP23 | 0.341 | 1.78E-15 |
| AC009404.2 | SLC7A6OS | 0.341 | 1.81E-15 |
| AC009404.2 | C19orf44 | 0.342 | 1.60E-15 |
| AC009404.2 | MYH8 | 0.342 | 1.53E-15 |
| AC009404.2 | ABHD14A-ACY1 | 0.342 | 1.65E-15 |
| AC009404.2 | AC022819.2 | 0.342 | 1.52E-15 |
| AC009404.2 | SPATA33 | 0.342 | 1.63E-15 |
| AC009404.2 | PRMT3 | 0.342 | 1.49E-15 |
| AC009404.2 | GPR78 | 0.342 | 1.63E-15 |
| AC009404.2 | DUS4L | 0.342 | 1.41E-15 |
| AC009404.2 | PLET1 | 0.342 | 1.44E-15 |
| AC009404.2 | LEKR1 | 0.342 | 1.66E-15 |
| AC009404.2 | ZMYND8 | 0.342 | 1.62E-15 |
| AC009404.2 | TAF1A | 0.342 | 1.56E-15 |
| AC009404.2 | METTL21C | 0.342 | 1.58E-15 |
| AC009404.2 | PTK2 | 0.342 | 1.41E-15 |
| AC009404.2 | POLE | 0.342 | 1.63E-15 |
| AC009404.2 | DIS3 | 0.342 | 1.44E-15 |
| AC009404.2 | PTPN14 | 0.342 | 1.47E-15 |
| AC009404.2 | MINA | 0.342 | 1.44E-15 |
| AC009404.2 | TNNT2 | 0.343 | 1.22E-15 |
| AC009404.2 | GPR75-ASB3 | 0.343 | 1.18E-15 |
| AC009404.2 | NAP1L1 | 0.343 | 1.30E-15 |
| AC009404.2 | ARIH2 | 0.343 | 1.19E-15 |
| AC009404.2 | COL7A1 | 0.343 | 1.26E-15 |
| AC009404.2 | ENO4 | 0.343 | 1.12E-15 |
| AC009404.2 | RAD50 | 0.343 | 1.28E-15 |
| AC009404.2 | DGAT2L6 | 0.343 | 1.25E-15 |
| AC009404.2 | MAU2 | 0.343 | 1.19E-15 |
| AC009404.2 | PTH | 0.343 | 1.35E-15 |
| AC009404.2 | INHBA | 0.343 | 1.17E-15 |
| AC009404.2 | ZFP14 | 0.343 | 1.16E-15 |
| AC009404.2 | MEGF11 | 0.343 | 1.32E-15 |
| AC009404.2 | NOC3L | 0.343 | 1.17E-15 |
| AC009404.2 | KIAA1524 | 0.343 | 1.19E-15 |
| AC009404.2 | WEE1 | 0.343 | 1.17E-15 |
| AC009404.2 | RP11-166N6.3 | 0.343 | 1.29E-15 |
| AC009404.2 | C12orf73 | 0.343 | 1.16E-15 |
| AC009404.2 | PLK5 | 0.343 | 1.34E-15 |
| AC009404.2 | ZBTB20 | 0.343 | 1.19E-15 |
| AC009404.2 | ZNF571 | 0.343 | 1.34E-15 |
| AC009404.2 | AL596220.1 | 0.344 | 1.10E-15 |
| AC009404.2 | HCG27 | 0.344 | 1.06E-15 |
| AC009404.2 | CEP97 | 0.344 | 1.11E-15 |
| AC009404.2 | BOLL | 0.344 | 9.78E-16 |
| AC009404.2 | PCDHGA8 | 0.344 | 9.59E-16 |
| AC009404.2 | RAB3IP | 0.344 | 1.09E-15 |
| AC009404.2 | LATS1 | 0.344 | 1.05E-15 |
| AC009404.2 | CCDC36 | 0.344 | 1.04E-15 |
| AC009404.2 | CFLAR | 0.344 | 9.49E-16 |
| AC009404.2 | GPR61 | 0.344 | 1.08E-15 |
| AC009404.2 | THAP2 | 0.344 | 9.21E-16 |
| AC009404.2 | ZNF17 | 0.344 | 9.53E-16 |
| AC009404.2 | SENP7 | 0.344 | 9.84E-16 |
| AC009404.2 | OR9A2 | 0.344 | 1.00E-15 |
| AC009404.2 | PSTK | 0.344 | 9.78E-16 |
| AC009404.2 | ARIH1 | 0.344 | 9.97E-16 |
| AC009404.2 | RP11-371E8.4 | 0.344 | 9.21E-16 |
| AC009404.2 | PRB3 | 0.344 | 1.08E-15 |
| AC009404.2 | UBA6 | 0.344 | 1.03E-15 |
| AC009404.2 | CLEC12B | 0.344 | 9.18E-16 |
| AC009404.2 | GK5 | 0.344 | 9.37E-16 |
| AC009404.2 | KIF21B | 0.345 | 8.39E-16 |
| AC009404.2 | ZNF695 | 0.345 | 8.22E-16 |
| AC009404.2 | IRF2BP2 | 0.345 | 7.62E-16 |
| AC009404.2 | VMA21 | 0.345 | 7.91E-16 |
| AC009404.2 | DTWD1 | 0.345 | 7.96E-16 |
| AC009404.2 | ZZZ3 | 0.345 | 8.18E-16 |
| AC009404.2 | C6orf25 | 0.345 | 8.14E-16 |
| AC009404.2 | ZBTB5 | 0.345 | 8.69E-16 |
| AC009404.2 | OR1Q1 | 0.345 | 7.83E-16 |
| AC009404.2 | ARID3A | 0.345 | 8.09E-16 |
| AC009404.2 | VWA8 | 0.345 | 7.87E-16 |
| AC009404.2 | NVL | 0.345 | 9.07E-16 |
| AC009404.2 | RWDD3 | 0.345 | 8.97E-16 |
| AC009404.2 | AL360294.1 | 0.345 | 7.45E-16 |
| AC009404.2 | USP47 | 0.345 | 7.81E-16 |
| AC009404.2 | RFX8 | 0.345 | 8.76E-16 |
| AC009404.2 | FAM120C | 0.345 | 8.31E-16 |
| AC009404.2 | NRF1 | 0.345 | 7.85E-16 |
| AC009404.2 | AAK1 | 0.346 | 6.15E-16 |
| AC009404.2 | CTD-2006C1.13 | 0.346 | 7.21E-16 |
| AC009404.2 | INTS8 | 0.346 | 6.47E-16 |
| AC009404.2 | SLC7A11 | 0.346 | 7.06E-16 |
| AC009404.2 | HSD17B3 | 0.346 | 6.41E-16 |
| AC009404.2 | AVL9 | 0.346 | 6.90E-16 |
| AC009404.2 | TIAM2 | 0.346 | 6.53E-16 |
| AC009404.2 | ZNF585A | 0.346 | 7.31E-16 |
| AC009404.2 | GLS | 0.346 | 6.80E-16 |
| AC009404.2 | NEBL | 0.346 | 6.67E-16 |
| AC009404.2 | SNTB2 | 0.346 | 6.40E-16 |
| AC009404.2 | ANKRD13D | 0.346 | 6.08E-16 |
| AC009404.2 | ANKS6 | 0.346 | 6.08E-16 |
| AC009404.2 | PAM16 | 0.346 | 6.91E-16 |
| AC009404.2 | MED1 | 0.346 | 7.16E-16 |
| AC009404.2 | SMIM13 | 0.346 | 6.53E-16 |
| AC009404.2 | RAB41 | 0.346 | 6.88E-16 |
| AC009404.2 | CSRP3 | 0.346 | 7.22E-16 |
| AC009404.2 | TAF4 | 0.346 | 6.83E-16 |
| AC009404.2 | G3BP1 | 0.347 | 5.87E-16 |
| AC009404.2 | RNF133 | 0.347 | 5.75E-16 |
| AC009404.2 | RAB22A | 0.347 | 5.74E-16 |
| AC009404.2 | AGK | 0.347 | 6.03E-16 |
| AC009404.2 | TRAF3IP1 | 0.347 | 5.02E-16 |
| AC009404.2 | CHRM5 | 0.347 | 5.10E-16 |
| AC009404.2 | TBRG1 | 0.347 | 5.02E-16 |
| AC009404.2 | ADAM21 | 0.347 | 5.15E-16 |
| AC009404.2 | OR10A5 | 0.347 | 5.07E-16 |
| AC009404.2 | CDK5RAP1 | 0.347 | 5.78E-16 |
| AC009404.2 | ALPK1 | 0.347 | 5.05E-16 |
| AC009404.2 | SPDYE2B | 0.347 | 5.06E-16 |
| AC009404.2 | TMEM78 | 0.347 | 5.63E-16 |
| AC009404.2 | ZNF697 | 0.347 | 5.37E-16 |
| AC009404.2 | OR1J1 | 0.347 | 5.65E-16 |
| AC009404.2 | ACP6 | 0.347 | 5.96E-16 |
| AC009404.2 | ZMYM1 | 0.347 | 5.53E-16 |
| AC009404.2 | CCSAP | 0.347 | 4.93E-16 |
| AC009404.2 | HIST4H4 | 0.347 | 5.80E-16 |
| AC009404.2 | EYA3 | 0.347 | 5.69E-16 |
| AC009404.2 | OTUD4 | 0.347 | 5.89E-16 |
| AC009404.2 | TTI1 | 0.347 | 5.38E-16 |
| AC009404.2 | RGL4 | 0.347 | 5.38E-16 |
| AC009404.2 | FER1L5 | 0.347 | 5.27E-16 |
| AC009404.2 | RP11-723O4.6 | 0.347 | 5.23E-16 |
| AC009404.2 | PDS5B | 0.348 | 4.23E-16 |
| AC009404.2 | SMC4 | 0.348 | 4.34E-16 |
| AC009404.2 | SPIDR | 0.348 | 4.10E-16 |
| AC009404.2 | AC026449.1 | 0.348 | 4.34E-16 |
| AC009404.2 | ARID1B | 0.348 | 4.50E-16 |
| AC009404.2 | TRIM59 | 0.348 | 4.49E-16 |
| AC009404.2 | FBXL4 | 0.348 | 4.78E-16 |
| AC009404.2 | NPAT | 0.348 | 4.04E-16 |
| AC009404.2 | MAP4K5 | 0.348 | 4.63E-16 |
| AC009404.2 | ANAPC7 | 0.348 | 4.39E-16 |
| AC009404.2 | AC231657.1 | 0.348 | 4.64E-16 |
| AC009404.2 | TRMT44 | 0.348 | 4.77E-16 |
| AC009404.2 | GPR22 | 0.348 | 4.40E-16 |
| AC009404.2 | BIVM | 0.348 | 4.81E-16 |
| AC009404.2 | TP53BP1 | 0.348 | 4.39E-16 |
| AC009404.2 | ANKRD11 | 0.348 | 4.61E-16 |
| AC009404.2 | MON2 | 0.348 | 4.76E-16 |
| AC009404.2 | ZCCHC4 | 0.348 | 4.03E-16 |
| AC009404.2 | ZDHHC11 | 0.348 | 4.02E-16 |
| AC009404.2 | ZNF860 | 0.348 | 4.69E-16 |
| AC009404.2 | ZNF572 | 0.348 | 4.47E-16 |
| AC009404.2 | OR2B2 | 0.348 | 4.58E-16 |
| AC009404.2 | MYH3 | 0.348 | 4.31E-16 |
| AC009404.2 | PRDM10 | 0.349 | 3.96E-16 |
| AC009404.2 | SYNCRIP | 0.349 | 3.95E-16 |
| AC009404.2 | SNX25 | 0.349 | 3.50E-16 |
| AC009404.2 | C17orf47 | 0.349 | 3.62E-16 |
| AC009404.2 | PIGL | 0.349 | 3.72E-16 |
| AC009404.2 | SNURF | 0.349 | 3.73E-16 |
| AC009404.2 | KPNA5 | 0.349 | 3.29E-16 |
| AC009404.2 | PROSER1 | 0.349 | 3.75E-16 |
| AC009404.2 | FARP2 | 0.349 | 3.64E-16 |
| AC009404.2 | ZNF707 | 0.349 | 3.94E-16 |
| AC009404.2 | WNT3 | 0.349 | 3.44E-16 |
| AC009404.2 | TTC21B | 0.349 | 3.50E-16 |
| AC009404.2 | SOX4 | 0.349 | 3.88E-16 |
| AC009404.2 | FAM156A | 0.349 | 3.37E-16 |
| AC009404.2 | CREB1 | 0.349 | 3.91E-16 |
| AC009404.2 | FBXO11 | 0.349 | 3.87E-16 |
| AC009404.2 | SLC19A2 | 0.349 | 3.47E-16 |
| AC009404.2 | DCP2 | 0.349 | 3.29E-16 |
| AC009404.2 | PTPRG | 0.349 | 3.65E-16 |
| AC009404.2 | C11orf95 | 0.349 | 3.77E-16 |
| AC009404.2 | FHAD1 | 0.35 | 2.76E-16 |
| AC009404.2 | EPB42 | 0.35 | 2.78E-16 |
| AC009404.2 | CCDC168 | 0.35 | 2.78E-16 |
| AC009404.2 | ARFGEF2 | 0.35 | 3.07E-16 |
| AC009404.2 | PRB4 | 0.35 | 2.79E-16 |
| AC009404.2 | FSD1L | 0.35 | 3.04E-16 |
| AC009404.2 | TJAP1 | 0.35 | 2.73E-16 |
| AC009404.2 | STRN | 0.35 | 2.86E-16 |
| AC009404.2 | PRKRIP1 | 0.35 | 2.92E-16 |
| AC009404.2 | ZNF529 | 0.35 | 2.87E-16 |
| AC009404.2 | TRPC5 | 0.35 | 2.76E-16 |
| AC009404.2 | ZC3H6 | 0.35 | 3.22E-16 |
| AC009404.2 | ETAA1 | 0.35 | 2.79E-16 |
| AC009404.2 | XKRX | 0.35 | 3.13E-16 |
| AC009404.2 | DPH7 | 0.351 | 2.29E-16 |
| AC009404.2 | PRICKLE4 | 0.351 | 2.34E-16 |
| AC009404.2 | CLTCL1 | 0.351 | 2.38E-16 |
| AC009404.2 | SLC2A11 | 0.351 | 2.48E-16 |
| AC009404.2 | MAP3K7 | 0.351 | 2.25E-16 |
| AC009404.2 | EFCAB7 | 0.351 | 2.45E-16 |
| AC009404.2 | BAZ1B | 0.351 | 2.22E-16 |
| AC009404.2 | SH3TC2 | 0.351 | 2.32E-16 |
| AC009404.2 | MYO19 | 0.351 | 2.25E-16 |
| AC009404.2 | RP11-12J10.3 | 0.351 | 2.64E-16 |
| AC009404.2 | SYCP2 | 0.351 | 2.30E-16 |
| AC009404.2 | FAM196B | 0.351 | 2.59E-16 |
| AC009404.2 | ZHX1-C8orf76 | 0.351 | 2.31E-16 |
| AC009404.2 | WDR66 | 0.351 | 2.62E-16 |
| AC009404.2 | SAMD7 | 0.351 | 2.48E-16 |
| AC009404.2 | FPGT-TNNI3K | 0.352 | 1.97E-16 |
| AC009404.2 | IKZF4 | 0.352 | 2.14E-16 |
| AC009404.2 | CRYBB3 | 0.352 | 2.14E-16 |
| AC009404.2 | STC2 | 0.352 | 1.86E-16 |
| AC009404.2 | RIC1 | 0.352 | 1.97E-16 |
| AC009404.2 | TIMM8A | 0.352 | 1.81E-16 |
| AC009404.2 | HIGD1C | 0.352 | 1.93E-16 |
| AC009404.2 | SLX4 | 0.352 | 1.82E-16 |
| AC009404.2 | NHLRC3 | 0.352 | 2.08E-16 |
| AC009404.2 | CSTL1 | 0.352 | 1.90E-16 |
| AC009404.2 | C1orf146 | 0.352 | 1.86E-16 |
| AC009404.2 | ARG1 | 0.352 | 2.14E-16 |
| AC009404.2 | ZBTB33 | 0.352 | 1.97E-16 |
| AC009404.2 | DHX35 | 0.352 | 1.99E-16 |
| AC009404.2 | PMM2 | 0.352 | 2.01E-16 |
| AC009404.2 | HNRNPA3 | 0.352 | 2.02E-16 |
| AC009404.2 | STARD9 | 0.352 | 2.03E-16 |
| AC009404.2 | CALML6 | 0.352 | 2.01E-16 |
| AC009404.2 | METTL10 | 0.352 | 2.03E-16 |
| AC009404.2 | MLLT6 | 0.353 | 1.71E-16 |
| AC009404.2 | FAM92A1 | 0.353 | 1.64E-16 |
| AC009404.2 | ZBTB2 | 0.353 | 1.60E-16 |
| AC009404.2 | LPIN3 | 0.353 | 1.56E-16 |
| AC009404.2 | FMR1 | 0.353 | 1.52E-16 |
| AC009404.2 | DHX15 | 0.353 | 1.69E-16 |
| AC009404.2 | RP5-966M1.6 | 0.353 | 1.45E-16 |
| AC009404.2 | MANSC4 | 0.353 | 1.49E-16 |
| AC009404.2 | RUNX1 | 0.353 | 1.44E-16 |
| AC009404.2 | ALG11 | 0.353 | 1.63E-16 |
| AC009404.2 | ZNF391 | 0.353 | 1.48E-16 |
| AC009404.2 | GTF3C4 | 0.353 | 1.57E-16 |
| AC009404.2 | GSE1 | 0.353 | 1.53E-16 |
| AC009404.2 | AC110615.1 | 0.353 | 1.67E-16 |
| AC009404.2 | AL139333.1 | 0.353 | 1.55E-16 |
| AC009404.2 | AMIGO3 | 0.353 | 1.46E-16 |
| AC009404.2 | HERC4 | 0.353 | 1.60E-16 |
| AC009404.2 | GTPBP4 | 0.354 | 1.29E-16 |
| AC009404.2 | SMIM18 | 0.354 | 1.21E-16 |
| AC009404.2 | AC069063.2 | 0.354 | 1.23E-16 |
| AC009404.2 | CABYR | 0.354 | 1.21E-16 |
| AC009404.2 | ZC3H13 | 0.354 | 1.36E-16 |
| AC009404.2 | SNAPC3 | 0.354 | 1.21E-16 |
| AC009404.2 | PSMC3IP | 0.354 | 1.41E-16 |
| AC009404.2 | CACNA1D | 0.354 | 1.26E-16 |
| AC009404.2 | MC1R | 0.354 | 1.23E-16 |
| AC009404.2 | TBC1D4 | 0.354 | 1.39E-16 |
| AC009404.2 | ZBTB11 | 0.354 | 1.31E-16 |
| AC009404.2 | ZNF680 | 0.355 | 9.69E-17 |
| AC009404.2 | TRH | 0.355 | 9.56E-17 |
| AC009404.2 | REC8 | 0.355 | 1.08E-16 |
| AC009404.2 | ZNF320 | 0.355 | 9.56E-17 |
| AC009404.2 | ZCCHC6 | 0.355 | 1.11E-16 |
| AC009404.2 | USP35 | 0.355 | 1.01E-16 |
| AC009404.2 | UPF2 | 0.355 | 1.10E-16 |
| AC009404.2 | SPDL1 | 0.355 | 9.34E-17 |
| AC009404.2 | EZH2 | 0.355 | 1.03E-16 |
| AC009404.2 | FAM133B | 0.355 | 1.01E-16 |
| AC009404.2 | MTPAP | 0.355 | 1.01E-16 |
| AC009404.2 | EMSY | 0.355 | 9.41E-17 |
| AC009404.2 | GRPEL2 | 0.355 | 9.73E-17 |
| AC009404.2 | IQCC | 0.356 | 8.52E-17 |
| AC009404.2 | USP42 | 0.356 | 8.76E-17 |
| AC009404.2 | RRP8 | 0.356 | 8.51E-17 |
| AC009404.2 | GOLGB1 | 0.356 | 8.35E-17 |
| AC009404.2 | HNRNPU | 0.356 | 7.58E-17 |
| AC009404.2 | PRSS55 | 0.356 | 8.45E-17 |
| AC009404.2 | TBC1D32 | 0.356 | 8.24E-17 |
| AC009404.2 | OR10A2 | 0.356 | 9.05E-17 |
| AC009404.2 | PBOV1 | 0.356 | 9.07E-17 |
| AC009404.2 | ATXN3 | 0.356 | 8.26E-17 |
| AC009404.2 | NPIPB11 | 0.356 | 8.93E-17 |
| AC009404.2 | SETDB2 | 0.357 | 6.72E-17 |
| AC009404.2 | ZNF281 | 0.357 | 6.29E-17 |
| AC009404.2 | MOB1B | 0.357 | 6.79E-17 |
| AC009404.2 | SPIN2A | 0.357 | 6.61E-17 |
| AC009404.2 | ACIN1 | 0.357 | 6.27E-17 |
| AC009404.2 | KRTAP5-1 | 0.357 | 6.40E-17 |
| AC009404.2 | DOCK6 | 0.357 | 7.38E-17 |
| AC009404.2 | AGO4 | 0.357 | 6.50E-17 |
| AC009404.2 | KRR1 | 0.357 | 6.99E-17 |
| AC009404.2 | B3GNTL1 | 0.357 | 6.42E-17 |
| AC009404.2 | POLA1 | 0.357 | 7.36E-17 |
| AC009404.2 | ZNF335 | 0.357 | 7.36E-17 |
| AC009404.2 | FXR1 | 0.357 | 7.47E-17 |
| AC009404.2 | AP3M2 | 0.358 | 5.53E-17 |
| AC009404.2 | C2orf44 | 0.358 | 5.80E-17 |
| AC009404.2 | CCDC40 | 0.358 | 5.83E-17 |
| AC009404.2 | ZNF557 | 0.358 | 6.07E-17 |
| AC009404.2 | ANGPT2 | 0.358 | 5.78E-17 |
| AC009404.2 | TMEM91 | 0.358 | 5.86E-17 |
| AC009404.2 | SMC6 | 0.358 | 6.06E-17 |
| AC009404.2 | ZRANB2 | 0.358 | 5.17E-17 |
| AC009404.2 | SOCS4 | 0.358 | 5.80E-17 |
| AC009404.2 | AC242988.1 | 0.358 | 5.45E-17 |
| AC009404.2 | AEBP2 | 0.358 | 5.32E-17 |
| AC009404.2 | NOL10 | 0.358 | 5.48E-17 |
| AC009404.2 | C1orf112 | 0.358 | 5.26E-17 |
| AC009404.2 | ODF2 | 0.358 | 5.54E-17 |
| AC009404.2 | ZNF765 | 0.358 | 5.72E-17 |
| AC009404.2 | TPRG1 | 0.358 | 5.22E-17 |
| AC009404.2 | AC016577.1 | 0.359 | 4.02E-17 |
| AC009404.2 | ZFP90 | 0.359 | 4.28E-17 |
| AC009404.2 | TTC26 | 0.359 | 4.41E-17 |
| AC009404.2 | UBFD1 | 0.359 | 4.38E-17 |
| AC009404.2 | STPG2 | 0.359 | 4.75E-17 |
| AC009404.2 | PATE1 | 0.359 | 4.89E-17 |
| AC009404.2 | NUP210L | 0.359 | 4.31E-17 |
| AC009404.2 | DDX26B | 0.359 | 4.30E-17 |
| AC009404.2 | BICD2 | 0.359 | 4.14E-17 |
| AC009404.2 | PRELID3A | 0.359 | 4.25E-17 |
| AC009404.2 | ZNF597 | 0.359 | 4.81E-17 |
| AC009404.2 | C7orf25 | 0.359 | 4.16E-17 |
| AC009404.2 | C9orf47 | 0.359 | 4.73E-17 |
| AC009404.2 | GMPS | 0.359 | 4.36E-17 |
| AC009404.2 | PNPT1 | 0.359 | 4.60E-17 |
| AC009404.2 | PCNXL4 | 0.36 | 3.67E-17 |
| AC009404.2 | ZNF284 | 0.36 | 3.44E-17 |
| AC009404.2 | GATAD2B | 0.36 | 3.22E-17 |
| AC009404.2 | VHL | 0.36 | 3.49E-17 |
| AC009404.2 | SLC35F4 | 0.36 | 3.67E-17 |
| AC009404.2 | ANGPTL3 | 0.36 | 3.39E-17 |
| AC009404.2 | ANKRD17 | 0.36 | 3.31E-17 |
| AC009404.2 | CRHR2 | 0.36 | 3.92E-17 |
| AC009404.2 | C12orf40 | 0.36 | 3.53E-17 |
| AC009404.2 | ZNF267 | 0.36 | 3.69E-17 |
| AC009404.2 | PLXNA3 | 0.36 | 3.49E-17 |
| AC009404.2 | ATP11C | 0.36 | 3.53E-17 |
| AC009404.2 | CLEC18B | 0.36 | 3.52E-17 |
| AC009404.2 | TNRC6B | 0.36 | 3.60E-17 |
| AC009404.2 | ZC3HAV1L | 0.36 | 3.32E-17 |
| AC009404.2 | PPFIA4 | 0.36 | 3.62E-17 |
| AC009404.2 | PCNX | 0.36 | 3.35E-17 |
| AC009404.2 | ZNF263 | 0.36 | 3.41E-17 |
| AC009404.2 | NUP58 | 0.36 | 3.41E-17 |
| AC009404.2 | TNPO2 | 0.36 | 3.65E-17 |
| AC009404.2 | PRSS54 | 0.361 | 2.63E-17 |
| AC009404.2 | BARD1 | 0.361 | 3.20E-17 |
| AC009404.2 | NEIL1 | 0.361 | 2.86E-17 |
| AC009404.2 | SLC4A7 | 0.361 | 2.78E-17 |
| AC009404.2 | JMY | 0.361 | 2.89E-17 |
| AC009404.2 | POLI | 0.361 | 2.96E-17 |
| AC009404.2 | LY6G6F | 0.361 | 2.60E-17 |
| AC009404.2 | CNOT2 | 0.361 | 2.77E-17 |
| AC009404.2 | NAB1 | 0.361 | 2.77E-17 |
| AC009404.2 | FO538757.2 | 0.361 | 2.79E-17 |
| AC009404.2 | SMYD3 | 0.361 | 3.17E-17 |
| AC009404.2 | ZNF711 | 0.361 | 3.08E-17 |
| AC009404.2 | HIVEP1 | 0.362 | 2.17E-17 |
| AC009404.2 | TAS2R30 | 0.362 | 2.45E-17 |
| AC009404.2 | BCL2L2-PABPN1 | 0.362 | 2.45E-17 |
| AC009404.2 | SPEN | 0.362 | 2.50E-17 |
| AC009404.2 | PRSS48 | 0.362 | 2.58E-17 |
| AC009404.2 | USF3 | 0.362 | 2.51E-17 |
| AC009404.2 | TAS2R13 | 0.362 | 2.15E-17 |
| AC009404.2 | CAMSAP1 | 0.362 | 2.09E-17 |
| AC009404.2 | UTP14A | 0.362 | 2.12E-17 |
| AC009404.2 | PIWIL4 | 0.362 | 2.54E-17 |
| AC009404.2 | INTS2 | 0.362 | 2.37E-17 |
| AC009404.2 | LCTL | 0.363 | 1.89E-17 |
| AC009404.2 | RPAP2 | 0.363 | 1.74E-17 |
| AC009404.2 | CREB5 | 0.363 | 1.93E-17 |
| AC009404.2 | TRIM39 | 0.363 | 1.88E-17 |
| AC009404.2 | PLEKHA3 | 0.363 | 1.82E-17 |
| AC009404.2 | RP5-1052I5.2 | 0.363 | 1.69E-17 |
| AC009404.2 | SIM2 | 0.363 | 1.87E-17 |
| AC009404.2 | NLRP14 | 0.363 | 1.98E-17 |
| AC009404.2 | MURC | 0.363 | 1.68E-17 |
| AC009404.2 | ENGASE | 0.363 | 2.01E-17 |
| AC009404.2 | COBLL1 | 0.363 | 1.70E-17 |
| AC009404.2 | GNRHR | 0.363 | 1.82E-17 |
| AC009404.2 | SHROOM4 | 0.363 | 2.04E-17 |
| AC009404.2 | SAMD8 | 0.363 | 2.07E-17 |
| AC009404.2 | POLR1A | 0.363 | 1.75E-17 |
| AC009404.2 | ROCK2 | 0.363 | 2.05E-17 |
| AC009404.2 | AL049794.1 | 0.363 | 2.07E-17 |
| AC009404.2 | AC009022.1 | 0.363 | 1.70E-17 |
| AC009404.2 | COPS7B | 0.363 | 1.99E-17 |
| AC009404.2 | TUBGCP4 | 0.363 | 1.76E-17 |
| AC009404.2 | C1QTNF3-AMACR | 0.363 | 1.90E-17 |
| AC009404.2 | TEAD1 | 0.364 | 1.61E-17 |
| AC009404.2 | PXYLP1 | 0.364 | 1.62E-17 |
| AC009404.2 | ESF1 | 0.364 | 1.63E-17 |
| AC009404.2 | ICA1L | 0.364 | 1.41E-17 |
| AC009404.2 | NSD1 | 0.364 | 1.57E-17 |
| AC009404.2 | AC073130.2 | 0.364 | 1.52E-17 |
| AC009404.2 | AC069063.1 | 0.364 | 1.57E-17 |
| AC009404.2 | PHF6 | 0.364 | 1.65E-17 |
| AC009404.2 | UTP15 | 0.364 | 1.58E-17 |
| AC009404.2 | U51561.1 | 0.364 | 1.66E-17 |
| AC009404.2 | ASAH2B | 0.364 | 1.41E-17 |
| AC009404.2 | ZFHX3 | 0.364 | 1.42E-17 |
| AC009404.2 | CTD-3088G3.8 | 0.364 | 1.40E-17 |
| AC009404.2 | ZNF141 | 0.364 | 1.55E-17 |
| AC009404.2 | PHF8 | 0.364 | 1.40E-17 |
| AC009404.2 | MARK3 | 0.364 | 1.46E-17 |
| AC009404.2 | EFCAB6 | 0.364 | 1.56E-17 |
| AC009404.2 | KLHL15 | 0.364 | 1.59E-17 |
| AC009404.2 | MPP4 | 0.365 | 1.19E-17 |
| AC009404.2 | ZNF808 | 0.365 | 1.24E-17 |
| AC009404.2 | USP6 | 0.365 | 1.16E-17 |
| AC009404.2 | TRIM33 | 0.365 | 1.10E-17 |
| AC009404.2 | RP5-862P8.2 | 0.365 | 1.30E-17 |
| AC009404.2 | TUBA8 | 0.365 | 1.09E-17 |
| AC009404.2 | ZCCHC8 | 0.365 | 1.22E-17 |
| AC009404.2 | DZANK1 | 0.365 | 1.10E-17 |
| AC009404.2 | PTBP2 | 0.365 | 1.13E-17 |
| AC009404.2 | RP11-231C14.4 | 0.365 | 1.32E-17 |
| AC009404.2 | AC005042.1 | 0.365 | 1.12E-17 |
| AC009404.2 | TMEM260 | 0.365 | 1.15E-17 |
| AC009404.2 | UIMC1 | 0.365 | 1.24E-17 |
| AC009404.2 | NUP155 | 0.365 | 1.11E-17 |
| AC009404.2 | LHCGR | 0.365 | 1.13E-17 |
| AC009404.2 | C1QTNF9B | 0.365 | 1.09E-17 |
| AC009404.2 | CEMIP | 0.365 | 1.28E-17 |
| AC009404.2 | NEB | 0.365 | 1.16E-17 |
| AC009404.2 | ULK4 | 0.365 | 1.12E-17 |
| AC009404.2 | FNIP1 | 0.365 | 1.23E-17 |
| AC009404.2 | IZUMO1 | 0.365 | 1.28E-17 |
| AC009404.2 | KIAA1147 | 0.366 | 9.70E-18 |
| AC009404.2 | LARP4 | 0.366 | 1.02E-17 |
| AC009404.2 | FAM91A1 | 0.366 | 8.99E-18 |
| AC009404.2 | WNT8B | 0.366 | 9.04E-18 |
| AC009404.2 | DNASE1 | 0.366 | 1.07E-17 |
| AC009404.2 | SUPT3H | 0.366 | 1.05E-17 |
| AC009404.2 | ATP6V1C2 | 0.366 | 1.04E-17 |
| AC009404.2 | SETDB1 | 0.366 | 1.00E-17 |
| AC009404.2 | AL365202.1 | 0.366 | 9.01E-18 |
| AC009404.2 | POLR3E | 0.366 | 1.05E-17 |
| AC009404.2 | KCNH7 | 0.366 | 9.91E-18 |
| AC009404.2 | PKP4 | 0.366 | 1.03E-17 |
| AC009404.2 | MITD1 | 0.366 | 1.09E-17 |
| AC009404.2 | LSG1 | 0.366 | 9.76E-18 |
| AC009404.2 | EIF2AK2 | 0.366 | 8.78E-18 |
| AC009404.2 | IQCH | 0.366 | 9.20E-18 |
| AC009404.2 | DHX36 | 0.367 | 7.19E-18 |
| AC009404.2 | MYBL1 | 0.367 | 7.01E-18 |
| AC009404.2 | PLGLB1 | 0.367 | 7.84E-18 |
| AC009404.2 | CELSR1 | 0.367 | 8.69E-18 |
| AC009404.2 | KRBOX4 | 0.367 | 8.19E-18 |
| AC009404.2 | BBOX1 | 0.367 | 7.40E-18 |
| AC009404.2 | JARID2 | 0.367 | 7.66E-18 |
| AC009404.2 | ARID4B | 0.367 | 7.32E-18 |
| AC009404.2 | CCDC142 | 0.367 | 8.58E-18 |
| AC009404.2 | RALGAPB | 0.367 | 7.92E-18 |
| AC009404.2 | DHX57 | 0.367 | 8.43E-18 |
| AC009404.2 | VCPKMT | 0.368 | 6.92E-18 |
| AC009404.2 | PTPN12 | 0.368 | 6.25E-18 |
| AC009404.2 | PUM2 | 0.368 | 6.18E-18 |
| AC009404.2 | FAM72A | 0.368 | 5.82E-18 |
| AC009404.2 | MED13L | 0.368 | 6.20E-18 |
| AC009404.2 | PGBD4 | 0.368 | 5.79E-18 |
| AC009404.2 | RNF219 | 0.368 | 5.75E-18 |
| AC009404.2 | RP11-248J23.7 | 0.368 | 5.70E-18 |
| AC009404.2 | TSACC | 0.368 | 6.10E-18 |
| AC009404.2 | C10orf131 | 0.368 | 6.18E-18 |
| AC009404.2 | ZMYM5 | 0.368 | 5.64E-18 |
| AC009404.2 | CDSN | 0.368 | 5.87E-18 |
| AC009404.2 | URB1 | 0.368 | 6.37E-18 |
| AC009404.2 | FAM71D | 0.368 | 6.57E-18 |
| AC009404.2 | TAS2R46 | 0.369 | 4.70E-18 |
| AC009404.2 | RAPGEF6 | 0.369 | 5.10E-18 |
| AC009404.2 | CHML | 0.369 | 4.96E-18 |
| AC009404.2 | SPATA25 | 0.369 | 4.71E-18 |
| AC009404.2 | CDK6 | 0.369 | 5.50E-18 |
| AC009404.2 | ZNF594 | 0.369 | 4.78E-18 |
| AC009404.2 | CCDC17 | 0.369 | 4.57E-18 |
| AC009404.2 | PTPN11 | 0.369 | 4.64E-18 |
| AC009404.2 | TMEM110-MUSTN1 | 0.369 | 5.48E-18 |
| AC009404.2 | STX6 | 0.369 | 5.20E-18 |
| AC009404.2 | FAM196A | 0.369 | 5.45E-18 |
| AC009404.2 | EME1 | 0.369 | 5.31E-18 |
| AC009404.2 | FANCI | 0.369 | 5.61E-18 |
| AC009404.2 | DEFB108B | 0.37 | 3.96E-18 |
| AC009404.2 | DNA2 | 0.37 | 3.87E-18 |
| AC009404.2 | TAS2R43 | 0.37 | 3.85E-18 |
| AC009404.2 | SNTB1 | 0.37 | 3.67E-18 |
| AC009404.2 | POU5F2 | 0.37 | 4.11E-18 |
| AC009404.2 | ANKHD1-EIF4EBP3 | 0.37 | 4.31E-18 |
| AC009404.2 | ARL17B | 0.37 | 3.76E-18 |
| AC009404.2 | TRA2A | 0.37 | 3.86E-18 |
| AC009404.2 | ZNF239 | 0.37 | 3.85E-18 |
| AC009404.2 | ZMYM6 | 0.37 | 3.97E-18 |
| AC009404.2 | E2F6 | 0.37 | 3.77E-18 |
| AC009404.2 | DNAH7 | 0.37 | 4.13E-18 |
| AC009404.2 | CUL9 | 0.37 | 4.29E-18 |
| AC009404.2 | PRPF40A | 0.37 | 3.83E-18 |
| AC009404.2 | AC100821.2 | 0.371 | 2.94E-18 |
| AC009404.2 | AC011380.1 | 0.371 | 3.54E-18 |
| AC009404.2 | CCDC15 | 0.371 | 3.05E-18 |
| AC009404.2 | AC079907.1 | 0.371 | 3.44E-18 |
| AC009404.2 | RNF113B | 0.371 | 3.02E-18 |
| AC009404.2 | ZNF266 | 0.371 | 3.37E-18 |
| AC009404.2 | BLM | 0.371 | 3.31E-18 |
| AC009404.2 | CYB5RL | 0.371 | 3.37E-18 |
| AC009404.2 | CBWD1 | 0.371 | 3.45E-18 |
| AC009404.2 | NIPBL | 0.371 | 2.95E-18 |
| AC009404.2 | MAPKAPK5 | 0.371 | 2.96E-18 |
| AC009404.2 | WDR88 | 0.371 | 3.47E-18 |
| AC009404.2 | PATE4 | 0.371 | 3.28E-18 |
| AC009404.2 | USP6NL | 0.371 | 3.25E-18 |
| AC009404.2 | NBPF10 | 0.371 | 3.26E-18 |
| AC009404.2 | SBF2 | 0.371 | 3.38E-18 |
| AC009404.2 | NCBP2 | 0.371 | 2.91E-18 |
| AC009404.2 | NFYA | 0.372 | 2.85E-18 |
| AC009404.2 | PPRC1 | 0.372 | 2.63E-18 |
| AC009404.2 | EPT1 | 0.372 | 2.38E-18 |
| AC009404.2 | WDFY3 | 0.372 | 2.48E-18 |
| AC009404.2 | SLC25A2 | 0.372 | 2.60E-18 |
| AC009404.2 | GDPD4 | 0.372 | 2.79E-18 |
| AC009404.2 | XPO1 | 0.372 | 2.36E-18 |
| AC009404.2 | NOTCH2NL | 0.372 | 2.32E-18 |
| AC009404.2 | LRRD1 | 0.372 | 2.78E-18 |
| AC009404.2 | RP3-382I10.7 | 0.372 | 2.38E-18 |
| AC009404.2 | CPT1B | 0.372 | 2.79E-18 |
| AC009404.2 | RP11-111H13.1 | 0.372 | 2.58E-18 |
| AC009404.2 | CAMSAP2 | 0.372 | 2.83E-18 |
| AC009404.2 | AC012363.2 | 0.372 | 2.38E-18 |
| AC009404.2 | GPR52 | 0.372 | 2.43E-18 |
| AC009404.2 | MKLN1 | 0.372 | 2.69E-18 |
| AC009404.2 | APLF | 0.372 | 2.73E-18 |
| AC009404.2 | WDR59 | 0.372 | 2.34E-18 |
| AC009404.2 | MYO9A | 0.373 | 2.31E-18 |
| AC009404.2 | FAM72D | 0.373 | 2.01E-18 |
| AC009404.2 | AC016549.1 | 0.373 | 1.96E-18 |
| AC009404.2 | KIF24 | 0.373 | 2.17E-18 |
| AC009404.2 | SEC16B | 0.373 | 1.88E-18 |
| AC009404.2 | LIG3 | 0.373 | 2.22E-18 |
| AC009404.2 | C2orf49 | 0.373 | 2.24E-18 |
| AC009404.2 | ALG9 | 0.373 | 2.05E-18 |
| AC009404.2 | WNK3 | 0.373 | 2.16E-18 |
| AC009404.2 | NUP107 | 0.373 | 2.02E-18 |
| AC009404.2 | ZNHIT6 | 0.373 | 2.27E-18 |
| AC009404.2 | GTF2H4 | 0.373 | 1.92E-18 |
| AC009404.2 | NEMF | 0.373 | 2.19E-18 |
| AC009404.2 | MED13 | 0.373 | 2.12E-18 |
| AC009404.2 | MPHOSPH9 | 0.373 | 2.19E-18 |
| AC009404.2 | RBM4 | 0.373 | 1.96E-18 |
| AC009404.2 | TGIF2 | 0.374 | 1.78E-18 |
| AC009404.2 | GCFC2 | 0.374 | 1.74E-18 |
| AC009404.2 | RAG2 | 0.374 | 1.55E-18 |
| AC009404.2 | ATP11A | 0.374 | 1.62E-18 |
| AC009404.2 | LDHAL6A | 0.374 | 1.53E-18 |
| AC009404.2 | SCLT1 | 0.374 | 1.62E-18 |
| AC009404.2 | SYCP2L | 0.374 | 1.52E-18 |
| AC009404.2 | ZNF318 | 0.374 | 1.49E-18 |
| AC009404.2 | PPIG | 0.374 | 1.57E-18 |
| AC009404.2 | ALS2CL | 0.374 | 1.67E-18 |
| AC009404.2 | RSPH4A | 0.375 | 1.33E-18 |
| AC009404.2 | GAPVD1 | 0.375 | 1.41E-18 |
| AC009404.2 | ZNF302 | 0.375 | 1.42E-18 |
| AC009404.2 | AMMECR1 | 0.375 | 1.45E-18 |
| AC009404.2 | SSH2 | 0.375 | 1.25E-18 |
| AC009404.2 | MYLK4 | 0.375 | 1.38E-18 |
| AC009404.2 | FANCM | 0.375 | 1.40E-18 |
| AC009404.2 | RNF32 | 0.375 | 1.30E-18 |
| AC009404.2 | SLC22A13 | 0.375 | 1.30E-18 |
| AC009404.2 | ENTHD2 | 0.375 | 1.47E-18 |
| AC009404.2 | MDC1 | 0.375 | 1.32E-18 |
| AC009404.2 | YJEFN3 | 0.375 | 1.42E-18 |
| AC009404.2 | CCDC173 | 0.375 | 1.28E-18 |
| AC009404.2 | PPAT | 0.375 | 1.25E-18 |
| AC009404.2 | TAS2R42 | 0.375 | 1.38E-18 |
| AC009404.2 | VCPIP1 | 0.375 | 1.27E-18 |
| AC009404.2 | ANLN | 0.375 | 1.32E-18 |
| AC009404.2 | KIF20B | 0.375 | 1.24E-18 |
| AC009404.2 | ZBTB34 | 0.376 | 9.41E-19 |
| AC009404.2 | TUBGCP6 | 0.376 | 1.12E-18 |
| AC009404.2 | ANGEL2 | 0.376 | 9.76E-19 |
| AC009404.2 | ZBTB40 | 0.376 | 1.09E-18 |
| AC009404.2 | ORC6 | 0.376 | 9.65E-19 |
| AC009404.2 | ACMSD | 0.376 | 1.08E-18 |
| AC009404.2 | CPNE7 | 0.376 | 9.53E-19 |
| AC009404.2 | SOGA1 | 0.376 | 1.07E-18 |
| AC009404.2 | MTHFD1L | 0.376 | 1.13E-18 |
| AC009404.2 | ZNF433 | 0.376 | 1.12E-18 |
| AC009404.2 | JPH1 | 0.376 | 1.13E-18 |
| AC009404.2 | RNF207 | 0.376 | 1.06E-18 |
| AC009404.2 | NR2C1 | 0.376 | 1.14E-18 |
| AC009404.2 | GSK3B | 0.376 | 9.61E-19 |
| AC009404.2 | GDF9 | 0.376 | 1.13E-18 |
| AC009404.2 | TSC22D2 | 0.376 | 9.73E-19 |
| AC009404.2 | DIDO1 | 0.376 | 1.15E-18 |
| AC009404.2 | TAS2R50 | 0.377 | 8.86E-19 |
| AC009404.2 | ATXN7L1 | 0.377 | 8.00E-19 |
| AC009404.2 | ZNF417 | 0.377 | 9.34E-19 |
| AC009404.2 | FAN1 | 0.377 | 8.68E-19 |
| AC009404.2 | ZNF419 | 0.377 | 8.97E-19 |
| AC009404.2 | TRMT13 | 0.377 | 8.72E-19 |
| AC009404.2 | CFAP157 | 0.377 | 9.31E-19 |
| AC009404.2 | ZNF142 | 0.377 | 9.01E-19 |
| AC009404.2 | SUGT1 | 0.377 | 8.14E-19 |
| AC009404.2 | ERVW-1 | 0.377 | 8.97E-19 |
| AC009404.2 | ATXN7L2 | 0.377 | 8.48E-19 |
| AC009404.2 | DNAH11 | 0.377 | 8.78E-19 |
| AC009404.2 | TRRAP | 0.377 | 9.28E-19 |
| AC009404.2 | SCGB2B2 | 0.378 | 6.99E-19 |
| AC009404.2 | CCNT1 | 0.378 | 6.10E-19 |
| AC009404.2 | LARP4B | 0.378 | 7.00E-19 |
| AC009404.2 | USP34 | 0.378 | 6.61E-19 |
| AC009404.2 | FANCL | 0.378 | 6.38E-19 |
| AC009404.2 | FIGNL1 | 0.378 | 6.21E-19 |
| AC009404.2 | TNFRSF25 | 0.378 | 6.97E-19 |
| AC009404.2 | AL357140.1 | 0.378 | 6.14E-19 |
| AC009404.2 | NEK10 | 0.378 | 6.45E-19 |
| AC009404.2 | PPP1R3E | 0.378 | 7.40E-19 |
| AC009404.2 | TIAL1 | 0.378 | 6.15E-19 |
| AC009404.2 | ATAD2 | 0.378 | 7.03E-19 |
| AC009404.2 | MRPL53 | 0.378 | 6.00E-19 |
| AC009404.2 | ZBED5 | 0.378 | 6.92E-19 |
| AC009404.2 | ERCC6L2 | 0.378 | 6.18E-19 |
| AC009404.2 | C5orf63 | 0.379 | 5.53E-19 |
| AC009404.2 | CCDC62 | 0.379 | 5.23E-19 |
| AC009404.2 | GIGYF2 | 0.379 | 5.05E-19 |
| AC009404.2 | TCF20 | 0.379 | 5.05E-19 |
| AC009404.2 | MTF2 | 0.379 | 5.48E-19 |
| AC009404.2 | LRRC9 | 0.379 | 5.81E-19 |
| AC009404.2 | NEMP2 | 0.379 | 4.99E-19 |
| AC009404.2 | C10orf113 | 0.379 | 5.40E-19 |
| AC009404.2 | CBFA2T2 | 0.379 | 4.85E-19 |
| AC009404.2 | CASP2 | 0.379 | 5.62E-19 |
| AC009404.2 | ACPT | 0.379 | 5.13E-19 |
| AC009404.2 | KCNRG | 0.379 | 5.81E-19 |
| AC009404.2 | LDLRAD3 | 0.379 | 5.41E-19 |
| AC009404.2 | DSTYK | 0.379 | 5.12E-19 |
| AC009404.2 | TMEM266 | 0.379 | 5.31E-19 |
| AC009404.2 | RC3H1 | 0.38 | 4.22E-19 |
| AC009404.2 | APOBEC2 | 0.38 | 4.01E-19 |
| AC009404.2 | GABBR1 | 0.38 | 4.21E-19 |
| AC009404.2 | VPS13C | 0.38 | 4.30E-19 |
| AC009404.2 | GABPB1 | 0.38 | 4.63E-19 |
| AC009404.2 | JMJD1C | 0.38 | 4.49E-19 |
| AC009404.2 | GCM2 | 0.38 | 3.97E-19 |
| AC009404.2 | OR2D3 | 0.38 | 3.79E-19 |
| AC009404.2 | ZACN | 0.38 | 4.31E-19 |
| AC009404.2 | KIAA1551 | 0.38 | 4.40E-19 |
| AC009404.2 | AKAP8 | 0.38 | 4.69E-19 |
| AC009404.2 | TSPAN16 | 0.38 | 4.67E-19 |
| AC009404.2 | KBTBD2 | 0.38 | 4.18E-19 |
| AC009404.2 | KMT2D | 0.38 | 4.29E-19 |
| AC009404.2 | MBD5 | 0.381 | 3.28E-19 |
| AC009404.2 | PIK3C2A | 0.381 | 3.02E-19 |
| AC009404.2 | ZFP69B | 0.381 | 3.31E-19 |
| AC009404.2 | ATXN7 | 0.381 | 3.58E-19 |
| AC009404.2 | FAM228B | 0.381 | 3.15E-19 |
| AC009404.2 | ADCY10 | 0.381 | 3.62E-19 |
| AC009404.2 | SPINT4 | 0.381 | 3.40E-19 |
| AC009404.2 | CCDC138 | 0.381 | 3.18E-19 |
| AC009404.2 | ZNF709 | 0.382 | 2.73E-19 |
| AC009404.2 | WBP2NL | 0.382 | 2.78E-19 |
| AC009404.2 | TANC1 | 0.382 | 2.84E-19 |
| AC009404.2 | ZNF235 | 0.382 | 2.62E-19 |
| AC009404.2 | SLC35B4 | 0.382 | 2.70E-19 |
| AC009404.2 | TTLL2 | 0.382 | 2.49E-19 |
| AC009404.2 | C15orf41 | 0.382 | 2.51E-19 |
| AC009404.2 | GLIPR1L1 | 0.382 | 2.92E-19 |
| AC009404.2 | RANBP2 | 0.382 | 2.71E-19 |
| AC009404.2 | FABP12 | 0.382 | 2.69E-19 |
| AC009404.2 | HPX | 0.382 | 2.72E-19 |
| AC009404.2 | KLHL42 | 0.382 | 2.41E-19 |
| AC009404.2 | TMEM63A | 0.382 | 2.83E-19 |
| AC009404.2 | DNMT3A | 0.382 | 2.97E-19 |
| AC009404.2 | FOXK1 | 0.383 | 2.23E-19 |
| AC009404.2 | CEP78 | 0.383 | 2.23E-19 |
| AC009404.2 | CCDC38 | 0.383 | 1.98E-19 |
| AC009404.2 | SASS6 | 0.383 | 2.25E-19 |
| AC009404.2 | AC110602.1 | 0.383 | 1.96E-19 |
| AC009404.2 | AJUBA | 0.383 | 2.21E-19 |
| AC009404.2 | RP11-552F3.12 | 0.383 | 2.14E-19 |
| AC009404.2 | ANKUB1 | 0.383 | 2.01E-19 |
| AC009404.2 | TEX22 | 0.383 | 2.02E-19 |
| AC009404.2 | ZNF66 | 0.383 | 1.88E-19 |
| AC009404.2 | ACSM6 | 0.383 | 1.92E-19 |
| AC009404.2 | NOD1 | 0.383 | 2.12E-19 |
| AC009404.2 | ZKSCAN1 | 0.383 | 1.95E-19 |
| AC009404.2 | C4orf45 | 0.383 | 2.09E-19 |
| AC009404.2 | REC114 | 0.383 | 2.29E-19 |
| AC009404.2 | PDCD11 | 0.383 | 2.28E-19 |
| AC009404.2 | PUS7 | 0.383 | 2.12E-19 |
| AC009404.2 | NRBP2 | 0.384 | 1.62E-19 |
| AC009404.2 | ZNF713 | 0.384 | 1.77E-19 |
| AC009404.2 | REL | 0.384 | 1.55E-19 |
| AC009404.2 | ZMAT1 | 0.384 | 1.63E-19 |
| AC009404.2 | TMC3 | 0.384 | 1.64E-19 |
| AC009404.2 | NUDT13 | 0.384 | 1.87E-19 |
| AC009404.2 | TEC | 0.384 | 1.72E-19 |
| AC009404.2 | ZNF490 | 0.384 | 1.63E-19 |
| AC009404.2 | AC024060.1 | 0.384 | 1.66E-19 |
| AC009404.2 | SLX4IP | 0.384 | 1.68E-19 |
| AC009404.2 | LARP1 | 0.385 | 1.35E-19 |
| AC009404.2 | KIAA1919 | 0.385 | 1.46E-19 |
| AC009404.2 | KDM7A | 0.385 | 1.30E-19 |
| AC009404.2 | C9orf84 | 0.385 | 1.30E-19 |
| AC009404.2 | MMP21 | 0.385 | 1.43E-19 |
| AC009404.2 | TTBK2 | 0.385 | 1.36E-19 |
| AC009404.2 | AMER1 | 0.385 | 1.44E-19 |
| AC009404.2 | ARHGEF7 | 0.385 | 1.25E-19 |
| AC009404.2 | ZBTB44 | 0.385 | 1.37E-19 |
| AC009404.2 | ZNF226 | 0.385 | 1.44E-19 |
| AC009404.2 | NUPL2 | 0.386 | 1.15E-19 |
| AC009404.2 | GS1-393G12.13 | 0.386 | 9.83E-20 |
| AC009404.2 | C9orf131 | 0.386 | 9.76E-20 |
| AC009404.2 | TTC23L | 0.386 | 1.05E-19 |
| AC009404.2 | ALS2CR11 | 0.386 | 1.14E-19 |
| AC009404.2 | TRAF1 | 0.386 | 9.99E-20 |
| AC009404.2 | ZNF221 | 0.386 | 1.02E-19 |
| AC009404.2 | E2F5 | 0.386 | 9.78E-20 |
| AC009404.2 | C8orf44-SGK3 | 0.386 | 9.63E-20 |
| AC009404.2 | ZNF19 | 0.386 | 9.46E-20 |
| AC009404.2 | MSL1 | 0.386 | 9.38E-20 |
| AC009404.2 | PALB2 | 0.386 | 9.55E-20 |
| AC009404.2 | AC010287.1 | 0.386 | 1.02E-19 |
| AC009404.2 | LBHD1 | 0.387 | 7.49E-20 |
| AC009404.2 | FBXO41 | 0.387 | 7.78E-20 |
| AC009404.2 | KDM3A | 0.387 | 8.69E-20 |
| AC009404.2 | NPHP3-ACAD11 | 0.387 | 7.79E-20 |
| AC009404.2 | SUN1 | 0.387 | 7.92E-20 |
| AC009404.2 | RAD18 | 0.387 | 7.35E-20 |
| AC009404.2 | FKTN | 0.387 | 7.77E-20 |
| AC009404.2 | FBXL2 | 0.387 | 9.13E-20 |
| AC009404.2 | REPS1 | 0.387 | 8.18E-20 |
| AC009404.2 | CFAP100 | 0.387 | 8.08E-20 |
| AC009404.2 | ZGLP1 | 0.388 | 5.95E-20 |
| AC009404.2 | FCHSD1 | 0.388 | 7.05E-20 |
| AC009404.2 | ASH1L | 0.388 | 6.38E-20 |
| AC009404.2 | FABP9 | 0.388 | 7.02E-20 |
| AC009404.2 | ACYP1 | 0.388 | 7.25E-20 |
| AC009404.2 | SENP6 | 0.388 | 7.29E-20 |
| AC009404.2 | PROCA1 | 0.388 | 6.59E-20 |
| AC009404.2 | AC092881.1 | 0.388 | 5.87E-20 |
| AC009404.2 | PBX4 | 0.388 | 6.23E-20 |
| AC009404.2 | TAF7L | 0.388 | 6.44E-20 |
| AC009404.2 | ZNF146 | 0.388 | 7.31E-20 |
| AC009404.2 | CEP350 | 0.388 | 6.50E-20 |
| AC009404.2 | CKAP5 | 0.388 | 6.97E-20 |
| AC009404.2 | CARD8 | 0.388 | 5.86E-20 |
| AC009404.2 | VAMP1 | 0.388 | 6.14E-20 |
| AC009404.2 | TEX29 | 0.389 | 4.63E-20 |
| AC009404.2 | ANKRD49 | 0.389 | 4.74E-20 |
| AC009404.2 | MACF1 | 0.389 | 5.23E-20 |
| AC009404.2 | APBB3 | 0.389 | 5.00E-20 |
| AC009404.2 | NUDT3 | 0.389 | 5.46E-20 |
| AC009404.2 | ERMARD | 0.389 | 5.00E-20 |
| AC009404.2 | ATP13A3 | 0.389 | 5.42E-20 |
| AC009404.2 | FANCA | 0.389 | 5.55E-20 |
| AC009404.2 | RASAL2 | 0.389 | 5.76E-20 |
| AC009404.2 | WEE2 | 0.389 | 4.78E-20 |
| AC009404.2 | TAOK1 | 0.389 | 5.11E-20 |
| AC009404.2 | SNX15 | 0.389 | 5.34E-20 |
| AC009404.2 | CTC-487M23.8 | 0.389 | 4.96E-20 |
| AC009404.2 | ASXL2 | 0.389 | 5.38E-20 |
| AC009404.2 | TMEM161B | 0.39 | 4.22E-20 |
| AC009404.2 | IFRD1 | 0.39 | 3.87E-20 |
| AC009404.2 | CCDC78 | 0.39 | 4.05E-20 |
| AC009404.2 | LIME1 | 0.39 | 3.72E-20 |
| AC009404.2 | CLCN6 | 0.39 | 4.52E-20 |
| AC009404.2 | JADE3 | 0.39 | 4.12E-20 |
| AC009404.2 | ZCCHC7 | 0.39 | 3.82E-20 |
| AC009404.2 | DNMT3B | 0.39 | 4.26E-20 |
| AC009404.2 | CEP72 | 0.39 | 4.18E-20 |
| AC009404.2 | SPATA13 | 0.39 | 4.37E-20 |
| AC009404.2 | TEX12 | 0.39 | 3.81E-20 |
| AC009404.2 | TAF2 | 0.39 | 3.71E-20 |
| AC009404.2 | MAPK15 | 0.391 | 3.16E-20 |
| AC009404.2 | TPP2 | 0.391 | 3.48E-20 |
| AC009404.2 | OR10AD1 | 0.391 | 3.48E-20 |
| AC009404.2 | ZNF799 | 0.391 | 3.42E-20 |
| AC009404.2 | HUWE1 | 0.391 | 3.14E-20 |
| AC009404.2 | AC009060.3 | 0.391 | 3.26E-20 |
| AC009404.2 | LYG1 | 0.391 | 3.32E-20 |
| AC009404.2 | ARID2 | 0.391 | 3.03E-20 |
| AC009404.2 | ADAM20 | 0.391 | 3.18E-20 |
| AC009404.2 | GEMIN5 | 0.391 | 3.32E-20 |
| AC009404.2 | FAM209A | 0.392 | 2.75E-20 |
| AC009404.2 | SPRY3 | 0.392 | 2.38E-20 |
| AC009404.2 | GPC2 | 0.392 | 2.70E-20 |
| AC009404.2 | RAD51AP2 | 0.392 | 2.34E-20 |
| AC009404.2 | RP4-614O4.11 | 0.392 | 2.30E-20 |
| AC009404.2 | BRIP1 | 0.392 | 2.64E-20 |
| AC009404.2 | ACACA | 0.392 | 2.24E-20 |
| AC009404.2 | XPOT | 0.392 | 2.64E-20 |
| AC009404.2 | PIF1 | 0.392 | 2.80E-20 |
| AC009404.2 | RFX7 | 0.392 | 2.52E-20 |
| AC009404.2 | BBIP1 | 0.392 | 2.68E-20 |
| AC009404.2 | ADAM17 | 0.393 | 1.84E-20 |
| AC009404.2 | C2CD2 | 0.393 | 1.83E-20 |
| AC009404.2 | DDX10 | 0.393 | 1.99E-20 |
| AC009404.2 | RICTOR | 0.393 | 1.93E-20 |
| AC009404.2 | POLR3A | 0.393 | 2.05E-20 |
| AC009404.2 | SPAG17 | 0.393 | 1.93E-20 |
| AC009404.2 | ZNF317 | 0.394 | 1.64E-20 |
| AC009404.2 | ZNF197 | 0.394 | 1.64E-20 |
| AC009404.2 | KCNC4 | 0.394 | 1.44E-20 |
| AC009404.2 | PCNT | 0.394 | 1.64E-20 |
| AC009404.2 | MKI67 | 0.394 | 1.69E-20 |
| AC009404.2 | MTHFD2L | 0.394 | 1.43E-20 |
| AC009404.2 | NUP43 | 0.394 | 1.65E-20 |
| AC009404.2 | PUM3 | 0.394 | 1.43E-20 |
| AC009404.2 | TRMT11 | 0.394 | 1.43E-20 |
| AC009404.2 | SIN3B | 0.394 | 1.53E-20 |
| AC009404.2 | DCLRE1C | 0.394 | 1.51E-20 |
| AC009404.2 | CIT | 0.394 | 1.53E-20 |
| AC009404.2 | GXYLT1 | 0.394 | 1.65E-20 |
| AC009404.2 | TNRC6C | 0.395 | 1.16E-20 |
| AC009404.2 | HOMER1 | 0.395 | 1.26E-20 |
| AC009404.2 | CDK12 | 0.395 | 1.25E-20 |
| AC009404.2 | REV3L | 0.395 | 1.30E-20 |
| AC009404.2 | TVP23C | 0.395 | 1.11E-20 |
| AC009404.2 | CTD-2369P2.10 | 0.395 | 1.29E-20 |
| AC009404.2 | MYO1H | 0.395 | 1.14E-20 |
| AC009404.2 | VN1R1 | 0.395 | 1.32E-20 |
| AC009404.2 | ENC1 | 0.396 | 9.49E-21 |
| AC009404.2 | ANGPTL8 | 0.396 | 1.07E-20 |
| AC009404.2 | KDM4C | 0.396 | 9.26E-21 |
| AC009404.2 | NSRP1 | 0.396 | 9.17E-21 |
| AC009404.2 | SPINK9 | 0.396 | 1.00E-20 |
| AC009404.2 | RPS6KL1 | 0.396 | 9.54E-21 |
| AC009404.2 | ZNF200 | 0.396 | 1.01E-20 |
| AC009404.2 | PHF10 | 0.396 | 9.94E-21 |
| AC009404.2 | IKBKAP | 0.396 | 9.85E-21 |
| AC009404.2 | SETD2 | 0.396 | 1.01E-20 |
| AC009404.2 | SUMO4 | 0.396 | 1.02E-20 |
| AC009404.2 | RFX3 | 0.396 | 1.06E-20 |
| AC009404.2 | MYCBP2 | 0.397 | 7.77E-21 |
| AC009404.2 | RRP15 | 0.397 | 7.32E-21 |
| AC009404.2 | NCOA3 | 0.397 | 8.01E-21 |
| AC009404.2 | POGK | 0.397 | 8.04E-21 |
| AC009404.2 | TTPAL | 0.397 | 8.09E-21 |
| AC009404.2 | PROX1 | 0.397 | 8.18E-21 |
| AC009404.2 | HAUS7 | 0.397 | 7.57E-21 |
| AC009404.2 | TET1 | 0.397 | 6.66E-21 |
| AC009404.2 | MTG1 | 0.397 | 6.76E-21 |
| AC009404.2 | MTERF2 | 0.397 | 7.74E-21 |
| AC009404.2 | CELSR3 | 0.397 | 8.04E-21 |
| AC009404.2 | MASP2 | 0.397 | 7.91E-21 |
| AC009404.2 | ZNF605 | 0.397 | 6.88E-21 |
| AC009404.2 | UBAP2 | 0.397 | 7.78E-21 |
| AC009404.2 | TTC13 | 0.397 | 7.09E-21 |
| AC009404.2 | SLC10A5 | 0.398 | 5.26E-21 |
| AC009404.2 | HNRNPDL | 0.398 | 5.80E-21 |
| AC009404.2 | GPATCH8 | 0.398 | 5.99E-21 |
| AC009404.2 | MTHFSD | 0.398 | 5.25E-21 |
| AC009404.2 | RCOR3 | 0.398 | 6.22E-21 |
| AC009404.2 | SLC6A6 | 0.398 | 5.46E-21 |
| AC009404.2 | CEP128 | 0.398 | 5.27E-21 |
| AC009404.2 | MSL2 | 0.398 | 6.54E-21 |
| AC009404.2 | LRCH3 | 0.398 | 6.32E-21 |
| AC009404.2 | GSAP | 0.398 | 5.39E-21 |
| AC009404.2 | ZNF10 | 0.398 | 5.83E-21 |
| AC009404.2 | POLR2J3 | 0.399 | 4.43E-21 |
| AC009404.2 | ZSCAN20 | 0.399 | 4.50E-21 |
| AC009404.2 | ZBTB39 | 0.399 | 4.20E-21 |
| AC009404.2 | ANKDD1A | 0.399 | 4.55E-21 |
| AC009404.2 | LRRC58 | 0.399 | 4.28E-21 |
| AC009404.2 | DIEXF | 0.399 | 4.65E-21 |
| AC009404.2 | FBF1 | 0.399 | 4.10E-21 |
| AC009404.2 | RP11-437B10.1 | 0.399 | 4.88E-21 |
| AC009404.2 | KCNH8 | 0.399 | 4.58E-21 |
| AC009404.2 | JMJD7 | 0.399 | 4.70E-21 |
| AC009404.2 | CAMKMT | 0.4 | 3.49E-21 |
| AC009404.2 | POU5F1 | 0.4 | 3.86E-21 |
| AC009404.2 | FAM217A | 0.4 | 3.21E-21 |
| AC009404.2 | MPP6 | 0.4 | 3.85E-21 |
| AC009404.2 | CC2D2B | 0.4 | 3.63E-21 |
| AC009404.2 | TAB3 | 0.4 | 3.84E-21 |
| AC009404.2 | TDRD6 | 0.4 | 3.59E-21 |
| AC009404.2 | TUBB1 | 0.4 | 3.54E-21 |
| AC009404.2 | ZNF738 | 0.4 | 4.03E-21 |
| AC009404.2 | USP40 | 0.4 | 3.25E-21 |
| AC009404.2 | BRD9 | 0.4 | 3.59E-21 |
| AC009404.2 | ZNF30 | 0.4 | 3.86E-21 |
| AC009404.2 | CELA2B | 0.4 | 3.75E-21 |
| AC009404.2 | ST20 | 0.4 | 3.74E-21 |
| AC009404.2 | PKD2L2 | 0.401 | 2.84E-21 |
| AC009404.2 | C15orf62 | 0.401 | 2.53E-21 |
| AC009404.2 | ZNF275 | 0.401 | 2.53E-21 |
| AC009404.2 | TAF1C | 0.401 | 2.70E-21 |
| AC009404.2 | ZBED6 | 0.401 | 3.02E-21 |
| AC009404.2 | DDX31 | 0.401 | 2.53E-21 |
| AC009404.2 | RPS6KB1 | 0.401 | 3.09E-21 |
| AC009404.2 | OSGEPL1 | 0.402 | 2.07E-21 |
| AC009404.2 | ZNF518A | 0.402 | 2.44E-21 |
| AC009404.2 | ZNF100 | 0.402 | 2.30E-21 |
| AC009404.2 | ZNF736 | 0.402 | 2.15E-21 |
| AC009404.2 | ZNF611 | 0.402 | 1.96E-21 |
| AC009404.2 | KIF3A | 0.402 | 2.09E-21 |
| AC009404.2 | DNAJC27 | 0.402 | 1.94E-21 |
| AC009404.2 | WDR5B | 0.402 | 2.00E-21 |
| AC009404.2 | TMEM178A | 0.402 | 2.17E-21 |
| AC009404.2 | TEX14 | 0.402 | 2.39E-21 |
| AC009404.2 | HEATR9 | 0.402 | 2.05E-21 |
| AC009404.2 | CHKB-CPT1B | 0.402 | 2.24E-21 |
| AC009404.2 | KLHL41 | 0.403 | 1.71E-21 |
| AC009404.2 | YOD1 | 0.403 | 1.64E-21 |
| AC009404.2 | COLQ | 0.403 | 1.59E-21 |
| AC009404.2 | DDX11 | 0.403 | 1.79E-21 |
| AC009404.2 | OR2D2 | 0.403 | 1.66E-21 |
| AC009404.2 | RAD52 | 0.403 | 1.74E-21 |
| AC009404.2 | ORC2 | 0.403 | 1.88E-21 |
| AC009404.2 | POLR1B | 0.403 | 1.67E-21 |
| AC009404.2 | TAS2R19 | 0.404 | 1.37E-21 |
| AC009404.2 | RSF1 | 0.404 | 1.44E-21 |
| AC009404.2 | C16orf52 | 0.404 | 1.19E-21 |
| AC009404.2 | AC010642.1 | 0.404 | 1.29E-21 |
| AC009404.2 | DBF4B | 0.404 | 1.48E-21 |
| AC009404.2 | WHSC1 | 0.404 | 1.41E-21 |
| AC009404.2 | VASH2 | 0.404 | 1.37E-21 |
| AC009404.2 | SNRNP48 | 0.404 | 1.26E-21 |
| AC009404.2 | SFPQ | 0.404 | 1.25E-21 |
| AC009404.2 | FAM111A | 0.404 | 1.44E-21 |
| AC009404.2 | RC3H2 | 0.405 | 1.08E-21 |
| AC009404.2 | PCMTD2 | 0.405 | 1.16E-21 |
| AC009404.2 | ZNF273 | 0.405 | 1.14E-21 |
| AC009404.2 | ZSCAN29 | 0.405 | 9.76E-22 |
| AC009404.2 | CENPI | 0.405 | 1.16E-21 |
| AC009404.2 | CEP162 | 0.405 | 9.19E-22 |
| AC009404.2 | MTAP | 0.405 | 1.01E-21 |
| AC009404.2 | KIAA1549 | 0.406 | 8.31E-22 |
| AC009404.2 | RUFY2 | 0.406 | 8.26E-22 |
| AC009404.2 | FAM186A | 0.406 | 7.76E-22 |
| AC009404.2 | C11orf42 | 0.406 | 7.86E-22 |
| AC009404.2 | PHF20 | 0.406 | 7.85E-22 |
| AC009404.2 | EPGN | 0.406 | 7.15E-22 |
| AC009404.2 | DIS3L2 | 0.406 | 8.84E-22 |
| AC009404.2 | FANCD2OS | 0.406 | 7.86E-22 |
| AC009404.2 | CTD-2313N18.7 | 0.406 | 7.93E-22 |
| AC009404.2 | ZXDC | 0.407 | 5.83E-22 |
| AC009404.2 | STXBP4 | 0.407 | 6.23E-22 |
| AC009404.2 | KIAA0895L | 0.407 | 6.15E-22 |
| AC009404.2 | PLCG1 | 0.407 | 6.06E-22 |
| AC009404.2 | YY2 | 0.407 | 5.71E-22 |
| AC009404.2 | SZT2 | 0.407 | 6.83E-22 |
| AC009404.2 | TAS2R31 | 0.407 | 6.37E-22 |
| AC009404.2 | IFT81 | 0.407 | 6.80E-22 |
| AC009404.2 | LAT | 0.407 | 7.09E-22 |
| AC009404.2 | SHPRH | 0.407 | 7.12E-22 |
| AC009404.2 | CCDC7 | 0.407 | 6.45E-22 |
| AC009404.2 | MECP2 | 0.407 | 6.19E-22 |
| AC009404.2 | LMLN | 0.407 | 6.59E-22 |
| AC009404.2 | CBX5 | 0.407 | 7.09E-22 |
| AC009404.2 | C12orf76 | 0.407 | 6.13E-22 |
| AC009404.2 | ASIP | 0.408 | 4.57E-22 |
| AC009404.2 | KLF7 | 0.408 | 4.84E-22 |
| AC009404.2 | DNAH12 | 0.408 | 4.73E-22 |
| AC009404.2 | ZNF510 | 0.409 | 3.42E-22 |
| AC009404.2 | ZNF207 | 0.409 | 3.68E-22 |
| AC009404.2 | ZNF12 | 0.409 | 4.04E-22 |
| AC009404.2 | GOLGA6L10 | 0.409 | 4.05E-22 |
| AC009404.2 | ASB16 | 0.409 | 3.76E-22 |
| AC009404.2 | HDAC8 | 0.409 | 3.63E-22 |
| AC009404.2 | ATP2A1 | 0.409 | 3.51E-22 |
| AC009404.2 | ERCC6 | 0.409 | 3.40E-22 |
| AC009404.2 | EML6 | 0.41 | 3.25E-22 |
| AC009404.2 | HPS4 | 0.41 | 2.87E-22 |
| AC009404.2 | SMIM17 | 0.41 | 3.32E-22 |
| AC009404.2 | CLK2 | 0.41 | 2.89E-22 |
| AC009404.2 | TEX10 | 0.41 | 2.95E-22 |
| AC009404.2 | C3orf49 | 0.41 | 2.99E-22 |
| AC009404.2 | MZF1 | 0.41 | 2.71E-22 |
| AC009404.2 | SCML1 | 0.41 | 2.80E-22 |
| AC009404.2 | COL27A1 | 0.41 | 2.81E-22 |
| AC009404.2 | UTP20 | 0.411 | 2.08E-22 |
| AC009404.2 | SFI1 | 0.411 | 2.03E-22 |
| AC009404.2 | GS1-114I9.3 | 0.411 | 2.06E-22 |
| AC009404.2 | HMBOX1 | 0.411 | 2.56E-22 |
| AC009404.2 | SPATA6L | 0.411 | 2.07E-22 |
| AC009404.2 | ZNF577 | 0.411 | 2.06E-22 |
| AC009404.2 | ANKLE2 | 0.411 | 2.08E-22 |
| AC009404.2 | EYS | 0.411 | 2.47E-22 |
| AC009404.2 | NBPF26 | 0.411 | 2.22E-22 |
| AC009404.2 | C9orf153 | 0.411 | 2.37E-22 |
| AC009404.2 | AKAP11 | 0.411 | 2.44E-22 |
| AC009404.2 | MFSD14C | 0.411 | 2.18E-22 |
| AC009404.2 | PPWD1 | 0.411 | 2.51E-22 |
| AC009404.2 | ASB1 | 0.412 | 1.56E-22 |
| AC009404.2 | ZBTB49 | 0.412 | 1.75E-22 |
| AC009404.2 | PSME4 | 0.412 | 1.65E-22 |
| AC009404.2 | POP1 | 0.412 | 1.68E-22 |
| AC009404.2 | EZH1 | 0.412 | 1.79E-22 |
| AC009404.2 | ZNF131 | 0.412 | 1.75E-22 |
| AC009404.2 | C11orf65 | 0.412 | 1.80E-22 |
| AC009404.2 | MTCP1 | 0.413 | 1.34E-22 |
| AC009404.2 | WFDC13 | 0.413 | 1.25E-22 |
| AC009404.2 | NHLRC2 | 0.413 | 1.21E-22 |
| AC009404.2 | UGGT2 | 0.413 | 1.44E-22 |
| AC009404.2 | NBPF15 | 0.413 | 1.27E-22 |
| AC009404.2 | TBC1D31 | 0.413 | 1.28E-22 |
| AC009404.2 | TMEM120B | 0.413 | 1.31E-22 |
| AC009404.2 | KRIT1 | 0.413 | 1.44E-22 |
| AC009404.2 | TMEM67 | 0.413 | 1.53E-22 |
| AC009404.2 | QSOX2 | 0.413 | 1.50E-22 |
| AC009404.2 | AKAP8L | 0.414 | 9.48E-23 |
| AC009404.2 | ZNF462 | 0.414 | 1.06E-22 |
| AC009404.2 | ZNF236 | 0.414 | 9.87E-23 |
| AC009404.2 | RP11-849H4.2 | 0.414 | 1.07E-22 |
| AC009404.2 | EP400 | 0.414 | 1.10E-22 |
| AC009404.2 | ZNF33B | 0.414 | 1.02E-22 |
| AC009404.2 | FP325331.1 | 0.414 | 1.17E-22 |
| AC009404.2 | SGIP1 | 0.414 | 1.12E-22 |
| AC009404.2 | ZNF83 | 0.414 | 1.12E-22 |
| AC009404.2 | IGFL4 | 0.415 | 9.12E-23 |
| AC009404.2 | IMPG2 | 0.415 | 9.05E-23 |
| AC009404.2 | GP6 | 0.415 | 7.85E-23 |
| AC009404.2 | MORC2 | 0.415 | 8.67E-23 |
| AC009404.2 | KLHDC4 | 0.415 | 8.77E-23 |
| AC009404.2 | FRA10AC1 | 0.415 | 8.98E-23 |
| AC009404.2 | ECT2L | 0.415 | 7.57E-23 |
| AC009404.2 | SERPINE3 | 0.415 | 8.16E-23 |
| AC009404.2 | ZNF670-ZNF695 | 0.415 | 7.72E-23 |
| AC009404.2 | NAPB | 0.415 | 7.68E-23 |
| AC009404.2 | TMPRSS9 | 0.415 | 7.43E-23 |
| AC009404.2 | MBTD1 | 0.415 | 8.14E-23 |
| AC009404.2 | PALM2 | 0.415 | 7.61E-23 |
| AC009404.2 | TTLL4 | 0.415 | 8.89E-23 |
| AC009404.2 | AC008074.1 | 0.416 | 6.80E-23 |
| AC009404.2 | KMT2C | 0.416 | 5.76E-23 |
| AC009404.2 | ZBTB26 | 0.416 | 7.00E-23 |
| AC009404.2 | HKR1 | 0.416 | 5.74E-23 |
| AC009404.2 | GPR89A | 0.416 | 5.66E-23 |
| AC009404.2 | LRP5L | 0.416 | 6.28E-23 |
| AC009404.2 | USP24 | 0.416 | 5.77E-23 |
| AC009404.2 | CSNK2A2 | 0.416 | 6.10E-23 |
| AC009404.2 | CASP8AP2 | 0.416 | 6.03E-23 |
| AC009404.2 | AC109829.1 | 0.416 | 6.24E-23 |
| AC009404.2 | CBL | 0.416 | 7.00E-23 |
| AC009404.2 | LYG2 | 0.416 | 5.58E-23 |
| AC009404.2 | HELZ | 0.416 | 6.82E-23 |
| AC009404.2 | IQGAP3 | 0.417 | 4.85E-23 |
| AC009404.2 | MLANA | 0.417 | 4.52E-23 |
| AC009404.2 | RSRC2 | 0.417 | 4.41E-23 |
| AC009404.2 | STK4 | 0.417 | 4.79E-23 |
| AC009404.2 | ZNF397 | 0.417 | 4.34E-23 |
| AC009404.2 | CCP110 | 0.417 | 5.10E-23 |
| AC009404.2 | IRF9 | 0.417 | 5.01E-23 |
| AC009404.2 | MUM1 | 0.417 | 4.90E-23 |
| AC009404.2 | BRWD1 | 0.418 | 3.47E-23 |
| AC009404.2 | ZNF573 | 0.418 | 4.12E-23 |
| AC009404.2 | ABHD1 | 0.418 | 3.78E-23 |
| AC009404.2 | POU5F1B | 0.418 | 3.44E-23 |
| AC009404.2 | RPA4 | 0.418 | 3.59E-23 |
| AC009404.2 | RGS17 | 0.418 | 3.58E-23 |
| AC009404.2 | WDFY2 | 0.418 | 3.87E-23 |
| AC009404.2 | STKLD1 | 0.418 | 3.96E-23 |
| AC009404.2 | CEP250 | 0.418 | 3.32E-23 |
| AC009404.2 | KCTD19 | 0.418 | 4.12E-23 |
| AC009404.2 | NME9 | 0.419 | 3.10E-23 |
| AC009404.2 | ZMYM2 | 0.419 | 2.98E-23 |
| AC009404.2 | ZNF717 | 0.419 | 2.80E-23 |
| AC009404.2 | C5orf34 | 0.419 | 3.08E-23 |
| AC009404.2 | TTF2 | 0.419 | 2.63E-23 |
| AC009404.2 | OTUD6B | 0.419 | 2.62E-23 |
| AC009404.2 | CDK13 | 0.419 | 2.71E-23 |
| AC009404.2 | SLC26A8 | 0.419 | 2.62E-23 |
| AC009404.2 | CCAR1 | 0.419 | 2.55E-23 |
| AC009404.2 | BRICD5 | 0.419 | 2.86E-23 |
| AC009404.2 | JRKL | 0.419 | 2.94E-23 |
| AC009404.2 | ZNF761 | 0.419 | 2.80E-23 |
| AC009404.2 | XPO5 | 0.419 | 3.23E-23 |
| AC009404.2 | HAUS6 | 0.42 | 2.35E-23 |
| AC009404.2 | TOP3B | 0.42 | 2.18E-23 |
| AC009404.2 | ZNF805 | 0.42 | 2.09E-23 |
| AC009404.2 | VPS8 | 0.42 | 2.40E-23 |
| AC009404.2 | RRH | 0.42 | 2.00E-23 |
| AC009404.2 | MTX3 | 0.42 | 2.25E-23 |
| AC009404.2 | RP11-65B7.2 | 0.42 | 2.44E-23 |
| AC009404.2 | ZNF678 | 0.42 | 2.00E-23 |
| AC009404.2 | CCDC73 | 0.42 | 2.17E-23 |
| AC009404.2 | CTD-2116N17.1 | 0.42 | 2.19E-23 |
| AC009404.2 | ZNF652 | 0.42 | 2.08E-23 |
| AC009404.2 | TICRR | 0.42 | 2.04E-23 |
| AC009404.2 | PGS1 | 0.42 | 2.20E-23 |
| AC009404.2 | USP45 | 0.42 | 2.45E-23 |
| AC009404.2 | TRIO | 0.42 | 2.30E-23 |
| AC009404.2 | AL138706.2 | 0.421 | 1.55E-23 |
| AC009404.2 | AC105009.1 | 0.421 | 1.68E-23 |
| AC009404.2 | ASB3 | 0.421 | 1.71E-23 |
| AC009404.2 | IKBKB | 0.421 | 1.87E-23 |
| AC009404.2 | ZNF506 | 0.421 | 1.88E-23 |
| AC009404.2 | NDUFAF7 | 0.421 | 1.92E-23 |
| AC009404.2 | ASPDH | 0.421 | 1.80E-23 |
| AC009404.2 | TMEM262 | 0.421 | 1.55E-23 |
| AC009404.2 | ZNF714 | 0.421 | 1.50E-23 |
| AC009404.2 | R3HDML | 0.421 | 1.61E-23 |
| AC009404.2 | TOPBP1 | 0.422 | 1.35E-23 |
| AC009404.2 | ZNF600 | 0.422 | 1.25E-23 |
| AC009404.2 | NEK5 | 0.422 | 1.35E-23 |
| AC009404.2 | SH3BP2 | 0.422 | 1.25E-23 |
| AC009404.2 | INTS6 | 0.422 | 1.19E-23 |
| AC009404.2 | USP37 | 0.422 | 1.17E-23 |
| AC009404.2 | HOOK3 | 0.422 | 1.36E-23 |
| AC009404.2 | RP11-192H23.4 | 0.422 | 1.40E-23 |
| AC009404.2 | ACCS | 0.422 | 1.43E-23 |
| AC009404.2 | BTBD19 | 0.422 | 1.38E-23 |
| AC009404.2 | ZBTB25 | 0.422 | 1.25E-23 |
| AC009404.2 | TEN1-CDK3 | 0.422 | 1.32E-23 |
| AC009404.2 | SYNGAP1 | 0.423 | 1.06E-23 |
| AC009404.2 | SRSF6 | 0.423 | 9.46E-24 |
| AC009404.2 | LRRC37A | 0.423 | 1.03E-23 |
| AC009404.2 | RAB40AL | 0.423 | 9.94E-24 |
| AC009404.2 | ZNF451 | 0.423 | 1.10E-23 |
| AC009404.2 | RFPL3S | 0.423 | 9.26E-24 |
| AC009404.2 | DNAJC24 | 0.423 | 1.03E-23 |
| AC009404.2 | RBM34 | 0.423 | 1.06E-23 |
| AC009404.2 | MGA | 0.423 | 9.08E-24 |
| AC009404.2 | ILF3 | 0.424 | 7.52E-24 |
| AC009404.2 | CENPE | 0.424 | 8.31E-24 |
| AC009404.2 | ZNF720 | 0.424 | 7.44E-24 |
| AC009404.2 | BAZ2B | 0.424 | 6.91E-24 |
| AC009404.2 | RANBP17 | 0.424 | 8.49E-24 |
| AC009404.2 | ATRX | 0.424 | 8.36E-24 |
| AC009404.2 | ZNF785 | 0.424 | 6.70E-24 |
| AC009404.2 | ZNF234 | 0.424 | 7.09E-24 |
| AC009404.2 | AC091180.1 | 0.424 | 8.57E-24 |
| AC009404.2 | CEP135 | 0.424 | 8.07E-24 |
| AC009404.2 | BRAF | 0.424 | 7.94E-24 |
| AC009404.2 | GLRA1 | 0.425 | 5.12E-24 |
| AC009404.2 | KCNIP2 | 0.425 | 6.49E-24 |
| AC009404.2 | TXLNG | 0.425 | 5.22E-24 |
| AC009404.2 | DNAJB7 | 0.425 | 6.06E-24 |
| AC009404.2 | QRICH2 | 0.425 | 6.55E-24 |
| AC009404.2 | VPS13A | 0.425 | 5.81E-24 |
| AC009404.2 | FAM188B | 0.425 | 5.62E-24 |
| AC009404.2 | RBM19 | 0.426 | 4.64E-24 |
| AC009404.2 | ELMOD3 | 0.426 | 4.01E-24 |
| AC009404.2 | ASAP1 | 0.426 | 4.04E-24 |
| AC009404.2 | ZNF791 | 0.426 | 4.28E-24 |
| AC009404.2 | PAXIP1 | 0.426 | 4.82E-24 |
| AC009404.2 | FAM161A | 0.426 | 4.88E-24 |
| AC009404.2 | FAM122B | 0.426 | 4.87E-24 |
| AC009404.2 | CRYBA1 | 0.426 | 4.04E-24 |
| AC009404.2 | ANAPC1 | 0.426 | 4.24E-24 |
| AC009404.2 | RP5-1021I20.4 | 0.427 | 3.14E-24 |
| AC009404.2 | UPF3B | 0.427 | 3.06E-24 |
| AC009404.2 | WDR43 | 0.427 | 3.57E-24 |
| AC009404.2 | NR6A1 | 0.427 | 3.31E-24 |
| AC009404.2 | FAM76B | 0.427 | 3.65E-24 |
| AC009404.2 | CCDC81 | 0.427 | 3.44E-24 |
| AC009404.2 | SRRM2 | 0.427 | 3.85E-24 |
| AC009404.2 | ZNF91 | 0.427 | 3.48E-24 |
| AC009404.2 | NKRF | 0.428 | 2.36E-24 |
| AC009404.2 | LRRC16B | 0.428 | 2.68E-24 |
| AC009404.2 | GOLGA8A | 0.428 | 2.41E-24 |
| AC009404.2 | RAD51D | 0.428 | 2.54E-24 |
| AC009404.2 | ZNF766 | 0.428 | 2.84E-24 |
| AC009404.2 | TAS2R3 | 0.428 | 2.32E-24 |
| AC009404.2 | PP2D1 | 0.428 | 2.75E-24 |
| AC009404.2 | PRH1 | 0.428 | 2.70E-24 |
| AC009404.2 | BRWD3 | 0.428 | 2.82E-24 |
| AC009404.2 | ACSBG2 | 0.428 | 2.58E-24 |
| AC009404.2 | MAPK8IP3 | 0.428 | 2.58E-24 |
| AC009404.2 | PMFBP1 | 0.428 | 2.48E-24 |
| AC009404.2 | RAB11FIP2 | 0.429 | 2.21E-24 |
| AC009404.2 | CDK5RAP3 | 0.429 | 1.77E-24 |
| AC009404.2 | THOC1 | 0.429 | 2.06E-24 |
| AC009404.2 | ZNF473 | 0.429 | 1.93E-24 |
| AC009404.2 | ANKRD31 | 0.429 | 1.98E-24 |
| AC009404.2 | NF1 | 0.429 | 2.06E-24 |
| AC009404.2 | ZNF326 | 0.429 | 1.78E-24 |
| AC009404.2 | ZNF846 | 0.43 | 1.40E-24 |
| AC009404.2 | ZNF443 | 0.43 | 1.48E-24 |
| AC009404.2 | RNF169 | 0.43 | 1.42E-24 |
| AC009404.2 | SF3B1 | 0.43 | 1.72E-24 |
| AC009404.2 | RP11-411B6.6 | 0.43 | 1.50E-24 |
| AC009404.2 | ATF7IP | 0.43 | 1.63E-24 |
| AC009404.2 | TRIM17 | 0.43 | 1.35E-24 |
| AC009404.2 | AGTPBP1 | 0.43 | 1.34E-24 |
| AC009404.2 | TTN | 0.43 | 1.59E-24 |
| AC009404.2 | PLAC8L1 | 0.43 | 1.58E-24 |
| AC009404.2 | TTC21A | 0.431 | 1.16E-24 |
| AC009404.2 | ZNF250 | 0.431 | 1.06E-24 |
| AC009404.2 | SPRN | 0.431 | 1.29E-24 |
| AC009404.2 | RAB40A | 0.431 | 1.05E-24 |
| AC009404.2 | MSI2 | 0.431 | 1.27E-24 |
| AC009404.2 | DDI2 | 0.431 | 1.07E-24 |
| AC009404.2 | CTC-479C5.12 | 0.431 | 1.16E-24 |
| AC009404.2 | ING5 | 0.431 | 1.32E-24 |
| AC009404.2 | MCM8 | 0.431 | 1.19E-24 |
| AC009404.2 | AC138969.4 | 0.431 | 1.30E-24 |
| AC009404.2 | BRCA1 | 0.432 | 9.83E-25 |
| AC009404.2 | CNTF | 0.432 | 7.93E-25 |
| AC009404.2 | OSBPL3 | 0.432 | 9.63E-25 |
| AC009404.2 | BIRC6 | 0.432 | 8.87E-25 |
| AC009404.2 | SLC9A5 | 0.432 | 9.33E-25 |
| AC009404.2 | C2CD3 | 0.432 | 9.33E-25 |
| AC009404.2 | ICE1 | 0.433 | 6.18E-25 |
| AC009404.2 | MAP3K4 | 0.433 | 7.12E-25 |
| AC009404.2 | ZNF292 | 0.433 | 6.77E-25 |
| AC009404.2 | ZNF430 | 0.433 | 6.51E-25 |
| AC009404.2 | AC004076.7 | 0.433 | 6.37E-25 |
| AC009404.2 | CDK3 | 0.434 | 4.76E-25 |
| AC009404.2 | QTRTD1 | 0.434 | 5.59E-25 |
| AC009404.2 | EXOG | 0.434 | 5.74E-25 |
| AC009404.2 | ANKRD26 | 0.434 | 5.59E-25 |
| AC009404.2 | ZRSR1 | 0.434 | 5.50E-25 |
| AC009404.2 | CUZD1 | 0.434 | 4.83E-25 |
| AC009404.2 | SLF2 | 0.434 | 5.43E-25 |
| AC009404.2 | DCAF17 | 0.434 | 4.57E-25 |
| AC009404.2 | OFD1 | 0.434 | 5.06E-25 |
| AC009404.2 | MSANTD1 | 0.434 | 5.00E-25 |
| AC009404.2 | COX19 | 0.434 | 4.79E-25 |
| AC009404.2 | C1orf101 | 0.435 | 3.86E-25 |
| AC009404.2 | DOPEY1 | 0.435 | 4.14E-25 |
| AC009404.2 | RP11-286N22.8 | 0.435 | 3.53E-25 |
| AC009404.2 | AOC2 | 0.435 | 3.85E-25 |
| AC009404.2 | RASA2 | 0.435 | 3.45E-25 |
| AC009404.2 | ZNF432 | 0.435 | 3.87E-25 |
| AC009404.2 | ZNF783 | 0.435 | 3.97E-25 |
| AC009404.2 | DLEU1 | 0.436 | 3.19E-25 |
| AC009404.2 | PRR3 | 0.436 | 2.85E-25 |
| AC009404.2 | ZNF778 | 0.436 | 3.33E-25 |
| AC009404.2 | IFT80 | 0.436 | 2.56E-25 |
| AC009404.2 | TTC14 | 0.436 | 3.10E-25 |
| AC009404.2 | SARNP | 0.436 | 2.86E-25 |
| AC009404.2 | QSER1 | 0.436 | 2.85E-25 |
| AC009404.2 | PTPN4 | 0.436 | 3.11E-25 |
| AC009404.2 | ZRANB3 | 0.436 | 2.84E-25 |
| AC009404.2 | HEATR1 | 0.437 | 2.43E-25 |
| AC009404.2 | AC018867.1 | 0.437 | 2.07E-25 |
| AC009404.2 | ZNF343 | 0.437 | 2.36E-25 |
| AC009404.2 | XPNPEP3 | 0.437 | 2.24E-25 |
| AC009404.2 | BPTF | 0.438 | 1.85E-25 |
| AC009404.2 | CLCN5 | 0.438 | 1.51E-25 |
| AC009404.2 | VMP1 | 0.438 | 1.57E-25 |
| AC009404.2 | KIAA1958 | 0.438 | 1.77E-25 |
| AC009404.2 | ZNF44 | 0.438 | 1.62E-25 |
| AC009404.2 | LRP6 | 0.438 | 1.59E-25 |
| AC009404.2 | ZNF708 | 0.439 | 1.18E-25 |
| AC009404.2 | RANBP6 | 0.439 | 1.17E-25 |
| AC009404.2 | GIGYF1 | 0.439 | 1.30E-25 |
| AC009404.2 | CCDC79 | 0.439 | 1.25E-25 |
| AC009404.2 | CNTRL | 0.439 | 1.44E-25 |
| AC009404.2 | BOD1L1 | 0.439 | 1.27E-25 |
| AC009404.2 | EFHC1 | 0.44 | 9.43E-26 |
| AC009404.2 | SYCP3 | 0.44 | 9.74E-26 |
| AC009404.2 | 6-Mar | 0.44 | 1.06E-25 |
| AC009404.2 | PVRIG | 0.44 | 1.06E-25 |
| AC009404.2 | ZNF81 | 0.44 | 9.19E-26 |
| AC009404.2 | TBC1D24 | 0.44 | 9.92E-26 |
| AC009404.2 | CFAP69 | 0.441 | 7.15E-26 |
| AC009404.2 | PRPF4B | 0.441 | 6.79E-26 |
| AC009404.2 | CCDC146 | 0.441 | 6.73E-26 |
| AC009404.2 | UBE3D | 0.441 | 6.63E-26 |
| AC009404.2 | RBL1 | 0.441 | 8.11E-26 |
| AC009404.2 | CDRT4 | 0.441 | 6.45E-26 |
| AC009404.2 | DNAH14 | 0.441 | 6.87E-26 |
| AC009404.2 | SECISBP2 | 0.441 | 7.04E-26 |
| AC009404.2 | ASPM | 0.442 | 4.85E-26 |
| AC009404.2 | CCDC180 | 0.442 | 5.24E-26 |
| AC009404.2 | GPCPD1 | 0.442 | 5.98E-26 |
| AC009404.2 | KIAA2026 | 0.442 | 5.48E-26 |
| AC009404.2 | AMMECR1L | 0.442 | 5.74E-26 |
| AC009404.2 | PHIP | 0.442 | 4.99E-26 |
| AC009404.2 | METTL8 | 0.442 | 4.95E-26 |
| AC009404.2 | WDR60 | 0.442 | 4.91E-26 |
| AC009404.2 | WDPCP | 0.442 | 5.34E-26 |
| AC009404.2 | HAUS3 | 0.442 | 5.65E-26 |
| AC009404.2 | ZNF75D | 0.442 | 5.17E-26 |
| AC009404.2 | FBXW2 | 0.443 | 4.46E-26 |
| AC009404.2 | C1orf100 | 0.443 | 3.89E-26 |
| AC009404.2 | SOCS7 | 0.443 | 3.60E-26 |
| AC009404.2 | TSGA10 | 0.443 | 4.43E-26 |
| AC009404.2 | ZNF485 | 0.443 | 4.42E-26 |
| AC009404.2 | ZNF546 | 0.443 | 3.95E-26 |
| AC009404.2 | R3HDM1 | 0.443 | 4.46E-26 |
| AC009404.2 | ADNP | 0.443 | 4.49E-26 |
| AC009404.2 | ALG13 | 0.443 | 4.15E-26 |
| AC009404.2 | USP36 | 0.444 | 2.93E-26 |
| AC009404.2 | WDR35 | 0.444 | 3.51E-26 |
| AC009404.2 | NEMP1 | 0.444 | 3.32E-26 |
| AC009404.2 | NBPF19 | 0.444 | 3.44E-26 |
| AC009404.2 | ALMS1 | 0.445 | 2.62E-26 |
| AC009404.2 | NOP58 | 0.445 | 2.00E-26 |
| AC009404.2 | DDHD1 | 0.445 | 2.27E-26 |
| AC009404.2 | C1orf111 | 0.446 | 1.68E-26 |
| AC009404.2 | CCDC7 | 0.446 | 1.50E-26 |
| AC009404.2 | CFAP70 | 0.446 | 1.83E-26 |
| AC009404.2 | EME2 | 0.446 | 1.75E-26 |
| AC009404.2 | PCBD2 | 0.446 | 1.75E-26 |
| AC009404.2 | PHC3 | 0.446 | 1.81E-26 |
| AC009404.2 | RPGRIP1L | 0.446 | 1.58E-26 |
| AC009404.2 | SAMD12 | 0.446 | 1.64E-26 |
| AC009404.2 | SLC39A10 | 0.447 | 1.27E-26 |
| AC009404.2 | RAD54B | 0.447 | 1.29E-26 |
| AC009404.2 | TRPV1 | 0.447 | 1.35E-26 |
| AC009404.2 | CCDC65 | 0.447 | 1.19E-26 |
| AC009404.2 | RP5-1042K10.14 | 0.447 | 1.30E-26 |
| AC009404.2 | SBNO1 | 0.447 | 1.19E-26 |
| AC009404.2 | CHRNG | 0.447 | 1.16E-26 |
| AC009404.2 | FBXL13 | 0.447 | 1.17E-26 |
| AC009404.2 | ABI2 | 0.448 | 9.94E-27 |
| AC009404.2 | TEDDM1 | 0.448 | 9.60E-27 |
| AC009404.2 | PRSS37 | 0.448 | 1.12E-26 |
| AC009404.2 | SRSF10 | 0.448 | 9.48E-27 |
| AC009404.2 | ZBTB24 | 0.448 | 1.01E-26 |
| AC009404.2 | DDX17 | 0.448 | 9.61E-27 |
| AC009404.2 | TP53TG5 | 0.448 | 9.81E-27 |
| AC009404.2 | RP4-583P15.15 | 0.448 | 8.61E-27 |
| AC009404.2 | CHORDC1 | 0.449 | 6.64E-27 |
| AC009404.2 | AGAP9 | 0.449 | 7.68E-27 |
| AC009404.2 | GPATCH2 | 0.449 | 6.32E-27 |
| AC009404.2 | ALS2 | 0.449 | 6.98E-27 |
| AC009404.2 | ARHGEF38 | 0.449 | 6.81E-27 |
| AC009404.2 | RBM25 | 0.449 | 7.02E-27 |
| AC009404.2 | POGZ | 0.45 | 5.30E-27 |
| AC009404.2 | LUC7L2 | 0.45 | 5.31E-27 |
| AC009404.2 | USPL1 | 0.45 | 5.17E-27 |
| AC009404.2 | ZNF565 | 0.45 | 5.66E-27 |
| AC009404.2 | SLC26A5 | 0.45 | 5.46E-27 |
| AC009404.2 | AHCTF1 | 0.45 | 4.93E-27 |
| AC009404.2 | MACC1 | 0.45 | 5.47E-27 |
| AC009404.2 | ANAPC4 | 0.451 | 4.28E-27 |
| AC009404.2 | RLIM | 0.451 | 4.68E-27 |
| AC009404.2 | ZNF107 | 0.451 | 4.53E-27 |
| AC009404.2 | SLC4A5 | 0.451 | 4.44E-27 |
| AC009404.2 | XPO4 | 0.452 | 2.63E-27 |
| AC009404.2 | AGER | 0.452 | 3.15E-27 |
| AC009404.2 | POLN | 0.452 | 3.12E-27 |
| AC009404.2 | DNAJC2 | 0.452 | 3.20E-27 |
| AC009404.2 | VPS13B | 0.452 | 3.37E-27 |
| AC009404.2 | ZNF566 | 0.452 | 3.41E-27 |
| AC009404.2 | PRPF40B | 0.452 | 2.71E-27 |
| AC009404.2 | S100PBP | 0.452 | 2.91E-27 |
| AC009404.2 | ZNF780A | 0.452 | 3.23E-27 |
| AC009404.2 | R3HDM2 | 0.452 | 2.87E-27 |
| AC009404.2 | DLEU7 | 0.452 | 3.25E-27 |
| AC009404.2 | ZNF133 | 0.452 | 2.68E-27 |
| AC009404.2 | CYP19A1 | 0.453 | 2.53E-27 |
| AC009404.2 | PRPF38B | 0.453 | 2.38E-27 |
| AC009404.2 | ZC3H12B | 0.453 | 2.31E-27 |
| AC009404.2 | PROZ | 0.453 | 2.29E-27 |
| AC009404.2 | PRKDC | 0.453 | 2.06E-27 |
| AC009404.2 | ZNF638 | 0.453 | 1.98E-27 |
| AC009404.2 | STRADA | 0.453 | 2.01E-27 |
| AC009404.2 | OXTR | 0.453 | 2.50E-27 |
| AC009404.2 | TTC34 | 0.454 | 1.68E-27 |
| AC009404.2 | FANCB | 0.454 | 1.82E-27 |
| AC009404.2 | CAPN3 | 0.454 | 1.76E-27 |
| AC009404.2 | DGKE | 0.454 | 1.68E-27 |
| AC009404.2 | ZNF493 | 0.454 | 1.66E-27 |
| AC009404.2 | LHX4 | 0.454 | 1.93E-27 |
| AC009404.2 | KCNV2 | 0.454 | 1.56E-27 |
| AC009404.2 | ATP6AP1L | 0.455 | 1.17E-27 |
| AC009404.2 | KIF14 | 0.455 | 1.33E-27 |
| AC009404.2 | PTCD2 | 0.455 | 1.44E-27 |
| AC009404.2 | OTULIN | 0.455 | 1.15E-27 |
| AC009404.2 | PHF20L1 | 0.455 | 1.39E-27 |
| AC009404.2 | BCO2 | 0.455 | 1.34E-27 |
| AC009404.2 | ZNF721 | 0.455 | 1.31E-27 |
| AC009404.2 | PRPF39 | 0.455 | 1.12E-27 |
| AC009404.2 | DCAF8 | 0.455 | 1.15E-27 |
| AC009404.2 | ASXL1 | 0.456 | 1.02E-27 |
| AC009404.2 | ORMDL1 | 0.456 | 9.85E-28 |
| AC009404.2 | CCDC175 | 0.456 | 8.50E-28 |
| AC009404.2 | ZFP62 | 0.456 | 9.92E-28 |
| AC009404.2 | CCNJ | 0.456 | 9.78E-28 |
| AC009404.2 | CENPP | 0.457 | 6.71E-28 |
| AC009404.2 | RBBP6 | 0.457 | 6.24E-28 |
| AC009404.2 | EFCAB10 | 0.457 | 7.29E-28 |
| AC009404.2 | TIMM23B | 0.457 | 6.20E-28 |
| AC009404.2 | TULP4 | 0.458 | 5.54E-28 |
| AC009404.2 | UPF3A | 0.458 | 4.62E-28 |
| AC009404.2 | ZNF674 | 0.458 | 4.98E-28 |
| AC009404.2 | GON4L | 0.458 | 4.71E-28 |
| AC009404.2 | U2SURP | 0.458 | 4.76E-28 |
| AC009404.2 | PUS7L | 0.458 | 4.86E-28 |
| AC009404.2 | U2AF1 | 0.459 | 3.75E-28 |
| AC009404.2 | PRRC2C | 0.459 | 3.36E-28 |
| AC009404.2 | ZCCHC11 | 0.459 | 4.00E-28 |
| AC009404.2 | SPDYE1 | 0.459 | 3.59E-28 |
| AC009404.2 | TPR | 0.459 | 3.71E-28 |
| AC009404.2 | RUFY3 | 0.459 | 4.37E-28 |
| AC009404.2 | HSF4 | 0.459 | 4.03E-28 |
| AC009404.2 | RP11-793H13.10 | 0.459 | 4.05E-28 |
| AC009404.2 | TAS2R10 | 0.46 | 2.70E-28 |
| AC009404.2 | RP11-80H18.3 | 0.46 | 2.93E-28 |
| AC009404.2 | NOM1 | 0.46 | 2.44E-28 |
| AC009404.2 | ZNF33A | 0.46 | 2.45E-28 |
| AC009404.2 | WSB1 | 0.461 | 1.85E-28 |
| AC009404.2 | LRGUK | 0.461 | 2.06E-28 |
| AC009404.2 | LTB4R | 0.461 | 1.83E-28 |
| AC009404.2 | NR2C2 | 0.462 | 1.72E-28 |
| AC009404.2 | MLLT10 | 0.462 | 1.42E-28 |
| AC009404.2 | MGEA5 | 0.462 | 1.58E-28 |
| AC009404.2 | TTC3 | 0.462 | 1.44E-28 |
| AC009404.2 | KMT2A | 0.462 | 1.41E-28 |
| AC009404.2 | CARF | 0.463 | 1.07E-28 |
| AC009404.2 | HELB | 0.463 | 1.01E-28 |
| AC009404.2 | ZNF117 | 0.463 | 1.26E-28 |
| AC009404.2 | MEMO1 | 0.463 | 1.05E-28 |
| AC009404.2 | HECTD4 | 0.463 | 1.07E-28 |
| AC009404.2 | NEU3 | 0.463 | 1.10E-28 |
| AC009404.2 | TIGD1 | 0.464 | 8.52E-29 |
| AC009404.2 | ADAMTS6 | 0.464 | 8.91E-29 |
| AC009404.2 | ZNF623 | 0.464 | 9.34E-29 |
| AC009404.2 | MTR | 0.464 | 8.40E-29 |
| AC009404.2 | KANSL1 | 0.464 | 8.00E-29 |
| AC009404.2 | ELK4 | 0.464 | 9.41E-29 |
| AC009404.2 | BICD1 | 0.464 | 8.67E-29 |
| AC009404.2 | RABGGTB | 0.464 | 7.18E-29 |
| AC009404.2 | HEATR4 | 0.464 | 8.22E-29 |
| AC009404.2 | UHRF1BP1 | 0.464 | 9.04E-29 |
| AC009404.2 | METTL22 | 0.464 | 8.90E-29 |
| AC009404.2 | WDR75 | 0.465 | 5.44E-29 |
| AC009404.2 | RP11-691N7.6 | 0.465 | 5.97E-29 |
| AC009404.2 | AP006285.2 | 0.465 | 6.45E-29 |
| AC009404.2 | KANSL1L | 0.465 | 7.09E-29 |
| AC009404.2 | CAPN10 | 0.466 | 5.18E-29 |
| AC009404.2 | WDR19 | 0.466 | 4.73E-29 |
| AC009404.2 | TUBA3D | 0.466 | 4.94E-29 |
| AC009404.2 | METTL3 | 0.466 | 4.20E-29 |
| AC009404.2 | ZNF852 | 0.466 | 5.13E-29 |
| AC009404.2 | UBR5 | 0.467 | 3.62E-29 |
| AC009404.2 | PHF14 | 0.467 | 3.03E-29 |
| AC009404.2 | ZNF587B | 0.467 | 3.68E-29 |
| AC009404.2 | CLASP1 | 0.467 | 3.41E-29 |
| AC009404.2 | ABCC5 | 0.468 | 2.14E-29 |
| AC009404.2 | SERHL2 | 0.468 | 2.51E-29 |
| AC009404.2 | WDR33 | 0.468 | 2.55E-29 |
| AC009404.2 | TIA1 | 0.468 | 2.80E-29 |
| AC009404.2 | UBXN7 | 0.468 | 2.47E-29 |
| AC009404.2 | KANSL3 | 0.469 | 1.73E-29 |
| AC009404.2 | NOXRED1 | 0.469 | 1.60E-29 |
| AC009404.2 | ZNF283 | 0.469 | 2.06E-29 |
| AC009404.2 | FBXW7 | 0.469 | 1.67E-29 |
| AC009404.2 | SLC25A36 | 0.47 | 1.43E-29 |
| AC009404.2 | MMS22L | 0.471 | 9.61E-30 |
| AC009404.2 | TUBE1 | 0.472 | 6.05E-30 |
| AC009404.2 | PMS1 | 0.472 | 7.42E-30 |
| AC009404.2 | MPP3 | 0.472 | 7.66E-30 |
| AC009404.2 | RPGR | 0.472 | 8.17E-30 |
| AC009404.2 | PAN3 | 0.472 | 7.41E-30 |
| AC009404.2 | SPDYE2 | 0.473 | 4.86E-30 |
| AC009404.2 | ZSCAN9 | 0.473 | 4.98E-30 |
| AC009404.2 | LRP8 | 0.473 | 4.59E-30 |
| AC009404.2 | CDKL3 | 0.473 | 4.44E-30 |
| AC009404.2 | HELLS | 0.473 | 5.07E-30 |
| AC009404.2 | BDP1 | 0.473 | 5.93E-30 |
| AC009404.2 | CCDC57 | 0.474 | 3.68E-30 |
| AC009404.2 | N4BP2L2 | 0.474 | 3.34E-30 |
| AC009404.2 | SETD4 | 0.474 | 3.95E-30 |
| AC009404.2 | UBE2G2 | 0.474 | 4.12E-30 |
| AC009404.2 | PAPD7 | 0.475 | 2.54E-30 |
| AC009404.2 | ATAD2B | 0.475 | 2.46E-30 |
| AC009404.2 | INVS | 0.475 | 2.64E-30 |
| AC009404.2 | NUTM2G | 0.475 | 2.98E-30 |
| AC009404.2 | MAK | 0.475 | 2.90E-30 |
| AC009404.2 | NABP1 | 0.475 | 2.63E-30 |
| AC009404.2 | UBE2V1 | 0.476 | 1.79E-30 |
| AC009404.2 | FBXW12 | 0.476 | 1.80E-30 |
| AC009404.2 | CENPF | 0.476 | 1.74E-30 |
| AC009404.2 | MSH5-SAPCD1 | 0.476 | 2.00E-30 |
| AC009404.2 | FAM186B | 0.476 | 2.00E-30 |
| AC009404.2 | THOC2 | 0.476 | 2.18E-30 |
| AC009404.2 | TAC4 | 0.476 | 2.26E-30 |
| AC009404.2 | SUPT20H | 0.477 | 1.36E-30 |
| AC009404.2 | SLC9C1 | 0.477 | 1.32E-30 |
| AC009404.2 | LRRC37A2 | 0.477 | 1.54E-30 |
| AC009404.2 | ZNF519 | 0.478 | 1.11E-30 |
| AC009404.2 | ZNF276 | 0.478 | 9.83E-31 |
| AC009404.2 | SRSF11 | 0.478 | 1.21E-30 |
| AC009404.2 | ZNF251 | 0.478 | 1.01E-30 |
| AC009404.2 | TSSK3 | 0.478 | 9.78E-31 |
| AC009404.2 | RP11-894J14.5 | 0.479 | 8.70E-31 |
| AC009404.2 | PIKFYVE | 0.48 | 5.94E-31 |
| AC009404.2 | CCDC30 | 0.48 | 5.23E-31 |
| AC009404.2 | AGBL3 | 0.48 | 6.30E-31 |
| AC009404.2 | CRAMP1 | 0.48 | 4.84E-31 |
| AC009404.2 | SLC7A6 | 0.48 | 5.71E-31 |
| AC009404.2 | MLH3 | 0.48 | 5.00E-31 |
| AC009404.2 | GNRH1 | 0.48 | 6.09E-31 |
| AC009404.2 | ZNF445 | 0.48 | 5.68E-31 |
| AC009404.2 | RIF1 | 0.481 | 3.70E-31 |
| AC009404.2 | FMNL2 | 0.481 | 3.50E-31 |
| AC009404.2 | ZDHHC17 | 0.481 | 3.49E-31 |
| AC009404.2 | SETD6 | 0.481 | 4.34E-31 |
| AC009404.2 | CPSF6 | 0.481 | 3.59E-31 |
| AC009404.2 | INTS3 | 0.481 | 4.44E-31 |
| AC009404.2 | GABRR2 | 0.482 | 2.51E-31 |
| AC009404.2 | ZNF160 | 0.482 | 3.31E-31 |
| AC009404.2 | GARNL3 | 0.482 | 3.19E-31 |
| AC009404.2 | CBWD2 | 0.482 | 2.73E-31 |
| AC009404.2 | ACRV1 | 0.482 | 2.63E-31 |
| AC009404.2 | KLC1 | 0.482 | 3.25E-31 |
| AC009404.2 | DCUN1D2 | 0.482 | 2.83E-31 |
| AC009404.2 | ATG16L2 | 0.483 | 1.95E-31 |
| AC009404.2 | AC087350.1 | 0.483 | 2.09E-31 |
| AC009404.2 | DCDC2B | 0.483 | 1.81E-31 |
| AC009404.2 | IRGQ | 0.483 | 2.30E-31 |
| AC009404.2 | FKBP14 | 0.484 | 1.53E-31 |
| AC009404.2 | CCDC93 | 0.484 | 1.47E-31 |
| AC009404.2 | ZNF449 | 0.484 | 1.62E-31 |
| AC009404.2 | KIAA1024 | 0.484 | 1.55E-31 |
| AC009404.2 | TET3 | 0.484 | 1.36E-31 |
| AC009404.2 | ARL17A | 0.484 | 1.31E-31 |
| AC009404.2 | NCR3LG1 | 0.484 | 1.74E-31 |
| AC009404.2 | GAN | 0.484 | 1.72E-31 |
| AC009404.2 | GRIN2B | 0.485 | 9.69E-32 |
| AC009404.2 | TSSK4 | 0.485 | 1.26E-31 |
| AC009404.2 | NAA25 | 0.485 | 9.94E-32 |
| AC009404.2 | E2F3 | 0.485 | 1.16E-31 |
| AC009404.2 | CHD6 | 0.486 | 7.77E-32 |
| AC009404.2 | CCNT2 | 0.486 | 6.78E-32 |
| AC009404.2 | SLC22A1 | 0.486 | 9.37E-32 |
| AC009404.2 | FAM208B | 0.487 | 5.15E-32 |
| AC009404.2 | ZKSCAN8 | 0.487 | 5.73E-32 |
| AC009404.2 | MEIOC | 0.487 | 5.81E-32 |
| AC009404.2 | ZNF558 | 0.488 | 3.80E-32 |
| AC009404.2 | RBM44 | 0.488 | 4.48E-32 |
| AC009404.2 | ZNF780B | 0.488 | 3.54E-32 |
| AC009404.2 | ASB14 | 0.488 | 3.72E-32 |
| AC009404.2 | ANKAR | 0.488 | 3.84E-32 |
| AC009404.2 | RAD54L2 | 0.488 | 4.25E-32 |
| AC009404.2 | ZNF562 | 0.489 | 3.30E-32 |
| AC009404.2 | NOL8 | 0.489 | 3.41E-32 |
| AC009404.2 | GPR135 | 0.489 | 2.93E-32 |
| AC009404.2 | BRCA2 | 0.489 | 3.04E-32 |
| AC009404.2 | L3MBTL1 | 0.489 | 2.96E-32 |
| AC009404.2 | SALL4 | 0.489 | 2.93E-32 |
| AC009404.2 | SCAI | 0.49 | 2.47E-32 |
| AC009404.2 | WDR92 | 0.491 | 1.45E-32 |
| AC009404.2 | MRE11A | 0.491 | 1.42E-32 |
| AC009404.2 | GOLGA8B | 0.491 | 1.73E-32 |
| AC009404.2 | CEP83 | 0.491 | 1.57E-32 |
| AC009404.2 | ANKS3 | 0.492 | 9.69E-33 |
| AC009404.2 | ANKRD36C | 0.492 | 1.15E-32 |
| AC009404.2 | KIF27 | 0.492 | 1.24E-32 |
| AC009404.2 | ARGLU1 | 0.492 | 1.01E-32 |
| AC009404.2 | ZNF878 | 0.492 | 1.00E-32 |
| AC009404.2 | TAF1 | 0.492 | 1.29E-32 |
| AC009404.2 | ALS2CR12 | 0.493 | 7.39E-33 |
| AC009404.2 | FGFR1OP | 0.493 | 8.63E-33 |
| AC009404.2 | RABL2A | 0.493 | 8.92E-33 |
| AC009404.2 | B3GAT2 | 0.494 | 4.97E-33 |
| AC009404.2 | ZBTB37 | 0.494 | 5.63E-33 |
| AC009404.2 | ATXN2 | 0.494 | 5.89E-33 |
| AC009404.2 | RSRP1 | 0.494 | 6.50E-33 |
| AC009404.2 | PRPF3 | 0.495 | 4.09E-33 |
| AC009404.2 | KCNJ14 | 0.495 | 3.89E-33 |
| AC009404.2 | HSPBAP1 | 0.495 | 4.49E-33 |
| AC009404.2 | CEP164 | 0.495 | 3.60E-33 |
| AC009404.2 | ZC3H11A | 0.496 | 2.86E-33 |
| AC009404.2 | LSM8 | 0.496 | 2.78E-33 |
| AC009404.2 | RTEL1 | 0.496 | 2.73E-33 |
| AC009404.2 | CCDC18 | 0.496 | 3.02E-33 |
| AC009404.2 | PCF11 | 0.496 | 2.61E-33 |
| AC009404.2 | LENG8 | 0.497 | 2.40E-33 |
| AC009404.2 | RBM15 | 0.497 | 1.95E-33 |
| AC009404.2 | DPY19L3 | 0.497 | 2.23E-33 |
| AC009404.2 | NUFIP2 | 0.497 | 1.94E-33 |
| AC009404.2 | ATM | 0.498 | 1.62E-33 |
| AC009404.2 | ACAD11 | 0.498 | 1.69E-33 |
| AC009404.2 | EFCAB13 | 0.498 | 1.22E-33 |
| AC009404.2 | RBM26 | 0.499 | 9.52E-34 |
| AC009404.2 | RBAK | 0.499 | 1.14E-33 |
| AC009404.2 | HNRNPA1L2 | 0.499 | 9.32E-34 |
| AC009404.2 | ZNF248 | 0.499 | 9.06E-34 |
| AC009404.2 | AF011889.5 | 0.499 | 9.47E-34 |
| AC009404.2 | ZNF280C | 0.5 | 7.84E-34 |
| AC009404.2 | PAN2 | 0.501 | 5.23E-34 |
| AC009404.2 | ZNF692 | 0.501 | 5.91E-34 |
| AC009404.2 | NBPF20 | 0.501 | 4.53E-34 |
| AC009404.2 | JRK | 0.501 | 5.26E-34 |
| AC009404.2 | ANKRD36B | 0.502 | 3.36E-34 |
| AC009404.2 | TBX19 | 0.502 | 3.74E-34 |
| AC009404.2 | ZNF7 | 0.502 | 3.74E-34 |
| AC009404.2 | ZNF559-ZNF177 | 0.502 | 3.48E-34 |
| AC009404.2 | ARR3 | 0.503 | 2.46E-34 |
| AC009404.2 | GCNT7 | 0.503 | 2.52E-34 |
| AC009404.2 | ZBTB43 | 0.503 | 2.47E-34 |
| AC009404.2 | RP11-111M22.2 | 0.504 | 1.69E-34 |
| AC009404.2 | NFXL1 | 0.504 | 1.88E-34 |
| AC009404.2 | FAM193B | 0.504 | 1.56E-34 |
| AC009404.2 | PDE7A | 0.504 | 2.05E-34 |
| AC009404.2 | VEGFA | 0.505 | 1.48E-34 |
| AC009404.2 | LA16c-431H6.6 | 0.505 | 1.49E-34 |
| AC009404.2 | GABPB2 | 0.505 | 1.50E-34 |
| AC009404.2 | CHD2 | 0.505 | 1.11E-34 |
| AC009404.2 | USP31 | 0.505 | 1.53E-34 |
| AC009404.2 | CBWD5 | 0.505 | 1.16E-34 |
| AC009404.2 | ZNF333 | 0.506 | 8.84E-35 |
| AC009404.2 | LRRC39 | 0.507 | 5.52E-35 |
| AC009404.2 | RBM41 | 0.507 | 7.47E-35 |
| AC009404.2 | TAMM41 | 0.508 | 4.43E-35 |
| AC009404.2 | THADA | 0.508 | 5.06E-35 |
| AC009404.2 | STRC | 0.508 | 3.83E-35 |
| AC009404.2 | HYPK | 0.508 | 4.69E-35 |
| AC009404.2 | CLK4 | 0.508 | 4.70E-35 |
| AC009404.2 | NSUN6 | 0.509 | 2.95E-35 |
| AC009404.2 | RP11-574K11.31 | 0.509 | 3.23E-35 |
| AC009404.2 | C20orf144 | 0.509 | 2.93E-35 |
| AC009404.2 | CHKB | 0.509 | 2.92E-35 |
| AC009404.2 | MTBP | 0.509 | 2.71E-35 |
| AC009404.2 | MYSM1 | 0.509 | 3.74E-35 |
| AC009404.2 | ZNF202 | 0.51 | 2.29E-35 |
| AC009404.2 | ANKRD18A | 0.51 | 1.89E-35 |
| AC009404.2 | INTU | 0.51 | 2.40E-35 |
| AC009404.2 | ZNF169 | 0.51 | 2.18E-35 |
| AC009404.2 | METAP1D | 0.51 | 2.03E-35 |
| AC009404.2 | ZNF182 | 0.51 | 2.01E-35 |
| AC009404.2 | SMG1 | 0.51 | 2.33E-35 |
| AC009404.2 | LRRC37B | 0.511 | 1.48E-35 |
| AC009404.2 | SPICE1 | 0.512 | 1.03E-35 |
| AC009404.2 | ZNF224 | 0.512 | 1.11E-35 |
| AC009404.2 | ZNF195 | 0.512 | 1.18E-35 |
| AC009404.2 | TAS2R14 | 0.513 | 6.51E-36 |
| AC009404.2 | DCAF16 | 0.513 | 7.62E-36 |
| AC009404.2 | XRCC2 | 0.514 | 4.51E-36 |
| AC009404.2 | NPIPA1 | 0.514 | 6.14E-36 |
| AC009404.2 | STX16 | 0.514 | 6.28E-36 |
| AC009404.2 | CCDC39 | 0.515 | 3.68E-36 |
| AC009404.2 | SUPT7L | 0.515 | 4.16E-36 |
| AC009404.2 | RBM5 | 0.515 | 3.59E-36 |
| AC009404.2 | ZNF507 | 0.515 | 4.28E-36 |
| AC009404.2 | LRP2BP | 0.515 | 3.47E-36 |
| AC009404.2 | FAM118A | 0.516 | 2.49E-36 |
| AC009404.2 | PLEKHA8 | 0.516 | 2.58E-36 |
| AC009404.2 | UVSSA | 0.517 | 1.52E-36 |
| AC009404.2 | PAXBP1 | 0.517 | 1.81E-36 |
| AC009404.2 | DGKH | 0.518 | 1.21E-36 |
| AC009404.2 | NFAT5 | 0.518 | 1.05E-36 |
| AC009404.2 | AGO3 | 0.519 | 7.92E-37 |
| AC009404.2 | EDRF1 | 0.52 | 6.68E-37 |
| AC009404.2 | SREK1 | 0.52 | 5.38E-37 |
| AC009404.2 | ZNF84 | 0.52 | 5.29E-37 |
| AC009404.2 | OCLM | 0.52 | 7.15E-37 |
| AC009404.2 | SENP5 | 0.521 | 4.33E-37 |
| AC009404.2 | ZNF589 | 0.521 | 4.08E-37 |
| AC009404.2 | ABL2 | 0.522 | 2.70E-37 |
| AC009404.2 | INO80D | 0.522 | 2.69E-37 |
| AC009404.2 | THUMPD2 | 0.522 | 2.86E-37 |
| AC009404.2 | ZNF700 | 0.522 | 3.30E-37 |
| AC009404.2 | MATR3 | 0.522 | 3.37E-37 |
| AC009404.2 | KLHL31 | 0.522 | 3.24E-37 |
| AC009404.2 | GPR89B | 0.523 | 2.03E-37 |
| AC009404.2 | CCDC191 | 0.523 | 2.27E-37 |
| AC009404.2 | ZNF891 | 0.524 | 1.15E-37 |
| AC009404.2 | GEN1 | 0.524 | 1.29E-37 |
| AC009404.2 | CELF1 | 0.524 | 1.63E-37 |
| AC009404.2 | ZNF814 | 0.524 | 1.20E-37 |
| AC009404.2 | GPATCH2L | 0.524 | 1.41E-37 |
| AC009404.2 | NAA16 | 0.525 | 9.43E-38 |
| AC009404.2 | C9orf50 | 0.525 | 8.91E-38 |
| AC009404.2 | ZNF782 | 0.525 | 1.02E-37 |
| AC009404.2 | TSC1 | 0.525 | 1.06E-37 |
| AC009404.2 | EIF4A1 | 0.526 | 6.32E-38 |
| AC009404.2 | NBPF12 | 0.526 | 6.96E-38 |
| AC009404.2 | ZC3H8 | 0.527 | 4.78E-38 |
| AC009404.2 | TTC17 | 0.527 | 4.32E-38 |
| AC009404.2 | RP11-11N7.5 | 0.527 | 3.78E-38 |
| AC009404.2 | AMY2B | 0.527 | 4.80E-38 |
| AC009404.2 | CEP290 | 0.528 | 3.60E-38 |
| AC009404.2 | ANKRD10 | 0.528 | 2.82E-38 |
| AC009404.2 | ZNF841 | 0.528 | 2.89E-38 |
| AC009404.2 | SAPCD1 | 0.528 | 3.62E-38 |
| AC009404.2 | PRR4 | 0.529 | 2.40E-38 |
| AC009404.2 | ATAD5 | 0.529 | 2.03E-38 |
| AC009404.2 | TYW5 | 0.529 | 2.05E-38 |
| AC009404.2 | C6orf163 | 0.529 | 1.88E-38 |
| AC009404.2 | YEATS2 | 0.529 | 1.85E-38 |
| AC009404.2 | ARHGAP8 | 0.53 | 1.58E-38 |
| AC009404.2 | CEP295 | 0.53 | 1.42E-38 |
| AC009404.2 | CLDN20 | 0.531 | 1.16E-38 |
| AC009404.2 | UHRF2 | 0.532 | 7.31E-39 |
| AC009404.2 | ATR | 0.532 | 7.45E-39 |
| AC009404.2 | IMPG1 | 0.532 | 7.50E-39 |
| AC009404.2 | STX16-NPEPL1 | 0.532 | 5.92E-39 |
| AC009404.2 | BTAF1 | 0.532 | 5.58E-39 |
| AC009404.2 | TNRC6A | 0.533 | 3.90E-39 |
| AC009404.2 | CATSPER2 | 0.533 | 5.30E-39 |
| AC009404.2 | ARL13A | 0.534 | 3.58E-39 |
| AC009404.2 | PRDM15 | 0.534 | 3.64E-39 |
| AC009404.2 | SEC61A2 | 0.534 | 2.64E-39 |
| AC009404.2 | LRIT3 | 0.534 | 3.07E-39 |
| AC009404.2 | PABPC1L | 0.534 | 2.68E-39 |
| AC009404.2 | DNAH6 | 0.534 | 3.03E-39 |
| AC009404.2 | RBM12B | 0.535 | 1.82E-39 |
| AC009404.2 | SS18L1 | 0.535 | 2.35E-39 |
| AC009404.2 | ZNF37A | 0.535 | 2.25E-39 |
| AC009404.2 | FAM71F2 | 0.535 | 2.46E-39 |
| AC009404.2 | SPTY2D1-AS1 | 0.535 | 1.91E-39 |
| AC009404.2 | LRIG2 | 0.535 | 2.49E-39 |
| AC009404.2 | POLQ | 0.536 | 1.54E-39 |
| AC009404.2 | NBPF9 | 0.536 | 1.63E-39 |
| AC009404.2 | L3HYPDH | 0.536 | 1.35E-39 |
| AC009404.2 | AC090154.1 | 0.536 | 1.75E-39 |
| AC009404.2 | ANKHD1 | 0.536 | 1.62E-39 |
| AC009404.2 | PRR5-ARHGAP8 | 0.537 | 8.75E-40 |
| AC009404.2 | ORAOV1 | 0.537 | 9.23E-40 |
| AC009404.2 | DNAH17 | 0.537 | 9.25E-40 |
| AC009404.2 | TRAF5 | 0.537 | 8.47E-40 |
| AC009404.2 | TRIM66 | 0.538 | 7.84E-40 |
| AC009404.2 | PCGF3 | 0.538 | 7.69E-40 |
| AC009404.2 | DFNB59 | 0.538 | 6.04E-40 |
| AC009404.2 | KCNMB3 | 0.539 | 4.56E-40 |
| AC009404.2 | PNN | 0.539 | 4.18E-40 |
| AC009404.2 | WDR73 | 0.539 | 3.89E-40 |
| AC009404.2 | RBM28 | 0.54 | 3.41E-40 |
| AC009404.2 | PROX2 | 0.54 | 3.20E-40 |
| AC009404.2 | AP000866.1 | 0.54 | 2.77E-40 |
| AC009404.2 | PTPDC1 | 0.541 | 1.70E-40 |
| AC009404.2 | ERICH6B | 0.541 | 2.29E-40 |
| AC009404.2 | CENPJ | 0.542 | 1.35E-40 |
| AC009404.2 | POU2F1 | 0.542 | 1.24E-40 |
| AC009404.2 | ANKMY1 | 0.542 | 1.20E-40 |
| AC009404.2 | PILRB | 0.543 | 1.13E-40 |
| AC009404.2 | OTUD3 | 0.543 | 7.70E-41 |
| AC009404.2 | GABRE | 0.543 | 9.75E-41 |
| AC009404.2 | PCNXL2 | 0.544 | 5.57E-41 |
| AC009404.2 | RNPC3 | 0.544 | 7.04E-41 |
| AC009404.2 | CCDC66 | 0.545 | 4.37E-41 |
| AC009404.2 | LSMEM1 | 0.545 | 3.52E-41 |
| AC009404.2 | CAPRIN2 | 0.545 | 5.02E-41 |
| AC009404.2 | AGAP5 | 0.545 | 3.75E-41 |
| AC009404.2 | CLK1 | 0.546 | 2.39E-41 |
| AC009404.2 | RP11-468E2.4 | 0.546 | 2.63E-41 |
| AC009404.2 | ZNF8 | 0.546 | 2.82E-41 |
| AC009404.2 | ANKZF1 | 0.547 | 1.84E-41 |
| AC009404.2 | TIAF1 | 0.547 | 2.06E-41 |
| AC009404.2 | SPDYA | 0.547 | 1.83E-41 |
| AC009404.2 | SLC13A4 | 0.547 | 1.74E-41 |
| AC009404.2 | ZNF587 | 0.548 | 1.19E-41 |
| AC009404.2 | SEC31B | 0.549 | 7.08E-42 |
| AC009404.2 | TSEN2 | 0.549 | 7.75E-42 |
| AC009404.2 | REV1 | 0.55 | 5.36E-42 |
| AC009404.2 | SRRM5 | 0.55 | 4.79E-42 |
| AC009404.2 | KNTC1 | 0.55 | 5.44E-42 |
| AC009404.2 | NBPF11 | 0.551 | 4.28E-42 |
| AC009404.2 | MATR3 | 0.551 | 3.28E-42 |
| AC009404.2 | STK36 | 0.553 | 1.66E-42 |
| AC009404.2 | AHI1 | 0.553 | 1.72E-42 |
| AC009404.2 | ATAT1 | 0.553 | 1.61E-42 |
| AC009404.2 | NPHP3 | 0.554 | 1.01E-42 |
| AC009404.2 | LRRC69 | 0.554 | 1.09E-42 |
| AC009404.2 | BNIPL | 0.555 | 6.59E-43 |
| AC009404.2 | PASK | 0.555 | 6.73E-43 |
| AC009404.2 | ZGRF1 | 0.555 | 8.27E-43 |
| AC009404.2 | TAS2R20 | 0.556 | 4.22E-43 |
| AC009404.2 | ACRC | 0.556 | 3.97E-43 |
| AC009404.2 | SPDYE5 | 0.556 | 4.37E-43 |
| AC009404.2 | TAF1D | 0.557 | 2.70E-43 |
| AC009404.2 | UBAP1L | 0.558 | 1.90E-43 |
| AC009404.2 | DDX55 | 0.559 | 1.51E-43 |
| AC009404.2 | ACVR2B | 0.559 | 1.16E-43 |
| AC009404.2 | RP3-461F17.3 | 0.559 | 1.61E-43 |
| AC009404.2 | EGFL8 | 0.56 | 8.42E-44 |
| AC009404.2 | CBWD7 | 0.56 | 1.01E-43 |
| AC009404.2 | DCAF4L1 | 0.56 | 9.43E-44 |
| AC009404.2 | SLFNL1 | 0.56 | 9.80E-44 |
| AC009404.2 | UBN2 | 0.56 | 9.38E-44 |
| AC009404.2 | PDE6C | 0.561 | 7.13E-44 |
| AC009404.2 | HNRNPH1 | 0.562 | 4.47E-44 |
| AC009404.2 | ZNF514 | 0.563 | 2.57E-44 |
| AC009404.2 | ANKRD36 | 0.564 | 2.05E-44 |
| AC009404.2 | ANKRD61 | 0.565 | 1.13E-44 |
| AC009404.2 | POLG2 | 0.566 | 6.79E-45 |
| AC009404.2 | BBS1 | 0.566 | 5.80E-45 |
| AC009404.2 | NPIPB4 | 0.566 | 7.15E-45 |
| AC009404.2 | CSPP1 | 0.567 | 4.74E-45 |
| AC009404.2 | RBM14-RBM4 | 0.568 | 2.67E-45 |
| AC009404.2 | ZNF789 | 0.57 | 1.46E-45 |
| AC009404.2 | DNAH1 | 0.571 | 8.97E-46 |
| AC009404.2 | PPT2-EGFL8 | 0.571 | 6.69E-46 |
| AC009404.2 | RP11-444E17.6 | 0.572 | 6.04E-46 |
| AC009404.2 | ZNF337 | 0.573 | 4.10E-46 |
| AC009404.2 | SETD5 | 0.573 | 3.23E-46 |
| AC009404.2 | TARBP1 | 0.573 | 4.05E-46 |
| AC009404.2 | CSAD | 0.573 | 3.70E-46 |
| AC009404.2 | MDN1 | 0.575 | 1.64E-46 |
| AC009404.2 | C8orf44 | 0.575 | 1.61E-46 |
| AC009404.2 | LY6G5B | 0.575 | 1.20E-46 |
| AC009404.2 | LUC7L3 | 0.575 | 1.60E-46 |
| AC009404.2 | PLA2G4B | 0.577 | 5.73E-47 |
| AC009404.2 | TCERG1 | 0.578 | 4.08E-47 |
| AC009404.2 | DDX47 | 0.579 | 2.08E-47 |
| AC009404.2 | GPR75 | 0.579 | 1.96E-47 |
| AC009404.2 | C5orf45 | 0.579 | 2.17E-47 |
| AC009404.2 | CBWD3 | 0.579 | 2.24E-47 |
| AC009404.2 | TTLL3 | 0.579 | 2.08E-47 |
| AC009404.2 | MDM4 | 0.581 | 1.00E-47 |
| AC009404.2 | SGK494 | 0.581 | 9.69E-48 |
| AC009404.2 | ARHGAP11B | 0.581 | 9.88E-48 |
| AC009404.2 | EBLN2 | 0.582 | 5.23E-48 |
| AC009404.2 | SFSWAP | 0.583 | 3.29E-48 |
| AC009404.2 | CEP152 | 0.585 | 1.36E-48 |
| AC009404.2 | CEP95 | 0.585 | 1.80E-48 |
| AC009404.2 | TRMT10B | 0.586 | 8.64E-49 |
| AC009404.2 | RBM39 | 0.587 | 7.04E-49 |
| AC009404.2 | JMJD7-PLA2G4B | 0.587 | 6.94E-49 |
| AC009404.2 | TAS2R4 | 0.588 | 4.17E-49 |
| AC009404.2 | ERVK3-1 | 0.589 | 2.60E-49 |
| AC009404.2 | ZNF354B | 0.593 | 3.16E-50 |
| AC009404.2 | MSS51 | 0.593 | 3.40E-50 |
| AC009404.2 | CHD7 | 0.594 | 2.43E-50 |
| AC009404.2 | SCLY | 0.594 | 2.20E-50 |
| AC009404.2 | CREBZF | 0.595 | 1.36E-50 |
| AC009404.2 | ZFC3H1 | 0.596 | 1.12E-50 |
| AC009404.2 | AGO2 | 0.596 | 7.97E-51 |
| AC009404.2 | ZNF23 | 0.596 | 1.03E-50 |
| AC009404.2 | RBM6 | 0.599 | 2.54E-51 |
| AC009404.2 | FAM227A | 0.599 | 2.81E-51 |
| AC009404.2 | MSANTD2 | 0.599 | 2.09E-51 |
| AC009404.2 | ZNF431 | 0.6 | 1.33E-51 |
| AC009404.2 | CRYGS | 0.601 | 7.45E-52 |
| AC009404.2 | CLHC1 | 0.603 | 2.95E-52 |
| AC009404.2 | ZNF621 | 0.607 | 3.66E-53 |
| AC009404.2 | USP49 | 0.608 | 2.61E-53 |
| AC009404.2 | EP400NL | 0.609 | 1.43E-53 |
| AC009404.2 | ANKRD23 | 0.609 | 1.64E-53 |
| AC009404.2 | LTB4R2 | 0.609 | 1.54E-53 |
| AC009404.2 | AARSD1 | 0.61 | 9.77E-54 |
| AC009404.2 | TRIM52 | 0.61 | 1.05E-53 |
| AC009404.2 | PRSS53 | 0.61 | 9.43E-54 |
| AC009404.2 | DNHD1 | 0.612 | 3.49E-54 |
| AC009404.2 | GOLGA6L9 | 0.613 | 2.04E-54 |
| AC009404.2 | PNISR | 0.613 | 1.97E-54 |
| AC009404.2 | CCNL2 | 0.613 | 2.64E-54 |
| AC009404.2 | TCTE3 | 0.614 | 1.74E-54 |
| AC009404.2 | RP11-434D12.1 | 0.616 | 5.24E-55 |
| AC009404.2 | SLC25A27 | 0.616 | 5.87E-55 |
| AC009404.2 | AGAP4 | 0.62 | 7.97E-56 |
| AC009404.2 | CCDC84 | 0.623 | 1.30E-56 |
| AC009404.2 | ADAT2 | 0.624 | 9.70E-57 |
| AC009404.2 | ZNF26 | 0.624 | 1.05E-56 |
| AC009404.2 | DMTF1 | 0.625 | 3.74E-57 |
| AC009404.2 | ALKBH6 | 0.628 | 7.88E-58 |
| AC009404.2 | CCDC14 | 0.629 | 7.20E-58 |
| AC009404.2 | LUC7L | 0.63 | 4.13E-58 |
| AC009404.2 | TAS2R5 | 0.631 | 1.94E-58 |
| AC009404.2 | CCNL1 | 0.631 | 1.74E-58 |
| AC009404.2 | RBM33 | 0.632 | 1.48E-58 |
| AC009404.2 | KIAA0907 | 0.636 | 1.18E-59 |
| AC009404.2 | SUGP2 | 0.642 | 5.67E-61 |
| AC009404.2 | CFAP44 | 0.642 | 4.44E-61 |
| AC009404.2 | NKTR | 0.648 | 1.52E-62 |
| AC009404.2 | RP11-33O4.2 | 0.649 | 1.04E-62 |
| AC009404.2 | DDX39B | 0.653 | 7.56E-64 |
| AC009404.2 | MSH5 | 0.653 | 8.13E-64 |
| AC009404.2 | FNBP4 | 0.655 | 2.00E-64 |
| AC009404.2 | CCDC150 | 0.657 | 8.15E-65 |
| AC009404.2 | AHSA2 | 0.66 | 1.40E-65 |
| AC009404.2 | OGT | 0.669 | 6.56E-68 |
| AC009404.2 | ZNF121 | 0.671 | 1.81E-68 |
| AC009404.2 | WDR27 | 0.712 | 1.43E-80 |
| AC009404.2 | AGAP6 | 0.717 | 4.10E-82 |
| AC073283.7 | ATP5A1 | -0.384 | 1.76E-19 |
| AC073283.7 | MYL6 | -0.356 | 7.74E-17 |
| AC073283.7 | PTRHD1 | -0.352 | 1.99E-16 |
| AC073283.7 | COX5A | -0.35 | 2.84E-16 |
| AC073283.7 | COX7A2 | -0.341 | 1.84E-15 |
| AC073283.7 | CA2 | -0.34 | 2.32E-15 |
| AC073283.7 | UQCRFS1 | -0.333 | 8.12E-15 |
| AC073283.7 | ACAA2 | -0.333 | 9.52E-15 |
| AC073283.7 | C12orf57 | -0.331 | 1.36E-14 |
| AC073283.7 | MRPL54 | -0.329 | 1.77E-14 |
| AC073283.7 | ATP5G3 | -0.329 | 2.09E-14 |
| AC073283.7 | ATP5F1 | -0.327 | 3.12E-14 |
| AC073283.7 | ATP5B | -0.321 | 8.33E-14 |
| AC073283.7 | SLC25A11 | -0.32 | 1.02E-13 |
| AC073283.7 | C14orf142 | -0.32 | 9.85E-14 |
| AC073283.7 | ACADS | -0.319 | 1.34E-13 |
| AC073283.7 | SQRDL | -0.319 | 1.34E-13 |
| AC073283.7 | HINT1 | -0.318 | 1.51E-13 |
| AC073283.7 | FAM162A | -0.318 | 1.40E-13 |
| AC073283.7 | MPDU1 | -0.318 | 1.55E-13 |
| AC073283.7 | NDUFB1 | -0.318 | 1.56E-13 |
| AC073283.7 | ETFA | -0.313 | 4.07E-13 |
| AC073283.7 | MRPL34 | -0.313 | 3.59E-13 |
| AC073283.7 | UQCR10 | -0.313 | 3.88E-13 |
| AC073283.7 | ADH5 | -0.31 | 6.93E-13 |
| AC073283.7 | CGRRF1 | -0.31 | 6.40E-13 |
| AC073283.7 | PRADC1 | -0.31 | 6.44E-13 |
| AC073283.7 | SPINK2 | -0.305 | 1.67E-12 |
| AC073283.7 | NDUFA6 | -0.305 | 1.48E-12 |
| AC073283.7 | NDUFS4 | -0.303 | 2.15E-12 |
| AC073283.7 | TOP3B | 0.301 | 3.02E-12 |
| AC073283.7 | PNN | 0.301 | 2.99E-12 |
| AC073283.7 | TBX19 | 0.301 | 2.95E-12 |
| AC073283.7 | CENPF | 0.301 | 3.17E-12 |
| AC073283.7 | ARMCX4 | 0.301 | 3.44E-12 |
| AC073283.7 | DCAF16 | 0.301 | 3.33E-12 |
| AC073283.7 | ZNF84 | 0.301 | 2.93E-12 |
| AC073283.7 | MSS51 | 0.301 | 3.20E-12 |
| AC073283.7 | NR2C2 | 0.302 | 2.87E-12 |
| AC073283.7 | MORC2 | 0.302 | 2.58E-12 |
| AC073283.7 | ERCC6L | 0.302 | 2.76E-12 |
| AC073283.7 | GPR89B | 0.302 | 2.79E-12 |
| AC073283.7 | ZMAT1 | 0.302 | 2.77E-12 |
| AC073283.7 | INTS6 | 0.302 | 2.54E-12 |
| AC073283.7 | ZNF398 | 0.302 | 2.72E-12 |
| AC073283.7 | CLHC1 | 0.302 | 2.85E-12 |
| AC073283.7 | AKAP11 | 0.302 | 2.49E-12 |
| AC073283.7 | NUP214 | 0.303 | 2.08E-12 |
| AC073283.7 | VEGFA | 0.303 | 2.07E-12 |
| AC073283.7 | CELSR1 | 0.303 | 2.34E-12 |
| AC073283.7 | RC3H2 | 0.303 | 2.36E-12 |
| AC073283.7 | LA16c-431H6.6 | 0.303 | 2.09E-12 |
| AC073283.7 | SPEN | 0.303 | 2.06E-12 |
| AC073283.7 | UNKL | 0.303 | 2.20E-12 |
| AC073283.7 | AC024060.1 | 0.303 | 2.13E-12 |
| AC073283.7 | FAM219A | 0.303 | 2.11E-12 |
| AC073283.7 | LUC7L | 0.303 | 2.16E-12 |
| AC073283.7 | COX19 | 0.303 | 2.39E-12 |
| AC073283.7 | KCNV2 | 0.303 | 2.07E-12 |
| AC073283.7 | FOXK1 | 0.304 | 1.84E-12 |
| AC073283.7 | TUBGCP6 | 0.304 | 1.89E-12 |
| AC073283.7 | CMTM1 | 0.304 | 1.83E-12 |
| AC073283.7 | LSM8 | 0.304 | 1.78E-12 |
| AC073283.7 | ZNF717 | 0.304 | 2.01E-12 |
| AC073283.7 | NAA16 | 0.304 | 1.98E-12 |
| AC073283.7 | SERHL2 | 0.304 | 1.98E-12 |
| AC073283.7 | GABPB2 | 0.304 | 2.02E-12 |
| AC073283.7 | TAF1C | 0.304 | 1.89E-12 |
| AC073283.7 | CDK13 | 0.304 | 1.94E-12 |
| AC073283.7 | ZNF587B | 0.304 | 1.83E-12 |
| AC073283.7 | BRWD3 | 0.304 | 2.05E-12 |
| AC073283.7 | PHC3 | 0.304 | 1.96E-12 |
| AC073283.7 | DLEU7 | 0.304 | 1.84E-12 |
| AC073283.7 | FAM47E | 0.304 | 2.03E-12 |
| AC073283.7 | TIGD1 | 0.305 | 1.51E-12 |
| AC073283.7 | LPIN3 | 0.305 | 1.57E-12 |
| AC073283.7 | NFATC2IP | 0.305 | 1.53E-12 |
| AC073283.7 | FBXW8 | 0.305 | 1.53E-12 |
| AC073283.7 | STK4 | 0.305 | 1.64E-12 |
| AC073283.7 | CSNK2A2 | 0.305 | 1.48E-12 |
| AC073283.7 | MRE11A | 0.305 | 1.66E-12 |
| AC073283.7 | ACSBG2 | 0.305 | 1.58E-12 |
| AC073283.7 | BRICD5 | 0.305 | 1.71E-12 |
| AC073283.7 | PLXNA1 | 0.305 | 1.68E-12 |
| AC073283.7 | QSOX2 | 0.305 | 1.57E-12 |
| AC073283.7 | SALL4 | 0.305 | 1.56E-12 |
| AC073283.7 | ZNF160 | 0.306 | 1.28E-12 |
| AC073283.7 | NRBP2 | 0.306 | 1.37E-12 |
| AC073283.7 | KCNIP2 | 0.306 | 1.35E-12 |
| AC073283.7 | ATP2A1 | 0.306 | 1.32E-12 |
| AC073283.7 | SMURF1 | 0.306 | 1.31E-12 |
| AC073283.7 | SLC7A6 | 0.306 | 1.34E-12 |
| AC073283.7 | TCERG1 | 0.306 | 1.40E-12 |
| AC073283.7 | AC018867.1 | 0.307 | 1.04E-12 |
| AC073283.7 | MDC1 | 0.307 | 1.12E-12 |
| AC073283.7 | TRMT2B | 0.307 | 1.13E-12 |
| AC073283.7 | PRRC2B | 0.307 | 1.04E-12 |
| AC073283.7 | ADNP | 0.307 | 1.18E-12 |
| AC073283.7 | LY6G6E | 0.307 | 1.19E-12 |
| AC073283.7 | CENPI | 0.307 | 1.14E-12 |
| AC073283.7 | ZNF335 | 0.307 | 1.14E-12 |
| AC073283.7 | FHAD1 | 0.308 | 9.73E-13 |
| AC073283.7 | ADAMTS6 | 0.308 | 9.12E-13 |
| AC073283.7 | EFHC1 | 0.308 | 1.01E-12 |
| AC073283.7 | MYCBP2 | 0.308 | 1.01E-12 |
| AC073283.7 | SPRY3 | 0.308 | 1.01E-12 |
| AC073283.7 | C2CD2 | 0.308 | 8.87E-13 |
| AC073283.7 | ACAD11 | 0.308 | 8.97E-13 |
| AC073283.7 | GON4L | 0.308 | 9.93E-13 |
| AC073283.7 | FBXL8 | 0.308 | 1.02E-12 |
| AC073283.7 | RAD18 | 0.308 | 9.73E-13 |
| AC073283.7 | ZNF248 | 0.308 | 8.78E-13 |
| AC073283.7 | CHD6 | 0.309 | 7.86E-13 |
| AC073283.7 | LIME1 | 0.309 | 7.94E-13 |
| AC073283.7 | PRR4 | 0.309 | 8.04E-13 |
| AC073283.7 | ANKAR | 0.309 | 8.13E-13 |
| AC073283.7 | KIF27 | 0.31 | 6.54E-13 |
| AC073283.7 | RBM12B | 0.31 | 6.32E-13 |
| AC073283.7 | MKI67 | 0.31 | 6.01E-13 |
| AC073283.7 | DDX31 | 0.31 | 6.78E-13 |
| AC073283.7 | ZKSCAN1 | 0.31 | 7.00E-13 |
| AC073283.7 | CELF1 | 0.31 | 6.69E-13 |
| AC073283.7 | TNK2 | 0.31 | 6.33E-13 |
| AC073283.7 | CENPP | 0.311 | 5.62E-13 |
| AC073283.7 | ZNF121 | 0.311 | 5.17E-13 |
| AC073283.7 | WBP2NL | 0.311 | 5.07E-13 |
| AC073283.7 | NEK5 | 0.311 | 5.73E-13 |
| AC073283.7 | HNRNPA1L2 | 0.311 | 5.36E-13 |
| AC073283.7 | JRK | 0.311 | 5.27E-13 |
| AC073283.7 | TBC1D24 | 0.311 | 5.53E-13 |
| AC073283.7 | FBXW2 | 0.312 | 4.67E-13 |
| AC073283.7 | UPF3B | 0.312 | 4.90E-13 |
| AC073283.7 | FAM118A | 0.312 | 4.84E-13 |
| AC073283.7 | ZNF276 | 0.312 | 4.77E-13 |
| AC073283.7 | PROCA1 | 0.312 | 4.76E-13 |
| AC073283.7 | SEMA4D | 0.312 | 4.89E-13 |
| AC073283.7 | LRCH1 | 0.312 | 4.29E-13 |
| AC073283.7 | CASP2 | 0.312 | 4.42E-13 |
| AC073283.7 | HDAC8 | 0.312 | 4.55E-13 |
| AC073283.7 | DDI2 | 0.312 | 4.18E-13 |
| AC073283.7 | POLA1 | 0.312 | 4.18E-13 |
| AC073283.7 | CELSR3 | 0.312 | 4.90E-13 |
| AC073283.7 | ZNF318 | 0.312 | 4.62E-13 |
| AC073283.7 | WFDC10B | 0.313 | 3.56E-13 |
| AC073283.7 | SPATA25 | 0.313 | 4.04E-13 |
| AC073283.7 | DCDC2B | 0.313 | 4.11E-13 |
| AC073283.7 | TTF2 | 0.313 | 3.65E-13 |
| AC073283.7 | SPDYE1 | 0.313 | 4.00E-13 |
| AC073283.7 | CHKB | 0.313 | 3.87E-13 |
| AC073283.7 | LRP8 | 0.313 | 3.72E-13 |
| AC073283.7 | TRIO | 0.313 | 4.03E-13 |
| AC073283.7 | ADAT2 | 0.314 | 3.12E-13 |
| AC073283.7 | RAB22A | 0.314 | 3.28E-13 |
| AC073283.7 | ARFGEF2 | 0.314 | 3.26E-13 |
| AC073283.7 | SUPT20H | 0.314 | 3.17E-13 |
| AC073283.7 | GRIN2D | 0.314 | 3.17E-13 |
| AC073283.7 | TAS2R38 | 0.314 | 2.97E-13 |
| AC073283.7 | CEP152 | 0.314 | 3.21E-13 |
| AC073283.7 | NR6A1 | 0.314 | 3.18E-13 |
| AC073283.7 | RBM5 | 0.314 | 3.13E-13 |
| AC073283.7 | SLC26A8 | 0.314 | 3.37E-13 |
| AC073283.7 | MED14 | 0.314 | 3.32E-13 |
| AC073283.7 | NPHP4 | 0.314 | 3.04E-13 |
| AC073283.7 | CIT | 0.314 | 2.97E-13 |
| AC073283.7 | POU2F1 | 0.315 | 2.53E-13 |
| AC073283.7 | LAT | 0.315 | 2.45E-13 |
| AC073283.7 | ARGLU1 | 0.315 | 2.45E-13 |
| AC073283.7 | ZNF785 | 0.315 | 2.69E-13 |
| AC073283.7 | N4BP2L2 | 0.315 | 2.73E-13 |
| AC073283.7 | ALOX12 | 0.315 | 2.69E-13 |
| AC073283.7 | CELA2B | 0.315 | 2.58E-13 |
| AC073283.7 | NSUN6 | 0.316 | 2.35E-13 |
| AC073283.7 | SYNGAP1 | 0.316 | 2.24E-13 |
| AC073283.7 | KRTAP5-1 | 0.316 | 2.12E-13 |
| AC073283.7 | JADE3 | 0.316 | 2.11E-13 |
| AC073283.7 | KCTD7 | 0.316 | 2.08E-13 |
| AC073283.7 | RP11-468E2.4 | 0.317 | 1.87E-13 |
| AC073283.7 | ATAD5 | 0.317 | 1.83E-13 |
| AC073283.7 | USP36 | 0.318 | 1.40E-13 |
| AC073283.7 | AHI1 | 0.318 | 1.51E-13 |
| AC073283.7 | ERICH6B | 0.318 | 1.48E-13 |
| AC073283.7 | NCR3LG1 | 0.318 | 1.63E-13 |
| AC073283.7 | ABL2 | 0.319 | 1.18E-13 |
| AC073283.7 | RBM26 | 0.319 | 1.33E-13 |
| AC073283.7 | REV1 | 0.319 | 1.35E-13 |
| AC073283.7 | RBM39 | 0.319 | 1.19E-13 |
| AC073283.7 | AC231657.1 | 0.319 | 1.39E-13 |
| AC073283.7 | TMEM63A | 0.319 | 1.37E-13 |
| AC073283.7 | SH3D21 | 0.319 | 1.30E-13 |
| AC073283.7 | LHX4 | 0.319 | 1.36E-13 |
| AC073283.7 | ZGRF1 | 0.319 | 1.21E-13 |
| AC073283.7 | UVSSA | 0.32 | 1.16E-13 |
| AC073283.7 | LRRC37B | 0.32 | 1.01E-13 |
| AC073283.7 | PROSER1 | 0.32 | 1.09E-13 |
| AC073283.7 | NOD1 | 0.32 | 1.02E-13 |
| AC073283.7 | ACRC | 0.32 | 1.01E-13 |
| AC073283.7 | ZXDC | 0.321 | 9.54E-14 |
| AC073283.7 | TRIM56 | 0.321 | 9.05E-14 |
| AC073283.7 | MTHFSD | 0.321 | 8.28E-14 |
| AC073283.7 | CCDC14 | 0.321 | 8.29E-14 |
| AC073283.7 | ZNF862 | 0.321 | 8.39E-14 |
| AC073283.7 | PAN3 | 0.321 | 8.87E-14 |
| AC073283.7 | SPATA13 | 0.321 | 8.34E-14 |
| AC073283.7 | RBM19 | 0.322 | 7.83E-14 |
| AC073283.7 | TTC21A | 0.322 | 7.95E-14 |
| AC073283.7 | XPO4 | 0.322 | 7.58E-14 |
| AC073283.7 | ANKRD36C | 0.322 | 6.97E-14 |
| AC073283.7 | ANKRD10 | 0.322 | 7.03E-14 |
| AC073283.7 | PRRC2C | 0.322 | 7.57E-14 |
| AC073283.7 | PRPF40B | 0.322 | 7.31E-14 |
| AC073283.7 | ACVR2B | 0.322 | 7.40E-14 |
| AC073283.7 | CEP250 | 0.322 | 7.22E-14 |
| AC073283.7 | BTAF1 | 0.322 | 7.50E-14 |
| AC073283.7 | RP11-444E17.6 | 0.322 | 7.02E-14 |
| AC073283.7 | JMJD7 | 0.322 | 7.50E-14 |
| AC073283.7 | ANKS3 | 0.323 | 5.76E-14 |
| AC073283.7 | NCOA3 | 0.323 | 6.12E-14 |
| AC073283.7 | EIF4A1 | 0.323 | 6.39E-14 |
| AC073283.7 | ANKZF1 | 0.323 | 6.14E-14 |
| AC073283.7 | FANCA | 0.323 | 6.18E-14 |
| AC073283.7 | CTC-479C5.12 | 0.323 | 6.17E-14 |
| AC073283.7 | C9orf173 | 0.323 | 5.90E-14 |
| AC073283.7 | NPIPA1 | 0.324 | 4.98E-14 |
| AC073283.7 | AC090154.1 | 0.324 | 4.60E-14 |
| AC073283.7 | CNTRL | 0.324 | 5.25E-14 |
| AC073283.7 | FAM193B | 0.324 | 4.73E-14 |
| AC073283.7 | PLK5 | 0.324 | 5.49E-14 |
| AC073283.7 | DCLRE1C | 0.324 | 5.17E-14 |
| AC073283.7 | UBE2V1 | 0.325 | 4.53E-14 |
| AC073283.7 | BNIPL | 0.325 | 3.80E-14 |
| AC073283.7 | RP11-691N7.6 | 0.325 | 4.27E-14 |
| AC073283.7 | CAMSAP1 | 0.325 | 4.00E-14 |
| AC073283.7 | USP6NL | 0.325 | 4.47E-14 |
| AC073283.7 | ALMS1 | 0.326 | 3.31E-14 |
| AC073283.7 | PLCG1 | 0.326 | 3.66E-14 |
| AC073283.7 | ZNF449 | 0.326 | 3.39E-14 |
| AC073283.7 | TRIM17 | 0.327 | 2.89E-14 |
| AC073283.7 | CBL | 0.327 | 3.03E-14 |
| AC073283.7 | ZNF445 | 0.327 | 2.73E-14 |
| AC073283.7 | JRKL | 0.327 | 2.82E-14 |
| AC073283.7 | RP11-111M22.2 | 0.328 | 2.25E-14 |
| AC073283.7 | SUN1 | 0.328 | 2.43E-14 |
| AC073283.7 | EP400 | 0.328 | 2.28E-14 |
| AC073283.7 | SRRM2 | 0.328 | 2.27E-14 |
| AC073283.7 | COL27A1 | 0.328 | 2.43E-14 |
| AC073283.7 | KMT2D | 0.328 | 2.57E-14 |
| AC073283.7 | RP11-723O4.6 | 0.328 | 2.19E-14 |
| AC073283.7 | EGFL8 | 0.329 | 1.98E-14 |
| AC073283.7 | HSF4 | 0.329 | 2.10E-14 |
| AC073283.7 | ATG9B | 0.329 | 1.94E-14 |
| AC073283.7 | TUBGCP3 | 0.329 | 2.05E-14 |
| AC073283.7 | PCF11 | 0.329 | 1.78E-14 |
| AC073283.7 | FAM208B | 0.33 | 1.75E-14 |
| AC073283.7 | USP49 | 0.33 | 1.46E-14 |
| AC073283.7 | ATF7IP | 0.331 | 1.38E-14 |
| AC073283.7 | UHRF1BP1 | 0.331 | 1.23E-14 |
| AC073283.7 | HECTD4 | 0.331 | 1.44E-14 |
| AC073283.7 | TTC3 | 0.331 | 1.22E-14 |
| AC073283.7 | GCNT7 | 0.331 | 1.45E-14 |
| AC073283.7 | KMT2A | 0.331 | 1.34E-14 |
| AC073283.7 | TET3 | 0.332 | 1.11E-14 |
| AC073283.7 | ATXN2 | 0.332 | 1.11E-14 |
| AC073283.7 | ANKRD11 | 0.332 | 1.04E-14 |
| AC073283.7 | RP4-583P15.15 | 0.332 | 1.09E-14 |
| AC073283.7 | DIDO1 | 0.332 | 1.03E-14 |
| AC073283.7 | MAPK8IP3 | 0.332 | 1.16E-14 |
| AC073283.7 | PSMG4 | 0.333 | 9.55E-15 |
| AC073283.7 | DNASE1 | 0.333 | 9.77E-15 |
| AC073283.7 | TUBA3D | 0.333 | 9.40E-15 |
| AC073283.7 | PNISR | 0.333 | 9.58E-15 |
| AC073283.7 | SLC7A1 | 0.333 | 8.87E-15 |
| AC073283.7 | TMEM262 | 0.333 | 9.63E-15 |
| AC073283.7 | RPGR | 0.334 | 7.81E-15 |
| AC073283.7 | BCL11B | 0.334 | 7.15E-15 |
| AC073283.7 | SENP5 | 0.334 | 6.75E-15 |
| AC073283.7 | IKBKB | 0.335 | 6.40E-15 |
| AC073283.7 | ZNF746 | 0.335 | 6.09E-15 |
| AC073283.7 | SZT2 | 0.335 | 6.66E-15 |
| AC073283.7 | RPA4 | 0.335 | 6.44E-15 |
| AC073283.7 | BTBD16 | 0.335 | 6.44E-15 |
| AC073283.7 | ZNF280C | 0.335 | 6.32E-15 |
| AC073283.7 | KIAA2026 | 0.335 | 5.69E-15 |
| AC073283.7 | TARBP1 | 0.335 | 6.20E-15 |
| AC073283.7 | AGAP5 | 0.335 | 6.40E-15 |
| AC073283.7 | DCUN1D2 | 0.335 | 5.99E-15 |
| AC073283.7 | ZFC3H1 | 0.336 | 4.74E-15 |
| AC073283.7 | KMT2C | 0.336 | 4.66E-15 |
| AC073283.7 | CRYGS | 0.336 | 5.33E-15 |
| AC073283.7 | RBM14-RBM4 | 0.336 | 5.06E-15 |
| AC073283.7 | NUTM2G | 0.336 | 5.33E-15 |
| AC073283.7 | ASXL1 | 0.337 | 4.30E-15 |
| AC073283.7 | PCNXL2 | 0.337 | 3.84E-15 |
| AC073283.7 | KIF24 | 0.337 | 3.91E-15 |
| AC073283.7 | AARSD1 | 0.337 | 3.96E-15 |
| AC073283.7 | PPP1R3E | 0.337 | 3.72E-15 |
| AC073283.7 | PABPC1L | 0.337 | 4.14E-15 |
| AC073283.7 | CCNL1 | 0.337 | 3.96E-15 |
| AC073283.7 | WFDC13 | 0.338 | 3.31E-15 |
| AC073283.7 | AGAP9 | 0.338 | 3.42E-15 |
| AC073283.7 | C5orf45 | 0.338 | 3.03E-15 |
| AC073283.7 | RLIM | 0.338 | 3.68E-15 |
| AC073283.7 | SCLY | 0.338 | 3.21E-15 |
| AC073283.7 | PPT2-EGFL8 | 0.338 | 3.29E-15 |
| AC073283.7 | SPTY2D1-AS1 | 0.338 | 3.10E-15 |
| AC073283.7 | SPICE1 | 0.339 | 2.87E-15 |
| AC073283.7 | STK36 | 0.339 | 2.85E-15 |
| AC073283.7 | RP11-411B6.6 | 0.339 | 2.56E-15 |
| AC073283.7 | RP11-11N7.5 | 0.339 | 2.56E-15 |
| AC073283.7 | PAN2 | 0.34 | 2.44E-15 |
| AC073283.7 | PRDM15 | 0.34 | 2.25E-15 |
| AC073283.7 | EP400NL | 0.34 | 2.11E-15 |
| AC073283.7 | CRAMP1 | 0.34 | 2.46E-15 |
| AC073283.7 | TP53TG5 | 0.34 | 2.35E-15 |
| AC073283.7 | ZNF37A | 0.34 | 2.43E-15 |
| AC073283.7 | PAPD7 | 0.341 | 1.97E-15 |
| AC073283.7 | XRCC2 | 0.341 | 2.02E-15 |
| AC073283.7 | ZNF354B | 0.341 | 1.67E-15 |
| AC073283.7 | ARHGAP11B | 0.341 | 1.78E-15 |
| AC073283.7 | PVRIG | 0.341 | 1.87E-15 |
| AC073283.7 | NBPF12 | 0.341 | 1.68E-15 |
| AC073283.7 | C11orf42 | 0.342 | 1.45E-15 |
| AC073283.7 | ANKRD36B | 0.342 | 1.58E-15 |
| AC073283.7 | PAXIP1 | 0.342 | 1.41E-15 |
| AC073283.7 | OFD1 | 0.342 | 1.54E-15 |
| AC073283.7 | MACC1 | 0.342 | 1.40E-15 |
| AC073283.7 | NOM1 | 0.343 | 1.19E-15 |
| AC073283.7 | CCNL2 | 0.343 | 1.28E-15 |
| AC073283.7 | GRIPAP1 | 0.343 | 1.24E-15 |
| AC073283.7 | MDN1 | 0.344 | 1.07E-15 |
| AC073283.7 | C8orf44 | 0.344 | 9.52E-16 |
| AC073283.7 | CHD7 | 0.344 | 1.02E-15 |
| AC073283.7 | LARP4B | 0.344 | 1.01E-15 |
| AC073283.7 | RP11-894J14.5 | 0.344 | 9.71E-16 |
| AC073283.7 | ZNF81 | 0.344 | 9.84E-16 |
| AC073283.7 | KCNC4 | 0.345 | 8.01E-16 |
| AC073283.7 | C15orf62 | 0.345 | 7.93E-16 |
| AC073283.7 | SS18L1 | 0.345 | 8.24E-16 |
| AC073283.7 | SGK494 | 0.345 | 8.32E-16 |
| AC073283.7 | TAB3 | 0.345 | 8.08E-16 |
| AC073283.7 | THOC2 | 0.346 | 6.27E-16 |
| AC073283.7 | TNRC6A | 0.346 | 6.60E-16 |
| AC073283.7 | CATSPER2 | 0.346 | 7.19E-16 |
| AC073283.7 | CCDC93 | 0.347 | 5.59E-16 |
| AC073283.7 | TAS2R5 | 0.347 | 5.17E-16 |
| AC073283.7 | TRRAP | 0.347 | 5.25E-16 |
| AC073283.7 | BRCA2 | 0.347 | 5.27E-16 |
| AC073283.7 | ARHGEF7 | 0.347 | 5.48E-16 |
| AC073283.7 | MTCP1 | 0.348 | 4.37E-16 |
| AC073283.7 | TTLL2 | 0.348 | 4.91E-16 |
| AC073283.7 | CAPRIN2 | 0.348 | 4.01E-16 |
| AC073283.7 | WDFY2 | 0.348 | 4.23E-16 |
| AC073283.7 | FAM122C | 0.348 | 4.17E-16 |
| AC073283.7 | C6orf25 | 0.349 | 3.75E-16 |
| AC073283.7 | TAS2R20 | 0.349 | 3.29E-16 |
| AC073283.7 | CREBZF | 0.349 | 3.55E-16 |
| AC073283.7 | ZNF182 | 0.349 | 3.42E-16 |
| AC073283.7 | NUP62CL | 0.35 | 2.92E-16 |
| AC073283.7 | SLFNL1 | 0.35 | 2.92E-16 |
| AC073283.7 | ANKRD36 | 0.35 | 3.06E-16 |
| AC073283.7 | IQGAP3 | 0.351 | 2.28E-16 |
| AC073283.7 | CLDN20 | 0.351 | 2.23E-16 |
| AC073283.7 | R3HDML | 0.351 | 2.27E-16 |
| AC073283.7 | ATR | 0.352 | 1.98E-16 |
| AC073283.7 | TMPRSS9 | 0.352 | 1.79E-16 |
| AC073283.7 | ANKS6 | 0.352 | 1.81E-16 |
| AC073283.7 | FAM156A | 0.352 | 1.95E-16 |
| AC073283.7 | CYP4F3 | 0.352 | 2.05E-16 |
| AC073283.7 | CCDC150 | 0.353 | 1.64E-16 |
| AC073283.7 | ACCS | 0.353 | 1.67E-16 |
| AC073283.7 | SFI1 | 0.354 | 1.26E-16 |
| AC073283.7 | POLQ | 0.354 | 1.39E-16 |
| AC073283.7 | SPDYE6 | 0.354 | 1.28E-16 |
| AC073283.7 | AP006285.2 | 0.354 | 1.36E-16 |
| AC073283.7 | SMG1 | 0.354 | 1.25E-16 |
| AC073283.7 | TRMT10B | 0.355 | 1.01E-16 |
| AC073283.7 | AGO2 | 0.355 | 1.06E-16 |
| AC073283.7 | SEC61A2 | 0.355 | 9.79E-17 |
| AC073283.7 | CCDC191 | 0.355 | 9.39E-17 |
| AC073283.7 | NBPF9 | 0.356 | 8.92E-17 |
| AC073283.7 | CAPN10 | 0.356 | 8.71E-17 |
| AC073283.7 | CCDC120 | 0.356 | 7.66E-17 |
| AC073283.7 | FCHSD1 | 0.357 | 6.62E-17 |
| AC073283.7 | LY6G5B | 0.357 | 6.22E-17 |
| AC073283.7 | GIGYF1 | 0.357 | 6.82E-17 |
| AC073283.7 | TSSK3 | 0.358 | 6.00E-17 |
| AC073283.7 | POLR2J3 | 0.359 | 4.61E-17 |
| AC073283.7 | UPF3A | 0.359 | 4.31E-17 |
| AC073283.7 | LTB4R2 | 0.359 | 4.73E-17 |
| AC073283.7 | GAN | 0.359 | 4.50E-17 |
| AC073283.7 | TAS2R4 | 0.36 | 3.52E-17 |
| AC073283.7 | CEP295 | 0.36 | 3.64E-17 |
| AC073283.7 | LY6G6F | 0.361 | 2.71E-17 |
| AC073283.7 | KNTC1 | 0.361 | 2.59E-17 |
| AC073283.7 | PLA2G4B | 0.362 | 2.12E-17 |
| AC073283.7 | KCNJ14 | 0.362 | 2.47E-17 |
| AC073283.7 | MSH5-SAPCD1 | 0.362 | 2.56E-17 |
| AC073283.7 | CBWD3 | 0.363 | 1.90E-17 |
| AC073283.7 | TTLL3 | 0.363 | 1.78E-17 |
| AC073283.7 | CFAP157 | 0.363 | 1.76E-17 |
| AC073283.7 | PLEKHA8 | 0.363 | 1.82E-17 |
| AC073283.7 | SPDYE5 | 0.363 | 1.69E-17 |
| AC073283.7 | CBWD5 | 0.363 | 1.71E-17 |
| AC073283.7 | GPR75 | 0.364 | 1.54E-17 |
| AC073283.7 | RP4-614O4.11 | 0.364 | 1.47E-17 |
| AC073283.7 | UBAP1L | 0.364 | 1.48E-17 |
| AC073283.7 | RTEL1 | 0.364 | 1.53E-17 |
| AC073283.7 | ZNF841 | 0.364 | 1.57E-17 |
| AC073283.7 | CBWD7 | 0.365 | 1.29E-17 |
| AC073283.7 | SEC31B | 0.366 | 1.01E-17 |
| AC073283.7 | SPDYE2 | 0.366 | 1.05E-17 |
| AC073283.7 | DNAH17 | 0.366 | 9.47E-18 |
| AC073283.7 | USP31 | 0.366 | 9.54E-18 |
| AC073283.7 | FAM188B | 0.366 | 8.79E-18 |
| AC073283.7 | KLHDC4 | 0.367 | 8.32E-18 |
| AC073283.7 | CEP164 | 0.367 | 7.61E-18 |
| AC073283.7 | ZBTB43 | 0.367 | 8.13E-18 |
| AC073283.7 | WWC3 | 0.368 | 6.24E-18 |
| AC073283.7 | ORAOV1 | 0.368 | 6.02E-18 |
| AC073283.7 | STX16-NPEPL1 | 0.368 | 6.29E-18 |
| AC073283.7 | ZNF621 | 0.368 | 5.98E-18 |
| AC073283.7 | PASK | 0.368 | 6.92E-18 |
| AC073283.7 | TSC1 | 0.368 | 6.03E-18 |
| AC073283.7 | JMJD7-PLA2G4B | 0.368 | 6.96E-18 |
| AC073283.7 | ZC3H12B | 0.369 | 4.53E-18 |
| AC073283.7 | WDR60 | 0.369 | 5.26E-18 |
| AC073283.7 | KCNMB3 | 0.37 | 3.63E-18 |
| AC073283.7 | PTPDC1 | 0.37 | 3.95E-18 |
| AC073283.7 | RBM6 | 0.37 | 4.26E-18 |
| AC073283.7 | ARL13A | 0.371 | 3.19E-18 |
| AC073283.7 | DCST1 | 0.371 | 3.57E-18 |
| AC073283.7 | CFAP44 | 0.371 | 3.40E-18 |
| AC073283.7 | SFSWAP | 0.371 | 2.91E-18 |
| AC073283.7 | AMER1 | 0.371 | 3.21E-18 |
| AC073283.7 | SAPCD1 | 0.371 | 3.49E-18 |
| AC073283.7 | STX16 | 0.372 | 2.57E-18 |
| AC073283.7 | ZNF337 | 0.372 | 2.78E-18 |
| AC073283.7 | YEATS2 | 0.372 | 2.82E-18 |
| AC073283.7 | SLC25A27 | 0.372 | 2.64E-18 |
| AC073283.7 | UBAP2 | 0.372 | 2.52E-18 |
| AC073283.7 | YY2 | 0.373 | 2.14E-18 |
| AC073283.7 | RNF32 | 0.373 | 1.91E-18 |
| AC073283.7 | SUGP2 | 0.373 | 2.30E-18 |
| AC073283.7 | RP3-461F17.3 | 0.373 | 2.12E-18 |
| AC073283.7 | TCTE3 | 0.374 | 1.73E-18 |
| AC073283.7 | ANKRD18A | 0.375 | 1.43E-18 |
| AC073283.7 | CENPJ | 0.376 | 1.00E-18 |
| AC073283.7 | CCDC84 | 0.376 | 1.07E-18 |
| AC073283.7 | LENG8 | 0.376 | 1.02E-18 |
| AC073283.7 | HAUS7 | 0.377 | 8.79E-19 |
| AC073283.7 | HUWE1 | 0.378 | 7.19E-19 |
| AC073283.7 | MECP2 | 0.379 | 5.09E-19 |
| AC073283.7 | TSEN2 | 0.38 | 3.80E-19 |
| AC073283.7 | DGKH | 0.38 | 3.80E-19 |
| AC073283.7 | OSBPL3 | 0.38 | 3.80E-19 |
| AC073283.7 | ZNF26 | 0.38 | 4.05E-19 |
| AC073283.7 | DDX39B | 0.381 | 3.15E-19 |
| AC073283.7 | UBN2 | 0.381 | 3.35E-19 |
| AC073283.7 | ALS2CL | 0.382 | 2.71E-19 |
| AC073283.7 | ZNF783 | 0.383 | 1.97E-19 |
| AC073283.7 | EBLN2 | 0.383 | 2.08E-19 |
| AC073283.7 | PHF8 | 0.383 | 1.89E-19 |
| AC073283.7 | SETD5 | 0.384 | 1.81E-19 |
| AC073283.7 | RBM28 | 0.385 | 1.26E-19 |
| AC073283.7 | NFAT5 | 0.385 | 1.31E-19 |
| AC073283.7 | PILRB | 0.385 | 1.20E-19 |
| AC073283.7 | DMTF1 | 0.386 | 1.16E-19 |
| AC073283.7 | KCNH8 | 0.386 | 1.15E-19 |
| AC073283.7 | ANKRD61 | 0.387 | 7.67E-20 |
| AC073283.7 | NPIPB4 | 0.387 | 7.46E-20 |
| AC073283.7 | ALKBH6 | 0.389 | 5.16E-20 |
| AC073283.7 | ARR3 | 0.39 | 3.98E-20 |
| AC073283.7 | ANKHD1 | 0.392 | 2.53E-20 |
| AC073283.7 | ZNF589 | 0.392 | 2.57E-20 |
| AC073283.7 | ATP11A | 0.393 | 2.05E-20 |
| AC073283.7 | RP11-434D12.1 | 0.394 | 1.49E-20 |
| AC073283.7 | FNBP4 | 0.394 | 1.48E-20 |
| AC073283.7 | RBM41 | 0.395 | 1.37E-20 |
| AC073283.7 | PRR5-ARHGAP8 | 0.395 | 1.25E-20 |
| AC073283.7 | C20orf144 | 0.397 | 6.77E-21 |
| AC073283.7 | TAF1 | 0.397 | 7.84E-21 |
| AC073283.7 | CLCN5 | 0.398 | 6.51E-21 |
| AC073283.7 | ATAT1 | 0.398 | 6.43E-21 |
| AC073283.7 | PROZ | 0.399 | 4.41E-21 |
| AC073283.7 | ZNF782 | 0.4 | 3.95E-21 |
| AC073283.7 | KLHL31 | 0.4 | 3.52E-21 |
| AC073283.7 | PLXNA3 | 0.401 | 2.94E-21 |
| AC073283.7 | BBS1 | 0.402 | 2.02E-21 |
| AC073283.7 | TRIM66 | 0.402 | 2.19E-21 |
| AC073283.7 | ZNF75D | 0.402 | 2.31E-21 |
| AC073283.7 | DNHD1 | 0.403 | 1.84E-21 |
| AC073283.7 | TRIM52 | 0.403 | 1.81E-21 |
| AC073283.7 | FAM186B | 0.405 | 1.02E-21 |
| AC073283.7 | KIAA0907 | 0.405 | 1.05E-21 |
| AC073283.7 | RAD54L2 | 0.406 | 7.14E-22 |
| AC073283.7 | NKTR | 0.407 | 6.21E-22 |
| AC073283.7 | PRSS53 | 0.407 | 6.83E-22 |
| AC073283.7 | L3MBTL1 | 0.407 | 5.84E-22 |
| AC073283.7 | FAM122B | 0.408 | 4.99E-22 |
| AC073283.7 | TIAF1 | 0.409 | 4.24E-22 |
| AC073283.7 | C9orf50 | 0.41 | 3.19E-22 |
| AC073283.7 | KIAA1549 | 0.413 | 1.44E-22 |
| AC073283.7 | SLC13A4 | 0.413 | 1.52E-22 |
| AC073283.7 | ZNF275 | 0.418 | 3.58E-23 |
| AC073283.7 | ARHGAP8 | 0.418 | 3.31E-23 |
| AC073283.7 | MFSD14C | 0.418 | 3.48E-23 |
| AC073283.7 | AGAP6 | 0.419 | 2.74E-23 |
| AC073283.7 | ZNF23 | 0.419 | 3.11E-23 |
| AC073283.7 | AHSA2 | 0.42 | 2.51E-23 |
| AC073283.7 | AGAP4 | 0.421 | 1.82E-23 |
| AC073283.7 | ANKRD23 | 0.422 | 1.26E-23 |
| AC073283.7 | ZNF789 | 0.426 | 4.47E-24 |
| AC073283.7 | WDR27 | 0.427 | 3.40E-24 |
| AC073283.7 | ANKMY1 | 0.428 | 2.43E-24 |
| AC073283.7 | DNAH1 | 0.433 | 7.25E-25 |
| AC073283.7 | RBM33 | 0.433 | 7.53E-25 |
| AC073283.7 | GABRE | 0.439 | 1.29E-25 |
| AC073283.7 | ZNF514 | 0.443 | 3.60E-26 |
| AC073283.7 | RP11-33O4.2 | 0.449 | 7.06E-27 |
| AC073283.7 | MSH5 | 0.455 | 1.32E-27 |
| AC073283.7 | OGT | 0.483 | 1.88E-31 |
| AC073283.7 | C2orf61 | 0.815 | 2.63E-123 |
| CTC-273B12.10 | ENTPD5 | -0.368 | 5.83E-18 |
| CTC-273B12.10 | WDR78 | -0.363 | 1.89E-17 |
| CTC-273B12.10 | SULT1B1 | -0.354 | 1.32E-16 |
| CTC-273B12.10 | CPM | -0.353 | 1.62E-16 |
| CTC-273B12.10 | ANK3 | -0.337 | 4.13E-15 |
| CTC-273B12.10 | SLC41A2 | -0.334 | 7.31E-15 |
| CTC-273B12.10 | LIMA1 | -0.328 | 2.41E-14 |
| CTC-273B12.10 | KIT | -0.321 | 8.02E-14 |
| CTC-273B12.10 | SLC35D1 | -0.318 | 1.61E-13 |
| CTC-273B12.10 | LPP | -0.317 | 1.81E-13 |
| CTC-273B12.10 | MIER1 | -0.316 | 2.19E-13 |
| CTC-273B12.10 | ETFDH | -0.316 | 2.07E-13 |
| CTC-273B12.10 | PPM1A | -0.315 | 2.55E-13 |
| CTC-273B12.10 | PPP2R3A | -0.314 | 3.12E-13 |
| CTC-273B12.10 | GRAMD3 | -0.314 | 3.14E-13 |
| CTC-273B12.10 | MARCKS | -0.313 | 4.03E-13 |
| CTC-273B12.10 | SOS2 | -0.312 | 4.73E-13 |
| CTC-273B12.10 | P2RY1 | -0.311 | 5.54E-13 |
| CTC-273B12.10 | PDE3A | -0.311 | 5.87E-13 |
| CTC-273B12.10 | PRKACB | -0.311 | 5.39E-13 |
| CTC-273B12.10 | BMX | -0.311 | 5.77E-13 |
| CTC-273B12.10 | MAGI3 | -0.31 | 6.06E-13 |
| CTC-273B12.10 | LIFR | -0.31 | 6.39E-13 |
| CTC-273B12.10 | HEATR5A | -0.309 | 7.99E-13 |
| CTC-273B12.10 | DDX60L | -0.309 | 7.83E-13 |
| CTC-273B12.10 | NEGR1 | -0.308 | 8.89E-13 |
| CTC-273B12.10 | LUZP1 | -0.308 | 9.30E-13 |
| CTC-273B12.10 | PIRT | -0.307 | 1.17E-12 |
| CTC-273B12.10 | PPP1R12B | -0.307 | 1.17E-12 |
| CTC-273B12.10 | UGP2 | -0.307 | 1.06E-12 |
| CTC-273B12.10 | KCTD9 | -0.306 | 1.24E-12 |
| CTC-273B12.10 | METTL7A | -0.306 | 1.22E-12 |
| CTC-273B12.10 | ADGRL3 | -0.306 | 1.34E-12 |
| CTC-273B12.10 | CPEB2 | -0.305 | 1.65E-12 |
| CTC-273B12.10 | PAFAH2 | -0.304 | 1.89E-12 |
| CTC-273B12.10 | NBEAL1 | -0.304 | 1.89E-12 |
| CTC-273B12.10 | PJA2 | -0.304 | 1.81E-12 |
| CTC-273B12.10 | UNC5C | -0.303 | 2.08E-12 |
| CTC-273B12.10 | SMIM14 | -0.303 | 2.06E-12 |
| CTC-273B12.10 | HPGDS | -0.303 | 2.42E-12 |
| CTC-273B12.10 | BMP3 | -0.302 | 2.71E-12 |
| CTC-273B12.10 | GLP2R | -0.302 | 2.72E-12 |
| CTC-273B12.10 | GPBP1L1 | -0.301 | 3.36E-12 |
| CTC-273B12.10 | TP53INP1 | -0.301 | 3.26E-12 |
| CTC-273B12.10 | TMEM220 | -0.301 | 3.40E-12 |
| CTC-273B12.10 | NME1 | 0.301 | 3.00E-12 |
| CTC-273B12.10 | HGS | 0.301 | 3.11E-12 |
| CTC-273B12.10 | AKAP17A | 0.301 | 3.35E-12 |
| CTC-273B12.10 | FOXQ1 | 0.301 | 3.32E-12 |
| CTC-273B12.10 | C6orf48 | 0.301 | 3.06E-12 |
| CTC-273B12.10 | TUBG1 | 0.301 | 3.26E-12 |
| CTC-273B12.10 | MAP3K10 | 0.301 | 2.93E-12 |
| CTC-273B12.10 | NPRL2 | 0.301 | 3.34E-12 |
| CTC-273B12.10 | SNRNP70 | 0.301 | 3.41E-12 |
| CTC-273B12.10 | SDCCAG3 | 0.301 | 3.29E-12 |
| CTC-273B12.10 | DDX56 | 0.301 | 3.16E-12 |
| CTC-273B12.10 | LRP3 | 0.302 | 2.78E-12 |
| CTC-273B12.10 | TOMM40 | 0.302 | 2.78E-12 |
| CTC-273B12.10 | DUS1L | 0.302 | 2.71E-12 |
| CTC-273B12.10 | SNRPA | 0.302 | 2.71E-12 |
| CTC-273B12.10 | PRELID3A | 0.302 | 2.57E-12 |
| CTC-273B12.10 | RPS5 | 0.302 | 2.83E-12 |
| CTC-273B12.10 | RPS28 | 0.302 | 2.83E-12 |
| CTC-273B12.10 | TRIM28 | 0.302 | 2.53E-12 |
| CTC-273B12.10 | SDHAF1 | 0.302 | 2.73E-12 |
| CTC-273B12.10 | H2AFX | 0.302 | 2.68E-12 |
| CTC-273B12.10 | HSD17B10 | 0.302 | 2.66E-12 |
| CTC-273B12.10 | DCUN1D2 | 0.302 | 2.72E-12 |
| CTC-273B12.10 | CCDC189 | 0.303 | 2.25E-12 |
| CTC-273B12.10 | ATP6V1F | 0.303 | 2.24E-12 |
| CTC-273B12.10 | ZDHHC24 | 0.303 | 2.27E-12 |
| CTC-273B12.10 | RTKN | 0.303 | 2.11E-12 |
| CTC-273B12.10 | EIF3G | 0.303 | 2.44E-12 |
| CTC-273B12.10 | NELFA | 0.303 | 2.41E-12 |
| CTC-273B12.10 | SNRPD2 | 0.303 | 2.15E-12 |
| CTC-273B12.10 | UBAC2 | 0.303 | 2.24E-12 |
| CTC-273B12.10 | RPL18A | 0.303 | 2.14E-12 |
| CTC-273B12.10 | ASB6 | 0.303 | 2.36E-12 |
| CTC-273B12.10 | POP7 | 0.303 | 2.42E-12 |
| CTC-273B12.10 | FARSA | 0.303 | 2.09E-12 |
| CTC-273B12.10 | NTHL1 | 0.304 | 1.75E-12 |
| CTC-273B12.10 | ZNF414 | 0.304 | 1.79E-12 |
| CTC-273B12.10 | SSBP4 | 0.304 | 1.74E-12 |
| CTC-273B12.10 | MPV17L2 | 0.304 | 1.81E-12 |
| CTC-273B12.10 | RPL18 | 0.304 | 1.75E-12 |
| CTC-273B12.10 | ZNF205 | 0.304 | 2.05E-12 |
| CTC-273B12.10 | NARF | 0.304 | 2.01E-12 |
| CTC-273B12.10 | ZNF48 | 0.304 | 1.87E-12 |
| CTC-273B12.10 | RFNG | 0.304 | 1.98E-12 |
| CTC-273B12.10 | PFDN2 | 0.304 | 1.91E-12 |
| CTC-273B12.10 | C19orf25 | 0.304 | 1.81E-12 |
| CTC-273B12.10 | RRP9 | 0.304 | 1.78E-12 |
| CTC-273B12.10 | CDK5 | 0.304 | 1.97E-12 |
| CTC-273B12.10 | C1orf35 | 0.305 | 1.48E-12 |
| CTC-273B12.10 | ZSWIM1 | 0.305 | 1.55E-12 |
| CTC-273B12.10 | ASF1B | 0.305 | 1.57E-12 |
| CTC-273B12.10 | ARL16 | 0.305 | 1.47E-12 |
| CTC-273B12.10 | DNAAF5 | 0.305 | 1.70E-12 |
| CTC-273B12.10 | COLGALT1 | 0.306 | 1.43E-12 |
| CTC-273B12.10 | SSSCA1 | 0.306 | 1.35E-12 |
| CTC-273B12.10 | YIF1B | 0.306 | 1.36E-12 |
| CTC-273B12.10 | DHX30 | 0.306 | 1.24E-12 |
| CTC-273B12.10 | CBX2 | 0.306 | 1.44E-12 |
| CTC-273B12.10 | PDRG1 | 0.306 | 1.42E-12 |
| CTC-273B12.10 | PSMA7 | 0.306 | 1.33E-12 |
| CTC-273B12.10 | POLD1 | 0.306 | 1.26E-12 |
| CTC-273B12.10 | EXOSC4 | 0.306 | 1.28E-12 |
| CTC-273B12.10 | TMEM160 | 0.307 | 1.12E-12 |
| CTC-273B12.10 | UTP4 | 0.307 | 1.15E-12 |
| CTC-273B12.10 | BANP | 0.307 | 1.16E-12 |
| CTC-273B12.10 | MRGBP | 0.308 | 9.05E-13 |
| CTC-273B12.10 | CLASRP | 0.308 | 8.73E-13 |
| CTC-273B12.10 | CLPP | 0.308 | 9.28E-13 |
| CTC-273B12.10 | RPL36 | 0.308 | 9.50E-13 |
| CTC-273B12.10 | DCAF15 | 0.308 | 1.01E-12 |
| CTC-273B12.10 | TBCB | 0.308 | 9.15E-13 |
| CTC-273B12.10 | ASPSCR1 | 0.308 | 9.29E-13 |
| CTC-273B12.10 | TERT | 0.308 | 8.57E-13 |
| CTC-273B12.10 | PDF | 0.308 | 1.00E-12 |
| CTC-273B12.10 | LRFN4 | 0.309 | 7.94E-13 |
| CTC-273B12.10 | LAGE3 | 0.309 | 8.07E-13 |
| CTC-273B12.10 | C19orf53 | 0.309 | 8.07E-13 |
| CTC-273B12.10 | SAPCD2 | 0.309 | 7.79E-13 |
| CTC-273B12.10 | LPAR2 | 0.309 | 8.02E-13 |
| CTC-273B12.10 | WRNIP1 | 0.31 | 7.12E-13 |
| CTC-273B12.10 | MRPS12 | 0.31 | 6.27E-13 |
| CTC-273B12.10 | C17orf53 | 0.31 | 6.48E-13 |
| CTC-273B12.10 | POMGNT2 | 0.31 | 6.30E-13 |
| CTC-273B12.10 | HSPBP1 | 0.31 | 6.08E-13 |
| CTC-273B12.10 | BCS1L | 0.31 | 6.84E-13 |
| CTC-273B12.10 | RTN4RL2 | 0.31 | 6.79E-13 |
| CTC-273B12.10 | ENTHD2 | 0.31 | 6.92E-13 |
| CTC-273B12.10 | MYBL2 | 0.31 | 6.35E-13 |
| CTC-273B12.10 | HAUS5 | 0.31 | 6.42E-13 |
| CTC-273B12.10 | PDCD2L | 0.31 | 6.28E-13 |
| CTC-273B12.10 | SF3A2 | 0.31 | 6.51E-13 |
| CTC-273B12.10 | AGBL5 | 0.31 | 6.51E-13 |
| CTC-273B12.10 | IER5L | 0.311 | 5.48E-13 |
| CTC-273B12.10 | NOSIP | 0.311 | 5.53E-13 |
| CTC-273B12.10 | CPLX1 | 0.311 | 5.06E-13 |
| CTC-273B12.10 | GADD45GIP1 | 0.311 | 5.61E-13 |
| CTC-273B12.10 | CPSF4 | 0.311 | 5.63E-13 |
| CTC-273B12.10 | TMEM198 | 0.311 | 5.23E-13 |
| CTC-273B12.10 | C15orf61 | 0.312 | 4.91E-13 |
| CTC-273B12.10 | LMTK3 | 0.312 | 4.45E-13 |
| CTC-273B12.10 | PARD6A | 0.312 | 4.88E-13 |
| CTC-273B12.10 | ASPHD1 | 0.312 | 4.48E-13 |
| CTC-273B12.10 | SND1 | 0.312 | 4.71E-13 |
| CTC-273B12.10 | PDDC1 | 0.312 | 4.55E-13 |
| CTC-273B12.10 | DHPS | 0.312 | 4.26E-13 |
| CTC-273B12.10 | PRMT7 | 0.313 | 4.16E-13 |
| CTC-273B12.10 | TMEM259 | 0.313 | 3.87E-13 |
| CTC-273B12.10 | WDR18 | 0.313 | 4.05E-13 |
| CTC-273B12.10 | E2F4 | 0.313 | 3.85E-13 |
| CTC-273B12.10 | PYCR1 | 0.314 | 3.43E-13 |
| CTC-273B12.10 | TPD52L2 | 0.314 | 3.22E-13 |
| CTC-273B12.10 | THOP1 | 0.314 | 3.25E-13 |
| CTC-273B12.10 | NSUN5 | 0.314 | 3.20E-13 |
| CTC-273B12.10 | PRICKLE3 | 0.314 | 3.33E-13 |
| CTC-273B12.10 | UBE2M | 0.315 | 2.79E-13 |
| CTC-273B12.10 | MED27 | 0.315 | 2.81E-13 |
| CTC-273B12.10 | PPP4C | 0.315 | 2.82E-13 |
| CTC-273B12.10 | DRD4 | 0.315 | 2.78E-13 |
| CTC-273B12.10 | UFSP1 | 0.316 | 2.17E-13 |
| CTC-273B12.10 | WDR62 | 0.316 | 2.20E-13 |
| CTC-273B12.10 | MXD3 | 0.316 | 2.32E-13 |
| CTC-273B12.10 | PAM16 | 0.316 | 2.25E-13 |
| CTC-273B12.10 | C17orf96 | 0.316 | 2.23E-13 |
| CTC-273B12.10 | CARS2 | 0.317 | 1.85E-13 |
| CTC-273B12.10 | KATNB1 | 0.317 | 1.92E-13 |
| CTC-273B12.10 | C12orf10 | 0.317 | 1.68E-13 |
| CTC-273B12.10 | RPS2 | 0.317 | 1.89E-13 |
| CTC-273B12.10 | ELFN1 | 0.317 | 1.93E-13 |
| CTC-273B12.10 | DUS3L | 0.318 | 1.62E-13 |
| CTC-273B12.10 | CMTM8 | 0.318 | 1.53E-13 |
| CTC-273B12.10 | MFSD13A | 0.318 | 1.44E-13 |
| CTC-273B12.10 | UBALD1 | 0.318 | 1.43E-13 |
| CTC-273B12.10 | MRPL38 | 0.318 | 1.46E-13 |
| CTC-273B12.10 | TSEN54 | 0.319 | 1.26E-13 |
| CTC-273B12.10 | JMJD6 | 0.319 | 1.26E-13 |
| CTC-273B12.10 | NUDT1 | 0.319 | 1.19E-13 |
| CTC-273B12.10 | C19orf24 | 0.319 | 1.33E-13 |
| CTC-273B12.10 | BRAT1 | 0.319 | 1.37E-13 |
| CTC-273B12.10 | YDJC | 0.319 | 1.33E-13 |
| CTC-273B12.10 | FBL | 0.319 | 1.16E-13 |
| CTC-273B12.10 | TMEM147 | 0.32 | 1.08E-13 |
| CTC-273B12.10 | MZT2A | 0.32 | 1.11E-13 |
| CTC-273B12.10 | TMEM201 | 0.32 | 1.12E-13 |
| CTC-273B12.10 | ANKRD13D | 0.32 | 1.13E-13 |
| CTC-273B12.10 | ZBTB12 | 0.32 | 1.02E-13 |
| CTC-273B12.10 | RPLP2 | 0.32 | 1.14E-13 |
| CTC-273B12.10 | SPC24 | 0.321 | 8.71E-14 |
| CTC-273B12.10 | PRDM12 | 0.321 | 8.08E-14 |
| CTC-273B12.10 | TROAP | 0.321 | 9.36E-14 |
| CTC-273B12.10 | CCDC85B | 0.321 | 9.09E-14 |
| CTC-273B12.10 | ZFPM1 | 0.321 | 8.05E-14 |
| CTC-273B12.10 | SAC3D1 | 0.321 | 8.19E-14 |
| CTC-273B12.10 | POLRMT | 0.321 | 8.47E-14 |
| CTC-273B12.10 | XAB2 | 0.321 | 9.28E-14 |
| CTC-273B12.10 | LMNTD2 | 0.321 | 8.73E-14 |
| CTC-273B12.10 | POLR2H | 0.321 | 9.11E-14 |
| CTC-273B12.10 | GALK1 | 0.321 | 8.37E-14 |
| CTC-273B12.10 | RUVBL2 | 0.322 | 6.85E-14 |
| CTC-273B12.10 | CCNO | 0.322 | 7.93E-14 |
| CTC-273B12.10 | MALSU1 | 0.322 | 7.95E-14 |
| CTC-273B12.10 | C4orf48 | 0.322 | 6.99E-14 |
| CTC-273B12.10 | RHEBL1 | 0.322 | 7.38E-14 |
| CTC-273B12.10 | SAMD10 | 0.322 | 6.68E-14 |
| CTC-273B12.10 | PTDSS2 | 0.322 | 7.22E-14 |
| CTC-273B12.10 | ANAPC11 | 0.324 | 5.40E-14 |
| CTC-273B12.10 | PUS1 | 0.324 | 5.20E-14 |
| CTC-273B12.10 | SRM | 0.324 | 5.26E-14 |
| CTC-273B12.10 | WDR24 | 0.324 | 5.39E-14 |
| CTC-273B12.10 | ILKAP | 0.325 | 4.37E-14 |
| CTC-273B12.10 | LIME1 | 0.325 | 3.89E-14 |
| CTC-273B12.10 | KAT2A | 0.325 | 4.25E-14 |
| CTC-273B12.10 | NPRL3 | 0.325 | 3.80E-14 |
| CTC-273B12.10 | TIMM17B | 0.325 | 3.90E-14 |
| CTC-273B12.10 | NPEPL1 | 0.325 | 3.77E-14 |
| CTC-273B12.10 | CCBL1 | 0.326 | 3.59E-14 |
| CTC-273B12.10 | CDC34 | 0.326 | 3.27E-14 |
| CTC-273B12.10 | PMPCA | 0.327 | 2.81E-14 |
| CTC-273B12.10 | RPS15 | 0.327 | 2.64E-14 |
| CTC-273B12.10 | NAT9 | 0.327 | 2.93E-14 |
| CTC-273B12.10 | FTSJ1 | 0.327 | 2.84E-14 |
| CTC-273B12.10 | C9orf16 | 0.327 | 2.84E-14 |
| CTC-273B12.10 | MRPL55 | 0.328 | 2.33E-14 |
| CTC-273B12.10 | SPATA33 | 0.328 | 2.43E-14 |
| CTC-273B12.10 | GPS1 | 0.328 | 2.23E-14 |
| CTC-273B12.10 | PAQR4 | 0.328 | 2.56E-14 |
| CTC-273B12.10 | ALG3 | 0.328 | 2.30E-14 |
| CTC-273B12.10 | BYSL | 0.328 | 2.41E-14 |
| CTC-273B12.10 | STX10 | 0.329 | 1.83E-14 |
| CTC-273B12.10 | C19orf48 | 0.329 | 1.79E-14 |
| CTC-273B12.10 | MAD2L2 | 0.329 | 1.79E-14 |
| CTC-273B12.10 | MIIP | 0.329 | 2.09E-14 |
| CTC-273B12.10 | TSSK6 | 0.329 | 1.88E-14 |
| CTC-273B12.10 | ENKD1 | 0.329 | 2.02E-14 |
| CTC-273B12.10 | PRMT1 | 0.329 | 1.91E-14 |
| CTC-273B12.10 | ZNF771 | 0.33 | 1.69E-14 |
| CTC-273B12.10 | SIGMAR1 | 0.33 | 1.72E-14 |
| CTC-273B12.10 | POLD2 | 0.33 | 1.54E-14 |
| CTC-273B12.10 | KLHL17 | 0.33 | 1.70E-14 |
| CTC-273B12.10 | SLC39A3 | 0.33 | 1.65E-14 |
| CTC-273B12.10 | FUT1 | 0.33 | 1.70E-14 |
| CTC-273B12.10 | ECE2 | 0.331 | 1.32E-14 |
| CTC-273B12.10 | UBA52 | 0.331 | 1.34E-14 |
| CTC-273B12.10 | PLK1 | 0.331 | 1.22E-14 |
| CTC-273B12.10 | NOB1 | 0.332 | 1.17E-14 |
| CTC-273B12.10 | MRPL36 | 0.332 | 1.10E-14 |
| CTC-273B12.10 | TIMM50 | 0.332 | 1.14E-14 |
| CTC-273B12.10 | C16orf59 | 0.332 | 1.19E-14 |
| CTC-273B12.10 | TSEN34 | 0.333 | 9.73E-15 |
| CTC-273B12.10 | MRPL4 | 0.333 | 9.21E-15 |
| CTC-273B12.10 | DXO | 0.333 | 9.25E-15 |
| CTC-273B12.10 | RECQL4 | 0.333 | 8.94E-15 |
| CTC-273B12.10 | C20orf195 | 0.334 | 8.08E-15 |
| CTC-273B12.10 | TRAIP | 0.334 | 7.00E-15 |
| CTC-273B12.10 | PAFAH1B3 | 0.334 | 7.07E-15 |
| CTC-273B12.10 | DDX49 | 0.335 | 6.02E-15 |
| CTC-273B12.10 | RPS16 | 0.336 | 5.30E-15 |
| CTC-273B12.10 | CHTF18 | 0.336 | 5.44E-15 |
| CTC-273B12.10 | CAPN10 | 0.336 | 5.01E-15 |
| CTC-273B12.10 | POLR2I | 0.336 | 5.29E-15 |
| CTC-273B12.10 | FAM58A | 0.336 | 5.42E-15 |
| CTC-273B12.10 | ZMYND19 | 0.336 | 4.68E-15 |
| CTC-273B12.10 | RNF208 | 0.336 | 5.44E-15 |
| CTC-273B12.10 | ZNF581 | 0.336 | 5.18E-15 |
| CTC-273B12.10 | ANKRD39 | 0.336 | 4.83E-15 |
| CTC-273B12.10 | TPRA1 | 0.337 | 4.37E-15 |
| CTC-273B12.10 | EXOSC5 | 0.337 | 4.12E-15 |
| CTC-273B12.10 | TFAP4 | 0.338 | 3.67E-15 |
| CTC-273B12.10 | CEBPB | 0.338 | 3.67E-15 |
| CTC-273B12.10 | WDR74 | 0.338 | 3.29E-15 |
| CTC-273B12.10 | NPM3 | 0.338 | 3.16E-15 |
| CTC-273B12.10 | APBA3 | 0.339 | 2.98E-15 |
| CTC-273B12.10 | DPM2 | 0.34 | 2.43E-15 |
| CTC-273B12.10 | E4F1 | 0.34 | 2.42E-15 |
| CTC-273B12.10 | ALYREF | 0.34 | 2.36E-15 |
| CTC-273B12.10 | RPUSD1 | 0.341 | 1.95E-15 |
| CTC-273B12.10 | RHPN1 | 0.341 | 1.93E-15 |
| CTC-273B12.10 | DPP7 | 0.342 | 1.52E-15 |
| CTC-273B12.10 | ETV4 | 0.342 | 1.51E-15 |
| CTC-273B12.10 | RCCD1 | 0.342 | 1.60E-15 |
| CTC-273B12.10 | KIF18B | 0.342 | 1.45E-15 |
| CTC-273B12.10 | C19orf52 | 0.342 | 1.53E-15 |
| CTC-273B12.10 | PLOD3 | 0.342 | 1.62E-15 |
| CTC-273B12.10 | RELL2 | 0.343 | 1.33E-15 |
| CTC-273B12.10 | ARMC6 | 0.343 | 1.35E-15 |
| CTC-273B12.10 | AHCY | 0.343 | 1.23E-15 |
| CTC-273B12.10 | ERF | 0.343 | 1.33E-15 |
| CTC-273B12.10 | TMEM161A | 0.343 | 1.16E-15 |
| CTC-273B12.10 | SULT2B1 | 0.343 | 1.34E-15 |
| CTC-273B12.10 | RPL13 | 0.344 | 9.59E-16 |
| CTC-273B12.10 | NAT6 | 0.344 | 1.01E-15 |
| CTC-273B12.10 | RNASEH2A | 0.345 | 8.30E-16 |
| CTC-273B12.10 | PPP1R35 | 0.345 | 7.55E-16 |
| CTC-273B12.10 | ATG4B | 0.346 | 6.29E-16 |
| CTC-273B12.10 | RPL28 | 0.347 | 5.30E-16 |
| CTC-273B12.10 | COPS7B | 0.347 | 5.46E-16 |
| CTC-273B12.10 | DNASE1L2 | 0.348 | 4.26E-16 |
| CTC-273B12.10 | CCDC78 | 0.349 | 3.27E-16 |
| CTC-273B12.10 | E2F1 | 0.349 | 3.60E-16 |
| CTC-273B12.10 | FAM96B | 0.349 | 3.36E-16 |
| CTC-273B12.10 | CBX4 | 0.349 | 3.63E-16 |
| CTC-273B12.10 | EMD | 0.35 | 2.87E-16 |
| CTC-273B12.10 | NUTF2 | 0.35 | 2.93E-16 |
| CTC-273B12.10 | F12 | 0.35 | 2.66E-16 |
| CTC-273B12.10 | ZNF580 | 0.35 | 3.08E-16 |
| CTC-273B12.10 | CLDND2 | 0.351 | 2.63E-16 |
| CTC-273B12.10 | ZNF579 | 0.351 | 2.36E-16 |
| CTC-273B12.10 | HM13 | 0.352 | 2.00E-16 |
| CTC-273B12.10 | EMC8 | 0.352 | 1.88E-16 |
| CTC-273B12.10 | DPH7 | 0.353 | 1.47E-16 |
| CTC-273B12.10 | FBXW9 | 0.353 | 1.61E-16 |
| CTC-273B12.10 | CPNE7 | 0.354 | 1.36E-16 |
| CTC-273B12.10 | SUV39H1 | 0.355 | 9.47E-17 |
| CTC-273B12.10 | GNB1L | 0.355 | 1.08E-16 |
| CTC-273B12.10 | UBE2S | 0.356 | 7.73E-17 |
| CTC-273B12.10 | WDR83 | 0.357 | 6.61E-17 |
| CTC-273B12.10 | CDT1 | 0.357 | 6.73E-17 |
| CTC-273B12.10 | C11orf84 | 0.357 | 6.50E-17 |
| CTC-273B12.10 | PKMYT1 | 0.358 | 5.90E-17 |
| CTC-273B12.10 | LSM7 | 0.358 | 5.78E-17 |
| CTC-273B12.10 | INPP5E | 0.358 | 5.68E-17 |
| CTC-273B12.10 | C16orf13 | 0.359 | 4.67E-17 |
| CTC-273B12.10 | WRAP73 | 0.359 | 4.76E-17 |
| CTC-273B12.10 | DHX34 | 0.359 | 4.11E-17 |
| CTC-273B12.10 | PPP2R3B | 0.359 | 4.92E-17 |
| CTC-273B12.10 | DEF8 | 0.361 | 2.71E-17 |
| CTC-273B12.10 | TEAD4 | 0.361 | 2.99E-17 |
| CTC-273B12.10 | CHCHD6 | 0.362 | 2.43E-17 |
| CTC-273B12.10 | DDX39A | 0.362 | 2.59E-17 |
| CTC-273B12.10 | C17orf89 | 0.362 | 2.23E-17 |
| CTC-273B12.10 | PCGF1 | 0.364 | 1.54E-17 |
| CTC-273B12.10 | CTU2 | 0.365 | 1.20E-17 |
| CTC-273B12.10 | GTPBP3 | 0.365 | 1.20E-17 |
| CTC-273B12.10 | MFSD12 | 0.365 | 1.15E-17 |
| CTC-273B12.10 | C19orf60 | 0.365 | 1.10E-17 |
| CTC-273B12.10 | OXLD1 | 0.367 | 7.51E-18 |
| CTC-273B12.10 | HAGHL | 0.368 | 6.30E-18 |
| CTC-273B12.10 | NAA10 | 0.369 | 5.33E-18 |
| CTC-273B12.10 | RPS21 | 0.37 | 4.49E-18 |
| CTC-273B12.10 | KMT5C | 0.371 | 2.93E-18 |
| CTC-273B12.10 | TRMT1 | 0.375 | 1.26E-18 |
| CTC-273B12.10 | ANKRD13B | 0.376 | 1.04E-18 |
| CTC-273B12.10 | QPCTL | 0.378 | 6.09E-19 |
| CTC-273B12.10 | UCKL1 | 0.378 | 6.42E-19 |
| CTC-273B12.10 | SLCO4A1 | 0.38 | 4.16E-19 |
| CTC-273B12.10 | C19orf73 | 0.381 | 3.64E-19 |
| CTC-273B12.10 | ACD | 0.384 | 1.70E-19 |
| CTC-273B12.10 | MRPL57 | 0.384 | 1.65E-19 |
| CTC-273B12.10 | WDR54 | 0.387 | 7.79E-20 |
| CTC-273B12.10 | PSMG4 | 0.388 | 6.62E-20 |
| CTC-273B12.10 | CTU1 | 0.396 | 1.05E-20 |
| CTC-273B12.10 | HIST3H2A | 0.403 | 1.52E-21 |
| CTC-273B12.10 | PRR7 | 0.405 | 1.10E-21 |
| CTC-273B12.10 | CBX8 | 0.435 | 3.61E-25 |
| RP4-816N1.7 | GABARAP | -0.329 | 1.87E-14 |
| RP4-816N1.7 | RP11-903H12.5 | -0.32 | 1.03E-13 |
| RP4-816N1.7 | RELT | 0.303 | 2.37E-12 |
| RP4-816N1.7 | DRD4 | 0.303 | 2.39E-12 |
| RP4-816N1.7 | RP11-87C12.2 | 0.303 | 2.13E-12 |
| RP4-816N1.7 | ATAD3B | 0.304 | 1.78E-12 |
| RP4-816N1.7 | HYPK | 0.307 | 1.05E-12 |
| RP4-816N1.7 | HSPA6 | 0.308 | 9.60E-13 |
| RP4-816N1.7 | MAMDC4 | 0.308 | 8.92E-13 |
| RP4-816N1.7 | UQCRHL | 0.308 | 1.01E-12 |
| RP4-816N1.7 | WDR90 | 0.309 | 8.15E-13 |
| RP4-816N1.7 | RTEL1-TNFRSF6B | 0.31 | 6.16E-13 |
| RP4-816N1.7 | CLK2 | 0.311 | 5.65E-13 |
| RP4-816N1.7 | ALKBH6 | 0.312 | 4.53E-13 |
| RP4-816N1.7 | CCNL2 | 0.316 | 2.06E-13 |
| RP4-816N1.7 | WDR27 | 0.318 | 1.57E-13 |
| RP4-816N1.7 | EP400NL | 0.318 | 1.41E-13 |
| RP4-816N1.7 | LMNTD2 | 0.318 | 1.44E-13 |
| RP4-816N1.7 | TMEM120B | 0.321 | 8.44E-14 |
| RP4-816N1.7 | NFKBID | 0.321 | 9.12E-14 |
| RP4-816N1.7 | NPIPB5 | 0.322 | 7.69E-14 |
| RP4-816N1.7 | CTU1 | 0.323 | 5.87E-14 |
| RP4-816N1.7 | PABPC3 | 0.324 | 5.27E-14 |
| RP4-816N1.7 | PRSS53 | 0.325 | 4.31E-14 |
| RP4-816N1.7 | ATRIP | 0.328 | 2.29E-14 |
| RP4-816N1.7 | LRP5L | 0.328 | 2.27E-14 |
| RP4-816N1.7 | ZC3HAV1L | 0.331 | 1.24E-14 |
| RP4-816N1.7 | ATP2A1 | 0.336 | 4.95E-15 |
| RP4-816N1.7 | CTD-2192J16.22 | 0.341 | 1.80E-15 |
| RP4-816N1.7 | GJD3 | 0.342 | 1.38E-15 |
| RP4-816N1.7 | PIF1 | 0.343 | 1.36E-15 |
| RP4-816N1.7 | ENO3 | 0.344 | 9.72E-16 |
| RP4-816N1.7 | DPH7 | 0.345 | 7.52E-16 |
| RP4-816N1.7 | CTC-479C5.12 | 0.347 | 6.00E-16 |
| RP4-816N1.7 | MZF1 | 0.348 | 4.46E-16 |
| RP4-816N1.7 | HAGHL | 0.35 | 2.67E-16 |
| RP4-816N1.7 | TMEM262 | 0.352 | 1.88E-16 |
| RP4-816N1.7 | ARPC4-TTLL3 | 0.353 | 1.67E-16 |
| RP4-816N1.7 | LTB4R | 0.353 | 1.49E-16 |
| RP4-816N1.7 | KLHL17 | 0.356 | 8.48E-17 |
| RP4-816N1.7 | NACA2 | 0.358 | 5.69E-17 |
| RP4-816N1.7 | GPS2 | 0.362 | 2.55E-17 |
| RP4-816N1.7 | H3F3C | 0.369 | 5.17E-18 |
| RP4-816N1.7 | EIF4A1 | 0.391 | 3.29E-20 |
| RP4-816N1.7 | CCDC78 | 0.394 | 1.51E-20 |
| RP11-167H9.4 | SECTM1 | -0.378 | 6.62E-19 |
| RP11-167H9.4 | TOM1L2 | -0.362 | 2.21E-17 |
| RP11-167H9.4 | CDKN1A | -0.361 | 3.15E-17 |
| RP11-167H9.4 | GCNT3 | -0.349 | 3.35E-16 |
| RP11-167H9.4 | CCDC68 | -0.344 | 1.06E-15 |
| RP11-167H9.4 | ISG20 | -0.336 | 4.87E-15 |
| RP11-167H9.4 | CAMTA2 | -0.333 | 8.92E-15 |
| RP11-167H9.4 | DGKA | -0.332 | 1.11E-14 |
| RP11-167H9.4 | FRMD3 | -0.331 | 1.39E-14 |
| RP11-167H9.4 | MYO1C | -0.33 | 1.53E-14 |
| RP11-167H9.4 | LRP10 | -0.326 | 3.53E-14 |
| RP11-167H9.4 | ZBTB7C | -0.326 | 3.73E-14 |
| RP11-167H9.4 | SEMA4B | -0.323 | 5.87E-14 |
| RP11-167H9.4 | RNF125 | -0.323 | 6.50E-14 |
| RP11-167H9.4 | CTSE | -0.322 | 7.85E-14 |
| RP11-167H9.4 | NDEL1 | -0.322 | 7.37E-14 |
| RP11-167H9.4 | ALKBH5 | -0.322 | 7.78E-14 |
| RP11-167H9.4 | MT2A | -0.321 | 8.35E-14 |
| RP11-167H9.4 | ATP9B | -0.318 | 1.50E-13 |
| RP11-167H9.4 | SLC4A4 | -0.317 | 1.81E-13 |
| RP11-167H9.4 | CTDP1 | -0.316 | 2.33E-13 |
| RP11-167H9.4 | RHOF | -0.315 | 2.63E-13 |
| RP11-167H9.4 | CD209 | -0.313 | 3.73E-13 |
| RP11-167H9.4 | HIVEP3 | -0.311 | 5.05E-13 |
| RP11-167H9.4 | KIR2DL4 | -0.31 | 7.04E-13 |
| RP11-167H9.4 | LTBP4 | -0.309 | 8.50E-13 |
| RP11-167H9.4 | FAM189A2 | -0.309 | 7.45E-13 |
| RP11-167H9.4 | RELL1 | -0.309 | 7.97E-13 |
| RP11-167H9.4 | ANKFY1 | -0.309 | 7.56E-13 |
| RP11-167H9.4 | ZZEF1 | -0.308 | 8.72E-13 |
| RP11-167H9.4 | BARX2 | -0.308 | 9.07E-13 |
| RP11-167H9.4 | FLII | -0.307 | 1.12E-12 |
| RP11-167H9.4 | FECH | -0.306 | 1.35E-12 |
| RP11-167H9.4 | RAB27B | -0.306 | 1.33E-12 |
| RP11-167H9.4 | IL6R | -0.306 | 1.40E-12 |
| RP11-167H9.4 | KIAA1211 | -0.306 | 1.29E-12 |
| RP11-167H9.4 | PRDM8 | -0.303 | 2.08E-12 |
| RP11-167H9.4 | RNF152 | -0.303 | 2.35E-12 |
| RP11-167H9.4 | ATP2A3 | -0.303 | 2.09E-12 |
| RP11-167H9.4 | ATP5A1 | -0.302 | 2.63E-12 |
| RP11-167H9.4 | PLA2G2A | -0.301 | 3.33E-12 |
| RP11-167H9.4 | PLEKHG7 | 0.301 | 2.94E-12 |
| RP11-167H9.4 | GPR143 | 0.301 | 3.09E-12 |
| RP11-167H9.4 | HAUS7 | 0.301 | 3.42E-12 |
| RP11-167H9.4 | PSPH | 0.301 | 2.91E-12 |
| RP11-167H9.4 | DHX35 | 0.301 | 3.38E-12 |
| RP11-167H9.4 | IMMP1L | 0.302 | 2.57E-12 |
| RP11-167H9.4 | PRDX4 | 0.302 | 2.57E-12 |
| RP11-167H9.4 | NOL10 | 0.302 | 2.61E-12 |
| RP11-167H9.4 | DARS | 0.303 | 2.17E-12 |
| RP11-167H9.4 | NIP7 | 0.303 | 2.45E-12 |
| RP11-167H9.4 | DNAJC2 | 0.303 | 2.38E-12 |
| RP11-167H9.4 | GATC | 0.303 | 2.34E-12 |
| RP11-167H9.4 | CLDN1 | 0.303 | 2.23E-12 |
| RP11-167H9.4 | MFSD14C | 0.303 | 2.45E-12 |
| RP11-167H9.4 | SETMAR | 0.304 | 1.82E-12 |
| RP11-167H9.4 | CSE1L | 0.304 | 1.75E-12 |
| RP11-167H9.4 | CELF5 | 0.304 | 1.93E-12 |
| RP11-167H9.4 | AKNAD1 | 0.304 | 1.89E-12 |
| RP11-167H9.4 | ORC5 | 0.304 | 1.77E-12 |
| RP11-167H9.4 | MRPL48 | 0.304 | 1.90E-12 |
| RP11-167H9.4 | GPR160 | 0.304 | 1.94E-12 |
| RP11-167H9.4 | MKS1 | 0.304 | 1.90E-12 |
| RP11-167H9.4 | OGT | 0.304 | 1.98E-12 |
| RP11-167H9.4 | KLHL31 | 0.304 | 2.00E-12 |
| RP11-167H9.4 | RSL1D1 | 0.305 | 1.66E-12 |
| RP11-167H9.4 | TSGA10 | 0.305 | 1.72E-12 |
| RP11-167H9.4 | PHF14 | 0.305 | 1.54E-12 |
| RP11-167H9.4 | POC5 | 0.305 | 1.71E-12 |
| RP11-167H9.4 | GYG2 | 0.305 | 1.55E-12 |
| RP11-167H9.4 | PSMD10 | 0.306 | 1.24E-12 |
| RP11-167H9.4 | ZNF443 | 0.306 | 1.33E-12 |
| RP11-167H9.4 | ZNF19 | 0.306 | 1.37E-12 |
| RP11-167H9.4 | PTGES3L | 0.306 | 1.32E-12 |
| RP11-167H9.4 | NUPL2 | 0.307 | 1.08E-12 |
| RP11-167H9.4 | NANOS3 | 0.307 | 1.08E-12 |
| RP11-167H9.4 | SUN3 | 0.307 | 1.08E-12 |
| RP11-167H9.4 | OXGR1 | 0.307 | 1.18E-12 |
| RP11-167H9.4 | WDR77 | 0.307 | 1.15E-12 |
| RP11-167H9.4 | CCDC113 | 0.307 | 1.03E-12 |
| RP11-167H9.4 | NDUFAF2 | 0.308 | 9.99E-13 |
| RP11-167H9.4 | AMZ2 | 0.308 | 9.23E-13 |
| RP11-167H9.4 | ZNF66 | 0.308 | 9.97E-13 |
| RP11-167H9.4 | ANKFN1 | 0.308 | 8.77E-13 |
| RP11-167H9.4 | MPLKIP | 0.308 | 9.59E-13 |
| RP11-167H9.4 | GGH | 0.308 | 9.59E-13 |
| RP11-167H9.4 | HSPE1 | 0.309 | 8.16E-13 |
| RP11-167H9.4 | GNL3 | 0.309 | 8.40E-13 |
| RP11-167H9.4 | MOSPD1 | 0.309 | 7.83E-13 |
| RP11-167H9.4 | CEBPZOS | 0.309 | 8.27E-13 |
| RP11-167H9.4 | VAV3 | 0.309 | 7.68E-13 |
| RP11-167H9.4 | SLC25A14 | 0.31 | 6.40E-13 |
| RP11-167H9.4 | GGCT | 0.31 | 6.14E-13 |
| RP11-167H9.4 | GSPT2 | 0.31 | 6.19E-13 |
| RP11-167H9.4 | PPM1H | 0.311 | 5.07E-13 |
| RP11-167H9.4 | RBM39 | 0.311 | 5.13E-13 |
| RP11-167H9.4 | NR6A1 | 0.311 | 5.04E-13 |
| RP11-167H9.4 | CLNS1A | 0.311 | 5.63E-13 |
| RP11-167H9.4 | KCNH8 | 0.311 | 5.75E-13 |
| RP11-167H9.4 | AMER1 | 0.312 | 4.64E-13 |
| RP11-167H9.4 | YIPF6 | 0.312 | 4.51E-13 |
| RP11-167H9.4 | SPTY2D1-AS1 | 0.312 | 4.20E-13 |
| RP11-167H9.4 | RAE1 | 0.313 | 3.52E-13 |
| RP11-167H9.4 | ESRRG | 0.313 | 3.81E-13 |
| RP11-167H9.4 | USP27X | 0.313 | 3.79E-13 |
| RP11-167H9.4 | TNNC2 | 0.313 | 3.63E-13 |
| RP11-167H9.4 | METAP1D | 0.313 | 4.14E-13 |
| RP11-167H9.4 | ADNP | 0.313 | 4.09E-13 |
| RP11-167H9.4 | DSN1 | 0.313 | 3.59E-13 |
| RP11-167H9.4 | RHEB | 0.313 | 3.50E-13 |
| RP11-167H9.4 | TMEM89 | 0.313 | 4.05E-13 |
| RP11-167H9.4 | ZNF717 | 0.314 | 3.26E-13 |
| RP11-167H9.4 | DUS4L | 0.314 | 2.92E-13 |
| RP11-167H9.4 | FAM122B | 0.314 | 3.03E-13 |
| RP11-167H9.4 | TMLHE | 0.315 | 2.74E-13 |
| RP11-167H9.4 | RDH12 | 0.315 | 2.54E-13 |
| RP11-167H9.4 | UBE3D | 0.316 | 2.06E-13 |
| RP11-167H9.4 | WDR5B | 0.316 | 2.16E-13 |
| RP11-167H9.4 | RPP40 | 0.316 | 2.26E-13 |
| RP11-167H9.4 | NXT2 | 0.316 | 2.35E-13 |
| RP11-167H9.4 | MSL3 | 0.316 | 2.16E-13 |
| RP11-167H9.4 | TAMM41 | 0.317 | 1.96E-13 |
| RP11-167H9.4 | SMYD5 | 0.317 | 1.94E-13 |
| RP11-167H9.4 | TOMM34 | 0.318 | 1.52E-13 |
| RP11-167H9.4 | FAM104B | 0.318 | 1.61E-13 |
| RP11-167H9.4 | C1GALT1C1 | 0.318 | 1.65E-13 |
| RP11-167H9.4 | TTC32 | 0.318 | 1.48E-13 |
| RP11-167H9.4 | UTP14A | 0.318 | 1.47E-13 |
| RP11-167H9.4 | SS18L2 | 0.319 | 1.36E-13 |
| RP11-167H9.4 | CCDC88B | 0.319 | 1.38E-13 |
| RP11-167H9.4 | MRPS33 | 0.32 | 9.74E-14 |
| RP11-167H9.4 | HEMK1 | 0.32 | 1.14E-13 |
| RP11-167H9.4 | TMPRSS13 | 0.32 | 1.10E-13 |
| RP11-167H9.4 | RCL1 | 0.32 | 1.00E-13 |
| RP11-167H9.4 | ATAD3C | 0.321 | 8.95E-14 |
| RP11-167H9.4 | AK9 | 0.321 | 8.47E-14 |
| RP11-167H9.4 | DRD2 | 0.321 | 9.54E-14 |
| RP11-167H9.4 | ACTR5 | 0.322 | 7.46E-14 |
| RP11-167H9.4 | FAM192A | 0.322 | 7.51E-14 |
| RP11-167H9.4 | PUS7 | 0.322 | 7.66E-14 |
| RP11-167H9.4 | GAS2 | 0.322 | 6.90E-14 |
| RP11-167H9.4 | METTL6 | 0.323 | 6.06E-14 |
| RP11-167H9.4 | PUM3 | 0.323 | 5.69E-14 |
| RP11-167H9.4 | C12orf73 | 0.323 | 6.53E-14 |
| RP11-167H9.4 | RNF43 | 0.323 | 5.68E-14 |
| RP11-167H9.4 | TMEM192 | 0.324 | 5.38E-14 |
| RP11-167H9.4 | ZSWIM3 | 0.324 | 5.28E-14 |
| RP11-167H9.4 | TMEM38B | 0.324 | 5.37E-14 |
| RP11-167H9.4 | PGRMC1 | 0.324 | 5.00E-14 |
| RP11-167H9.4 | SLC7A6OS | 0.324 | 4.66E-14 |
| RP11-167H9.4 | NCBP2 | 0.324 | 5.31E-14 |
| RP11-167H9.4 | METTL5 | 0.325 | 4.53E-14 |
| RP11-167H9.4 | MYRIP | 0.325 | 4.37E-14 |
| RP11-167H9.4 | C11orf71 | 0.325 | 4.03E-14 |
| RP11-167H9.4 | TGIF2 | 0.326 | 3.49E-14 |
| RP11-167H9.4 | ACSL6 | 0.326 | 3.68E-14 |
| RP11-167H9.4 | POFUT1 | 0.327 | 2.66E-14 |
| RP11-167H9.4 | CDK5RAP1 | 0.327 | 3.06E-14 |
| RP11-167H9.4 | POLA1 | 0.327 | 3.09E-14 |
| RP11-167H9.4 | PFDN4 | 0.327 | 3.00E-14 |
| RP11-167H9.4 | GNG4 | 0.327 | 2.79E-14 |
| RP11-167H9.4 | RBMX2 | 0.327 | 2.83E-14 |
| RP11-167H9.4 | EIF1AX | 0.328 | 2.18E-14 |
| RP11-167H9.4 | COA5 | 0.328 | 2.19E-14 |
| RP11-167H9.4 | NARS2 | 0.328 | 2.37E-14 |
| RP11-167H9.4 | LY6G6E | 0.328 | 2.45E-14 |
| RP11-167H9.4 | RP9 | 0.328 | 2.31E-14 |
| RP11-167H9.4 | DHODH | 0.328 | 2.47E-14 |
| RP11-167H9.4 | METTL2B | 0.329 | 1.99E-14 |
| RP11-167H9.4 | FERMT1 | 0.33 | 1.51E-14 |
| RP11-167H9.4 | SLC6A6 | 0.33 | 1.49E-14 |
| RP11-167H9.4 | NKAP | 0.33 | 1.63E-14 |
| RP11-167H9.4 | UQCC1 | 0.33 | 1.50E-14 |
| RP11-167H9.4 | C2orf61 | 0.33 | 1.66E-14 |
| RP11-167H9.4 | PIR | 0.331 | 1.20E-14 |
| RP11-167H9.4 | KRBOX4 | 0.331 | 1.30E-14 |
| RP11-167H9.4 | BTBD16 | 0.331 | 1.41E-14 |
| RP11-167H9.4 | TMEM252 | 0.331 | 1.25E-14 |
| RP11-167H9.4 | MTMR8 | 0.331 | 1.27E-14 |
| RP11-167H9.4 | CAMKMT | 0.332 | 1.12E-14 |
| RP11-167H9.4 | 1-Dec | 0.332 | 1.05E-14 |
| RP11-167H9.4 | ZNF280B | 0.332 | 1.08E-14 |
| RP11-167H9.4 | FTCDNL1 | 0.332 | 1.15E-14 |
| RP11-167H9.4 | GPSM2 | 0.333 | 9.46E-15 |
| RP11-167H9.4 | CMSS1 | 0.333 | 8.89E-15 |
| RP11-167H9.4 | MRPS17 | 0.334 | 7.25E-15 |
| RP11-167H9.4 | ANKRD49 | 0.335 | 6.45E-15 |
| RP11-167H9.4 | USPL1 | 0.335 | 6.05E-15 |
| RP11-167H9.4 | IYD | 0.335 | 5.66E-15 |
| RP11-167H9.4 | PLCB4 | 0.336 | 5.01E-15 |
| RP11-167H9.4 | LAMP2 | 0.336 | 4.54E-15 |
| RP11-167H9.4 | VMA21 | 0.337 | 4.44E-15 |
| RP11-167H9.4 | MCMDC2 | 0.337 | 3.75E-15 |
| RP11-167H9.4 | PNO1 | 0.337 | 3.75E-15 |
| RP11-167H9.4 | MAGEB5 | 0.337 | 3.90E-15 |
| RP11-167H9.4 | APIP | 0.337 | 3.96E-15 |
| RP11-167H9.4 | TIGD1 | 0.338 | 3.55E-15 |
| RP11-167H9.4 | WWOX | 0.338 | 3.20E-15 |
| RP11-167H9.4 | ZNF182 | 0.338 | 3.68E-15 |
| RP11-167H9.4 | DDX27 | 0.338 | 3.09E-15 |
| RP11-167H9.4 | RCN1 | 0.338 | 3.53E-15 |
| RP11-167H9.4 | TRIM24 | 0.338 | 3.22E-15 |
| RP11-167H9.4 | C2orf15 | 0.339 | 2.74E-15 |
| RP11-167H9.4 | FUNDC2 | 0.339 | 2.72E-15 |
| RP11-167H9.4 | CXorf56 | 0.34 | 2.12E-15 |
| RP11-167H9.4 | GEMIN6 | 0.34 | 2.19E-15 |
| RP11-167H9.4 | OFD1 | 0.34 | 2.29E-15 |
| RP11-167H9.4 | CASK | 0.34 | 2.41E-15 |
| RP11-167H9.4 | LAS1L | 0.34 | 2.32E-15 |
| RP11-167H9.4 | SPACA3 | 0.34 | 2.18E-15 |
| RP11-167H9.4 | DPM1 | 0.341 | 1.73E-15 |
| RP11-167H9.4 | BRCC3 | 0.341 | 1.90E-15 |
| RP11-167H9.4 | ZNF3 | 0.341 | 1.80E-15 |
| RP11-167H9.4 | ENOX2 | 0.341 | 1.93E-15 |
| RP11-167H9.4 | TP53RK | 0.342 | 1.45E-15 |
| RP11-167H9.4 | ZNF502 | 0.342 | 1.66E-15 |
| RP11-167H9.4 | SPAG1 | 0.342 | 1.56E-15 |
| RP11-167H9.4 | RNF32 | 0.343 | 1.24E-15 |
| RP11-167H9.4 | SHROOM4 | 0.343 | 1.31E-15 |
| RP11-167H9.4 | FAAH2 | 0.344 | 9.74E-16 |
| RP11-167H9.4 | ARMC10 | 0.344 | 1.02E-15 |
| RP11-167H9.4 | RBM11 | 0.345 | 8.70E-16 |
| RP11-167H9.4 | WT1 | 0.346 | 6.42E-16 |
| RP11-167H9.4 | TXLNG | 0.347 | 5.80E-16 |
| RP11-167H9.4 | RLN2 | 0.347 | 5.82E-16 |
| RP11-167H9.4 | RP11-723O4.6 | 0.347 | 5.56E-16 |
| RP11-167H9.4 | RBBP7 | 0.348 | 4.79E-16 |
| RP11-167H9.4 | RPL36A | 0.349 | 3.77E-16 |
| RP11-167H9.4 | CCDC53 | 0.349 | 3.69E-16 |
| RP11-167H9.4 | KRT23 | 0.35 | 2.80E-16 |
| RP11-167H9.4 | MEST | 0.35 | 3.19E-16 |
| RP11-167H9.4 | APLF | 0.35 | 2.99E-16 |
| RP11-167H9.4 | RRAGB | 0.351 | 2.50E-16 |
| RP11-167H9.4 | DNAJC19 | 0.351 | 2.57E-16 |
| RP11-167H9.4 | C12orf66 | 0.351 | 2.48E-16 |
| RP11-167H9.4 | PIGU | 0.352 | 1.85E-16 |
| RP11-167H9.4 | ZNF563 | 0.352 | 1.79E-16 |
| RP11-167H9.4 | RIPPLY3 | 0.352 | 1.87E-16 |
| RP11-167H9.4 | NKRF | 0.353 | 1.49E-16 |
| RP11-167H9.4 | CTTNBP2 | 0.353 | 1.61E-16 |
| RP11-167H9.4 | TTLL2 | 0.353 | 1.52E-16 |
| RP11-167H9.4 | CCDC170 | 0.353 | 1.58E-16 |
| RP11-167H9.4 | TRMT2B | 0.353 | 1.69E-16 |
| RP11-167H9.4 | LY6G6F | 0.354 | 1.36E-16 |
| RP11-167H9.4 | RPIA | 0.354 | 1.16E-16 |
| RP11-167H9.4 | CTPS2 | 0.354 | 1.31E-16 |
| RP11-167H9.4 | DDX10 | 0.356 | 8.68E-17 |
| RP11-167H9.4 | XXbac-BPG32J3.19 | 0.356 | 8.94E-17 |
| RP11-167H9.4 | LY6G6D | 0.356 | 9.21E-17 |
| RP11-167H9.4 | PLAC1 | 0.356 | 9.29E-17 |
| RP11-167H9.4 | RNF114 | 0.357 | 6.88E-17 |
| RP11-167H9.4 | WFDC10A | 0.357 | 7.11E-17 |
| RP11-167H9.4 | GRPR | 0.357 | 6.11E-17 |
| RP11-167H9.4 | DKC1 | 0.358 | 5.06E-17 |
| RP11-167H9.4 | MCTS1 | 0.358 | 5.13E-17 |
| RP11-167H9.4 | ERP27 | 0.358 | 4.99E-17 |
| RP11-167H9.4 | SCML2 | 0.358 | 5.66E-17 |
| RP11-167H9.4 | C9orf43 | 0.359 | 3.99E-17 |
| RP11-167H9.4 | GEMIN8 | 0.363 | 1.81E-17 |
| RP11-167H9.4 | FABP6 | 0.363 | 1.75E-17 |
| RP11-167H9.4 | TSEN2 | 0.364 | 1.52E-17 |
| RP11-167H9.4 | ATP6V1C2 | 0.364 | 1.47E-17 |
| RP11-167H9.4 | FUNDC1 | 0.365 | 1.33E-17 |
| RP11-167H9.4 | TIGD4 | 0.365 | 1.35E-17 |
| RP11-167H9.4 | PIN4 | 0.366 | 9.00E-18 |
| RP11-167H9.4 | PAAF1 | 0.367 | 8.68E-18 |
| RP11-167H9.4 | C3orf67 | 0.367 | 7.54E-18 |
| RP11-167H9.4 | LRRC2 | 0.368 | 5.96E-18 |
| RP11-167H9.4 | SLC13A3 | 0.369 | 5.08E-18 |
| RP11-167H9.4 | ELF5 | 0.369 | 4.62E-18 |
| RP11-167H9.4 | FGGY | 0.37 | 3.80E-18 |
| RP11-167H9.4 | ZNF202 | 0.372 | 2.74E-18 |
| RP11-167H9.4 | HILPDA | 0.372 | 2.88E-18 |
| RP11-167H9.4 | TSPAN6 | 0.377 | 8.81E-19 |
| RP11-167H9.4 | NIT2 | 0.377 | 8.88E-19 |
| RP11-167H9.4 | ZDHHC9 | 0.377 | 8.47E-19 |
| RP11-167H9.4 | SLC25A26 | 0.377 | 8.38E-19 |
| RP11-167H9.4 | SCML1 | 0.379 | 4.73E-19 |
| RP11-167H9.4 | PAH | 0.381 | 3.61E-19 |
| RP11-167H9.4 | ASB9 | 0.384 | 1.87E-19 |
| RP11-167H9.4 | KIAA1257 | 0.385 | 1.38E-19 |
| RP11-167H9.4 | ARMCX5 | 0.386 | 1.02E-19 |
| RP11-167H9.4 | C11orf1 | 0.386 | 1.15E-19 |
| RP11-167H9.4 | HDAC8 | 0.386 | 1.07E-19 |
| RP11-167H9.4 | HOGA1 | 0.387 | 8.54E-20 |
| RP11-167H9.4 | XKRX | 0.387 | 9.11E-20 |
| RP11-167H9.4 | LSM8 | 0.388 | 7.26E-20 |
| RP11-167H9.4 | POU5F1B | 0.392 | 2.43E-20 |
| RP11-167H9.4 | C20orf196 | 0.393 | 1.79E-20 |
| RP11-167H9.4 | TDGF1 | 0.393 | 1.89E-20 |
| RP11-167H9.4 | SMIM4 | 0.396 | 1.07E-20 |
| RP11-167H9.4 | YAE1D1 | 0.4 | 3.49E-21 |
| RP11-167H9.4 | CTSV | 0.403 | 1.63E-21 |
| RP11-167H9.4 | IMMP2L | 0.405 | 1.08E-21 |
| RP11-167H9.4 | TIMM8A | 0.409 | 4.27E-22 |
| RP11-167H9.4 | PMFBP1 | 0.409 | 3.92E-22 |
| RP11-167H9.4 | JADE3 | 0.41 | 3.23E-22 |
| RP11-167H9.4 | IZUMO2 | 0.417 | 4.73E-23 |
| RP11-167H9.4 | MAGEB17 | 0.418 | 4.06E-23 |
| RP11-167H9.4 | LRRC36 | 0.419 | 2.82E-23 |
| RP11-167H9.4 | KCNK9 | 0.422 | 1.44E-23 |
| RP11-400N13.2e99000228437 | MAOAe99000189221 | -0.34 | 2.23E-15 |
| RP11-400N13.2e99000228437 | B3GNT8e99000177191 | -0.337 | 3.81E-15 |
| RP11-400N13.2e99000228437 | MEP1Ae99000112818 | -0.329 | 1.98E-14 |
| RP11-400N13.2e99000228437 | ACOX2e99000168306 | -0.316 | 2.34E-13 |
| RP11-400N13.2e99000228437 | TBX10e99000167800 | -0.305 | 1.67E-12 |
| RP11-400N13.2e99000228437 | PITPNC1e99000154217 | 0.301 | 3.36E-12 |
| RP11-400N13.2e99000228437 | RIPK2e99000104312 | 0.306 | 1.22E-12 |
| RP11-400N13.2e99000228437 | SERPINB9e99000170542 | 0.306 | 1.22E-12 |
| RP11-400N13.2e99000228437 | FRMD5e99000171877 | 0.314 | 3.37E-13 |
| RP11-400N13.2e99000228437 | RAP2Be99000181467 | 0.317 | 1.84E-13 |
| RP11-400N13.2e99000228437 | SLCO1B3e99000111700 | 0.326 | 3.24E-14 |
| RP11-400N13.2e99000228437 | FOSL1e99000175592 | 0.33 | 1.72E-14 |
| RP11-400N13.2e99000228437 | SERPINB5e99000206075 | 0.347 | 5.97E-16 |
| RP11-400N13.2e99000228437 | RPE65e99000116745 | 0.368 | 6.32E-18 |
| RP11-400N13.2e99000228437 | NPSR1e99000187258 | 0.383 | 1.89E-19 |
| RP11-400N13.2e99000228437 | DUSP10e99000143507 | 0.389 | 5.20E-20 |
| RP11-400N13.2e99000228437 | CALB1e99000104327 | 0.448 | 1.08E-26 |
